# Biological underpinnings of radiomic magnetic resonance imaging phenotypes for risk stratification in IDH wild-type glioblastoma

**DOI:** 10.1186/s12967-023-04551-3

**Published:** 2023-11-22

**Authors:** Fangzhan Guan, Zilong Wang, Yuning Qiu, Yu Guo, Dongling Pei, Minkai Wang, Aoqi Xing, Zhongyi Liu, Bin Yu, Jingliang Cheng, Xianzhi Liu, Yuchen Ji, Dongming Yan, Jing Yan, Zhenyu Zhang

**Affiliations:** 1 Department of Neurosurgery, The First Affiliated Hospital of Zhengzhou University, Zhengzhou, 450052 Henan China; 2 Academy of Medical Sciences, Zhengzhou University, Zhengzhou, 450001 Henan China; 3 Department of MRI, The First Affiliated Hospital of Zhengzhou University, Zhengzhou, 450052 Henan China

**Keywords:** IDH wild-type glioblastoma, Conventional sequences, Machine learning, Biological underpinnings, Survival

## Abstract

**Background:**

To develop and validate a conventional MRI-based radiomic model for predicting prognosis in patients with IDH wild-type glioblastoma (GBM) and reveal the biological underpinning of the radiomic phenotypes.

**Methods:**

A total of 801 adult patients (training set, N = 471; internal validation set, N = 239; external validation set, N = 91) diagnosed with IDH wild-type GBM were included. A 20-feature radiomic risk score (Radscore) was built for overall survival (OS) prediction by univariate prognostic analysis and least absolute shrinkage and selection operator (LASSO) Cox regression in the training set. GSEA and WGCNA were applied to identify the intersectional pathways underlying the prognostic radiomic features in a radiogenomic analysis set with paired MRI and RNA-seq data (N = 132). The biological meaning of the conventional MRI sequences was revealed using a Mantel test.

**Results:**

Radscore was demonstrated to be an independent prognostic factor (P < 0.001). Incorporating the Radscore into a clinical model resulted in a radiomic-clinical nomogram predicting survival better than either the Radscore model or the clinical model alone, with better calibration and classification accuracy (a total net reclassification improvement of 0.403, P < 0.001). Three pathway categories (proliferation, DNA damage response, and immune response) were significantly correlated with the prognostic radiomic phenotypes.

**Conclusion:**

Our findings indicated that the prognostic radiomic phenotypes derived from conventional MRI are driven by distinct pathways involved in proliferation, DNA damage response, and immunity of IDH wild-type GBM.

**Supplementary Information:**

The online version contains supplementary material available at 10.1186/s12967-023-04551-3.

## Introduction

Glioblastoma (GBM) accounts for 50.1% of primary malignant brain tumors, with a median survival rate of 8 months and 5-year survival rate of 6.9% [1]. The current consensus treatment for GBM is maximal resection followed by chemoradiotherapy and chemotherapy; however, the median survival is still less than 15 months [2]. The poor prognosis of patients with GBM is partly attributed to intratumoral heterogeneity, which is reflected in complex mutations in genes and disordered biological pathways [3]. Isocitrate dehydrogenase (IDH) mutation have been established as a key prognostic factor and the pivotal molecular diagnostic marker for adult-type diffuse gliomas [4, 5]. Recently, several studies have shown that patients with IDH wild-type GBM have heterogeneous clinical outcomes [6–8]. Therefore, prognostic markers stratifying patients with IDH wild-type GBM are beneficial for guiding tumor management and informing personalized treatments.

Radiomics can non-invasively quantify tumor phenotypes by converting visual medical images into robust sub-visual digital indicators [9], and is a promising imaging biomarker in several GBM studies [10–12]. Radiogenomics, a rapidly booming field, provides biological interpretability to the data-driven nature of radiomics. Recent radiogenomics studies have uncovered the association between radiomics signatures and biological underpinnings in GBM [13–15]. However, previous radiogenomic studies have revealed the biological pathways behind radiomic features using either gene set enrichment analysis (GSEA) or weighted gene coexpression network analysis (WGCNA) methods [13, 14]. However, the focus of the two methods is different. The goal of GSEA is to determine whether genes in a gene set tend to appear enriched at the top or bottom of a preordered gene list [16], while WGCNA focuses on finding collections (modules) of genes that are synergistically expressed (with consistent trends of variation) in the overall genes [17]. Koyama et al. [18] identified the same immune-related processes using both GSEA and WGCNA methods in their study. However, few studies have investigated the biological pathways underlying radiomic phenotypes using both GSEA and WGCNA in radiogenomic analysis. Leveraging both GSEA and WGCNA to obtain the intersectional pathways for biological interpretation of the radiomics phenotypes will be more convincing with increase reproducibility and robustness.

Conventional MRI sequences have been extensively investigated in radiomics because of their accessibility and widespread use [13, 14, 19]. Different conventional MRI sequences are related to explicit tumor imaging morphologies in gliomas [20]. In particular, radiomic signatures derived from single conventional MRI sequences exhibit an excellent diagnostic value in gliomas. For example, Chen et al. [21] developed a radiomics-based model derived from the T1c sequence to differentiate gliomas from brain metastases, with an area under the ROC curve (AUC) of 0.80. Li et al. [22] confirmed that radiomic features based on the T2 FLAIR sequence can predict the expression of Ki-67, S-100, wave proteins, and CD34. However, the biological basis of each MRI sequence remains elusive, and adequate biological evidence is lacking for single-sequence radiomics applications and promotion.

Therefore, the current study has two objectives. First, a prognostic radiomic risk score (Radscore) was constructed and validated to stratify the patients with IDH wild-type GBM. Second, radiogenomic analysis utilized intersectional pathways enriched by both GSEA and WGCNA to explore the biological underpinnings of prognostic radiomic phenotypes.

## Materials and methods

### Study design

**Fig. 1:**
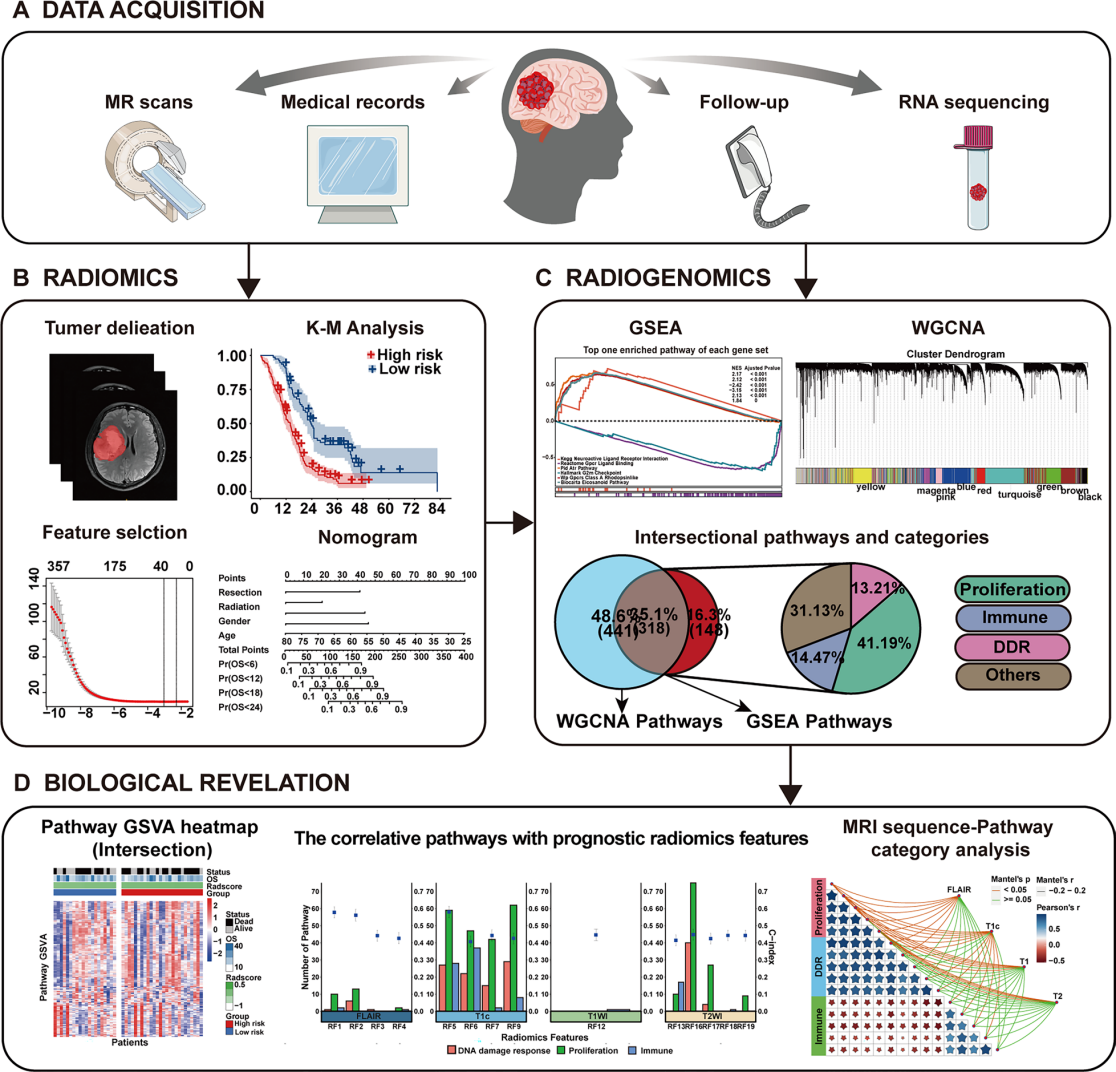
Workflow of this study. **A** Data acquisition. **B** Radiomic model construction and validation. **C** Radiogenomics analysis: using both GSEA and WGCNA methods to reveal the biological basis behind radiomics, and obtained the intersectional pathways. **D** The intersectional pathways of the two approaches was used to explore the biological basis behind individual prognostic features and sequences

This study was a part of the ongoing research of the registered clinical trial “MR Based Survival Prediction of Glioma Patients Using Artificial Intelligence” (WHO International Clinical Trial Registry Platform: ClinicalTrials.gov, Trial registration ID: NCT04215211). The Human Scientific Ethics Committee of the First Affiliated Hospital of Zhengzhou University (FAHZZU) and Henan Provincial People’s Hospital (HPPH) approved the study, and the requirement for written informed consent was waived due to the retrospective nature of this analysis. For patients providing fresh tumor specimens, informed consent was obtained. The study framework is shown in Fig. 1 includes two parts: radiomics profiling and radiogenomics analysis. First, an MRI-based Radscore was developed for predicting overall survival based on a training set and validated on an internal and external validation set. Then, based on the radiogenomics analysis set with both MRI and RNA-seq, two methods were used to identify the driving pathways underlying the prognostic Radscore: WGCNA and GSEA. Third, the intersectional pathways of the two methods were used to annotate prognostic radiomic phenotypes.

### Study cohorts

This study collected information on patients with histologically confirmed IDH wild-type GBM from FAHZZU and HPPH, between January 2011 and December 2021. Our study cohort (n = 801) had three sets: (1) a radiomics analysis set (n = 710, from FAHZZU) with preoperative conventional MRI sequences including T2-weighted fluid-attenuated inversion recovery, T1-weighted gadolinium contrast-enhanced, T1-weighted, and T2-weighted images (FLAIR, T1c, T1, and T2) for developing and validating the prognostic Radscore; (2) an external validation set (n = 91, from HPPH) with preoperative MRI (FLAIR, T1c, T1, and T2) to externally validate the reproducibility of the prognostic radiomic model; and (3) a radiogenomics analysis set (n = 132, from FAHZZU) with paired MRI and RNA-seq to identify biological pathways underlying the radiomic features, which is a subset of the radiomics analysis set. Specifically, patients in the radiomics analysis set were randomly divided into a training data set (n = 471) and an internal validation set (n = 239), where clinical parameters including sex, age, preoperative Karnofsky performance status (KPS) scale, extent of resection, and adjuvant therapies were balanced. The selection procedure is shown in Additional file 1: Fig. S1.

### Image acquisition, image processing and tumor delineation

This study followed image biomarker standardization initiative (IBSI) guidelines [23, 24]. All the necessary details are presented in Additional file 1: Table S2 to ensure the robustness of the extracted features. The modalities and parameters of the MRI are consistent between the external validation set and the radiomics analysis set and adhere to rules in Additional file 1: A1 and Table S2. First, N4ITK-based bias field distortion correction preprocessing to achieve image standardization. Subsequently, all voxels were isotropically resampled into 1 × 1 × 1 mm^3^ voxels using trilinear interpolation. Rigid registration was performed on the MRI for each patient using axial resampled T1c as a template with a mutual information similarity metric, generating the registered images, namely rFLAIR, rT1c, rT1, and rT2. Histogram matching was performed to normalize intensity distributions. The whole tumor area (including enhancing area, non-enhancing area, and necrosis, if any) was delineated as the signal abnormal regions in the white matter on rFLAIR images, whereas rT2w and rT1c images were used to cross-check the extension of the whole tumor areas. A neuroradiologist (J.Y.) with 12 years of experience, who was blinded to the clinical data, manually delineated the tumor contours section by section on transverse sections using open-source software (ITK-SNAP, version 3.8.0; http://www.itk-snap.org). To select robust features against intra-rater and inter-rater variations, the delineation was repeated by the same neuroradiologist (J.Y.) and another neuroradiologist (Z.Y.Z, 12 years of experience) on 100 randomly selected patients, yielding an intra-rater repeatability test dataset and an inter-rater data set, respectively.

### Radiomic feature extraction

Based on this delineation, we extracted 4746 IBSI-based features using PyRadiomics (version 3.0) within the quantitative image feature pipeline from all four MRI sequences. The extracted features included shape features, first-order intensity features, and higher-order texture features. All the extracted features are summarized in Additional file 1: Table S1.

### Statistical analysis

#### Radiomic model construction and validation

The radiomics analysis set was divided into a radiomics training subset (n = 471) and an internal validation subset (n = 239), using stratified random sampling at a ratio of 2:1 with balanced patient characteristics. A three-stage feature selection approach was used. First, to enhance the robustness of the features, any feature with an intraclass correlation coefficient (ICC) < 0.85 is discarded. Second, we selected the features that were highly correlated with OS. The remaining features with univariate concordance index (C-index) ≥ 0.55 (positive correlation) or ≤ 0.45 (negative correlation) were selected as better prognostic variables for further analysis. Third, least absolute shrinkage and selection operator (LASSO) penalized Cox proportional hazards regression [25] was used on the training set to select the optimal feature subset. Finally, a Radscore based on 20 features (denoted as RF1–RF20 in Additional file 1: Fig. S2) was developed. A Radscore-based cutoff value calculated with the R package “survminer” divides the training set into high- and low-risk groups and is subsequently applied to the validation set. The association between Radscore and survival was evaluated using Kaplan–Meier analysis. C-index was calculated to measure the discrimination performance of the model. A calibration curve was used to assess the agreement between the predicted and observed results in the model and a decision curve was used to measure the clinical usefulness of the model. Net Reclassification Improvement (NRI) and Akaike Information Criterion (AIC) were used to assess the improved performance and potential risk of overfitting in the model, respectively.

#### Radiogenomics analysis

Based on the radiogenomics analysis set with both MRI and RNA-seq, the biological pathways underlying Radscore were identified using GSEA and WGCNA.

#### GSEA analysis for driving pathways identification

First, differentially expressed genes (DEGs) between the high- and low-risk subgroups stratified by Radscore were identified using the R package DESeq2. Then, the values of Log_2_FoldChange for each gene were arranged in reverse order and used to enrich the overrepresented pathways with an R package clusterPro-filer based on six annotated genes: Kyoto Encyclopedia of Genes and Genomes (KEGG), Hallmark, Reactome, BioCarta, Pathway Interaction Database (PID), and WikiPathways (WP). False-discovery rate (FDR)-corrected P < 0.05 was considered as significant enrichment. Thereafter, a gene set variation analysis (GSVA) was used on each enriched pathway to quantify its activity [26]. Pearson correlation was performed to assess if the pathway GSVA score was significantly associated (FDR < 0.01) with the Radscore. Finally, the significantly correlated pathways were selected for further analysis.

#### WGCNA analysis for driving pathways identification

First, WGCNA was performed on the radiogenomics analysis set to cluster highly interconnected genes into a few gene modules that may be involved in common biological processes [17]. Modules significantly associated with the Radscore were identified based on a sample-based GSVA, where FDR < 0.01 indicated significance. The detailed process of WGCNA is described in Fig. 3A, B. Within each Radscore-associated module, enrichment analyses were performed for KEGG, Hallmark, Reactome, BioCarta, PID, and WP using the R package clusterProfiler [27]. Finally, significantly correlated pathways (FDR < 0.01) were identified.

#### Biological pathways underlying Radiomic phenotypes

First, the intersectional pathways of GSEA and WGCNA were identified to improve the biological reliability of the enriched pathways. The identified pathways were categorized into several types. A Pearson correlation was performed on prognostic radiomic features and intersection pathways, where FDR < 0.05 indicated significance. The top feature-associated pathways of each pathway category were selected for further exploration. Finally, a Mantel test was used to assess the potential associations between the pathway categories and MRI sequences using ‘vegan’ R package.

## Results

### Patients’ characteristics

In total, 801 patients were included in this study. The distribution of clinical characteristics was balanced between the training and validation sets (chi-square or Wilcoxon p-value > 0.05). The demographic and clinical information of the study cohort is summarized in Additional file 1: Table S3.

### Radiomic model construction and validation

#### Radiomic model construction

After the three-stage feature selection, 20 features remained. Based on the LASSO COX regression model (Additional file 1: Figs. S3, S4), Radscore is calculated according to the following formula: Radscore = 0.0312141·RF1 + 0.0862303·RF2 − 0.0182051·RF3 − 0.0329717·RF4 + 0.0145359·RF5 − 0.0302801·RF6 − 0.0597537·RF7 − 0.0195800·RF8 − 0.0079536·RF9 − 0.0010230·RF10 − 0.0620081·RF11 − 0.0130946·RF12 − 0.065238·RF13 + 0.0197421·RF14 + 0.0004487·RF15 − 0.0020062·RF16 − 0.0599923·RF17 − 0.0440648·RF18 − 0.0590665·RF19 − 0.0581274·RF20. The optimal Radscore cutoff was − 0.1026. Patients were categorized into a high-risk group (Radscore of at least − 0.1026) and low-risk group (Radscore less than − 0.1026).

#### Radiomic model validation

**Fig. 2:**
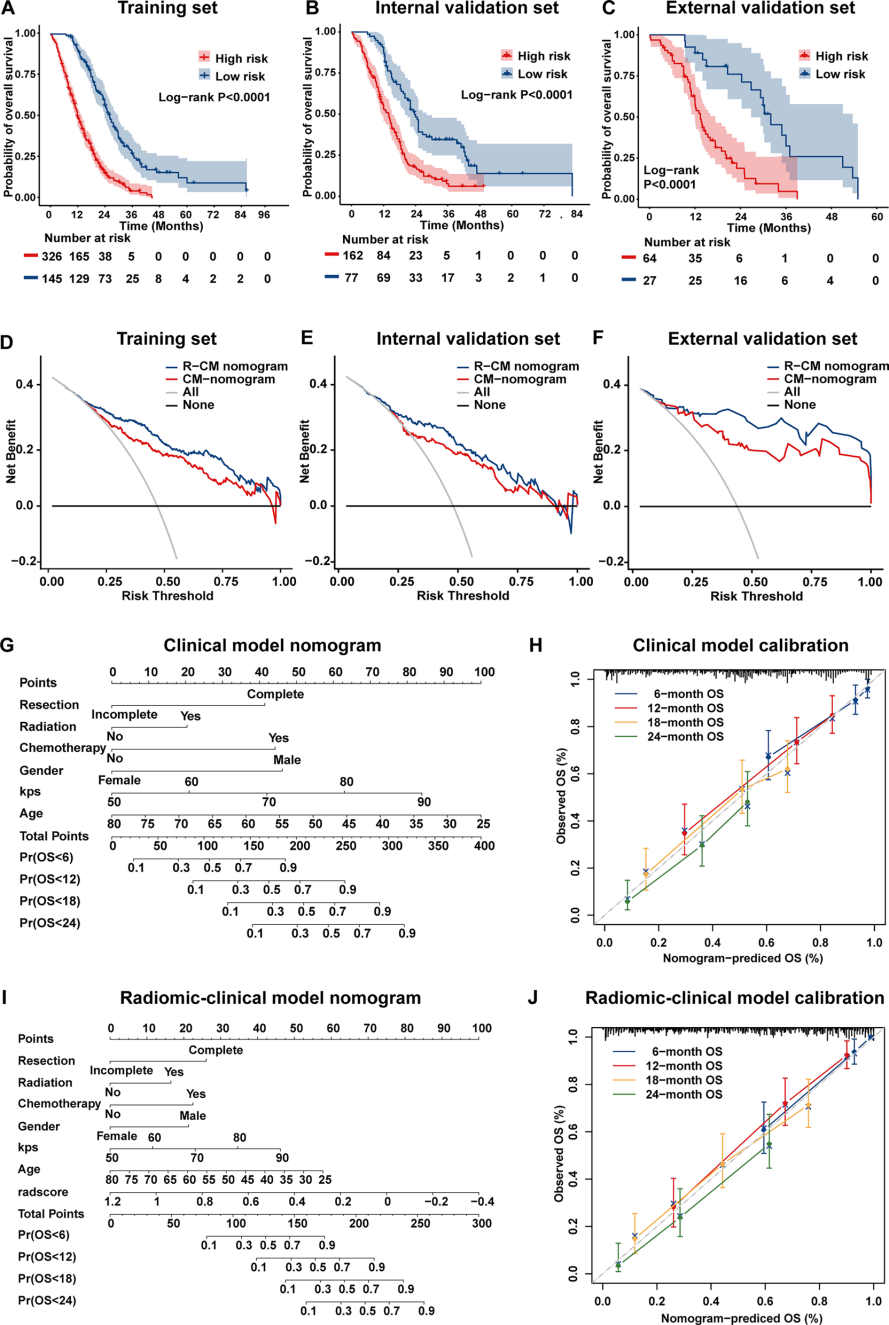
Validation of the radiomic signature. **A**–**C** Kaplan–Meier curves for patients stratified by the Radscore (cutoff = − 0.1026) in the training set (**A**), internal validation set (**B**) and external validation set (**C**). **D**–**F** Decision curve analysis (DCA) for radiomic-clinical model nomogram and clinical model nomogram to estimate the OS in the training set (**D**), internal validation set (**E**) and external validation set (**F**). The x-axis represents the threshold probability and the y-axis measures the net benefit. **G**–**J** The clinical model nomogram (**G**) and the radiomic-clinical model nomogram (**I**) for predicting the 6-, 12-, 18-, and 24-month OS, along with the calibration curves for assessment of the clinical model nomogram (**H**) and the radiomic-clinical model nomogram (**J**)

Association of Radscore with OS was found in the training set (log-rank P < 0.001; hazard ratio [HR] = 18.55, 95% CI 11.78, 29.23) and further confirmed in the internal validation set (log-rank P < 0.001; hazard ratio [HR] = 15.68, 95% CI 8.01, 30.68) and external validation sets (log-rank P < 0.001; hazard ratio [HR] = 12.84, 95% CI 5.31, 31.04), as shown by the Kaplan–Meier curves in Fig. 2A–C, respectively. The clinical modal (CM) nomogram and radiomic-clinical modal (R-CM) nomograms are shown in Fig. 2G, I. The C-index and AIC values for the three models are summarized in Additional file 1: Table S4. With lower AIC values, the C-index of the R-CM nomogram improved by 0.047, 0.058, and 0.054 for the training, internal, and external validation sets, respectively, compared with the CM nomogram. The calibration curve of the R-CM nomogram demonstrated better agreement between the predicted and observed survival for the probability of 6-, 12-, 18-, and 24-month deaths, as shown in Fig. 2H, J. Similarly, a total NRI of 0.403 (95% CI:0.308,0.473, p < 0.001) on the R-CM nomogram indicated improved classification performance. The decision curves (Fig. 2D–F) indicated that the R-CM nomogram was more beneficial than the CM nomogram alone. Multivariate Cox analysis revealed that the Radscore was an independent risk factor (HR = 39.998; 95% CI 12.803, 124.962; P < 0.001).

### GSEA analysis for driving pathways identification

**Fig. 3:**
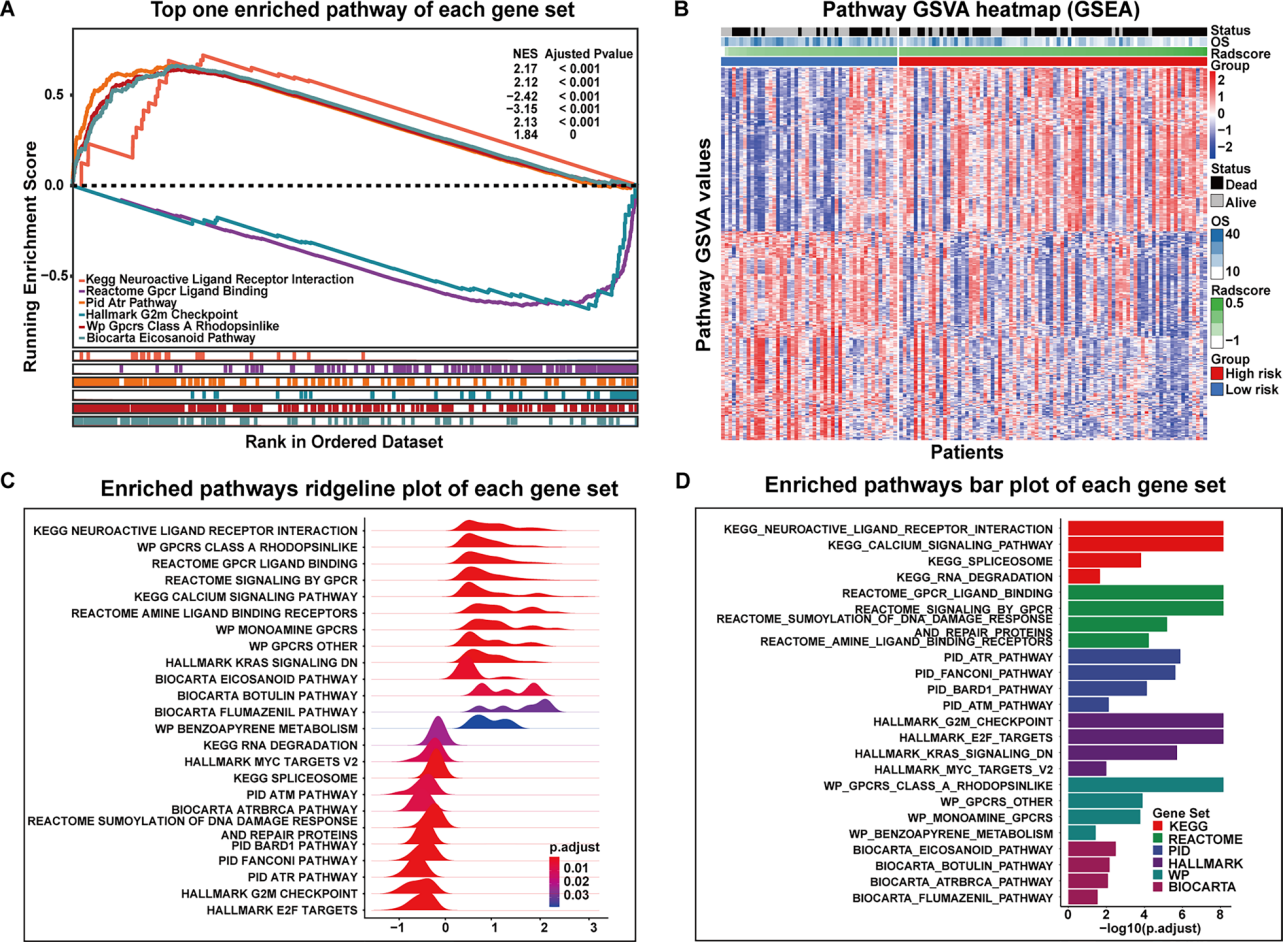
Results of gene set enrichment analysis. **A** Six representative pathways showing the most significantly enriched one from Kyoto Encyclopedia of the Genome (KEGG), Hallmark, Reactome, BioCarta, Pathway Interaction Database (PID), and WikiPathways sorted by enriched FDRs. **B** A heatmap of the distribution of clinical features and gene set variation analysis (GSVA) scores of GSEA enrichment-related pathways in 132 patients in the radiogenomics analysis set. After performing a pearson correlation, 466 pathways were selected as prognostically relevant (FDR < 0.01) and clustered according to their specific function. Patients were ranked according to their radscore, and pathway activity (GSVA values) demonstrated differences between high and low risk groups. **C** The Ridgeline plot shows 16 representative pathways (according to FDR) originating from 6 datasets were selected to demonstrate the distribution of enriched pathways between different datasets and the distribution of correlations with radscore. **D** Bar plot shows the FDR values for the top four pathways enriched by each gene set

Based on the radiogenomics analysis set, 646 pathways were enriched by GSEA analysis, where FDR < 0.05 was considered as significant enrichment. Then, 466 derived from the 646 pathways were obtained after performing a Pearson correlation between the enriched pathways and the Radscore, where FDR < 0.01 was considered as a significant association. A complete list of radscore-related pathways is provided in Additional file 1: Table S5, and a heatmap is shown in Fig. 3B. The top enriched pathways in each gene set are shown in Fig. 3A, C and D, respectively.

### WGCNA analysis for driving pathways identification

**Fig. 4:**
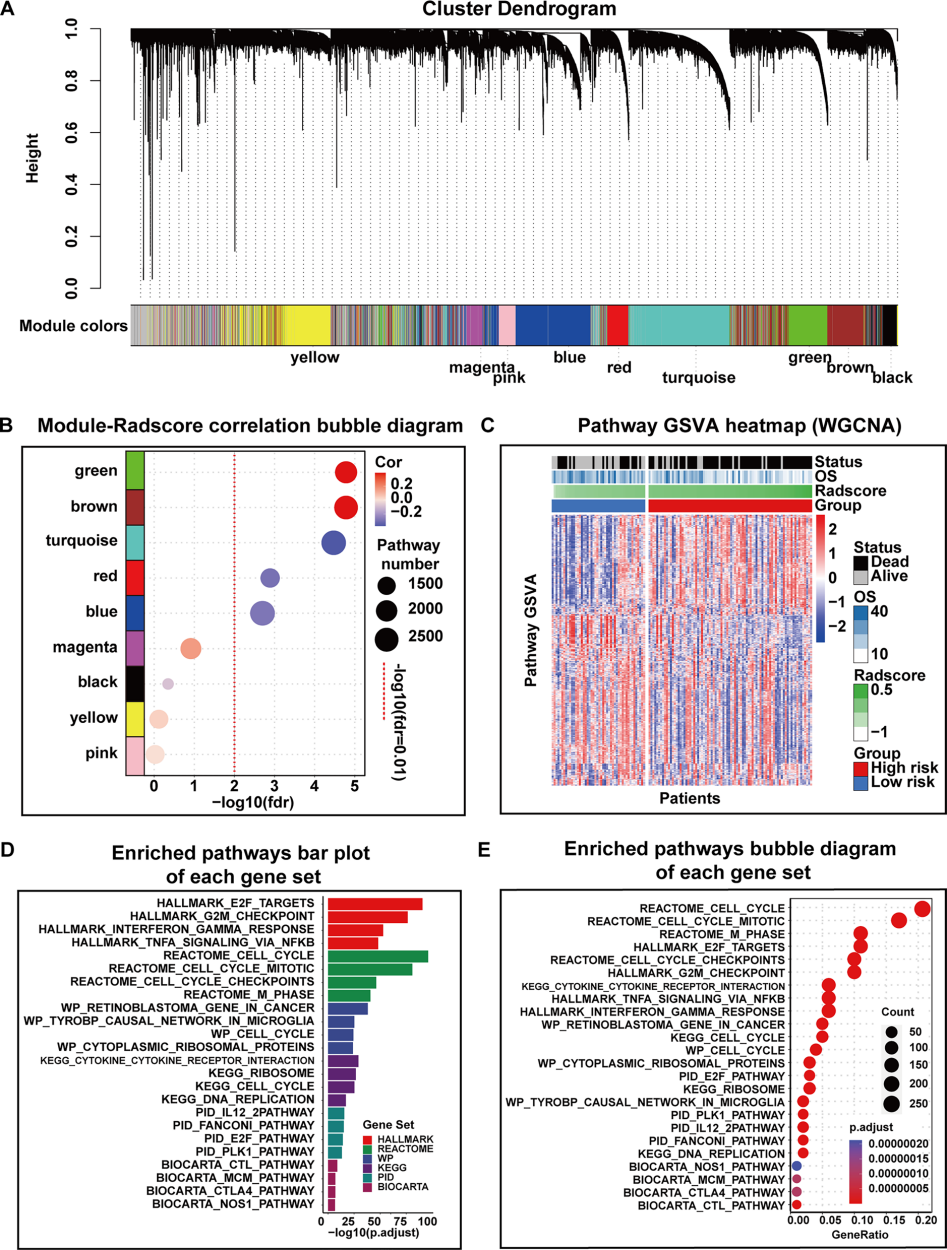
Results of weighted gene coexpression network analysis. **A** Clustering dendrogram of identified coexpression gene modules in the radiogenomics analysis set represented in the color-coded row. **B** Bubble charts show the results of GSVA values and radscore correlation of the 9 modules, FDR less than 0.01 is considered as significant correlation of modules with radscore (modules on the right side of the red line). **C** A heatmap of the distribution of clinical features and gene set variation analysis (GSVA) scores of modules enrichment-related pathways in 132 patients in the radiogenomics analysis set. After performing a Pearson correlation, 759 pathways were selected as prognostically relevant (FDR < 0.01) and clustered according to their specific function. Patients were ranked according to their radscore, and pathway activity (GSVA values) demonstrated differences between high and low risk groups. **D** Bar plot shows the FDR values for the top four pathways enriched by each gene set. **E** Bubble diagram showing enriched pathways count and generation for 16 representative pathways (according to FDR) from 6 gene sets

The results of WGCNA are summarized in Fig. 4A. Nine gene modules were derived based on the radiogenomic analysis set. In order to obtain MRI-related modules, we calculated the module GSVA values for each patient and subsequently did a pearson correlation with its corresponding Radscore. Five (turquoise module, 3722 genes; blue module, 3186 genes; brown module, 2820 genes; green module, 2,031 genes; red module, 884 genes; as listed in Additional file 1: Table S6) of the nine modules were correlated with Radscore (Pearson correlation r = − 0.37, FDR < 0.01 for turquoise module; Pearson correlation r = − 0.28, FDR < 0.01 for blue module; Pearson correlation r = 0.39, FDR < 0.01 for brown module; Pearson correlation r = 0.39, FDR < 0.01 for green module; Pearson correlation r = − 0.30, FDR < 0.01 for red module). The module-selection process is illustrated in Fig. 4B. A pathway enrichment analysis was performed using the selected modules. After controlling for FDR less than 0.01, 759 enriched pathways were identified, which were considered to have a significant correlation with the Radscore. A complete list of radscore-related pathways is provided in Additional file 1: Table S7. A GSVA heatmap of the enriched pathways is shown in Fig. 4C. The top enriched pathways in each gene set are shown in Fig. 4D, E.

### Biological pathways underlying radiomic phenotypes

#### Identification of the intersectional pathways

**Fig. 5:**
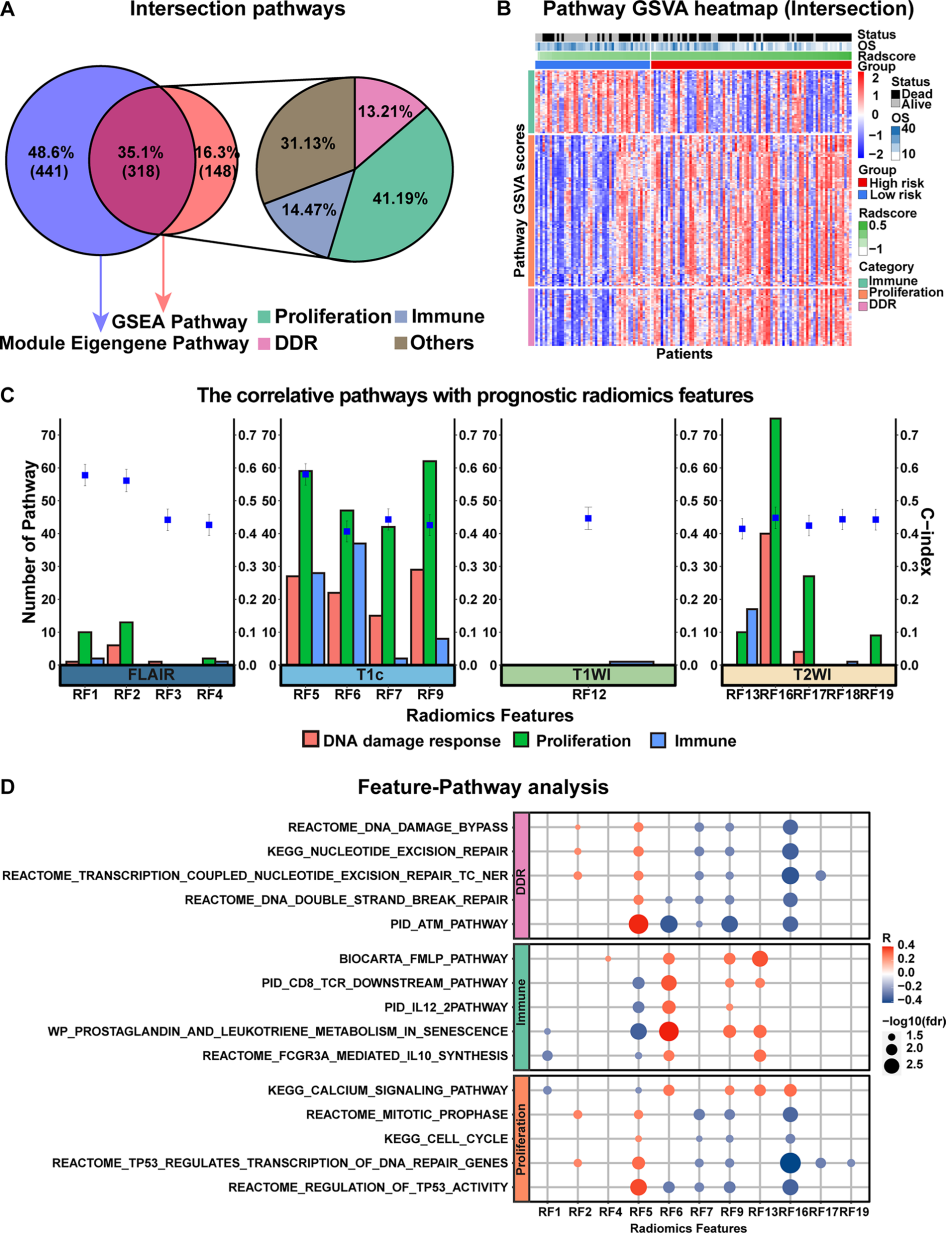
Radiogenomics linking between 20 radiomic features constituting the Radscore and their significantly associated pathways. **A** Venn diagram and Pie chart of the intersective pathways. **B** A heatmap showing the activation of 318 intersective pathways clustered according to their corresponding biological pathway categories (proliferation, immunity, DNA damage response) in a high- and low-risk group of 132 patients. **C** Bar charts revealed the number of biological pathways behind individual features. Fourteen features derived from different MRI sequences have 219 different pathways (proliferation of 131, immune of 46, DDR of 42) associated behind them. **D** A bubble plots of correlation between prognostic radiomic features and classic biological pathways. Correlation results for the top 5 correlated pathways (FDR < 0.01) and prognostic features in each pathway category

A total of 318 intersectional pathways were derived from GSEA and WGCNA approaches, as illustrated in Fig. 5A. 318 intersectional pathways from 6 common databases: 27 from BIOCARTA, 32 from KEGG, 17 from PID, 182 from REACTOME and 51 from WP (P < 0.01), they are summarized in Additional file 1: Table S8. We annotated the biological functions of each of these pathways at different database levels by reviewing the relevant literature and accessing https://www.gsea-msigdb.org, and subsequently these pathways were classified into several different categories according to their different biological functions, including tumor proliferation, immune regulation, DNA damage response (DDR), and others (including viral infections, ion channel transport, transmitter transport, and complex cellular functions). We focused on revealing the potential biological underpinnings of the radiomic phenotype in the former three distinct pathway categories. The pathways and their categories are summarized in Additional file 1: Table S9 and presented as a heat map in Fig. 5B.

#### Identification of biological underpinning of the radiomic phenotypes

**Fig. 6:**
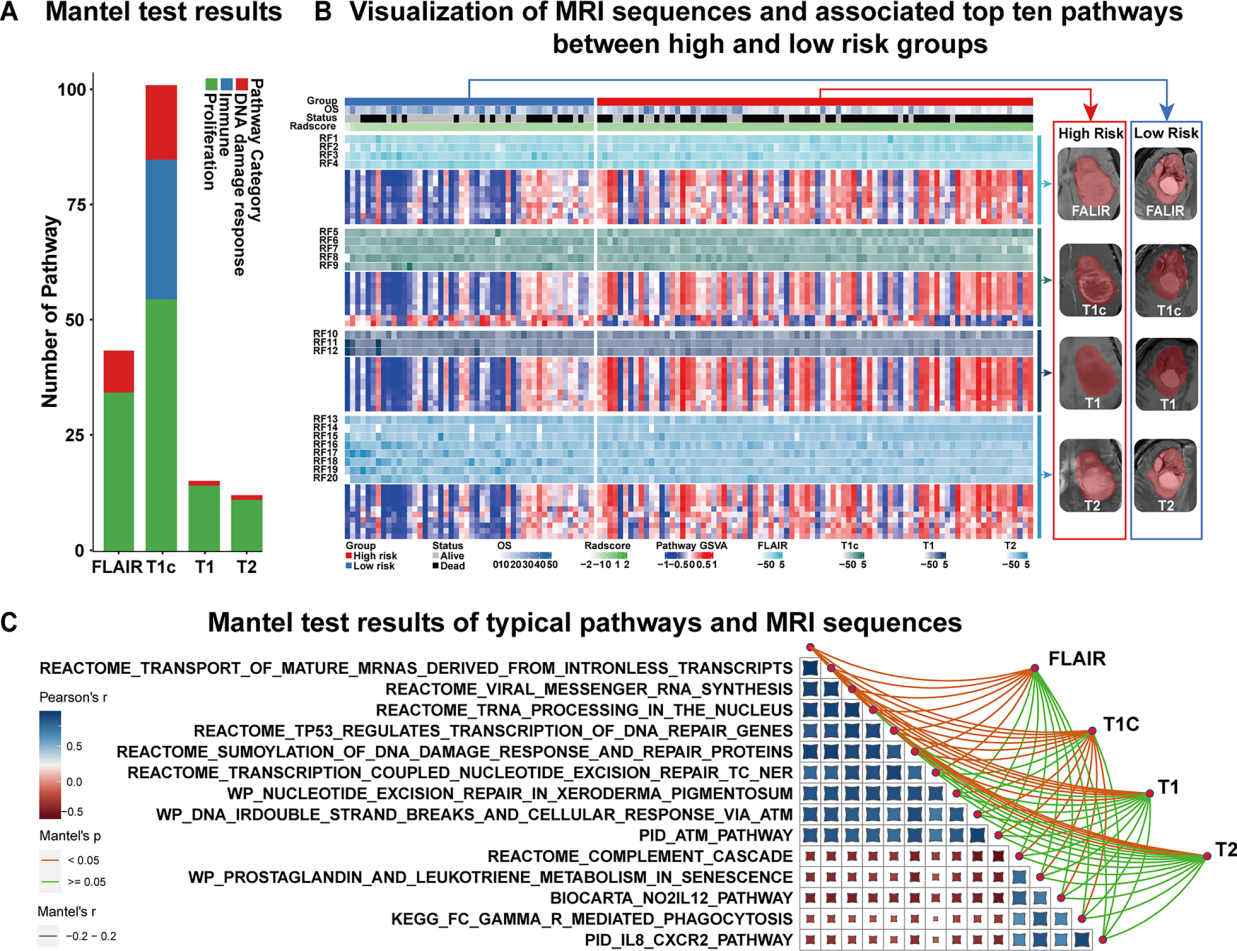
Radiogenomics linking four MRI sequences consist of 20 prognostic radiomic features and their significantly associated pathways. **A** The mantel test results of the number and category of biological pathways behind each sequence. **B** Heatmap of four sequences along with their top 10 significantly associated pathways represented by their pathway gene set variation analysis (GSVA) score across the radiogenomics analysis set with risk groups, Radscore, overall survival, and survival status. The 10 rows immediately after each sequences represented by these prognostic features indicate the activation level (in gene set variation analysis score) of the top 10 significant pathways. **C** Mantel test results between four MRI sequences and typical pathways derived from three biological pathway categories

First, After performing a Pearson correlation with the prognosis-related features, 14 features (FLAIR of 4, T1c of 4, T1 of 1, T2 of 5) and 219 pathways (proliferation of 131, immune of 46, DDR of 42) were obtained, where the Pearson’s FDR < 0.05 was considered significant. The detailed results are shown in Fig. 5C. Representative pathways for each pathway category are shown in Fig. 5D. Second, a Mantel test was performed to further explore the radiogenomics link between pathway categories (131 proliferation-associated, 46 immune-associated, and 42 DNA damage-response-associated) and MRI sequences (FLAIR 4 features, T1c 5 features, T1 3 features and T2 8 features). 170 pathways derived from intersectional pathways were identified, which were considered to have a significant correlation with the MRI sequences (P < 0.05), and the number and category of the associated pathways behind each sequence were summarized in the Fig. 6A. A heat map of each sequence with their associated top 10 pathways in the radiogenomics analysis set is shown in Fig. 6B. The top 5 typical pathways associated with each pathway category were selected are showing in Fig. 6C.

## Discussion

Our research focused on the following three endeavors. First, we constructed and independently validated a Radscore calculated from preoperative conventional MRI sequences for predicting OS in adult IDH wild-type GBM. Second, in contrast to previous studies in radiogenomics that used pathways enriched by a single approach, our study acquired the intersectional pathways enriched by both GSEA and WGCNA methods to reveal the biological underpinnings behind radiomic features. Third, the biological pathways underlying the radiomic phenotypes were systematically investigated.

As the field of radiomics flourishes, biological validation will become an indispensable assessment criterion in clinical decision-making, and has recently been applied to radiomics studies [28]. For GBM, two radiogenomic studies have explored the biological meaning behind radiomic phenotypes based on conventional MRI sequences [13, 14]. However, both studies investigated patients with pathologically diagnosed GBM which includes IDH mutant astrocytomas according to the 2021 WHO classifications of the central nervous system tumors (CNS5) [5]. Our study focused on IDH wild-type GBM, which is more in accordance with the definition of GBM according to the WHO CNS5 [5].

Several previous studies have confirmed the association of MRI features with prognosis and molecular subgroups of gliomas [29–31]. In this study, Radscore was shown to be significantly associated with patient survival prognosis (OS and status) with a higher predictive power than existing clinical indicators. Patients stratified by the same radscore in both the training and internal validation groups as well as the external validation group showed a different prognosis (log-rank P < 0.001), with the radscore < − 0.1026 subgroup surviving significantly longer than patients in the radscore ≥ − 0.1026 subgroup. Similarly, in radiomic-clinical modal (R-CM) nomograms, patients with smaller radscore scores had longer overall survival. Furthermore, radcore significantly outperformed existing clinical predictors (gender, age, preoperative Karnofsky performance status (KPS) score, extent of resection, radiotherapy and chemotherapy) in the OS predictions at 6, 12, 18 and 24 months.

We elaborated on the biological meaning of individual prognostic features in terms of the categories and number of driving pathways. For the categories of driving pathways, our radiogenomics analysis revealed that five prognostic radiomic features (i.e., RF1, RF5-RF7, RF9) are associated with three major pathway categories (DDR, proliferation, and immune pathways), while the RF12 are only associated with one pathway related to the immune response. These findings demonstrate that multiple biological processes may be involved in different radiomic features. It is worth noting that RF5-RF7 and RF9 all belong to the T1c sequence, whereas RF12 is the only radiomic feature derived from the T1 sequence, which indicates that the radiomic features from the T1c sequence are associated with more biological information than those from other sequences. For the number of driving pathways, we found that each of the top features (RF5, RF6, and RF16) had more than 100 associated pathways. Further radiogenomic analysis revealed that these three features were all derived from imaging textures. Consistent with previous radiomics findings [32, 33], our results also demonstrated the prominence of textural features in the prognostic imaging features. In addition, the most enriched features of DDR and proliferation pathways were RF6 derived from T1c, and RF16 derived from T2 was the most enriched feature for the immune pathways.

When it comes to MRI sequence, radiomic features extracting form T1c and FLAIR were more related to biological pathways of GBM compared to T1 and T2. For T1c, the result shows that all three pathway categories (DDR, proliferation, and immune pathways) and 100 intersectional pathways (58.8%) are associated with radiomic features derived from T1c. This partly explains why imaging features derived from T1c showed substantially incremental value in GBM prognostication [34, 35]. For FLAIR, there were two pathway categories (DDR and proliferation) and 43 intersectional pathways (25.3%) associated with radiomic features derived from FLAIR. These radiogenomic result biologically corroborates the potent performance of FLAIR in the progression of GBM [36, 37].

Combining the genetic characteristics of IDH wildtype GBM and our radiogenomic findings, we propose potential explanations for the biological mechanisms underlying the radiomic model. First, the high-risk group identified by the radiomic model is associated with proliferation and DDR pathway categories that promote GBM progression. More specifically, the positive activity of proliferation and DDR pathways in high-riks group, including MAPK signaling pathway, P53 pathway, STAT3 pathway, and DNA damage response pathways are reported to result in the induction of GBM growth [38–40]. Second, the high-risk group is also assoicated with immune pathways that promote GBM progression under immunosuppressive conditions. Previous studies have confirmed that GBM cells can inhibit the maturation and functioning of immune cells by secreting a variety of cytokines that upregulating immune checkpoint pathways such as programmed cell death protein-1 (PD-1) pathway, which leads to the progression of GBM [41, 42]. Recently, targeted therapies, such as DDR inhibitors and anti-PD-1 immunotherapy, are reported to be promising in elongation of GBM patients’ clinical outcomes [43, 44]. Hence, our radiogenomic results may shed light on noninasive identification of key pathways of IDH wild-type GBM at different risk stratification, and further informing individualized treatment strategies.

This study has several limitations. First, the current study is retrospective and needs to be substantiated by prospective multicenter studies. Second, incorporation of advanced MRI sequences, such as perfusion-weighted imaging (PWI), diffusion tensor imaging (DTI), and magnetic resonance spectroscopy (MRS), may provide additional imaging information and enhance prognostication performance of the radiomic model. Third, IDH wild-type astrocytomas with TERT promoter mutations, EGFR amplification, and + 7/− 10 chromosome copy number changes need to be further considered in future study to fully elucidate the intratumoral heterogeneity of IDH wild-type GBM according to the WHO CNS5.

In conclusion, this study proposed a radiomic model using preoperative conventional MRI sequences to predict the clinical outcomes of patients with IDH wild-type GBM. Remarkably, radiogenomic results demonstrated that prognostic radiomic phenotypes derived from conventional MRI are associated with distinct pathways involved in proliferation, DDR, and immunity in IDH wild-type GBM.

### Supplementary Information

**Additional file 1.** A1: MRI sequence parameters. A2: Description of radiomic features used in our study. A3: RNA samples preparation and sequencing. A4: Detection of IDH mutation. A5: WGCNA process and module acquisition details. **Table S1.** A summary of the radiomic features extracted. **Table S2.** A summary of the parameters according to Image Biomarker Standardisation Initiative (IBSI). **Table S3.** Characteristics of patients in the training set, internal validation set and external validation set. **Table S4.** A summary of the C-index and AIC values for OS prediction of three models. **Table S5.** A summary of the Radscore-related pathways enriched by GSEA. **Table S6.** A summary of the genes in the five Radscore-related modules. **Table S7.** A summary of the pathways enriched by Radscore-related modules. **Table S8.** A summary of the intersectional pathways enriched by GSEA and WGCNA. **Table S9.** A summary of the pathway categories on proliferation, DDR and Immune. **Figure S1.** The criteria for patients’ inclusion and exclusion. **Figure S2.** Forest plot of prognostic radiomic features. **Figure S3.** Radiomic feature selection 1. **Figure S4.** Radiomic feature selection 2. **Figure S5.** Incremental value of radiomic model. **Figure S6.** Radscore for each patient. **Figure S7.** Cluster dendrogram and corresponding trait heat map for each patient in WGCNA. **Figure S8.** The Soft threshold selection process. **Figure S9.** Kaplan–Meier plot of fivefold cross-validation.

## Data Availability

Data and materials used to support the findings of this study are available from the corresponding author upon request.
