# Supplementary material for: Biological underpinnings of radiomic magnetic resonance imaging phenotypes for risk stratification in IDH wild-type glioblastoma
Source: J Transl Med. 2023 Nov 22;21:841. doi: 10.1186/s12967-023-04551-3 (PMC10664532; doi:10.1186/s12967-023-04551-3)
Supplement: Supplementary file 1 — Additional file 1. A1: MRI sequence parameters. A2: Description of radiomic features used in our study. A3: RNA samples preparation and sequencing. A4: Detection of IDH mutation. A5: WGCNA process and module acquisition details. Table S1. A summary of the radiomic features extracted. Table S2. A summary of the parameters according to Image Biomarker Standardisation Initiative (IBSI). Table S3. Characteristics of patients in the training set, internal validation set and external validation set. Table S4. A summary of the C-index and AIC values for OS prediction of three models. Table S5. A summary of the Radscore-related pathways enriched by GSEA. Table S6. A summary of the genes in the five Radscore-related modules. Table S7. A summary of the pathways enriched by Radscore-related modules. Table S8. A summary of the intersectional pathways enriched by GSEA and WGCNA. Table S9. A summary of the pathway categories on proliferation, DDR and Immune. Figure S1. The criteria for patients’ inclusion and exclusion. Figure S2. Forest plot of prognostic radiomic features. Figure S3. Radiomic feature selection 1. Figure S4. Radiomic feature selection 2. Figure S5. Incremental value of radiomic model. Figure S6. Radscore for each patient. Figure S7. Cluster dendrogram and corresponding trait heat map for each patient in WGCNA. Figure S8. The Soft threshold selection process. Figure S9. Kaplan–Meier plot of fivefold cross-validation. [file 12967_2023_4551_MOESM1_ESM.docx]

**Additional file 1**

**This Additional file includes:**

A1: MRI Sequence parameters.

A2: Description of radiomic features used in our study.

A3: RNA samples preparation and sequencing.

A4: Detection of IDH mutation.

A5: WGCNA Process and Module Acquisition Details.

Table S1: A summary of the radiomic features extracted.

Table S2: A summary of the parameters according to Image Biomarker Standardisation Initiative (IBSI).

Table S3: Characteristics of patients in the training set, internal validation set and external validation set.

Table S4: A summary of the C-index and AIC values for OS prediction of three models.

Table S5: A summary of the Radscore-related pathways enriched by GSEA.

Table S6: A summary of the genes in the five Radscore-related modules.

Table S7: A summary of the pathways enriched by Radscore-related modules.

Table S8: A summary of the intersectional pathways enriched by GSEA and WGCNA.

Table S9: A summary of the pathway categories on proliferation, DDR and Immune.

Figure S1: The criteria for patients’ inclusion and exclusion.

Figure S2: Forest plot of prognostic radiomic features.

Figure S3: Radiomic feature selection 1.

Figure S4: Radiomic feature selection 2.

Figure S5: Incremental value of radiomic model.

Figure S6: Radscore for each patient.

Figure S7: Cluster dendrogram and corresponding trait heat map for each patient in WGCNA.

Figure S8: The Soft threshold selection process.

Figure S9: Kaplan-Meier plot of 5-fold cross-validation.

**A1: MRI Sequence parameters**

All MR images were acquired during routine clinical work-up on either a 3.0 T MR scanner (Magnetom Skyra, Siemens Healthcare, Erlangen, Germany) with an integrated 20-channel head and neck coil, or a 3.0 T MR scanner (Magnetom Trio TIM, Siemens Healthcare, Erlangen, Germany) with a 12-channel head coil, or a 3.0 T MR scanner (Discovery MR 750, GE Healthcare, Milwaukee, WI, USA) with an 8-channel head coil, or a 3.0 T MR scanner (Ingenia, Philips Healthcare, Best, Netherlands) with a 15-channel head coil. The brain imaging protocol at our institution includes the following sequences: (a) precontrast axial and sagittal T1-weighted imaging (T1); (b) axial T2-weighted imaging (T2); (c) axial T2-weighted fluid-attenuated inversion recovery (FLAIR) imaging; (d) contrast-enhanced axial, sagittal, and coronal T1-weighted imaging (T1c). The contrast-enhanced sequences were acquired immediately after intravenous administration of a 0.1 mmol/kg dose of gadolinium-based contrast agent (Gadolinium-diethylenetriamine pentaacetic acid [Gd-DTPA], Bayer Healthcare, Leverkusen, Germany, or Gadoteric Acid Meglumine Salt Injection, Hengrui Healthcare, Jiangsu, China). Parameters for all the sequences acquisition were as follow:

T1 and T1c: repetition time (TR), 220-1750 ms; echo time (TE), 2.3-24 ms; echo train length (ETL), 1-12; section thickness, 5 mm; image slice spacing, 1.5 mm; number of averages/excitations, 1; flip angle (FA), 70°-111°; field of view (FOV), 220×192-240×240 mm^2^; matrix, 256×162-320×256 mm^2^.

T2: TR, 1873-5390 ms; TE, 70-117 ms; ETL, 16-32; section thickness, 5 mm; image slice spacing, 1.5 mm; number of averages/excitations, 1; FA, 90°-142°; FOV, 220×192-240×240 mm^2^; matrix, 320×238-512×512 mm^2^.

FLAIR: TR, 4500-8400 ms; TE, 85-150 ms; inversion time (TI), 1670-2250 ms; ETL, 1-38; section thickness, 5 mm; image slice spacing, 1.5 mm; number of averages/excitations, 1; FA, 90°-150°; FOV, 220×192-240×240 mm^2^; matrix, 256×179-256×256 mm^2^.

**A2: Description of radiomic features used in our study**

**(1) Description of radiomic features.** High-dimensional radiomics features extraction from the delineated volume of interest (VOI) of tumor area is the heart of radiomics analysis. Different from the traditional radiological features that are more familiar with radiologists, radiomics features are mathematically extracted quantitative descriptors which are generally not part of the radiologists’ lexicon. These features are identified and calculated by automatic algorithms that capture patterns in the imaging, such as voxel intensity variations, lesion shape characteristics and image textures. These radiomics features are well designed to capture microscale information hidden within conventional imaging beyond what is visible to the naked human eye.

**(2) Description of wavelet images.** To fully characterize the image patterns, radiomics features are usually extracted from not only the original medical images but also the transformed, or derived images by using filters onto the original images. In our study we extracted radiomics features from three types of images: original images, wavelet images, and LoG (Laplacian of Gaussian) images.

Wavelet images were obtained by applying wavelet transform on the original images. Wavelet transform can decouple informative textures by decomposing the original images into multiple low- and high-frequency components. In this study, a three-dimensional wavelet transform was applied to each sequence of MR images, where the original MR image ***I*** was decomposed into eight component images. Let *H* and *L* be a high-pass and low-pass wavelet function, respectively. Here coiflet1 wavelet was used. Then, the eight decomposed images can be denoted as ***I****_HHH_*, ***I****_HHL_*, ***I****_HLH_*, ***I****_HLL_*, ***I****_LHH_*, ***I****_LHL_*, ***I****_LLH_*, ***I****_LLL_*, where the three subscripts meant the high- or low-pass filtering operations along *x*, *y* and *z* directions of the original 3D MR image. For example, ***I****_HHL_* was obtained by applying a high-pass filter *H* in *x* direction, a high-pass filter *H* in y direction, and a low-pass filter *L* in *z* direction as

$$\boldsymbol{I}_{HHL}\left( x,y,z \right) = \sum_{l = 1}^{F_{H}} \sum_{m = 1}^{F_{H}} \sum_{n = 1}^{F_{L}} H\left( l \right)H\left( m \right)L\left( n \right)\boldsymbol{I}_{HHL}\left( x+l,y+m,z+n \right), (1)$$

where *F_H_* and *F_L_* denoted the length of the high-pass filter and the low-pass filter, respectively. The size of each decomposed image equaled the original image, so the delineated VOI can be directly applied on the decomposed images for feature extraction.

**(3) Description of LoG images.** LoG images were obtained by applying LoG filtering operation on the original images. LoG performs two filtering operations, a Gaussian filtering and a Laplacian filtering. The first Gaussian filter smooths the original image to make the subsequent Laplacian filter less sensitive to image noise. The Laplacian filter is a convolutional filter used to detect edges. Laplacian filter is a second derivative measurement and thus sensitive to noise. Without the first Gaussian filter, small edges derived from noise may detract from larger meaningful edges. The Gaussian filter centered on zero is defined as

$$G\left( x,y,z,\sigma\right) = \frac{1}{\left( \sigma\sqrt{2\pi} \right)^{3}}e^{-\frac{x^{2}+y^{2}+z^{2}}{2\sigma^{2}}}, (2)$$

where $\sigma$ is the standard deviation. After convolved by the second derivative Laplacian kernel $\nabla^{2}G\left( x,y,z \right)$, which is sensitive to areas with rapidly changing intensities, enhancing edges, the LoG filter has the form as

$$LoG\left( x,y,z,\sigma\right) = -\frac{1}{\pi\sigma^{4}}\left[ 1-\frac{x^{2}+y^{2}+z^{2}}{2\sigma^{2}} \right]e^{-\frac{x^{2}+y^{2}+z^{2}}{2\sigma^{2}}}. (3)$$

The parameter $\sigma$, determining the width of the Gaussian kernel, can be used to emphasize finer (low $\sigma$ values) or coarser (high $\sigma$ values) textures. The value of $\sigma$ must be provided by the user. In our study, four values, 2.0, 3.0, 4.0 and 5.0 were used to emphasize edges with different scales.

**(4) Description of the extracted radiomics features.** In the image processing and computer vision communities, many well-defined imaging features are used to quantitatively describe the shape or the first-/high-order patterns. These features have well-defined imaging meanings. In this study, from the VOIs of the original images, the wavelet images and the LoG images, three groups of features were extracted, including first-order intensity statistics, shape descriptors, and texture features, as shown in the **Table S1** in this Additional file. The detailed calculation of these features can be found in the literatures reported by Aerts HJ et al^1^ and Lambin P et al^2^. In total, 4746 features were finally extracted from foure MR sequences.

**(5) Description of selected features used for LASSO model building.** After feature selection, 20 features were finally selected for LASSO Cox model building, including ten order intensity features (RF1, RF2, RF7, RF9, RF11, RF12, RF14, RF15, RF17, RF19) and ten higher order texture features (RF3-RF6, RF8, RF10, RF13, RF16, RF18, RF20), as shown in **Figure S2**.

In our study, five basic matrices were used to define imaging textures: gray-level co-occurrence matrix (GLCM), gray-level run length matrix (GLRLM), gray level size zone matrix (GLSZM), gray level dependence matrix (GLDM), and neighborhood gray-tone difference matrix (NGTDM). These textures are similar with visual features that human being uses in interpreting pictorial information. GLCM, proposed by Haralick RM et al^3^, estimates image textural properties between two voxels. Each entry (*i*, *j*) in GLCM matrix corresponds to the number of occurrences of voxel pair *i* and *j*. GLRLM is another approach proposed by Galloway MM^4^ for high-order texture analysis. GLRLM reflects the distribution of gray level runs, i.e. a set of consecutive collinear voxels having the same gray level. GLSZM makes use of the gray-level size zone, which is a flat area with the same gray-level^5^. GLDM quantifies gray level dependencies, i.e. the number of connected voxels within certain distance that are dependent on the center voxel^6^. NGTDM reflects a gray-level difference between voxels with certain gray-level and their neighboring voxels^7^. These texture features have been widely used in object recognition and image interpretation tasks. Next, we will introduce the specific meaning of the 20 selected features.

RF1: Kurtosis extracted from the LoG image derived from FLAIR with $\sigma$ = 2, Kurtosis is a measure of the ‘peakedness’ of the distribution of values in the image ROI. A higher kurtosis implies that the mass of the distribution is concentrated towards the tail(s) rather than towards the mean. A lower kurtosis implies the reverse: that the mass of the distribution is concentrated towards a spike near the Mean value.

RF2: Mean extracted from the LoG image derived from FLAIR with $\sigma$ = 3.The average gray level intensity within the ROI.

RF3: GLRLM. Long Run Low Gray-Level Emphasis extracted from the LLH wavelet transformed image derived from FLAIR. LRLGLRE measures the joint distribution of long run lengths with lower gray-level values.

RF4: GLRLM. Long Run Low Gray-Level Emphasis extracted from the LLL wavelet transformed image derived from FLAIR. LRLGLRE measures the joint distribution of long run lengths with lower gray-level values.

RF5: GLDM. Large Dependence Low Gray-Level Emphasis extracted from the LoG image derived from T1c with $\sigma$ = 3. A measure of the joint distribution of large dependence with lower gray-level values.

RF6: GLDM. Large Dependence Low Gray-Level Emphasis extracted from the LoG image derived from T1c with $\sigma$= 4. A measure of the joint distribution of large dependence with lower gray-level values.

RF7: 10th percentile extracted from the LHL wavelet transformed image derived from T1c. The 10^th^ percentile of X.

RF8: GLCM. Informational Measure of Correlation (IMC) 1 extracted from the LHL wavelet transformed image derived from T1c. IMC1 assesses the correlation between the probability distributions of ii and jj (quantifying the complexity of the texture), using mutual information.

RF9: Kurtosis extracted from the HHL wavelet transformed image derived from T1c. Kurtosis is a measure of the ‘peakedness’ of the distribution of values in the image ROI. A higher kurtosis implies that the mass of the distribution is concentrated towards the tail(s) rather than towards the mean. A lower kurtosis implies the reverse: that the mass of the distribution is concentrated towards a spike near the Mean value.

RF10: GLDM. Dependence Non-Uniformity Normalized (DNN) extracted from the LoG image derived from T1 with $\sigma$ = 2. Measures the similarity of dependence throughout the image, with a lower value indicating more homogeneity among dependencies in the image. This is the normalized version of the DLN formula.

RF11: Energy extracted from the LoG image derived from T1 with $\sigma$ = 3. Energy is a measure of the magnitude of voxel values in an image. A larger values implies a greater sum of the squares of these values.

RF12: Range extracted from the LoG image derived from T1 with $\sigma$ = 3. The range of gray values in the ROI.

RF13: GLDM. Dependence Non-Uniformity Normalized (DNN) extracted from the LoG image derived from T2 with $\sigma$ = 2. Measures the similarity of dependence throughout the image, with a lower value indicating more homogeneity among dependencies in the image. This is the normalized version of the DLN formula.

RF14: 10th percentile extracted from the LLH wavelet transformed image derived from T1c. The 10^th^ percentile of X.

RF15: Mean extracted from the LLH wavelet transformed image derived from T2. The average gray level intensity within the ROI.

RF16: GLCM. Cluster Prominence extracted from the LLH wavelet transformed image derived from T2. Cluster Prominence is a measure of the skewness and asymmetry of the GLCM. A higher values implies more asymmetry about the mean while a lower value indicates a peak near the mean value and less variation about the mean.

RF17: Variance extracted from the LHL wavelet transformed image derived from T2. Variance is the the mean of the squared distances of each intensity value from the Mean value. This is a measure of the spread of the distribution about the mean. By definition, variance =$\sigma$ ^2^.

RF18: GLCM. Informational Measure of Correlation (IMC) 2 extracted from the LHL wavelet transformed image derived from T2. IMC2 also assesses the correlation between the probability distributions of ii and jj (quantifying the complexity of the texture).

RF19: 90th percentile extracted from the HLL wavelet transformed image derived from T2. The 90^th^ percentile of X.

RF20: GLCM. Cluster Tendency extracted from the LHL wavelet transformed image derived from T2. Cluster Tendency is a measure of groupings of voxels with similar gray-level values.

**A3: RNA samples preparation and sequencing**

**(1) RNA quantification and qualification:** RNA degradation and contamination were monitored on 1% agarose gels.  RNA purity was checked using the NanoPhotometer® spectrophotometer (IMPLEN, CA, USA). RNA concentration was measured using Qubit® RNA Assay Kit in Qubit® 2.0 Flurometer (Life Technologies, CA, USA). RNA integrity was assessed using the RNA Nano 6000 Assay Kit of the Bioanalyzer 2100 system (Agilent Technologies, CA, USA).

**(2) Library preparation for Transcriptome sequencing:** A total amount of 3 µg RNA per sample was used as input material for the RNA sample preparations. Sequencing libraries were generated using NEBNext® UltraTM RNA Library Prep Kit for Illumina® (NEB, USA) following manufacturer’s recommendations and index codes were added to attribute sequences to each sample. Briefly, mRNA was purified from total RNA using poly-T oligo-attached magnetic beads. Fragmentation was carried out using divalent cations under elevated temperature in NEBNext First Strand Synthesis Reaction Buffer (5X). First strand cDNA was synthesized using random hexamer primer and M-MuLV Reverse Transcriptase (Rnase H^-^). Second strand cDNA synthesis was subsequently performed using DNA Polymerase I and RNase H. Remaining overhangs were converted into blunt ends via exonuclease/polymerase activities. After adenylation of 3’ ends of DNA fragments, NEBNext Adaptor with hairpin loop structure were ligated to prepare for hybridization. In order to select cDNA fragments of preferentially 150 ~ 200 bp in length, the library fragments were purified with AMPure XP system (Beckman Coulter, Beverly, USA). Then 3 µl USER Enzyme (NEB, USA) was used with size-selected, adaptor-ligated cDNA at 37°C for 15 min followed by 5 min at 95 °C before PCR. Then PCR was performed with Phusion High-Fidelity DNA polymerase, Universal PCR primers and Index (X) Primer. At last, PCR products were purified (AMPure XP system) and library quality was assessed on the Agilent Bioanalyzer 2100 system.

**(3) Clustering and sequencing:** The clustering of the index-coded samples was performed on a cBot Cluster Generation System using TruSeq PE Cluster Kit v3-cBot-HS (Illumia). After cluster generation, the library preparations were sequenced on an Illumina Hiseq platform and 125 bp/150 bp paired-end reads were generated.

**(4) Quality control:** Raw data (raw reads) of fastq format were firstly processed through in-house perl scripts. In this step, clean data (clean reads) were obtained by removing reads containing adapter, reads containing ploy-N and low-quality reads from raw data. At the same time, Q20, Q30 and GC content the clean data were calculated. All the downstream analyses were based on the clean data with high quality.

**(5) Reads mapping to the reference genome:** Reference genome and gene model annotation files were downloaded from genome website directly. Index of the reference genome was built using STAR and paired-end clean reads were aligned to the reference genome using STAR (v2.5.1b). STAR used the method of Maximal Mappable Prefix (MMP) which can generate a precise mapping result for junction reads.

**(6) Quantification of gene expression level:** HTSeq v0.6.0 was used to count the reads numbers mapped to each gene. And then FPKM (expected number of Fragments Per Kilobase of transcript sequence per Millions base pairs sequenced) of each gene was calculated based on the length of the gene and reads count mapped to this gene.

**A4: Detection of IDH mutation**

Mutational hotspots of IDH1/IDH2 were evaluated by direct sequencing. Tissues from representative tumor area (the proportion of tumor cells＞20%) were scrapped off from dewaxed sections and treated with PCR reaction solution A 10μl (reaction mixture containing 1μl of cell lysate, 0.3mM of each dNTP, 2.5mM MgCl2, 0.3μM of each primer and 0.2U of KAPA HiFi HotStart DNA Polymerase (Kapa Biosystems Inc., Wilmington, USA)), Shrimp Alkaline Phosphatase (SAP) enzyme (NEB, Ipswich, MA, USA) 2μl and BigDye (BigDye™ Terminator v3.1 Cycle Sequencing Kit, Thermo Fisher Scientific, Waltham, MA, USA) 1μl for centrifugation at 2000 rpm for 10 sec. The crude cell lysate was centrifuged and supernatant was used for subsequent PCR analysis. The forward primer primers (IDH1-F:5’-CGGTCTTCAGAGAAGCCATT-3’,IDH1-R:5’-CACATTATTGCCAACATGAC-3’,IDH2-F:5’-AGCCCATCATCTGCAAAAAC-3’,IDH2-R:5’-CTAGGCGAGGAGCTCCAGT-3’) were used to amplify the region of mutational hotspots of IDH1/IDH2. ①PCR was performed was initiated at 95°C for 5 min, followed by 40 cycles of 95°C for 20 sec, 57°C for 30 sec and 72°C for 1min, and a final extension of 72°C for 5 min and 10°C for 10 min. ②5μl PCR products were then mixed with 2μl SAP enzyme and reacted at 37°C for 40min and then at 80° C for 15min. ③Then 18μl PCR reaction solution C（CWBIO, Beijing, Chima）, 1μl products from ② step, and 1μl BigDye were mixed and reacted at 96°C for 1 min, followed by 30 cycles of 96°C for 10 sec, 50° C for 5 sec and 60° C for 2 min, and a final extension of 25°C for 1 min and 10°C for 10 min. Then 50μl natrium asceticism-ethanol mixture (3M NaAc: ethanol=1:15) were added and the mixture was centrifuged for 30min (12000 rpm, 4°C), with the supernatant being discarded. Then 70μl 75% ethanol were added and the mixture was centrifugated for 15min (12000 rpm, 4°C), with the supernatant being discarded. After complete volatilization of the ethanol at room temperature, 12μl Hi-Di™ Formamide (Thermo Fisher Scientific, Waltham, MA, USA) were added into the precipitate to dissolve the DNA. The dissolved products were sequenced on Applied Biosystems™ 3500DxGenetic Analyzer (Thermo Fisher Scientific, Waltham, MA, USA), and analyzed by Chromas software (Technelysium, South Brisbane, Australia). The sequencing results were compared with wild-type sequences of IDH1/IDH2 for analysis.

**A5: WGCNA Process and Module Acquisition Details**

We performed WGCNA analysis of 132 cases of FPKM data from the radiogenomics analysis set with the following details: First, screening for genes in the top 75% of median absolute deviation with MAD greater than at least 0.01, and using "average" clustering method to detect outliers and cluster samples. Then, we obtained 9 modules and their corresponding gene sets by setting the following parameters: soft threshold = 6, maxBlockSize = 21000, corType = "pearson", TOMType = "unsigned", minModuleSize = 300, deepSplit=3 , mergeCutHeight = 0.15. Finally, in order to obtain MRI-related modules, we calculated the module GSVA values for each patient and subsequently did a Pearson correlation with its corresponding Radscore. 5 modules (turquoise module, 3,722 genes; blue module, 3,186 genes; brown module, 2,820 genes; green module, 2,031 genes; red module, 884 genes) with FDR less than 0.01 were selected as significantly correlated. The included genes of the 5 correlated modules are shown in **Table 6**.

**Reference**

1. Aerts HJ, Velazquez ER, Leijenaar RT, et al. Decoding tumour phenotype by noninvasive imaging using a quantitative radiomics approach. Nat Commun. 2014;5:4006.
2. Lambin P, Leijenaar R, Deist TM, et al. Radiomics: the bridge between medical imaging and personalized medicine. Nat Rev Clin Oncol. 2017:14(12):749.
3. Haralick RM, Shanmugam K, Dinstein IH. Textural features for image classification. IEEE T Syst Man Cyb. 1973;6:610-621.
4. Galloway MM. Texture analysis using gray level run lengths. Computer Graphics and Image Processing. 1975;4(2):172-179.
5. Guillaume T, Bernard F, Claire N, Sandrine P, Pierre C, Nicolas L, Jean S, Jean-Luc M. Texture indexes and gray level size zone matrix: application to cell nuclei classification. Pattern Recognition and Information Processing (PRIP). 2009;140-145.
6. Sun C, Wee WG. Neighboring Gray Level Dependence Matrix for Texture Classification. Lect Note S Comput SC. 1983;23(3):341-352.
7. Amadasun M, King R. Textural features corresponding to textural properties. IEEE T Syst Man Cyb. 1989;19:1264-1274.

**Table S1.** A summary of the radiomic features extracted. Fourteen shape features describing the 3D geometric characteristics of the VOI were extracted. From the original images and the transformed images (applying wavelet transform or LoG filtering), 234 intensity features describing the first-order distribution of the intensities were extracted, while 949 texture features were computed to describe the patterns, or the high-order intensity distributions with five methods, including gray-level co-occurrence matrix (GLCM), gray-level run length matrix (GLRLM), gray level size zone matrix (GLSZM), gray level dependence matrix (GLDM), and neighborhood gray-tone difference matrix (NGTDM). Informational Measure of Correlation have two calculation methods.^1^ Totally, 4746 features were extracted from foure MR sequences.

| **Feature Classes** | | **Feature Names** |
| --- | --- | --- |
| Shape Features | | Elongation, Flatness, Least Axis Length, Major Axis Length, Maximum 2D Diameter Column, Maximum 2D Diameter Row, Maximum 2D Diameter Slice, Maximum 3D Diameter, Mesh Volume, Minor Axis Length, Sphericity, Surface Area, Surface Volume Ratio, Voxel Volume |
| Intensity Features | | Maximum, Median, Minimum, Mean, Energy, Entropy, Variance, Kurtosis, Root Mean Square, Skewness, 10th Percentile, 90th Percentile, Mean Absolute Deviation, Uniformity, Range, Robust Mean Absolute Deviation, Total Energy, Interquartile Range |
| Texture Features | GLCM  Features | Contrast, Correlation, Autocorrelation, Cluster Tendency, Sum Average, Sum Entropy, Sum Squares, Difference Average, Difference Variance, Difference Entropy, Cluster Prominence, Cluster Shade, Maximum Probability, Inverse Difference Moment, Informational Measure of Correlation 1/2, Inverse Difference Moment Normalized, Inverse Difference Normalized, Inverse Difference, Inverse Variance, Maximal Correlation Coefficient, Joint Average, Joint Energy, Joint Entropy |
|  | GLDM  Features | Dependence Entropy, Dependence Non-Uniformity, Dependence Non-Uniformity Normalized, Dependence Variance, Gray-Level Non-Uniformity, Gray-Level Variance, High Gray-Level Emphasis, Large Dependence Emphasis, Large Dependence High Gray-Level Emphasis, Large Dependence Low Gray-Level Emphasis, Low Gray-Level Emphasis, Small Dependence Emphasis, Small Dependence High Gray-Level Emphasis, Small Dependence Low Gray-Level Emphasis |
|  | GLRLM  Features | Gray-Level Non-uniformity, Gray-Level Non-uniformity Normalized, Gray-Level Variance, High Gray-Level Run Emphasis, Long Run Emphasis, Long Run High Gray-Level Emphasis, Long Run Low Gray-Level Emphasis, Low Gray-Level Run Emphasis, Run Entropy, Run Length Non-Uniformity, Run Length Non-Uniformity Normalized, Run Percentage, Run Variance, Short Run Emphasis, Short Run High Gray-Level Emphasis, Short Run Low Gray-Level Emphasis |
|  | GLSZM  Features | Gray-Level Non-Uniformity, Gray-Level Non-Uniformity Normalized, Gray-Level Non-Uniformity Normalized, High Gray-Level Zone Emphasis, Large Area Emphasis, Large Area High Gray-Level Emphasis, Large Area Low Gray-Level Emphasis, Low Gray-Level Zone Emphasis, Size Zone Non-Uniformity, Size Zone Non-Uniformity Normalized, Small Area Emphasis, Small Area High Gray-Level Emphasis, Small Area Low Gray-Level Emphasis, Zone Entropy, Zone Percentage, Zone Variance |
|  | NGTDM  Features | Coarseness, Contrast, Busyness, Complexity, Strength |

**Table S2.** A summary of the parameters according to Image Biomarker Standardisation Initiative (IBSI). Details on image processing and biomarker extraction according to the IBSI radiomics reporting guideline (<https://ibsi.readthedocs.io/en/latest/04_Radiomics_reporting_guidelines_and_nomenclature.html>).

| **Topic** | **Description** |
| --- | --- |
| **Patients** | |
| Region of interest | Brain tumor |
| Patient preparation | Administration of sedative hypnotics to the patients who cannot cooperate with the examination prior to image acquisition; The use of ear plugs for patient comfort during scanning |
| Contrast agent | See details in MRI Sequence parameters in this Additional file |
| Comorbidities | None |
| **Acquisition** | |
| Acquisition protocol | See details in MRI Sequence parameters in this Additional file |
| Scanner type | See details in MRI Sequence parameters in this Additional file |
| Imaging modality | See details in MRI Sequence parameters in this Additional file |
| Static/dynamic scans | Static |
| Scanner calibration | Phantom scan, automatic prescan, and coil sensitivity calibration scan are used for calibration |
| Patient instructions | Tell the patient not to move during the acquisition and to be calm and relaxed |
| Anatomical motion correction | The scanned position is head. Normally there is no motion artifacts from heart beating and breathing. We will always tell the patient not to move before scan. And we will check the image right after every scan. If there is a motion, we will discard that scan and rescan |
| Scan duration | From 6 minutes and 46 seconds to 8 minutes and 57 seconds |
| RF coil | See details in MRI Sequence parameters in this Additional file |
| Scanning sequence | See details in MRI Sequence parameters in this Additional file |
| Repetition time | See details in MRI Sequence parameters in this Additional file |
| Echo time | See details in MRI Sequence parameters in this Additional file |
| Echo train length | See details in MRI Sequence parameters in this Additional file |
| Inversion time | See details in MRI Sequence parameters in this Additional file |
| Flip angle | See details in MRI Sequence parameters in this Additional file |
| Acquisition type | 2D |
| k-space traversal | The acquisition trajectory for T1, T1c, T2, and FLAIR image is Cartesian |
| Number of averages/excitations | See details in MRI Sequence parameters in this Additional file |
| Magnetic field strength | 3.0 T |
| **Reconstruction** | |
| In-plane resolution | We describe in-plane resolution as the field of view and matrix size. See details in MRI Sequence parameters in this Additional file |
| Image slice thickness | 5.0 mm |
| Image slice spacing | 1.5 mm |
| Reconstruction method | The normal reconstruction method used to reconstruct the image from the k-space information are 2D Fourier transformation. For contrast-enhanced image, we use the difference image. The asset calibration is here used during reconstruction to suppress artifacts from coil sensitivity. |
| Diffusion-weighted imaging | b = 0 and b = 1000 s/mm^2^ |
| **Image registration** | |
| Registration method | Rigid registration was performed using axial resampled T1c as a template with mutual information similarity metric |
| **Image processing** | |
| **Data conversion** | |
| ADC computation | See details in MRI Sequence parameters in this Additional file |
| **Post-acquisition processing** | |
| Anti-aliasing | None |
| Noise suppression | T1, T1c, T2 and FLAIR denoising was done by using fast non-local means tool within 3DSlicer |
| Skull stripping | A skull-stripping filter included in ITK software (<https://itk.org/>) |
| Non-uniformity correction | N4ITK-based bias field distortion correction |
| Intensity normalization | Histogram matching |
| Other post-acquisition processing methods | None |
| **Segmentation** | |
| Segmentation method | Manually |
| Conversion to mask | Directly generated by using ITK-SNAP software |
| **Image interpolation** | |
| Interpolation method | Trilinear interpolation |
| Voxel dimensions | 1x1x1 mm^3^ |
| **ROI interpolation** | |
| Interpolation method | Not applicable |
| Partially masked voxels | Not applicable, because the image was interpolated before ROI was segmented |
| **Re-segmentation** | |
| Re-segmentation methods | Not applicable, we did not use re-segmentation |
| **Discretization** | |
| Discretization method | Fix bin number |
| **Image transformation** | |
| Image filter | Wavelet and Laplacian of Gaussian filters, see details in Additional file |
| **Image biomarker computation** | |
| Biomarker set | Training data set (n = 471) |
| IBSI compliance | the software used is compliant with the IBSI benchmarks (digit phantom and radiomics CT phantom). |
| Robustness | Test-retest and multi-delineation test |
| Software availability | Pyradiomics 3.0 |
| **Image biomarker computation – texture parameters** | |
| Texture matrix aggregation | GLCM, GLRLM features were computed from a single matrix after merging all 3D directional matrices; GLSZM, GLDM and NGTDM features were computed from a 3D matrix |
| Distance weighting | No weighting |
| CM symmetry | Symmetric |
| CM distance | 1 |
| SZM linkage distance | 1 |
| DZM linkage distance | 1 |
| NGTDM distance | 1 |
| **Machine learning and radiomics analysis** | |
| Diagnostic and prognostic modeling | Our radiomics prognostic study adhered to TRIPOD reporting guideline of a multivariable prediction model for individual prognosis or diagnosis |
| Comparison with known factors | Yes, compared with clinicomolecular factors including age, gender (female or male), KPS, extent of resection (complete or incomplete), radiation therapy (yes or no) and chemotherapy (yes or no) |
| Multicollinearity | Limited the possible effect of multicollinearity by using LASSO penalized Cox proportional hazards regression in radiomics signature development; For radiomic-clinical model and clinical model, variance inflation factor (VIF) was used to assess multicollinearity. All VIFs were less than 10, indicating there were no problematic multicollinearity. |
| Model availability | Available via reasonable request from the corresponding authors |
| Data availability | Available via reasonable request from the corresponding authors |

**Table S3.** Characteristics of patients in the training set, internal validation set and external validation set.

| **Characteristic** | **Overall (n=710)** | **Training (n=471)** | **Internal validation (n=239)** | ***P*-value** | **External validation(n=91)** |
| --- | --- | --- | --- | --- | --- |
| **KPS*** | 76.75±11.98 | 76.67±11.73 | 76.90±12.49 | 0.5627 | 75.71±10.76 |
| **Age (year)*** | 55.25±11.57 | 55.11±11.71 | 55.54±11.31 | 0.7074 | 55.20±12.36 |
| **OS (month)*** | 17.36±11.94 | 17.16±11.77 | 17.76±12.29 | 0.6775 | 17.68±11.83 |
| **Sex** |  |  |  | 0.1274 |  |
| **Male** | 404(56.90%) | 258(54.80%) | 146(61.10%) |  | 46(50.50%) |
| **Female** | 306(43.10%) | 213(45.20%) | 93(38.90%) |  | 45(49.50%) |
| **Extent of Resection** |  |  |  | 0.5274 |  |
| **Complete** | 541(76.20%) | 335(75.40%) | 186(77.80%) |  | 65(71.40%) |
| **Incomplete** | 169(23.80%) | 116(24.60%) | 53(22.20%) |  | 26(28.60%) |
| **Radiation therapy** |  |  |  | 0.3409 |  |
| **Yes** | 487(68.60%) | 317(67.30%) | 170(71.10%) |  | 65(71.40%) |
| **No** | 223(31.40%) | 154(32.70%) | 69(28.90%) |  | 26(28.60%) |
| **Chemotherapy** |  |  |  | 0.5278 |  |
| **Yes** | 602(84.80%) | 396(84.10%) | 206(86.20%) |  | 63(69.20%) |
| **No** | 108(15.20%) | 75(15.90%) | 33(13.80%) |  | 28(30.80%) |

Data are numbers of patients, with percentages in parentheses. KPS = Karnofsky performance status. OS = Overall survival.

*Data are means ± standard deviations.

**Table S4.** A summary of the C-index and AIC values for OS prediction of three models.

| **Model** | **Index** | **Training set** | **Internal validation set** | **External validation set** |
| --- | --- | --- | --- | --- |
| **Radscore** | C-index | 0.707(0.683,0.731) | 0.686(0.651,0.721) | 0.691(0.63,0.751) |
|  | AIC | 3919.480 | 1747.370 | 465.630 |
| **CM nomogram** | C-index | 0.712(0.685,0.74) | 0.701(0.662,0.739) | 0.786(0.731,0.841) |
|  | AIC | 3894.888 | 1732.854 | 442.757 |
| **R-CM nomogram** | C-index | 0.772(0.749,0.794) | 0.759(0.726,0.792) | 0.839(0.797,0.88) |
|  | AIC | 3793.893 | 1691.803 | 412.034 |

CM = Clinical Model, R-CM = Radiomic Clinical Model. C-index = Concordance index. AIC = Akaike information criterion.

**Table S5.** A summary of the Radscore-related pathways enriched by GSEA. GSEA was performed using the R package clusterProfiler, querying the following annotated gene sets: Kyoto Encyclopedia of Genes and Genomes (KEGG), Hallmark, Reactome, BioCarta, Pathway Interaction Database (PID), WikiPathways. False discovery rate (FDR)-adjusted hypergeometric *P* < 0.01 indicated significantly correlated with Radscore.

| **Pathway** | **Enrichment Score** | **Enrichment's FDR** | **Core** Enrichment | **Pearson Correlation's FDR** | **Gene set** |
| --- | --- | --- | --- | --- | --- |
| KEGG_NEUROACTIVE_LIGAND_RECEPTOR_INTERACTION | 0.665748287 | 7.11E-09 | DRD1/GABRA5/GRIN1/GABRA1/GABRB2/GRM1/HTR2A/HRH3/CHRM1/GPR83/GABRG1/GABRA4/RXFP1/GRIN3A/GABRG2/ADRA1B/PRSS3/GABRD/VIPR1/GRM2/PTGER2/GRM3/CRHR1/P2RX5/GRM7/GRIN2A/HTR1B/NTSR2/GABRA2/S1PR5/GABRG3/ADRA2C/PTH2R/PTGFR/ADRA2B/ADRB1/NPFFR1/GRIN2B/SSTR1/CALCR/GLRA2/S1PR4/SSTR2/GABRB3/P2RY10/CHRM5/GABBR1/HRH2/ADRA2A/GZMA/NPY1R/FPR2/GABRB1/CHRNA1/P2RY14/LEPR/C5AR1/LPAR1/PTH1R/FPR3/HTR7/THRB/ADRA1A/PRLR/HTR2B/CHRM3/GRIN2C/PTGER3/ADORA3/S1PR1/GABBR2/F2RL2/DRD4/TACR2/GLRB/P2RY8/ADRB2/PTAFR/GRM5/C3AR1/GIPR/P2RX1/GRM8/PTGER1/P2RY13/THRA/CYSLTR1/CHRNA7/CHRM4/FPR1/OPRL1/OPRD1/PTGER4/APLNR/CYSLTR2/F2RL3/P2RY2 | 2.19E-10 | KEGG |
| REACTOME_AMINE_LIGAND_BINDING_RECEPTORS | 0.781567804 | 6.00E-05 | DRD1/HTR2A/HRH3/CHRM1/GPR143/ADRA1B/HTR1B/ADRA2C/ADRA2B/ADRB1/CHRM5/HRH2/ADRA2A/HTR7/ADRA1A/HTR2B/CHRM3/DRD4/ADRB2 | 4.83E-10 | REACTOME |
| WP_MONOAMINE_GPCRS | 0.76608457 | 0.000167329 | DRD1/HTR2A/CHRM1/ADRA1B/HTR1B/ADRA2C/ADRA2B/ADRB1/CHRM5/HRH2/ADRA2A/HTR7/ADRA1A/HTR2B/CHRM3/DRD4/ADRB2 | 5.45E-10 | WP |
| BIOCARTA_FLUMAZENIL_PATHWAY | 0.816250344 | 0.029041766 | GABRA5/GABRA1/GABRA4/GABRA2/PRKCE | 5.57E-10 | BIOCARTA |
| REACTOME_GPCR_LIGAND_BINDING | 0.642488474 | 7.11E-09 | DRD1/VIP/CCK/GRM1/HTR2A/HRH3/CHRM1/PDYN/WNT10B/RXFP1/GPR143/ADRA1B/GNG3/SST/PTGDR2/CCL19/CRHBP/CCL3/CCL3L3/VIPR1/GRM2/PTGER2/GRM3/CRHR1/CORT/GRM7/HTR1B/CXCL11/NTSR2/FFAR3/CCL4/S1PR5/CXCL12/ADRA2C/PTH2R/EDN3/PTGFR/CXCR1/ADRA2B/XK/WNT4/ADRB1/NPFFR1/PPBP/SSTR1/CALCR/TAC1/NPY/S1PR4/SSTR2/WNT16/P2RY10/CXCR2/DHH/CCL5/KEL/CXCL9/CHRM5/CXCL10/CXCL1/GABBR1/HRH2/CXCR3/ADRA2A/SUCNR1/NPY1R/CXCR6/FPR2/FZD10/P2RY12/P2RY14/PROK2/GPBAR1/C5AR1/LPAR1/CMKLR1/PTH1R/HCAR2/C5AR2/GPR37/FPR3/CD55/HTR7/QRFPR/CCR6/RAMP3/PNOC/ADRA1A/WNT2B/CXCL6/CCL28/ADGRE3/CCR1/CCR4/OXER1/HTR2B/POMC/CXCL3/CHRM3/CCRL2/HCAR3/GNGT2/PTGER3/ADORA3/S1PR1/CCR2/GPR132/GABBR2/PENK/GPR18/F2RL2/DRD4/TACR2/CCR7/FFAR2/ADRB2/TAC3/LPAR5/EDN1/PTAFR/GHRL/GRM5/C3AR1/CXCL2/GIPR/CXCL5/GRM8/PTGER1/P2RY13/CYSLTR1/NPB/GPR68/GPR183/PSAP/CHRM4/FPR1/OPRL1/CCR5/OPRD1/FFAR4/GNB5/PTGER4/APLNR/CYSLTR2/ECE2/F2RL3/P2RY2/RAMP2/CXCL8/CX3CR1/OPN3/HEBP1/GNAS/GNG11 | 1.28E-09 | REACTOME |
| WP_GPCRS_CLASS_A_RHODOPSINLIKE | 0.663912926 | 7.11E-09 | DRD1/HTR2A/HRH3/CHRM1/GPR83/ADRA1B/GPR22/PTGDR2/GPR21/PTGER2/HTR1B/NTSR2/FFAR3/ADRA2C/PTGFR/CXCR1/ADRA2B/ADRB1/NPFFR1/SSTR1/GPR174/SSTR2/P2RY10/CXCR2/CHRM5/HRH2/CXCR3/ADRA2A/SUCNR1/NPY1R/FPR2/GPR171/P2RY12/P2RY14/CMKLR1/GPR12/C5AR2/GPR37/FPR3/HTR7/CCR6/ADRA1A/CCR1/CCR4/HTR2B/CHRM3/CCRL2/HCAR3/PTGER3/ADORA3/CCR2/GPR18/F2RL2/DRD4/OR7C1/CCR7/FFAR2/GPR34/ADRB2/LPAR5/PTAFR/C3AR1/PTGER1/P2RY13/CYSLTR1/GPR68/CHRM4/GPR63/FPR1/OPRL1/CCR5/OPRD1/PTGER4/APLNR/CYSLTR2/GPR27/F2RL3/P2RY2/CX3CR1/OPN3 | 2.18E-09 | WP |
| REACTOME_SIGNALING_BY_GPCR | 0.62044368 | 7.11E-09 | PRKCG/DRD1/CAMK2A/VIP/CCK/GRM1/HTR2A/HRH3/CHRM1/PDYN/GPR83/WNT10B/RXFP1/GPR143/ADRA1B/PDE2A/GNG3/PDE1A/RASGRF2/SST/PDE1B/PTGDR2/CCL19/CRHBP/GPR150/CCL3/CCL3L3/VIPR1/GRM2/PTGER2/GRM3/PPP1R1B/CCL4L2/NGEF/CAMKK1/CRHR1/CORT/PRKCB/RGS7/GRM7/HTR1B/CXCL11/NTSR2/FFAR3/CCL4/S1PR5/CXCL12/ADRA2C/PTH2R/EDN3/PTGFR/CXCR1/ADRA2B/ARRB1/RGS4/XK/WNT4/CAMK4/ADRB1/NPFFR1/CAMK2B/PPBP/SSTR1/CALCR/TAC1/NPY/S1PR4/SSTR2/WNT16/P2RY10/CXCR2/DHH/CCL5/KEL/PDE11A/CXCL9/CHRM5/CXCL10/CXCL1/GABBR1/HRH2/RGS20/CXCR3/PAK1/DGKE/ADRA2A/SUCNR1/MCF2/NPY1R/CXCR6/FPR2/GNA14/FZD10/P2RY12/P2RY14/ITPR1/PROK2/PRKAR1B/GPBAR1/PPP3CB/CAMK2G/GNAL/C5AR1/LPAR1/ADCY1/CMKLR1/PTH1R/HCAR2/C5AR2/GPR37/FPR3/CALM1/PRKCQ/PLEKHG5/CD55/HTR7/QRFPR/ADCY4/CCR6/DGKB/RAMP3/RGS14/NET1/PNOC/PRKCE/ADRA1A/WNT2B/DGKZ/CXCL6/ADCY5/RGS5/CAMKK2/GRK3/CCL28/ADGRE3/PPP3R1/FGD2/CCR1/CCR4/GNAI1/OXER1/RGS2/HTR2B/POMC/ARHGEF37/CXCL3/CHRM3/RGS10/CCRL2/HCAR3/RGS18/ADCY2/KALRN/MAPK3/RGS22/PRKACB/GNGT2/PTGER3/ADORA3/S1PR1/CCR2/GPR132/GABBR2/PENK/PRKAR2B/GPR18/F2RL2/MCF2L/DRD4/PRKCD/ARHGEF15/TACR2/PLCB4/VAV1/CCR7/RGS1/FFAR2/PIK3R6/RPS6KA2/GPSM3/ADRB2/TAC3/LPAR5/RGS11/PRKCH/EDN1/PIK3R5/PTAFR/GHRL/PPP3CA/GRM5/BTK/C3AR1/ARRB2/CXCL2/GIPR/CXCL5/GRM8/PTGER1/P2RY13/CYSLTR1/NPB/ABR/GPR68/PLCB2/GPR183/PSAP/PDE1C/GNA15/CHRM4/FPR1/OPRL1/CCR5/GPR84/OPRD1/FFAR4/PLCB1/GNB5/PTGER4/ARHGEF33/APLNR/CYSLTR2/GPR27/ECE2/PDE4A/F2RL3/GRK2/P2RY2/RAMP2/CXCL8/NBEA/CX3CR1/GNAZ/ABHD6/OPN3/HEBP1/GNAS/GNG11/RPS6KA1/PDE7B/ITSN1 | 2.63E-09 | REACTOME |
| REACTOME_SUMOYLATION_OF_DNA_DAMAGE_RESPONSE_AND_REPAIR_PROTEINS | -0.517927959 | 6.60E-06 | RANBP2/BMI1/RNF168/UBE2I/HERC2/NUP54/RING1/RNF2/NUP58/SCMH1/NUP62/PIAS4/NUP133/NUP37/NUP43/RAE1/RPA1/NUP50/PHC3/NUP88/PARP1/NUP98/PHC2/NUP85/NUP93/CBX8/TDG/STAG2/PIAS2/NUP153/SMC3/POM121/RAD52/SMC6/TPR/AAAS/MDC1/PCGF2/STAG1/WRN/NUP155/NUP188/SMC5/POM121C/NUP35/NDC1/NUP205/NUP160/SMC1A/BRCA1/NUP42/PHC1/NUP210/NUP107/BLM/CBX2 | 2.75E-09 | REACTOME |
| REACTOME_G_ALPHA_I_SIGNALLING_EVENTS | 0.670610431 | 7.11E-09 | PRKCG/CAMK2A/PDYN/GNG3/PDE1A/SST/PDE1B/PTGDR2/CCL19/GRM2/GRM3/PPP1R1B/CCL4L2/CAMKK1/CORT/RGS7/GRM7/HTR1B/CXCL11/CCL4/S1PR5/CXCL12/ADRA2C/CXCR1/ADRA2B/RGS4/CAMK4/CAMK2B/PPBP/SSTR1/NPY/S1PR4/SSTR2/CXCR2/CCL5/CXCL9/CXCL10/CXCL1/GABBR1/RGS20/CXCR3/ADRA2A/SUCNR1/NPY1R/CXCR6/FPR2/GNA14/P2RY12/P2RY14/ITPR1/PRKAR1B/PPP3CB/CAMK2G/GNAL/C5AR1/LPAR1/ADCY1/HCAR2/GPR37/FPR3/CALM1/ADCY4/CCR6/RGS14/PNOC/CXCL6/ADCY5/RGS5/CAMKK2/CCL28/PPP3R1/CCR1/CCR4/GNAI1/OXER1/POMC/CXCL3/RGS10/HCAR3/RGS18/ADCY2/RGS22/PRKACB/GNGT2/PTGER3/ADORA3/CCR2/GABBR2/PENK/PRKAR2B/GPR18/DRD4/PRKCD/PLCB4/CCR7/RGS1/GPSM3/LPAR5/RGS11/PPP3CA/C3AR1/CXCL2/CXCL5/GRM8/P2RY13/NPB/PLCB2/GPR183/PSAP/PDE1C/GNA15/CHRM4/FPR1/OPRL1/CCR5/OPRD1/PLCB1/GNB5/APLNR/PDE4A/GRK2/CXCL8/NBEA/CX3CR1/GNAZ/OPN3/HEBP1/GNAS/GNG11 | 3.14E-09 | REACTOME |
| REACTOME_ADORA2B_MEDIATED_ANTI_INFLAMMATORY_CYTOKINES_PRODUCTION | 0.666748577 | 1.95E-07 | DRD1/VIP/GPR83/RXFP1/GNG3/GPR150/VIPR1/PTGER2/CRHR1/PTH2R/ADRB1/IL6/CALCR/HRH2/PRKAR1B/GPBAR1/ADCY1/PTH1R/HTR7/ADCY4/RAMP3/ADCY5/GNAI1/POMC/ADCY2/PRKACB/GNGT2/PRKAR2B/ADRB2/GIPR/GPR84/GNB5/PTGER4/CYSLTR2/GPR27/RAMP2/GNAZ/GNAS/GNG11 | 4.00E-09 | REACTOME |
| REACTOME_STIMULI_SENSING_CHANNELS | 0.536398951 | 0.002924615 | CASQ2/RYR2/ANO3/TRPV6/CLCA4/ANO9/TRPM6/ASIC2/ANO4/SGK2/CALM1/BEST1/CLCN4/MLKL/TRPM3/CLIC2/WNK2/MCOLN2/ANO1/TRPM2/FKBP1B/RIPK3/ANO2/NALCN/RYR1/TRPV2/SGK1/UNC80/TRPM8/SGK3/CASQ1/TRPV4 | 4.05E-09 | REACTOME |
| REACTOME_G_ALPHA_S_SIGNALLING_EVENTS | 0.662400486 | 4.74E-08 | DRD1/VIP/GPR83/RXFP1/PDE2A/GNG3/PDE1A/PDE1B/GPR150/VIPR1/PTGER2/CRHR1/PTH2R/ARRB1/ADRB1/CALCR/PDE11A/HRH2/GPBAR1/ADCY1/PTH1R/HTR7/ADCY4/RAMP3/ADCY5/GRK3/GNAI1/POMC/ADCY2/GNGT2/ADRB2/ARRB2/GIPR/GPR84/GNB5/PTGER4/CYSLTR2/GPR27/PDE4A/GRK2/RAMP2/GNAZ/GNAS/GNG11/PDE7B | 5.42E-09 | REACTOME |
| REACTOME_CLASS_A_1_RHODOPSIN_LIKE_RECEPTORS | 0.669054307 | 7.11E-09 | DRD1/CCK/HTR2A/HRH3/CHRM1/PDYN/RXFP1/GPR143/ADRA1B/SST/PTGDR2/CCL19/CCL3/CCL3L3/PTGER2/CORT/HTR1B/CXCL11/NTSR2/FFAR3/CCL4/S1PR5/CXCL12/ADRA2C/EDN3/PTGFR/CXCR1/ADRA2B/XK/ADRB1/NPFFR1/PPBP/SSTR1/TAC1/NPY/S1PR4/SSTR2/P2RY10/CXCR2/CCL5/KEL/CXCL9/CHRM5/CXCL10/CXCL1/HRH2/CXCR3/ADRA2A/SUCNR1/NPY1R/CXCR6/FPR2/P2RY12/P2RY14/PROK2/GPBAR1/C5AR1/LPAR1/CMKLR1/HCAR2/C5AR2/GPR37/FPR3/HTR7/QRFPR/CCR6/PNOC/ADRA1A/CXCL6/CCL28/CCR1/CCR4/OXER1/HTR2B/POMC/CXCL3/CHRM3/CCRL2/HCAR3/PTGER3/ADORA3/S1PR1/CCR2/GPR132/PENK/GPR18/F2RL2/DRD4/TACR2/CCR7/FFAR2/ADRB2/TAC3/LPAR5/EDN1/PTAFR/GHRL/C3AR1/CXCL2/CXCL5/PTGER1/P2RY13/CYSLTR1/NPB/GPR68/GPR183/PSAP/CHRM4/FPR1/OPRL1/CCR5/OPRD1/FFAR4/PTGER4/APLNR/CYSLTR2/ECE2/F2RL3/P2RY2/CXCL8/CX3CR1/OPN3/HEBP1 | 8.88E-09 | REACTOME |
| REACTOME_SUMOYLATION_OF_RNA_BINDING_PROTEINS | -0.606740942 | 3.33E-05 | RANBP2/BMI1/UBE2I/NUP54/RING1/RNF2/NUP58/SCMH1/HNRNPK/NUP62/NUP133/NUP37/NUP43/RAE1/NUP50/PHC3/NUP88/NUP98/PHC2/NUP85/NUP93/CBX8/NUP153/POM121/TPR/AAAS/PCGF2/NOP58/NUP155/NUP188/POM121C/NUP35/NDC1/NUP205/NUP160/NUP42/PHC1/NUP210/NUP107/CBX2 | 1.03E-08 | REACTOME |
| WP_GPCRS_OTHER | 0.637364253 | 0.000126528 | GRM1/HTR2A/GPR83/RXFP1/GPR143/GPR88/GPR61/GPR62/PTGFR/CXCR1/LGR6/SSTR2/CXCR2/CXCR3/C5AR2/HTR7/ADGRE3/CHRM3/OR2A20P/ADORA3/S1PR1/GPR132/GPR18/DRD4/ADRB2/GRM8/P2RY13/ADGRD1/GPR183/ADGRF5/CCR5/GPR84 | 1.09E-08 | WP |
| REACTOME_SUMOYLATION_OF_UBIQUITINYLATION_PROTEINS | -0.58036997 | 0.000443301 | MDM2/RANBP2/UBE2I/NUP54/TRIM27/NUP58/NUP62/PIAS4/NUP133/NUP37/NUP43/RAE1/NUP50/NUP88/NUP98/NUP85/NUP93/VHL/PIAS2/NUP153/POM121/TPR/AAAS/NUP155/NUP188/POM121C/NUP35/NDC1/NUP205/NUP160/NUP42/NUP210/NUP107 | 1.20E-08 | REACTOME |
| REACTOME_SUMOYLATION_OF_CHROMATIN_ORGANIZATION_PROTEINS | -0.516437057 | 9.37E-05 | NUP214/H4C15/HDAC1/SUMO2/RANBP2/BMI1/UBE2I/NUP54/RING1/RNF2/NUP58/SCMH1/NUP62/NUP133/NUP37/NUP43/RAE1/NUP50/PHC3/NUP88/NUP98/PHC2/H4C5/NUP85/SATB2/NUP93/CBX8/HDAC2/H4C14/PIAS2/NUP153/POM121/H4-16/TPR/AAAS/ZBED1/PCGF2/H4C9/CBX5/NUP155/NUP188/SUZ12/POM121C/NUP35/NDC1/NUP205/NUP160/NUP42/PHC1/NUP210/NUP107/H4C11/CBX2 | 1.29E-08 | REACTOME |
| REACTOME_SNRNP_ASSEMBLY | -0.606411988 | 2.87E-06 | NCBP2/NUP58/NUP62/NUP133/NUP37/NUP43/RAE1/NUP50/SNRPE/NUP88/NCBP1/NUP98/SNRPF/GEMIN6/SNRPD1/SMN1/NUP85/NUP93/SNRPG/TGS1/GEMIN2/GEMIN7/GEMIN4/GEMIN5/NUP153/PRMT5/DDX20/POM121/TPR/AAAS/SNRPB/NUP155/NUP188/POM121C/NUP35/NDC1/NUP205/NUP160/NUP42/NUP210/NUP107 | 1.59E-08 | REACTOME |
| REACTOME_INWARDLY_RECTIFYING_K_CHANNELS | 0.716161239 | 0.000312209 | KCNJ12/KCNJ3/GNG3/KCNJ6/KCNJ4/ABCC8/KCNJ11/KCNJ9/GABBR1/KCNJ5/KCNJ15/GNGT2/GABBR2 | 2.22E-08 | REACTOME |
| REACTOME_NS1_MEDIATED_EFFECTS_ON_HOST_PATHWAYS | -0.530295703 | 0.004196395 | NUP58/NUP62/NUP133/NUP37/NUP43/RAE1/NUP50/NUP88/NUP98/NUP85/NUP93/CPSF4/KPNB1/NUP153/POM121/TPR/AAAS/NUP155/NUP188/POM121C/NUP35/NDC1/NUP205/KPNA2/NUP160/NUP42/NUP210/NUP107 | 2.52E-08 | REACTOME |
| REACTOME_SUMOYLATION_OF_SUMOYLATION_PROTEINS | -0.599757465 | 0.00054953 | NUP214/SUMO2/RANBP2/UBE2I/NUP54/NUP58/NUP62/PIAS4/NUP133/NUP37/NUP43/RAE1/NUP50/NUP88/NUP98/NUP85/NUP93/NUP153/POM121/TPR/AAAS/NUP155/NUP188/POM121C/NUP35/NDC1/NUP205/NUP160/NUP42/NUP210/NUP107 | 3.11E-08 | REACTOME |
| WP_BENZOAPYRENE_METABOLISM | 0.832917584 | 0.037035719 | AKR1C2/CYP1B1/AKR1C1/AKR1C3/EPHX1 | 3.60E-08 | WP |
| REACTOME_TRANSPORT_OF_MATURE_MRNAS_DERIVED_FROM_INTRONLESS_TRANSCRIPTS | -0.600594681 | 9.72E-05 | NUP54/NCBP2/NUP58/NUP62/NUP133/NUP37/NUP43/RAE1/NUP50/NUP88/NCBP1/CPSF1/NUP98/NUP85/NUP93/CPSF4/WDR33/NUP153/CPSF3/POM121/TPR/AAAS/NUP155/NUP188/ALYREF/POM121C/FIP1L1/NUP35/NDC1/NUP205/NUP160/NUP42/NUP210/NUP107 | 4.19E-08 | REACTOME |
| WP_DNA_REPAIR_PATHWAYS_FULL_NETWORK | -0.513284943 | 7.11E-09 | CENPX/XRCC5/XRCC1/RFC1/MNAT1/RPA1/MSH2/POLE3/RPA3/CUL4B/PARP1/HMGB1/ATR/RAD23B/RAD51C/MUTYH/FAAP100/TDG/RFC2/APEX1/LIG3/ERCC4/GTF2H4/GTF2H2C/POLD3/GTF2H2/POLI/RAD52/FANCG/PALB2/UNG/H2AX/RFC5/POLD1/WRN/MSH6/RAD54B/PRKDC/USP1/PCNA/FAAP24/FANCE/FEN1/POLH/LIG1/FANCM/RFC4/BRCA1/FANCA/POLE2/FANCC/RFC3/POLE/RAD51/FANCD2/FANCB/REV3L/NEIL3/CHEK1/BRCA2/FANCI/BRIP1/EXO1 | 4.34E-08 | WP |
| REACTOME_TRNA_PROCESSING | -0.489312357 | 5.85E-07 | TRNT1/NSUN2/NUP58/PRORP/TRMT10A/NUP62/NUP133/NUP37/PUS1/TRMT12/TSEN2/QTRT1/NUP43/RAE1/RPP40/THG1L/THADA/RPPH1/NUP50/WDR4/NUP88/CPSF1/C2orf49/TP53RK/NUP98/HSD17B10/TPRKB/ADAT1/EPRS1/TYW1/GTPBP3/NUP85/TRMT61B/TRMU/CDKAL1/FAM98B/NUP93/TRMT5/CPSF4/ALKBH8/TRMT10C/POP5/LCMT2/TYW5/TRMT6/QTRT2/NUP153/TRMT13/POP1/POM121/XPOT/TPR/AAAS/CSTF2/PUS3/NUP155/NUP188/PUS7/POM121C/NUP35/NDC1/NUP205/NUP160/NUP42/NUP210/NUP107/METTL1 | 4.60E-08 | REACTOME |
| REACTOME_TRANSPORT_OF_MATURE_TRANSCRIPT_TO_CYTOPLASM | -0.542876784 | 9.05E-07 | NUP54/POLDIP3/SRSF2/NCBP2/EIF4A3/NUP58/RNPS1/SRSF3/NUP62/NUP133/NUP37/NUP43/DHX38/RAE1/MAGOH/DDX39B/NUP50/NUP88/GLE1/NCBP1/CPSF1/CHTOP/SRSF9/NUP98/NXT1/SRSF6/SRSF11/NUP85/NUP93/RBM8A/CPSF4/WDR33/SRSF1/ZC3H11A/U2AF2/THOC1/MAGOHB/NUP153/CPSF3/DDX39A/UPF3B/U2AF1/POM121/TPR/AAAS/THOC2/NUP155/NUP188/ALYREF/POM121C/FIP1L1/NUP35/NDC1/NUP205/NUP160/NUP42/NUP210/NUP107 | 4.62E-08 | REACTOME |
| HALLMARK_KRAS_SIGNALING_DN | 0.577055187 | 1.97E-06 | SNCB/RYR2/ARHGDIG/SLC30A3/MYOT/PNMT/KCNN1/CACNA1F/HTR1B/ABCG4/ADRA2C/CPEB3/PTGFR/SIDT1/MAST3/EFHD1/ITIH3/WNT16/NR4A2/SELENOP/ARPP21/NRIP2/TFCP2L1/PDCD1/CAMK1D/PRODH/MYO15A/TEX15/CYP39A1/SPTBN2/THRB/FGF22/LYPD3/RSAD2/DTNB/MEFV/PKP1/TENM2/CD80/SLC25A23/MFSD6/ZBTB16/CLSTN3/HSD11B2/MX1/RGS11/RYR1/GPRC5C/EDN1/SGK1/CCNA1/PDE6B/PRKN/SLC38A3/KCNQ2/SLC29A3 | 4.74E-08 | HALLMARK |
| REACTOME_TRNA_PROCESSING_IN_THE_NUCLEUS | -0.495685828 | 0.001089803 | RANBP2/CLP1/RPP14/TSEN15/NUP54/TRNT1/NUP58/NUP62/NUP133/NUP37/TSEN2/NUP43/RAE1/RPP40/RPPH1/NUP50/NUP88/CPSF1/C2orf49/NUP98/NUP85/FAM98B/NUP93/CPSF4/POP5/NUP153/POP1/POM121/XPOT/TPR/AAAS/CSTF2/NUP155/NUP188/POM121C/NUP35/NDC1/NUP205/NUP160/NUP42/NUP210/NUP107 | 5.65E-08 | REACTOME |
| WP_SEROTONIN_AND_ANXIETY | 0.778016079 | 0.005325766 | GABRA1/GRM1/HTR2A/PRKCB/CAMK2B/ADRA1A/PLEK/POMC | 5.68E-08 | WP |
| REACTOME_INTERACTIONS_OF_VPR_WITH_HOST_CELLULAR_PROTEINS | -0.54112699 | 0.002325473 | NUP58/NUP62/NUP133/NUP37/NUP43/RAE1/NUP50/NUP88/NUP98/NUP85/NUP93/NUP153/POM121/TPR/AAAS/HMGA1/NUP155/NUP188/POM121C/NUP35/NDC1/NUP205/NUP160/NUP42/NUP210/NUP107 | 6.44E-08 | REACTOME |
| REACTOME_EXPORT_OF_VIRAL_RIBONUCLEOPROTEINS_FROM_NUCLEUS | -0.562655153 | 0.002481558 | NUP58/NUP62/NUP133/NUP37/NUP43/RAE1/NUP50/NUP88/XPO1/NUP98/NUP85/NUP93/NUP153/POM121/TPR/AAAS/NUP155/NUP188/POM121C/NUP35/NDC1/NUP205/NUP160/NUP42/NUP210/NUP107 | 6.57E-08 | REACTOME |
| KEGG_CALCIUM_SIGNALING_PATHWAY | 0.633570571 | 7.11E-09 | PRKCG/DRD1/CAMK2A/GRIN1/SLC8A2/GRM1/CACNA1B/HTR2A/ATP2B3/RYR2/CACNA1I/CHRM1/ADRA1B/PDE1A/PDE1B/P2RX5/PRKCB/CACNA1F/GRIN2A/PLN/PTGFR/CAMK4/ADRB1/CAMK2B/ATP2B2/CHRM5/ITPKA/HRH2/GNA14/ITPR1/PPP3CB/CAMK2G/GNAL/ADCY1/CALM1/HTR7/ADCY4/ADRA1A/CALM3/PPP3R1/HTR2B/CHRM3/ADCY2/GRIN2C/PRKACB/PTGER3/TACR2/PLCB4/ATP2A3/ADRB2/NOS3/RYR1/CD38/PDGFRB/CACNA1E/PTAFR/PPP3CA/GRM5/CACNA1D/PLCG2/P2RX1/PTGER1/CYSLTR1/PTK2B/CHRNA7/PLCB2/PDE1C/GNA15/ATP2B1/BST1/SLC25A4/PLCB1/CYSLTR2/MYLK | 6.60E-08 | KEGG |
| KEGG_RNA_DEGRADATION | -0.399553027 | 0.022308518 | MPHOSPH6/PNPT1/TTC37/EXOSC7/LSM6/PAPOLA/XRN1/EXOSC5/LSM1/EXOSC10/ENO1/MTREX/DCP1B/CNOT2/EXOSC3/DCP1A/HSPA9/DCPS/CNOT10/HSPD1/EXOSC4/CNOT3/EDC4/EXOSC8/DDX6/EXOSC2/CNOT9/CNOT4/SKIV2L/TENT4A/CNOT6/EXOSC9/DIS3/CNOT1/LSM5/LSM7/XRN2/EDC3/PATL1/ENO3/LSM8 | 6.77E-08 | KEGG |
| REACTOME_NUCLEAR_IMPORT_OF_REV_PROTEIN | -0.623287463 | 0.000223808 | NPM1/RANBP2/NUP54/NUP58/NUP62/NUP133/NUP37/NUP43/RAE1/NUP50/NUP88/NUP98/NUP85/NUP93/KPNB1/NUP153/POM121/TPR/AAAS/NUP155/NUP188/POM121C/NUP35/NDC1/NUP205/NUP160/RCC1/NUP42/NUP210/NUP107 | 7.65E-08 | REACTOME |
| REACTOME_TRANSPORT_OF_THE_SLBP_DEPENDANT_MATURE_MRNA | -0.601800971 | 0.000209103 | NUP54/NCBP2/NUP58/NUP62/NUP133/NUP37/NUP43/RAE1/NUP50/NUP88/NCBP1/NUP98/NUP85/NUP93/NUP153/POM121/TPR/AAAS/NUP155/NUP188/ALYREF/POM121C/NUP35/NDC1/NUP205/NUP160/NUP42/NUP210/NUP107 | 7.76E-08 | REACTOME |
| REACTOME_REGULATION_OF_GLUCOKINASE_BY_GLUCOKINASE_REGULATORY_PROTEIN | -0.588136185 | 0.002133254 | NUP58/NUP62/NUP133/NUP37/NUP43/RAE1/NUP50/NUP88/NUP98/NUP85/NUP93/NUP153/POM121/TPR/AAAS/GCKR/NUP155/NUP188/POM121C/NUP35/NDC1/NUP205/NUP160/NUP42/NUP210/NUP107 | 8.54E-08 | REACTOME |
| REACTOME_ION_CHANNEL_TRANSPORT | 0.573207608 | 4.35E-07 | CAMK2A/CASQ2/ATP2B3/RYR2/ANO3/TRPV6/CLCA4/ATP8A2/PLN/ANO9/TRPM6/ASIC2/ATP6V1G2/CAMK2B/FXYD7/ATP8A1/ATP1A3/ATP1B1/ATP2B2/ANO4/SGK2/ATP10A/CAMK2G/CALM1/BEST1/CLCN4/MLKL/TRPM3/CLIC2/ATP6V1H/FXYD1/WNK2/MCOLN2/ANO1/TRPM2/ATP6V1E1/FKBP1B/RIPK3/ATP2A3/ATP6V1B2/ANO2/ATP1A2/ATP6V1A/NALCN/RYR1/TRPV2/SGK1/UNC80/ATP6V0A1/TRPM8/ATP6V0C/SGK3/FXYD2/ATP6V1C1/ATP8B4/CASQ1/TRPV4/ATP2B1/SLN/ATP8B1/ATP2C2/TRPM4/TCIRG1/STOM/ATP6V0D1/TTYH2/ATP6V1D/TSC22D3/ATP6V0B/ATP10B/WWP1/TPCN1 | 8.55E-08 | REACTOME |
| REACTOME_ARACHIDONIC_ACID_METABOLISM | 0.623468422 | 0.003430373 | PTGDS/CYP1B1/FAAH/CYP4F3/LTC4S/HPGD/AKR1C3/DPEP2/CYP2J2/HPGDS/PTGIS/PTGES/CYP2C8/TBXAS1/GPX1/CBR1/GPX4/PTGS2/PON3/ALOX5/GGT5/CYP4F11/GGT1/PTGES2 | 8.94E-08 | REACTOME |
| REACTOME_INTERACTIONS_OF_REV_WITH_HOST_CELLULAR_PROTEINS | -0.589515357 | 0.000326336 | NUP58/NUP62/NUP133/NUP37/NUP43/RAE1/NUP50/NUP88/XPO1/NUP98/NUP85/NUP93/KPNB1/NUP153/RANBP1/POM121/TPR/AAAS/NUP155/NUP188/POM121C/NUP35/NDC1/NUP205/NUP160/RCC1/NUP42/NUP210/NUP107 | 9.11E-08 | REACTOME |
| WP_DNA_IRDAMAGE_AND_CELLULAR_RESPONSE_VIA_ATR | -0.615258685 | 7.11E-09 | TP53BP1/PARP1/ATRIP/ATR/TDP1/DCLRE1A/UIMC1/SP1/CLK2/MCPH1/HUS1/TRIM28/RFWD3/RMI1/CEP164/RAD52/PALB2/H2AX/MDC1/BCL6/TOP3A/WRN/RECQL/TP53/PRKDC/USP1/PCNA/FEN1/SMC1A/TOPBP1/BRCA1/RBBP8/BARD1/FANCA/E2F1/RAD51/FANCD2/CDK2/MCM2/CHEK1/BRCA2/PLK1/CDC25C/CDK1/FANCI/CDC45/FOXM1/CLSPN/BRIP1/EXO1 | 9.70E-08 | WP |
| REACTOME_ADRENOCEPTORS | 0.813961925 | 0.030646212 | ADRA1B/ADRA2C/ADRA2B/ADRB1/ADRA2A/ADRA1A/ADRB2 | 1.01E-07 | REACTOME |
| REACTOME_SUMOYLATION | -0.315412768 | 0.002607187 | NUP62/PIAS4/SAFB/NUP133/NUP37/NUP43/RAE1/RPA1/MBD1/NUP50/UBA2/PHC3/NUP88/TP53BP1/PARP1/SP3/NUP98/PHC2/H4C5/CREBBP/TOP2B/MTA1/NUP85/DAXX/SATB2/ZNF350/NCOR2/NUP93/CBX8/VHL/HDAC2/RELA/TDG/AR/EP300/TRIM28/H4C14/STAG2/PIAS2/NUP153/DNMT3A/RARA/SIN3A/SMC3/POM121/TOP1/RAD52/H4-16/SMC6/TPR/AAAS/ZNF131/ZBED1/MDC1/PCGF2/NOP58/H4C9/STAG1/CBX5/WRN/NUP155/TP53/NUP188/SMC5/SUZ12/POM121C/PCNA/NUP35/NDC1/DNMT1/NUP205/NUP160/SENP1/INCENP/SMC1A/BRCA1/NUP42/PHC1/TFAP2A/NUP210/DNMT3B/AURKA/NUP107/H4C11/BLM/CDCA8/AURKB/BIRC5/CBX2/TOP2A | 1.08E-07 | REACTOME |
| REACTOME_EPIGENETIC_REGULATION_OF_GENE_EXPRESSION | -0.466217151 | 1.62E-06 | UBTF/BAZ2A/GSK3B/POLR2H/MNAT1/DDX21/GATAD2A/H3C6/TAF1B/MTF2/RBBP7/H4C5/H2BC11/H2AZ1/MYBBP1A/H2AZ2/SF3B1/MTA1/MTA2/RBBP4/SMARCA5/HDAC2/TDG/CHD4/SAP130/EP300/AEBP2/H2BC5/TTF1/TET2/GATAD2B/H4C14/GTF2H4/POLR1C/DEK/DNMT3A/SIN3A/GTF2H2/CBX3/H4-16/PHF19/TAF1A/JARID2/H2AX/H2AC8/H4C9/BAZ1B/POLR1A/SUZ12/SUV39H1/DNMT1/H3C10/POLR1B/TET3/H2AC20/DNMT3B/TET1/H4C11/H2BC6/H3C14/H3C15/EZH2/H2BC9/UHRF1 | 1.10E-07 | REACTOME |
| REACTOME_CGMP_EFFECTS | 0.717784389 | 0.016539524 | PDE2A/PDE1A/PDE1B/PDE11A/ITPR1/KCNMB4/IRAG1/KCNMA1/PRKG1/PDE5A/KCNMB2 | 1.23E-07 | REACTOME |
| REACTOME_DEADENYLATION_DEPENDENT_MRNA_DECAY | -0.387095221 | 0.035303587 | DCP1B/CNOT2/EXOSC3/DCP1A/DCPS/CNOT10/EIF4A3/EXOSC4/PAIP1/CNOT3/EDC4/EXOSC8/DDX6/EXOSC2/EIF4A1/TNKS1BP1/CNOT9/CNOT4/EIF4G1/SKIV2L/CNOT6/EXOSC9/DIS3/CNOT1/LSM5/LSM7/PAN2/EDC3/PATL1/TUT4 | 1.37E-07 | REACTOME |
| REACTOME_VIRAL_MESSENGER_RNA_SYNTHESIS | -0.536035909 | 0.001330653 | NUP58/NUP62/NUP133/NUP37/POLR2H/NUP43/RAE1/NUP50/NUP88/POLR2A/NUP98/POLR2J/NUP85/NUP93/NUP153/POLR2B/POM121/POLR2D/TPR/AAAS/NUP155/NUP188/POM121C/NUP35/NDC1/NUP205/NUP160/NUP42/NUP210/NUP107 | 1.38E-07 | REACTOME |
| REACTOME_SUMOYLATION_OF_DNA_METHYLATION_PROTEINS | -0.61310939 | 0.043641688 | BMI1/UBE2I/RING1/RNF2/SCMH1/PHC3/PHC2/CBX8/DNMT3A/PCGF2/DNMT1/PHC1/DNMT3B/CBX2 | 1.43E-07 | REACTOME |
| REACTOME_HDR_THROUGH_SINGLE_STRAND_ANNEALING_SSA | -0.633597512 | 3.70E-05 | RAD1/RPA1/RAD9B/RPA3/ABL1/ATRIP/ATR/HUS1/RFC2/ERCC4/RMI1/RHNO1/RAD52/RFC5/TOP3A/WRN/TOPBP1/RFC4/BRCA1/RBBP8/BARD1/RFC3/DNA2/RAD51/BLM/BRIP1/EXO1 | 1.50E-07 | REACTOME |
| REACTOME_NUCLEAR_PORE_COMPLEX_NPC_DISASSEMBLY | -0.613623701 | 0.000127222 | NUP58/NUP62/NUP133/NUP37/NUP43/RAE1/NUP50/NUP88/NUP98/NUP85/NUP93/NUP153/NEK6/POM121/TPR/AAAS/NUP155/NUP188/POM121C/NUP35/NDC1/NUP205/NUP160/NUP42/NUP210/NUP107/CCNB1/CDK1/CCNB2 | 1.53E-07 | REACTOME |
| WP_NUCLEOTIDE_EXCISION_REPAIR_IN_XERODERMA_PIGMENTOSUM | -0.471014057 | 0.000439868 | POLK/XAB2/GTF2H3/ERCC3/USP7/ERCC8/HMGN1/XRCC1/CHD1L/RFC1/MNAT1/RPA1/POLE3/RPA3/CUL4B/PARP1/H4C5/RAD23B/SLX4IP/RFC2/LIG3/ERCC4/H4C14/GTF2H4/GTF2H2C/POLD3/GTF2H2/H4-16/H4C9/RFC5/POLD1/SLX4/UVSSA/PCNA/POLH/LIG1/RFC4/BRCA1/RAD18/POLE2/H4C11/RFC3/POLE | 1.59E-07 | WP |
| WP_MRNA_PROCESSING | -0.411795119 | 5.68E-05 | TMED10/SF3A3/PSKH1/SRSF2/SRP54/CLASRP/NCBP2/SNRNP40/SUPT5H/HNRNPK/CSTF1/CELF1/RNPS1/SRSF3/HNRNPM/SNRPA1/DHX38/PRPF6/SF3B2/SNRPE/HNRNPL/NCBP1/CPSF1/SNRPB2/POLR2A/HNRNPA1/SRSF9/DHX8/SUGP2/SNRPF/SRSF10/RBM39/SRSF6/SNRPA/SNRPD1/SF3B1/NUDT21/PCBP2/RNMT/TRA2B/SNRPG/SF3B3/CLK2/SF3B4/METTL3/SRPK1/CPSF4/EFTUD2/SRSF1/YBX1/SFSWAP/U2AF2/HNRNPD/PPM1G/LSM7/HNRNPA2B1/HNRNPU/DHX15/PRPF4B/CPSF3/DHX9/SNRNP70/XRN2/SFPQ/DDX20/HNRNPR/PTBP1/PRPF8/PRPF40A/U2AF1/FUS/HNRNPAB/CSTF2/RBMX/PRPF3/PRPF4/HNRNPH1/SREK1/NONO/SF3A2/SNRPB/SMC1A | 1.63E-07 | WP |
| REACTOME_PROCESSING_OF_CAPPED_INTRONLESS_PRE_MRNA | -0.514035192 | 0.018322044 | SNRPD3/PABPN1/PAPOLA/SLBP/CSTF3/SYMPK/CLP1/NCBP2/CSTF1/SNRPE/NCBP1/CPSF1/SNRPF/NUDT21/CPSF7/SNRPG/CPSF4/WDR33/PCF11/CPSF3/CSTF2/ZNF473/SNRPB/FIP1L1 | 1.86E-07 | REACTOME |
| REACTOME_NITRIC_OXIDE_STIMULATES_GUANYLATE_CYCLASE | 0.697537261 | 0.007697503 | PDE2A/PDE1A/PDE1B/PDE11A/GUCY1B1/ITPR1/KCNMB4/GUCY1A1/IRAG1/KCNMA1/NOS3/PRKG1/PDE5A/KCNMB2 | 1.87E-07 | REACTOME |
| BIOCARTA_ATRBRCA_PATHWAY | -0.613241662 | 0.008425638 | FANCG/TP53/FANCE/BRCA1/FANCA/FANCC/RAD51/FANCD2/CHEK1/BRCA2 | 1.90E-07 | BIOCARTA |
| REACTOME_ANTI_INFLAMMATORY_RESPONSE_FAVOURING_LEISHMANIA_PARASITE_INFECTION | 0.642615197 | 7.11E-09 | DRD1/VIP/GPR83/RXFP1/GNG3/GPR150/VIPR1/IL10/PTGER2/CRHR1/PTH2R/ADRB1/IL6/CALCR/IGHG4/HRH2/ITPR1/PRKAR1B/GPBAR1/CD247/ADCY1/PTH1R/CALM1/HTR7/ADCY4/RAMP3/ADCY5/CD163/GNAI1/PLK2/DPEP2/POMC/CD3G/HCK/ADCY2/PRKACB/GNGT2/PRKAR2B/FCGR3A/SYK/ADRB2/FCGR2A/PLCG2/GIPR/RHBDF2/CYSLTR1/FCGR1A/GPR84/GNB5/PTGER4/CYSLTR2/GPR27/RAMP2/GNAZ/GNAS/GNG11/LYN | 2.16E-07 | REACTOME |
| REACTOME_RNA_POLYMERASE_II_TRANSCRIPTION_TERMINATION | -0.474786595 | 0.000771187 | SARNP/CSTF3/SRSF4/SYMPK/CLP1/POLDIP3/SRSF2/NCBP2/EIF4A3/CSTF1/RNPS1/SRSF3/DHX38/MAGOH/DDX39B/SNRPE/NCBP1/CPSF1/CHTOP/SRSF9/SNRPF/SRSF6/SRSF11/NUDT21/CPSF7/SNRPG/RBM8A/CPSF4/WDR33/SRSF1/ZC3H11A/PCF11/U2AF2/THOC1/MAGOHB/CPSF3/DDX39A/UPF3B/U2AF1/CSTF2/THOC2/ZNF473/SNRPB/ALYREF/FIP1L1 | 2.30E-07 | REACTOME |
| REACTOME_CLASS_B_2_SECRETIN_FAMILY_RECEPTORS | 0.569617924 | 0.003331613 | VIP/WNT10B/GNG3/CRHBP/VIPR1/CRHR1/PTH2R/WNT4/CALCR/WNT16/DHH/FZD10/PTH1R/CD55/RAMP3/WNT2B/ADGRE3/GNGT2/GIPR/GNB5/CYSLTR2/RAMP2/GNAS/GNG11/WNT9B/WNT6/WNT10A/ADGRE1/FZD4/GNG7/SHH/WNT5A | 2.31E-07 | REACTOME |
| REACTOME_HDR_THROUGH_HOMOLOGOUS_RECOMBINATION_HRR | -0.595540718 | 2.14E-07 | MUS81/RAD1/RFC1/RPA1/POLE3/RAD9B/RPA3/ATRIP/ATR/RTEL1/RAD51C/HUS1/RFC2/XRCC3/RMI1/POLD3/RHNO1/PALB2/RFC5/POLD1/TOP3A/WRN/SLX4/PCNA/POLH/TOPBP1/RFC4/BRCA1/RBBP8/BARD1/POLE2/RFC3/DNA2/POLE/RAD51/BLM/GEN1/RAD51AP1/CHEK1/BRCA2/EME1/BRIP1/EXO1/XRCC2 | 2.37E-07 | REACTOME |
| REACTOME_NUCLEAR_ENVELOPE_BREAKDOWN | -0.448732152 | 0.006841663 | NUP62/NUP133/NUP37/LEMD3/NUP43/RAE1/LEMD2/NUP50/NUP88/NUP98/NUP85/NUP93/PRKCA/NUP153/NEK6/POM121/TPR/AAAS/VRK1/NUP155/NUP188/POM121C/NUP35/NDC1/NUP205/NUP160/NUP42/TMPO/NUP210/NUP107/CCNB1/PLK1/CDK1/CCNB2/LMNB1 | 2.38E-07 | REACTOME |
| REACTOME_HOMOLOGOUS_DNA_PAIRING_AND_STRAND_EXCHANGE | -0.641611646 | 9.86E-06 | RAD1/RPA1/RAD9B/RPA3/ATRIP/ATR/RAD51C/HUS1/RFC2/XRCC3/RMI1/RHNO1/PALB2/RFC5/TOP3A/WRN/TOPBP1/RFC4/BRCA1/RBBP8/BARD1/RFC3/DNA2/RAD51/BLM/RAD51AP1/CHEK1/BRCA2/BRIP1/EXO1/XRCC2 | 2.60E-07 | REACTOME |
| REACTOME_TRANSCRIPTIONAL_REGULATION_BY_E2F6 | -0.628485224 | 0.000181043 | CBX3/MGA/PCGF2/CBX5/TFDP2/SUZ12/E2F6/BRCA1/RBBP8/PHC1/E2F1/RAD51/CDC7/CHEK1/EZH2/RRM2 | 2.65E-07 | REACTOME |
| WP_METAPATHWAY_BIOTRANSFORMATION_PHASE_I_AND_II | 0.53577282 | 0.000167329 | SULT4A1/HS3ST4/NDST3/HS6ST3/CYP4X1/AKR1C2/KCNAB2/CYP46A1/SULT1C2/FMO2/CYP1B1/HS3ST2/KCNAB1/CHST13/HS3ST5/GSTM5/AKR1C1/SULT1A1/CYP39A1/CYP4F3/AKR1C3/AKR7A3/SULT1B1/INMT/CHST1/GSTO2/CYP2E1/CYP2J2/GSTO1/EPHX1/SULT1A4/CYP26B1/NAT8L/NAT2/CYP7B1/CYP2C8/GPX3/MGST3/CYP24A1/CYP27A1/SULT2B1/HS3ST1/FMO4/GSTM3/GPX4 | 2.83E-07 | WP |
| REACTOME_HATS_ACETYLATE_HISTONES | -0.416676964 | 0.000223808 | ING5/HAT1/BRPF3/DMAP1/H3C6/MBIP/ELP2/ACTL6A/BRD1/ELP5/YEATS4/SUPT20H/BRD8/MRGBP/RBBP7/H4C5/KAT6B/H2BC11/CREBBP/BRPF1/ELP1/KANSL1/OGT/SAP130/EP400/EP300/H2BC5/H4C14/TAF5L/YEATS2/HCFC1/WDR5/ING3/H4-16/H2AC8/KAT7/JADE3/H4C9/KANSL2/PAX3/KAT6A/TRRAP/H3C10/H2AC20/H2AC11/H4C11/H2BC6/H3C14/H3C15/H2BC9 | 3.01E-07 | REACTOME |
| WP_NOCGMPPKG_MEDIATED_NEUROPROTECTION | 0.677136008 | 0.000347021 | CAMK2A/GRIN1/NEFL/PDE2A/GRIN2A/TNF/CAMK2B/GRIN2B/GUCY1B1/CAMK2G/CALM1/DLG4/GUCY1A1/GRIN2C/CNGA4/NOS3/NPR1/IL1B/CALM2 | 3.05E-07 | WP |
| KEGG_SPLICEOSOME | -0.395726742 | 0.000152351 | SRSF4/PRPF38B/DDX5/XAB2/SF3A1/WBP11/RBM22/SNRPC/DHX16/CDC5L/PPIE/SF3A3/SRSF2/NCBP2/SNRNP40/EIF4A3/HNRNPK/SRSF3/HNRNPM/SF3B6/SNRPA1/DHX38/MAGOH/HNRNPA1L2/PRPF6/DDX39B/SF3B2/SNRPE/NCBP1/SNRPB2/CRNKL1/HNRNPA1/SRSF9/SART1/DHX8/USP39/SNRPF/SRSF10/SRSF6/HNRNPA3/SNRPA/SNRPD1/TRA2A/SF3B1/TRA2B/SNRPG/SF3B3/PPIH/DDX42/DDX46/SF3B4/RBM8A/EFTUD2/SRSF1/AQR/TCERG1/U2AF2/THOC1/U2SURP/LSM5/PRPF38A/LSM7/MAGOHB/HNRNPU/DHX15/ACIN1/SNRNP70/PRPF8/PRPF40A/CHERP/U2AF1/THOC2/RBMX/PRPF3/PRPF4/DDX23/LSM8/SF3A2/SNRPB/ALYREF/SNRNP200 | 3.34E-07 | KEGG |
| REACTOME_FANCONI_ANEMIA_PATHWAY | -0.582730196 | 0.000410662 | POLN/CENPX/MUS81/RPA1/RPA3/ATRIP/ATR/DCLRE1A/FAAP100/FAAP20/ERCC4/FANCG/DCLRE1B/SLX4/USP1/FAAP24/FANCE/FANCM/FANCA/FANCC/FANCD2/UBE2T/FANCB/EME1/FANCI | 3.44E-07 | REACTOME |
| KEGG_HOMOLOGOUS_RECOMBINATION | -0.603324324 | 0.002776311 | RAD51D/MRE11/NBN/SSBP1/MUS81/RPA1/RPA3/RAD51C/TOP3B/XRCC3/POLD3/RAD52/POLD1/TOP3A/RAD54B/RAD51/BLM/RAD54L/BRCA2/EME1/XRCC2 | 3.55E-07 | KEGG |
| REACTOME_MET_RECEPTOR_ACTIVATION | 0.836480873 | 0.035780552 | HPN/SPINT2 | 3.62E-07 | REACTOME |
| REACTOME_REGULATION_OF_TP53_ACTIVITY_THROUGH_PHOSPHORYLATION | -0.409272296 | 0.001227616 | RFC2/TAF5/CSNK2A1/TAF2/DYRK2/RMI1/SSRP1/RHNO1/PRKAA2/RFC5/TAF4/TOP3A/SUPT16H/WRN/TP53/TAF1/TOPBP1/RFC4/BRCA1/RBBP8/BARD1/AURKA/RFC3/DNA2/BLM/CDK2/MDM4/CHEK1/AURKB/CCNA2/TPX2/BRIP1/EXO1 | 3.68E-07 | REACTOME |
| WP_HISTONE_MODIFICATIONS | -0.512438038 | 0.000981429 | SETD4/H3C6/KMT5A/SET/H4C5/KMT5B/KMT5C/SETD1B/SETD6/KMT2E/SETDB1/EHMT1/AEBP2/KMT2B/SETD2/NSD1/SETD1A/KMT2C/H4-16/KMT2D/KMT2A/H4C9/SUV39H1/H3C10/SETBP1/SETD5/DOT1L/SUV39H2/H4C11/H3C14/H3C15/EZH2 | 4.01E-07 | WP |
| REACTOME_TP53_REGULATES_TRANSCRIPTION_OF_DNA_REPAIR_GENES | -0.404253083 | 0.011862221 | GTF2F1/TCEA1/ATM/JUN/PMS2/NELFB/GTF2H3/RAD51D/NELFCD/ERCC3/CCNK/SUPT5H/CCNT2/POLR2H/MNAT1/MSH2/NELFA/POLR2A/ATR/POLR2J/CDK13/ELOA/GTF2H4/SSRP1/POLR2B/GTF2H2/CDK12/CCNT1/POLR2D/MDC1/SUPT16H/TP53/BRCA1/FANCC/FANCD2/CHEK1/FANCI | 4.21E-07 | REACTOME |
| REACTOME_POTASSIUM_CHANNELS | 0.69490663 | 7.11E-09 | HCN1/KCNC2/KCNJ12/KCNJ3/KCNH5/KCNA4/GNG3/KCNS1/KCNJ6/KCNJ4/KCNH3/KCNK4/KCNN1/KCNAB2/KCNA1/KCNK1/ABCC8/KCNQ5/KCNAB1/KCNG1/HCN2/KCNJ11/KCNJ9/GABBR1/KCNJ5/KCNH1/KCNB1/KCNQ3/KCNMB4/KCNC1/KCNK9/KCNG2/KCNC3/KCNMA1/KCNJ15/KCNC4/GNGT2/GABBR2/KCNA5/KCNQ1/KCNH8/KCNQ4/KCNQ2/KCNK6/HCN4/GNB5/KCNK13/KCNMB2 | 4.55E-07 | REACTOME |
| REACTOME_DNA_DOUBLE_STRAND_BREAK_REPAIR | -0.51373062 | 7.11E-09 | XRCC5/XRCC1/MUS81/RAD1/RFC1/RPA1/POLE3/RAD9B/RPA3/ABL1/TP53BP1/PARP1/ATRIP/ATR/RTEL1/EYA4/H4C5/TDP1/RAD51C/H2BC11/UIMC1/EYA2/SMARCA5/HUS1/RFC2/H2BC5/LIG3/ERCC4/H4C14/XRCC3/RIF1/RMI1/POLD3/RHNO1/RAD52/H4-16/PALB2/H2AX/MDC1/KDM4A/H4C9/RFC5/POLD1/BAZ1B/TOP3A/WRN/TP53/SLX4/PRKDC/PCNA/FEN1/POLH/KPNA2/KDM4B/TOPBP1/PAXIP1/RFC4/TIPIN/BRCA1/RBBP8/NSD2/BARD1/POLE2/H4C11/RFC3/TIMELESS/DNA2/POLE/RAD51/H2BC6/BLM/GEN1/CDK2/RAD51AP1/CHEK1/H2BC9/BRCA2/EYA1/EME1/CCNA2/CLSPN/POLQ/BRIP1/EXO1/XRCC2 | 5.10E-07 | REACTOME |
| KEGG_OLFACTORY_TRANSDUCTION | 0.690708942 | 0.005729801 | CAMK2A/CLCA4/CAMK2B/CAMK2G/GNAL/CALM1/CALM3/GRK3/PRKACB/CNGA4/OR7C1/ARRB2/PRKG1/PDE1C/CALM2/OR51E1/OR2H2 | 5.12E-07 | KEGG |
| REACTOME_DISEASES_OF_DNA_REPAIR | -0.691444427 | 4.16E-06 | PALB2/TOP3A/WRN/MSH6/BRCA1/RBBP8/BARD1/DNA2/RAD51/BLM/RAD51AP1/NEIL3/BRCA2/BRIP1/EXO1/XRCC2 | 5.25E-07 | REACTOME |
| REACTOME_MRNA_SPLICING | -0.402930133 | 3.14E-07 | CSTF3/GTF2F1/SNRNP27/CCAR1/SRSF4/GCFC2/SYMPK/DDX5/XAB2/CLP1/SF3A1/WBP11/RBM22/MTREX/SNRPC/DHX16/CWC27/SRRM2/CDC5L/PPIE/SF3A3/SRSF2/SF1/NCBP2/PDCD7/TFIP11/SNRNP40/EIF4A3/HNRNPK/CSTF1/RNPS1/SRSF3/HNRNPM/SF3B6/POLR2H/ZCRB1/SNRPA1/DHX38/MAGOH/PRPF6/SF3B2/SNRPE/HNRNPL/NCBP1/CPSF1/SNRPB2/CRNKL1/POLR2A/HNRNPA1/SRSF9/SNRNP48/SART1/USP39/SNRPF/SRSF10/PPIL3/SRSF6/HNRNPA3/POLR2J/SNRPA/SNRPD1/SF3B1/SRSF11/CWC22/NUDT21/PCBP2/TRA2B/CPSF7/SNRPG/SF3B3/PPIH/DDX42/DDX46/SF3B4/RBM8A/CPSF4/WDR33/EFTUD2/SRSF1/YBX1/PCF11/AQR/ELAVL1/SRRT/U2AF2/HNRNPD/U2SURP/LSM5/PRPF38A/LSM7/MAGOHB/HNRNPA2B1/HNRNPU/DHX15/CPSF3/DHX9/SNRNP70/HNRNPR/POLR2B/PTBP1/PRPF8/UPF3B/PRPF40A/CHERP/U2AF1/FUS/POLR2D/CSTF2/RBMX/PRPF3/PRPF4/HNRNPH1/HNRNPUL1/DDX23/HNRNPA0/LSM8/SF3A2/SNRPB/ALYREF/FIP1L1/SNRNP200 | 5.31E-07 | REACTOME |
| REACTOME_POSTMITOTIC_NUCLEAR_PORE_COMPLEX_NPC_REFORMATION | -0.563174399 | 0.008586789 | UBE2I/NUP54/NUP58/NUP62/NUP133/NUP37/NUP43/NUP98/NUP85/NUP93/KPNB1/AHCTF1/POM121/TNPO1/NUP155/NUP188/NUP35/NDC1/NUP205/NUP160/RCC1/NUP107 | 5.37E-07 | REACTOME |
| REACTOME_SUMOYLATION_OF_DNA_REPLICATION_PROTEINS | -0.595686334 | 4.46E-05 | NUP58/NUP62/PIAS4/NUP133/NUP37/NUP43/RAE1/NUP50/NUP88/NUP98/TOP2B/NUP85/NUP93/NUP153/POM121/TOP1/TPR/AAAS/NUP155/NUP188/POM121C/PCNA/NUP35/NDC1/NUP205/NUP160/INCENP/NUP42/NUP210/AURKA/NUP107/CDCA8/AURKB/BIRC5/TOP2A | 5.45E-07 | REACTOME |
| REACTOME_HOMOLOGY_DIRECTED_REPAIR | -0.546229691 | 7.11E-09 | RFC2/H2BC5/LIG3/ERCC4/H4C14/XRCC3/RMI1/POLD3/RHNO1/RAD52/H4-16/PALB2/H2AX/MDC1/H4C9/RFC5/POLD1/TOP3A/WRN/SLX4/PCNA/FEN1/POLH/TOPBP1/RFC4/TIPIN/BRCA1/RBBP8/NSD2/BARD1/POLE2/H4C11/RFC3/TIMELESS/DNA2/POLE/RAD51/H2BC6/BLM/GEN1/CDK2/RAD51AP1/CHEK1/H2BC9/BRCA2/EME1/CCNA2/CLSPN/POLQ/BRIP1/EXO1/XRCC2 | 5.56E-07 | REACTOME |
| PID_ATM_PATHWAY | -0.531803145 | 0.007686644 | TRIM28/SMC3/H2AX/MDC1/TOP3A/SMC1A/BRCA1/RBBP8/BLM/FANCD2/CDC25C/CDC25A | 5.69E-07 | PID |
| WP_DNA_IRDOUBLE_STRAND_BREAKS_AND_CELLULAR_RESPONSE_VIA_ATM | -0.536805658 | 0.000108128 | ABL1/ACTL6A/TP53BP1/PARP1/TRAF6/ATR/MCPH1/TRIM28/RIF1/SMC3/RAD52/H2AX/MDC1/TP53/PRKDC/PCNA/SMC1A/BRCA1/E2F1/RAD51/BLM/TP73/FANCD2/CHEK1/BRCA2/CDC25C/EXO1 | 5.99E-07 | WP |
| REACTOME_SENSORY_PERCEPTION | 0.495351184 | 0.000482829 | GRM1/RBP4/SNAP25/CABP1/SYN1/RAB3A/SCN2B/USH1C/SYP/GPIHBP1/STX1A/LRP2/SCN2A/ATP2B2/PCLO/CLIC5/RIPOR2/STRA6/BSN/SCN1B/AKR1C1/EPB41L3/DHRS9/OTOF/STRC/GNAL/MYO15A/CALM1/HSD17B6/PRKCQ/EPB41L1/VAMP2/AKR1C3/MPP1/KCNMA1/ABCA4/GPC3/CACNB2/PPEF1/CNGA4/CACNA2D2/OR7C1/GPC5/ANO2/RDH12/CACNA1D/GSN/PDE6B/MYO7A/SCN4B/KCNQ4/PLCB2/ATP2B1/GNB5/SDC2 | 6.74E-07 | REACTOME |
| KEGG_ARACHIDONIC_ACID_METABOLISM | 0.592973042 | 0.023001228 | PTGDS/CYP4F3/LTC4S/AKR1C3/CYP2E1/CYP2J2/HPGDS/PTGIS/PTGES/CYP2C8/TBXAS1/GPX3/PLA2G2A/GPX1/CBR1/GPX4/PTGS2/ALOX5 | 6.86E-07 | KEGG |
| PID_BARD1_PATHWAY | -0.67426047 | 7.59E-05 | FANCG/TP53/PRKDC/PCNA/FANCE/TOPBP1/CCNE1/BRCA1/RBBP8/BARD1/FANCA/FANCC/RAD51/FANCD2/CDK2 | 7.19E-07 | PID |
| REACTOME_TRNA_MODIFICATION_IN_THE_NUCLEUS_AND_CYTOSOL | -0.476272481 | 0.01208385 | TRMT61A/NSUN2/TRMT10A/PUS1/TRMT12/QTRT1/THG1L/THADA/WDR4/TP53RK/TPRKB/ADAT1/EPRS1/TYW1/CDKAL1/TRMT5/ALKBH8/LCMT2/TYW5/TRMT6/QTRT2/TRMT13/PUS3/PUS7/METTL1 | 7.44E-07 | REACTOME |
| PID_FANCONI_PATHWAY | -0.644193951 | 2.38E-06 | FAAP100/HUS1/RFC2/XRCC3/RMI1/FANCG/PALB2/H2AX/RFC5/TOP3A/USP1/FAAP24/FANCE/TOPBP1/FANCM/RFC4/BRCA1/FANCA/FANCC/RFC3/BLM/FANCD2/UBE2T/FANCB/CHEK1/BRCA2/FANCI/BRIP1 | 7.66E-07 | PID |
| WP_BENZENE_METABOLISM | 0.877637346 | 0.036897421 | DHDH/MPO/CYP2E1/EPHX1/NQO1 | 7.81E-07 | WP |
| REACTOME_MITOTIC_PROPHASE | -0.433394943 | 4.24E-05 | NUP133/NUP37/LEMD3/NUP43/RAE1/LEMD2/H3C6/NUP50/KMT5A/NUP88/NUP98/SET/H4C5/H2BC11/H2AZ1/H2AZ2/NUP85/NUP93/MCPH1/NUMA1/PRKCA/H2BC5/H4C14/NUP153/NEK6/PHF8/POM121/MASTL/H4-16/TPR/AAAS/H2AX/H2AC8/H4C9/VRK1/NUP155/NUP188/POM121C/NUP35/NDC1/NCAPD3/NUP205/H3C10/NUP160/NUP42/H2AC20/TMPO/SMC2/NUP210/NUP107/H4C11/H2BC6/NCAPG2/H3C14/H3C15/CCNB1/H2BC9/PLK1/CDK1/SMC4/CCNB2/LMNB1 | 7.85E-07 | REACTOME |
| REACTOME_PHASE_4_RESTING_MEMBRANE_POTENTIAL | 0.689428531 | 0.034192793 | KCNJ12/KCNJ4/KCNK4/KCNK1/KCNK12/KCNK9/KCNK6/KCNK13/KCNK3/KCNK5/KCNJ2 | 8.09E-07 | REACTOME |
| REACTOME_PEPTIDE_LIGAND_BINDING_RECEPTORS | 0.67312358 | 7.11E-09 | CCK/PDYN/RXFP1/SST/CCL19/CCL3/CCL3L3/CORT/CXCL11/NTSR2/CCL4/CXCL12/EDN3/CXCR1/XK/NPFFR1/PPBP/SSTR1/TAC1/NPY/SSTR2/CXCR2/CCL5/KEL/CXCL9/CXCL10/CXCL1/CXCR3/NPY1R/CXCR6/FPR2/PROK2/C5AR1/C5AR2/GPR37/FPR3/QRFPR/CCR6/PNOC/CXCL6/CCL28/CCR1/CCR4/POMC/CXCL3/CCRL2/CCR2/PENK/F2RL2/TACR2/CCR7/TAC3/EDN1/GHRL/C3AR1/CXCL2/CXCL5/NPB/PSAP/FPR1/OPRL1/CCR5/OPRD1/APLNR/ECE2/F2RL3/CXCL8/CX3CR1/HEBP1 | 8.35E-07 | REACTOME |
| REACTOME_GENE_SILENCING_BY_RNA | -0.384196517 | 0.00154937 | NUP62/NUP133/NUP37/POLR2H/NUP43/RAE1/H3C6/NUP50/MYBL1/NUP88/POLR2A/NUP98/TSN/H4C5/TNRC6B/H2BC11/POLR2J/DROSHA/H2AZ1/H2AZ2/NUP85/NUP93/ANG/BCDIN3D/H2BC5/TARBP2/H4C14/IPO8/NUP153/AGO3/DGCR8/POLR2B/TNRC6A/POM121/H4-16/POLR2D/TPR/AAAS/TNRC6C/H2AX/H2AC8/H4C9/NUP155/NUP188/AGO2/POM121C/NUP35/NDC1/NUP205/H3C10/NUP160/NUP42/H2AC20/XPO5/NUP210/NUP107/H4C11/H2BC6/H3C14/H3C15/H2BC9 | 9.87E-07 | REACTOME |
| REACTOME_RESOLUTION_OF_D_LOOP_STRUCTURES_THROUGH_SYNTHESIS_DEPENDENT_STRAND_ANNEALING_SDSA | -0.69929731 | 8.25E-05 | XRCC3/RMI1/PALB2/TOP3A/WRN/BRCA1/RBBP8/BARD1/DNA2/RAD51/BLM/RAD51AP1/BRCA2/BRIP1/EXO1/XRCC2 | 9.90E-07 | REACTOME |
| PID_ATR_PATHWAY | -0.681395257 | 1.31E-06 | TOPBP1/RFC4/TIPIN/MCM7/RFC3/TIMELESS/RAD51/FANCD2/CDK2/MCM2/CDC6/CHEK1/BRCA2/PLK1/CDC25C/CCNA2/CLSPN/CDC25A | 1.07E-06 | PID |
| REACTOME_RESOLUTION_OF_D_LOOP_STRUCTURES | -0.698703987 | 2.35E-06 | PALB2/TOP3A/WRN/SLX4/BRCA1/RBBP8/BARD1/DNA2/RAD51/BLM/GEN1/RAD51AP1/BRCA2/EME1/BRIP1/EXO1/XRCC2 | 1.12E-06 | REACTOME |
| REACTOME_RRNA_MODIFICATION_IN_THE_NUCLEUS_AND_CYTOSOL | -0.442215943 | 0.005909829 | FBL/UTP4/UTP25/FCF1/DHX37/NAT10/PNO1/UTP3/DDX47/EMG1/UTP14A/NOP14/WDR46/WDR75/WDR36/KRR1/PDCD11/NOP56/DCAF13/DKC1/DDX52/NOP58/NOL11/UTP20/GAR1/UTP15/WDR3/WDR43/NOP2/HEATR1 | 1.19E-06 | REACTOME |
| REACTOME_PLATELET_HOMEOSTASIS | 0.600680414 | 6.16E-05 | SLC8A2/ATP2B3/PDE2A/GNG3/PDE1A/PDE1B/P2RX5/ATP2B2/PDE11A/GUCY1B1/ITPR1/KCNMB4/CALM1/GUCY1A1/IRAG1/KCNMA1/GNGT2/ATP2A3/NOS3/P2RX1/PTPN6/PRKG1/ATP2B1/PDE5A/GNB5/KCNMB2/PECAM1/P2RX4/GNAS/GNG11/FGR/PPP2R5A/SLC8A1/P2RX6/PTGIR/STIM1/PDE10A/GNG7 | 1.21E-06 | REACTOME |
| REACTOME_MUSCLE_CONTRACTION | 0.602337998 | 7.11E-09 | CAMK2A/SLC8A2/CASQ2/ATP2B3/RYR2/MYH11/KCNJ12/KCNJ4/SCN2B/FGF13/SCN3B/KCNK4/PLN/KCNK1/KCNK12/CAMK2B/FXYD7/ATP1A3/SCN2A/ATP1B1/ATP2B2/KCNIP2/KCNJ11/CACNG8/KCNIP4/PAK1/SCN1B/MYBPC1/MYL3/GUCY1B1/ITPR1/CAMK2G/CALM1/GUCY1A1/TNNT1/KCNK9/DYSF/SCN8A/NPPC/KCNE1/ACTG2/LMOD1/CLIC2/FXYD1/CACNB2/CACNA2D2/MYL4/FGF14/CACNB1/FKBP1B/ATP2A3/KCNQ1/KCNE5/ATP1A2/TPM1/RYR1/TTN/SCN4B/FGF12/FXYD2/CASQ1/ATP2B1/NPR1/MYL9/KCNK6/ACTA2/SLN/KCNK13/MYLK/KCNE3/TMOD2/DMD/SCN7A/CACNA1C | 1.21E-06 | REACTOME |
| REACTOME_PROCESSING_OF_DNA_DOUBLE_STRAND_BREAK_ENDS | -0.51599441 | 6.00E-06 | RHNO1/H4-16/H2AX/MDC1/H4C9/RFC5/TOP3A/WRN/TOPBP1/RFC4/TIPIN/BRCA1/RBBP8/NSD2/BARD1/H4C11/RFC3/TIMELESS/DNA2/H2BC6/BLM/CDK2/CHEK1/H2BC9/CCNA2/CLSPN/BRIP1/EXO1 | 1.25E-06 | REACTOME |
| REACTOME_REGULATION_OF_TP53_ACTIVITY | -0.313803521 | 0.009437054 | RAD1/BRPF3/RPA1/TP53INP1/RAD9B/RPA3/GATAD2A/KMT5A/BRD1/TAF15/ATRIP/TP53RK/ATR/RBBP7/MAPKAP1/CSNK2A2/TAF7/BRPF1/DAXX/MTA2/NOC2L/RBBP4/HUS1/HDAC2/CHD4/RFC2/EHMT1/EP300/TAF5/GATAD2B/CSNK2A1/TAF2/DYRK2/PRMT5/RMI1/SSRP1/RHNO1/PRKAA2/RFC5/TAF4/TOP3A/SUPT16H/WRN/KAT6A/TP53/TAF1/TOPBP1/RFC4/BRCA1/RBBP8/BARD1/AURKA/RFC3/DNA2/BLM/TP73/CDK2/MDM4/CHEK1/CDK1/AURKB/CCNA2/TPX2/BRIP1/EXO1/POU4F1 | 1.32E-06 | REACTOME |
| REACTOME_SENSORY_PROCESSING_OF_SOUND | 0.58290368 | 0.001311999 | SNAP25/CABP1/SYN1/RAB3A/USH1C/SYP/STX1A/ATP2B2/PCLO/CLIC5/RIPOR2/BSN/EPB41L3/OTOF/STRC/MYO15A/EPB41L1/VAMP2/MPP1/KCNMA1/CACNB2/CACNA2D2/CACNA1D/GSN/MYO7A/KCNQ4/ATP2B1 | 1.37E-06 | REACTOME |
| REACTOME_CONSTITUTIVE_SIGNALING_BY_ABERRANT_PI3K_IN_CANCER | 0.510301501 | 0.036914895 | FGF7/NRG3/KL/CD28/LCK/FGF17/FGF22/FGFR2/FLT3/FGF9/FGF18/CD80/PIK3CD/TRAT1/PIK3AP1/FGF20/FGF1/VAV1/RAC2/PDGFRB/NRG4/PDGFA/ESR1/PDGFB/RHOG/CD86/TGFA/EREG/PIK3CB | 1.42E-06 | REACTOME |
| REACTOME_PKMTS_METHYLATE_HISTONE_LYSINES | -0.508934963 | 0.000443301 | RBBP7/H4C5/KMT5B/KMT5C/SETD1B/SETD6/RBBP4/KMT2E/SETDB1/RELA/EHMT1/AEBP2/KMT2B/H4C14/SETD2/WDR5/NSD1/SETD1A/KMT2C/H4-16/KMT2D/KMT2A/H4C9/ATF7IP/SUZ12/SUV39H1/H3C10/NSD2/DOT1L/SUV39H2/H4C11/H3C14/H3C15/EZH2 | 1.49E-06 | REACTOME |
| WP_SMALL_LIGAND_GPCRS | 0.728866298 | 0.021158258 | PTGER2/PTGFR/S1PR4/LPAR1/PTGER3/S1PR1/PTAFR/PTGER1/PTGER4/S1PR3/PTGIR | 1.62E-06 | WP |
| BIOCARTA_EICOSANOID_PATHWAY | 0.720728256 | 0.003274336 | PTGER2/PTGFR/CYP2J2/PTGER3/EPHX1/HPGDS/PTGIS/PTGES/PTGER1/CYSLTR1/TBXAS1/PLCB1/PTGER4/CYSLTR2/PTGS2/ALOX5/PTGIR | 1.66E-06 | BIOCARTA |
| WP_NEUROINFLAMMATION_AND_GLUTAMATERGIC_SIGNALING | 0.598376831 | 1.71E-07 | PRKCG/CAMK2A/GRIN1/SLC17A7/GRM1/GLS2/SLC7A10/GRIN3A/GRM2/IL10/CAMKK1/PRKCB/GRM7/GRIN2A/IGF1/TNF/CAMK4/IL6/CAMK2B/GRIN2B/GOT1/SLC1A6/CAMK2G/ADCY1/CALM1/SLC1A2/CAMKK2/IL10RA/TGFBR3/STAT6/GLS/MAPK3/GRIN2C/IL1R1/GLUL/PLCB4/NSMF/CNTF/TGFBR2/GRM5/GRM8/SLC38A3/PLCB2/LRRC8C/IL4R/PLCB1/IL1B/FOS/CALM2/LRRC8B/TRPM4/SMAD7 | 2.06E-06 | WP |
| WP_PURINERGIC_SIGNALING | 0.625604594 | 0.024638159 | P2RX5/P2RY10/P2RY12/P2RY14/GNAI1/ADORA3/P2RY8/GNAO1/P2RX1/P2RY13/P2RY2/P2RX4/GNAZ/GNAS | 2.36E-06 | WP |
| WP_ALTERNATIVE_PATHWAY_OF_FETAL_ANDROGEN_SYNTHESIS | 0.853535758 | 0.011104988 | AKR1C2/STAR/HSD17B3/HSD17B6 | 2.40E-06 | WP |
| WP_SLEEP_REGULATION | 0.66677404 | 0.009119487 | DRD1/HTR2A/CACNA1I/STAR/PTGDS/GRIN2A/IL6/CST3/GHRL/FOS/IL18/BTBD9/CHRNB2/PER3 | 2.41E-06 | WP |
| REACTOME_G2_M_DNA_DAMAGE_CHECKPOINT | -0.48031941 | 3.41E-05 | RHNO1/H4-16/H2AX/MDC1/H4C9/RFC5/TOP3A/WRN/TP53/TOPBP1/RFC4/BRCA1/RBBP8/NSD2/BARD1/H4C11/RFC3/DNA2/H2BC6/BLM/WEE1/CHEK1/CCNB1/H2BC9/CDC25C/CDK1/BRIP1/EXO1 | 2.45E-06 | REACTOME |
| REACTOME_GABA_RECEPTOR_ACTIVATION | 0.730671417 | 1.84E-07 | GABRA5/GABRA1/GABRB2/GABRA4/KCNJ12/KCNJ3/GABRG2/GNG3/KCNJ6/KCNJ4/GABRA2/GABRG3/GABRB3/KCNJ9/GABBR1/KCNJ5/GABRB1/GNAL/ADCY1/ADCY4/ADCY5/KCNJ15/GNAI1/ADCY2/GNGT2/NPTN/GABBR2 | 2.47E-06 | REACTOME |
| REACTOME_NONHOMOLOGOUS_END_JOINING_NHEJ | -0.394926572 | 0.038937548 | H4C15/RAD50/ATM/H2BC8/RNF168/KAT5/HERC2/MRE11/BRCC3/NBN/PIAS4/XRCC5/TP53BP1/H4C5/TDP1/H2BC11/UIMC1/H2BC5/H4C14/RIF1/H4-16/H2AX/MDC1/H4C9/PRKDC/PAXIP1/BRCA1/NSD2/BARD1/H4C11/H2BC6/H2BC9 | 2.52E-06 | REACTOME |
| WP_VITAMIN_A_AND_CAROTENOID_METABOLISM | 0.627305364 | 0.011186795 | RBP4/CD36/RXRG/CRABP1/RBP7/ALDH1A2/ALDH1A1/CRABP2/SULT1A1/CYP2E1/CYP26B1/RDH12/ALDH1A3/SULT2B1/DHRS3 | 2.93E-06 | WP |
| REACTOME_PI3K_AKT_SIGNALING_IN_CANCER | 0.489793014 | 0.016239884 | FGF7/NRG3/NR4A1/KL/CD28/LCK/FGF17/FGF22/FGFR2/FLT3/FGF9/FGF18/PRR5/CD80/FOXO4/PIK3CD/TRAT1/PIK3AP1/FGF20/FGF1/VAV1/RAC2/PDGFRB/NRG4/PDGFA/ESR1/PDGFB/RHOG/CD86/TGFA/PTEN/EREG/PIK3CB | 2.94E-06 | REACTOME |
| WP_SMC1SMC3_ROLE_IN_DNA_DAMAGE_CORNELIA_DE_LANGE_SYNDROME | -0.671149572 | 0.040646772 | RAD50/ATM/MRE11/NBN/SMC3/MDC1/SMC1A/PAXIP1/BRCA1/RAD18 | 2.95E-06 | WP |
| WP_NUCLEOTIDE_EXCISION_REPAIR | -0.446521385 | 0.018670219 | RFC1/MNAT1/RPA1/POLE3/RPA3/CUL4B/RAD23B/RFC2/ERCC4/GTF2H4/GTF2H2C/POLD3/GTF2H2/RFC5/POLD1/PCNA/LIG1/RFC4/POLE2/RFC3/POLE | 3.00E-06 | WP |
| WP_MBDNF_AND_PROBDNF_REGULATION_OF_GABA_NEUROTRANSMISSION | 0.693343574 | 0.000217358 | GABRA5/GABRA1/SLC12A5/GABRB2/GABRG1/GABRA4/GABRG2/GABRD/GABRA2/GABRG3/GABRB3/GABRB1 | 3.09E-06 | WP |
| REACTOME_TRANSCRIPTIONAL_REGULATION_BY_SMALL_RNAS | -0.535347765 | 1.96E-06 | NUP62/NUP133/NUP37/POLR2H/NUP43/RAE1/H3C6/NUP50/NUP88/POLR2A/NUP98/H4C5/H2BC11/POLR2J/H2AZ1/H2AZ2/NUP85/NUP93/H2BC5/H4C14/IPO8/NUP153/POLR2B/TNRC6A/POM121/H4-16/POLR2D/TPR/AAAS/H2AX/H2AC8/H4C9/NUP155/NUP188/AGO2/POM121C/NUP35/NDC1/NUP205/H3C10/NUP160/NUP42/H2AC20/NUP210/NUP107/H4C11/H2BC6/H3C14/H3C15/H2BC9 | 3.20E-06 | REACTOME |
| REACTOME_SIGNALING_BY_ERBB4 | 0.552960587 | 0.011953666 | GABRA1/GABRB2/GABRG2/PGR/ADAP1/NRG3/GABRG3/CXCL12/GABRB3/GABRB1/DLG4/S100B | 3.46E-06 | REACTOME |
| REACTOME_FORMATION_OF_SENESCENCE_ASSOCIATED_HETEROCHROMATIN_FOCI_SAHF | -0.665889352 | 0.023473549 | CABIN1/EP400/H1-0/HMGA1/TP53/LMNB1/HMGA2 | 3.50E-06 | REACTOME |
| REACTOME_RESOLUTION_OF_ABASIC_SITES_AP_SITES | -0.541599567 | 0.000981429 | XRCC1/RFC1/RPA1/POLE3/RPA3/PARP1/MUTYH/TDG/RFC2/APEX1/LIG3/POLD3/UNG/RFC5/POLD1/PCNA/FEN1/LIG1/RFC4/POLE2/RFC3/POLE | 3.58E-06 | REACTOME |
| KEGG_METABOLISM_OF_XENOBIOTICS_BY_CYTOCHROME_P450 | 0.655310372 | 0.007338774 | AKR1C2/CYP1B1/DHDH/GSTM5/AKR1C1/AKR1C3/GSTO2/CYP2E1/GSTO1/EPHX1/CYP2C8/ALDH1A3/MGST3/GSTM3/CYP2S1/MGST2/ALDH3B1 | 3.95E-06 | KEGG |
| REACTOME_G_ALPHA_Q_SIGNALLING_EVENTS | 0.545210064 | 7.16E-06 | CCK/GRM1/HTR2A/CHRM1/GPR143/ADRA1B/GNG3/NTSR2/FFAR3/EDN3/PTGFR/RGS4/NPFFR1/TAC1/P2RY10/CHRM5/DGKE/FPR2/GNA14/ITPR1/PROK2/LPAR1/PRKCQ/QRFPR/DGKB/PRKCE/ADRA1A/DGKZ/RGS5/RGS2/HTR2B/CHRM3/RGS18/KALRN/MAPK3/GNGT2/GPR132/F2RL2/PRKCD/TACR2/PLCB4/RGS1/FFAR2/RPS6KA2/TAC3/LPAR5/PRKCH/EDN1/PTAFR/GHRL/GRM5/BTK/PTGER1/CYSLTR1/GPR68/PLCB2/GNA15/FFAR4/PLCB1/GNB5/CYSLTR2/F2RL3/GRK2/P2RY2/ABHD6/GNG11/RPS6KA1/GPR65/AGTR1/RASGRP2/DGKG/DGKQ/GRK5/SAA1 | 4.12E-06 | REACTOME |
| KEGG_NUCLEOTIDE_EXCISION_REPAIR | -0.438488164 | 0.031381089 | GTF2H3/ERCC3/ERCC8/RFC1/MNAT1/RPA1/POLE3/RPA3/CUL4B/RAD23B/RFC2/ERCC4/GTF2H4/POLD3/GTF2H2/RFC5/POLD1/PCNA/LIG1/RFC4/POLE2/RFC3/POLE | 4.14E-06 | KEGG |
| REACTOME_MRNA_SPLICING_MINOR_PATHWAY | -0.409477143 | 0.026524415 | SRSF2/NCBP2/PDCD7/SNRNP40/SF3B6/POLR2H/ZCRB1/PRPF6/SF3B2/SNRPE/NCBP1/POLR2A/SNRNP48/SNRPF/SRSF6/POLR2J/SNRPD1/SF3B1/SNRPG/SF3B3/DDX42/SF3B4/EFTUD2/SRSF1/YBX1/POLR2B/PRPF8/POLR2D/DDX23/SNRPB/SNRNP200 | 4.33E-06 | REACTOME |
| REACTOME_MITOTIC_PROMETAPHASE | -0.484962212 | 7.11E-09 | SFI1/DSN1/HAUS6/CSNK1E/ITGB3BP/CEP290/CSNK2A1/CEP76/STAG2/TUBA1A/AHCTF1/MIS12/MAPRE1/CENPQ/SKA2/TUBB/CDK5RAP2/SMC3/NEK6/CEP131/CEP164/CEP192/CEP57/CENPP/CLASP1/SPDL1/HAUS3/CEP72/STAG1/ODF2/NEDD1/RCC2/TUBGCP4/DCTN2/CENPL/HAUS5/TUBB2B/CEP250/NUP160/INCENP/SMC1A/HAUS8/ALMS1/ZWILCH/CENPN/CENPJ/OFD1/SMC2/NCAPD2/CENPU/CENPO/CENPH/NUP107/CEP152/ZWINT/CENPM/KNTC1/CEP135/CDCA5/SPC24/CCNB1/CENPK/PLK1/NDC80/MAD2L1/CDC20/PLK4/CDK1/NCAPG/CDCA8/AURKB/BIRC5/KIF2C/SGO2/SPC25/NUF2/SKA1/CENPE/ERCC6L/SMC4/KIF18A/BUB1B/CCNB2/NEK2/CENPA/SGO1/CENPI/BUB1/NCAPH/CENPF/KNL1 | 4.49E-06 | REACTOME |
| WP_PEPTIDE_GPCRS | 0.631773041 | 0.003157983 | NTSR2/CXCR1/SSTR1/ATP8A1/SSTR2/CXCR2/CXCR3/NPY1R/CXCR6/FPR2/C5AR1/FPR3/CCR6/CCR1/CCR4/CCR2/TACR2/CCR7/C3AR1/FPR1/OPRL1/CCR5/OPRD1/CX3CR1/AGTR1 | 4.74E-06 | WP |
| REACTOME_HIV_LIFE_CYCLE | -0.276756211 | 0.047187407 | NELFB/GTF2H3/NELFCD/ERCC3/NUP54/CCNK/TAF9B/TBP/NCBP2/NUP58/SUPT5H/CCNT2/NUP62/XRCC5/NUP133/NUP37/POLR2H/NUP43/MNAT1/RAE1/NUP50/NELFA/NUP88/NCBP1/POLR2A/TAF15/XPO1/NUP98/POLR2J/TAF7/NUP85/RNMT/NUP93/ELOA/TAF5/GTF2H4/NUP153/TAF2/SSRP1/RANBP1/POLR2B/GTF2H2/POM121/VPS37D/CCNT1/POLR2D/TPR/AAAS/TAF4/VPS37B/SUPT16H/HMGA1/NUP155/NUP188/POM121C/TAF1/NUP35/NDC1/FEN1/NUP205/NUP160/RCC1/LIG1/NUP42/NUP210/NUP107 | 4.78E-06 | REACTOME |
| WP_PHOSPHODIESTERASES_IN_NEURONAL_FUNCTION | 0.623026128 | 0.001750979 | DRD1/GRIN1/PDE2A/PDE1A/PDE1B/PPP1R1B/GRIN2A/GRIN2B/PDE11A/GUCY1B1/ADCY1/ADCY4/GUCY1A1/ADCY5/ADCY2/GRIN2C/PDE6B/CHRNA7/PDE1C/PDE5A/CHRFAM7A/PDE4A | 4.95E-06 | WP |
| REACTOME_ANCHORING_OF_THE_BASAL_BODY_TO_THE_PLASMA_MEMBRANE | -0.374131198 | 0.009029842 | SFI1/HAUS6/CSNK1E/CEP290/TCTN1/CEP76/TUBA1A/MAPRE1/TUBB/CDK5RAP2/C2CD3/CEP131/B9D1/CEP164/CEP192/CEP57/TCTN2/MKS1/CLASP1/HAUS3/CEP72/ODF2/NEDD1/DCTN2/CEP89/KIF24/HAUS5/CEP250/HAUS8/ALMS1/CENPJ/OFD1/RAB3IP/CEP152/CEP135/PLK1/PLK4/CDK1/NEK2 | 5.29E-06 | REACTOME |
| REACTOME_G2_M_CHECKPOINTS | -0.513199448 | 7.11E-09 | RHNO1/MCM5/H4-16/H2AX/MDC1/H4C9/RFC5/TOP3A/WRN/TP53/MCM4/MCM3/TOPBP1/MCM6/RFC4/BRCA1/RBBP8/MCM7/NSD2/BARD1/H4C11/RFC3/DNA2/H2BC6/BLM/PKMYT1/DBF4/CDK2/MCM2/CDC6/CDC7/WEE1/MCM8/ORC6/CHEK1/CCNB1/H2BC9/CDC25C/CDK1/CDC45/ORC1/CCNB2/CLSPN/BRIP1/MCM10/EXO1/GTSE1/CDC25A | 5.32E-06 | REACTOME |
| REACTOME_POST_TRANSLATIONAL_MODIFICATION_SYNTHESIS_OF_GPI_ANCHORED_PROTEINS | 0.622218267 | 6.97E-05 | FCGR3B/FOLR2/CNTN5/GPIHBP1/ART3/RTN4RL1/LY6H/CNTN4/NEGR1/CNTN3/MSLN/LY6K/VNN2/LYPD5/CD52/XPNPEP2/ALPL/VNN1/LY6E/LYPD3/RTN4RL2/PRND/SPRN | 5.35E-06 | REACTOME |
| REACTOME_E2F_ENABLED_INHIBITION_OF_PRE_REPLICATION_COMPLEX_FORMATION | -0.881570224 | 0.000279308 | MCM8/ORC6/CCNB1/CDK1/ORC1 | 5.39E-06 | REACTOME |
| WP_FATTY_ACID_TRANSPORTERS | 0.69920246 | 0.038801494 | CD36/FABP4/FABP6/FABP3/ACSL6/ACSL5/ACSL1/ACSL4/FABP5 | 5.82E-06 | WP |
| WP_GABA_RECEPTOR_SIGNALING | 0.763980805 | 2.65E-05 | GABRA5/GABRA1/GABRB2/GAD2/GABRG1/GABRA4/GABRG2/SLC32A1/GABRD/GABRA2/GABRG3/GABRB3/GABBR1/GABRB1/SLC6A1/GABBR2/GAD1 | 6.10E-06 | WP |
| REACTOME_MITOTIC_G2_G2_M_PHASES | -0.334995543 | 0.000186878 | MAPRE1/CDC25B/TUBB/CDK5RAP2/CEP131/CEP164/CEP192/CEP57/CLASP1/HAUS3/CEP72/ODF2/NEDD1/TUBGCP4/TP53/DCTN2/E2F3/HAUS5/TUBB2B/AJUBA/BORA/CEP250/PPP2R3B/HAUS8/ALMS1/CENPJ/OFD1/E2F1/LIN9/HMMR/AURKA/PHLDA1/CEP152/PKMYT1/CDK2/CEP135/WEE1/CCNB1/PLK1/CDC25C/PLK4/CDK1/CCNA2/TPX2/MYBL2/CCNB2/NEK2/FOXM1/GTSE1/CDC25A/CENPF | 6.12E-06 | REACTOME |
| REACTOME_EXTENSION_OF_TELOMERES | -0.572819988 | 3.32E-05 | PRIM2/PIF1/WRAP53/POLD3/DKC1/RFC5/POLD1/GAR1/WRN/DSCC1/PCNA/FEN1/PRIM1/CHTF18/LIG1/RFC4/POLA1/RFC3/DNA2/BLM/CDK2/CCNA2/TERT | 6.23E-06 | REACTOME |
| REACTOME_CONDENSATION_OF_PROMETAPHASE_CHROMOSOMES | -0.860657422 | 0.000148397 | SMC2/NCAPD2/CCNB1/CDK1/NCAPG/SMC4/CCNB2/NCAPH | 6.49E-06 | REACTOME |
| REACTOME_TRANSCRIPTION_COUPLED_NUCLEOTIDE_EXCISION_REPAIR_TC_NER | -0.337609401 | 0.044942326 | COPS7B/TCEA1/POLK/XAB2/GTF2H3/ERCC3/USP7/PPIE/ERCC8/HMGN1/XRCC1/POLR2H/RFC1/MNAT1/RPA1/POLE3/RPA3/CUL4B/POLR2A/POLR2J/ZNF830/AQR/RFC2/EP300/LIG3/ERCC4/GTF2H4/POLD3/POLR2B/GTF2H2/POLR2D/RFC5/POLD1/UVSSA/PCNA/LIG1/RFC4/POLE2/RFC3/POLE | 6.73E-06 | REACTOME |
| KEGG_MISMATCH_REPAIR | -0.605168964 | 0.009446301 | MSH3/SSBP1/RFC1/RPA1/MSH2/RPA3/MLH3/RFC2/POLD3/RFC5/POLD1/MSH6/PCNA/LIG1/RFC4/RFC3/EXO1 | 6.89E-06 | KEGG |
| WP_METASTATIC_BRAIN_TUMOR | -0.788395863 | 0.022674436 | TP53/E2F3/MYC/CDK6/MIRLET7BHG | 7.12E-06 | WP |
| REACTOME_CARDIAC_CONDUCTION | 0.624022426 | 7.87E-08 | CAMK2A/SLC8A2/CASQ2/ATP2B3/RYR2/KCNJ12/KCNJ4/SCN2B/FGF13/SCN3B/KCNK4/PLN/KCNK1/KCNK12/CAMK2B/FXYD7/ATP1A3/SCN2A/ATP1B1/ATP2B2/KCNIP2/KCNJ11/CACNG8/KCNIP4/SCN1B/ITPR1/CAMK2G/CALM1/KCNK9/SCN8A/NPPC/KCNE1/CLIC2/FXYD1/CACNB2/CACNA2D2/FGF14/CACNB1/FKBP1B/ATP2A3/KCNQ1/KCNE5/ATP1A2/RYR1/SCN4B/FGF12/FXYD2/CASQ1/ATP2B1/NPR1/KCNK6/SLN/KCNK13 | 7.18E-06 | REACTOME |
| REACTOME_GABA_B_RECEPTOR_ACTIVATION | 0.688726235 | 0.000107774 | KCNJ12/KCNJ3/GNG3/KCNJ6/KCNJ4/KCNJ9/GABBR1/KCNJ5/GNAL/ADCY1/ADCY4/ADCY5/KCNJ15/GNAI1/ADCY2/GNGT2/GABBR2 | 7.61E-06 | REACTOME |
| REACTOME_TRANSPORT_OF_INORGANIC_CATIONS_ANIONS_AND_AMINO_ACIDS_OLIGOPEPTIDES | 0.608878301 | 1.64E-05 | SLC12A5/SLC8A2/SLC17A7/SLC38A4/SLC7A10/SLC26A9/SLC32A1/SLC24A4/SLC4A10/SLC6A12/SLC24A2/SLC6A15/SLC9A2/SLC1A6/SLC26A4/SLC25A18/SLC1A7/SLC7A8/SLC7A2/CALM1/SLC1A2/SLC15A3/SLC25A22/SLC43A1/SLC9A6/SLC7A7/SLC12A2/SLC38A3 | 7.64E-06 | REACTOME |
| BIOCARTA_BOTULIN_PATHWAY | 0.928039955 | 0.006906888 | SNAP25/CHRM1/STX1A/CHRNA1/VAMP2 | 7.83E-06 | BIOCARTA |
| WP_CALCIUM_REGULATION_IN_CARDIAC_CELLS | 0.665671022 | 7.11E-09 | PRKCG/CAMK2A/CASQ2/CACNA1B/ATP2B3/RYR2/GJB6/CHRM1/KCNJ3/ADRA1B/GNG3/PRKCB/RGS7/PLN/GJB1/ARRB1/RGS4/CAMK4/GJA5/ADRB1/CAMK2B/GJC2/ATP1B1/ATP2B2/PRKCZ/CHRM5/RGS20/KCNJ5/YWHAH/KCNB1/ITPR1/PRKAR1B/CAMK2G/ADCY1/CALM1/PRKCQ/ADCY4/RGS14/PRKCE/ADRA1A/CALM3/ADCY5/RGS5/GNAI1/SFN/RGS2/CHRM3/RGS10/RGS18/ADCY2/PRKACB/GJA4/PRKAR2B/PRKCD/CACNB1/RGS1/ATP2A3/ADRB2/RGS11/GNAO1/RYR1/PRKCH/CACNA1E/CACNA1D/ARRB2/CACNB3/FXYD2/CASQ1/ATP2B1/CHRM4/GNB5/CALM2/GNAZ/GNAS/GNG11/CACNA1C/YWHAG | 8.17E-06 | WP |
| REACTOME_HCMV_LATE_EVENTS | -0.472690461 | 0.000126528 | H2AC15/NUP58/HNRNPK/VPS25/NUP62/NUP133/NUP37/NUP43/RAE1/H3C6/NUP50/NUP88/NUP98/H4C5/H2BC11/NUP85/NUP93/H2BC5/H4C14/NUP153/POM121/H4-16/VPS37D/TPR/AAAS/H2AC8/H4C9/VPS37B/NUP155/NUP188/POM121C/NUP35/NDC1/NUP205/H3C10/NUP160/NUP42/H2AC20/NUP210/H2AC11/NUP107/H4C11/H2BC6/H3C14/H3C15/H2BC9 | 8.46E-06 | REACTOME |
| REACTOME_ION_HOMEOSTASIS | 0.704517753 | 3.55E-06 | CAMK2A/SLC8A2/CASQ2/ATP2B3/RYR2/PLN/CAMK2B/FXYD7/ATP1A3/ATP1B1/ATP2B2/KCNJ11/ITPR1/CAMK2G/CALM1/CLIC2/FXYD1/FKBP1B/ATP2A3/ATP1A2/RYR1/FXYD2/CASQ1/ATP2B1/SLN | 8.49E-06 | REACTOME |
| REACTOME_ERYTHROCYTES_TAKE_UP_OXYGEN_AND_RELEASE_CARBON_DIOXIDE | 0.90937174 | 0.003636441 | HBB/HBA2/CA4/HBA1 | 8.81E-06 | REACTOME |
| REACTOME_CELLULAR_SENESCENCE | -0.291015026 | 0.015772684 | RBBP7/H4C5/TNRC6B/H2BC11/H2AZ1/FZR1/H2AZ2/STAT3/SP1/CABIN1/RBBP4/CBX8/CCNE2/RELA/EP400/EHMT1/KDM6B/H2BC5/H4C14/ANAPC1/AGO3/H1-0/TNRC6A/H4-16/TNRC6C/H2AX/H2AC8/H4C9/TFDP2/HMGA1/ERF/TP53/SUZ12/E2F3/UBE2S/CDKN2C/H3C10/CCNE1/PHC1/H2AC20/MAPK7/E2F1/H4C11/ID1/H2BC6/CDK2/MDM4/H3C14/H3C15/EZH2/H2BC9/CDK6/CDK4/CCNA2/E2F2/CBX2/LMNB1/UBE2C/HMGA2 | 8.87E-06 | REACTOME |
| REACTOME_DNA_DOUBLE_STRAND_BREAK_RESPONSE | -0.373927478 | 0.028569016 | H4C15/RAD50/ATM/H2BC8/RNF168/UBE2I/KAT5/HERC2/MRE11/BRCC3/NBN/PIAS4/ABL1/TP53BP1/EYA4/H4C5/H2BC11/UIMC1/EYA2/SMARCA5/H2BC5/H4C14/H4-16/H2AX/MDC1/KDM4A/H4C9/BAZ1B/TP53/KPNA2/KDM4B/BRCA1/NSD2/BARD1/H4C11/H2BC6/H2BC9/EYA1 | 9.02E-06 | REACTOME |
| HALLMARK_MYC_TARGETS_V2 | -0.438977477 | 0.010191615 | NPM1/NOC4L/HK2/PHB/TCOF1/RABEPK/RRP9/HSPD1/PRMT3/PUS1/AIMP2/WDR74/MPHOSPH10/TBRG4/MRTO4/NIP7/MYBBP1A/DDX18/PPRC1/GNL3/NOP16/GRWD1/PES1/CBX3/MCM5/PA2G4/TFB2M/NOP56/UNG/BYSL/UTP20/WDR43/MCM4/IPO4/PPAN/NOP2/TMEM97/MYC/PLK1/PLK4/CDK4 | 9.09E-06 | HALLMARK |
| WP_CELLTYPE_DEPENDENT_SELECTIVITY_OF_CCK2R_SIGNALING | 0.724544583 | 0.044442943 | CCK/RYR2/ITPR1/GNAI1/RYR1/CD38/PLCB1 | 9.38E-06 | WP |
| REACTOME_ACTIVATION_OF_ATR_IN_RESPONSE_TO_REPLICATION_STRESS | -0.747182088 | 1.32E-08 | MCM4/MCM3/MCM6/RFC4/MCM7/RFC3/DBF4/CDK2/MCM2/CDC6/CDC7/MCM8/ORC6/CHEK1/CDC25C/CDC45/ORC1/CLSPN/MCM10/CDC25A | 9.42E-06 | REACTOME |
| BIOCARTA_PLCD_PATHWAY | 0.914448718 | 0.011441295 | ADRA1B/PRKCB/TGM2 | 9.69E-06 | BIOCARTA |
| WP_ATM_SIGNALING_PATHWAY | -0.577359686 | 0.000585361 | CASP2/H2AX/MDC1/TP53/SMC1A/CCNE1/BRCA1/RAD51/TP73/FANCD2/CDK2/MDM4/CHEK1/CCNB1/CDC25C/CDK1/CDC25A | 1.02E-05 | WP |
| REACTOME_MITOTIC_SPINDLE_CHECKPOINT | -0.59080073 | 7.11E-09 | CENPP/CLASP1/SPDL1/RCC2/CENPL/UBE2S/NUP160/INCENP/ZWILCH/CENPN/CENPU/CENPO/CENPH/NUP107/ZWINT/CENPM/KNTC1/SPC24/CENPK/PLK1/NDC80/MAD2L1/CDC20/CDCA8/AURKB/BIRC5/KIF2C/SGO2/SPC25/NUF2/SKA1/CENPE/ERCC6L/KIF18A/BUB1B/CENPA/SGO1/UBE2C/CENPI/BUB1/CENPF/KNL1 | 1.05E-05 | REACTOME |
| WP_MONOAMINE_TRANSPORT | 0.657929487 | 0.012014064 | HRH3/SYN1/STX1A/TNF/AMPH/TDO2/SLC6A1/DBH/IL1R1 | 1.12E-05 | WP |
| PID_MYC_ACTIV_PATHWAY | -0.376989559 | 0.01370013 | TFRC/HMGA1/TP53/TRRAP/TK1/E2F3/POLR3D/CAD/PEG10/RCC1/FOSL1/BCAT1/ODC1/MYC/CCNB1/CDCA7/CDK4/BIRC5/CDC25A/TERT | 1.16E-05 | PID |
| WP_RAS_SIGNALING | 0.535769639 | 1.24E-05 | PRKCG/GRIN1/PLA1A/GNG3/RASGRF2/RASGRF1/RASAL1/KSR2/PAK6/PRKCB/GRIN2A/GRIN2B/RAPGEF5/ZAP70/PAK1/TEK/CALM1/HTR7/RASGRP4/FGFR2/FLT3/CALM3/MAPK10/PLD1/FOXO4/PIK3CD/RASGRP3/MAPK3/PAK3/PRKACB/PLA2G4C/GNGT2/PAK5/MAPK9/ETS2/RASAL3/RAC2/PLAAT3/PDGFRB/ELK1/PLCG2/CSF1R/PLA2G2A/MRAS/GNB5/CALM2/RALB/LAT/GNG11/RASSF5/NGFR/MAP2K1/PIK3CB | 1.24E-05 | WP |
| WP_GPR143_IN_MELANOCYTES_AND_RETINAL_PIGMENT_EPITHELIUM_CELLS | 0.636564825 | 0.019102744 | GPR143/PRKCB/ARRB1/SERPINF1/ITPR1/ADCY4/POMC/ADCY2/PRKACB/PLCB4/ARRB2/PLCB2/GNA15/PLCB1/MITF/GNAS | 1.25E-05 | WP |
| REACTOME_DNA_DAMAGE_BYPASS | -0.450149282 | 0.01245147 | MAD2L2/POLD3/POLI/RFC5/POLD1/USP1/PCNA/POLH/RFC4/RAD18/POLE2/RFC3/POLE/REV3L/PCLAF/DTL | 1.26E-05 | REACTOME |
| HALLMARK_G2M_CHECKPOINT | -0.664392096 | 7.11E-09 | NCL/CKS1B/MAP3K20/LIG3/KPNB1/HNRNPD/SYNCRIP/HIF1A/MEIS1/HNRNPU/PRPF4B/NASP/PRMT5/PRIM2/DDX39A/CDC25B/SFPQ/MT2A/NOTCH2/MCM5/DTYMK/TOP1/CCNT1/H2AX/DKC1/SQLE/STAG1/ODF2/JPT1/SMARCC1/HMGA1/WRN/CHAF1A/E2F3/SUV39H1/ILF3/UBE2S/SLC38A1/LBR/CDKN2C/KPNA2/MCM3/CDKN3/INCENP/SMC1A/CCNF/KIF20B/MCM6/ODC1/NSD2/KIF22/BARD1/PTTG1/TMPO/SMC2/E2F1/HMMR/AURKA/RBL1/FANCC/TACC3/MYC/POLE/TRAIP/GINS2/CKS2/HMGB3/FBXO5/DBF4/STIL/MCM2/CDC6/CDC7/RACGAP1/RAD54L/ORC6/CHEK1/EZH2/BRCA2/PLK1/NDC80/MAD2L1/KIF4A/CDC20/PLK4/CDK1/CDK4/AURKB/BIRC5/KIF2C/PRC1/CCNA2/CENPE/CDC45/SMC4/KIF23/TPX2/E2F2/HOXC10/TTK/MYBL2/CCNB2/NEK2/CENPA/LMNB1/UBE2C/KIF15/POLQ/PBK/EXO1/KIF11/NUSAP1/BUB1/CDC25A/TROAP/ESPL1/CENPF/KNL1/TOP2A/MKI67 | 1.27E-05 | HALLMARK |
| WP_CELL_CYCLE | -0.53152886 | 7.11E-09 | STAG1/TFDP2/E2F5/TP53/PRKDC/PCNA/E2F3/MCM4/CDKN2C/MCM3/SMC1A/CCNE1/MCM6/MCM7/PTTG1/E2F1/RBL1/MYC/PKMYT1/DBF4/TGFB2/CDK2/MCM2/CDC6/CDC7/WEE1/ORC6/CHEK1/CCNB1/PLK1/CDK6/CDC25C/CDC20/CDK1/CDK4/CCNA2/CDC45/E2F2/TTK/ORC1/CCNB2/BUB1/CDC25A/ESPL1 | 1.41E-05 | WP |
| KEGG_CELL_CYCLE | -0.540458064 | 7.11E-09 | STAG1/TFDP2/E2F5/TP53/PRKDC/PCNA/E2F3/MCM4/CDKN2C/MCM3/SMC1A/CCNE1/MCM6/MCM7/PTTG1/E2F1/RBL1/MYC/PKMYT1/DBF4/TGFB2/CDK2/MCM2/CDC6/CDC7/WEE1/ORC6/CHEK1/CCNB1/PLK1/MAD2L1/CDK6/CDC25C/CDC20/CDK1/CDK4/CCNA2/CDC45/E2F2/TTK/BUB1B/ORC1/CCNB2/BUB1/CDC25A/ESPL1 | 1.45E-05 | KEGG |
| WP_GASTRIC_ACID_PRODUCTION | 0.998675159 | 0.000410662 | VIP/CCK | 1.49E-05 | WP |
| REACTOME_SURFACTANT_METABOLISM | 0.654607211 | 0.038471346 | SFTPC/ADRA2C/GATA6/ADRA2A/DMBT1/CSF2RB/ADA2/CSF2RA/CTSH/ADGRF5/P2RY2 | 1.49E-05 | REACTOME |
| REACTOME_SLC_MEDIATED_TRANSMEMBRANE_TRANSPORT | 0.543408686 | 7.73E-07 | SLC6A13/SLC12A5/SLC8A2/SLC17A7/SLC38A4/SLC7A10/SLC30A3/SLCO1A2/SLC26A9/SLC32A1/SLC24A4/SLC27A6/SLC4A10/SLC6A12/SLC13A5/SLC5A11/SLC24A2/SLC6A15/SLC45A3/SLC14A1/SLC9A2/SLC40A1/SLC1A6/SLC13A3/SLC5A4/SLC26A4/SLC22A3/SLC25A18/SLC1A7/LCN12/SLC7A8/SLC7A2/CALM1/SLC1A2/APOD/SLC6A1/SLCO2B1/SLC16A8/SLC2A12/SLC30A10/SLC15A3/SLC22A15/SLC25A22/SLCO3A1/SLC43A1/EMB/SLCO2A1/SLC2A4/SLC9A6/SLC7A7/SLC12A2/SLCO1C1/SLC5A10/SLC38A3/SLC29A3/SLC2A9/SLC25A4/SLC9A9 | 1.50E-05 | REACTOME |
| REACTOME_NEGATIVE_EPIGENETIC_REGULATION_OF_RRNA_EXPRESSION | -0.429702088 | 0.003517643 | ARID4B/GTF2H3/ERCC3/TAF1D/TBP/UBTF/BAZ2A/POLR2H/MNAT1/H3C6/TAF1B/H4C5/H2BC11/H2AZ1/H2AZ2/SMARCA5/HDAC2/SAP130/H2BC5/TTF1/H4C14/GTF2H4/POLR1C/SIN3A/GTF2H2/H4-16/TAF1A/H2AX/H2AC8/H4C9/POLR1A/SUV39H1/DNMT1/H3C10/POLR1B/H2AC20/DNMT3B/H4C11/H2BC6/H3C14/H3C15/H2BC9 | 1.60E-05 | REACTOME |
| WP_SULFATION_BIOTRANSFORMATION_REACTION | 0.713704335 | 0.036897421 | SULT4A1/SULT1C2/SULT1A1/SULT1B1/SULT1A4/SULT2B1/PAPSS2 | 1.61E-05 | WP |
| REACTOME_SEPARATION_OF_SISTER_CHROMATIDS | -0.434053946 | 1.32E-08 | CENPL/TUBB2B/UBE2S/NUP160/INCENP/SMC1A/ZWILCH/CENPN/PTTG1/CENPU/CENPO/CENPH/NUP107/ZWINT/CENPM/KNTC1/CDCA5/SPC24/CENPK/PLK1/NDC80/MAD2L1/CDC20/CDCA8/AURKB/BIRC5/KIF2C/SGO2/SPC25/NUF2/SKA1/CENPE/ERCC6L/KIF18A/BUB1B/CENPA/SGO1/UBE2C/CENPI/BUB1/ESPL1/CENPF/KNL1 | 1.68E-05 | REACTOME |
| REACTOME_AURKA_ACTIVATION_BY_TPX2 | -0.403731016 | 0.005256911 | NINL/CKAP5/HAUS1/SFI1/HAUS6/CSNK1E/CEP290/CEP76/TUBA1A/MAPRE1/TUBB/CDK5RAP2/CEP131/CEP164/CEP192/CEP57/CLASP1/HAUS3/CEP72/ODF2/NEDD1/DCTN2/HAUS5/CEP250/HAUS8/ALMS1/CENPJ/OFD1/HMMR/AURKA/CEP152/CEP135/PLK1/PLK4/CDK1/TPX2/NEK2 | 1.77E-05 | REACTOME |
| REACTOME_REGULATION_OF_PLK1_ACTIVITY_AT_G2_M_TRANSITION | -0.349238705 | 0.026868562 | SFI1/HAUS6/CSNK1E/CEP290/CEP76/TUBA1A/MAPRE1/TUBB/CDK5RAP2/CEP131/CEP164/CEP192/CEP57/CLASP1/HAUS3/CEP72/ODF2/NEDD1/DCTN2/HAUS5/AJUBA/BORA/CEP250/HAUS8/ALMS1/CENPJ/OFD1/AURKA/CEP152/CEP135/CCNB1/PLK1/PLK4/CDK1/CCNB2/NEK2 | 1.77E-05 | REACTOME |
| BIOCARTA_PTC1_PATHWAY | -0.72985199 | 0.015772684 | PTCH1/CCNB1/CDC25C/CDK1/CDC25A | 1.80E-05 | BIOCARTA |
| KEGG_PROXIMAL_TUBULE_BICARBONATE_RECLAMATION | 0.68805291 | 0.016890608 | GLS2/CA4/ATP1A3/ATP1B1/GLUD2/GLS/MDH1/ATP1A2/GLUD1/FXYD2/SLC38A3 | 1.84E-05 | KEGG |
| PID_E2F_PATHWAY | -0.535806839 | 7.68E-06 | RRM1/CBX5/TFDP2/E2F5/TRRAP/TK1/E2F3/E2F6/CDKN2C/MCM3/TOPBP1/CCNE1/DHFR/BRCA1/RBBP8/E2F1/POLA1/RBL1/MYC/SERPINE1/TP73/CDK2/CDC6/TYMS/CDK1/CCNA2/E2F2/E2F7/ORC1/MYBL2/RRM2/CDC25A | 1.89E-05 | PID |
| REACTOME_POSITIVE_EPIGENETIC_REGULATION_OF_RRNA_EXPRESSION | -0.453581063 | 0.001175872 | GSK3B/POLR2H/DDX21/GATAD2A/H3C6/TAF1B/RBBP7/H4C5/H2BC11/H2AZ1/MYBBP1A/H2AZ2/SF3B1/MTA1/MTA2/RBBP4/SMARCA5/HDAC2/CHD4/EP300/H2BC5/TTF1/GATAD2B/H4C14/POLR1C/DEK/CBX3/H4-16/TAF1A/H2AX/H2AC8/H4C9/BAZ1B/POLR1A/H3C10/POLR1B/H2AC20/H4C11/H2BC6/H3C14/H3C15/H2BC9 | 1.91E-05 | REACTOME |
| REACTOME_S_PHASE | -0.480270511 | 7.11E-09 | STAG1/RFC5/POLD1/TFDP2/E2F5/PCNA/GINS3/FEN1/MCM4/PRIM1/UBE2S/MCM3/SMC1A/CCNE1/LIG1/MCM6/RFC4/MCM7/E2F1/POLE2/LIN9/POLA1/RFC3/DNA2/MYC/POLE/GINS2/GINS1/GINS4/CDK2/MCM2/CDC6/CDT1/WEE1/MCM8/CDCA5/ORC6/CDK4/CCNA2/CDC45/ORC1/UBE2C/CDC25A/ESCO2 | 2.00E-05 | REACTOME |
| WP_COMMON_PATHWAYS_UNDERLYING_DRUG_ADDICTION | 0.709871609 | 5.00E-05 | PRKCG/DRD1/CAMK2A/GRIN1/PPP1R1A/GRM1/PRKCB/GRIN2A/GJB1/CAMK4/ADCY1/CALM1/GNAI1/ACTG2/MAPK3/PRKACB/DRD4 | 2.07E-05 | WP |
| HALLMARK_E2F_TARGETS | -0.685031907 | 7.11E-09 | IPO7/CSE1L/CKS1B/HNRNPD/SYNCRIP/PAN2/DEK/NUP153/NASP/PRIM2/DDX39A/SSRP1/CDC25B/RANBP1/TUBB/POLD3/SMC3/ING3/MMS22L/MCM5/RNASEH2A/PA2G4/CTPS1/SMC6/NOP56/UNG/H2AX/DCLRE1B/PNN/TFRC/STAG1/TCF19/CBX5/POLD1/JPT1/SNRPB/WDR90/HMGA1/TP53/DSCC1/PRKDC/USP1/PSMC3IP/PCNA/TK1/GINS3/SUV39H1/ILF3/DNMT1/MCM4/UBE2S/NUP205/LBR/CDKN2C/KPNA2/MCM3/CDKN3/SMC1A/HMGB2/CCNE1/LIG1/MCM6/TIPIN/BRCA1/MCM7/KIF22/BARD1/DIAPH3/PTTG1/TMPO/HELLS/NCAPD2/HMMR/AURKA/SPAG5/NUP107/TACC3/MXD3/RFC3/TIMELESS/MYC/POLE/PAICS/CKS2/GINS1/GINS4/E2F8/HMGB3/UBE2T/CDCA3/CENPM/MCM2/ATAD2/RAD51AP1/ASF1B/DEPDC1/WEE1/RACGAP1/SPC24/MELK/ORC6/CHEK1/TRIP13/EZH2/BRCA2/PLK1/MAD2L1/KIF4A/CDC20/PLK4/CDK1/CDK4/CDCA8/AURKB/BIRC5/KIF2C/SPC25/CENPE/SMC4/BUB1B/MYBL2/CCNB2/LMNB1/DLGAP5/RRM2/CDC25A/ESPL1/KIF18B/TOP2A/MKI67 | 2.16E-05 | HALLMARK |
| REACTOME_TELOMERE_C_STRAND_LAGGING_STRAND_SYNTHESIS | -0.569758367 | 0.002325473 | POLA2/RFC2/PRIM2/POLD3/RFC5/POLD1/WRN/DSCC1/PCNA/FEN1/PRIM1/CHTF18/LIG1/RFC4/POLA1/RFC3/DNA2/BLM | 2.18E-05 | REACTOME |
| REACTOME_MITOTIC_TELOPHASE_CYTOKINESIS | -0.672580868 | 0.022338494 | STAG2/SMC3/NIPBL/STAG1/SMC1A/PLK1/KIF20A/KIF23 | 2.24E-05 | REACTOME |
| REACTOME_BILE_ACID_AND_BILE_SALT_METABOLISM | 0.66129931 | 0.003274336 | SLCO1A2/AKR1C2/CYP46A1/CH25H/FABP6/AKR1C1/CYP39A1/AKR1C3/OSBPL1A/PTGIS/CYP7B1/STARD5 | 2.24E-05 | REACTOME |
| BIOCARTA_EFP_PATHWAY | -0.619320443 | 0.038937548 | TP53/CDK2/CCNB1/CDK6/CDK1/CDK4/CCNB2 | 2.25E-05 | BIOCARTA |
| REACTOME_NEGATIVE_REGULATION_OF_THE_PI3K_AKT_NETWORK | 0.472024703 | 0.034303649 | FGF7/NRG3/KL/CD28/LCK/IER3/PIP5K1B/FGF17/PIP4K2A/FGF22/FGFR2/FLT3/FGF9/FGF18/CD80/PIK3CD/MAPK3/TRAT1/PIK3AP1/FGF20/FGF1/VAV1/RAC2/PDGFRB/NRG4/PDGFA/ESR1/PDGFB/RHOG/CD86/TGFA/PTEN/EREG/PIK3CB/PIP5K1C/PPP2R5A | 2.28E-05 | REACTOME |
| REACTOME_CDC6_ASSOCIATION_WITH_THE_ORC_ORIGIN_COMPLEX | -0.821535712 | 0.000987952 | E2F3/E2F1/CDC6/MCM8/ORC6/E2F2/ORC1 | 2.40E-05 | REACTOME |
| REACTOME_INHIBITION_OF_THE_PROTEOLYTIC_ACTIVITY_OF_APC_C_REQUIRED_FOR_THE_ONSET_OF_ANAPHASE_BY_MITOTIC_SPINDLE_CHECKPOINT_COMPONENTS | -0.531440428 | 0.048189995 | UBE2S/MAD2L1/CDC20/BUB1B/UBE2C | 2.46E-05 | REACTOME |
| REACTOME_GABA_SYNTHESIS_RELEASE_REUPTAKE_AND_DEGRADATION | 0.847249588 | 8.94E-06 | SLC6A13/SYT1/SNAP25/GAD2/RAB3A/SLC32A1/CPLX1/STX1A/SLC6A12/RIMS1/STXBP1/VAMP2/SLC6A1/GAD1 | 2.53E-05 | REACTOME |
| REACTOME_INITIATION_OF_NUCLEAR_ENVELOPE_NE_REFORMATION | -0.582825576 | 0.039851977 | VRK1/LBR/TMPO/CCNB1/CDK1/CCNB2/LMNB1 | 2.53E-05 | REACTOME |
| REACTOME_RNA_POLYMERASE_I_TRANSCRIPTION | -0.381249233 | 0.008768632 | POLR2H/MNAT1/GATAD2A/H3C6/TAF1B/RBBP7/H4C5/H2BC11/H2AZ1/H2AZ2/MTA1/MTA2/RBBP4/HDAC2/CHD4/H2BC5/TTF1/GATAD2B/H4C14/GTF2H4/POLR1C/GTF2H2/RRN3/CBX3/H4-16/TAF1A/H2AX/H2AC8/H4C9/POLR1A/H3C10/POLR1B/H2AC20/H4C11/H2BC6/H3C14/H3C15/H2BC9 | 2.56E-05 | REACTOME |
| REACTOME_E2F_MEDIATED_REGULATION_OF_DNA_REPLICATION | -0.697640407 | 0.000519913 | TFDP2/PRIM1/PPP2R3B/E2F1/POLA1/MCM8/ORC6/CCNB1/CDK1/ORC1 | 2.64E-05 | REACTOME |
| REACTOME_TRANSCRIPTION_OF_E2F_TARGETS_UNDER_NEGATIVE_CONTROL_BY_DREAM_COMPLEX | -0.742940915 | 0.000353931 | TFDP2/E2F5/PCNA/E2F1/LIN9/RBL1/MYC/CDC6/CDC25A/TOP2A | 2.69E-05 | REACTOME |
| BIOCARTA_RACC_PATHWAY | 0.732896875 | 0.021824481 | KCNQ5/GUCY1B1/KCNQ3/ADCY1/GUCY1A1/NOS3/KCNQ4/KCNQ2/TRPV4/GNAS | 2.77E-05 | BIOCARTA |
| REACTOME_RESOLUTION_OF_SISTER_CHROMATID_COHESION | -0.511866959 | 1.85E-08 | CENPP/CLASP1/SPDL1/STAG1/RCC2/CENPL/TUBB2B/NUP160/INCENP/SMC1A/ZWILCH/CENPN/CENPU/CENPO/CENPH/NUP107/ZWINT/CENPM/KNTC1/CDCA5/SPC24/CCNB1/CENPK/PLK1/NDC80/MAD2L1/CDC20/CDK1/CDCA8/AURKB/BIRC5/KIF2C/SGO2/SPC25/NUF2/SKA1/CENPE/ERCC6L/KIF18A/BUB1B/CCNB2/CENPA/SGO1/CENPI/BUB1/CENPF/KNL1 | 2.79E-05 | REACTOME |
| KEGG_BASE_EXCISION_REPAIR | -0.495866771 | 0.017993867 | XRCC1/POLE3/PARP1/HMGB1/MUTYH/TDG/APEX1/LIG3/POLD3/UNG/POLD1/PCNA/FEN1/LIG1/POLE2/POLE/NEIL3 | 2.97E-05 | KEGG |
| WP_PROSTAGLANDIN_AND_LEUKOTRIENE_METABOLISM_IN_SENESCENCE | 0.617113758 | 0.036211611 | PTGER2/PTGDS/LTC4S/GNAI1/PTGER3/PTGIS/PTGES/PTGER1/CYSLTR1/TBXAS1/PLCB1/PTGER4/GNAS/PTGS2/MAPK11/ALOX5/GNAQ/HRAS | 2.98E-05 | WP |
| REACTOME_PROCESSIVE_SYNTHESIS_ON_THE_C_STRAND_OF_THE_TELOMERE | -0.581278517 | 0.04042466 | POLD3/POLD1/WRN/PCNA/FEN1/LIG1/DNA2/BLM | 3.01E-05 | REACTOME |
| REACTOME_FGFR2C_LIGAND_BINDING_AND_ACTIVATION | 0.82622876 | 0.021953141 | FGF17/FGFR2/FGF9/FGF18/FGF20/FGF1 | 3.34E-05 | REACTOME |
| REACTOME_ROLE_OF_PHOSPHOLIPIDS_IN_PHAGOCYTOSIS | 0.59703404 | 0.044980365 | IGHG4/ITPR1/CD247/PRKCE/CD3G/PLD1/PLD3/FCGR3A/PRKCD/SYK/FCGR2A/PLCG2/FCGR1A/PLD4 | 3.44E-05 | REACTOME |
| HALLMARK_MYOGENESIS | 0.515390761 | 7.09E-05 | CASQ2/MYH11/COL15A1/CD36/LDB3/VIPR1/COL6A3/IGF1/GJA5/CAMK2B/HRC/COL3A1/MYL3/COX7A1/KCNH1/TPD52L1/COL1A1/MYOM1/FABP3/CHRNA1/MEF2C/CFD/TNNT1/SGCA/BIN1/APOD/FOXO4/FXYD1/MAPRE3/SOD3/SVIL/SCD/MYL4/PPP1R3C/FKBP1B/PRNP/NQO1/PTGIS/CKMT2/GNAO1/RYR1/CRYAB/ACSL1/HSPB8/GSN/SYNGR2/MEF2D/GPX3/CASQ1/MRAS/SGCD/MYOM2/APLNR/SLN/CKB/MYLK/PC/DMD/MYOZ1/LSP1/COL6A2/ABLIM1/SPHK1/GABARAPL2/GADD45B/MEF2A/CDH13/HDAC5/GAA/AK1/BAG1/TPM3/DTNA/EIF4A2/MYH9/LARGE1/TCAP/SH3BGR/PPFIA4/DAPK2/PDE4DIP/HSPB2/ACHE | 3.52E-05 | HALLMARK |
| REACTOME_CHROMOSOME_MAINTENANCE | -0.561277708 | 7.11E-09 | ANKRD28/PPP6R3/ATRX/POLR2A/RTEL1/RBBP7/H4C5/H2BC11/POLR2J/H2AZ1/H2AZ2/MIS18BP1/DAXX/RBBP4/SMARCA5/POLA2/RFC2/H2BC5/ITGB3BP/H4C14/CENPQ/PRIM2/PIF1/WRAP53/POLD3/POLR2B/RSF1/MIS18A/H4-16/POLR2D/H2AX/DKC1/H2AC8/CENPP/H4C9/RFC5/POLD1/GAR1/WRN/DSCC1/PCNA/CENPL/FEN1/PRIM1/CHTF18/OIP5/LIG1/CENPN/RFC4/H2AC20/CENPU/POLA1/CENPO/CENPH/H4C11/RFC3/DNA2/H2BC6/BLM/CDK2/CENPM/CENPK/H2BC9/CCNA2/CENPA/CENPI/HJURP/KNL1/TERT | 3.62E-05 | REACTOME |
| REACTOME_G1_S_SPECIFIC_TRANSCRIPTION | -0.777040456 | 6.02E-08 | TFDP2/E2F5/PCNA/TK1/E2F6/CCNE1/DHFR/E2F1/LIN9/POLA1/RBL1/FBXO5/CDC6/TYMS/CDT1/CDK1/CDC45/ORC1/RRM2/CDC25A | 3.66E-05 | REACTOME |
| HALLMARK_MITOTIC_SPINDLE | -0.380621655 | 1.35E-06 | SEPTIN9/CEP250/FLNA/INCENP/SMC1A/PLEKHG2/ALMS1/SASS6/KIF20B/CENPJ/TRIO/KIF22/AURKA/FBXO5/KNTC1/RACGAP1/ECT2/BRCA2/PLK1/NDC80/KIF4A/CDK1/BIRC5/KIF2C/PRC1/CENPE/SMC4/KIF23/TPX2/TTK/CCNB2/NEK2/LMNB1/KIF15/DLGAP5/KIF11/NUSAP1/BUB1/ESPL1/CENPF/TOP2A | 3.67E-05 | HALLMARK |
| REACTOME_GLUCAGON_TYPE_LIGAND_RECEPTORS | 0.636303138 | 0.043641688 | VIP/GNG3/VIPR1/GNGT2/GIPR/GNB5/CYSLTR2/GNAS/GNG11 | 3.70E-05 | REACTOME |
| HALLMARK_MYC_TARGETS_V1 | -0.412662524 | 1.78E-08 | CLNS1A/SF3A1/ERH/PHB/CCT3/TFDP1/GSPT1/IMPDH2/EIF1AX/SRSF2/RRP9/NCBP2/HSPD1/PSMD3/SSBP1/RNPS1/SRSF3/DUT/UBE2E1/G3BP1/SNRPA1/PHB2/POLE3/DDX21/AIMP2/TARDBP/UBA2/NCBP1/SNRPB2/FBL/IARS1/XPO1/ORC2/HNRNPA1/SET/SERBP1/EIF4A1/NME1/EPRS1/RAD23B/HNRNPA3/SNRPA/SNRPD1/H2AZ1/DDX18/CANX/IFRD1/ABCE1/TRA2B/SNRPG/SF3B3/ILF2/SRPK1/EIF3B/HDAC2/SRSF1/PRDX4/GNL3/NOP16/APEX1/LDHA/TRIM28/KPNB1/HNRNPD/PPM1G/SYNCRIP/LSM7/PGK1/DEK/HNRNPA2B1/HNRNPU/DHX15/RANBP1/HNRNPR/U2AF1/CBX3/MCM5/XPOT/PA2G4/CTPS1/NOP56/CSTF2/RRM1/SMARCC1/USP1/PCNA/MCM4/CAD/CCT2/KPNA2/MCM6/ODC1/RFC4/MCM7/MYC/CDK2/MCM2/TYMS/MAD2L1/CDC20/CDK4/CCNA2/CDC45 | 3.87E-05 | HALLMARK |
| BIOCARTA_ATM_PATHWAY | -0.535053642 | 0.047396194 | RAD50/ATM/MDM2/JUN/MRE11/NBN/GADD45A/ABL1/RELA/TP53/BRCA1/RBBP8/RAD51/TP73/CHEK1 | 4.09E-05 | BIOCARTA |
| REACTOME_TRANSCRIPTION_OF_E2F_TARGETS_UNDER_NEGATIVE_CONTROL_BY_P107_RBL1_AND_P130_RBL2_IN_COMPLEX_WITH_HDAC1 | -0.739054388 | 0.001311999 | TFDP2/E2F5/E2F1/LIN9/RBL1/CDK1/CCNA2/MYBL2 | 4.18E-05 | REACTOME |
| REACTOME_CHEMOKINE_RECEPTORS_BIND_CHEMOKINES | 0.745894354 | 1.33E-06 | CCL19/CCL3/CCL3L3/CXCL11/CCL4/CXCL12/CXCR1/PPBP/CXCR2/CCL5/CXCL9/CXCL10/CXCL1/CXCR3/CXCR6/CCR6/CXCL6/CCL28/CCR1/CCR4/CXCL3/CCRL2/CCR2/CCR7/CXCL2/CXCL5/CCR5/CXCL8/CX3CR1 | 4.20E-05 | REACTOME |
| REACTOME_DISEASES_OF_MITOTIC_CELL_CYCLE | -0.466200042 | 0.011001334 | ANAPC7/UBE2E1/CDC23/ANAPC4/ATRX/FZR1/CCND2/DAXX/CCNE2/ANAPC1/SKP2/TFDP2/E2F3/UBE2S/CCNE1/E2F1/CDK2/CDK6/CDK4/E2F2/UBE2C | 4.39E-05 | REACTOME |
| REACTOME_TP53_REGULATES_TRANSCRIPTION_OF_GENES_INVOLVED_IN_G2_CELL_CYCLE_ARREST | -0.564432968 | 0.045262991 | TFDP2/TP53/PCNA/AURKA/RBL1/CCNB1/CDC25C/CDK1 | 4.40E-05 | REACTOME |
| BIOCARTA_MCM_PATHWAY | -0.765953769 | 0.00016603 | MCM4/MCM3/CCNE1/MCM6/MCM7/CDK2/MCM2/CDC6/CDT1/ORC6/ORC1 | 4.43E-05 | BIOCARTA |
| REACTOME_VOLTAGE_GATED_POTASSIUM_CHANNELS | 0.733422358 | 6.15E-05 | KCNC2/KCNH5/KCNA4/KCNS1/KCNH3/KCNAB2/KCNA1/KCNQ5/KCNAB1/KCNG1/KCNH1/KCNB1/KCNQ3/KCNC1/KCNG2/KCNC3/KCNC4/KCNA5/KCNQ1/KCNH8/KCNQ4/KCNQ2 | 4.63E-05 | REACTOME |
| REACTOME_REGULATION_OF_INSULIN_SECRETION | 0.686009367 | 2.35E-07 | KCNC2/SNAP25/GNG3/CD36/RAPGEF4/SYT5/AKAP5/STX1A/ADRA2C/STXBP1/ABCC8/KCNJ11/ADRA2A/KCNB1/GNA14/ITPR1/PRKAR1B/VAMP2/RAPGEF3/ADCY5/KCNG2/GNAI1/CHRM3/CACNB2/PRKACB/GNGT2/CACNA2D2/PRKAR2B/CACNA1E/CACNA1D/CACNB3/PLCB2/GNA15/PLCB1/GNB5/ACSL4/GNAS/GNG11/CACNA1C | 4.69E-05 | REACTOME |
| REACTOME_TELOMERE_EXTENSION_BY_TELOMERASE | -0.528585531 | 0.04647918 | PIF1/WRAP53/DKC1/GAR1/CDK2/CCNA2/TERT | 4.71E-05 | REACTOME |
| REACTOME_DNA_REPLICATION | -0.504648295 | 7.11E-09 | MCM5/SKP2/H4-16/H2AX/H2AC8/GMNN/H4C9/RFC5/POLD1/PCNA/GINS3/E2F3/FEN1/MCM4/PRIM1/UBE2S/H3C10/MCM3/CCNE1/LIG1/MCM6/RFC4/MCM7/H2AC20/E2F1/POLE2/POLA1/H4C11/RFC3/DNA2/POLE/GINS2/H2BC6/GINS1/GINS4/DBF4/CDK2/MCM2/CDC6/CDT1/H3C14/H3C15/CDC7/MCM8/ORC6/H2BC9/CCNA2/CDC45/E2F2/ORC1/UBE2C/MCM10 | 4.80E-05 | REACTOME |
| REACTOME_SYNTHESIS_OF_DNA | -0.469481948 | 9.66E-07 | PCNA/GINS3/FEN1/MCM4/PRIM1/UBE2S/MCM3/CCNE1/LIG1/MCM6/RFC4/MCM7/POLE2/POLA1/RFC3/DNA2/POLE/GINS2/GINS1/GINS4/CDK2/MCM2/CDC6/CDT1/MCM8/ORC6/CCNA2/CDC45/ORC1/UBE2C | 4.80E-05 | REACTOME |
| REACTOME_RHO_GTPASES_ACTIVATE_FORMINS | -0.451029133 | 6.35E-07 | CENPP/CLASP1/SPDL1/DVL2/RCC2/CENPL/TUBB2B/NUP160/INCENP/ZWILCH/CENPN/DIAPH3/CENPU/CENPO/CENPH/NUP107/ZWINT/CENPM/KNTC1/SPC24/CENPK/PLK1/NDC80/MAD2L1/CDC20/CDCA8/AURKB/BIRC5/KIF2C/SGO2/SPC25/NUF2/SKA1/CENPE/ERCC6L/KIF18A/BUB1B/CENPA/SGO1/CENPI/BUB1/CENPF/KNL1 | 4.95E-05 | REACTOME |
| WP_GPCRS_CLASS_C_METABOTROPIC_GLUTAMATE_PHEROMONE | 0.719771749 | 0.044739547 | GRM1/GRM2/GRM3/GRM7/GABBR1/GABBR2/GPRC5C/GRM5/GRM8 | 4.96E-05 | WP |
| WP_EFFECT_OF_PROGERIN_ON_GENES_INVOLVED_IN_HUTCHINSONGILFORD_PROGERIA_SYNDROME | -0.503708965 | 0.023473549 | CBX1/H3C6/RBBP7/MTA1/MTA2/RBBP4/HDAC2/CHD4/CBX3/CBX5/KDM1A/TP53/SUV39H1/H3C10/E2F1/H3C14/H3C15 | 4.98E-05 | WP |
| WP_DEREGULATION_OF_RAB_AND_RAB_EFFECTOR_GENES_IN_BLADDER_CANCER | 0.728139549 | 0.01245147 | SYTL1/MYRIP/RPH3A/RAB27B/MLPH/MADD/RPH3AL/SYTL5/UNC13D/EXPH5/SYTL2/SYTL3/GCC2 | 5.18E-05 | WP |
| KEGG_LONG_TERM_DEPRESSION | 0.561598197 | 0.010876024 | PRKCG/GRM1/CRHR1/PRKCB/IGF1/GUCY1B1/ITPR1/GUCY1A1/GNAI1/MAPK3/PLCB4/GNAO1/RYR1/GRM5/PRKG1/PLCB2/PLA2G2A/PLCB1/GNAZ/GNAS/LYN/MAP2K1 | 5.35E-05 | KEGG |
| PID_ANTHRAX_PATHWAY | 0.721507435 | 0.019901658 | PGR/TNF/NLRP1/MAPK3/MAP2K4/IL1B/IL18/CASP1/MAP2K1 | 5.46E-05 | PID |
| KEGG_VASCULAR_SMOOTH_MUSCLE_CONTRACTION | 0.612131279 | 3.54E-06 | PRKCG/MYH11/ADRA1B/PRKCB/CACNA1F/PPP1R14A/GUCY1B1/ITPR1/KCNMB4/ADCY1/CALM1/PRKCQ/ADCY4/GUCY1A1/RAMP3/PRKCE/ADRA1A/CALM3/ADCY5/IRAG1/KCNMA1/ACTG2/ADCY2/MAPK3/PRKACB/PRKCD/PLCB4/PRKCH/PPP1R12B/CACNA1D/PRKG1/PLCB2/NPR1/PLA2G2A/MYL9/PLCB1/ACTA2/MYLK/KCNMB2/CALM2/RAMP2/GNAS/CACNA1C/MAP2K1/AGTR1 | 5.47E-05 | KEGG |
| REACTOME_NEUROTRANSMITTER_RELEASE_CYCLE | 0.826399058 | 7.11E-09 | SLC6A13/SLC17A7/SYT1/GLS2/SYN2/SNAP25/GAD2/SYN1/RAB3A/SLC32A1/CPLX1/STX1A/SLC6A12/RIMS1/STXBP1/SLC1A6/SLC1A7/TSPOAP1/PPFIA2/VAMP2/SLC1A2/PPFIA3/APBA1/LIN7B/SLC6A1/GLS/MAOA/GAD1/NAAA/SYN3 | 5.48E-05 | REACTOME |
| REACTOME_ORGANIC_ANION_TRANSPORTERS | 0.837379397 | 0.034303649 | SLC17A7/SLC25A18/SLC25A22 | 5.60E-05 | REACTOME |
| BIOCARTA_BAD_PATHWAY | 0.670256825 | 0.01089435 | IGF1/YWHAH/PRKAR1B/ADCY1/CSF2RB/MAPK3/PRKACB/PRKAR2B/IL3RA | 5.78E-05 | BIOCARTA |
| REACTOME_PLATELET_CALCIUM_HOMEOSTASIS | 0.651143727 | 0.017104908 | SLC8A2/ATP2B3/P2RX5/ATP2B2/ITPR1/CALM1/ATP2A3/P2RX1/ATP2B1/P2RX4/SLC8A1/P2RX6/STIM1 | 5.85E-05 | REACTOME |
| PID_PLK1_PATHWAY | -0.689069138 | 7.97E-08 | CENPU/AURKA/FBXO5/WEE1/SPC24/CCNB1/ECT2/PLK1/NDC80/CDC25C/CDC20/KIF20A/CDK1/PRC1/CENPE/ERCC6L/TPX2/BUB1B/CLSPN/SGO1/BUB1 | 6.09E-05 | PID |
| REACTOME_AMINO_ACID_TRANSPORT_ACROSS_THE_PLASMA_MEMBRANE | 0.611424202 | 0.024044424 | SLC38A4/SLC7A10/SLC6A12/SLC6A15/SLC7A8/SLC7A2/SLC43A1/SLC7A7/SLC38A3 | 6.31E-05 | REACTOME |
| REACTOME_CYCLIN_A_B1_B2_ASSOCIATED_EVENTS_DURING_G2_M_TRANSITION | -0.752853312 | 4.72E-06 | PPP2R3B/PKMYT1/CDK2/WEE1/CCNB1/PLK1/CDC25C/CDK1/CCNA2/CCNB2/FOXM1/CDC25A | 6.32E-05 | REACTOME |
| WP_PROSTAGLANDIN_SYNTHESIS_AND_REGULATION | 0.641793048 | 0.001775128 | ANXA3/PTGER2/AKR1C2/PTGDS/PTGFR/AKR1C1/HPGD/HSD11B1/AKR1C3/PPARG/PTGER3/HPGDS/PTGIS/HSD11B2/PTGES/EDN1/PTGER1/TBXAS1/PTGER4/PPARGC1A/MITF/CBR1/ANXA4/PTGS2/PTGIR | 6.41E-05 | WP |
| BIOCARTA_CSK_PATHWAY | 0.785387946 | 0.000353931 | LCK/ZAP70/CD3E/PRKAR1B/CD247/ADCY1/CD3D/CD3G/PRKACB/PRKAR2B/CD4/HLA-DRB1/HLA-DRA/HLA-DRB5/PTPRC | 6.56E-05 | BIOCARTA |
| REACTOME_RESOLUTION_OF_AP_SITES_VIA_THE_MULTIPLE_NUCLEOTIDE_PATCH_REPLACEMENT_PATHWAY | -0.585839518 | 0.009855177 | RFC1/RPA1/POLE3/RPA3/PARP1/RFC2/APEX1/POLD3/RFC5/POLD1/PCNA/FEN1/LIG1/RFC4/POLE2/RFC3/POLE | 6.61E-05 | REACTOME |
| WP_BASE_EXCISION_REPAIR | -0.502578813 | 0.025582851 | XRCC1/POLE3/PARP1/HMGB1/MUTYH/TDG/APEX1/LIG3/POLD3/UNG/POLD1/PCNA/FEN1/LIG1/POLE2/POLE/NEIL3 | 6.67E-05 | WP |
| BIOCARTA_PGC1A_PATHWAY | 0.751431841 | 0.007595192 | CAMK1G/CAMK4/YWHAH/PPP3CB/CALM1/CALM3/PPP3CA/SLC2A4/PPARGC1A/CALM2 | 7.05E-05 | BIOCARTA |
| WP_MIRNA_REGULATION_OF_DNA_DAMAGE_RESPONSE | -0.4785045 | 0.000345964 | TP53/PRKDC/SMC1A/CCNE1/BRCA1/MCM7/E2F1/MYC/RAD51/FANCD2/CDK2/CHEK1/CCNB1/CDK6/CDC25C/CDK1/CDK4/CCNB2/MIR17HG/CDC25A | 7.08E-05 | WP |
| WP_DNA_DAMAGE_RESPONSE | -0.455369952 | 0.001472045 | TP53/PRKDC/SMC1A/CCNE1/BRCA1/E2F1/MYC/RAD51/FANCD2/CDK2/CHEK1/CCNB1/CDK6/CDC25C/CDK1/CDK4/CCNB2/CDC25A | 7.41E-05 | WP |
| WP_GASTRIC_CANCER_NETWORK_2 | -0.612471512 | 0.001251249 | PLAC8/AHCTF1/CEBPZ/COL9A3/EGFR/TP53/DSCC1/LBR/CHTF18/RFC4/COL9A1/RFC3/MYC/UBE2T/ATAD2/LMNB2/FANCI/UBE2C/TOP2A | 7.49E-05 | WP |
| REACTOME_TP53_REGULATES_TRANSCRIPTION_OF_CELL_CYCLE_GENES | -0.418782359 | 0.028790816 | TFDP2/TP53/PCNA/CCNE1/CENPJ/E2F1/AURKA/RBL1/E2F8/CDK2/CCNB1/CDC25C/CDK1/CCNA2/E2F7 | 7.87E-05 | REACTOME |
| REACTOME_TELOMERE_MAINTENANCE | -0.503226434 | 2.87E-06 | POLR2H/RFC1/RPA1/RPA3/ANKRD28/PPP6R3/ATRX/POLR2A/RTEL1/H4C5/H2BC11/POLR2J/H2AZ1/H2AZ2/DAXX/POLA2/RFC2/H2BC5/H4C14/PRIM2/PIF1/WRAP53/POLD3/POLR2B/H4-16/POLR2D/H2AX/DKC1/H2AC8/H4C9/RFC5/POLD1/GAR1/WRN/DSCC1/PCNA/FEN1/PRIM1/CHTF18/LIG1/RFC4/H2AC20/POLA1/H4C11/RFC3/DNA2/H2BC6/BLM/CDK2/H2BC9/CCNA2/TERT | 7.96E-05 | REACTOME |
| WP_DNA_REPLICATION | -0.709293452 | 8.10E-08 | PRIM2/POLD3/MCM5/GMNN/RFC5/POLD1/PCNA/MCM4/PRIM1/MCM3/MCM6/RFC4/MCM7/POLE2/POLA1/RFC3/POLE/DBF4/CDK2/MCM2/CDC6/CDT1/CDC7/ORC6/CDC45/ORC1/MCM10 | 8.09E-05 | WP |
| REACTOME_ESTABLISHMENT_OF_SISTER_CHROMATID_COHESION | -0.676967902 | 0.038948023 | ESCO1/STAG2/SMC3/STAG1/SMC1A/CDCA5/ESCO2 | 8.38E-05 | REACTOME |
| BIOCARTA_GABA_PATHWAY | 0.883497239 | 0.001008846 | GABRA5/GABRA1/GABRA4/GABRA2/NSF | 8.60E-05 | BIOCARTA |
| REACTOME_ACTIVATION_OF_ANTERIOR_HOX_GENES_IN_HINDBRAIN_DEVELOPMENT_DURING_EARLY_EMBRYOGENESIS | -0.455798507 | 7.46E-05 | RBBP7/H4C5/CNOT9/H2BC11/POLR2J/H2AZ1/H2AZ2/CREBBP/RBBP4/CNOT6/HOXA2/EP300/H2BC5/H4C14/PIAS2/MEIS1/RARA/POLR2B/WDR5/KMT2C/H4-16/POLR2D/KMT2D/H2AX/PCGF2/H2AC8/H4C9/HOXB4/NCOA6/SUZ12/AJUBA/H3C10/PAXIP1/H2AC20/HOXC4/HOXA3/H4C11/HOXA4/H2BC6/H3C14/H3C15/HOXD3/HOXD4/EZH2/H2BC9 | 8.82E-05 | REACTOME |
| REACTOME_SYNTHESIS_OF_BILE_ACIDS_AND_BILE_SALTS | 0.618188066 | 0.031381089 | AKR1C2/CYP46A1/CH25H/AKR1C1/CYP39A1/AKR1C3/OSBPL1A/PTGIS/CYP7B1 | 9.29E-05 | REACTOME |
| WP_COHESIN_COMPLEX_CORNELIA_DE_LANGE_SYNDROME | -0.606815791 | 0.00048419 | ESCO1/STAG2/SMC3/NIPBL/REC8/SMC1A/PTTG1/CDCA5/PLK1/CDK1/AURKB/SGO2/SGO1/ESPL1/ESCO2 | 9.35E-05 | WP |
| REACTOME_MEIOSIS | -0.475747418 | 7.59E-05 | H2BC5/H4C14/STAG2/SUN1/SMC3/H4-16/REC8/DIDO1/H2AX/H2AC8/H4C9/STAG1/TOP3A/PSMC3IP/H3C10/SMC1A/BRCA1/RBBP8/H2AC20/SYCP3/H4C11/RAD51/H2BC6/BLM/MSH5/SYNE2/CDK2/H3C14/H3C15/MND1/H2BC9/BRCA2/CDK4/LMNB1 | 9.37E-05 | REACTOME |
| WP_RETINOBLASTOMA_GENE_IN_CANCER | -0.663266787 | 7.11E-09 | CDC25B/SIN3A/POLD3/SMC3/SKP2/FANCG/RRM1/RFC5/TFDP2/MSH6/TP53/PRKDC/PCNA/E2F3/SUV39H1/DNMT1/MCM4/PRIM1/MCM3/SMC1A/HMGB2/CCNE1/MCM6/DHFR/RFC4/MCM7/BARD1/SMC2/E2F1/POLE2/POLA1/RFC3/MYC/POLE/CDK2/TYMS/CDT1/CDC7/WEE1/CHEK1/CCNB1/CDK6/KIF4A/PLK4/CDK1/CDK4/CCNA2/CDC45/E2F2/TTK/ORC1/CCNB2/RRM2/CDC25A/TOP2A | 9.40E-05 | WP |
| WP_H19_ACTION_RBE2F1_SIGNALING_AND_CDKBETACATENIN_ACTIVITY | -0.715856536 | 0.003681911 | TULP3/MED1/E2F1/JAG1/CDK4/H19/CDH1/SOX4 | 9.43E-05 | WP |
| WP_TUMOR_SUPPRESSOR_ACTIVITY_OF_SMARCB1 | -0.515174493 | 0.017409342 | SMARCA4/ARID1A/SMO/SMARCC1/SMARCD1/SUZ12/DPF3/PTCH1/GLI1/EZH2/GLI2/CDK6/CDK4 | 9.58E-05 | WP |
| WP_DNA_MISMATCH_REPAIR | -0.617050777 | 0.006849108 | RFC1/RPA1/MSH2/POLE3/RPA3/RFC2/POLD3/RFC5/POLD1/MSH6/PCNA/LIG1/RFC4/POLE2/RFC3/POLE/EXO1 | 9.59E-05 | WP |
| REACTOME_PRE_NOTCH_EXPRESSION_AND_PROCESSING | -0.43079415 | 0.000617693 | POGLUT1/RFNG/H4C5/TNRC6B/H2BC11/H2AZ1/H2AZ2/CREBBP/EP300/H2BC5/H4C14/MAML1/AGO3/TNRC6A/NOTCH2/H4-16/TNRC6C/H2AX/H2AC8/H4C9/TFDP2/TP53/MAMLD1/AGO2/E2F3/H3C10/MAML2/POFUT1/H2AC20/E2F1/NOTCH1/H4C11/H2BC6/H3C14/H3C15/H2BC9 | 9.62E-05 | REACTOME |
| REACTOME_MITOTIC_G1_PHASE_AND_G1_S_TRANSITION | -0.525242923 | 7.11E-09 | PCNA/TK1/E2F3/MCM4/PRIM1/E2F6/CDKN2C/MCM3/PPP2R3B/CCNE1/MCM6/DHFR/MCM7/E2F1/POLE2/LIN9/POLA1/RBL1/MYC/POLE/FBXO5/DBF4/CDK2/MCM2/CDC6/TYMS/CDT1/CDC7/WEE1/MCM8/ORC6/CCNB1/CDK6/CDK1/CDK4/CCNA2/CDC45/E2F2/ORC1/MYBL2/MCM10/RRM2/CDC25A/TOP2A | 0.000100862 | REACTOME |
| WP_TRYPTOPHAN_METABOLISM | 0.63408341 | 0.015772684 | CYP1B1/ALDH1A2/ALDH1A1/TDO2/INMT/CYP2E1/CYP2J2/AOX1/HAAO/ALDH2/CYP7B1/IDO1/KYNU/DHCR24/ECHS1 | 0.000101899 | WP |
| KEGG_TYPE_II_DIABETES_MELLITUS | 0.604062144 | 0.007479416 | CACNA1B/TNF/ABCC8/PRKCZ/KCNJ11/PRKCE/MAPK10/PIK3CD/MAPK3/HK3/PRKCD/MAPK9/PIK3R5/CACNA1E/CACNA1D/SOCS1/SLC2A4/GCK/CACNA1C/HK1/PIK3CB | 0.000115819 | KEGG |
| REACTOME_POLYMERASE_SWITCHING_ON_THE_C_STRAND_OF_THE_TELOMERE | -0.527545332 | 0.021158258 | POLA2/RFC2/PRIM2/POLD3/RFC5/POLD1/DSCC1/PCNA/PRIM1/CHTF18/RFC4/POLA1/RFC3 | 0.000117664 | REACTOME |
| WP_PPAR_SIGNALING_PATHWAY | 0.592192444 | 0.00401933 | CD36/SLC27A6/RXRG/FABP4/PLIN1/FABP6/FABP3/ACSL6/ACSL5/PPARG/ME1/SCD/MMP1/ACSL1/NR1H3/PLTP/CYP27A1/ACSL4/PCK2/FABP5 | 0.000117859 | WP |
| REACTOME_ACTIVATION_OF_THE_PRE_REPLICATIVE_COMPLEX | -0.752709862 | 1.33E-07 | MCM4/PRIM1/MCM3/MCM6/MCM7/POLE2/POLA1/POLE/DBF4/CDK2/MCM2/CDC6/CDT1/CDC7/MCM8/ORC6/CDC45/ORC1/MCM10 | 0.000118599 | REACTOME |
| WP_SYNAPTIC_VESICLE_PATHWAY | 0.779236932 | 8.30E-09 | UNC13C/SLC17A7/CACNA1B/SYT1/SYN2/SNAP25/CPLX3/CPLX2/SYN1/RAB3A/SLC32A1/SYP/DNM1/CPLX1/STX1B/STX1A/RIMS1/STXBP1/NSF/SLC22A3/VAMP2/UNC13A/DNM3 | 0.000123915 | WP |
| REACTOME_G_ALPHA_Z_SIGNALLING_EVENTS | 0.648046278 | 0.00106683 | PRKCG/GNG3/PRKCB/ADRA2C/ADRA2B/RGS4/RGS20/ADRA2A/ADCY1/PRKCQ/ADCY4/PRKCE/ADCY5/GNAI1/ADCY2/GNGT2/PRKCD/PRKCH/GNB5/GNAZ/GNAS/GNG11 | 0.000125255 | REACTOME |
| REACTOME_G0_AND_EARLY_G1 | -0.723185645 | 1.76E-05 | TFDP2/E2F5/PCNA/CCNE1/E2F1/LIN9/RBL1/MYC/CDK2/CDC6/CDK1/CCNA2/MYBL2/CDC25A/TOP2A | 0.00012806 | REACTOME |
| WP_ENDOTHELIN_PATHWAYS | 0.642650415 | 0.010278809 | ADRB1/NPY/NPY1R/CALM1/CNN1/ADRA1A/GNAI1/NOS3/EDN1/RIIAD1/GNA15/PLCB1/GNB5/MYLK/CALM2/GNAS/MAP2K1 | 0.000128108 | WP |
| REACTOME_SWITCHING_OF_ORIGINS_TO_A_POST_REPLICATIVE_STATE | -0.382330936 | 0.007223988 | MCM4/UBE2S/MCM3/CCNE1/MCM6/MCM7/CDK2/MCM2/CDC6/CDT1/MCM8/ORC6/CCNA2/ORC1/UBE2C | 0.000130315 | REACTOME |
| REACTOME_LAGGING_STRAND_SYNTHESIS | -0.659803369 | 0.00349003 | RFC1/RPA1/RPA3/POLA2/RFC2/PRIM2/POLD3/RFC5/POLD1/PCNA/FEN1/PRIM1/LIG1/RFC4/POLA1/RFC3/DNA2 | 0.000130727 | REACTOME |
| REACTOME_PCNA_DEPENDENT_LONG_PATCH_BASE_EXCISION_REPAIR | -0.603874055 | 0.01012486 | RFC1/RPA1/POLE3/RPA3/RFC2/APEX1/POLD3/RFC5/POLD1/PCNA/FEN1/LIG1/RFC4/POLE2/RFC3/POLE | 0.000131359 | REACTOME |
| REACTOME_DNA_REPLICATION_PRE_INITIATION | -0.50877082 | 9.08E-09 | MCM5/H4-16/H2AX/H2AC8/GMNN/H4C9/E2F3/MCM4/PRIM1/H3C10/MCM3/MCM6/MCM7/H2AC20/E2F1/POLE2/POLA1/H4C11/POLE/H2BC6/DBF4/CDK2/MCM2/CDC6/CDT1/H3C14/H3C15/CDC7/MCM8/ORC6/H2BC9/CDC45/E2F2/ORC1/MCM10 | 0.000131851 | REACTOME |
| REACTOME_POLO_LIKE_KINASE_MEDIATED_EVENTS | -0.873763276 | 5.12E-07 | LIN9/PKMYT1/WEE1/CCNB1/PLK1/CDC25C/MYBL2/CCNB2/FOXM1/CDC25A/CENPF | 0.000137593 | REACTOME |
| REACTOME_BASE_EXCISION_REPAIR | -0.493875477 | 0.000116844 | XRCC1/RFC1/RPA1/POLE3/RPA3/PARP1/H4C5/H2BC11/MUTYH/H2AZ1/H2AZ2/TDG/RFC2/APEX1/H2BC5/LIG3/H4C14/POLD3/H4-16/UNG/H2AX/H2AC8/H4C9/RFC5/POLD1/PCNA/FEN1/LIG1/RFC4/H2AC20/POLE2/H4C11/RFC3/POLE/H2BC6/NEIL3/H2BC9 | 0.000138292 | REACTOME |
| BIOCARTA_G2_PATHWAY | -0.591398248 | 0.007686644 | TP53/PRKDC/BRCA1/WEE1/CHEK1/CCNB1/PLK1/CDC25C/CDK1/CDC25A/MYT1 | 0.000143319 | BIOCARTA |
| REACTOME_ANTIGEN_ACTIVATES_B_CELL_RECEPTOR_BCR_LEADING_TO_GENERATION_OF_SECOND_MESSENGERS | 0.621201386 | 0.01743907 | CD22/ITPR1/CALM1/PIK3CD/DAPP1/PIK3AP1/VAV1/SYK/CD79A/BLNK/CD79B/BTK/PLCG2/PTPN6/LYN/GRB2/STIM1 | 0.000143787 | REACTOME |
| KEGG_ALDOSTERONE_REGULATED_SODIUM_REABSORPTION | 0.62160702 | 0.008513281 | PRKCG/PRKCB/IGF1/ATP1A3/ATP1B1/HSD11B1/NR3C2/SFN/PIK3CD/MAPK3/SLC9A3R2/HSD11B2/ATP1A2/PIK3R5/SGK1/FXYD2 | 0.000143789 | KEGG |
| HALLMARK_BILE_ACID_METABOLISM | 0.571030169 | 0.000293574 | ABCA9/SLCO1A2/CYP46A1/RXRG/BMP6/ABCG4/ABCA6/LCK/ALDH1A1/CH25H/TFCP2L1/AQP9/ABCA8/ABCA2/HSD17B6/CYP39A1/ACSL5/SULT1B1/LIPE/NR3C2/ABCA4/OPTN/DIO2/BCAR3/PAOX/CYP7B1/ACSL1/DHCR24/SOD1/IDI1/ALDH8A1/CYP27A1/SULT2B1 | 0.000151038 | HALLMARK |
| KEGG_MAPK_SIGNALING_PATHWAY | 0.530558855 | 2.88E-07 | PRKCG/CACNA1B/CACNA1I/CACNA2D3/PTPRR/RASGRF2/RASGRF1/FGF13/PTPN5/DUSP2/FGF7/NR4A1/PRKCB/CACNA1F/MAPK8IP2/TNF/ARRB1/HSPA2/CACNG8/PAK1/MAP3K8/FGF17/MEF2C/PPP3CB/RASGRP4/DUSP9/FGF22/FGFR2/FGF9/FGF18/DUSP8/RPS6KA5/MAPK10/PPP3R1/CACNB2/DUSP1/RASGRP3/MAPK3/CACNB4/PRKACB/MAPK13/IL1R1/CACNA2D2/CD14/FGF20/FGF14/CACNB1/MAP2K4/FGF1/MAPK9/RPS6KA2/HSPA1A/MAP3K5/RAC2/PDGFRB/CACNA1E/TGFBR2/MAP4K2/PDGFA/PPP3CA/MAPK8IP1/CACNA1D/ELK1/ARRB2/FGF12/CACNB3/NFATC2/MKNK1/MECOM/PLA2G2A/PDGFB/MRAS/IL1B/FOS/MAP4K1/DUSP3/RPS6KA4/DUSP7/NLK/RPS6KA1/JUND/CACNA1C/CACNA2D1/MAP2K1/CHP1/RASGRP2/GADD45B/MAPK8IP3/MAP3K3/CACNA1G/MAPK11/CACNA2D4/DDIT3/MAPT/RRAS/GRB2 | 0.00015228 | KEGG |
| WP_G1_TO_S_CELL_CYCLE_CONTROL | -0.595546897 | 2.88E-07 | TFDP2/TP53/PCNA/E2F3/MCM4/PRIM1/CDKN2C/MCM3/CCNE1/MCM6/MCM7/E2F1/POLE2/MYC/POLE/CDK2/MCM2/WEE1/ORC6/CCNB1/CDK6/CDK1/CDK4/CDC45/E2F2/ORC1/CDC25A/MYT1 | 0.000153484 | WP |
| WP_REGULATION_OF_SISTER_CHROMATID_SEPARATION_AT_THE_METAPHASEANAPHASE_TRANSITION | -0.792661101 | 0.000206158 | SMC3/STAG1/SMC1A/PTTG1/MAD2L1/CDC20/CENPE/BUB1B/BUB1/ESPL1 | 0.000153893 | WP |
| REACTOME_GLUTAMATE_NEUROTRANSMITTER_RELEASE_CYCLE | 0.864485538 | 1.56E-08 | SLC17A7/SYT1/GLS2/SNAP25/RAB3A/CPLX1/STX1A/RIMS1/STXBP1/SLC1A6/SLC1A7/TSPOAP1/PPFIA2/VAMP2/SLC1A2/PPFIA3/GLS | 0.000154286 | REACTOME |
| REACTOME_NOREPINEPHRINE_NEUROTRANSMITTER_RELEASE_CYCLE | 0.853825526 | 5.68E-05 | SYT1/SNAP25/RAB3A/CPLX1/STX1A/RIMS1/STXBP1/TSPOAP1/PPFIA2/VAMP2/PPFIA3/MAOA | 0.000163525 | REACTOME |
| BIOCARTA_NO1_PATHWAY | 0.612108816 | 0.048482021 | RYR2/CHRM1/PDE2A/CHRNA1/PRKAR1B/CALM1/CALM3/PRKACB/PRKAR2B/NOS3 | 0.000168984 | BIOCARTA |
| REACTOME_NA_CL_DEPENDENT_NEUROTRANSMITTER_TRANSPORTERS | 0.837058165 | 0.017568706 | SLC6A13/SLC6A12/SLC6A15/SLC6A1 | 0.000172495 | REACTOME |
| REACTOME_NEURONAL_SYSTEM | 0.666665093 | 7.11E-09 | PRKCG/SYT2/SLC6A13/HCN1/CAMK2A/GABRA5/GRIN1/GABRA1/NRGN/KCNC2/GABRB2/SLC17A7/GRM1/CACNA1B/SYT1/GLS2/SYN2/SNAP25/NEFL/DLGAP2/GAD2/TUBA8/GABRA4/KCNJ12/GRIN3A/KCNJ3/SYN1/CACNA2D3/GABRG2/KCNH5/KCNA4/GNG3/RAB3A/RASGRF2/RASGRF1/KCNS1/KCNJ6/KCNJ4/SHANK1/SLC32A1/TUBA4A/KCNH3/KCNK4/KCNN1/SYT7/CPLX1/AKAP5/KCNAB2/DLG2/CAMKK1/KCNA1/STX1A/SLC6A12/PRKCB/PANX2/RIMS1/GRIN2A/GABRA2/LRRC7/GABRG3/TUBB4A/KCNK1/DLGAP3/STXBP1/SLITRK4/ABCC8/KCNQ5/CAMK4/CAMK2B/KIF17/GRIN2B/KCNAB1/LRFN2/KCNG1/NRXN3/GLRA2/TUBB1/HCN2/GABRB3/KCNJ11/KCNJ9/CACNG8/SLC1A6/GABBR1/KCNJ5/NSF/KCNH1/EPB41L3/KCNB1/GABRB1/KCNQ3/CHRNA1/KCNMB4/PRKAR1B/CAMK2G/SLC1A7/GNAL/TSPOAP1/ADCY1/DLGAP1/PPFIA2/CALM1/ADCY4/EPB41L1/VAMP2/DLG4/KCNC1/SYT9/KCNK9/ADCY5/SLC1A2/PPFIA3/CAMKK2/KCNG2/KCNC3/SLITRK1/KCNMA1/KCNJ15/APBA1/KCNC4/GNAI1/LIN7B/SHANK2/SLC6A1/GLS/GRIP2/CACNB2/ADCY2/MAPK3/CACNB4/GRIN2C/PRKACB/GNGT2/NPTN/GLUL/CACNA2D2/GABBR2/PRKAR2B/MAOA/GAD1/KCNA5/NAAA/CACNB1/SYN3/GLRB/KCNQ1/ALDH2/BEGAIN/RPS6KA2 | 0.000172677 | REACTOME |
| HALLMARK_ESTROGEN_RESPONSE_LATE | 0.442101557 | 0.009845584 | CCN5/TPSAB1/PGR/DUSP2/CLIC3/CXCL12/ST6GALNAC2/CALCR/LLGL2/NBL1/RAPGEFL1/KLF4/LAMC2/OLFM1/NPY1R/TPD52L1/BATF/LSR/DNAJC12/PRLR/SEMA3B/TPBG/EGR3/SFN/IGFBP4/HPRT1/ITPK1/MAPK13/PTGER3/CACNA2D2/PRKAR2B/FRK/ZFP36/TMPRSS3/IGSF1/ADD3/CYP26B1/RPS6KA2/IMPA2/PLAAT3/S100A9/PTGES/TFAP2C/SGK1/BLVRB/CCNA1/WFS1/FOXC1/HSPB8/ISG20/JAK2/TRIM29/PTPN6/CKB/CXCL14/FOS/SULT2B1/PPIF/PAPSS2/OPN3/CPE/SNX10/PERP/FABP5/ETFB/PDCD4 | 0.000174276 | HALLMARK |
| KEGG_TYPE_I_DIABETES_MELLITUS | 0.726564825 | 9.72E-05 | GAD2/CD28/TNF/PTPRN/HLA-DPB1/HLA-DQA1/HLA-B/HLA-DQB1/GZMB/CD80/PRF1/HLA-DPA1/GAD1/HLA-E/HLA-DMA/HLA-F/HLA-DRB1/HLA-DRA/HLA-DRB5/PTPRN2/HLA-DMB/HLA-C/IL1B/HLA-DOA/CD86/CPE | 0.000174835 | KEGG |
| BIOCARTA_NO2IL12_PATHWAY | 0.805389663 | 0.002017218 | STAT4/IL12RB2/CD3E/CXCR3/CD247/CD3D/CD2/CD3G/IL12RB1/CD4/JAK2/CCR5 | 0.00018226 | BIOCARTA |
| REACTOME_GOLGI_CISTERNAE_PERICENTRIOLAR_STACK_REORGANIZATION | -0.632652824 | 0.041268526 | CCNB1/PLK1/CDK1/CCNB2 | 0.000183773 | REACTOME |
| REACTOME_TRANSMISSION_ACROSS_CHEMICAL_SYNAPSES | 0.687643511 | 7.11E-09 | PRKCG/SLC6A13/CAMK2A/GABRA5/GRIN1/GABRA1/NRGN/GABRB2/SLC17A7/CACNA1B/SYT1/GLS2/SYN2/SNAP25/NEFL/GAD2/TUBA8/GABRA4/KCNJ12/GRIN3A/KCNJ3/SYN1/CACNA2D3/GABRG2/GNG3/RAB3A/RASGRF2/RASGRF1/KCNJ6/KCNJ4/SLC32A1/TUBA4A/CPLX1/AKAP5/DLG2/CAMKK1/STX1A/SLC6A12/PRKCB/RIMS1/GRIN2A/GABRA2/LRRC7/GABRG3/TUBB4A/STXBP1/CAMK4/CAMK2B/KIF17/GRIN2B/GLRA2/TUBB1/GABRB3/KCNJ9/CACNG8/SLC1A6/GABBR1/KCNJ5/NSF/GABRB1/CHRNA1/PRKAR1B/CAMK2G/SLC1A7/GNAL/TSPOAP1/ADCY1/PPFIA2/CALM1/ADCY4/EPB41L1/VAMP2/DLG4/ADCY5/SLC1A2/PPFIA3/CAMKK2/KCNJ15/APBA1/GNAI1/LIN7B/SLC6A1/GLS/GRIP2/CACNB2/ADCY2/MAPK3/CACNB4/GRIN2C/PRKACB/GNGT2/NPTN/GLUL/CACNA2D2/GABBR2/PRKAR2B/MAOA/GAD1/NAAA/CACNB1/SYN3/GLRB | 0.000191643 | REACTOME |
| BIOCARTA_BARD1_PATHWAY | -0.831171706 | 0.009029842 | FANCG/FANCE/BRCA1/BARD1/FANCA/FANCC/FANCD2 | 0.000191755 | BIOCARTA |
| WP_HEMATOPOIETIC_STEM_CELL_DIFFERENTIATION | 0.62445976 | 0.002475109 | NFE2/IL6/CBFA2T3/FOSB/ITGA2B/ABO/THRB/TRAF3IP3/LYL1/FLI1/IKZF1/VAV1/NCKAP1L/GATA2/CD34/RHOH/IRF5/TRIM29/NFATC2/SPI1/CIITA/IL1B/FOS | 0.000193918 | WP |
| REACTOME_COMPLEMENT_CASCADE | 0.612416394 | 0.007175297 | C7/CR1/GZMM/FCN1/IGHG4/CFH/C5AR1/CFD/C5AR2/CD55/C2/C1QC/C1QA/CFP/C1QB | 0.00019493 | REACTOME |
| WP_DEVELOPMENT_AND_HETEROGENEITY_OF_THE_ILC_FAMILY | 0.656406818 | 0.037683132 | EOMES/TNF/IL6/GFI1/TBX21/IL15/ZBTB16/IL1B/IL18/IL7/IL23A | 0.000194973 | WP |
| REACTOME_ERYTHROCYTES_TAKE_UP_CARBON_DIOXIDE_AND_RELEASE_OXYGEN | 0.782906714 | 0.023772025 | HBB/HBA2/CA4/HBA1 | 0.000195022 | REACTOME |
| REACTOME_ROS_AND_RNS_PRODUCTION_IN_PHAGOCYTES | 0.610643999 | 0.024048694 | ATP6V1G2/MPO/NCF4/ATP6V1H/ATP6V1E1/NCF2/ATP6V1B2/NCF1/NOS3/ATP6V1A/RAC2/CYBA/ATP6V0A1/ATP6V0C/CYBB/ATP6V1C1/TCIRG1/ATP6V0D1/ATP6V1D/ATP6V0B | 0.000197965 | REACTOME |
| PID_AURORA_B_PATHWAY | -0.705299152 | 2.35E-07 | INCENP/AURKC/SMC2/NCAPD2/AURKA/KLHL13/RACGAP1/NDC80/KIF20A/NCAPG/CDCA8/AURKB/BIRC5/KIF2C/SMC4/KIF23/CENPA/SGO1/BUB1/NCAPH | 0.000207765 | PID |
| PID_IL8_CXCR1_PATHWAY | 0.725874525 | 0.000395733 | PRKCG/DNM1/PRKCB/CXCR1/ARRB1/GNA14/PRKCE/PLD1/HCK/PIK3R6/ARRB2/PLCB2/GNA15/PLCB1/GRK2/CXCL8/LYN/FGR | 0.000208833 | PID |
| REACTOME_APC_CDC20_MEDIATED_DEGRADATION_OF_NEK2A | -0.533893355 | 0.01846082 | UBE2S/MAD2L1/CDC20/BUB1B/NEK2/UBE2C | 0.000209549 | REACTOME |
| REACTOME_APC_C_MEDIATED_DEGRADATION_OF_CELL_CYCLE_PROTEINS | -0.448211417 | 0.000295173 | PTTG1/AURKA/FBXO5/CDK2/CCNB1/PLK1/MAD2L1/CDC20/CDK1/AURKB/CCNA2/BUB1B/NEK2/UBE2C | 0.000211323 | REACTOME |
| REACTOME_HDMS_DEMETHYLATE_HISTONES | -0.558303674 | 0.005309531 | KDM6A/JMJD6/H3C6/KDM3B/H4C5/KDM5C/KDM6B/H4C14/PHF2/KDM3A/PHF8/H4-16/KDM4A/H4C9/KDM1A/KDM4D/H3C10/KDM4B/KDM5A/KDM5B/H4C11/H3C14/H3C15 | 0.000222732 | REACTOME |
| KEGG_DNA_REPLICATION | -0.64807678 | 5.97E-05 | POLA2/RFC2/PRIM2/POLD3/MCM5/RNASEH2A/RFC5/POLD1/PCNA/FEN1/MCM4/PRIM1/MCM3/LIG1/MCM6/RFC4/MCM7/POLE2/POLA1/RFC3/DNA2/POLE/MCM2 | 0.000229137 | KEGG |
| REACTOME_ION_TRANSPORT_BY_P_TYPE_ATPASES | 0.660379343 | 0.000114362 | CAMK2A/ATP2B3/ATP8A2/PLN/CAMK2B/FXYD7/ATP8A1/ATP1A3/ATP1B1/ATP2B2/ATP10A/CAMK2G/CALM1/FXYD1/ATP2A3/ATP1A2/FXYD2/ATP8B4/ATP2B1/SLN/ATP8B1/ATP2C2 | 0.000230711 | REACTOME |
| REACTOME_REDUCTION_OF_CYTOSOLIC_CA_LEVELS | 0.718540935 | 0.045684924 | SLC8A2/ATP2B3/ATP2B2/CALM1/ATP2A3/ATP2B1 | 0.0002324 | REACTOME |
| BIOCARTA_DSP_PATHWAY | 0.787568295 | 0.029584962 | DUSP2/DUSP9/DUSP8/DUSP1/MAPK3 | 0.000235416 | BIOCARTA |
| WP_SUDDEN_INFANT_DEATH_SYNDROME_SIDS_SUSCEPTIBILITY_PATHWAYS | 0.47544704 | 0.006906888 | GRIN1/GABRA1/HTR2A/RYR2/SNAP25/SST/SCN3B/VIPR1/TPPP/IL10/PLP1/TNF/IL6/SSTR1/TAC1/ATP1A3/SSTR2/TF/YWHAH/PRKAR1B/MEF2C/VAMP2/THRB/TSPYL1/PRKACB/SNTA1/PRKAR2B/MAOA/IL1RN/GPD1L/KCNQ1/CREM/GATA2/DEAF1/RUNX3/SCN4B/CHRNA7/CEBPB/SLC25A4/PPARGC1A/IL1B/CHRFAM7A | 0.000236071 | WP |
| REACTOME_LEISHMANIA_INFECTION | 0.561683326 | 4.74E-08 | DRD1/VIP/GPR83/RXFP1/GNG3/GPR150/VIPR1/IL10/PTGER2/CRHR1/PTH2R/ADRB1/IL6/CALCR/IGHG4/HMOX1/HRH2/ITPR1/PRKAR1B/GPBAR1/CD247/ADCY1/PTH1R/CALM1/HTR7/ADCY4/RAMP3/ADCY5/CD163/MEFV/GNAI1/PLK2/DPEP2/POMC/CD3G/HCK/ADCY2/MAPK3/PRKACB/GNGT2/WIPF3/PRKAR2B/FCGR3A/VAV1/ELMO1/CYFIP2/SYK/PYCARD/ARPC1B/ADRB2/NCKAP1L/WASF1/CYBA/NLRP3/FCGR2A/BTK/C3AR1/PLCG2/GIPR/RHBDF2/CYSLTR1/FCGR1A/GPR84/GNB5/PTGER4/CYSLTR2/GPR27/IL1B/RAMP2/P2RX4/GNAZ/IL18/WASF3/GNAS/GNG11/LYN/CASP1/NOXA1/FGR/WAS/MYO5A | 0.000240222 | REACTOME |
| REACTOME_SYNTHESIS_OF_IP3_AND_IP4_IN_THE_CYTOSOL | 0.666467564 | 0.010369307 | PLCH2/ITPKA/PLCH1/CALM1/INPP5J/ITPK1/PLCB4/SYNJ1/PLCG2/PLCB2/INPP5D/PLCB1/PLD4/PTEN | 0.000246467 | REACTOME |
| REACTOME_SIGNALING_BY_RETINOIC_ACID | 0.559224801 | 0.047983921 | RXRG/CRABP1/PDK4/ALDH1A2/ALDH1A1/CRABP2/DHRS9/AKR1C3/CYP26B1/ALDH1A3/ALDH8A1/DHRS3/PDK2/FABP5 | 0.000258623 | REACTOME |
| REACTOME_DNA_DAMAGE_TELOMERE_STRESS_INDUCED_SENESCENCE | -0.491609049 | 0.001094728 | H4C5/H2BC11/H2AZ1/H2AZ2/CABIN1/CCNE2/EP400/H2BC5/H4C14/H1-0/H4-16/H2AX/H2AC8/H4C9/HMGA1/TP53/CCNE1/H2AC20/H4C11/H2BC6/CDK2/H2BC9/CCNA2/LMNB1/HMGA2 | 0.00025886 | REACTOME |
| REACTOME_ORC1_REMOVAL_FROM_CHROMATIN | -0.405919282 | 0.008414118 | MCM4/MCM3/MCM6/MCM7/CDK2/MCM2/CDC6/CDT1/MCM8/ORC6/CCNA2/ORC1 | 0.000264046 | REACTOME |
| WP_MIRNAS_INVOLVED_IN_DNA_DAMAGE_RESPONSE | -0.667036982 | 0.012885361 | RAD52/H2AX/TP53/CCNE1/E2F1/MYC/CDK6/CDC25A | 0.000268004 | WP |
| REACTOME_REPRODUCTION | -0.42523069 | 0.000482829 | SUN1/SMC3/CATSPERG/H4-16/REC8/DIDO1/H2AX/H2AC8/H4C9/STAG1/TOP3A/PSMC3IP/H3C10/SMC1A/BRCA1/RBBP8/H2AC20/SYCP3/H4C11/RAD51/H2BC6/BLM/MSH5/SYNE2/CDK2/H3C14/H3C15/MND1/H2BC9/BRCA2/CDK4/LMNB1 | 0.000280666 | REACTOME |
| REACTOME_INTEGRATION_OF_ENERGY_METABOLISM | 0.634747076 | 4.35E-07 | KCNC2/SNAP25/GNG3/CD36/RAPGEF4/SYT5/AKAP5/STX1A/ADRA2C/STXBP1/ABCC8/KCNJ11/ADRA2A/KCNB1/GNA14/ITPR1/PRKAR1B/ADCY1/ADCY4/VAMP2/RAPGEF3/ADCY5/KCNG2/GNAI1/CHRM3/CACNB2/ADCY2/PRKACB/GNGT2/CACNA2D2/MLXIPL/PRKAR2B/PRKAG2/CACNA1E/CACNA1D/CACNB3/PLCB2/GNA15/PLCB1/GNB5/CYSLTR2/ACSL4/GNAS/GNG11/CACNA1C | 0.000284358 | REACTOME |
| REACTOME_RNA_POLYMERASE_I_PROMOTER_ESCAPE | -0.445472912 | 0.008414118 | GTF2H3/ERCC3/TAF1D/TBP/UBTF/POLR2H/MNAT1/H3C6/TAF1B/H4C5/H2BC11/H2AZ1/H2AZ2/H2BC5/H4C14/GTF2H4/POLR1C/GTF2H2/RRN3/CBX3/H4-16/TAF1A/H2AX/H2AC8/H4C9/POLR1A/H3C10/POLR1B/H2AC20/H4C11/H2BC6/H3C14/H3C15/H2BC9 | 0.00029119 | REACTOME |
| REACTOME_DEPOSITION_OF_NEW_CENPA_CONTAINING_NUCLEOSOMES_AT_THE_CENTROMERE | -0.634368752 | 3.14E-07 | RBBP7/H4C5/H2BC11/H2AZ1/H2AZ2/MIS18BP1/RBBP4/SMARCA5/H2BC5/ITGB3BP/H4C14/CENPQ/RSF1/MIS18A/H4-16/H2AX/H2AC8/CENPP/H4C9/CENPL/OIP5/CENPN/H2AC20/CENPU/CENPO/CENPH/H4C11/H2BC6/CENPM/CENPK/H2BC9/CENPA/CENPI/HJURP/KNL1 | 0.000293182 | REACTOME |
| WP_MYOMETRIAL_RELAXATION_AND_CONTRACTION_PATHWAYS | 0.593514666 | 6.35E-07 | PRKCG/CAMK2A/RYR2/RXFP1/GNG3/CRHR1/PRKCB/RGS7/ARRB1/RGS4/IL6/CAMK2B/PRKCZ/RGS20/YWHAH/ITPR1/PRKAR1B/CAMK2G/LPAR1/ADCY1/CALM1/PRKCQ/ADCY4/GUCY1A1/RAMP3/RGS14/CNN1/PRKCE/CALM3/DGKZ/ADCY5/RGS5/SFN/IGFBP4/RGS2/RGS10/RGS18/ADCY2/PRKACB/GSTO1/PRKAR2B/MYL4/PRKCD/RGS1/ATP2A3/ETS2/NOS3/RGS11/RYR1/PRKCH/PLCG2/ARRB2/CACNB3/GNB5/ACTA2/IL1B/FOS/CALM2/RAMP2/GNAS/GNG11 | 0.000293718 | WP |
| REACTOME_B_WICH_COMPLEX_POSITIVELY_REGULATES_RRNA_EXPRESSION | -0.460427804 | 0.004515932 | GSK3B/POLR2H/DDX21/H3C6/TAF1B/H4C5/H2BC11/H2AZ1/MYBBP1A/H2AZ2/SF3B1/SMARCA5/EP300/H2BC5/H4C14/POLR1C/DEK/H4-16/TAF1A/H2AX/H2AC8/H4C9/BAZ1B/POLR1A/H3C10/POLR1B/H2AC20/H4C11/H2BC6/H3C14/H3C15/H2BC9 | 0.000297544 | REACTOME |
| WP_MAPK_SIGNALING_PATHWAY | 0.538675066 | 2.49E-07 | PRKCG/CACNA1B/CACNA1I/CACNA2D3/PTPRR/RASGRF2/RASGRF1/FGF13/PTPN5/DUSP2/FGF7/NR4A1/CACNA1F/MAPK8IP2/TNF/ARRB1/HSPA2/CACNG8/PAK1/MAP3K8/FGF17/MEF2C/PPP3CB/RASGRP4/DUSP9/FGF22/FGFR2/FGF9/FGF18/DUSP8/RPS6KA5/MAPK10/PPP3R1/CACNB2/DUSP1/RASGRP3/MAPK3/CACNB4/PRKACB/PLA2G4C/MAPK13/IL1R1/CACNA2D2/CD14/FGF20/PRKCD/FGF14/CACNB1/MAP2K4/FGF1/MAPK9/HSPA1A/MAP3K5/RAC2/PDGFRB/CACNA1E/TGFBR2/MAP4K2/PDGFA/PPP3CA/MAPK8IP1/CACNA1D/ELK1/ARRB2/FGF12/CACNB3/MKNK1/PDGFB/MRAS/IL1B/FOS/MAP4K1/DUSP3/RPS6KA4/DUSP7/NLK/JUND/CACNA1C/CACNA2D1/MAP2K1 | 0.000306074 | WP |
| REACTOME_DNA_STRAND_ELONGATION | -0.728207386 | 7.73E-07 | PRIM2/POLD3/MCM5/RFC5/POLD1/PCNA/GINS3/FEN1/MCM4/PRIM1/MCM3/LIG1/MCM6/RFC4/MCM7/POLA1/RFC3/DNA2/GINS2/GINS1/GINS4/MCM2/MCM8/CDC45 | 0.000314576 | REACTOME |
| WP_PKCGAMMA_CALCIUM_SIGNALING_PATHWAY_IN_ATAXIA | 0.633999753 | 0.0468974 | PRKCG/GRM1/ATP2B2/GNA14/ITPR1/PLCB4/PLCB2/GNA15/PLCB1 | 0.00032652 | WP |
| KEGG_PPAR_SIGNALING_PATHWAY | 0.584881104 | 0.00609515 | CD36/SLC27A6/RXRG/FABP4/PLIN1/FABP6/FABP3/ACSL6/ACSL5/PPARG/ME1/SCD/MMP1/ACSL1/NR1H3/PLTP/CYP27A1/ACSL4/PCK2/FABP5 | 0.00033245 | KEGG |
| REACTOME_FCGR3A_MEDIATED_IL10_SYNTHESIS | 0.578753978 | 0.020779943 | IL10/IGHG4/ITPR1/PRKAR1B/CD247/ADCY1/CALM1/ADCY4/ADCY5/CD3G/HCK/ADCY2/PRKACB/PRKAR2B/FCGR3A/SYK/FCGR2A/PLCG2/FCGR1A | 0.000332784 | REACTOME |
| REACTOME_PROCESSIVE_SYNTHESIS_ON_THE_LAGGING_STRAND | -0.644285107 | 0.021953141 | RPA1/RPA3/POLA2/PRIM2/POLD3/POLD1/PCNA/FEN1/PRIM1/LIG1/POLA1/DNA2 | 0.00033308 | REACTOME |
| WP_VITAMIN_DSENSITIVE_CALCIUM_SIGNALING_IN_DEPRESSION | 0.586968373 | 0.030643571 | GRIN1/ATP2B3/CHRM1/GRIN2A/GRIN2B/CALB1/ATP2B2/KCNQ3/ITPR1/GRIN2C/GRM5/KCNQ2/ATP2B1/CYP27A1/CACNA1C/GPX4/SLC8A1 | 0.000334526 | WP |
| REACTOME_BIOLOGICAL_OXIDATIONS | 0.491323035 | 0.004047172 | SULT4A1/CYP46A1/SULT1C2/FMO2/CYP1B1/AOC3/ALDH1A1/GSTM5/SULT1A1/CYP39A1/ACY3/CYP4F3/AKR7A3/SULT1B1/DPEP2/GSTO2/POMC/CYP2E1/CYP2J2/GSTO1/EPHX1/MAOA/HPGDS/SULT1A4/CYP26B1/PTGIS/ALDH2/PAOX/NAT2/CYP7B1/CYP2C8/ARNT2/TBXAS1/MGST3/CYP24A1/CYP27A1/SULT2B1/PAPSS2/ACSM5/GSTM3/TPST2/NQO2/CYP21A2/CYP2S1/MGST2/SULT1A3/NAT1/AKR1A1/AIP/TPMT/MAT2B/OPLAH/GGT5/CYP4F11/CYP4V2/GCLC/GSTM4/FMO3/ACY1/PDCD6-AHRR/GGT1/GCLM/CYP11A1/AHR/MGST1/ABHD14B/FDX2/CNDP2 | 0.000349078 | REACTOME |
| REACTOME_PHOSPHOLIPASE_C_MEDIATED_CASCADE_FGFR2 | 0.824978761 | 0.007999071 | FGF7/FGF17/FGF22/FGFR2/FGF9/FGF18/FGF20/FGF1 | 0.000361905 | REACTOME |
| BIOCARTA_RB_PATHWAY | -0.759885145 | 0.002133254 | CDK2/WEE1/CHEK1/CDC25C/CDK1/CDK4/CDC25A/MYT1 | 0.000364844 | BIOCARTA |
| WP_CILIOPATHIES | -0.290880486 | 0.016208176 | DRC1/TMEM107/TBC1D32/IFT52/KIZ/ANKS3/NPHP1/CILK1/RPGRIP1L/CEP78/INPP5E/TMEM237/DNAAF2/EFHC1/TMEM67/DDX59/DYNC2H1/DNAAF4/USP9X/GAS8/INTU/BBS10/ANKS6/FAM161A/NEK8/DNAH6/WDR19/CSPP1/NPHP3/ADCY6/CCDC28B/DNAAF5/RP2/CEP290/MAK/TCTN1/RSPH9/DNAI1/INVS/C2CD3/KIAA0753/IFT140/ARL13B/B9D1/SPAG1/CEP164/CCDC40/KIAA0586/TCTN2/CPLANE1/MKS1/TTC21B/SMO/IFT81/DNAJB13/WDR35/DNAAF3/EVC2/CCDC65/ALMS1/POC1A/OFD1/EVC/KIF7/HYDIN/GPR161/RP1L1/GLI2/PLK4/NEK2/CENPF/DNAH11 | 0.000366461 | WP |
| REACTOME_TRANSPORT_OF_SMALL_MOLECULES | 0.510455551 | 7.11E-09 | SLC6A13/CAMK2A/SLC12A5/HBB/SLC8A2/SLC17A7/CASQ2/ATP2B3/RYR2/SLC38A4/SLC7A10/ABCA9/HBA2/SLC30A3/SLCO1A2/GNG3/SLC26A9/ANO3/DMTN/TRPV6/SLC32A1/SLC24A4/CLCA4/CA4/SLC27A6/SLC4A10/ATP8A2/GPIHBP1/SLC6A12/CYGB/SLC13A5/PLN/ANO9/ABCG4/TRPM6/SLC5A11/ASIC2/ABCA6/SLC24A2/ATP6V1G2/HBA1/SLC6A15/SLC45A3/CAMK2B/SLC14A1/FXYD7/ATP8A1/ATP1A3/NIPAL4/ATP1B1/ATP2B2/TF/ANO4/KCNJ11/SLC9A2/SGK2/HMOX1/SLC40A1/SLC1A6/AQP3/PCSK6/SLC13A3/ATP10A/SLC5A4/MICU3/LIPC/SLC26A4/AQP9/FTL/ABCB1/ABCA8/NIPAL3/ABCA2/SLC22A3/SLC25A18/PRKAR1B/CAMK2G/SLC1A7/STEAP2/LCN12/ADCY1/ABCA10/SLC7A8/SLC7A2/CALM1/ADCY4/LSR/LIPA/LDLRAP1/ADCY5/SLC1A2/BEST1/NIPAL2/ABCG2/APOD/NIPA1/CLCN4/FTH1/ABCA4/SLC6A1/MLKL/TRPM3/SLCO2B1/SLC16A8/CLIC2/NCEH1/ATP6V1H/FXYD1/SLC2A12/CFTR/ADCY2/SLC30A10/PRKACB/SLC15A3/SLC22A15/GNGT2/WNK2/MCOLN2/ANO1/TRPM2/ATP6V1E1/SLC25A22/SLCO3A1/PRKAR2B/CYB5R2/SLC43A1/FKBP1B/RIPK3/EMB/ADD3/SLCO2A1/ATP2A3/ATP6V1B2/ANO2/ATP1A2/ATP6V1A/NALCN/RYR1/TRPV2/SGK1/UNC80/MYLIP/ATP6V0A1/TRPM8/ATP6V0C/SLC2A4/SLC9A6/SLC7A7/SLC12A2/SLCO1C1/SLC5A10/SGK3/NR1H3/FXYD2/ATP6V1C1/SLC38A3/ATP8B4/CASQ1/SLC29A3/TRPV4/ATP2B1/LRRC8C/SLC2A9/HMOX2/SLC25A4/RAB11FIP2/GNB5/ANKH/SLC9A9/SLN/ATP8B1/ATP2C2/PLTP/LRRC8B/TRPM4/AP2A2/ABCA7/NPC2/GNAS/GNG11/TCIRG1/STOM/ATP6V0D1/APOE/SLC22A17/ARL2/APOC1/TTYH2/ATP6V1D/TSC22D3/ATP6V0B/SLC39A10/PSMB10/ATP10B/WWP1/SLC2A11/PSMB9/RHCG/TPCN1/SLC8A1 | 0.000367936 | REACTOME |
| BIOCARTA_G1_PATHWAY | -0.518275909 | 0.015362149 | TP53/CCNE1/DHFR/E2F1/TGFB2/CDK2/CDK6/CDK1/CDK4/CDC25A | 0.000392002 | BIOCARTA |
| KEGG_ARGININE_AND_PROLINE_METABOLISM | 0.543776761 | 0.036466362 | GLS2/CKMT1A/CKMT1B/GOT1/GLUD2/PRODH/GLS/AGMAT/GLUL/OAT/MAOA/ALDH2/CKMT2/NOS3/AZIN2/GLUD1/CKB/SAT1/ALDH4A1/NAGS/LAP3/NOS2/ALDH9A1/ACY1/GAMT/ARG2/NOS1/ALDH7A1 | 0.000415099 | KEGG |
| REACTOME_TNFS_BIND_THEIR_PHYSIOLOGICAL_RECEPTORS | 0.703734304 | 0.003911188 | TNFSF18/CD27/TNFSF9/EDA/TNFRSF8/TNFRSF6B/TNFRSF4/TNFRSF25/TNFSF8/TNFRSF14/EDARADD/TNFRSF1B/TNFSF15/TNFSF13/TNFSF13B/TNFRSF18 | 0.000421009 | REACTOME |
| WP_VITAMIN_D_RECEPTOR_PATHWAY | 0.464754915 | 0.013612562 | CREG2/ADRA1B/TRPV6/SOSTDC1/SULT1C2/STEAP4/KL/BMP6/KLK6/KLF4/ABCB1/CTLA4/BGLAP/CLMN/HLA-DQA1/PRKCQ/MX2/PNOC/CDKN2B/SEMA3B/CDKN2D/CD200/SLC37A2/THBD/CD14/CRACR2B/CD40/LGALS9/ADRB2/TPM1/IRF4/S100A9/ITGAM/SERPINB1/IRF8/CEBPA/SLC2A4/IRF5/HLA-DRB1/LRRC25/NFATC2/ATP2B1/PTGER4/PRDM1/ATP2C2/CYP24A1/GXYLT2/EFNA5/TNFAIP3/NINJ1/CEACAM1/CASP5/S100A8/STAM/SLC8A1/COL13A1/CYP2S1/ALOX5 | 0.00043737 | WP |
| BIOCARTA_NOS1_PATHWAY | 0.750710013 | 0.001873946 | GRIN1/PRKCB/GRIN2A/GRIN2B/PRKAR1B/PPP3CB/CALM1/DLG4/CALM3/GRIN2C/PRKACB/PRKAR2B/PPP3CA | 0.000457637 | BIOCARTA |
| WP_INTEGRATED_CANCER_PATHWAY | -0.482361208 | 0.007611049 | POU2F1/MSH6/TP53/BRCA1/BARD1/E2F1/MYC/BLM/CDK2/CHEK1/PLK1/CDK1/CDK4/CDC25A | 0.0004712 | WP |
| REACTOME_RECOGNITION_OF_DNA_DAMAGE_BY_PCNA_CONTAINING_REPLICATION_COMPLEX | -0.49167267 | 0.024638159 | RFC1/RPA1/POLE3/RPA3/CUL4B/RFC2/POLD3/RFC5/POLD1/USP1/PCNA/RFC4/RAD18/POLE2/RFC3/POLE/DTL | 0.000473404 | REACTOME |
| REACTOME_PHOSPHORYLATION_OF_EMI1 | -0.91251652 | 0.001094728 | FBXO5/CCNB1/PLK1/CDC20/CDK1 | 0.000483653 | REACTOME |
| REACTOME_POLYMERASE_SWITCHING | -0.679775658 | 0.018267204 | POLA2/RFC2/PRIM2/POLD3/RFC5/POLD1/PCNA/PRIM1/RFC4/POLA1/RFC3 | 0.000485852 | REACTOME |
| WP_GASTRIC_CANCER_NETWORK_1 | -0.663306963 | 0.000116874 | MCM4/KIF20B/LIN9/AURKA/NUP107/NOTCH1/ECT2/TPX2/E2F7/MYBL2/UBE2C/KIF15/CENPF/TOP2A | 0.000490513 | WP |
| KEGG_CYTOKINE_CYTOKINE_RECEPTOR_INTERACTION | 0.586869966 | 7.11E-09 | CCL19/CCL8/CCL3/CCL3L3/IL10/CCL4L2/TNFSF18/CXCL11/CCL4/CXCL12/TNF/CXCR1/CD27/IL6/LTB/PPBP/IL12RB2/OSM/CXCR2/CCL5/TNFSF9/CXCL9/CXCL10/CXCL1/CXCR3/VEGFD/CXCR6/IL18RAP/LEPR/MPL/EDA/CCR6/TNFRSF8/FLT3/IL2RB/CXCL6/IL18R1/TNFRSF6B/PRLR/TNFSF10/CCL28/CSF2RB/CCR1/CCR4/IL10RA/LTBR/CXCL3/INHBA/IL12RB1/IL11RA/IL1R1/CCR2/TNFRSF4/IL15/TNFRSF25/CCR7/IL3RA/CD40/CSF2RA/IL7R/CNTF/TNFSF8/IL15RA/IL20RA/IL2RG/PDGFRB/TGFBR2/TNFRSF10A/PDGFA/TNFRSF11A/TNFRSF14/CXCL2/CSF3R/TNFSF12/CXCL5/CSF1R/IL2RA/PDGFB/IL4R/CCR5/PLEKHO2/IL1B/CXCL14/CXCL8/CX3CR1/IL18/ACVRL1/NGFR/TNFRSF1B/IL7/IL23A/TNFSF15/TNFSF13/TNFSF13B/IFNGR1 | 0.000491848 | KEGG |
| KEGG_HEMATOPOIETIC_CELL_LINEAGE | 0.665958372 | 2.15E-06 | CD36/CR1/TNF/IL6/CD8B/CD22/MME/CD3E/ITGA2B/CD5/CD55/CD3D/FLT3/CD2/CD7/CD37/CD3G/CD8A/IL11RA/ANPEP/IL1R1/CD14/IL3RA/CSF2RA/IL7R/CD38/ITGAM/CD4/CD34/CSF3R/CD33/CSF1R/FCGR1A/HLA-DRB1/IL2RA/HLA-DRA/HLA-DRB5/IL4R/THPO/IL1B/CD1D/CD1C/IL7 | 0.000494035 | KEGG |
| KEGG_CARDIAC_MUSCLE_CONTRACTION | 0.53540184 | 0.027435044 | RYR2/CACNA2D3/CACNA1F/ATP1A3/ATP1B1/CACNG8/MYL3/COX7A1/COX4I2/CACNB2/CACNB4/CACNA2D2/CACNB1/ATP1A2/TPM1/CACNA1D/SLC9A6/CACNB3/FXYD2/COX4I1/UQCRB/CACNA1C/CACNA2D1/SLC8A1/CACNA2D4/COX7A2L/TPM3/COX5B/COX7A2/COX5A/ACTC1/UQCRC2/COX6A1/UQCRH | 0.000528692 | KEGG |
| KEGG_CELL_ADHESION_MOLECULES_CAMS | 0.580880124 | 1.08E-05 | CNTNAP2/SELE/MAG/SIGLEC1/CD28/CD8B/NRXN3/CD22/NEGR1/CNTN2/ITGA8/PDCD1/CTLA4/HLA-DPB1/CLDN11/HLA-DQA1/CD6/CLDN5/CD2/HLA-B/SELPLG/HLA-DQB1/CLDN10/L1CAM/CD80/ICAM2/CD8A/HLA-DPA1/CLDN4/ITGB7/ICOSLG/CD40/HLA-E/MADCAM1/ITGAM/CD4/CLDN9/ITGAL/CD226/CD34/HLA-DMA/HLA-F/CDH15/HLA-DRB1/HLA-DRA/PTPRM/CNTNAP1/SELL/HLA-DRB5/CDH5/PTPRC/HLA-DMB/NCAM2/CD274/SDC2/HLA-C/HLA-DOA/CD86/NRXN1/PECAM1 | 0.000531746 | KEGG |
| REACTOME_SEROTONIN_NEUROTRANSMITTER_RELEASE_CYCLE | 0.863935403 | 1.24E-05 | SYT1/SYN2/SNAP25/SYN1/RAB3A/CPLX1/STX1A/RIMS1/STXBP1/TSPOAP1/PPFIA2/VAMP2/PPFIA3 | 0.000556817 | REACTOME |
| WP_KISSPEPTINKISSPEPTIN_RECEPTOR_SYSTEM_IN_THE_OVARY | 0.592781158 | 0.023207975 | PRKCG/STAR/PRKCB/ARRB1/MMP9/PRKCQ/PRKCE/PIK3CD/MAPK3/PRKCD/PLCB4/PRKCH/ARRB2/PLCB2/PLCB1/MAP2K1/PIK3CB | 0.000557199 | WP |
| WP_CORTICOTROPINRELEASING_HORMONE_SIGNALING_PATHWAY | 0.564795764 | 0.001211276 | CAMK2A/CRHBP/STAR/NR4A1/CRHR1/PRKCB/ARRB1/NR4A2/FOSB/PRKCQ/RAPGEF3/GRK3/GNAI1/POMC/MAPK3/PRKCD/MAPK9/MAP3K5/NOS3/GNAO1/ELK1/PLCG2/ARRB2/GNB5/TLR4/FOS/CXCL8/GNAZ/IL18/GNAS/JUND/MAP2K1 | 0.000561717 | WP |
| REACTOME_RHO_GTPASES_ACTIVATE_NADPH_OXIDASES | 0.702429531 | 0.006499151 | PRKCB/PRKCZ/NCF4/MAPK3/PRKCD/NCF2/NCF1/RAC2/S100A9/CYBA/CYBB/NOXA1/S100A8/MAPK11/PIN1 | 0.000562201 | REACTOME |
| REACTOME_G2_M_DNA_REPLICATION_CHECKPOINT | -0.9592804 | 0.000393265 | WEE1/CCNB1/CDK1/CCNB2 | 0.000605337 | REACTOME |
| REACTOME_ACETYLCHOLINE_NEUROTRANSMITTER_RELEASE_CYCLE | 0.886471706 | 1.52E-05 | SYT1/SNAP25/RAB3A/CPLX1/STX1A/RIMS1/STXBP1/TSPOAP1/PPFIA2/VAMP2/PPFIA3 | 0.00062164 | REACTOME |
| REACTOME_NEUROTOXICITY_OF_CLOSTRIDIUM_TOXINS | 0.904490998 | 0.00017439 | SYT2/SV2B/SYT1/SNAP25/SV2C/STX1B/STX1A/VAMP1/VAMP2 | 0.000629661 | REACTOME |
| REACTOME_RMTS_METHYLATE_HISTONE_ARGININES | -0.400843416 | 0.036646717 | SMARCE1/H3C6/ACTL6A/SMARCB1/RBBP7/H4C5/H2AZ1/H2AZ2/PRMT6/PBRM1/H4C14/DNMT3A/PRMT5/WDR5/SMARCA4/ARID1A/H4-16/H2AX/H2AC8/H4C9/ARID2/SMARCC1/SMARCD1/H3C10/H2AC20/H2AC11/H4C11/H3C14/H3C15/CDK4 | 0.000646243 | REACTOME |
| WP_NRF2_PATHWAY | 0.49324235 | 0.011587727 | SLC6A13/CES4A/SLC6A17/FGF13/SLC5A11/SLC6A15/SLC39A12/HMOX1/SLC5A4/GSTM5/FTL/FTH1/ME1/SLC6A1/SLC2A12/SOD3/NQO1/HSPA1A/TGFBR2/BLVRB/SLC2A4/SLC5A10/GPX3/MGST3/SLC2A9/PDGFB/SLC2A5/TGFA/CBR1/GSTM3/SLC39A10/SLC2A11/SLC2A6/MGST2/HSP90AA1/PRDX1/SLC39A4/SLC2A3/GCLC/TGFB1/G6PD/GSTM4/SQSTM1/GGT1/GCLM/EPHA2 | 0.000666219 | WP |
| REACTOME_INTERACTION_BETWEEN_L1_AND_ANKYRINS | 0.639577165 | 0.011104988 | SCN2B/SCN3B/SPTB/SCN2A/SCN1B/KCNQ3/ANK3/SPTBN2/SCN8A/L1CAM/SPTBN4/SCN4B/KCNQ2/ANK2/SCN7A | 0.000667486 | REACTOME |
| WP_REGULATORY_CIRCUITS_OF_THE_STAT3_SIGNALING_PATHWAY | 0.522869629 | 0.016809205 | PTPRT/DUSP2/PRKCB/IL12RB2/MPL/DEPTOR/IL2RB/CSF2RB/MAPK10/IL10RA/JAK3/MAPK3/IL11RA/MAPK13/F2RL2/MAPK9/IL3RA/CSF2RA/IL7R/IL15RA/IL20RA/IL2RG/PDGFRB/CSF3R/JAK2/IL2RA/PTPRC/F2RL3/IL27RA | 0.000668344 | WP |
| BIOCARTA_P53_PATHWAY | -0.685807942 | 0.008715453 | TP53/PCNA/CCNE1/E2F1/CDK2/CDK4 | 0.000674052 | BIOCARTA |
| KEGG_FC_EPSILON_RI_SIGNALING_PATHWAY | 0.522477974 | 0.021465401 | PRKCB/TNF/PRKCE/MAPK10/PIK3CD/MAPK3/MAPK13/PRKCD/MAP2K4/VAV1/MAPK9/SYK/RAC2/PIK3R5/FCER1G/BTK/PLCG2/INPP5D/PLA2G2A/LAT/FCER1A/LYN/LCP2/MAP2K1/PIK3CB | 0.000675322 | KEGG |
| KEGG_AMYOTROPHIC_LATERAL_SCLEROSIS_ALS | 0.604192123 | 0.003019565 | NEFM/GRIN1/NEFL/PRPH2/GRIN2A/NEFH/TNF/GRIN2B/PPP3CB/SLC1A2/PPP3R1/GRIN2C/MAPK13/MAP3K5/PPP3CA/SOD1/GPX1/TNFRSF1B/CASP1/CHP1 | 0.000685679 | KEGG |
| REACTOME_PI_3K_CASCADE_FGFR2 | 0.71468146 | 0.029584962 | FGF7/FGF17/FGF22/FGFR2/FGF9/FGF18/FGF20/FGF1 | 0.000693325 | REACTOME |
| REACTOME_HDACS_DEACETYLATE_HISTONES | -0.449583506 | 0.004817051 | GATAD2A/H3C6/TBL1X/HDAC8/RBBP7/H4C5/RCOR1/H2BC11/MTA1/MTA2/NCOR2/RBBP4/HDAC2/CHD4/H2BC5/GATAD2B/H4C14/REST/H4-16/H2AC8/H4C9/KDM1A/H3C10/H2AC20/H2AC11/H4C11/H2BC6/H3C14/H3C15/H2BC9 | 0.000698477 | REACTOME |
| WP_COVID19_ADVERSE_OUTCOME_PATHWAY | 0.784107784 | 0.014755316 | CCL3/IL10/TNF/IL6/CXCL10/IL2RA/IL1B/CXCL8/IL7/CCL2 | 0.000759826 | WP |
| KEGG_PHOSPHATIDYLINOSITOL_SIGNALING_SYSTEM | 0.623103186 | 3.12E-05 | PRKCG/PRKCB/CDS1/PIP5K1B/ITPKA/DGKE/ITPR1/PIP4K2A/SYNJ2/CALM1/DGKB/INPP5J/CALM3/DGKZ/PI4KA/PIK3CD/ITPK1/INPP5A/PLCB4/IMPA2/PIK3R5/SYNJ1/PLCG2/PLCB2/INPP5D/PLCB1/INPP4B/CALM2/PTEN/PIK3CB/PIP5K1C/DGKG/DGKQ | 0.000779419 | KEGG |
| BIOCARTA_IL12_PATHWAY | 0.760123384 | 0.002201418 | STAT4/IL12RB2/CD3E/CXCR3/CD247/CD3D/IL18R1/CD3G/IL12RB1/JAK2/CCR5/IL18 | 0.000782694 | BIOCARTA |
| REACTOME_NEUROTRANSMITTER_RECEPTORS_AND_POSTSYNAPTIC_SIGNAL_TRANSMISSION | 0.646350003 | 7.11E-09 | PRKCG/CAMK2A/GABRA5/GRIN1/GABRA1/NRGN/GABRB2/NEFL/TUBA8/GABRA4/KCNJ12/GRIN3A/KCNJ3/GABRG2/GNG3/RASGRF2/RASGRF1/KCNJ6/KCNJ4/TUBA4A/AKAP5/DLG2/CAMKK1/PRKCB/GRIN2A/GABRA2/LRRC7/GABRG3/TUBB4A/CAMK4/CAMK2B/KIF17/GRIN2B/GLRA2/TUBB1/GABRB3/KCNJ9/CACNG8/GABBR1/KCNJ5/NSF/GABRB1/CHRNA1/PRKAR1B/CAMK2G/GNAL/ADCY1/CALM1/ADCY4/EPB41L1/DLG4/ADCY5/CAMKK2/KCNJ15/APBA1/GNAI1/LIN7B/GRIP2/ADCY2/MAPK3/GRIN2C/PRKACB/GNGT2/NPTN/GABBR2/PRKAR2B | 0.00081166 | REACTOME |
| REACTOME_IMMUNOREGULATORY_INTERACTIONS_BETWEEN_A_LYMPHOID_AND_A_NON_LYMPHOID_CELL | 0.683234542 | 7.11E-09 | LILRB5/ICAM5/SIGLEC1/SH2D1A/CD8B/CD200R1/CD22/SLAMF6/KLRB1/IFITM1/KLRD1/COL1A2/COL3A1/LILRB2/CD3E/COL17A1/COL1A1/CD96/CD247/SIGLEC8/LILRA6/JAML/CD3D/SIGLEC11/HLA-B/LILRA4/CRTAM/PILRA/CD3G/ICAM2/CD200/SIGLEC7/CD8A/COLEC12/TYROBP/SLAMF7/ITGB7/LILRA5/LAIR1/FCGR3A/KLRK1/CD40/HCST/SIGLEC12/LILRA2/HLA-E/OSCAR/CD300LB/MADCAM1/CD300LF/ITGAL/CD226/TREML1/CD34/CD300E/HLA-F/CD33/FCGR1A/LILRB4/LILRA1/SELL/LILRB1/SIGLEC10/CD300C/SIGLEC9/TREM2/HLA-C/LILRB3/CD1D | 0.000867449 | REACTOME |
| KEGG_ASTHMA | 0.73783638 | 0.005630312 | IL10/TNF/HLA-DPB1/HLA-DQA1/HLA-DQB1/HLA-DPA1/CD40/FCER1G/HLA-DMA/HLA-DRB1/HLA-DRA/HLA-DRB5/HLA-DMB/HLA-DOA/FCER1A | 0.000878516 | KEGG |
| BIOCARTA_DC_PATHWAY | 0.800046458 | 0.022109505 | IL10/CD5/CD2/CD7/ANPEP/CD40/CD33/TLR7 | 0.000944078 | BIOCARTA |
| REACTOME_INOSITOL_PHOSPHATE_METABOLISM | 0.595451594 | 0.006749786 | PLCH2/IP6K3/ITPKA/MTMR7/PLCH1/CALM1/INPP5J/ITPK1/INPP5A/PLCB4/IMPA2/SYNJ1/PLCG2/PLCB2/INPP5D/PLCB1/PPIP5K1/PLD4/INPP4B/PTEN/NUDT11/NUDT3/IMPA1/INPP4A/INPP1 | 0.0009519 | REACTOME |
| REACTOME_DOPAMINE_NEUROTRANSMITTER_RELEASE_CYCLE | 0.831475382 | 5.38E-06 | SYT1/SYN2/SNAP25/SYN1/RAB3A/CPLX1/STX1A/RIMS1/STXBP1/TSPOAP1/PPFIA2/VAMP2/PPFIA3/APBA1/LIN7B/SYN3 | 0.00097611 | REACTOME |
| REACTOME_DISEASES_OF_PROGRAMMED_CELL_DEATH | -0.472265557 | 0.000300935 | RBBP7/H4C5/H2BC11/H2AZ1/H2AZ2/RBBP4/POLA2/H2BC5/H4C14/DNMT3A/PRIM2/CDC25B/H4-16/H2AX/H2AC8/BCL2L11/H4C9/TP53/SUZ12/DNMT1/PRIM1/H3C10/H2AC20/DNMT3B/POLA1/H4C11/H2BC6/H3C14/H3C15/EZH2/H2BC9/CDC25C/LMNB1/CDC25A | 0.001018265 | REACTOME |
| REACTOME_FCERI_MEDIATED_CA_2_MOBILIZATION | 0.663145591 | 0.003845954 | GRAP2/ITPR1/PPP3CB/CALM1/PPP3R1/ITK/TEC/VAV1/SYK/FCER1G/PPP3CA/BTK/PLCG2/NFATC2/LAT/FCER1A/LYN/LCP2 | 0.001046145 | REACTOME |
| BIOCARTA_CTLA4_PATHWAY | 0.769908511 | 0.001472045 | CD28/LCK/CD3E/CTLA4/CD247/CD3D/CD80/CD3G/ITK/ICOSLG/HLA-DRB1/HLA-DRA/HLA-DRB5/CD86 | 0.001052114 | BIOCARTA |
| REACTOME_OPIOID_SIGNALLING | 0.653656806 | 3.78E-07 | PRKCG/CAMK2A/PDYN/GNG3/PDE1A/PDE1B/PPP1R1B/CAMKK1/CAMK4/CAMK2B/GNA14/ITPR1/PRKAR1B/PPP3CB/CAMK2G/GNAL/ADCY1/CALM1/ADCY4/ADCY5/CAMKK2/PPP3R1/GNAI1/POMC/ADCY2/PRKACB/GNGT2/PRKAR2B/PRKCD/PLCB4/PPP3CA/PLCB2/PDE1C/GNA15/PLCB1/GNB5/PDE4A/GRK2/NBEA/GNG11 | 0.001063568 | REACTOME |
| REACTOME_MEIOTIC_RECOMBINATION | -0.549671821 | 0.000123027 | RPA1/RPA3/H3C6/MLH3/H4C5/RAD51C/H2BC11/H2AZ1/H2AZ2/DMC1/H2BC5/H4C14/H4-16/H2AX/H2AC8/H4C9/TOP3A/PSMC3IP/H3C10/BRCA1/RBBP8/H2AC20/H4C11/RAD51/H2BC6/BLM/MSH5/CDK2/H3C14/H3C15/MND1/H2BC9/BRCA2/CDK4 | 0.001072705 | REACTOME |
| REACTOME_PRC2_METHYLATES_HISTONES_AND_DNA | -0.590787676 | 0.00015619 | MTF2/RBBP7/H4C5/H2BC11/H2AZ1/H2AZ2/RBBP4/AEBP2/H2BC5/H4C14/DNMT3A/H4-16/PHF19/JARID2/H2AX/H2AC8/H4C9/SUZ12/DNMT1/H3C10/H2AC20/DNMT3B/H4C11/H2BC6/H3C14/H3C15/EZH2/H2BC9 | 0.001081704 | REACTOME |
| WP_PATHOGENESIS_OF_SARSCOV2_MEDIATED_BY_NSP9NSP10_COMPLEX | 0.762852461 | 0.004515932 | IL6/CD8B/LCK/ZAP70/CD3E/CD247/CD2/CD3G/CD8A/CD4/HLA-DRB1/HLA-DRA/HLA-DRB5/CXCL8 | 0.001081781 | WP |
| REACTOME_NEGATIVE_REGULATION_OF_NMDA_RECEPTOR_MEDIATED_NEURONAL_TRANSMISSION | 0.827224799 | 1.87E-05 | CAMK2A/GRIN1/NEFL/DLG2/GRIN2A/LRRC7/CAMK4/CAMK2B/GRIN2B/CAMK2G/CALM1/DLG4/GRIN2C | 0.001093314 | REACTOME |
| REACTOME_INHIBITION_OF_REPLICATION_INITIATION_OF_DAMAGED_DNA_BY_RB1_E2F1 | -0.636421889 | 0.040238571 | POLA2/PRIM2/TFDP2/PRIM1/PPP2R3B/E2F1/POLA1 | 0.001112419 | REACTOME |
| KEGG_FC_GAMMA_R_MEDIATED_PHAGOCYTOSIS | 0.556129607 | 0.000754397 | PRKCG/DNM1/PRKCB/AMPH/PIP5K1B/PAK1/PRKCE/DNM3/PLD1/HCK/PIK3CD/MAPK3/FCGR3A/PRKCD/DOCK2/VAV1/SYK/ARPC1B/NCF1/RAC2/WASF1/PIK3R5/FCGR2A/PLCG2/GSN/FCGR1A/INPP5D/PTPRC/PLPP2/LAT/WASF3/LYN/MAP2K1/PIK3CB/WAS/SPHK1/PIP5K1C | 0.001151233 | KEGG |
| REACTOME_ACTIVATION_OF_NIMA_KINASES_NEK9_NEK6_NEK7 | -0.799688924 | 0.015705048 | NEK6/CCNB1/PLK1/CDK1/CCNB2 | 0.001215699 | REACTOME |
| WP_SELECTIVE_EXPRESSION_OF_CHEMOKINE_RECEPTORS_DURING_TCELL_POLARIZATION | 0.694262771 | 0.012480496 | CCL3/CCL4/CD28/IL12RB2/CXCR3/IL18R1/CCR1/CCR4/IL12RB1/CCR2/CCR7/CD4/IL4R/CCR5 | 0.00123848 | WP |
| REACTOME_FORMATION_OF_THE_BETA_CATENIN_TCF_TRANSACTIVATING_COMPLEX | -0.467435216 | 0.001070334 | H4C5/LEO1/H2BC11/H2AZ1/H2AZ2/CREBBP/TLE2/BCL9L/TCF7L1/EP300/H2BC5/H4C14/MEN1/SMARCA4/H4-16/KMT2D/H2AX/H2AC8/H4C9/TRRAP/BCL9/TCF4/H3C10/H2AC20/PYGO1/H4C11/MYC/H2BC6/H3C14/H3C15/H2BC9/TERT | 0.001281232 | REACTOME |
| REACTOME_G_PROTEIN_MEDIATED_EVENTS | 0.675851226 | 3.57E-05 | PRKCG/CAMK2A/PDE1A/PDE1B/CAMKK1/CAMK4/CAMK2B/GNA14/ITPR1/PRKAR1B/CAMK2G/GNAL/ADCY1/CALM1/ADCY4/ADCY5/CAMKK2/GNAI1/ADCY2/PRKACB/PRKAR2B/PRKCD/PLCB4/PLCB2/PDE1C/GNA15/PLCB1/GRK2/NBEA | 0.001321669 | REACTOME |
| KEGG_CHEMOKINE_SIGNALING_PATHWAY | 0.606441314 | 7.11E-09 | GNG3/CCL19/CCL8/CCL3/CCL3L3/CCL4L2/PRKCB/CXCL11/CCL4/CXCL12/CXCR1/ARRB1/PPBP/CXCR2/PRKCZ/CCL5/CXCL9/CXCL10/CXCL1/CXCR3/PAK1/CXCR6/ADCY1/ADCY4/CCR6/CXCL6/ADCY5/GRK3/CCL28/CCR1/CCR4/GNAI1/JAK3/CXCL3/HCK/PIK3CD/ADCY2/MAPK3/PRKACB/ITK/GNGT2/CCR2/PRKCD/DOCK2/PLCB4/VAV1/CCR7/ELMO1/NCF1/RAC2/PIK3R5/ARRB2/CXCL2/CXCL5/JAK2/PTK2B/PLCB2/CCR5/PLCB1/GNB5/CXCL14/GRK2/CXCL8/CX3CR1/GNG11/LYN/MAP2K1/FGR/PIK3CB/WAS/RASGRP2/GRK5/CCL2 | 0.00134841 | KEGG |
| REACTOME_GLUCAGON_LIKE_PEPTIDE_1_GLP1_REGULATES_INSULIN_SECRETION | 0.626509779 | 0.006122514 | KCNC2/GNG3/RAPGEF4/AKAP5/KCNB1/ITPR1/PRKAR1B/RAPGEF3/ADCY5/KCNG2/PRKACB/GNGT2/PRKAR2B | 0.001382509 | REACTOME |
| BIOCARTA_TH1TH2_PATHWAY | 0.766440225 | 0.00467087 | CD28/IL12RB2/IL18R1/IL12RB1/CD40/HLA-DRB1/IL2RA/HLA-DRA/HLA-DRB5/IL4R/CD86/IL18/IFNGR1 | 0.001419585 | BIOCARTA |
| REACTOME_UNWINDING_OF_DNA | -0.848875096 | 7.19E-05 | GINS3/MCM4/MCM3/MCM6/MCM7/GINS2/GINS1/GINS4/MCM2/MCM8/CDC45 | 0.001447353 | REACTOME |
| WP_OVERVIEW_OF_PROINFLAMMATORY_AND_PROFIBROTIC_MEDIATORS | 0.651431599 | 0.00016603 | CCL19/CCL8/CCL3/CCL3L3/IL10/CCL4L2/CXCL11/CCL4/CXCL12/TNF/IL6/PPBP/OSM/CCL5/CXCL9/MMP9/CXCL10/CXCL1/CXCL6/CCL28/CXCL3/EBI3/IL15/IL1RN/CNTF/MMP1/CXCL2/CXCL5/IL1B/CXCL14/CXCL8/IL18/IL7/IL23A/TNFSF13/TNFSF13B/CCL2/IL17D/CXCL16 | 0.001556682 | WP |
| BIOCARTA_CK1_PATHWAY | 0.732627066 | 0.015879306 | DRD1/GRM1/PPP1R1B/PRKAR1B/PRKACB/CDK5R1/PRKAR2B/PPP3CA/PLCB1 | 0.001562487 | BIOCARTA |
| PID_IL12_2PATHWAY | 0.721112763 | 1.00E-07 | CCL3/STAT4/EOMES/CCL4/CD8B/LCK/IL12RB2/CD3E/GZMA/TBX21/IL18RAP/CD247/PPP3CB/CD3D/IL2RB/IL18R1/PPP3R1/STAT6/GZMB/CD3G/CD8A/IL12RB1/IL1R1/IL2RG/CD4/PPP3CA/SOCS1/JAK2/IL2RA/HLA-DRA/CCR5/IL1B/FOS/IL18/B2M/HLX/GADD45B | 0.001582272 | PID |
| REACTOME_LGI_ADAM_INTERACTIONS | 0.777403082 | 0.010397753 | LGI3/ADAM11/STX1B/STX1A/LGI1/CACNG8/DLG4/LGI4 | 0.001589038 | REACTOME |
| PID_IL8_CXCR2_PATHWAY | 0.683141594 | 0.001245853 | PRKCG/DNM1/PRKCB/ARRB1/CXCR2/GNA14/HCK/DOCK2/ELMO1/PIK3R6/RAC2/ARRB2/PLCB2/GNA15/PLCB1/CXCL8/LYN/FGR | 0.001595149 | PID |
| BIOCARTA_TCRA_PATHWAY | 0.811532062 | 0.002844633 | LCK/ZAP70/CD3E/CD247/CD3D/CD3G/CD4/HLA-DRB1/HLA-DRA/HLA-DRB5/PTPRC | 0.001596192 | BIOCARTA |
| BIOCARTA_NKT_PATHWAY | 0.692439328 | 0.01873364 | CCL3/CCL4/CD28/IL12RB2/CXCR3/IL18R1/CCR1/CCR4/IL12RB1/CCR7/CD4/IL4R/CCR5 | 0.001634584 | BIOCARTA |
| HALLMARK_KRAS_SIGNALING_UP | 0.488100323 | 0.000417829 | RBP4/SNAP25/RELN/PTPRR/USH1C/GPNMB/SNAP91/ALDH1A2/PPBP/NAP1L2/MAP7/MMP9/CXCL10/KLF4/ADAMDEC1/SCN1B/CFH/EPB41L3/ABCB1/KIF5C/CMKLR1/GYPC/GUCY1A1/FUCA1/HSD11B1/FGF9/ADGRA2/SEMA3B/APOD/ITGBL1/CD37/IL10RA/CTSS/INHBA/ANO1/CLEC4A/IKZF1/DOCK2/TLR8/CSF2RA/LCP1/IL7R/IL2RG/FCER1G/IRF8/TSPAN7/F13A1/C3AR1/ALDH1A3/MALL/PCSK1N/TMEM176B/PRDM1/ANKH/RABGAP1L/IL1B/ENG/MAFB/PECAM1/MAP4K1/MMP11/TMEM176A/LAPTM5/GALNT3/GNG11/MMD/CPE/TNFRSF1B/EREG/TNFAIP3/CAB39L/ST6GAL1/IGF2/USP12/SCG5/LAT2/CCSER2/MTMR10/PTGS2/TSPAN1/PDCD1LG2/ITGB2/ADGRL4/LY96/PLVAP/BTBD3/JUP/MPZL2/PCP4/CXCR4/SPARCL1/PLAUR/CFB | 0.001709548 | HALLMARK |
| REACTOME_TRANSPORT_OF_BILE_SALTS_AND_ORGANIC_ACIDS_METAL_IONS_AND_AMINE_COMPOUNDS | 0.563183273 | 0.008188794 | SLC6A13/SLC30A3/SLC6A12/SLC13A5/SLC5A11/SLC6A15/SLC14A1/SLC40A1/SLC13A3/SLC22A3/SLC6A1/SLC16A8/SLC30A10/SLC22A15/EMB | 0.001724611 | REACTOME |
| KEGG_LONG_TERM_POTENTIATION | 0.655267017 | 9.85E-06 | PRKCG/CAMK2A/GRIN1/PPP1R1A/GRM1/PRKCB/GRIN2A/CAMK4/CAMK2B/GRIN2B/ITPR1/PPP3CB/CAMK2G/ADCY1/CALM1/RAPGEF3/CALM3/PPP3R1/MAPK3/GRIN2C/PRKACB/PLCB4/RPS6KA2/PPP3CA/GRM5/PLCB2/PLCB1/CALM2/RPS6KA1/CACNA1C/MAP2K1/CHP1 | 0.001756575 | KEGG |
| WP_CONTROL_OF_IMMUNE_TOLERANCE_BY_VASOACTIVE_INTESTINAL_PEPTIDE | 0.788982818 | 0.029149 | VIP/IL10/CD28/CTLA4/CD80 | 0.001786252 | WP |
| BIOCARTA_CDC25_PATHWAY | -0.767234359 | 0.019885676 | WEE1/CHEK1/CDC25C/CDK1/CDC25A/MYT1 | 0.001879382 | BIOCARTA |
| PID_FOXM1_PATHWAY | -0.675575548 | 2.07E-06 | GAS1/MYC/CDK2/CCNB1/BRCA2/PLK1/CDK1/CDK4/AURKB/BIRC5/CCNA2/CCNB2/NEK2/CENPA/FOXM1/CENPF/ONECUT1 | 0.001881672 | PID |
| REACTOME_FRUCTOSE_CATABOLISM | 0.859788842 | 0.047577068 | ALDH1A1/ALDOB | 0.001974003 | REACTOME |
| REACTOME_DISINHIBITION_OF_SNARE_FORMATION | 0.866208726 | 0.042251245 | PRKCG/PRKCB | 0.001992949 | REACTOME |
| PID_TXA2PATHWAY | 0.627437294 | 0.000750453 | PRKCG/SELE/DNM1/PRKCB/LCK/TGM2/PRKCZ/GNA14/PRKCQ/PRKCE/GRK3/HCK/PRKCD/SYK/PIK3R6/NOS3/PRKCH/PIK3R5/ARRB2/PLCB2/GNA15/GNB5/GRK2/LYN/FGR/MAPK11/ICAM1/PTGIR | 0.001998689 | PID |
| REACTOME_CONDENSATION_OF_PROPHASE_CHROMOSOMES | -0.607501498 | 7.19E-05 | H3C6/KMT5A/SET/H4C5/H2BC11/H2AZ1/H2AZ2/MCPH1/H2BC5/H4C14/PHF8/H4-16/H2AX/H2AC8/H4C9/NCAPD3/H3C10/H2AC20/SMC2/H4C11/H2BC6/NCAPG2/H3C14/H3C15/CCNB1/H2BC9/PLK1/CDK1/SMC4 | 0.002115197 | REACTOME |
| REACTOME_MEIOTIC_SYNAPSIS | -0.418694661 | 0.021074291 | ATR/H4C5/H2BC11/H2AZ1/H2AZ2/H2BC5/H4C14/STAG2/SUN1/SMC3/H4-16/REC8/DIDO1/H2AX/H2AC8/H4C9/STAG1/SMC1A/BRCA1/H2AC20/SYCP3/H4C11/H2BC6/SYNE2/H2BC9/LMNB1 | 0.002119748 | REACTOME |
| REACTOME_LONG_TERM_POTENTIATION | 0.783400844 | 7.46E-05 | CAMK2A/GRIN1/NRGN/NEFL/DLG2/GRIN2A/LRRC7/CAMK2B/GRIN2B/CAMK2G/CALM1/DLG4/GRIN2C | 0.002188056 | REACTOME |
| BIOCARTA_WNT_LRP6_PATHWAY | 0.903967065 | 0.030050807 | DKK2/KREMEN2 | 0.002237637 | BIOCARTA |
| REACTOME_PD_1_SIGNALING | 0.75647007 | 0.001434827 | LCK/CD3E/PDCD1/HLA-DPB1/CD247/HLA-DQA1/CD3D/HLA-DQB1/CD3G/HLA-DPA1/CD4/HLA-DRB1/PTPN6/HLA-DRA/HLA-DRB5/CD274 | 0.00248299 | REACTOME |
| REACTOME_DEFECTIVE_CHST6_CAUSES_MCDC1 | 0.839378962 | 0.016368969 | OGN/OMD/LUM | 0.002567622 | REACTOME |
| REACTOME_TOXICITY_OF_BOTULINUM_TOXIN_TYPE_D_BOTD | 0.864584085 | 0.044286621 | SV2B/SV2C/VAMP1/VAMP2 | 0.002645684 | REACTOME |
| WP_G_PROTEIN_SIGNALING_PATHWAYS | 0.593721156 | 7.46E-05 | PRKCG/KCNJ3/GNG3/PDE1A/PDE1B/AKAP5/PRKCB/PRKCZ/GNA14/ITPR1/PRKAR1B/GNAL/ADCY1/CALM1/PRKCQ/ADCY4/PRKCE/ADCY5/GNAI1/ADCY2/PRKACB/GNGT2/PRKAR2B/PRKCD/GNAO1/PRKCH/PPP3CA/PDE1C/GNA15/GNB5/PDE4A/CALM2/GNAZ/GNAS/GNG11/PDE7B/PALM2AKAP2/PDE8B/AKAP6 | 0.002841291 | WP |
| WP_SPLICING_FACTOR_NOVA_REGULATED_SYNAPTIC_PROTEINS | 0.623741344 | 0.005896396 | GRIN1/GABRG2/KCNJ6/NCDN/GRIN2B/PRKCZ/EPB41L3/ANK3/CAMK2G/EPB41L1/KCNMA1/GABBR2/PLCB4/MAPK9/STXBP2/RAP1GAP/KCNQ2/ATP2B1 | 0.002853474 | WP |
| KEGG_ADIPOCYTOKINE_SIGNALING_PATHWAY | 0.57693493 | 0.005256911 | CD36/CAMKK1/RXRG/TNF/NPY/LEPR/PRKCQ/ACSL6/ACSL5/CAMKK2/MAPK10/POMC/MAPK9/PRKAG2/ACSL1/SLC2A4/JAK2/PPARGC1A/ACSL4/PCK2/TNFRSF1B | 0.002887341 | KEGG |
| WP_CANNABINOID_RECEPTOR_SIGNALING | 0.626924231 | 0.038801494 | FAAH/PRKAR1B/ADCY1/MAPK10/MAPK3/PRKACB/MAPK13/PRKAR2B/MAPK9/MAPK11/ADCY7/ADORA2A/MAPK8/PRKAR1A/AHR/PRKACA/NAPEPLD/DAGLA/MAPK1 | 0.002888379 | WP |
| KEGG_TRYPTOPHAN_METABOLISM | 0.581975965 | 0.045262991 | CYP1B1/OGDHL/KMO/TDO2/IL4I1/INMT/MAOA/AOX1/HAAO/ALDH2/IDO1/KYNU/ECHS1 | 0.002894333 | KEGG |
| PID_IL27_PATHWAY | 0.68680335 | 0.016111307 | STAT4/TNF/IL6/IL12RB2/TBX21/IL12RB1/EBI3/JAK2/IL1B/IL27RA/IL18 | 0.002930011 | PID |
| PID_ALPHA_SYNUCLEIN_PATHWAY | 0.641331443 | 0.015772684 | SNCA/KLK6/LCK/UCHL1/PLD1/HCK/MAPK3/PRKCD/SYK/PRKN/PTK2B/PLCB2/LYN/FGR/GRK5/STUB1 | 0.002967602 | PID |
| KEGG_INTESTINAL_IMMUNE_NETWORK_FOR_IGA_PRODUCTION | 0.695863019 | 0.000295042 | IL10/CXCL12/CD28/IL6/HLA-DPB1/HLA-DQA1/CCL28/HLA-DQB1/LTBR/CD80/HLA-DPA1/ITGB7/IL15/ICOSLG/CD40/IL15RA/MADCAM1/HLA-DMA/HLA-DRB1/HLA-DRA/HLA-DRB5/HLA-DMB/HLA-DOA/CD86/TNFSF13/TNFSF13B | 0.002990931 | KEGG |
| PID_CD8_TCR_DOWNSTREAM_PATHWAY | 0.674095121 | 4.28E-05 | EGR4/STAT4/EOMES/PRKCB/TNF/CD8B/CD3E/CD247/PPP3CB/PRKCQ/CD3D/PRKCE/IL2RB/PPP3R1/GZMB/CD3G/PRF1/CD8A/MAPK3/TNFRSF4/MAPK9/IL2RG/PPP3CA/ELK1/NFATC2/IL2RA/FOS/B2M/MAP2K1 | 0.00305344 | PID |
| REACTOME_GENERATION_OF_SECOND_MESSENGER_MOLECULES | 0.699373288 | 0.00071883 | GRAP2/LCK/ZAP70/CD3E/PAK1/HLA-DPB1/CD247/HLA-DQA1/CD3D/HLA-DQB1/CD3G/HLA-DPA1/PAK3/ITK/CD4/PLCG2/HLA-DRB1/HLA-DRA/HLA-DRB5/FYB1/LAT/LCP2/WAS | 0.003061119 | REACTOME |
| BIOCARTA_CACAM_PATHWAY | 0.84032356 | 0.007166882 | CAMK1G/CAMKK1/CAMK4/CALM1/CALM3/CAMKK2 | 0.003171101 | BIOCARTA |
| BIOCARTA_IL5_PATHWAY | 0.829009734 | 0.03904385 | IL6/CD4/HLA-DRB1/HLA-DRA/HLA-DRB5/IL1B | 0.003228895 | BIOCARTA |
| BIOCARTA_TCR_PATHWAY | 0.589281925 | 0.01089435 | PRKCB/LCK/ZAP70/CD3E/CD247/PPP3CB/CALM1/CD3D/CALM3/CD3G/MAPK3/MAP2K4/VAV1/PPP3CA/ELK1/NFATC2/FOS/CALM2/LAT/MAP2K1 | 0.003329725 | BIOCARTA |
| HALLMARK_XENOBIOTIC_METABOLISM | 0.479868889 | 0.001881822 | RBP4/CD36/AKR1C2/SLC6A12/PTGDS/IGF1/PDK4/SPINT2/NDRG2/HMOX1/CDA/AQP9/F10/TDO2/GABARAPL1/HSD11B1/AKR1C3/VNN1/IGFBP4/HPRT1/CYP2E1/PINK1/MAN1A1/TTPA/FBLN1/CYP2J2/IL1R1/GSTO1/FAH/EPHX1/MAOA/GAD1/AOX1/CYFIP2/NQO1/MPP2/ALDH2/ETS2/RAP1GAP/PTGES/BLVRB/GCH1/IRF8/KYNU/ESR1/TMEM176B/CYP27A1/PC/PAPSS2/ABHD6/ACO2/APOE/CBR1/SLC46A3/NINJ1/PSMB10 | 0.003558453 | HALLMARK |
| KEGG_DILATED_CARDIOMYOPATHY | 0.52213881 | 0.008846002 | RYR2/CACNA2D3/CACNA1F/PLN/IGF1/TNF/ADRB1/CACNG8/ITGA8/MYL3/ITGA2B/ADCY1/ADCY4/ADCY5/SGCA/ITGA10/CACNB2/ADCY2/CACNB4/PRKACB/ITGB7/CACNA2D2/CACNB1/TPM1/CACNA1D/TTN/CACNB3/SGCD/DMD/GNAS/CACNA1C/ITGA9/CACNA2D1 | 0.003602504 | KEGG |
| KEGG_MELANOGENESIS | 0.503369064 | 0.017993867 | PRKCG/CAMK2A/WNT10B/PRKCB/WNT4/CAMK2B/WNT16/FZD10/CAMK2G/ADCY1/CALM1/ADCY4/CALM3/WNT2B/ADCY5/TCF7/GNAI1/POMC/ADCY2/MAPK3/PRKACB/PLCB4/GNAO1/EDN1/PLCB2/PLCB1/CALM2/MITF/GNAS/MAP2K1/WNT9B/WNT6/WNT10A/LEF1/FZD4/ADCY7/WNT5A/GNAQ/WNT5B | 0.003796975 | KEGG |
| REACTOME_ERCC6_CSB_AND_EHMT2_G9A_POSITIVELY_REGULATE_RRNA_EXPRESSION | -0.505611927 | 0.002351921 | GATAD2A/H3C6/RBBP7/H4C5/H2BC11/H2AZ1/H2AZ2/MTA1/MTA2/RBBP4/HDAC2/CHD4/H2BC5/TTF1/GATAD2B/H4C14/CBX3/H4-16/H2AX/H2AC8/H4C9/H3C10/H2AC20/H4C11/H2BC6/H3C14/H3C15/H2BC9 | 0.003850528 | REACTOME |
| REACTOME_UPTAKE_AND_ACTIONS_OF_BACTERIAL_TOXINS | 0.774054285 | 3.68E-05 | SYT2/SV2B/SYT1/SNAP25/SV2C/STX1B/STX1A/VAMP1/CALM1/VAMP2 | 0.003854319 | REACTOME |
| REACTOME_CA_DEPENDENT_EVENTS | 0.707244591 | 9.13E-05 | PRKCG/CAMK2A/PDE1A/PDE1B/CAMKK1/CAMK4/CAMK2B/PRKAR1B/CAMK2G/ADCY1/CALM1/ADCY4/ADCY5/CAMKK2/ADCY2/PRKACB/PRKAR2B/PRKCD | 0.003879732 | REACTOME |
| WP_TCELL_ACTIVATION_SARSCOV2 | 0.622430482 | 2.36E-05 | CCL19/GRAP2/STAT4/CD28/TNF/LCK/IL12RB2/ZAP70/CD3E/CTLA4/ITPR1/CD247/DEPTOR/PRKCQ/CD3D/IL18R1/CCL28/CARD11/CD80/CD3G/PIK3CD/MAPK3/IL12RB1/CD4/JAK2/HLA-DRB1/HLA-DRA/FOS/CD86/LAT/LCP2/PTEN/IL23A/MAP2K1 | 0.003962587 | WP |
| REACTOME_UNBLOCKING_OF_NMDA_RECEPTORS_GLUTAMATE_BINDING_AND_ACTIVATION | 0.800938848 | 9.08E-05 | CAMK2A/GRIN1/NEFL/DLG2/GRIN2A/LRRC7/CAMK2B/GRIN2B/CAMK2G/CALM1/DLG4/GRIN2C | 0.004059269 | REACTOME |
| PID_IL12_STAT4_PATHWAY | 0.720647785 | 0.000563356 | STAT4/CD28/CD3E/TBX21/IL18RAP/CD247/PPP3CB/CD3D/IL18R1/PPP3R1/CD80/CD3G/PRF1/MAPK9/CD4/PPP3CA/IL2RA/HLA-DRA/FOS/CD86/IL18 | 0.004275142 | PID |
| REACTOME_GPVI_MEDIATED_ACTIVATION_CASCADE | 0.595952281 | 0.029149 | LCK/MPIG6B/PRKCZ/COL1A2/COL1A1/VAV1/SYK/PIK3R6/RAC2/PIK3R5/FCER1G/PLCG2/PTPN6/RHOG/LAT/LYN/LCP2/PIK3CB | 0.0042943 | REACTOME |
| REACTOME_RAS_ACTIVATION_UPON_CA2_INFLUX_THROUGH_NMDA_RECEPTOR | 0.846574813 | 3.64E-06 | CAMK2A/GRIN1/NEFL/RASGRF2/RASGRF1/DLG2/LRRC7/CAMK2B/GRIN2B/CAMK2G/CALM1/DLG4 | 0.004416458 | REACTOME |
| REACTOME_RELAXIN_RECEPTORS | 0.994293284 | 0.048469198 | RXFP1 | 0.004583654 | REACTOME |
| WP_CHEMOKINE_SIGNALING_PATHWAY | 0.587216549 | 1.80E-07 | GNG3/CCL19/CCL3/PRKCB/CXCL11/CCL4/CXCL12/ARRB1/PPBP/CXCR2/PRKCZ/CCL5/CXCL9/CXCL10/CXCR3/PAK1/CXCR6/ADCY1/ADCY4/CCR6/ADCY5/CCL28/CCR1/CCR4/GNAI1/JAK3/CXCL3/HCK/PIK3CD/ADCY2/MAPK3/PRKACB/ITK/GNGT2/CCR2/PRKCD/DOCK2/PLCB4/VAV1/CCR7/ELMO1/NCF1/RAC2/PIK3R5/ARRB2/CXCL5/JAK2/PTK2B/PLCB2/PLCB1/GNB5/CXCL14/GRK2/CX3CR1/GNG11/LYN/MAP2K1/FGR/PIK3CB/WAS/RASGRP2/GRK5 | 0.004745415 | WP |
| REACTOME_DAG_AND_IP3_SIGNALING | 0.705870173 | 7.19E-05 | PRKCG/CAMK2A/PDE1A/PDE1B/CAMKK1/CAMK4/CAMK2B/ITPR1/PRKAR1B/CAMK2G/ADCY1/CALM1/ADCY4/PRKCE/ADCY5/CAMKK2/ADCY2/PRKACB/PRKAR2B/PRKCD | 0.004765451 | REACTOME |
| WP_MICROGLIA_PATHOGEN_PHAGOCYTOSIS_PATHWAY | 0.662906433 | 0.000687956 | NCF4/C1QC/C1QA/HCK/PIK3CD/SIGLEC7/TYROBP/C1QB/VAV1/NCF2/SYK/PIK3R6/ARPC1B/NCF1/NCKAP1L/RAC2/ITGAM/FCER1G/CYBA/PLCG2/CYBB/FCGR1A/PTPN6/TREM2/LAT/LYN/PIK3CB | 0.004929316 | WP |
| KEGG_AUTOIMMUNE_THYROID_DISEASE | 0.747267518 | 0.000110871 | IL10/CD28/CTLA4/HLA-DPB1/HLA-DQA1/HLA-B/HLA-DQB1/GZMB/CD80/PRF1/HLA-DPA1/CD40/HLA-E/HLA-DMA/HLA-F/HLA-DRB1/HLA-DRA/HLA-DRB5/HLA-DMB/HLA-C/HLA-DOA/CD86 | 0.004939161 | KEGG |
| REACTOME_INTERLEUKIN_RECEPTOR_SHC_SIGNALING | 0.637155131 | 0.043641688 | IL2RB/CSF2RB/JAK3/PIK3CD/IL3RA/CSF2RA/IL2RG/JAK2/PTPN6/IL2RA/INPP5D/PIK3CB/GRB2 | 0.005094541 | REACTOME |
| REACTOME_CARGO_RECOGNITION_FOR_CLATHRIN_MEDIATED_ENDOCYTOSIS | 0.544666991 | 0.001093535 | SYT2/SYT1/SH3GL3/KIAA0319/SH3GL2/REPS2/SNAP91/ARRB1/LRP2/TF/DAB2/SGIP1/VAMP2/FCHO1/CD3D/SYT9/LDLRAP1/GRK3/CD3G/CFTR/IL7R/ADRB2/VAMP8/CD4/ARRB2/AAK1/GRK2/TGFA/AP2A2/CLTB/ITSN1/STON2/EREG/AGTR1/EPS15/STAM/NECAP1/CLTA/GRB2/FZD4/WNT5A/AP2S1/AP2M1/ITSN2/UBB | 0.00511908 | REACTOME |
| REACTOME_ASSEMBLY_OF_THE_ORC_COMPLEX_AT_THE_ORIGIN_OF_REPLICATION | -0.561608041 | 0.001142342 | ORC2/H4C5/H2BC11/H2AZ1/H2AZ2/H2BC5/KPNB1/H4C14/H4-16/H2AX/H2AC8/H4C9/H3C10/H2AC20/H4C11/H2BC6/H3C14/H3C15/ORC6/H2BC9/ORC1 | 0.005218937 | REACTOME |
| BIOCARTA_IL17_PATHWAY | 0.793526159 | 0.005978013 | IL6/CD3E/CD247/CD3D/CD2/CD3G/CD8A/CD4/CD34/CXCL8 | 0.005415608 | BIOCARTA |
| REACTOME_TRP_CHANNELS | 0.659188507 | 0.035453649 | TRPV6/TRPM6/MLKL/TRPM3/MCOLN2/TRPM2/RIPK3/TRPV2/TRPM8/TRPV4/TRPM4 | 0.005448698 | REACTOME |
| REACTOME_GLYCEROPHOSPHOLIPID_BIOSYNTHESIS | 0.447843423 | 0.045262991 | CPNE6/PLA1A/ETNPPL/CPNE7/GPAT2/CDS1/PITPNM3/PHOSPHO1/PLAAT5/GPD1/PLD1/PLA2G4C/PLD3/GPD1L/PLBD1/PLAAT3/LPCAT4/LPIN3/MIGA1/PLA2G2A/PLD4/PLB1/PITPNM1/PLAAT4/GPAM/PLA2G15/DDHD2/LPCAT2/MGLL/PNPLA2/LPGAT1/MBOAT1/DGAT2/ACHE/PNPLA3/AGPAT4/STARD10/CDIPT/PLD2/HADHB/PCYT2 | 0.005491997 | REACTOME |
| REACTOME_NEUREXINS_AND_NEUROLIGINS | 0.687892291 | 1.10E-05 | SYT2/GRIN1/GRM1/SYT1/DLGAP2/SHANK1/SYT7/DLG2/STX1A/GRIN2A/DLGAP3/STXBP1/GRIN2B/NRXN3/EPB41L3/DLGAP1/EPB41L1/DLG4/SYT9/APBA1/LIN7B/SHANK2/GRIN2C | 0.005512268 | REACTOME |
| PID_RAC1_REG_PATHWAY | 0.559550821 | 0.038471346 | RASGRF2/RASGRF1/NGEF/CHN1/MCF2/CHN2/KALRN/DOCK2/VAV1/ELMO1/RAP1GDS1/ARHGAP9/ABR/DEF6/PREX2/TIAM2/ABI1/ARHGAP1/DOCK1 | 0.005831922 | PID |
| WP_NONGENOMIC_ACTIONS_OF_125_DIHYDROXYVITAMIN_D3 | 0.61165893 | 0.000162629 | PRKCG/CAMK2A/PRKCB/RXRG/TNF/IL6/CAMK2B/PRKCZ/CAMK2G/PRKCQ/PRKCE/RSAD2/OAS2/ISG15/MAPK3/MAPK13/PRKCD/PLCB4/MAPK9/CD40/TLR8/PRKCH/PLCG2/PLCB2/PLCB1/TLR2/CYP24A1/TLR4/CXCL8/IFI27L2/NOD2/IFI44L/CCL2/MAPK11 | 0.005927296 | WP |
| REACTOME_CREB1_PHOSPHORYLATION_THROUGH_NMDA_RECEPTOR_MEDIATED_ACTIVATION_OF_RAS_SIGNALING | 0.749769728 | 9.46E-05 | CAMK2A/GRIN1/NEFL/RASGRF2/RASGRF1/DLG2/LRRC7/CAMK2B/GRIN2B/CAMK2G/CALM1/DLG4/MAPK3/RPS6KA2 | 0.006228604 | REACTOME |
| KEGG_NATURAL_KILLER_CELL_MEDIATED_CYTOTOXICITY | 0.586294371 | 3.58E-05 | PRKCG/FCGR3B/PRKCB/TNF/SH2D1A/LCK/KLRD1/ZAP70/PAK1/CD247/PPP3CB/TNFSF10/HLA-B/PPP3R1/GZMB/ICAM2/PRF1/PIK3CD/MAPK3/TYROBP/CD48/FCGR3A/CD244/KLRK1/VAV1/HCST/SYK/HLA-E/RAC2/PIK3R5/TNFRSF10A/FCER1G/PPP3CA/ITGAL/PLCG2/PTK2B/PTPN6/NFATC2/HLA-C/LAT/LCP2/MAP2K1/CHP1/PIK3CB/IFNGR1 | 0.006399825 | KEGG |
| PID_P73PATHWAY | -0.362500747 | 0.037626803 | MYC/SERPINE1/RAD51/TP73/CDK2/CHEK1/CCNB1/BRCA2/PLK1/CDK6/CDK1/CCNA2/BUB1 | 0.00650309 | PID |
| PID_RAS_PATHWAY | 0.672166169 | 0.003661128 | RASGRF2/RASGRF1/RASAL1/PRKCB/CAMK2B/PRKCZ/RASGRP4/PRKCE/RASGRP3 | 0.006519986 | PID |
| REACTOME_BASE_EXCISION_REPAIR_AP_SITE_FORMATION | -0.474332612 | 0.012805583 | H4C5/H2BC11/MUTYH/H2AZ1/H2AZ2/TDG/H2BC5/H4C14/H4-16/UNG/H2AX/H2AC8/H4C9/H2AC20/H4C11/H2BC6/NEIL3/H2BC9 | 0.006577534 | REACTOME |
| REACTOME_HEMOSTASIS | 0.465134163 | 8.97E-08 | PRKCG/HBB/SLC8A2/ATP2B3/TUBA8/SLC7A10/PDE2A/GNG3/PDE1A/PDE1B/CD36/KIF5A/SELE/TUBA4A/RAPGEF4/MAG/P2RX5/PRKCB/IGF1/NFE2/TUBB4A/ADRA2C/GATA6/ADRA2B/ARRB1/ITIH3/PPBP/TUBB1/LCK/ATP1B1/ATP2B2/MPIG6B/SELENOP/TF/PRKCZ/PDE11A/ISLR/COL1A2/DGKE/ADRA2A/KIF6/GUCY1B1/VEGFD/EPCAM/DOK2/ITGA2B/GNA14/COL1A1/CLEC3B/DOCK5/TEK/P2RY12/ITPR1/KCNMB4/PRKAR1B/DOCK3/MPL/CFD/F10/SLC7A8/GYPC/CALM1/PRKCQ/JAML/DGKB/KIF1A/GUCY1A1/RAPGEF3/DOCK9/PRKCE/DGKZ/CD2/IRAG1/PLEK/ITGA10/SELPLG/KCNMA1/KIF1C/GNAI1/L1CAM/PHACTR2/EHD3/SLC16A8/CABLES1/ITPK1/MAPK3/PRKACB/SRGN/CD48/THBD/GNGT2/RARRES2/PRKAR2B/CD84/CTSW/F2RL2/PRKCD/CD244/DOCK2/VAV1/ATP2A3/SYK/PCDH7/GAS6/KLC1/PIK3R6/RAB27B/MMP1/STXBP2/NOS3/RAC2/GATA2/PRKCH/FERMT3/ITGAM/KIFC2/PIK3R5/TNFRSF10A/FCER1G/VWF/PDGFA/MERTK/ITGAL/TSPAN7/F13A1/PLCG2/ARRB2/P2RX1/DOCK8/SLC7A7/TTN/JAK2/PTPN6/KIF3C/PRKG1/SIRPA/PSAP/GNA15/ATP2B1/HABP4/PDE5A/INPP5D/SELL/PDGFB/SOD1/CD74/GNB5/APBB1IP/SDC2/THPO/KCNMB2/F2RL3/RHOG/PECAM1/VTI1B/P2RX4/PROCR/ABHD6/LAT/CD99L2/GNAS/GNG11/LYN/LCP2/KIF19/FGR/KLC2/PIK3CB/IGF2/KIF3A/CEACAM1/RASGRP2/ESAM/MMRN1/DGKG/DGKQ/IRF1/PPP2R5A/SLC8A1/TMSB4X/VEGFC/CD47/F8/P2RX6/ANGPT1/ECM1/MGLL/ITGB2 | 0.006736745 | REACTOME |
| BIOCARTA_FMLP_PATHWAY | 0.62436371 | 0.012471314 | CAMK1G/PAK1/PPP3CB/CALM1/CALM3/MAPK3/NCF2/NCF1/PPP3CA/ELK1/NFATC2/GNA15/FPR1/PLCB1/CALM2/MAP2K1 | 0.006897674 | BIOCARTA |
| BIOCARTA_TCAPOPTOSIS_PATHWAY | 0.815477455 | 0.029481676 | CD28/CD3E/CD247/CD3D/CD3G/CD4/CCR5 | 0.007116204 | BIOCARTA |
| REACTOME_SYNTHESIS_OF_VERY_LONG_CHAIN_FATTY_ACYL_COAS | 0.647755199 | 0.044534316 | ELOVL7/HSD17B3/ACSL6/ACSL5/HACD4/ELOVL1/ACSL1/ELOVL4/ACSL4 | 0.00714715 | REACTOME |
| REACTOME_ADRENALINE_NORADRENALINE_INHIBITS_INSULIN_SECRETION | 0.653880323 | 0.01532381 | GNG3/ADRA2C/ADRA2A/ADCY5/GNAI1/CACNB2/GNGT2/CACNA2D2/CACNA1D/CACNB3/GNB5/GNG11/CACNA1C | 0.007240984 | REACTOME |
| PID_IL23_PATHWAY | 0.633784983 | 0.013013882 | STAT4/TNF/IL6/MPO/CXCL9/CXCL1/CD3E/IL18RAP/IL18R1/IL12RB1/CD4/JAK2/IL1B/IL18/IL23A/CCL2 | 0.007344183 | PID |
| KEGG_GRAFT_VERSUS_HOST_DISEASE | 0.75926496 | 5.48E-05 | CD28/TNF/IL6/KLRD1/HLA-DPB1/HLA-DQA1/HLA-B/HLA-DQB1/GZMB/CD80/PRF1/HLA-DPA1/HLA-E/HLA-DMA/HLA-F/HLA-DRB1/HLA-DRA/HLA-DRB5/HLA-DMB/HLA-C/IL1B/HLA-DOA/CD86 | 0.007380433 | KEGG |
| HALLMARK_ESTROGEN_RESPONSE_EARLY | 0.442774992 | 0.019262768 | CCN5/PGR/CLIC3/CALB2/CXCL12/CALCR/TGM2/SYNGR1/NBL1/RAPGEFL1/KLF4/AQP3/CBFA2T3/OLFM1/NPY1R/TPD52L1/DEPTOR/ADCY1/SLC7A2/SEMA3B/TPBG/EGR3/SFN/IGFBP4/ITPK1/FCMR/SVIL/KRT18/FRK/TMPRSS3/SH3BP5/ADD3/CYP26B1/RPS6KA2/OLFML3/PLAAT3/PTGES/TFAP2C/BLVRB/WFS1/FOXC1/HSPB8/MLPH/JAK2/ZNF185/B4GALT1/SYT12/PMAIP1/THSD4/FOS/P2RY2/SULT2B1/PPIF/PAPSS2/OPN3/INPP5F/DHRS3 | 0.007465443 | HALLMARK |
| KEGG_ALLOGRAFT_REJECTION | 0.761143438 | 7.67E-05 | IL10/CD28/TNF/HLA-DPB1/HLA-DQA1/HLA-B/HLA-DQB1/GZMB/CD80/PRF1/HLA-DPA1/CD40/HLA-E/HLA-DMA/HLA-F/HLA-DRB1/HLA-DRA/HLA-DRB5/HLA-DMB/HLA-C/HLA-DOA/CD86 | 0.007659642 | KEGG |
| BIOCARTA_FCER1_PATHWAY | 0.582462111 | 0.0187472 | PRKCB/PPP3CB/CALM1/CALM3/MAPK3/MAP2K4/VAV1/SYK/FCER1G/PPP3CA/BTK/ELK1/NFATC2/FOS/CALM2/FCER1A/LYN/MAP2K1 | 0.007701062 | BIOCARTA |
| REACTOME_INTERLEUKIN_10_SIGNALING | 0.682264395 | 0.000234926 | CCL19/CCL3/CCL3L3/IL10/CCL4/TNF/IL6/CCL5/CXCL10/CXCL1/CCR1/IL10RA/CD80/IL1R1/CCR2/IL1RN/PTAFR/CXCL2/FPR1/CCR5/IL1B/CD86/CXCL8/IL18/TNFRSF1B/CCL2/PTGS2/ICAM1 | 0.007921782 | REACTOME |
| REACTOME_PHASE_0_RAPID_DEPOLARISATION | 0.688675326 | 0.001618801 | CAMK2A/SCN2B/FGF13/SCN3B/CAMK2B/SCN2A/CACNG8/SCN1B/CAMK2G/CALM1/SCN8A/CACNB2/CACNA2D2/FGF14/CACNB1/SCN4B/FGF12 | 0.008008039 | REACTOME |
| KEGG_B_CELL_RECEPTOR_SIGNALING_PATHWAY | 0.549555946 | 0.003845954 | PRKCB/CD22/IFITM1/PPP3CB/CARD11/PPP3R1/PIK3CD/RASGRP3/MAPK3/DAPP1/PIK3AP1/VAV1/SYK/CD79A/RAC2/PIK3R5/BLNK/CD79B/PPP3CA/BTK/PLCG2/PTPN6/NFATC2/INPP5D/CD72/LILRB3/FOS/LYN/MAP2K1/CHP1/PIK3CB | 0.008475255 | KEGG |
| REACTOME_COSTIMULATION_BY_THE_CD28_FAMILY | 0.594315748 | 0.000846628 | GRAP2/CD28/LCK/CD3E/PAK1/MAP3K8/PDCD1/CTLA4/HLA-DPB1/CD247/HLA-DQA1/CD3D/HLA-DQB1/PRR5/CD80/CD3G/HLA-DPA1/PAK3/ICOSLG/VAV1/CD4/TNFRSF14/HLA-DRB1/PTPN6/HLA-DRA/HLA-DRB5/CD274/CD86/LYN | 0.008862673 | REACTOME |
| REACTOME_INTERLEUKIN_2_FAMILY_SIGNALING | 0.665708285 | 0.00093484 | STAT4/LCK/IL2RB/CSF2RB/JAK3/PIK3CD/IL15/IL3RA/CSF2RA/SYK/LGALS9/IL15RA/IL2RG/HAVCR2/JAK2/PTK2B/PTPN6/IL2RA/INPP5D | 0.008915382 | REACTOME |
| KEGG_GAP_JUNCTION | 0.562319672 | 0.001418467 | PRKCG/DRD1/GRM1/HTR2A/TUBA8/TUBA4A/PRKCB/TUBB4A/ADRB1/TUBB1/GUCY1B1/ITPR1/LPAR1/ADCY1/ADCY4/GUCY1A1/ADCY5/GNAI1/HTR2B/ADCY2/MAPK3/PRKACB/PLCB4/PDGFRB/PDGFA/GRM5/PRKG1/PLCB2/PDGFB/PLCB1 | 0.009150607 | KEGG |
| WP_ALLOGRAFT_REJECTION | 0.622349164 | 3.21E-05 | CCL19/C7/IL10/CXCL11/CXCL12/CD28/TNF/PRKCZ/CXCL9/ABCB1/CTLA4/HLA-DPB1/BHMT2/HLA-DQA1/CD55/C2/HLA-B/HLA-DQB1/C1QC/C1QA/GZMB/CD80/PRF1/HLA-DPA1/C1QB/CD40/HLA-E/HLA-DMA/HLA-F/HLA-DRB1/IL2RA/HLA-DRA/HLA-DRB5/HLA-DMB/HLA-C/IL1B/HLA-DOA/CD86/GDNF/CXCL8 | 0.00934691 | WP |
| REACTOME_REGULATION_OF_TLR_BY_ENDOGENOUS_LIGAND | 0.702689713 | 0.048323881 | CD36/S100A1/CD14/S100A9/TLR2/TLR4/S100A8/LY96/TLR6/TLR1 | 0.009837479 | REACTOME |
| WP_INTERACTIONS_OF_NATURAL_KILLER_CELLS_IN_PANCREATIC_CANCER | 0.657617445 | 0.026538166 | CCL3/CCL4/TNF/CCL5/CD96/GZMB/PRF1/FCGR3A/KLRK1/GATA2/CD226 | 0.009942563 | WP |

**Table S6.** A summary of the genes in the five Radscore-related modules.

| **Module** | | **Genes within the module** | |
| --- | --- | --- | --- |
| Turquoise module (ME1) | | AACS, AAK1, AAMDC, AAR2, AATF, ABCA1, ABCA5, ABCA9, ABCB1, ABCB9, ABCC8, ABCD1, ABCD2, ABCG2, ABCG4, ABHD11, ABHD4, ABHD5, ABI1, ABLIM1, ABLIM2, ABR, AC000093.1, AC000095.3, AC000403.1, AC004221.1, AC004233.2, AC004241.5, AC004816.1, AC004947.2, AC005070.3, AC005224.3, AC005330.1, AC005609.5, AC005696.4, AC005726.1, AC005753.3, AC005833.1, AC006058.3, AC006115.2, AC006449.7, AC006450.3, AC006504.3, AC006538.1, AC007326.4, AC007375.3, AC007622.1, AC007666.1, AC007686.3, AC007743.1, AC008038.1, AC008073.3, AC008105.1, AC008115.4, AC008397.2, AC008771.1, AC008875.3, AC008894.2, AC009005.1, AC009065.7, AC009084.2, AC009102.2, AC009119.1, AC009126.1, AC010132.3, AC010186.2, AC010186.3, AC010336.2, AC010336.5, AC010501.2, AC010624.2, AC010735.2, AC011330.1, AC011455.6, AC011484.1, AC012085.1, AC015712.2, AC015726.1, AC015971.1, AC016629.3, AC016924.1, AC018730.1, AC019069.1, AC019171.1, AC020915.2, AC021016.1, AC021683.1, AC022417.1, AC022960.2, AC023301.1, AC023593.1, AC023906.3, AC024293.1, AC024597.1, AC025287.2, AC026273.1, AC026356.1, AC026367.2, AC026403.1, AC026979.3, AC026979.4, AC027097.1, AC027097.2, AC027130.1, AC027307.2, AC027307.3, AC048382.5, AC053513.1, AC055839.2, AC063919.1, AC066612.2, AC072061.1, AC073046.4, AC073270.2, AC073346.2, AC073476.2, AC073508.2, AC074387.1, AC078819.1, AC079140.6, AC079834.1, AC079848.1, AC080038.1, AC083799.1, AC083862.2, AC084866.1, AC087289.5, AC090241.3, AC090510.1, AC091152.2, AC091729.3, AC091825.3, AC092171.3, AC092376.2, AC092376.3, AC092691.1, AC092718.3, AC093673.1, AC096636.1, AC096667.1, AC096733.2, AC097382.3, AC097639.1, AC098582.1, AC098595.1, AC099521.4, AC099754.1, AC100793.3, AC103591.4, AC103719.1, AC104072.1, AC104836.1, AC105345.1, AC105749.1, AC107027.3, AC107398.3, AC107918.4, AC107959.1, AC108479.1, AC108519.1, AC110619.1, AC112503.2, AC113189.2, AC113935.1, AC114284.1, AC114811.2, AC115284.2, AC116565.1, AC120036.4, AC121493.1, AC123768.3, AC124016.2, AC124303.2, AC127070.2, AC129492.3, AC129507.1, AC132219.2, AC134312.1, AC136632.1, AC138393.1, AC138430.2, AC138649.1, AC139795.1, AC188617.1, AC231759.2, AC233723.2, AC241952.1, AC243829.1, AC244669.2, AC245297.1, ACADSB, ACAP2-IT1, ACAT2, ACBD7, ACKR3, ACO2, ACOT4, ACOT7, ACP6, ACSL6, ACTA1, ACTC1, ACTG1, ACTL10, ACTL6A, ACTL6B, ACTN4, ACTR10, ACTR1A, ACTR1B, ACTR3B, ACVR1B, ACVR1C, ADAD2, ADAM11, ADAM1B, ADAM22, ADAM23, ADAMTS8, ADAMTS9, ADARB1, ADARB2, ADCY1, ADCY2, ADCY5, ADCYAP1, ADD3, ADGRA1-AS1, ADGRD1, ADGRE5, ADGRF5P1, ADGRV1, ADHFE1, ADPRHL1, ADPRS, ADRA1A, ADRA1B, ADRA2A, ADRA2C, ADRB1, ADRM1, AEBP1, AEN, AF106564.1, AF111167.2, AF131216.3, AFF3, AGA, AGAP11, AGAP2, AGAP2-AS1, AGAP3, AGBL4, AGPAT3, AGTPBP1, AHCY, AHCYL1, AHI1, AIFM3, AIMP1, AIMP2, AJAP1, AJM1, AJUBA, AK2, AKAP11, AKAP5, AKAP6, AKR1C1, AKR1C2, AKR7A3, AKT1S1, AKTIP, AL008718.2, AL008729.2, AL022313.4, AL022322.1, AL022328.3, AL022328.4, AL022345.4, AL034348.1, AL035701.1, AL049779.4, AL049796.1, AL096706.1, AL109809.4, AL117332.1, AL117334.1, AL117335.1, AL121929.2, AL132656.2, AL132656.4, AL133351.4, AL136964.1, AL136981.3, AL137009.1, AL138955.1, AL139094.1, AL139232.1, AL139384.2, AL158211.1, AL158211.5, AL158212.3, AL159978.1, AL161668.4, AL161772.1, AL162171.1, AL162171.3, AL162311.3, AL162412.1, AL353743.2, AL354718.3, AL355385.1, AL355472.3, AL356019.2, AL356056.2, AL356776.2, AL357874.3, AL359317.1, AL359715.3, AL365361.1, AL390755.1, AL390955.2, AL391422.4, AL450326.1, AL451085.2, AL512785.2, AL583856.2, AL589987.1, AL590560.2, AL590867.2, AL662844.4, AL670729.3, AL671277.2, AL691432.4, AL691447.2, AL713852.1, AL731533.2, ALDH2, ALDH4A1, ALDH6A1, ALDOC, ALG2, ALG3, ALG5, ALG8, ALKBH1, AMIGO1, AMN1, AMOT, AMPD2, AMPH, AMZ1, ANAPC11, ANK1, ANK2, ANK3, ANKRD16, ANKRD19P, ANKRD24, ANKRD29, ANKRD33B, ANKRD34A, ANKRD39, ANKRD46, ANKS1A, ANKS1B, ANO3, ANO5, ANO7, ANP32B, ANXA2R, ANXA3, ANXA5, ANXA6, AP000223.1, AP000280.1, AP000311.1, AP000344.1, AP000345.2, AP000350.5, AP000356.2, AP000356.5, AP001148.1, AP001160.3, AP001178.3, AP001372.2, AP001486.2, AP001528.2, AP001972.5, AP002414.2, AP002847.1, AP003108.1, AP003119.3, AP003486.1, AP003721.1, AP1S2, AP2A1, AP2A2, AP2M1, AP3B1, AP3B2, AP3M2, AP4M1, AP4S1, AP5M1, AP5Z1, APBA1, APBA3, APBB1, APEH, APEX2, APH1A, APLF, APOBEC3B, APOLD1, APRT, APTR, AQP11, AQP3, ARAF, ARF3, ARF4, ARFGAP2, ARFGAP3, ARFGEF2, ARFGEF3, ARFIP1, ARHGAP17, ARHGAP32, ARHGAP44, ARHGAP5-AS1, ARHGDIG, ARHGEF17, ARHGEF25, ARHGEF28, ARHGEF33, ARHGEF4, ARHGEF40, ARHGEF9, ARID5A, ARL13B, ARL3, ARL6, ARMCX5-GPRASP2, ARMCX6, ARMH3, ARNILA, ARPC1A, ARPP19, ARPP21, ARRB1, ARSD, ART3, ARX, ASB13, ASB2, ASCC2, ASIC2, ASPDH, ASPHD2, ASXL3, ATAD1, ATE1, ATF4, ATF7IP2, ATG13, ATG2B, ATG4A, ATL1, ATL3, ATOH7, ATP10A, ATP13A2, ATP1A2, ATP1A3, ATP1B1, ATP2B1, ATP2B1-AS1, ATP2B2, ATP2B3, ATP5MPL, ATP6V0A1, ATP6V0C, ATP6V0D1, ATP6V0E1, ATP6V1A, ATP6V1B2, ATP6V1C1, ATP6V1D, ATP6V1E1, ATP6V1FNB, ATP6V1G2, ATP6V1H, ATP7B, ATP8A2, ATP9A, ATPAF1, ATRAID, ATRNL1, ATXN10, ATXN7L3, AUH, AUP1, AUXG01000058.1, B3GALT6, B3GAT1, B3GNT4, B3GNT5, B4GALT1-AS1, B4GALT4, B4GALT6, B4GALT7, B4GAT1, BAG4, BAIAP2-DT, BAIAP2L2, BAIAP3, BAK1, BANF1, BASP1, BATF3, BAX, BBS7, BCAS4, BCDIN3D, BCKDK, BCL11A, BCL11B, BCL2, BCL2L12, BCL2L2, BCL7C, BCR, BCYRN1, BDH1, BEGAIN, BEND3P3, BEND6, BET1, BEX1, BEX2, BEX3, BEX4, BEX5, BFSP1, BHLHB9, BHLHE22, BICDL1, BIN3, BLCAP, BLOC1S4, BLOC1S5, BLVRA, BMERB1, BMP6, BNIP1, BNIP2, BOLA2B, BORCS5, BORCS8-MEF2B, BRAT1, BRI3BP, BRINP1, BRIX1, BRMS1, BRSK1, BRSK2, BRWD1, BSCL2, BSN, BSPRY, BTBD10, BTBD3, BTBD6, BTBD8, BTBD9, BTF3, BTG3, BTN2A2, BTN2A3P, BTNL9, BTRC, BUD23, BUD31, BX255923.2, C10orf88, C10orf95, C11orf24, C11orf42, C11orf87, C12orf43, C14orf119, C14orf132, C15orf39, C16orf70, C17orf102, C17orf107, C17orf49, C17orf50, C17orf75, C19orf12, C19orf48, C19orf54, C1GALT1C1, C1orf115, C1orf216, C1orf226, C1orf52, C1QL2, C1QL3, C1QTNF1, C1QTNF4, C1QTNF5, C1QTNF7, C20orf194, C20orf27, C21orf62, C22orf39, C2CD2L, C2CD4C, C2CD4D-AS1, C2orf69, C2orf74, C2orf76, C3orf14, C3orf18, C3orf33, C3orf80, C6orf136, C6orf47, C6orf62, C7orf26, C7orf31, C8orf58, C8orf76, C9orf129, C9orf16, CA11, CA4, CA7, CA8, CAB39, CAB39L, CABLES1, CABP1, CABP4, CABYR, CACNA1A, CACNA1C, CACNA1D, CACNA1E, CACNA1G, CACNA1G-AS1, CACNA1I, CACNA2D1, CACNA2D2, CACNA2D3, CACNB1, CACNB2, CACNB3, CACNB4, CACNG3, CACNG8, CACTIN-AS1, CACYBP, CADM3, CADM3-AS1, CADPS, CADPS2, CALB1, CALB2, CALM1, CALM2, CALM3, CALN1, CALR, CALY, CAMK1D, CAMK1G, CAMK2A, CAMK2B, CAMK2G, CAMK2N1, CAMK2N2, CAMK4, CAMKK1, CAMKK2, CAMKV, CAMLG, CAMSAP3, CAMTA1, CAMTA2, CANT1, CAP2, CAPN2, CAPS2, CARD8, CARMIL2, CASD1, CASKIN1, CASP3, CASP6, CASQ1, CASQ2, CASZ1, CAVIN2, CBFA2T3, CBFB, CBLN1, CBLN2, CBLN4, CBX3, CBX6, CBX7, CCDC102A, CCDC107, CCDC110, CCDC136, CCDC144A, CCDC144B, CCDC149, CCDC177, CCDC186, CCDC22, CCDC58, CCDC59, CCDC6, CCDC62, CCDC68, CCDC71, CCDC80, CCDC85A, CCDC90B, CCDC92, CCDC92B, CCK, CCKBR, CCL28, CCM2L, CCN4, CCNA1, CCNK, CCNY, CCSAP, CCSER2, CCZ1, CD151, CD200, CD320, CD47, CD63, CD9, CD99L2, CDC26, CDC37L1, CDC42BPB, CDC42SE1, CDH13, CDH18, CDH22, CDH8, CDHR1, CDIP1, CDK17, CDK5R1, CDK5R2, CDKL1, CDKL2, CDKL5, CDKN2D, CDS1, CDS2, CDYL2, CEBPG, CELF2, CELF2-AS1, CELF3, CELF4, CELF5, CELF6, CEND1, CENPB, CEP112, CEP170B, CERK, CERNA1, CERT1, CES4A, CFAP58-DT, CGAS, CGREF1, CGRRF1, CHCHD3, CHCHD5, CHCHD6, CHD3, CHD5, CHGA, CHGB, CHIC1, CHID1, CHM, CHN1, CHRAC1, CHRD, CHRDL1, CHRM1, CHRM3, CHRM4, CHRNA7, CHRNB2, CHST1, CHST14, CIAO2A, CIAO2B, CIPC, CISD1, CIT, CKAP4, CKLF, CKMT1A, CKMT1B, CLCN4, CLCN6, CLDN10, CLDN9, CLEC18B, CLEC2L, CLIC5, CLIP3, CLN3, CLN5, CLP1, CLPP, CLPTM1L, CLSTN1, CLSTN2, CLSTN3, CLTB, CLUL1, CLVS1, CLVS2, CMAS, CMC4, CMIP, CMTM3, CMTM4, CNBP, CNIH3, CNIH4, CNKSR2, CNN3, CNNM1, CNNM2, CNOT11, CNPPD1, CNPY4, CNRIP1, CNST, CNTN3, CNTN4, CNTN5, CNTN6, CNTNAP1, CNTNAP2, CNTNAP5, COA1, COA4, COASY, COG1, COL22A1, COL26A1, COMMD4, COMMD5, COMMD6, COPG1, COPS6, COPZ1, COQ2, COQ5, COQ8B, CORO2A, CORO6, CORT, COTL1, COX15, COX16, COX5A, COX6A1, COX7A1, CPE, CPEB1, CPEB2, CPEB3, CPEB4, CPLX1, CPLX2, CPLX3, CPNE1, CPNE5, CPNE6, CPNE7, CPNE9, CRABP1, CRACDL, CRBN, CREG2, CRHBP, CRHR1, CRIM1-DT, CRLF1, CRLS1, CROT, CRPPA, CRTAC1, CRTC1, CRY2, CRYM, CRYZ, CRYZL1, CSDC2, CSNK2B, CSRNP3, CSRP2, CT75, CTDNEP1, CTDP1, CTDSP1, CTIF, CTNNA1, CTNNA2, CTNNBL1, CTNND1, CTNS, CTXN1, CTXN2, CU633906.5, CUEDC2, CUL4A, CUX2, CWC25, CX3CL1, CXorf38, CYB5B, CYBC1, CYFIP2, CYGB, CYP26B1, CYP46A1, CYP4X1, CYREN, CYRIA, CYS1, DACH1, DACT3, DAD1, DALRD3, DAP, DAP3, DAPK1, DAXX, DBH, DBNL, DBR1, DCAF11, DCAF6, DCLK1, DCLK3, DCTN1, DCUN1D5, DDAH2, DDB2, DDHD2, DDN, DDN-AS1, DDOST, DDX24, DDX25, DDX39A, DDX47, DDX54, DDX56, DDX59, DEAF1, DEF8, DEFB131E, DEGS1, DEGS2, DENND10, DENND11, DENND2A, DEPDC5, DERA, DERL2, DERPC, DESI1, DGCR5, DGCR6, DGKB, DGKE, DGKG, DGKQ, DGKZ, DHRS1, DHRS11, DHRSX, DHX37, DIABLO, DIO3, DIO3OS, DIRAS1, DIRAS2, DIRAS3, DISP1, DISP2, DISP3, DIXDC1, DKK2, DKK3, DLEU7, DLG2, DLG3, DLG4, DLGAP1, DLGAP1-AS1, DLGAP1-AS2, DLGAP3, DLX1, DLX6-AS1, DMRTA2, DMRTC1, DMRTC1B, DMTN, DNAAF5, DNAJA4, DNAJB11, DNAJB14, DNAJC1, DNAJC12, DNAJC22, DNAJC27, DNAJC28, DNAJC5, DNAJC6, DNAJC8, DNM1, DNM1L, DNM3, DNTTIP1, DOC2A, DOC2B, DOCK3, DOCK9, DOCK9-DT, DOK6, DPAGT1, DPF3, DPM3, DPP9, DPY19L2P1, DRD1, DRP2, DSCAS, DSTYK, DTNB, DTNBP1, DUSP10, DUSP11, DUSP19, DUSP26, DUSP6, DUSP8, DUSP9, DVL1, DYNC1H1, DYNC1I1, DYNLT1, DYRK1A, DYRK4, DZIP3, E2F4, EBPL, ECE2, ECI2, EED, EEF1A1, EEF1A2, EEF1AKMT2, EEF1AKMT4, EEF1D, EEF1G, EFEMP2, EFNA1, EFNA3, EFNA5, EFNB3, EFR3A, EFR3B, EGR3, EGR4, EHD3, EIF2B2, EIF2D, EIF2S3, EIF3B, EIF3D, EIF3E, EIF3G, EIF3I, EIF3M, EIF4A1, EIF4A2, EIF4E3, EIF4EBP1, EIF5A, EIF5A2, EIF6, ELAPOR1, ELAVL2, ELAVL3, ELAVL4, ELK1, ELMO1, ELMOD1, ELOVL4, EMC10, EMC1-AS1, EMD, EMG1, EMILIN1, EML4, EML5, EMP1, EMX1, EMX2, EMX2OS, ENC1, ENDOU, ENHO, ENO2, ENOX1, ENSA, ENTPD3, ENTPD4, ENTPD6, ENTR1, ENY2, EPB41L1, EPB41L3, EPB41L4A-AS1, EPCAM, EPHA10, EPHA4, EPHA5, EPHA7, EPHB6, EPHX1, EPHX4, EPN1, EPOP, EPS15, ERBB4, ERC2, ERCC1, ERF, ERFL, ERGIC3, ERH, ERI1, ERICH3, ERN1, ERP44, ESYT3, ETNPPL, ETV6, EVA1B, EVC2, EVL, EXOC6, EXOC6B, EXOSC4, EXOSC7, EXTL1, EZH1, F10, F2R, FAAP20, FABP3, FABP6, FADD, FAIM2, FAM102B, FAM106A, FAM107A, FAM111A, FAM114A2, FAM126B, FAM131A, FAM131C, FAM133A, FAM135B, FAM13A, FAM13B, FAM149A, FAM155A, FAM160B1, FAM161B, FAM163B, FAM167A, FAM168B, FAM169A, FAM171B, FAM174B, FAM174C, FAM189A1, FAM204A, FAM216A, FAM217B, FAM219A, FAM228A, FAM234A, FAM234B, FAM240C, FAM241B, FAM243A, FAM243B, FAM47E, FAM50A, FAM66A, FAM71E1, FAM81A, FAM86C1P, FAM86C2P, FAM86DP, FAM86EP, FANCF, FAR2P2, FAR2P3, FASTK, FAU, FAXC, FBL, FBXL12, FBXL15, FBXL16, FBXL17, FBXL18, FBXL2, FBXL22, FBXO16, FBXO2, FBXO25, FBXO27, FBXO3, FBXO30-DT, FBXO31, FBXO33, FBXO34, FBXO4, FBXO41, FBXO44, FBXO9, FBXW7, FDFT1, FDX1, FEZF2, FGD6, FGF12, FGF13, FGF17, FGF18, FGF22, FGF7P6, FGF9, FGFR1OP2, FGFR3, FHIT, FHL3, FHL5, FIG4, FJX1, FKBP10, FKBP1B, FKBPL, FLRT2, FLYWCH1, FMC1, FMN1, FNDC11, FNDC5, FNDC9, FNIP2, FO393414.3, FO681492.1, FOCAD, FOXD4L3, FOXD4L6, FOXP2, FP236383.3, FPGS, FRAT1, FRG1BP, FRG1CP, FRMD4A, FRMD8, FRMPD2B, FRRS1L, FRS3, FRY, FSD1L, FSTL4, FSTL5, FUT1, FUT2, FUT9, FXR2, FXYD7, FZD2, FZD9, G3BP1, G3BP2, GABARAPL1, GABBR1, GABBR2, GABRA1, GABRA2, GABRA4, GABRA5, GABRB1, GABRB2, GABRB3, GABRD, GABRG1, GAD2, GAL3ST4, GALK1, GALNS, GALNT10, GALNT16, GALNT17, GALNT18, GALNT9, GANAB, GANC, GAR1, GARNL3, GAS5, GAS6-DT, GAS7, GATAD1, GATAD2A, GATB, GCC1, GCC2, GCNT4, GCSIR, GDA, GDAP1, GDAP1L1, GDAP2, GDF10, GDI1, GDPD5, GEMIN7, GET3, GFM2, GFOD1, GFRA2, GGN, GHITM, GIT1, GJA3, GJB5, GJB6, GLA, GLB1L, GLIPR2, GLIS1, GLIS3, GLRA2, GLRB, GLS, GLS2, GLT1D1, GLT8D1, GLTPD2, GLUD1, GLUD2, GLYCTK, GMFB, GMPPA, GMPPB, GNA13, GNAI1, GNAI2, GNAI3, GNAL, GNAO1, GNAQ, GNAS-AS1, GNAZ, GNB1, GNB2, GNB5, GNG12, GNG3, GNG5, GNL2, GNL3, GNPDA1, GNPTAB, GOLGA7B, GOLGA8M, GOLPH3, GON7, GORAB, GOT1, GOT2, GPAM, GPAT4, GPC5, GPD1L, GPLD1, GPN1, GPR108, GPR12, GPR143, GPR150, GPR155, GPR158, GPR162, GPR176, GPR179, GPR22, GPR27, GPR61, GPR68, GPR88, GPRASP1, GPRASP2, GPRIN1, GRAMD1A, GRAMD1B, GRAMD1C, GREB1, GREB1L, GREM2, GRIN1, GRIN2A, GRIN2B, GRIN2C, GRIN3A, GRIP1, GRIP2, GRIPAP1, GRK3, GRM1, GRM2, GRM5, GRM5-AS1, GRM7, GRWD1, GSDME, GSK3A, GSS, GSTK1, GSTM3, GSTO2, GSTP1, GTDC1, GTF2A2, GTF2B, GTF2E2, GTF2H5, GTF3C6, GUCD1, GUCY1A1, GUCY1B1, GUSB, GYS1, H2AC20, H2AC8, H3-3B, H3C10, H4C11, H6PD, HABP4, HAGH, HAPLN4, HAR1A, HAS2, HBP1, HBQ1, HCN4, HDAC1, HDAC5, HDAC7, HEBP2, HECTD4, HECW1, HECW2, HELZ2, HENMT1, HERC1, HERC2P3, HERC3, HERC5, HESX1, HGH1, HHIPL1, HIBADH, HID1, HIF1A, HIF1AN, HIF3A, HINT3, HIVEP2, HK1, HLF, HM13, HMBS, HMG20B, HMGCLL1, HMGN4, HMOX2, HNF4G, HNRNPF, HOMER1, HOOK1, HOTAIRM1, HOXA1, HOXA5, HOXB2, HOXB7, HPCA, HPCAL1, HPCAL4, HPRT1, HRH2, HRH3, HRK, HS3ST2, HS3ST3B1, HS3ST4, HS6ST3, HSD17B10, HSD17B6, HSD3BP5, HSF1, HSFX2, HSP90AB1, HSP90B1, HSPA12A, HSPA12B, HSPA4L, HSPA5, HSPB3, HSPD1, HSPE1, HSPH1, HTR1B, HTR2A, HTR7P1, HTT, HUS1, HYAL1, IAH1, ICA1, ICAM5, IDI1, IDI2-AS1, IDS, IER5, IER5L, IFITM10, IFRD1, IFRD2, IFT43, IGDCC4, IGFBP2, IGFL4, IGLV5-52, IGSF21, IKBKB, IL1RAP, IL1RAPL1, IL20RB, IL34, ILK, ILRUN, IMMP2L, IMPA1, IMPDH2, INA, INPP4A, INPP4B, INPP5A, INPP5F, INPP5J, INPPL1, INSYN2B, INTS14, IP6K1, IPCEF1, IPO4, IQCA1, IQGAP2, IQSEC1, IQSEC2, IQSEC3, IRAG1, IRAK1, IRAK4, ISCA1, ISG20L2, ISLR2, ISM1, ISY1, ITFG1, ITGA7, ITGA9, ITGA9-AS1, ITGB3BP, ITGB5, ITIH5, ITPA, ITPKA, ITPR1, ITSN1, IVNS1ABP, JAG1, JAG2, JAGN1, JAKMIP1, JCAD, JPH1, JPH3, JPH4, KALRN, KANK3, KATNA1, KBTBD11, KBTBD3, KCNA1, KCNA2, KCNA3, KCNA5, KCNAB1, KCNAB2, KCNB1, KCNC1, KCNC3, KCNC4, KCNH1, KCNH3, KCNH4, KCNIP2, KCNIP3, KCNIP4, KCNJ11, KCNJ12, KCNJ3, KCNJ4, KCNJ6, KCNJ9, KCNK1, KCNK10, KCNK12, KCNK3, KCNK4, KCNK4-TEX40, KCNK9, KCNMA1, KCNN1, KCNQ2, KCNQ3, KCNQ5, KCNS1, KCNS3, KCTD16, KCTD17, KCTD2, KCTD4, KCTD8, KDELR1, KDELR2, KEAP1, KHDRBS2, KHNYN, KIAA0319, KIAA0513, KIAA1109, KIAA1217, KIAA1522, KIAA1549L, KIAA1671, KIAA2013, KIF17, KIF1A, KIF3A, KIF3B, KIF3C, KIF5A, KIF5C, KIFAP3, KIFBP, KIFC2, KIRREL3, KIT, KLC1, KLC2, KLF13, KLF8, KLHDC2, KLHDC3, KLHDC8A, KLHL26, KLHL3, KLHL35, KLLN, KNDC1, KRCC1, KREMEN2, KRI1, KRT10, KRT18P34, KRT8P12, KRTAP5-AS1, KRTCAP2, KXD1, L1CAM, L3HYPDH, LAMA3, LAMA5, LAMB2, LAMP5, LAMTOR2, LAMTOR5, LARGE1, LARP7, LBX2, LBX2-AS1, LCN12, LCNL1, LDB1, LDB2, LDOC1, LEMD2, LEPR, LFNG, LGALS3BP, LGI1, LGI2, LHFPL1, LHFPL4, LHFPL5, LHX6, LIG4, LIN7B, LINC00242, LINC00294, LINC00574, LINC00595, LINC00622, LINC00634, LINC00641, LINC00672, LINC00847, LINC00865, LINC00943, LINC00951, LINC00957, LINC00963, LINC01018, LINC01106, LINC01123, LINC01128, LINC01140, LINC01260, LINC01411, LINC01534, LINC01574, LINC01664, LINC01686, LINC01750, LINC01819, LINC01998, LINC02012, LINC02185, LINC02427, LINC02495, LINC02600, LINC02693, LINC02716, LINC02875, LINGO3, LMAN2, LMBRD2, LMCD1, LMNA, LMO3, LMO7, LMOD1, LMTK2, LMTK3, LNCOC1, LONRF2, LOXL3, LPCAT3, LPCAT4, LPGAT1, LPIN1, LPIN2, LPL, LRFN1, LRFN2, LRFN5, LRP11, LRRC10B, LRRC20, LRRC37A, LRRC3B, LRRC56, LRRC7, LRRC73, LRRC8B, LRRTM4, LRWD1, LSG1, LSM1, LSM10, LSM11, LSM2, LSM5, LSM6, LSM7, LSR, LTBP3, LURAP1, LUZP1, LY6E-DT, LY6H, LYAR, LYNX1, LYPD5, LYPLA1, LYPLA2, LYPLAL1-DT, LYRM4-AS1, LYRM9, LZTS3, MACIR, MACROD2, MADCAM1, MADD, MAF1, MAGEE1, MAGOH, MAL2, MALSU1, MAMSTR, MAN1B1, MAN2A2, MANF, MAOA, MAP1A, MAP1LC3A, MAP1S, MAP2K1, MAP2K4, MAP3K10, MAP3K14, MAP3K21, MAP3K7CL, MAP3K9, MAP4K2, MAP4K3-DT, MAP6, MAP7D2, MAP7D3, MAPK1, MAPK10, MAPK3, MAPK7, MAPK8IP1, MAPK8IP2, MAPK8IP3, MAPK9, MAPKAPK2, MAPRE1, MAPRE3, MAPT-IT1, MARCHF4, MARCHF6, MARF1, MARK4, MAST1, MAST3, MATK, MBLAC1, MBLAC2, MC1R, MCF2, MCF2L, MCF2L2, MCTP1, MDH1, MDK, ME1, ME3, MEAF6, MED11, MED14OS, MED18, MED22, MEF2A, MEF2C, MEF2D, MEG3, MEGF10, MEGF9, MEPCE, MEPE, MESD, MESP2, METTL18, METTL23, METTL7B, MEX3C, MEX3D, MFN2, MFSD10, MFSD13A, MFSD14B, MFSD4A, MFSD5, MFSD6, MGAT3, MGAT4B, MGAT5B, MGLL, MGME1, MIAT, MICAL2, MICAL3, MICU3, MIDN, MIGA1, MINDY3, MINK1, MIR124-1HG, MIR124-2HG, MIR137HG, MIR1915HG, MIR193BHG, MIR3659HG, MIR3936HG, MIR600HG, MIR7-3HG, MKX, MLEC, MLLT6, MLX, MMD, MMP17, MMP24, MN1, MNT, MOAP1, MOB3A, MOGS, MORC4, MOSMO, MOSPD3, MOV10, MPDU1, MPHOSPH8, MPLKIP, MPO, MPP2, MPP6, MPP7, MPPED1, MPRIP, MPV17, MPV17L2, MRAP2, MRAS, MRC2, MRGBP, MRM2, MRO, MROH1, MRPL11, MRPL12, MRPL13, MRPL14, MRPL17, MRPL32, MRPL36, MRPL4, MRPL47, MRPL51, MRPL58, MRPS12, MRPS18B, MRPS24, MRPS9-AS2, MRTFB, MSMO1, MSRB1, MSRB2, MTA3, MTATP6P1, MTCH1, MTFP1, MTFR1, MTG2, MTHFR, MTMR11, MTMR12, MTMR6, MTMR7, MTMR9, MTX1, MTX2, MUSTN1, MVB12A, MXI1, MYADML2, MYBPC1, MYCBP, MYCBP2, MYDGF, MYH11, MYH15, MYH7B, MYL6, MYLK4, MYO5A, MYO9B, MYOM1, MYOM2, MYORG, MYPOP, MYRIP, MYT1L, N4BP2L1, N4BP3, NAA30, NAALAD2, NACA, NADK, NAGLU, NAGPA-AS1, NALCN, NAP1L2, NAP1L3, NAP1L5, NAP1L6P, NAPB, NAPG, NAPRT, NAT8L, NAV2-AS2, NAV3, NBEA, NBL1, NBPF14, NBPF8, NCDN, NCEH1, NCK1, NCKIPSD, NCLN, NCOA2, NCOA7, NCR3LG1, NCS1, NCSTN, NDFIP1, NDFIP2, NDN, NDRG2, NDRG3, NDRG4, NDST3, NDUFA10, NDUFA4, NDUFA5, NDUFA6-DT, NDUFA9, NDUFAF4, NDUFAF5, NDUFB8, NDUFB9, NEBL, NEBL-AS1, NECAB1, NECAB2, NECAB3, NECAP1, NECAP2, NECTIN1, NEDD4L, NEDD9, NEFH, NEFL, NEFM, NEGR1, NEK6, NELFE, NELL1, NELL2, NEMF, NES, NET1, NEU3, NEURL1, NEURL4, NEUROD1, NEUROD2, NEXMIF, NFASC, NGB, NGEF, NGRN, NHLRC1, NHSL2, NICN1, NIFK, NIP7, NIPAL2, NIPSNAP3B, NISCH, NKD2, NKIRAS1, NKRF, NKX2-5, NLK, NMB, NMD3, NME2, NME4, NMNAT2, NMT1, NMT2, NNAT, NOL8, NOP16, NOP2, NORAD, NOS1, NOS1AP, NOTCH2NLB, NOVA2, NPAS1, NPDC1, NPM1, NPM1P27, NPM2, NPM3, NPPC, NPTN, NPTN-IT1, NPTX1, NPTX2, NPTXR, NPY, NPY1R, NR1D2, NR2C2AP, NR2F1-AS1, NR3C2, NRBP1, NRG3, NRGN, NRIP2, NRIP3, NRSN1, NRSN2, NRXN1, NRXN3, NSF, NSFP1, NSG1, NSG2, NSMAF, NSMCE1, NSMCE2, NSMF, NSRP1, NSUN2, NSUN5, NSUN7, NT5C1A, NT5C2, NT5DC3, NTM, NTMT1, NTN4, NTNG2, NTRK2, NTSR2, NUAK1, NUBP1, NUDCD3, NUDT19, NUDT22, NUDT3, NUDT7, NUP37, NUP50-DT, NUP54, NUP62, NUS1P2, NWD1, NXPH2, NXT1, NYAP1, OAT, OBI1-AS1, OCIAD1, OCLN, OGA, OGDHL, OGFOD2, OGFOD3, OIP5-AS1, OLA1, OLFM1, OLFM3, OLFM4, OMA1, OMD, OPA1, OPRD1, OPRL1, OPTN, OR2C1, OR2L13, ORMDL2, OSBP2, OSBPL9, OST4, OSTC, OTUB1, OTUD5, OTUD6B-AS1, OTULIN, OVCA2, OXA1L, OXCT1, OXR1, OXSM, P2RX5, P2RX6, P2RX6P, P2RY14, P3H1, P3H3, P3H4, P4HB, PABPC1, PABPC1L2B, PABPC1P4, PACSIN1, PAF1, PAFAH1B1, PAFAH2, PAIP2, PAK1, PAK3, PAK4, PAK5, PAK6, PALLD, PALM, PANX2, PAQR9, PARD6A, PARM1, PARP4, PART1, PBDC1, PC, PCBP1, PCBP2, PCBP3, PCCA-DT, PCDH20, PCDH7, PCDH8, PCDHA12, PCDHA5, PCDHA6, PCDHAC2, PCDHGA2, PCDHGA3, PCDHGB5, PCDHGC5, PCLO, PCMT1, PCP4, PCP4L1, PCSK1N, PCSK2, PCYOX1L, PCYT1A, PDAP1, PDCD5, PDCD6-AHRR, PDCL3, PDE1A, PDE1B, PDE2A, PDE3B, PDE4A, PDE4DIP, PDE8B, PDGFC, PDHA1, PDHX, PDIA3, PDIA4, PDIA6, PDK2, PDLIM3, PDLIM7, PDP1, PDPK1, PDRG1, PDS5B, PDXK, PDXP, PDYN, PDZD2, PDZD4, PDZD7, PDZD8, PDZD9, PEA15, PEBP1, PEBP4, PECR, PEG13, PEG3, PELI3, PENK, PER3, PEX26, PFDN5, PFKFB2, PFKP, PFN1, PFN2, PGAM1, PGAP4, PGAP6, PGBD5, PGLS, PGM2, PGP, PGPEP1, PHACTR1, PHB2, PHC2, PHF24, PHF5A, PHLDB3, PHLPP2, PHYHIP, PHYKPL, PI4KA, PIANP, PIGBOS1, PIGC, PIGCP1, PIGZ, PIK3CB, PIK3R1, PIN1, PINK1, PINLYP, PIP4K2B, PIP4K2C, PIP5K1B, PIP5K1C, PISD, PITHD1, PITPNA, PITPNM2, PITPNM3, PITRM1, PITX1, PKD2, PKDCC, PKIA, PKMP3, PKN1, PKP2, PLA1A, PLA2G12A, PLAAT5, PLCB1, PLCB3, PLCB4, PLCH1, PLCL2, PLCXD2, PLCXD3, PLD6, PLEKHA1, PLEKHA4, PLEKHA5, PLEKHA6, PLEKHA8P1, PLEKHB2, PLEKHF2, PLEKHG2, PLEKHG5, PLEKHH3, PLEKHJ1, PLEKHM2, PLEKHM3, PLGRKT, PLIN1, PLIN5, PLK2, PLOD3, PLPPR3, PLPPR4, PLSCR3, PLXNA3, PLXNB2, PMS2CL, PMS2P1, PNCK, PNKP, PNMA2, PNMA3, PNMA6A, PNMA8A, PNMA8B, PNMA8C, PNMT, PNO1, PNOC, PNP, POC5, POFUT2, POGLUT3, POLB, POLD1, POLD2, POLM, POLR2G, POLR2H, POLR2J, POLR3A, POMGNT1, POP5, POPDC3, POR, POT1, PPARGC1B, PPFIA2, PPFIA3, PPFIA4, PPIA, PPIB, PPIE, PPIL3, PPIP5K1, PPM1A, PPM1H, PPM1K, PPM1L, PPP1CA, PPP1CB, PPP1R12B, PPP1R13B, PPP1R14B, PPP1R14C, PPP1R16B, PPP1R18, PPP1R1A, PPP1R1B, PPP1R21, PPP1R26-AS1, PPP1R35, PPP1R37, PPP1R3B, PPP1R3E, PPP1R3F, PPP1R7, PPP1R9A, PPP1R9A-AS1, PPP1R9B, PPP2CA, PPP2R2C, PPP2R2D, PPP2R5B, PPP3CA, PPP3CB, PPP3R1, PPP4C, PPP4R1L, PPP4R4, PRDM11, PRDM16, PRDM16-DT, PRDM2, PRDM8, PRDX4, PRELID1, PRELID3B, PRELP, PREPL, PRICKLE1, PRICKLE2, PRKAB2, PRKACB, PRKAG2, PRKAG2-AS1, PRKAR1A, PRKAR1B, PRKAR1B-AS1, PRKAR2B, PRKCB, PRKCE, PRKCG, PRKCSH, PRKCZ, PRKD2, PRKN, PRKRIP1, PRMT1, PRMT8, PRNP, PRODH, PRPF19, PRPH, PRPH2, PRRC1, PRRG3, PRRT1, PRRT2, PRRT3, PRRX1, PRSS3, PRXL2A, PRXL2C, PSD, PSD3, PSENEN, PSMA2, PSMA3, PSMA4, PSMA7, PSMB1, PSMB3, PSMB4, PSMC2, PSMC4, PSMD13, PSMD9, PSME1, PSME2, PSMF1, PSMG3, PSTK, PTBP1, PTCD1, PTDSS1, PTER, PTGDR2, PTGES2, PTGES3P2, PTH1R, PTK2B, PTPN12, PTPN20, PTPN3, PTPN4, PTPN5, PTPRB, PTPRM, PTPRN, PTPRN2, PTPRR, PTPRT, PTRH2, PTTG1IP, PUM3, PURA, PVALB, PVT1, PWAR5, PWWP2B, PWWP3B, PXDC1, PXN, PYGL, PYGM, PYURF, QARS1, QPCT, QTRT2, R3HDM1, R3HDM2, R3HDM4, RAB11FIP2, RAB11FIP4, RAB11FIP5, RAB15, RAB18, RAB26, RAB27B, RAB31, RAB3A, RAB3B, RAB3C, RAB40C, RAB4A, RAB5IF, RAB6A, RAB6B, RAB8A, RAB9B, RABEP1, RABGAP1L, RABL6, RAC1, RACK1, RAD23A, RAET1E-AS1, RAI2, RALA, RALY, RALYL, RAMAC, RANBP3L, RANGAP1, RAP1B, RAP1GAP, RAP1GAP2, RAP1GDS1, RAPGEF2, RAPGEF4, RAPGEFL1, RASA4CP, RASAL1, RASD2, RASGEF1A, RASGRF2, RASGRP1, RASL10A, RB1CC1, RBCK1, RBFOX1, RBFOX2, RBFOX3, RBIS, RBM11, RBM22, RBM24, RBM3, RBM34, RBM38, RBM42, RBMS3, RBP1, RBP4, RCAN2, RCC1L, RCE1, RDH12, RDH5, REEP1, REEP5, RELA, RELCH, RELL1, RELL2, RELN, REM2, REPS2, RER1, RERG, RERGL, REST, RETREG2, REXO1, RFPL1S, RFXANK, RGL1, RGL2, RGPD6, RGPD8, RGR, RGS11, RGS20, RGS4, RGS5, RGS7, RGS7BP, RGS8, RHBDF1, RHBDL1, RHCG, RHOA, RHOBTB2, RHOC, RHOF, RHOJ, RHOV, RIIAD1, RIMBP2, RIMKLA, RIMS1, RIMS2, RIMS3, RIMS4, RIPK1, RIPK2, RIPOR1, RIPOR2, RIT1, RND1, RNF103, RNF103-CHMP3, RNF11, RNF112, RNF114, RNF115, RNF144A-AS1, RNF150, RNF170, RNF175, RNF208, RNF41, RNF43, RNF44, RNF7, RNPEP, ROCK1P1, ROMO1, ROPN1B, RORB-AS1, RP2, RP9, RPA3, RPARP-AS1, RPF1, RPF2, RPH3A, RPIA, RPL10, RPL10A, RPL11, RPL12, RPL12P4, RPL13, RPL13A, RPL14, RPL17, RPL18, RPL18A, RPL19, RPL22L1, RPL23, RPL23A, RPL24, RPL26L1, RPL27, RPL27A, RPL28, RPL29, RPL30, RPL32, RPL35, RPL35A, RPL36, RPL36A, RPL39, RPL41, RPL5, RPL6, RPL7, RPL7A, RPL8, RPL9, RPLP0, RPLP0P6, RPLP1, RPLP2, RPN1, RPN2, RPRML, RPS10, RPS10-NUDT3, RPS11, RPS13, RPS14, RPS15, RPS15A, RPS16, RPS17, RPS18, RPS19, RPS2, RPS20, RPS21, RPS25, RPS27L, RPS28, RPS2P32, RPS2P5, RPS3, RPS3A, RPS3AP38, RPS4X, RPS5, RPS6KA5, RPS6KB2, RPS7, RPS8, RPS9, RPSA, RRBP1, RRN3P3, RRP36, RRP8, RRP9, RSKR, RSPO2, RTBDN, RTL10, RTL8C, RTN1, RTN2, RTN3, RTN4, RTN4R, RTN4RL1, RTN4RL2, RTP1, RUBCNL, RUFY1, RUNDC3A, RUNDC3A-AS1, RUNDC3B, RUNX1T1, RUSC1, RUSC2, RXRG, RYK, RYR1, RYR2, S100A16, S100A2, S100A3, S1PR1, S1PR2, SACM1L, SAMD12, SAMD9, SAMD9L, SAP30, SARAF, SARNP, SAYSD1, SBF1, SBNO1, SC5D, SCAI, SCAMP1, SCAMP3, SCAMP4, SCAMP5, SCAPER, SCG5, SCN2A, SCN2B, SCN3B, SCN4B, SCN8A, SCNM1, SCOC, SCP2, SCRT1, SDF2, SDF4, SDHA, SDHAF2, SDHAF4, SEC11A, SEC13, SEC16B, SEC61A1, SEC61A2, SEC61B, SEC61G, SEC62, SELENBP1, SELENOI, SELENOK, SELENOS, SEM1, SEMA3G, SEMA4A, SEMA4F, SEMA6B, SEPHS1P6, SEPTIN3, SEPTIN5, SEPTIN6, SERINC1, SERINC3, SERP2, SERPINB6, SERPINI1, SERTAD4, SERTM1, SESN1, SEZ6L2, SF3B5, SF3B6, SFTA1P, SFTPD, SGCD, SGCG, SGIP1, SGPP2, SGSH, SGSM1, SGSM2, SGTB, SH2B2, SH2D4A, SH2D5, SH3BGRL2, SH3BP2, SH3BP5, SH3GL1, SH3GL2, SH3GLB1, SH3PXD2B, SHANK1, SHANK2, SHANK3, SHARPIN, SHE, SHF, SHISA5, SHISA8, SHISA9, SHISAL1, SHMT2, SHOC2, SHOX2, SIDT1, SIK3, SIL1, SINHCAF, SIRT5, SIVA1, SKIV2L, SLC12A4, SLC12A5, SLC12A9, SLC13A3, SLC13A5, SLC14A1, SLC16A1, SLC16A7, SLC16A8, SLC17A7, SLC1A2, SLC1A3, SLC1A4, SLC22A17, SLC22A3, SLC24A4, SLC25A12, SLC25A13, SLC25A14, SLC25A16, SLC25A18, SLC25A21-AS1, SLC25A22, SLC25A23, SLC25A27, SLC25A33, SLC25A39, SLC25A4, SLC25A41, SLC25A42, SLC25A48, SLC26A2, SLC26A4, SLC27A4, SLC2A10, SLC2A11, SLC2A12, SLC2A13, SLC2A4RG, SLC2A6, SLC30A10, SLC30A3, SLC30A4, SLC30A5, SLC30A7, SLC30A9, SLC32A1, SLC33A1, SLC35A2, SLC35A4, SLC35B3, SLC35C2, SLC35D2, SLC35F2, SLC35F3, SLC35F5, SLC35F6, SLC38A10, SLC39A1, SLC39A10, SLC39A12, SLC39A7, SLC41A3, SLC43A3, SLC45A1, SLC45A4, SLC4A10, SLC4A2, SLC52A2, SLC52A3, SLC5A4, SLC66A1, SLC66A2, SLC6A1, SLC6A12, SLC6A13, SLC6A15, SLC6A17, SLC7A10, SLC7A14, SLC7A4, SLC8A1, SLC8A2, SLC9A5, SLC9A6, SLC9A7, SLC9A7P1, SLC9B2, SLCO1C1, SLIT2, SLIT3, SLITRK1, SLITRK4, SLITRK5, SLK, SLU7, SLX1A, SMAP2, SMARCA2, SMARCAL1, SMIM10L2A, SMIM10L2B, SMIM13, SMIM15, SMIM17, SMIM24, SMIM30, SMIM32, SMIM4, SMIM7, SMN1, SMO, SMPD3, SNAI3-AS1, SNAP25, SNAP25-AS1, SNAP47, SNAP91, SNAPC1, SNAPC2, SNCA, SNCB, SNCG, SND1, SNHG12, SNHG14, SNHG15, SNHG16, SNHG17, SNHG18, SNHG26, SNHG28, SNHG3, SNHG7, SNPH, SNRPB, SNRPB2, SNRPC, SNRPD2, SNRPE, SNRPF, SNRPG, SNRPN, SNTA1, SNTB1, SNU13, SNURF, SNW1, SNX32, SNX33, SNX5, SNX7, SNX8, SOCS2, SORBS1, SORBS2, SORCS1, SORCS2, SOSTDC1, SOWAHA, SOX1, SOX17, SOX1-OT, SOX7, SP9, SPARC, SPARCL1, SPATA2, SPATA5L1, SPATS2L, SPCS2, SPG21, SPHKAP, SPIN1, SPIRE2, SPNS1, SPOCK1, SPPL2A, SPRN, SPRY1, SPRY2, SPRY4, SPRYD3, SPRYD7, SPSB2, SPTB, SPTBN1, SPTBN2, SPTBN4, SPTSSA, SPX, SRA1, SREBF2, SRPRB, SRPX, SRRM3, SRRM4, SRSF9, SSBP1, SSBP3, SSNA1, SSR1, SSR2, SSR4, SST, SSTR1, SSTR2, SSX2IP, ST6GAL2, ST6GALNAC1, ST6GALNAC5, ST6GALNAC6, ST8SIA3, ST8SIA6, STAC2, STAM, STAMBPL1, STAP2, STAR, STARD10, STARD3NL, STARD4, STAT4, STAU2, STEAP2, STIM1, STK10, STK16, STK17A, STK3, STK32C, STK38, STK40, STMN2, STMN3, STMN4, STMP1, STOML1, STOX1, STOX2, STRIP2, STS, STT3A, STT3B, STUM, STX10, STX1A, STX1B, STX5, STX7, STXBP1, STXBP5, STXBP5L, STXBP6, STYX, STYXL1, SUCLA2, SULT4A1, SUMF2, SV2A, SV2B, SV2C, SVOP, SWI5, SYAP1, SYBU, SYDE1, SYF2, SYN1, SYN2, SYN3, SYNDIG1L, SYNE1, SYNGAP1, SYNGR1, SYNGR3, SYNJ1, SYNPR, SYP, SYS1, SYT1, SYT12, SYT13, SYT14, SYT15, SYT16, SYT3, SYT4, SYT5, SYT7, SYTL5, SYVN1, SZRD1, TAC1, TAC3, TACR2, TADA3, TAF12, TAF4B, TAFA1, TAFA2, TAFA5, TAGLN3, TAMALIN, TAPT1, TARBP2, TARS1, TATDN3, TBC1D24, TBC1D30, TBC1D9, TBCA, TBL2, TBR1, TBRG1, TBRG4, TCEA1, TCEAL1, TCEAL2, TCEAL5, TCEAL6, TCEAL7, TCEAL9, TCF3, TCP11L1, TDRD9, TEAD2, TEAD3, TECPR2, TEF, TEFM, TEK, TEN1, TENM2, TENM3, TENT2, TENT5A, TERF2IP, TESPA1, TEX2, TEX29, TFG, TFPI, TGDS, TGFB1I1, TGFBR3, TGFBR3L, TGIF1, TGIF2, THADA, THBS3, THEMIS, THG1L, THOC6, THPO, THRA, THRB, THSD4, TIAM1, TIGD6, TIMM13, TIMM8B, TIMM9, TIMMDC1, TINCR, TINF2, TLCD3B, TLCD4, TLE5, TLE6, TLN2, TM2D3, TM9SF1, TMBIM6, TMCO1, TMED1, TMED10, TMED2, TMED3, TMED4, TMED8, TMED9, TMEFF1, TMEFF2, TMEM101, TMEM107, TMEM11, TMEM115, TMEM120A, TMEM121B, TMEM123, TMEM130, TMEM134, TMEM138, TMEM145, TMEM147, TMEM14A, TMEM150C, TMEM151B, TMEM155, TMEM165, TMEM170B, TMEM179, TMEM179B, TMEM18, TMEM196, TMEM200A, TMEM208, TMEM209, TMEM214, TMEM218, TMEM233, TMEM240, TMEM242, TMEM245, TMEM25, TMEM251, TMEM258, TMEM266, TMEM268, TMEM271, TMEM35A, TMEM38A, TMEM38B, TMEM39A, TMEM51, TMEM59L, TMEM60, TMEM63C, TMEM74, TMEM79, TMEM99, TMOD2, TMTC1, TMUB1, TMX1, TMX4, TNC, TNFAIP6, TNFRSF10B, TNFRSF13C, TNFRSF19, TNIP2, TNK2-AS1, TNNI3K, TNNT1, TOGARAM1, TOLLIP, TOM1L2, TOMM20, TOMM40, TOMM40L, TOMM6, TOR3A, TOX, TOX2, TP53, TP53I13, TP53I3, TPBGL, TPD52, TPD52L1, TPM1-AS, TPM2, TPRA1, TPRG1L, TPRKB, TPT1, TPTE2P1, TRABD2A, TRAM1L1, TRAPPC2B, TRAPPC3, TRAPPC4, TRAPPC6B, TRDC, TRIAP1, TRIM14, TRIM17, TRIM2, TRIM23, TRIM3, TRIM37, TRIM4, TRIM44, TRIM47, TRIM5, TRIM65, TRIOBP, TRIP10, TRIP4, TRIQK, TRMT112, TRMT6, TRMT61A, TRNAU1AP, TRNP1, TRPC1, TRPM7, TRUB1, TSC22D1-AS1, TSEN34, TSKU, TSNARE1, TSPAN10, TSPAN5, TSPAN6, TSPOAP1, TSPOAP1-AS1, TSPYL1, TSPYL2, TSPYL4, TSPYL5, TSSC4, TSTA3, TTBK1, TTBK2, TTC23, TTC27, TTC33, TTC38, TTC7B, TTC9, TTC9B, TTLL4, TTLL7, TUB, TUBA4A, TUBA8, TUBB2A, TUBG2, TULP3, TULP4, TUNAR, TUSC3, TVP23B, TWF1, TWISTNB, TWSG1, TXLNA, TXNDC12, TXNDC17, TYK2, TYRO3, U91328.1, UBA52, UBA6-AS1, UBALD1, UBALD2, UBE2A, UBE2E1, UBE2E2, UBE2H, UBE2J2, UBE2K, UBE2QL1, UBE2V2, UBE2W, UBE4B, UBQLN2, UBR3, UCHL1, UCKL1, UFSP1, UHRF1BP1L, ULK1, UNC13A, UNC45A, UNC5A, UNC5D, UNC79, UNC80, UNKL, UPP2, UQCC3, UQCRB, UQCRC2, URM1, UROS, USHBP1, USP11, USP12, USP27X, USP3, USP32P1, USP35, USP46, USP5, USP51, UTP15, UTP6, UXT, VAMP1, VAMP2, VAMP4, VAPB, VAT1, VAT1L, VDAC3, VEGFB, VIP, VIPR1, VIPR2, VKORC1, VN1R20P, VPS13D, VPS36, VPS53, VRK3, VSNL1, VSTM2A, VSTM2L, VSTM5, VSX1, VTI1A, VTI1B, VWA5B2, VWA7, VWC2, VWC2L, WAC-AS1, WARS2, WASF3, WASIR2, WBP1LP2, WBP2, WBP4, WDFY3-AS2, WDR17, WDR31, WDR37, WDR46, WDR47, WDR53, WDR55, WDR7, WDR83OS, WDTC1, WEE1, WFDC1, WFIKKN1, WFS1, WIF1, WIPF2, WIPF3, WNK2, WNT10B, WNT2B, WNT7A, WSB2, WWC1, WWP1, WWTR1-AS1, XK, XKR4, YAE1, YIF1A, YIPF2, YJEFN3, YKT6, YPEL3, YPEL4, YPEL5, YTHDF1, YWHAB, YWHAG, YWHAH, YWHAZ, Z70719.1, Z83844.2, Z97653.2, Z99129.4, Z99572.1, ZBTB16, ZBTB4, ZBTB42, ZBTB45P1, ZBTB7A, ZBTB8OS, ZC3H13, ZC3H7B, ZC3HAV1, ZC3HC1, ZCCHC12, ZCCHC17, ZCCHC4, ZCCHC9, ZDBF2, ZDHHC4, ZDHHC8P1, ZER1, ZFAND1, ZFAND2A, ZFAND6, ZFAS1, ZFHX2-AS1, ZFP36L2, ZFP92, ZFPL1, ZFR2, ZFYVE27, ZFYVE9, ZMAT4, ZMIZ2, ZNF204P, ZNF230, ZNF25, ZNF280B, ZNF321P, ZNF330, ZNF33A, ZNF33B, ZNF365, ZNF366, ZNF385B, ZNF385D, ZNF394, ZNF408, ZNF410, ZNF423, ZNF483, ZNF503-AS2, ZNF526, ZNF540, ZNF554, ZNF561, ZNF575, ZNF581, ZNF587B, ZNF593, ZNF625-ZNF20, ZNF658B, ZNF667, ZNF672, ZNF691, ZNF701, ZNF702P, ZNF768, ZNF784, ZNF787, ZNF816, ZNF837, ZNF880, ZNHIT1, ZNRD1, ZNRF3, ZPR1, ZRANB1, ZSCAN21, ZSCAN31, ZSWIM4, ZYX | |
| Blue module (ME2) | | A2M, A4GALT, AADAT, AARS1, AARSD1, AASDHPPT, ABAT, ABCA11P, ABCA13, ABCA3, ABCA7, ABCC3, ABCG1, ABHD10, ABHD14A-ACY1, ABHD14B, ABHD15, ABI2, ABI3, ABI3BP, ABRACL, AC002091.2, AC004158.1, AC004264.1, AC004687.1, AC004846.1, AC004847.1, AC004854.2, AC004865.2, AC004921.1, AC005034.5, AC005034.6, AC005082.1, AC005225.4, AC005280.3, AC005332.6, AC005498.3, AC005831.1, AC006027.1, AC006033.2, AC006116.10, AC006130.3, AC006213.6, AC006213.7, AC006449.5, AC006963.1, AC007541.1, AC007786.1, AC007877.1, AC007938.1, AC007938.3, AC008105.3, AC008525.1, AC008567.3, AC008738.7, AC008760.2, AC008915.3, AC008957.1, AC009070.1, AC009407.1, AC009630.4, AC010247.1, AC010247.2, AC010319.1, AC010536.1, AC011446.2, AC011511.5, AC011558.1, AC011899.2, AC012181.2, AC012378.2, AC012513.3, AC012558.1, AC012645.1, AC012645.3, AC015813.6, AC015819.1, AC015909.5, AC015911.10, AC015912.3, AC016582.2, AC016745.1, AC016876.2, AC018529.2, AC018630.2, AC018755.2, AC018809.2, AC019205.1, AC020763.4, AC020915.4, AC020916.1, AC020931.1, AC021016.2, AC022509.1, AC022509.2, AC022613.1, AC022706.1, AC023043.1, AC023480.1, AC023794.3, AC024560.3, AC025580.3, AC027702.1, AC034198.2, AC037198.1, AC037198.2, AC040168.1, AC040970.1, AC046134.2, AC060766.1, AC060766.4, AC060766.7, AC061992.2, AC063977.6, AC067773.1, AC068057.2, AC068580.3, AC068643.1, AC069224.1, AC069368.2, AC073111.4, AC078883.1, AC079776.5, AC083837.1, AC083862.1, AC087203.3, AC087854.1, AC090559.1, AC090625.2, AC091167.4, AC091181.1, AC091271.1, AC091912.3, AC092111.1, AC092111.2, AC092295.2, AC092299.1, AC092329.4, AC092368.3, AC092437.1, AC092667.1, AC092718.8, AC093323.1, AC093458.2, AC093620.1, AC093627.4, AC093895.1, AC096921.2, AC097505.1, AC099063.4, AC099332.1, AC099332.2, AC099518.1, AC099850.1, AC100858.3, AC100861.1, AC104024.4, AC104088.3, AC104530.1, AC104695.2, AC106739.1, AC106820.4, AC106865.1, AC107294.2, AC108047.1, AC108134.3, AC108488.3, AC108673.2, AC110995.1, AC112220.2, AC116347.1, AC116535.1, AC123595.2, AC124067.2, AC124242.1, AC124312.3, AC125611.3, AC126283.2, AC127502.1, AC127502.2, AC130304.1, AC130371.2, AC131097.2, AC132008.2, AC133552.2, AC135983.3, AC136475.3, AC138207.4, AC138207.9, AC138356.1, AC138356.2, AC139495.2, AC139768.1, AC140725.1, AC144652.1, AC144831.1, AC145098.1, AC145098.2, AC147067.1, AC215522.2, AC243960.1, AC243960.3, AC244033.2, AC245407.2, ACACA, ACAD8, ACAP1, ACAP3, ACBD5, ACOT9, ACP2, ACP3, ACP5, ACRBP, ACSL1, ACSL4, ACSL5, ACTB, ACTN1, ACTR6, AD000671.2, ADA2, ADAM15, ADAM28, ADAM8, ADAMDEC1, ADAMTSL4, ADAP2, ADCK2, ADCY7, ADCYAP1R1, ADD1, ADGRB1, ADGRB2, ADGRB3, ADGRE1, ADGRE2, ADGRE3, ADGRE4P, ADGRG1, ADGRG3, ADGRL1, ADIPOR1, ADORA3, ADPGK, ADPRH, ADRB2, AF131216.4, AF274853.1, AF274858.1, AF287957.1, AFG1L, AGPAT2, AGTRAP, AHNAK, AIF1, AIFM2, AIM2, AK9, AKAP7, AKNA, AKR1A1, AKR1B1, AKT3, AL021308.1, AL021392.1, AL022069.1, AL022069.2, AL022069.3, AL023806.1, AL031118.1, AL031595.1, AL031668.2, AL034548.2, AL035252.3, AL035653.1, AL049838.1, AL049839.2, AL050341.2, AL078604.2, AL096865.1, AL121933.2, AL121987.1, AL122035.1, AL122035.2, AL132709.8, AL133415.1, AL133520.1, AL136090.2, AL136116.3, AL136366.1, AL137077.2, AL138724.1, AL139384.1, AL139424.3, AL157392.3, AL157400.5, AL160313.1, AL161785.1, AL162253.2, AL353807.5, AL355297.3, AL355377.2, AL355916.1, AL356215.1, AL356740.1, AL357033.4, AL359198.1, AL359258.3, AL359513.1, AL359643.2, AL359762.1, AL391280.2, AL391807.1, AL391834.2, AL392089.1, AL441992.1, AL512353.1, AL589740.1, AL590764.1, AL596087.2, AL627095.2, AL627309.6, AL627309.7, AL645933.2, AL645933.3, AL645939.2, AL662795.2, AL691432.2, AL713852.2, AL928654.1, ALDH16A1, ALDH1A2, ALDH3A1, ALDH3B1, ALDH5A1, ALDH7A1, ALG1L9P, ALOX15B, ALOX5, ALOX5AP, ALPK1, ALPK2, AMDHD1, AMDHD2, AMPD3, AMZ2P1, ANAPC16, ANG, ANGEL2, ANGPTL6, ANKRD1, ANKRD13B, ANKRD20A2P, ANKRD22, ANKRD26, ANKRD37, ANPEP, ANTKMT, ANXA1, ANXA2, ANXA2P2, ANXA4, AOAH, AOX1, AP000487.1, AP000593.4, AP000648.4, AP000708.1, AP000766.1, AP000866.1, AP001025.1, AP001056.1, AP002807.1, AP002954.1, AP003071.4, AP003419.1, AP003465.1, AP004608.1, AP1B1, AP2B1, AP3S1, AP3S2, APBA2, APBB1IP, APBB2, APC, APC2, APCDD1, APCDD1L, APIP, APOBEC3A, APOBEC3C, APOBEC3D, APOBEC3F, APOBEC3G, APOBEC3H, APOBR, APOC1, APOC1P1, APOC2, APOC4-APOC2, APOE, APOL1, APOL2, APOL3, APOL6, AQP5, AQP6, AQP9, ARAP1, ARC, AREG, ARHGAP10, ARHGAP15, ARHGAP18, ARHGAP21, ARHGAP24, ARHGAP25, ARHGAP27, ARHGAP30, ARHGAP35, ARHGAP39, ARHGAP4, ARHGAP42-AS1, ARHGAP45, ARHGAP9, ARHGDIB, ARID3A, ARL11, ARL14EP, ARL6IP1, ARL6IP5, ARMC8, ARMT1, ARNT2, ARPC1B, ARPC2, ARPC3, ARPC4, ARPC5, ARRB2, ARRDC5, ARSA, ARSI, ARSJ, ASAH1, ASB3, ASB5, ASB8, ASB9, ASCL1, ASCL2, ASF1A, ASGR2, ASIC1, ASIP, ASPRV1, ASRGL1, ASS1, ASTN1, ATF3, ATF5, ATG3, ATG7, ATG9A, ATN1, ATP13A4, ATP13A4-AS1, ATP1B3, ATP2A3, ATP2C2, ATP5F1A, ATP6AP2, ATP6V0B, ATP6V0D2, ATP6V0E2, ATP6V0E2-AS1, ATP6V1E2, ATP8B3, ATP8B4, ATP9B, ATXN2, ATXN7L3B, AVEN, AXIN2, AXL, AZIN1, B2M, B3GALNT1, B3GALT1, B3GALT2, B3GALT4, B3GAT2, B3GNT2, B3GNT8, B4GALNT4, B4GALT1, BAALC, BACE2, BAG3, BAIAP2L1, BAP1, BATF, BATF2, BBS2, BCAN, BCAP31, BCAS3, BCDIN3D-AS1, BCL2A1, BCL3, BDKRB2, BEND5, BET1L, BICC1, BIK, BIN2, BIRC3, BIRC7, BISPR, BLNK, BMF, BMP2K, BMP7, BNC2, BNIP3P1, BOLA3-AS1, BOP1, BRD3OS, BRD7, BRD8, BRD9, BRF1, BRI3, BRINP2, BRK1, BRMS1L, BST1, BST2, BTBD11, BTBD17, BTBD19, BTBD2, BTD, BTG2, BTK, BTN3A1, BTN3A2, BTN3A3, BX640514.2, C10orf55, C11orf21, C11orf45, C11orf49, C12orf29, C12orf60, C12orf73, C15orf48, C16orf54, C17orf100, C17orf113, C18orf32, C19orf38, C1orf162, C1orf21, C1orf54, C1orf61, C1QA, C1QB, C1QC, C1R, C1RL, C1RL-AS1, C1S, C2, C20orf197, C2CD2, C2orf42, C2orf72, C3, C3AR1, C5AR1, C5AR2, C6orf141, C7, C9orf139, CA12, CA14, CACNA2D4, CACNG7, CADM2, CADM4, CAHM, CALCOCO1, CALCOCO2, CALHM2, CALHM6, CALML4, CAMP, CAMSAP2, CAND2, CAPG, CAPZA1, CAPZB, CARD11, CARD16, CARD19, CARD6, CARD8-AS1, CARD9, CARF, CASKIN2, CASP1, CASP10, CASP4, CASP4LP, CASP5, CASP7, CASP8, CASS4, CAST, CASTOR2, CASTOR3, CAT, CATSPER1, CAVIN1, CAVIN3, CBFA2T2, CBLN3, CBR4, CC2D1B, CCDC15-DT, CCDC184, CCDC200, CCDC25, CCDC69, CCDC71L, CCDC85C, CCDC88B, CCL18, CCL2, CCL20, CCL26, CCL3, CCL3L3, CCL4, CCL4L2, CCL5, CCL7, CCL8, CCN1, CCND2, CCNG1, CCR1, CCR2, CCR4, CCR5, CCR6, CCR7, CCRL2, CD109, CD14, CD163, CD163L1, CD180, CD1C, CD1D, CD2, CD200R1, CD209, CD226, CD244, CD247, CD27, CD274, CD28, CD2AP, CD300A, CD300C, CD300E, CD300LB, CD300LF, CD33, CD36, CD37, CD3D, CD3E, CD3G, CD4, CD40, CD40LG, CD44, CD48, CD5, CD52, CD53, CD55, CD6, CD68, CD69, CD7, CD70, CD72, CD74, CD79A, CD79B, CD80, CD81, CD83, CD84, CD86, CD8A, CD8B, CD96, CD99, CD99P1, CDA, CDADC1, CDC42EP1, CDC42EP5, CDCP1, CDH10, CDH20, CDK14, CDKN1A, CDKN1B, CDKN2AIPNL, CEACAM1, CEACAM21, CEACAM4, CEBPA, CEBPB, CEBPD, CEBPE, CEMIP, CEP250-AS1, CEP68, CEROX1, CERS2, CERS4, CES1, CFB, CFD, CFH, CFI, CFLAR, CH25H, CHCHD7, CHDH, CHI3L1, CHI3L2, CHMP1B, CHMP4C, CHRNA6, CHRNA9, CHRNE, CHST10, CHST13, CHST15, CHST2, CHST8, CIAO1, CIB1, CIB2, CIDEB, CIDECP1, CIITA, CISH, CISTR, CITED2, CLASP2, CLCF1, CLCNKB, CLDN1, CLDN23, CLDN4, CLDN7, CLEC10A, CLEC11A, CLEC12A, CLEC17A, CLEC1A, CLEC2B, CLEC4A, CLEC4E, CLEC5A, CLEC7A, CLECL1, CLIC1, CLIC2, CLIP2, CLMP, CLN6, CLNS1A, CLPB, CLUAP1, CMAHP, CMC1, CMKLR1, CMPK2, CMSS1, CMTM2, CMTM7, CNN2, CNNM3, CNPY3, CNTFR, CNTN1, COA5, COL10A1, COL11A2, COL14A1, COL20A1, COL7A1, COL8A1, COL8A2, COL9A2, COL9A3, COLEC12, COLGALT1, COMMD8, COPS8, COQ10B, COQ3, CORO1A, CORO1B, CORO2B, COX20, CP, CPD, CPED1, CPQ, CPT1C, CPVL, CPZ, CR1, CRACR2A, CRACR2B, CRB1, CREG1, CREM, CRIPT, CRISPLD2, CRKL, CRLF3, CRTAM, CRYBB1, CRYBG1, CS, CSF1, CSF1R, CSF2RA, CSF2RB, CSF3, CSF3R, CSGALNACT2, CSK, CSP2, CSPG5, CSRNP1, CSRNP2, CST6, CST7, CSTA, CSTB, CTBS, CTLA4, CTNNBIP1, CTNND2, CTSA, CTSB, CTSC, CTSD, CTSH, CTSK, CTSL, CTSO, CTSS, CTSW, CTSZ, CTTNBP2, CTXND1, CUEDC1, CUL7, CXCL1, CXCL10, CXCL11, CXCL12, CXCL13, CXCL16, CXCL2, CXCL3, CXCL5, CXCL6, CXCL8, CXCL9, CXCR1, CXCR2, CXCR3, CXCR4, CXCR6, CXorf21, CXXC4, CYB561, CYB561A3, CYB561D2, CYB5R1, CYB5R4, CYBA, CYBB, CYBRD1, CYFIP1, CYP19A1, CYP1B1, CYP27A1, CYP2S1, CYRIB, CYSLTR1, CYTH4, CYTIP, DAB2, DAGLA, DANCR, DAPP1, DAZAP2, DBIL5P, DBT, DCBLD2, DCLK2, DCN, DCUN1D2, DCXR-DT, DDAH1, DDHD1, DDIT4, DDR2, DDX1, DDX51, DDX60, DDX60L, DEDD2, DEF6, DEFA1, DENND1C, DENND2D, DENND3, DENND5B, DEPP1, DERL3, DGAT2, DGCR2, DHCR7, DHPS, DHRS3, DHRS4, DHRS4-AS1, DHX30, DHX58, DIAPH2, DIPK1B, DIPK1C, DIS3L, DKK1, DLL1, DMAC2L, DMD, DNAH10OS, DNAJB1, DNAJC18, DNAJC19, DNAJC27-AS1, DNAJC3-DT, DNAJC5B, DNAL1, DNASE1L1, DNASE2, DNM2, DOCK11, DOCK2, DOCK8, DOK1, DOK2, DOK3, DPEP2, DPP4, DPP6, DPY19L2P2, DPY19L2P3, DPYD, DRAM1, DRAM2, DROSHA, DSC2, DSCR9, DSE, DTD2, DTX1, DTX2, DTX2P1, DTX3L, DTX4, DUBR, DUS4L-BCAP29, DUSP1, DUSP2, DUSP23, DUSP27, DUSP28, DYNLL2, DYNLT3, DYSF, DZIP1, EAF2, EBI3, ECHDC3, ECM2, EDEM1, EDEM2, EDNRB, EDRF1-DT, EFCAB7, EFEMP1, EFNA4, EGF, EGFL6, EGLN1, EGR1, EGR2, EHBP1L1, EHD2, EHMT2, EID1, EID2, EID2B, EIF1, EIF3C, EIF3EP1, EIF3J-DT, EIPR1, ELF1, ELF4, ELK3, ELL, ELL2, ELMO2, ELP2, ELP4, EMB, EMILIN3, EML2-AS1, EML3, EML6, EMP3, ENDOG, ENOPH1, ENPP1, EOMES, EPHA1, EPHB4, EPHX3, EPN2, EPOR, EPSTI1, ERI3, ERICH1, ERP29, ERRFI1, ESPNL, ESRRG, ESYT1, ESYT2, ETFBKMT, ETS2, ETV7, EVA1A, EVI2B, EXD2, EXOSC6, F11R, F13A1, F5, FABP4, FADS1, FADS2, FAH, FAM104B, FAM110C, FAM117A, FAM118B, FAM120B, FAM157A, FAM161A, FAM168A, FAM177B, FAM181B, FAM184A, FAM184B, FAM200B, FAM20A, FAM20C, FAM214B, FAM241A, FAM66C, FAM66D, FAM78A, FAM78B, FAM83G, FAM8A1, FAP, FAS, FASLG, FASN, FAT3, FBLN1, FBLN5, FBP1, FBXO21, FBXO32, FBXO45, FBXO6, FBXW11, FCAR, FCER1A, FCER1G, FCGBP, FCGR1A, FCGR1B, FCGR1CP, FCGR2A, FCGR2B, FCGR2C, FCGR3A, FCGR3B, FCGRT, FCMR, FCN1, FCRL6, FER1L4, FERMT3, FES, FFAR2, FFAR3, FFAR4, FGD2, FGD3, FGFBP3, FGL2, FGR, FHL2, FHOD1, FIZ1, FKBP11, FKBP15, FKBP4, FLI1, FLII, FLJ16779, FLNB, FLNC, FLOT1, FLVCR2, FMN2, FMNL1, FMO3, FMO4, FNDC1, FOLR2, FOS, FOSB, FOSL1, FOSL2, FOXC1, FOXK2, FOXN3-AS1, FOXO3, FOXP3, FOXRED2, FP565260.3, FPR1, FPR2, FPR3, FRMPD1, FRRS1, FRS2, FSTL3, FTH1, FTH1P10, FTH1P11, FTH1P2, FTH1P20, FTH1P7, FTH1P8, FTL, FTLP3, FTO, FUCA1, FUCA2, FUOM, FUT4, FUT7, FXYD5, FYB1, FYN, FZD7, G0S2, G6PD, GAA, GAB3, GADD45A, GADD45B, GAL3ST2, GAL3ST3, GALC, GALM, GALNT12, GALNT4, GALNT5, GALNT6, GAN, GAPLINC, GAPT, GAREM2, GAS6, GAS6-AS1, GASK1B, GATA6, GBA, GBA2, GBGT1, GBP1, GBP1P1, GBP2, GBP3, GBP4, GBP5, GCA, GCAT, GCDH, GCH1, GCKR, GCLM, GCNT1, GCSH, GDF11, GDF15, GDF5, GDF9, GEM, GEMIN8P4, GFPT2, GGA2, GGT5, GGTA1P, GHRL, GID4, GIMAP1, GIMAP2, GIMAP4, GIMAP5, GIMAP6, GIMAP7, GIMAP8, GIN1, GJD3, GK, GKAP1, GLB1, GLIPR1, GLMP, GLRX, GLT8D2, GM2A, GMDS-DT, GMFG, GMIP, GNA15, GNAS, GNG10, GNG11, GNGT2, GNL1, GNLY, GNPDA2, GNRHR2, GNS, GOLGA2P7, GOLGA8H, GPAT3, GPBAR1, GPBP1, GPBP1L1, GPHN, GPM6A, GPM6B, GPNMB, GPR132, GPR141, GPR157, GPR160, GPR171, GPR174, GPR18, GPR183, GPR34, GPR37L1, GPR65, GPR82, GPR84, GPRC5B, GPRIN3, GPS1, GPSM3, GPT2, GPX1, GPX1P1, GPX3, GPX8, GRAP2, GRB2, GRIA3, GRIA4, GRIK1, GRIK4, GRIK5, GRK4, GRN, GRSF1, GSAP, GSDMA, GSDMD, GSG1L, GSN, GTF2IP1, GTF2IP4, GTSF1, GUCY1A2, GUF1, GUSBP5, GVINP1, GXYLT2, GYPC, GZMA, GZMB, GZMH, GZMK, GZMM, H1-2, H2AC18, H2AC6, H2AJ, H2AZ2, H2BC12, HAAO, HACD4, HACE1, HAMP, HAPLN3, HARS1, HAS1, HAUS4, HAVCR2, HBEGF, HCAR2, HCAR3, HCG17, HCK, HCLS1, HCP5, HCST, HDGFL3, HDHD2, HEPACAM, HERPUD1, HES5, HES6, HEXA, HEXA-AS1, HEXB, HFE, HGD, HGF, HHEX, HIBCH, HIF1A-AS3, HIP1R, HIPK3, HIRIP3, HK3, HLA-A, HLA-B, HLA-C, HLA-DMA, HLA-DMB, HLA-DOA, HLA-DOB, HLA-DPA1, HLA-DPB1, HLA-DPB2, HLA-DQA1, HLA-DQA2, HLA-DQB1, HLA-DRA, HLA-DRB1, HLA-E, HLA-F, HLX, HMGCL, HMGCR, HMGCS1, HMGN5, HMOX1, HNRNPUL2, HOMER3-AS1, HOXB6, HP, HPD, HPDL, HPGDS, HPS5, HPSE, HR, HRH1, HS1BP3, HSCB, HSD17B11, HSD3B7, HSF2, HSH2D, HSPA1A, HSPA1B, HSPA6, HSPA7, HTATIP2, HTATSF1, HTR2B, HTR7, HTRA4, HUNK, ICA1L, ICAM1, ICAM3, ICAM4, ICOS, ICOSLG, ID1, ID3, IDO1, IER2, IER3, IER3-AS1, IFI27, IFI30, IFI35, IFI44, IFIH1, IFIT2, IFIT3, IFITM1, IFITM2, IFITM3, IFNGR1, IFNGR2, IFNLR1, IGDCC3, IGF1, IGFBP1, IGFBP6, IGFLR1, IGHA1, IGHA2, IGHG1, IGHG2, IGHG3, IGHG4, IGHM, IGKC, IGKV1-5, IGKV1D-39, IGKV3-11, IGKV3-15, IGKV3-20, IGKV4-1, IGLC1, IGLC2, IGLC3, IGLL5, IGLON5, IGLV1-40, IGLV1-44, IGLV2-14, IGLV2-8, IGSF11, IGSF6, IKBKE, IKZF1, IKZF3, IL10, IL10RA, IL10RB, IL10RB-DT, IL11, IL12RB1, IL13RA1, IL15, IL15RA, IL16, IL17RA, IL17RB, IL18, IL18BP, IL18R1, IL18RAP, IL1A, IL1B, IL1R1, IL1R2, IL1RN, IL21R, IL23A, IL2RA, IL2RB, IL2RG, IL32, IL3RA, IL4I1, IL4R, IL6, IL6R, IL7, IL7R, IMP4, IMPA2, IMPDH1, INAFM1, INAFM2, INHBA, INMT, INPP5D, INSRR, INSYN1, INSYN1-AS1, IPO5P1, IQGAP1, IRAG2, IRAK2, IRAK3, IRF1, IRF1-AS1, IRF2, IRF5, IRF7, IRF8, IRF9, ISG20, ISLR, ITGA11, ITGA2, ITGAL, ITGAM, ITGAX, ITGB2, ITGB2-AS1, ITGB4, ITGB7, ITGBL1, ITK, ITM2C, ITPKC, ITPRIP, ITSN2, JADE1, JAK2, JAK3, JAKMIP2, JAML, JCHAIN, JMY, JPH2, JSRP1, JUN, JUNB, KANK1, KANK2, KAT14, KAT2B, KAT6B, KATNB1, KBTBD6, KBTBD7, KCNAB3, KCNE4, KCNE5, KCNF1, KCNIP1, KCNJ10, KCNJ16, KCNJ5, KCNK13, KCNK15, KCNK6, KCNMB1, KCNMB2, KCNN4, KCNQ1, KCP, KCTD1, KCTD11, KCTD12, KCTD9, KDM4D, KHDC1, KHDC1L, KIAA0232, KIAA1191, KIAA1328, KIAA1614, KIF19, KIF1B, KIF1C-AS1, KIF21A, KIF2A, KIF9-AS1, KIRREL2, KIZ, KLC4, KLF10, KLF11, KLF12, KLF15, KLF2, KLF4, KLF5, KLHDC7B, KLHDC7B-DT, KLHL21, KLHL22, KLHL42, KLHL6, KLRB1, KMO, KPNA3, KPNA5, KRT18, KRT7, KRT80, KYAT1, KYNU, L2HGDH, L3MBTL4-AS1, LACTB, LAG3, LAIR1, LAMC2, LAMP1, LAMP3, LAMTOR1, LAP3, LAPTM5, LAT2, LATS2, LAYN, LCK, LCMT1, LCP1, LCP2, LCTL, LDHB, LDLRAD2, LEF1, LENG9, LEPROT, LGALS1, LGALS12, LGALS2, LGALS3, LGALS9, LGALSL, LGMN, LHB, LHFPL2, LIF, LIFR-AS1, LILRA1, LILRA2, LILRA5, LILRA6, LILRB1, LILRB2, LILRB3, LILRB4, LILRB5, LIMK2, LIMS1, LIN52, LINC00324, LINC00327, LINC00339, LINC00528, LINC00601, LINC00606, LINC00888, LINC00909, LINC00945, LINC01003, LINC01094, LINC01102, LINC01127, LINC01137, LINC01150, LINC01159, LINC01238, LINC01270, LINC01278, LINC01322, LINC01426, LINC01503, LINC01504, LINC01535, LINC01678, LINC01852, LINC01857, LINC01896, LINC01907, LINC01914, LINC01943, LINC01948, LINC01963, LINC02084, LINC02285, LINC02449, LINC02541, LINC02611, LINC02696, LINC02732, LINC02773, LINC02828, LIPA, LITAF, LKAAEAR1, LMCD1-AS1, LMF1, LMNTD2, LNCAROD, LNCOG, LNCTAM34A, LNP1, LOXL4, LPAR5, LPAR6, LPCAT2, LPXN, LRATD1, LRG1, LRIG3, LRMDA, LRP10, LRP1B, LRP3, LRP4, LRP4-AS1, LRP8, LRRC24, LRRC25, LRRC4, LRRC47, LRRC49, LRRC4B, LRRC75A, LRRFIP1, LRRK1, LRRK2-DT, LRRN2, LRRN4CL, LRRTM3, LSAMP, LSP1, LST1, LTA, LTA4H, LTB, LTBP1, LTBP2, LTBR, LTC4S, LTF, LUCAT1, LY75, LY86, LY96, LYL1, LYN, LYPD3, LYPD6, LYRM7, LYSMD1, LYVE1, LYZ, M6PR, MACC1, MAEL, MAF, MAFB, MAFK, MAGED1, MAGED2, MAGED4, MAGED4B, MAGI1, MAGI2, MAGI3, MALL, MAML3, MAN1A1, MAN1C1, MAN2A1, MAN2B1, MAN2B2, MANBA, MANEAL, MANSC1, MAP2K3, MAP2K6, MAP3K11, MAP3K6, MAP3K8, MAP4K1, MAPK13, MAPKAPK3, MAPT, MAPT-AS1, MARCHF3, MARCO, MARK1, MATR3, MAX, MBD2, MBD4, MBNL1, MBOAT1, MBOAT2, MCEMP1, MCF2L-AS1, MCMDC2, MCOLN1, MCOLN2, MCPH1-AS1, MCUB, MCUR1, MDFIC, MED10, MED12L, MED8, MED9, MEDAG, MEFV, MEI1, MEIS1, MELTF, MERTK, MESTIT1, MET, METRN, METRNL, METTL24, METTL27, METTL9, MFAP4, MFAP5, MFF, MFNG, MFSD1, MFSD12, MGAM, MGAT1, MGAT4A, MGP, MGST1, MGST2, MIA2, MICA, MICB, MILR1, MIR1-1HG-AS1, MIR155HG, MIR222HG, MIR223HG, MIR22HG, MIR762HG, MKKS, MKNK1, MKNK2, MKRN2, MLF2, MLKL, MLPH, MMD2, MME, MMP1, MMP10, MMP19, MMP7, MNDA, MOB3C, MOCOS, MON1A, MORN4, MPEG1, MPP1, MPP5, MPPED2, MPPED2-AS1, MPV17L, MPZL3, MR1, MRC1, MREG, MRFAP1, MRFAP1L1, MRGPRF, MROH8, MRPL35, MRPL38, MRPS14, MRPS23, MRPS27, MRPS31P5, MRPS9, MS4A14, MS4A4A, MS4A4E, MS4A6A, MS4A7, MSANTD3-TMEFF1, MSANTD4, MSC, MSR1, MT1H, MT1L, MT1X, MT2A, MTARC1, MTERF4, MTHFS, MTMR14, MTSS2, MUC1, MUC12-AS1, MVK, MVP, MX1, MX2, MXD1, MXRA8, MYADM, MYBPH, MYEF2, MYH7, MYL12A, MYL12B, MYL4, MYL6B, MYLIP, MYO10, MYO16, MYO1E, MYO1F, MYO1G, MYO6, MYO7A, MZB1, N6AMT1, NAAA, NAALADL1, NABP1, NAGA, NAGK, NAGS, NAIP, NAIPP1, NAMPT, NAMPTP1, NANOS3, NANS, NAP1L4, NAPEPLD, NAPSB, NAT1, NAXD, NBEAL2, NCAM2, NCAN, NCF1, NCF1B, NCF1C, NCF2, NCF4, NCKAP1L, NCOA1, NDRG1, NDUFAF6, NDUFS1, NEAT1, NECTIN2, NEO1, NEU1, NEU4, NEURL3, NEXN, NFAM1, NFATC2, NFE2, NFE2L1, NFE2L3, NFIA, NFIX, NFKB1, NFKB2, NFKBIA, NFKBID, NFKBIE, NFKBIZ, NFYB, NGF, NHLRC4, NIBAN1, NIBAN2, NIF3L1, NIM1K, NINJ1, NKAIN3, NKAIN4, NKG7, NKX2-2, NKX3-1, NLGN3, NLRC4, NLRC5, NLRP1, NLRP12, NLRP3, NME1-NME2, NMI, NMNAT1, NNMT, NNT, NNT-AS1, NOD1, NOD2, NOLC1, NOP10, NOP14-AS1, NOVA1, NPAS3, NPC2, NPL, NPRL3, NQO1, NQO2, NR1H3, NR4A2, NR4A3, NRBF2, NREP, NRG2, NRP2, NRROS, NRXN2, NT5C3B, NT5M, NTAN1, NTHL1, NTRK3, NUAK2, NUCB1, NUCKS1, NUDT12, NUDT16P1, NUMB, NUPR1, NUTM2B, NXPH3, OAF, OARD1, OAS1, OAS2, OASL, OBI1, OCIAD2, ODC1-DT, ODF3B, OLFM2, OLFML3, OLIG1, OLIG2, OLR1, OPHN1, OPN3, OR2I1P, OR52K3P, ORC4, OSBPL6, OSCAR, OSER1-DT, OSGEPL1-AS1, OSGIN1, OSM, OSTF1, OTOGL, OTUD1, OXCT1-AS1, P2RX1, P2RX4, P2RX5-TAX1BP3, P2RY10, P2RY13, P2RY6, P2RY8, P3H2, P4HA3, PABPC4, PACRGL, PAFAH1B2, PALMD, PAN3-AS1, PANK1, PANK4, PAPLN, PAPPA, PAPSS2, PARP10, PARP12, PARP14, PARP15, PARP2, PARP9, PARTICL, PARVG, PATL2, PAX8, PBX1, PCBP4, PCCB, PCDH10, PCDH19, PCDH9, PCDHA3, PCDHGC3, PCED1B, PCED1B-AS1, PCK2, PCNT, PCNX2, PCTP, PCYT1B, PDCD1, PDCD10, PDCD1LG2, PDCD6IPP2, PDE2A-AS2, PDE4B, PDE6D, PDE7B, PDE9A, PDGFRL, PDLIM2, PDP2, PDPN, PDZK1IP1, PEAK3, PELATON, PELI2, PELP1, PEMT, PERP, PEX5, PFKFB3, PFKL, PFKM, PHACTR3, PHAX, PHF10, PHF11, PHF14, PHGDH, PHLDA3, PHLDB1, PHLPP1, PHOSPHO1, PI3, PI4K2A, PICALM, PID1, PIEZO1, PIGB, PIGT, PIGX, PIK3AP1, PIK3C2B, PIK3C3, PIK3CD, PIK3CG, PIK3R5, PIK3R6, PILRA, PIM1, PIM2, PIM3, PITPNC1, PKM, PLA2G15, PLA2G2A, PLAAT4, PLAC8, PLAGL1, PLAU, PLAUR, PLB1, PLBD1, PLBD2, PLCB2, PLCG2, PLD2, PLD5, PLEK, PLEKHA3P1, PLEKHF1, PLEKHO1, PLEKHO2, PLIN2, PLIN3, PLK3, PLP2, PLPP3, PLPP7, PLS3, PLSCR1, PLTP, PLXDC2, PLXNB1, PLXNC1, PMAIP1, PML, PMS2P10, PNMA1, PNPLA2, PNRC1, PODNL1, PODXL2, POLD4, POLE4, POLR3B, POM121L9P, POMGNT2, POMT2, PORCN, POU2F2, POU3F3, POU6F1, PPARD, PPARG, PPARGC1A, PPBP, PPCDC, PPCS, PPIC, PPIF, PPM1B, PPM1M, PPM1N, PPME1, PPP1R14B-AS1, PPP1R15A, PPP1R26, PPP2R1A, PPP2R2B, PPP2R5C, PPT1, PRAM1, PRDM1, PRDX2, PRF1, PRKAA2, PRKCA, PRKCD, PRKCH, PRKCZ-AS1, PROCR, PROK2, PROS1, PRPS2, PRR13, PRR13P5, PRR15, PRR33, PRRG4, PRRX2, PRSS23, PRSS36, PRTFDC1, PSAP, PSAT1, PSD4, PSIP1, PSMA1, PSMA5, PSMB10, PSMB8, PSMB8-AS1, PSMB9, PSME2P2, PSME3IP1, PSMG2, PSTPIP1, PSTPIP2, PTAFR, PTCD3, PTCH1, PTCRA, PTGER1, PTGER2, PTGER4, PTGES, PTGES3L, PTGES3P1, PTGFR, PTGR1, PTGS1, PTGS2, PTK2, PTP4A2, PTPN11, PTPN18, PTPN2, PTPN22, PTPN6, PTPN7, PTPRA, PTPRC, PTPRCAP, PTPRD, PTPRD-AS1, PTPRN2-AS1, PTX3, PURG, PVRIG, PWWP3A, PYCARD, QKI, RAB11B, RAB11FIP1, RAB11FIP3, RAB13, RAB20, RAB27A, RAB29, RAB30, RAB32, RAB38, RAB39B, RAB3IL1, RAB42, RAB43, RAB43P1, RAB5B, RAC2, RAD1, RADIL, RALGAPA1, RALGPS1, RAMP2-AS1, RAMP3, RAP1A, RAP2B, RARRES1, RARRES2, RASAL3, RASGRP4, RASL10B, RASSF1, RASSF3, RASSF4, RASSF5, RBM26, RBM47, RBP5, RBPMS, RBPMS-AS1, RBSN, RCAN3, RCBTB1, RCCD1, RCHY1, RCN3, RCSD1, RDH10, RDH11, REEP4, REL, RELB, RELT, RENBP, REPS1, RESF1, RETN, REXO2, RFTN1, RFTN2, RFX8, RFXAP, RGL3, RGS1, RGS10, RGS12, RGS16, RGS18, RGS19, RGS2, RHBDF2, RHBDL3, RHOBTB3, RHOD, RHOG, RHOH, RHOT1, RIC3, RILPL2, RIN3, RIPK3, RLBP1, RMDN2, RMND5A, RMND5B, RNASE1, RNASE2, RNASE3, RNASE4, RNASE6, RNASET2, RNF135, RNF144B, RNF146, RNF149, RNF157, RNF157-AS1, RNF166, RNF181, RNF187, RNF19B, RNF213, RNF217, RNF8, RNFT2, RNH1, RNPEPL1, ROBO2, ROR2, RPL7P38, RPLP0P2, RPP38-DT, RPRD1A, RPS6KA1, RPSAP41, RRAD, RRAS, RREB1, RSAD2, RSRC1, RTF1, RTL5, RTP4, RUFY3, RUNDC1, RUNX2, RUNX3, RWDD2A, S100A10, S100A11, S100A12, S100A4, S100A6, S100A8, S100A9, S100P, S100Z, S1PR4, SAA1, SAA2, SALL2, SAMD10, SAMD13, SAMHD1, SAMSN1, SASH1, SASH3, SAT1, SATB2, SATB2-AS1, SBNO2, SCAMP2, SCARF1, SCD5, SCG3, SCHIP1, SCIMP, SCIN, SCNN1B, SCO2, SCPEP1, SCRG1, SCRIB, SCRN1, SDC2, SDC3, SDCBP2, SDF2L1, SDS, SDSL, SECTM1, SELE, SELL, SELPLG, SEMA3C, SEMA4G, SEMA6A-AS2, SEMA6D, SENP2, SENP8, SEPHS1, SEPHS1P1, SEPTIN1, SEPTIN7, SERF1B, SERF2, SERINC2, SERP1, SERPINA1, SERPINA3, SERPINA5, SERPINB1, SERPINB9, SERPINE1, SERPINF1, SERPINF2, SERPING1, SERTAD1, SERTAD3, SESN3, SESTD1, SEZ6, SFMBT2, SFRP2, SFRP4, SFT2D1, SFXN1, SGK1, SGMS2, SGTA, SH2D1A, SH2D2A, SH3BGRL3, SH3BP1, SH3D21, SH3TC1, SHB, SHISA6, SHISAL2A, SHKBP1, SHMT1, SHROOM2, SIGLEC1, SIGLEC10, SIGLEC11, SIGLEC12, SIGLEC16, SIGLEC22P, SIGLEC5, SIGLEC7, SIGLEC9, SIK1B, SIPA1, SIRPB1, SIRPB2, SIRPG, SIT1, SIX5, SKAP1, SKAP2, SKIDA1, SLA, SLA2, SLAMF1, SLAMF6, SLAMF7, SLAMF8, SLAMF9, SLC10A3, SLC11A1, SLC12A7, SLC15A3, SLC16A10, SLC16A3, SLC16A5, SLC16A6, SLC16A9, SLC17A8, SLC17A9, SLC18B1, SLC1A5, SLC20A1, SLC22A18, SLC22A18AS, SLC22A4, SLC24A3, SLC25A19, SLC25A30, SLC25A37, SLC25A43, SLC25A44, SLC27A6, SLC29A1, SLC29A3, SLC29A4, SLC2A3, SLC2A5, SLC2A9, SLC35E2A, SLC35G1, SLC37A2, SLC38A3, SLC38A6, SLC39A13, SLC39A14, SLC39A8, SLC43A2, SLC49A3, SLC4A8, SLC50A1, SLC66A3, SLC6A11, SLC6A6, SLC7A7, SLC7A8, SLC8B1, SLC9A9, SLC9B1, SLCO2B1, SLCO4A1, SLFN11, SLFN12, SLFN13, SLFN5, SLITRK3, SLN, SLPI, SMAD6, SMAD7, SMAD9, SMAP1, SMARCC2, SMARCD2, SMCO4, SMIM12, SMIM2-AS1, SMIM3, SMIM8, SMPDL3A, SMYD5, SNAI1, SNAI3, SNAP23, SNED1, SNN, SNRK, SNRNP25, SNX11, SNX15, SNX2, SNX20, SNX6, SNX9, SOBP, SOCS1, SOCS3, SOD2, SOD2-OT1, SOD3, SOS1-IT1, SOWAHD, SOX21-AS1, SOX8, SP100, SP110, SP140, SP140L, SP6, SPECC1, SPHK1, SPHK2, SPI1, SPIN2B, SPINT1, SPIRE1, SPN, SPNS3, SPOCD1, SPON1, SPOP, SPP1, SPR, SPRED3, SPTAN1, SPTLC3, SQOR, SQSTM1, SREBF2-AS1, SRGAP3, SRGN, SRPK2, SRPX2, SRR, SRSF12, SRSF8, SRXN1, SS18L1, SSC5D, SSH1, ST14, ST3GAL1, ST3GAL3, ST6GALNAC4, ST8SIA4, STAB1, STAC3, STAP1, STARD5, STARD8, STAT2, STAT5A, STAT6, STC1, STEAP1, STEAP3, STEAP4, STING1, STK17B, STK25, STK26, STN1, STOM, STRA6, STRAP, STRBP, STX11, STX3, STX4, STXBP2, SUB1, SUCLG1, SUCNR1, SUDS3, SUGT1, SULF1, SULT1B1, SULT1C2, SUMF1, SUOX, SUPT3H, SUSD3, SUSD6, SVBP, SVEP1, SWAP70, SWSAP1, SYK, SYNGR2, SYNPO, SYPL1, SYT17, SYTL1, SYTL2, SYTL3, TACC2, TACSTD2, TADA1, TADA2A, TAGAP, TAGLN2, TAOK2, TAOK3, TAP1, TAP2, TAPBP, TAPBPL, TAX1BP3, TBC1D1, TBC1D10A, TBC1D10C, TBC1D14, TBC1D2, TBC1D22A, TBC1D2B, TBC1D8, TBC1D8B, TBCD, TBCEL-TECTA, TBKBP1, TBXAS1, TC2N, TCF7L2, TCIM, TCIRG1, TCN2, TDO2, TDP2, TEC, TECTA, TENT5C, TEP1, TERF1, TFAP2C, TFE3, TFEC, TFPI2, TGFB1, TGFBI, TGFBR1, TGFBR2, TGM2, THAP10, THBD, THBS1, THEMIS2, THNSL1, THOP1, THSD7A, THTPA, THYN1, TICAM1, TICAM2, TIFA, TIGD3, TIGIT, TIMD4, TIMM21, TIMP1, TIMP4, TINAGL1, TIPARP, TJP1, TKT, TLCD2, TLDC2, TLE2, TLN1, TLNRD1, TLR1, TLR2, TLR3, TLR4, TLR5, TLR6, TLR7, TLR8, TM2D2, TM4SF19, TM6SF1, TMBIM1, TMBIM4, TMC6, TMC8, TMEM102, TMEM106A, TMEM108, TMEM109, TMEM119, TMEM140, TMEM14C, TMEM150A, TMEM150B, TMEM154, TMEM156, TMEM171, TMEM176A, TMEM176B, TMEM177, TMEM178B, TMEM198, TMEM200B, TMEM201, TMEM237, TMEM243, TMEM254-AS1, TMEM255B, TMEM26, TMEM26-AS1, TMEM35B, TMEM37, TMEM50A, TMEM52B, TMEM59, TMEM70, TMEM71, TMEM86A, TMEM87B, TMEM8B, TMEM9, TMEM94, TMIGD3, TMPRSS5, TMSB10, TMSB4X, TNF, TNFAIP2, TNFAIP3, TNFAIP8, TNFAIP8L2, TNFAIP8L3, TNFRSF10A, TNFRSF10A-AS1, TNFRSF10C, TNFRSF10D, TNFRSF11A, TNFRSF11B, TNFRSF12A, TNFRSF14, TNFRSF14-AS1, TNFRSF1A, TNFRSF1B, TNFRSF4, TNFRSF6B, TNFRSF8, TNFSF10, TNFSF13, TNFSF13B, TNFSF14, TNFSF15, TNFSF4, TNFSF8, TNIK, TNIP1, TNK2, TNNI2, TOB1-AS1, TOM1, TOMM70, TOR4A, TP53BP2, TPD52L2, TPI1P1, TPK1, TPM3, TPP1, TPRG1-AS1, TPSAB1, TPST2, TPTEP2-CSNK1E, TRAC, TRADD, TRAF1, TRAF3IP2-AS1, TRAF3IP3, TRAK1, TRAP1, TRAPPC12, TRAPPC13, TRAPPC9, TRAT1, TRBC1, TRBC2, TRBV19, TRBV20-1, TRBV28, TRBV29-1, TREM1, TREM2, TREML1, TREML2, TREML3P, TRG-AS1, TRGV3, TRGV5, TRGV7, TRIB1, TRIL, TRIM13, TRIM21, TRIM22, TRIM34, TRIM38, TRIM52-AS1, TRIM56, TRIM9, TRMT9B, TRPM2, TRPM4, TRPV2, TRPV4, TRUB2, TSBP1-AS1, TSC22D1, TSEN2, TSLP, TSPAN1, TSPAN2, TSPAN3, TSPAN32, TSPAN4, TSPAN7, TSPO, TSPY26P, TTC19, TTC7A, TTLL1, TTYH1, TUBA1C, TUBB2B, TUBGCP4, TWF2, TWIST1, TWIST2, TXN, TXNIP, TXNL1, TYMP, TYROBP, U62317.3, UAP1, UBA7, UBD, UBE2B, UBE2D3, UBE2F, UBE2L6, UBE2N, UBE2Z, UBE3D, UBL3, UBQLN4, UBTD1, UCHL5, UCP2, UGCG, ULK4P1, ULK4P2, UNC13D, UNC93B1, UNK, UPP1, USP2, USP20, USP30, USP30-AS1, USP43, USP46-DT, USP47, UTP14C, UTS2, VAMP3, VAMP5, VAMP8, VASN, VASP, VAV1, VCAM1, VDR, VEGFC, VENTX, VIM, VIM-AS1, VMO1, VMP1, VNN1, VNN2, VPS13B-DT, VPS18, VPS26B, VPS37C, VPS37D, VPS39, VPS4A, VPS52, VSIG4, VSIR, VSTM1, VTA1, VWA5A, VWA8, VXN, WARS1, WAS, WASF2, WBP1L, WDFY4, WDR45P1, WDR48, WDR81, WFIKKN2, WIPF1, WIPI1, WNT3, WNT5A, WNT5A-AS1, WRNIP1, WSCD1, WTAP, WWOX, WWTR1, XAF1, XBP1, XCL1, XCL2, YAF2, YBX3, YIPF1, YTHDC1, YWHAE, Z95114.3, Z95115.1, Z97989.1, Z98257.1, ZAP70, ZBED3, ZBP1, ZBTB18, ZBTB7B, ZC3H12A, ZC3H12D, ZC3H8, ZCCHC18, ZDHHC12, ZDHHC17, ZDHHC18, ZFHX2, ZFP1, ZFP2, ZFP36, ZFP36L1, ZFP41, ZFR, ZHX3, ZMIZ1-AS1, ZMYM2, ZMYND11, ZMYND15, ZMYND8, ZNF138, ZNF185, ZNF213, ZNF217, ZNF233, ZNF267, ZNF287, ZNF34, ZNF346, ZNF37BP, ZNF382, ZNF415, ZNF418, ZNF428, ZNF48, ZNF503, ZNF534, ZNF600, ZNF603P, ZNF610, ZNF622, ZNF652, ZNF664, ZNF667-AS1, ZNF683, ZNF709, ZNF771, ZNF775, ZNF793-AS1, ZNF833P, ZNF835, ZNF84, ZNF843, ZNF853, ZNF888, ZNFX1, ZNRF2, ZSCAN1, ZSCAN18, ZSCAN26 | |
| Brown module (ME3) | | A1BG, AARS2, AASDH, ABCB6, ABCB7, ABCC4, ABCD3, ABCE1, ABHD13, ABHD17A, ABHD2, ABITRAM, ABL2, ABRAXAS1, ABRAXAS2, ABTB1, AC000120.4, AC000123.3, AC002094.1, AC002310.5, AC002350.2, AC003005.1, AC003005.2, AC003086.1, AC004461.3, AC004492.1, AC004706.3, AC004771.2, AC004877.1, AC004908.1, AC004918.3, AC005261.5, AC005280.2, AC005523.2, AC005562.1, AC005618.1, AC005670.3, AC005998.1, AC006001.4, AC006213.2, AC006213.3, AC006504.5, AC007191.1, AC007192.2, AC007255.1, AC007365.1, AC007996.1, AC008115.3, AC008119.1, AC008522.1, AC008764.2, AC008969.1, AC008993.1, AC008993.2, AC009053.1, AC009220.3, AC009237.3, AC009630.1, AC009812.3, AC010435.1, AC010507.1, AC010522.1, AC010615.1, AC010834.3, AC011451.2, AC011451.3, AC011477.2, AC011815.3, AC012313.4, AC012313.5, AC012618.3, AC012676.1, AC015813.5, AC015813.7, AC015818.10, AC016722.2, AC018635.1, AC018638.4, AC018647.2, AC018754.1, AC020763.3, AC020910.6, AC021087.3, AC021092.1, AC021106.1, AC021106.3, AC021739.2, AC022107.1, AC022137.4, AC022415.2, AC022506.2, AC023509.1, AC024075.1, AC024588.1, AC025423.2, AC025917.1, AC026202.3, AC026254.2, AC026310.3, AC026412.3, AC026464.4, AC027271.1, AC027644.3, AC034231.1, AC036108.2, AC037459.2, AC037459.4, AC044860.1, AC053513.2, AC055811.4, AC064807.1, AC067750.1, AC067968.1, AC068620.1, AC068790.2, AC068790.3, AC069288.1, AC072022.2, AC073569.3, AC074138.1, AC074194.1, AC079385.3, AC079416.2, AC079921.2, AC084337.1, AC090114.3, AC090181.2, AC090181.3, AC090948.2, AC090971.2, AC091057.3, AC091057.4, AC091167.1, AC091551.1, AC092171.1, AC092171.2, AC092645.1, AC092647.5, AC092718.5, AC092835.1, AC092881.1, AC092919.2, AC093010.2, AC093525.10, AC093525.4, AC093525.7, AC093525.9, AC093827.5, AC097448.1, AC100821.1, AC103923.1, AC104066.5, AC104447.1, AC104596.1, AC104794.3, AC105036.3, AC105206.2, AC106782.1, AC106820.5, AC108010.1, AC108449.2, AC108488.1, AC108673.3, AC108704.2, AC112128.1, AC112484.5, AC112722.1, AC115223.1, AC116158.3, AC117464.1, AC117503.4, AC118344.1, AC118553.2, AC122129.1, AC126755.1, AC131392.1, AC133555.6, AC135050.5, AC138035.1, AC138207.8, AC138393.3, AC138866.1, AC138866.2, AC138932.1, AC139100.1, AC141586.1, AC211429.1, AC211476.6, AC231981.1, AC234775.3, AC234782.1, AC243919.2, AC245140.2, ACADS, ACAP2, ACBD3, ACER2, ACER3, ACO1, ACOX1, ACSL3, ACTG1P1, ACTG1P10, ACTG1P20, ACTR8, ACTRT3, ADAM17, ADAR, ADAT1, ADCY6, ADCY9, ADGRA3, ADK, ADM5, ADNP, ADNP2, AF111169.3, AF131215.5, AFF1, AFF4, AFTPH, AGBL3, AGFG1, AGGF1, AGL, AGO1, AGO2, AGO3, AGO4, AGPAT1, AGPAT5, AGPS, AHCTF1, AHDC1, AIDA, AIP, AKAP1, AKAP13, AKAP9, AKIRIN1, AL008707.1, AL020996.1, AL021368.4, AL021937.1, AL021997.3, AL031670.1, AL031708.1, AL035413.1, AL049777.2, AL049844.2, AL049871.1, AL109614.1, AL109918.1, AL117339.4, AL117340.1, AL117344.2, AL121594.1, AL121845.2, AL121900.1, AL132800.1, AL133227.1, AL133243.2, AL133352.1, AL133353.2, AL133523.1, AL135905.2, AL136419.3, AL137058.2, AL139274.2, AL139287.1, AL157902.2, AL157935.1, AL162595.1, AL353759.1, AL354718.1, AL355075.2, AL355574.1, AL356273.3, AL357079.3, AL358472.6, AL390198.1, AL390719.1, AL390728.4, AL391121.1, AL442003.1, AL442067.3, AL445222.2, AL445288.1, AL445309.1, AL445423.1, AL445423.3, AL445524.1, AL512625.1, AL589993.1, AL590399.1, AL590399.4, AL591684.2, AL604028.2, AL669830.2, AL731577.2, AL844908.1, ALDH1L2, ALDH3A2, ALDOA, ALG10, ALG10B, ALG11, ALKBH6, ALKBH7, ALKBH8, ALMS1, ALS2, AMBRA1, AMER1, AMFR, AMOTL1, ANAPC1, ANAPC15, ANKAR, ANKFY1, ANKHD1, ANKIB1, ANKMY2, ANKRD10-IT1, ANKRD11, ANKRD12, ANKRD13C, ANKRD17, ANKRD18EP, ANKRD20A4P, ANKRD27, ANKRD28, ANKRD36C, ANKRD49, ANKRD49P1, ANKRD50, ANKRD52, ANKRD61, ANKS6, ANO8, ANTXR1, AP000560.1, AP001432.1, AP001767.4, AP001931.1, AP001931.2, AP002813.1, AP003392.1, AP1G1, AP2S1, AP3M1, AP4E1, APAF1, APH1B, API5, APOOL, APPBP2, APPL1, AQR, ARCN1, AREL1, ARFGEF1, ARHGAP12, ARHGAP31, ARHGAP31-AS1, ARHGAP42, ARHGAP5, ARHGEF11, ARHGEF12, ARHGEF18, ARHGEF26, ARHGEF3, ARHGEF6, ARID1A, ARID1B, ARID2, ARID4A, ARID4B, ARIH1, ARL10, ARL2, ARL2BP, ARL5A, ARL5B, ARL6IP4, ARMCX3, ARMH4, ARPIN, ARSB, ARSK, ASAP1, ASAP2, ASB16, ASB7, ASCC3, ASH1L, ASXL1, ASXL2, ATAD2B, ATF2, ATF6, ATF6B, ATF7, ATF7IP, ATG10, ATL2, ATM, ATMIN, ATOX1, ATP10D, ATP11A, ATP11B, ATP2A2, ATP2B4, ATP2C1, ATP5F1D, ATP5F1E, ATP5MC1, ATP5MC2, ATP5MC3, ATP5MD, ATP5ME, ATP5MF, ATP5MF-PTCD1, ATP5MG, ATP5PD, ATP6V1F, ATP6V1G2-DDX39B, ATP7A, ATP8B2, ATR, ATRN, ATRX, ATXN1, ATXN1-AS1, ATXN1L, ATXN2L, ATXN7, ATXN7L1, AURKAIP1, AUTS2, AVL9, AVPI1, AZI2, B3GALNT2, B3GALT5, B3GAT3, BABAM1, BAD, BAG1, BAG5, BAG6, BAHD1, BAZ1B, BAZ2A, BAZ2B, BBS10, BBS9, BBX, BCAP29, BCCIP, BCHE, BCL9, BCL9L, BCLAF1, BCORL1, BDP1, BGLAP, BICD1, BICD2, BICRAL, BIRC2, BIRC6, BLM, BLOC1S1, BLOC1S6, BLZF1, BMPR1A, BMPR2, BMS1, BMS1P1, BMS1P4, BMT2, BOD1L1, BORCS6, BORCS8, BPNT2, BPTF, BRAF, BRAP, BRCC3, BRD2, BRD3, BROX, BRPF3, BRWD3, BSDC1, BTAF1, BTBD1, BTBD7, BX322562.1, BX322635.1, BX571846.1, BX679664.3, BZW1P2, C11orf54, C12orf4, C12orf49, C12orf66, C16orf72, C16orf86, C16orf91, C17orf80, C18orf25, C19orf53, C1D, C1orf109, C1orf122, C1orf50, C1orf74, C2CD3, C2orf16, C2orf49, C4orf48, C5, C5orf24, C5orf51, C5orf66, C6orf120, C6orf89, C9orf147, C9orf64, CABIN1, CADM1, CALCRL, CAND1, CANX, CAPN7, CAPNS1, CAPRIN1, CARNMT1, CASP8AP2, CASTOR1, CBL, CBLL1, CBWD2, CBX3P2, CBX4, CCAR1, CCDC106, CCDC12, CCDC126, CCDC144CP, CCDC159, CCDC171, CCDC174, CCDC183-AS1, CCDC28A, CCDC47, CCDC50, CCDC82, CCDC85B, CCDC88A, CCDC93, CCM2, CCNJ, CCNQ, CCNT1, CCS, CDC14A, CDC14B, CDC27, CDC40, CDC42BPA, CDC5L, CDC73, CDH1, CDH2, CDK12, CDK13, CDK2AP2, CDK5RAP2, CDKN2AIP, CDON, CDPF1, CECR2, CELF1, CELSR2, CENPC, CEP104, CEP120, CEP135, CEP152, CEP162, CEP164P1, CEP170, CEP170P1, CEP192, CEP250, CEP295, CEP350, CEP57, CEP70, CEP85L, CEPT1, CERS6, CES2, CFAP97, CGGBP1, CGN, CH17-340M24.3, CHD2, CHD4, CHD6, CHD8, CHD9, CHL1-AS1, CHML, CHMP4A, CHROMR, CHST3, CHST9, CHTF8, CHURC1-FNTB, CIC, CICP14, CICP16, CICP3, CISD3, CITED4, CKAP5, CLASP1, CLCC1, CLCN3, CLCN5, CLEC16A, CLIC3, CLINT1, CLIP1, CLOCK, CLPX, CLTC, CMTM1, CMTR1, CMTR2, CNEP1R1, CNFN, CNKSR3, CNOT1, CNOT4, CNOT6, CNOT6L, CNOT7, CNTRL, COA3, COA7, COG2, COG5, COG6, COG8, COIL, COMMD1, COMTD1, COPA, COPE, COPG2, COPRS, COPS9, COQ7, COX10-AS1, COX11, COX14, COX17, COX18, COX4I1, COX5B, COX6B1, COX6C, COX7B, COX7C, COX8A, CPLANE1, CPNE2, CPNE3, CPSF2, CPSF7, CR381653.2, CRACD, CRAMP1, CRAT, CREB1, CREB5, CREBBP, CRISPLD1, CRK, CRTC3, CRY1, CRYZP1, CSDE1, CSNK1G1, CSNK1G3, CSNK2A1, CSNK2A2, CSNK2A3, CSPG4P10, CTAGE7P, CTBP1-DT, CTCF, CTDSPL2, CTNNB1, CTPS2, CTR9, CTTNBP2NL, CUL3, CUL4B, CUL5, CUX1, CWC22, CWC27, CWF19L2, CYB5RL, CYP20A1, CYP2U1, CYP51A1, CYTH3, DAAM1, DAG1, DARS1, DARS2, DCAF1, DCAF10, DCAF17, DCAF5, DCAF7, DCHS1, DCK, DCLRE1A, DCLRE1C, DCP1A, DCTN4, DCTN5, DCUN1D4, DCXR, DDB1, DDI2, DDR1, DDT, DDX19A, DDX19B, DDX20, DDX3X, DDX42, DDX46, DDX5, DDX6, DENND1B, DENND4A, DENND4C, DENND5A, DET1, DFFA, DGCR6L, DGKH, DHFR2, DHRS4L2, DHTKD1, DHX15, DHX29, DHX33, DHX35, DHX36, DHX38, DHX40, DHX57, DHX8, DHX9, DIDO1, DIMT1, DIS3, DISC1, DLAT, DLD, DLEU2L, DLG5, DLGAP4, DMWD, DMXL1, DMXL2, DNAJA2, DNAJA3, DNAJC10, DNAJC11, DNAJC13, DNAJC14, DNAJC16, DNAJC24, DNAJC4, DND1P1, DNMBP, DNMT3A, DOP1B, DPP8, DPY19L1, DPY19L3, DPY19L4, DPY30, DR1, DST, DSTNP1, DUS4L, DUSP16, DUSP18, DYM, DYNC1I2, DYNC1LI1, DYNC1LI2, DYNLL1, DYNLRB1, E2F6P1, EARS2, EBLN2, EBLN3P, ECD, ECH1, ECHS1, EDC4, EDEM3, EDF1, EDRF1, EEA1, EEF1B2, EEF1B2P3, EFCAB14, EFHD2, EFL1P1, EIF1AX, EIF2AK2, EIF2AK3, EIF2AK4, EIF3A, EIF3K, EIF4EBP2, EIF4EP2, EIF4G1, EIF4G2, EIF4G3, ELF2, ELK4, ELOB, ELOCP19, ELOVL5, ELOVL6, ELP1, ELP3, EMC1, EMSY, ENAH, EOLA1, EP300, EP400, EPB41, EPB41L4A, EPB41L5, EPC1, EPC2, EPG5, EPM2AIP1, EPRS1, EPS8, ERC1, ERCC4, ERCC6, ERCC6L2, ERCC8, ERGIC2, ERI2, ERI3-IT1, ERLEC1, ERLIN2, ERV3-1, ERVW-1, ESF1, ETAA1, ETFB, ETV1, ETV3, EVI5, EXOC1, EXOC2, EXOC4, EXOC5, EXOC7, EXOC8, EXOSC5, EXTL2, EYA3, FAF2, FAM104A, FAM120A, FAM120AOS, FAM120C, FAM122A, FAM135A, FAM172A, FAM193A, FAM199X, FAM207A, FAM20B, FAM214A, FAM222B, FAM3C, FAM3C2, FAM76B, FAM98B, FAM98C, FAN1, FARP2, FASTKD2, FAT1, FBH1, FBXL14, FBXL4, FBXL5, FBXO11, FBXO22, FBXO28, FBXO30, FBXO38, FBXO48, FBXW2, FBXW8, FDX2, FEM1A, FEM1B, FER, FERMT2, FGD5-AS1, FGF2, FHL1, FIBIN, FIGN, FIS1, FITM2, FKBP14, FKBP2, FKBP8, FKTN, FLRT3, FLVCR1, FNBP1L, FNBP1P1, FNDC3A, FNIP1, FOXJ2, FOXJ3, FOXK1, FOXN3, FP671120.4, FPGT, FRG1HP, FRMPD3, FRYL, FSBP, FUBP1, FUBP3, FUT10, FXR1, FYTTD1, FZD3, GAB1, GABARAP, GABPA, GADD45GIP1, GALNT1, GAMT, GAPVD1, GARRE1, GAS5-AS1, GATAD2B, GATC, GCHFR, GEMIN5, GFM1, GFPT1, GGNBP2, GIGYF2, GIT2, GK5, GLCE, GLG1, GLIDR, GLIS2, GLMN, GLUD1P3, GLYR1, GMCL1, GMDS, GNA11, GNA12, GNE, GNPNAT1, GNPTG, GOLGA1, GOLGA2, GOLGA2P10, GOLGA4, GOLIM4, GOLM1, GOLM2, GOLT1A, GON4L, GOSR1, GP1BA, GPALPP1, GPATCH1, GPATCH11, GPATCH2L, GPATCH8, GPD2, GPR107, GPR75, GPR89A, GPR89B, GPX4, GRIK3, GRK2, GRPEL2, GRPR, GSE1, GSK3B, GSPT1, GSPT2, GSTCD, GSTO1, GSTZ1, GTF2A1, GTF2H2, GTF2H2C, GTF2H3, GTF2I, GTF2IRD1P1, GTF3C1, GTF3C3, GTF3C4, GTPBP10, GUK1, GUSBP3, GUSBP9, GXYLT1, H2AC15, H2BC20P, H2BC7, H2BP1, H3C13, H3P4, H4-16, HACD2, HACD3, HBA1, HBA2, HBB, HCFC1, HCFC1R1, HCFC2, HCG18, HDAC9, HDX, HEATR1, HEATR5A, HEATR5B, HEATR6, HECTD1, HELQ, HELZ, HERC2, HHLA3, HIC2, HIGD2A, HINT1, HIP1, HIRA, HIVEP1, HLCS, HLTF, HMBOX1, HMG20A, HMGN3, HMGN3-AS1, HNRNPCP7, HNRNPH2, HNRNPLL, HOOK3, HP1BP3, HRAS, HSBP1L1, HSD17B12, HSPA13, HSPA14, HSPA1L, HSPA4, HSPD1P11, HTD2, HUWE1, IARS1, IARS2, IBA57, IBTK, ICE1, ICE2, IDE, IFFO2, IFI27L2, IFNAR1, IFT81, IGF1R, IKBKG, IKZF2, IKZF4, IL17RD, IL1RL2, IL6ST, ILDR2, IMP3, IMPACT, INE2, ING3, INIP, INO80, INO80D, INTS12, INTS2, INTS6, INVS, IPMK, IPO11, IPO7, IPO8, IPO9, IPP, IPPK, IRAK1BP1, IREB2, IRF2BPL, IRGQ, ISOC2, ITCH, ITGAE, ITGAV, ITGB8, ITPR2, ITPRID2, JAK1, JAM3, JARID2, JMJD1C, JMJD7, JOSD2, JRK, JRKL, JTB, JUND, KANSL1, KANSL1L, KAT6A, KAT7, KATNAL1, KBTBD4, KBTBD8, KCMF1, KCNK7, KCTD15, KCTD3, KCTD7, KDM1B, KDM2A, KDM3A, KDM3B, KDM4A, KDM5A, KDM5B, KDM5C, KDM6A, KDM7A, KHSRP, KIAA0100, KIAA0319L, KIAA0586, KIAA1143, KIAA1586, KIAA1958, KIAA2026, KLF3, KLF7, KLF7-IT1, KLHDC10, KLHL11, KLHL12, KLHL13, KLHL15, KLHL18, KLHL20, KLHL24, KLHL28, KLHL36, KLHL4, KLHL7, KLHL8, KLRF1, KLRG1, KMT2A, KMT2C, KMT2D, KMT2E, KPNA1, KPNA4, KPNA6, KRAS, KRIT1, KRR1, KRT17, KRT8P46, KTN1, L3MBTL3, LAGE3, LAMTOR3, LAMTOR4, LARP1, LARP1B, LARP4, LARS1, LARS2, LATS1, LCA5, LCLAT1, LCMT2, LCOR, LDHAP2, LDLRAD3, LEMD3, LENG1, LEO1, LEPROTL1, LETM1, LETM2, LIG3, LIN54, LINC00205, LINC00216, LINC00235, LINC00461, LINC00471, LINC00652, LINC00654, LINC00662, LINC00665, LINC00667, LINC00928, LINC01347, LINC01550, LINC01909, LINC02282, LINS1, LMBR1, LMLN, LNPK, LNX2, LONP2, LRATD2, LRBA, LRCH3, LRFN3, LRIG1, LRIG2, LRP12, LRP5, LRP6, LRPAP1, LRPPRC, LRRC37A16P, LRRC37A4P, LRRC40, LRRC57, LRRC58, LRRC8A, LRRCC1, LRRFIP1P1, LRRN3, LSM14A, LTN1, LY6E, LYRM2, LYST, MACF1, MAGI2-AS3, MAML2, MAN1A2, MANEA, MANEA-DT, MAP10, MAP2K2, MAP3K1, MAP3K2, MAP3K20, MAP3K7, MAP4K3, MAP9, MAPK14, MAPK6, MARCHF2, MARCHF5, MARCHF7, MARK2, MARS2, MASP1, MAST2, MAT2A, MAVS, MBD1, MBD5, MBTPS1, MBTPS2, MCCC1, MCCC2, MCM3AP, MCM3AP-AS1, MCM9, MCRIP2, MCTS2P, MDC1, MEAK7, MECP2, MED1, MED12, MED13, MED13L, MED14, MED21, MED23, MED28, MEF2C-AS2, MEGF8, MEIS3P1, METTL14, METTL16, METTL26, METTL2A, METTL4, METTL6, METTL8, MFAP3, MFHAS1, MFN1, MFSD14C, MFSD3, MGA, MGAT5, MGMT, MGRN1, MIA3, MIB1, MICOS10, MICOS13, MID1, MIEF1, MIEN1, MIER1, MIER3, MIF, MINDY2, MIOS, MIR100HG, MKLN1, MKLN1-AS, MLH3, MLLT10, MMAA, MMGT1, MMUT, MOB1B, MOB2, MOB4, MON1B, MON2, MORC3, MORF4L1P1, MPC2, MPDZ, MPG, MPHOSPH9, MROH6, MRPL19, MRPL20, MRPL21, MRPL23, MRPL27, MRPL30, MRPL34, MRPL41, MRPL43, MRPL50, MRPL53, MRPL54, MRPS30, MRPS31P4, MSH3, MSI2, MSL2, MSTN, MTCL1, MTCO1P28, MTDH, MTF1, MTIF2, MTIF3, MTLN, MTM1, MTMR1, MTMR2, MTMR3, MTND1P8, MTO1, MTOR, MTPN, MTR, MTREX, MTRF1L, MTRNR2L4, MTX3, MYG1, MYNN, MYO5B, MYO9A, MZT2A, MZT2B, N4BP1, N4BP2, NAA15, NAA16, NAA25, NAA35, NAA38, NAALADL2, NAB1, NACC1, NAE1, NAF1, NAIPP2, NAP1L4P1, NARS1, NARS2, NAT10, NATD1, NAV1, NAV2, NBAS, NBDY, NBEAL1, NBN, NBPF1, NBPF11, NBPF12, NBPF25P, NBPF26, NBPF3, NBR1, NCBP1, NCBP2, NCK2, NCKAP1, NCKAP5, NCOA3, NCOA6, NCOR1, NCOR2, NDST1, NDUFA1, NDUFA11, NDUFA13, NDUFA2, NDUFA3, NDUFA7, NDUFA8, NDUFAF3, NDUFAF8, NDUFB1, NDUFB10, NDUFB11, NDUFB2, NDUFB3, NDUFB4, NDUFB7, NDUFS5, NDUFS7, NDUFS8, NDUFV1, NEB, NEDD4, NEDD8, NEK1, NEK4, NEMP2, NENF, NFAT5, NFATC2IP, NFATC3, NFRKB, NFX1, NFXL1, NFYA, NHLRC2, NHLRC3, NHSL1, NIN, NIPA2, NIPBL, NKAPD1, NLGN1, NLGN4X, NLN, NME3, NME6, NOA1, NOL6, NOM1, NOMO1, NOMO3, NOSIP, NOTCH1, NOTCH2, NPAT, NPEPPS, NPR2, NR2C2, NR3C1, NRCAM, NRDE2, NRIP1, NSD1, NSD3, NSUN3, NTN3, NUDT14, NUDT21, NUDT4, NUDT4B, NUDT8, NUFIP2, NUMA1, NUP133, NUP153, NUP58, NUP98, NUS1P1, NXT2, OAZ1, OBSL1, OCRL, ODR4, OGFOD1, OGG1, ONECUT1, OR7E128P, OR7E38P, OR7E7P, ORC2, ORC3, OSBPL11, OSBPL8, OSGEPL1, OTUD4, OTUD6B, OXNAD1, PACS1, PAG1, PAICS, PAIP1, PALB2, PAM16, PANK3, PAPOLA, PAPOLG, PAQR3, PARD3, PARG, PARGP1, PARN, PARS2, PARVB, PBRM1, PCDH1, PCDH17, PCDHB11, PCDHB12, PCDHB13, PCDHGA1, PCDHGA4, PCDHGB2, PCDHGB9P, PCF11, PCM1, PCMTD2, PCNX1, PCNX4, PCSK7, PCYOX1, PDCL, PDE12, PDF, PDIA3P1, PDLIM5, PDPK2P, PDPR, PDS5A, PDXDC1, PEAK1, PEF1, PEX1, PEX12, PEX16, PEX19, PEX3, PFN1P4, PFN1P6, PFN1P8, PGAP1, PGBD4, PGGT1B, PGM5P2, PHC1, PHC1P1, PHC3, PHF12, PHF2, PHF20, PHF20L1, PHF21A, PHF3, PHF6, PHF8, PHIP, PHKB, PHPT1, PI4KB, PIAS1, PIAS2, PIAS3, PIBF1, PIGM, PIGN, PIGO, PIGS, PIH1D1, PIK3C2A, PIK3CA, PIK3R4, PIKFYVE, PIP5K1A, PJA2, PKD1, PKD1P5, PKD1P6, PKN2, PKNOX1, PLAC9, PLAGL2, PLCE1, PLCG1, PLD3, PLPP6, PLRG1, PLXNA2, PLXNA4, PMM1, PMPCB, PMS1, PMS2, PNKD, PNPLA8, PNPT1, PNRC2, POGK, POLDIP3, POLK, POLL, POLR1A, POLR1B, POLR2A, POLR2B, POLR2C, POLR2E, POLR2I, POLR3E, POM121, POM121C, POMC, POMK, POMP, POU2F1, POU3F2, PPARA, PPAT, PPDPF, PPFIA1, PPFIBP1, PPIG, PPIP5K2, PPM1D, PPP1R12A, PPP1R12A-AS1, PPP1R12C, PPP2R3A, PPP2R5D, PPP2R5E, PPP4R3A, PPP4R3B, PPP6C, PPP6R3, PPT2, PPTC7, PRAL, PRDM10, PRDM4, PRDX1, PRDX5, PRKAA1, PRKAR2A, PRKCI, PRKD1, PRKD3, PRKDC, PRKXP1, PRMT3, PRMT9, PRORP, PRPF38A, PRPF40A, PRPF4B, PRPF8, PRPS1P2, PRPSAP1, PRR12, PRR14L, PRR5, PRRC2A, PRRC2B, PRRC2C, PRTG, PRUNE1, PSKH1, PSMD1, PSMD12, PSMD2, PSME4, PTAR1, PTCD2, PTCHD1, PTN, PTPN13, PTPN14, PTPN21, PTPN23, PTPRE, PTPRF, PTPRG, PTPRZ1, PTRH1, PUM1, PUM2, PURB, PUS7L, PWWP2A, PXYLP1, PYGO1, QRSL1, QSER1, R3HCC1L, RAB10, RAB14, RAB22A, RAB23, RAB28P5, RAB2B, RAB39A, RAB3GAP1, RAB3GAP2, RAB40A, RAB4B-EGLN2, RAB5A, RAB8B, RABAC1, RABGAP1, RABGGTA, RAD17, RAD50, RAD51B, RAD54L2, RAI1, RALGAPA2, RALGAPB, RANBP10, RANBP2, RANBP6, RANBP9, RAP2C-AS1, RAPGEF1, RAPGEF6, RAPH1, RASA1, RASAL2, RASSF10-DT, RASSF7, RAVER1, RB1, RBAK, RBBP5, RBBP6, RBL2, RBM12B, RBM12B-AS1, RBM14-RBM4, RBM15, RBM18, RBM26-AS1, RBM27, RBM41, RBM43, RBMXL1, RBX1, RC3H1, RC3H2, RDX, RECQL, REV1, REV3L, REX1BD, RFC1, RFK, RFX3, RFX3-AS1, RFX5, RFX7, RGMB, RGP1, RGS17, RHEBL1, RIC1, RIC8B, RIF1, RIMBP3, RIMKLBP2, RINL, RIOK2, RLF, RLIM, RNASEK, RNASEK-C17orf49, RNF111, RNF121, RNF126, RNF139, RNF169, RNF180, RNF185, RNF185-AS1, RNF20, RNF216, RNF24, RNF26, RNF34, RNF38, RNF40, RNGTT, RNMT, RO60, ROCK1, ROCK2, RPAP1, RPAP2, RPAP3, RPE, RPGRIP1L, RPL10AP6, RPL10P16, RPL10P9, RPL12P16, RPL13AP5, RPL14P1, RPL15P3, RPL18AP3, RPL18AP7, RPL21, RPL21P16, RPL22, RPL22P1, RPL28P2, RPL31, RPL34, RPL3P4, RPL4P4, RPL6P27, RPL7AP10, RPL7L1, RPL7P57, RPP14, RPP25L, RPRD2, RPS12, RPS15AP36, RPS19BP1, RPS23P8, RPS24, RPS27A, RPS27AP5, RPS3AP26, RPS3AP6, RPS6KB1, RPS7P1, RPTOR, RRAGD, RRN3, RSBN1, RSBN1L, RSF1, RSPRY1, RTL6, RUBCN, RXRB, SACS, SALL1, SAMD4B, SAP130, SAP30L, SARM1, SART3, SBDSP1, SBF2, SCAF11, SCAF4, SCAF8, SCAP, SCARB2, SCFD1, SCMH1, SCO1, SCRN3, SCYGR4, SCYL2, SCYL3, SDAD1, SDCBP2-AS1, SDE2, SDHAF1, SDK2, SEC16A, SEC22B, SEC22C, SEC23A, SEC24B, SEC24C, SEC63, SECISBP2, SELENOH, SEMA5A, SEMA6A, SENP3-EIF4A1, SENP6, SENP7, SEPSECS-AS1, SEPTIN11, SEPTIN2, SERAC1, SERTAD2, SETBP1, SETD1A, SETD1B, SETD2, SETD5, SETX, SF1, SF3A1, SFT2D2, SGCB, SGPL1, SH3BP5L, SH3RF1, SH3RF3-AS1, SHC4, SHPRH, SHQ1P1, SIGIRR, SIK2, SIKE1, SIN3A, SIPA1L2, SIPA1L3, SKI, SLC10A7, SLC11A2, SLC12A6, SLC16A2, SLC19A2, SLC23A2, SLC25A25, SLC25A28, SLC25A36, SLC25A46, SLC26A1, SLC30A1, SLC30A6, SLC31A1, SLC35A1, SLC35A3, SLC35A5, SLC35B4, SLC35E1P1, SLC36A1, SLC36A4, SLC38A9, SLC39A4, SLC39A9, SLC49A4, SLC6A9, SLC7A1, SLC7A6OS, SLC8A1-AS1, SLC9A3, SLC9A8, SLF1, SLF2, SLIRP, SLMAP, SLX4, SLX4IP, SMAD1, SMAD5, SMARCA1, SMARCA5, SMARCA5-AS1, SMARCAD1, SMC5, SMC5-AS1, SMC6, SMCHD1, SMDT1, SMG1, SMG1P1, SMG1P3, SMG1P6, SMG5, SMG6, SMG7, SMG7-AS1, SMG8, SMIM1, SMIM19, SMIM29, SMPD2, SMPD5, SMPDL3B, SMU1, SMURF1, SMURF2, SMYD4, SNAPC3, SNHG31, SNHG8, SNRNP200, SNRNP27, SNRNP48, SNX12, SNX13, SNX14, SNX18P9, SNX19, SNX25, SOCS4, SOCS5, SOGA1, SON, SOS1, SOS2, SOX2, SOX9, SP1, SP2, SP2-AS1, SP3, SP4, SPAG7, SPAG9, SPATA46, SPATA5, SPATA6, SPECC1L, SPEN, SPG11, SPRED1, SPRED2, SPTLC1, SRC, SRCAP, SREK1IP1, SRFBP1, SRGAP1, SRGAP2, SRGAP2B, SRGAP2C, SRGAP2D, SRP14, SRP68, SRP72, SRRM2, ST8SIA1, STAG1, STAG2, STAG3, STAG3L2, STAG3L3, STAM2, STARD4-AS1, STARD7, STARD7-AS1, STARD9, STAT5B, STIM2, STIMATE, STK32A, STK35, STK4, STRA6LP, STRN3, STUB1, STX12, STX17, STXBP4, SUCO, SUGT1P4-STRA6LP, SUPT6H, SURF1, SYNE2, SYNRG, SYT11, SZT2, TAB2, TAB3, TADA2B, TAF1, TAF10, TAF1A, TAF1B, TAF2, TAF7, TAF9B, TALDO1, TANC1, TANC2, TANGO6, TAOK1, TAS2R3, TAS2R31, TASOR, TASOR2, TATDN2P2, TAX1BP1, TBC1D19, TBC1D20, TBC1D22B, TBC1D23, TBC1D25, TBC1D32, TBC1D5, TBCB, TBCCD1, TBCEL, TBCK, TBL1X, TBL1XR1, TCAF1P1, TCAIM, TCEA1P2, TCEA2, TCEANC, TCEANC2, TCF12, TCF20, TCF4, TEAD1, TECPR1, TECR, TECRP1, TENM1, TENT4B, TET1, TET2, TET3, TEX10, TEX261, TFAM, TFCP2, TFDP2, TFF3, TFPT, TGFB2, TGFBRAP1, TGS1, THAP1, THAP12, THAP5, THAP6, THAP9, THBS2, THOC2, THOC7, THRAP3, THUMPD1, THUMPD3, TIAL1, TIGAR, TIGD1, TIGD4, TIGD7, TIMM10, TIMM23B-AGAP6, TIMM8A, TIRAP, TLCD4-RWDD3, TLE1P1, TLK2, TM7SF3, TM9SF2, TM9SF3, TMA7, TMCC1, TMCC1-AS1, TMCO1-AS1, TMED7, TMEM106B, TMEM131, TMEM141, TMEM160, TMEM161B, TMEM167A, TMEM168, TMEM170A, TMEM181, TMEM184C, TMEM185A, TMEM185B, TMEM191B, TMEM191C, TMEM192, TMEM200C, TMEM202-AS1, TMEM219, TMEM255A, TMEM267, TMEM30A, TMEM30A-DT, TMEM33, TMEM63B, TMEM68, TMEM91, TMF1, TMLHE, TMTC3, TMX2P1, TMX3, TNFRSF18, TNFSF12, TNFSF9, TNKS, TNKS2, TNN, TNNC1, TNPO1, TNPO2, TNPO3, TNRC18, TNRC6A, TNRC6B, TOB2P1, TOP1, TOPORS, TOR1AIP2, TP53BP1, TP53INP1, TPGS1, TPP2, TPR, TPT1P9, TPTEP2, TRAF6, TRAPPC1, TRAPPC11, TRAPPC2L, TRAPPC5, TRAPPC6A, TRAPPC8, TRDMT1, TREH, TREX1, TRGC2, TRIM26, TRIM32, TRIM33, TRIM35, TRIM39, TRIM68, TRIO, TRIP11, TRIP12, TRIR, TRMT10A, TRMT10B, TRMT12, TRMT44, TRRAP, TSC22D2, TSNAX, TSPAN12, TSR1, TSSK3, TSSK4, TSSK6, TSTD2, TTC1, TTC13, TTC17, TTC21B, TTC28, TTC37, TTC3P1, TTLL12, TTLL5, TTPAL, TUBD1, TUBGCP5, TUT4, TXLNG, TXNDC16, TXNRD2, TYW5, UBA2, UBA5, UBAP2L, UBE2D3-AS1, UBE2G2, UBE2L3, UBE2Q2P2, UBE3A, UBE3B, UBE3C, UBE4A, UBFD1, UBIAD1, UBL5, UBLCP1, UBN1, UBP1, UBQLN1, UBR1, UBR2, UBR4, UBR5, UBR5-AS1, UBR7, UBXN1, UBXN2B, UBXN4, UBXN6, UBXN7, UEVLD, UFL1, UFSP2, UGDH-AS1, UGGT2, UHRF1BP1, UNC119B, UNC13B, UNC50, UPK3A, UPRT, UQCR10, UQCR11, UQCRH, UQCRQ, URB1, URB2, USE1, USF2, USF3, USP10, USP14, USP15, USP19, USP24, USP27X-AS1, USP28, USP32, USP33, USP34, USP37, USP38, USP4, USP42, USP45, USP48, USP6NL, USP7, USP8, USP9X, USPL1, UST, UTP20, UTP23, UTP25, UTP4, UTRN, UVSSA, VANGL2, VCPIP1, VEZF1, VIRMA, VKORC1L1, VMA21, VN1R108P, VN1R83P, VPS13A, VPS13B, VPS13C, VPS26C, VPS33A, VPS35, VPS35L, VPS41, VPS45, VPS54, VPS8, VSIG10, WAPL, WASH5P, WASHC4, WASHC5, WASL, WBP11, WDFY1, WDFY2, WDFY3, WDPCP, WDR12, WDR20, WDR26, WDR3, WDR35, WDR36, WDR41, WDR43, WDR44, WDR75, WDR82, WIZ, WLS, WNK3, WNT11, XIAP, XKR6, XPC, XPO1, XPO4, XPO6, XPO7, XPOT, XPR1, XRN1, XYLB, YBEY, YBX2P2, YDJC, YEATS2, YIF1B, YIPF4, YIPF6, YLPM1, YOD1, YTHDC2, YTHDF3, YY1, YY2, Z68871.1, Z92544.1, Z94721.2, Z97055.2, Z98884.2, ZBED6, ZBED8, ZBTB1, ZBTB10, ZBTB11, ZBTB14, ZBTB20, ZBTB21, ZBTB22, ZBTB24, ZBTB26, ZBTB33, ZBTB34, ZBTB37, ZBTB38, ZBTB39, ZBTB40, ZBTB41, ZBTB43, ZBTB49, ZBTB5, ZBTB6, ZC3H10, ZC3H12C, ZC3H14, ZC3H18, ZC3H4, ZC3H6, ZC3H7A, ZCCHC10, ZCCHC14, ZCCHC3, ZDHHC15, ZDHHC3, ZEB1, ZFAND3, ZFAND4, ZFAT, ZFC3H1, ZFHX3, ZFHX4, ZFHX4-AS1, ZFP14, ZFP28, ZFP3, ZFP30, ZFP37, ZFP62, ZFP64, ZFP82, ZFP90, ZFP91, ZFX, ZFYVE19, ZFYVE26, ZHX1, ZHX2, ZKSCAN1, ZKSCAN2, ZKSCAN3, ZKSCAN4, ZKSCAN8, ZMAT5, ZMIZ1, ZMPSTE24, ZMYM3, ZMYM4, ZNF100, ZNF107, ZNF112, ZNF117, ZNF12, ZNF131, ZNF132, ZNF136, ZNF137P, ZNF14, ZNF142, ZNF146, ZNF148, ZNF154, ZNF155, ZNF160, ZNF17, ZNF174, ZNF175, ZNF181, ZNF182, ZNF189, ZNF19, ZNF192P1, ZNF197, ZNF200, ZNF207, ZNF211, ZNF219, ZNF221, ZNF222, ZNF223, ZNF225, ZNF227, ZNF229, ZNF234, ZNF235, ZNF239, ZNF24, ZNF252P, ZNF253, ZNF254, ZNF256, ZNF260, ZNF263, ZNF264, ZNF268, ZNF271P, ZNF275, ZNF280C, ZNF281, ZNF283, ZNF285, ZNF285B, ZNF286A, ZNF292, ZNF30, ZNF302, ZNF304, ZNF30-AS1, ZNF317, ZNF318, ZNF319, ZNF320, ZNF322, ZNF343, ZNF345, ZNF347, ZNF354B, ZNF354C, ZNF358, ZNF362, ZNF37A, ZNF383, ZNF384, ZNF391, ZNF407, ZNF41, ZNF417, ZNF420, ZNF425, ZNF426, ZNF429, ZNF43, ZNF430, ZNF431, ZNF433, ZNF440, ZNF441, ZNF442, ZNF445, ZNF449, ZNF45, ZNF451, ZNF454, ZNF460, ZNF461, ZNF462, ZNF468, ZNF470, ZNF480, ZNF484, ZNF485, ZNF490, ZNF500, ZNF501, ZNF502, ZNF506, ZNF507, ZNF510, ZNF512, ZNF516, ZNF518B, ZNF521, ZNF524, ZNF527, ZNF528, ZNF529, ZNF532, ZNF542P, ZNF543, ZNF548, ZNF549, ZNF550, ZNF555, ZNF557, ZNF558, ZNF559-ZNF177, ZNF562, ZNF563, ZNF564, ZNF565, ZNF566, ZNF567, ZNF568, ZNF569, ZNF57, ZNF570, ZNF571, ZNF572, ZNF573, ZNF580, ZNF583, ZNF585A, ZNF585B, ZNF587, ZNF592, ZNF594, ZNF595, ZNF597, ZNF605, ZNF606, ZNF607, ZNF608, ZNF609, ZNF611, ZNF613, ZNF614, ZNF615, ZNF616, ZNF618, ZNF619, ZNF620, ZNF621, ZNF623, ZNF624, ZNF626, ZNF627, ZNF629, ZNF630, ZNF644, ZNF646, ZNF649, ZNF654, ZNF655, ZNF658, ZNF66, ZNF660, ZNF665, ZNF671, ZNF674, ZNF675, ZNF677, ZNF678, ZNF680, ZNF684, ZNF687, ZNF689, ZNF696, ZNF697, ZNF699, ZNF70, ZNF708, ZNF710, ZNF713, ZNF714, ZNF717, ZNF718, ZNF720, ZNF721, ZNF730, ZNF740, ZNF746, ZNF747, ZNF75D, ZNF761, ZNF765, ZNF766, ZNF770, ZNF772, ZNF773, ZNF776, ZNF778, ZNF780A, ZNF782, ZNF785, ZNF786, ZNF79, ZNF790, ZNF791, ZNF792, ZNF793, ZNF800, ZNF805, ZNF81, ZNF827, ZNF829, ZNF836, ZNF844, ZNF850, ZNF852, ZNF879, ZNF883, ZNF8-ERVK3-1, ZNF91, ZNHIT6, ZNRF2P1, ZNRF2P2, ZRANB3, ZSCAN12, ZSCAN12P1, ZSCAN2, ZSCAN20, ZSCAN22, ZSCAN23, ZSCAN25, ZSCAN29, ZSCAN30, ZSCAN32, ZSWIM1, ZSWIM3, ZSWIM5, ZSWIM6, ZSWIM8, ZUP1, ZXDA, ZXDB, ZXDC, ZZEF1, ZZZ3 | |
| Green module  (ME5) | | ABCB10, ABCF1, ABCF2, ABHD8, ABL1, ABLIM3, ABT1, AC002116.2, AC002310.1, AC002550.2, AC004540.1, AC004540.2, AC004943.1, AC004943.2, AC005336.1, AC005821.1, AC006960.3, AC007383.1, AC007620.2, AC008543.1, AC008894.3, AC009041.3, AC009118.2, AC009303.4, AC009309.2, AC009486.2, AC009533.1, AC009902.2, AC009902.3, AC009955.4, AC010173.1, AC010205.1, AC010504.1, AC011447.7, AC011468.4, AC011503.2, AC011700.1, AC012073.1, AC012184.3, AC012306.2, AC012360.3, AC012447.1, AC012640.2, AC013477.2, AC015909.2, AC016027.1, AC016065.1, AC016355.1, AC017104.1, AC018695.4, AC020765.2, AC020951.1, AC021037.1, AC021242.3, AC022150.2, AC022150.4, AC022211.1, AC022211.2, AC022413.1, AC025171.1, AC025283.2, AC026401.3, AC026471.1, AC026471.6, AC027601.5, AC027612.1, AC046143.1, AC046185.3, AC053503.4, AC064875.1, AC067931.1, AC068473.5, AC068831.6, AC069499.1, AC073046.1, AC073343.2, AC073389.1, AC073413.1, AC073508.3, AC073896.3, AC074135.1, AC079015.1, AC083805.3, AC087501.4, AC087632.2, AC090114.2, AC090692.1, AC091057.1, AC091057.6, AC091057.7, AC091060.1, AC092614.1, AC092687.3, AC092718.4, AC093525.2, AC093627.5, AC093675.1, AC093677.2, AC096677.1, AC097358.2, AC098588.2, AC099568.2, AC100810.1, AC104561.4, AC105020.4, AC107214.1, AC107871.1, AC109460.2, AC110285.6, AC110597.1, AC112777.1, AC114956.2, AC115618.1, AC120114.1, AC124248.1, AC125437.1, AC125611.4, AC126407.1, AC126474.1, AC127496.7, AC131097.3, AC131212.3, AC132938.4, AC135048.3, AC135050.1, AC136624.2, AC138409.3, AC138811.2, AC138904.1, AC145124.1, AC234917.3, AC243964.3, AC244517.1, AC244517.5, AC244517.6, AC244517.7, ACBD6, ACLY, ACOT11, ACSBG1, ACSF2, ACTR5, ACVR2B-AS1, ACY3, ACYP2, AD000671.1, ADAMTS6, ADAMTS7, ADH5, ADSL, AFAP1, AGBL5, AKAP8, AKNAD1, AL009178.2, AL009179.1, AL023803.2, AL024507.2, AL033527.2, AL033529.1, AL034550.3, AL078644.1, AL079343.1, AL080276.2, AL109936.8, AL117327.1, AL121603.2, AL121894.2, AL133215.2, AL133390.1, AL133500.1, AL136295.6, AL137060.3, AL137802.3, AL138781.1, AL138789.1, AL139234.1, AL139393.3, AL161729.1, AL161891.1, AL162258.2, AL163051.1, AL353135.2, AL358472.3, AL358472.4, AL365181.2, AL365181.3, AL391987.2, AL451123.2, AL512408.1, AL512625.3, AL589666.1, AL590004.3, AL590666.2, AL592211.2, AL662797.1, AL662907.1, AL732314.6, ALDH18A1, ALDH1A1, ALDH1B1, ALDH1L1, ALG1, ALG6, ALG9, ALKBH2, ALMS1-IT1, ALS2CL, ALYREF, AMACR, AMMECR1, AMMECR1L, ANAPC1P2, ANGPTL2, ANKH, ANKLE2, ANKRD35, ANP32A, ANP32E, ANXA11, ANXA7, AP000251.1, AP000255.1, AP000553.2, AP001107.9, AP001453.3, AP002026.1, AP002495.1, AP002990.1, AP006545.1, AP3D1, APEX1, APOL4, ARAP3, ARHGAP11A, ARHGAP11B, ARHGAP19, ARHGAP26, ARHGDIA, ARHGEF2, ARHGEF39, ARL1, ARL16, ARL4A, ARL6IP6, ARL8B, ARMC6, ARMC7, ARNTL2, ASF1B, ASH1L-AS1, ASNSD1, ASPM, ASTE1, ATAD2, ATAD3A, ATAD5, ATG12, ATP2A1-AS1, ATP5PF, ATP6V0A2, ATP6V1G1, ATPAF2, ATRIP, AUNIP, AURKA, AURKB, AXIN1, B3GNT10, B4GALT2, B4GALT3, BANK1, BANP, BARD1, BARHL1, BAZ1A, BCAR3, BCAT1, BCL2L11, BDH2, BEND3, BFAR, BHLHE41, BHMT2, BIRC5, BIVM, BLMH, BLOC1S2, BNIP3P11, BNIP3P17, BOD1, BOLA2P2, BOLA2-SMG1P6, BORA, BORCS7, BPHL, BPNT1, BRCA1, BRCA2, BRD1, BRD4, BRIP1, BRPF1, BTF3L4, BTG3-AS1, BUB1, BUB1B, BUB3, BUD13, BX323046.2, BX842570.1, BYSL, BZW1, BZW2, C10orf143, C11orf1, C11orf94, C12orf65, C14orf93, C15orf41, C16orf89, C17orf58, C18orf21, C18orf54, C19orf47, C19orf57, C1GALT1C1L, C1orf112, C1orf131, C1orf56, C1orf94, C1QL4, C20orf144, C21orf58, C2orf68, C3orf38, C4orf3, C4orf46, C5orf34, C8orf31, C8orf82, C8orf88, CA3-AS1, CABLES2, CACFD1, CAD, CAPN5, CARHSP1, CARMIL1, CASP2, CBLB, CBX2, CBX8, CCAR2, CCDC112, CCDC134, CCDC137, CCDC138, CCDC142, CCDC15, CCDC150, CCDC167, CCDC18, CCDC188, CCDC28B, CCDC34, CCDC43, CCDC77, CCDC84-DT, CCDC86, CCDC91, CCDC97, CCHCR1, CCNA2, CCNB1, CCNB1IP1, CCNB2, CCNDBP1, CCNE1, CCNE2, CCNF, CCNJL, CCNYL1, CCPG1, CCT2, CCT3, CCT4, CCT5, CCT6A, CCT6P1, CCT7, CD2BP2, CD59, CDC20, CDC20P1, CDC23, CDC25A, CDC25B, CDC25C, CDC42SE2, CDC45, CDC6, CDC7, CDCA2, CDCA3, CDCA4, CDCA5, CDCA7, CDCA7L, CDCA8, CDH23, CDH24, CDK1, CDK2, CDK4, CDK6, CDK8, CDKAL1, CDKN2C, CDKN3, CDT1, CDYL, CEBPA-DT, CEBPZ, CENPA, CENPE, CENPF, CENPH, CENPI, CENPJ, CENPK, CENPL, CENPM, CENPN, CENPO, CENPP, CENPQ, CENPU, CENPW, CENPX, CEP128, CEP131, CEP41, CEP55, CEP57L1, CEP72, CEP76, CEP83-DT, CEP85, CEP89, CERS5, CFAP20DC, CFAP410, CFDP1, CGNL1, CHAC2, CHAF1A, CHAF1B, CHAMP1, CHCHD10, CHD1, CHEK1, CHEK2, CHERP, CHIC2, CHMP1A, CHMP6, CHMP7, CHP1, CHRNA5, CHST11, CHTF18, CHTOP, CIAPIN1, CIP2A, CIZ1, CKAP2, CKAP2L, CKLF-CMTM1, CKMT2, CKS1B, CKS2, CLCN3P1, CLEC3B, CLEC9A, CLIP4, CLSPN, CLTRN, CLYBL, CNOT10, CNOT8, CNOT9, CNPY2, CNTLN, CNTROB, COL23A1, COMMD2, COP1, COPS3, COPS7B, CORO1C, COX10, COX7A2L, CPSF3, CPSF4, CPXM1, CRCP, CREB3L4, CREBL2, CRNDE, CRYL1, CRYZL2P, CSE1L, CSMD2, CSPG4, CSPG4P13, CSRP1, CST3, CSTF1, CSTF2, CSTF3, CTHRC1, CTNNAL1, CTPS1, CTSF, CTSV, CTU2, CU633906.2, CU634019.5, CU638689.1, CU638689.5, CXADR, CXCL14, CYLD, CYP11A1, CYP4F11, CYTH2, DBF4, DBP, DCAF12, DCAF13, DCAF16, DCBLD1, DCLRE1B, DCP2, DCPS, DCTN3, DCTPP1, DDIAS, DDIT4L, DDX10, DDX11, DDX11-AS1, DDX12P, DDX18, DDX21, DDX23, DDX27, DDX28, DDX31, DDX39B, DDX49, DDX50, DDX52, DEDD, DEK, DENND6A, DENR, DEPDC1, DEPDC1B, DESI2, DFFB, DGCR8, DGKD, DGLUCY, DGUOK, DHDDS-AS1, DHFR, DHFRP1, DHODH, DHRS12, DHRS13, DHX16, DIAPH1, DIAPH3, DIO2, DIPK2A, DKC1, DLEU1, DLEU2, DLGAP5, DMGDH, DNA2, DNAAF2, DNAJB5, DNAJC21, DNAJC9, DNMT1, DNMT3B, DOK4, DOLK, DOLPP1, DONSON, DOT1L, DPF2, DPH2, DPH5, DPM2, DPP3, DPYSL3, DRAXIN, DSCC1, DSEL, DSN1, DTD1, DTL, DTYMK, DUS2, DUS3L, DUSP12, DUSP22, DUSP5P1, DUXAP8, DUXAP9, DVL2, DVL3, DYRK2, E2F1, E2F2, E2F3, E2F5, E2F6, E2F7, E2F8, EBF4, ECT2, EDA, EDC3, EDN3, EEF2KMT, EEFSEC, EEPD1, EFNA2, EFTUD2, EGFEM1P, EGFL7, EHD1, EHMT1, EI24, EIF1AD, EIF2A, EIF2AK1, EIF2AK3-DT, EIF2B1, EIF2B3, EIF2S1, EIF4A3, ELAC1, ELAC2, ELAVL1, ELFN1, ELMOD2, ELOA, ELP5, EMC2, EMC3-AS1, EMC8, EME1, EN1, EOLA2, EPB41L4A-DT, EPHB2, EPHB3, EPHX2, ERAL1, ERCC6L, ERLIN1, ESCO1, ESCO2, ESPL1, ESS2, EXO1, EXOC3, EXOG, EXOSC10, EXOSC2, EXOSC3, EXOSC9, EXTL3, EZH2, FAAP100, FAAP24, FAF1, FAM110A, FAM111B, FAM122C, FAM126A, FAM133B, FAM136A, FAM174A, FAM27E3, FAM32A, FAM72A, FAM72B, FAM72C, FAM72D, FAM83D, FAM83H, FAM89B, FANCA, FANCB, FANCC, FANCD2, FANCE, FANCG, FANCI, FANCM, FARSB, FBXO42, FBXO43, FBXO5, FDXACB1, FEN1, FENDRR, FGD4, FGF1, FGF20, FHDC1, FIGNL1, FIP1L1, FLAD1, FLOT2, FLVCR1-DT, FMO2, FOXD1, FOXD2-AS1, FOXD3, FOXD3-AS1, FOXM1, FOXO3B, FOXP1, FREM2, FSCN1, FTSJ1, FTSJ3, FUS, FXYD1, FZR1, G2E3, G6PC3, GABPB1, GADD45G, GALE, GALR1, GART, GAS1, GAS2L3, GASAL1, GATA2, GATA2-AS1, GBX2, GCFC2, GCN1, GCNT2, GEMIN2, GEMIN4, GEMIN6, GEN1, GFAP, GFER, GGH, GGPS1, GID8, GINS1, GINS2, GINS3, GINS4, GJA1, GJC1, GLDC, GLE1, GLOD4, GLUL, GLYCTK-AS1, GMEB1, GMEB2, GMNN, GMPS, GNA14, GNB4, GNL3L, GNL3LP1, GNPAT, GOLGA8N, GOLGA8O, GORASP2, GOSR2, GPANK1, GPAT2, GPATCH2, GPC1, GPC2, GPR146, GPR156, GPR161, GPT, GPX7, GRINA, GRK6, GSX1, GTF2E1, GTF2F2, GTF2H4, GTF2IRD1, GTF2IRD2B, GTF3C2, GTF3C5, GTPBP6, GTPBP8, GTSE1, H1-10, H1-3, H19, H2AC11, H2AC13, H2AC16, H2AC17, H2AX, H2AZ1, H2BC17, H2BC9, H2BW3P, H3-3A, H3C2, H3C3, H3P6, HAND2, HARBI1, HAS2-AS1, HASPIN, HAT1, HAUS1, HAUS2, HAUS3, HAUS6, HAUS8, HCG11, HCG15, HDAC2, HDAC8, HDGF, HDGFL2, HDHD5, HEATR3, HEBP1, HELLS, HEPH, HEY1, HHAT, HIGD1A, HJURP, HMGA1, HMGB1, HMGB1P6, HMGB2, HMGB3, HMGN1, HMGN2, HMGXB4, HMMR, HNRNPA0, HNRNPA1, HNRNPA1P10, HNRNPA1P16, HNRNPA1P48, HNRNPA1P7, HNRNPA2B1, HNRNPA3, HNRNPA3P6, HNRNPAB, HNRNPC, HNRNPD, HNRNPH1, HNRNPH3, HNRNPK, HNRNPL, HNRNPLP2, HNRNPM, HNRNPR, HNRNPU, HNRNPUL1, HOGA1, HOXA10, HOXA11, HOXA6, HOXA-AS3, HOXC4, HOXD10, HOXD11, HOXD13, HOXD3, HOXD4, HOXD8, HOXD9, HOXD-AS2, HPF1, HPR, HPS1, HROB, HSBP1, HSD11B1, HSD17B14, HSPB2, HTRA1, HTRA2, HYKK, IDH1, IDH2, IDH3A, IER3IP1, IFT52, IGF2BP3, IGIP, IGSF3, IGSF9, ILF2, ILF3, INCENP, ING1, ING4, INO80C, INSM1, INSYN2A, INTS1, INTS10, INTS13, INTS4, INTS5, INTS7, INTS8, INTS9, IPO5, IQCC, IQGAP3, IRX5, ISCU, ISL2, IST1, ITM2B, ITPKB, ITPRIPL1, IWS1, IZUMO4, JADE2, JMJD1C-AS1, JPT1, JPT2, KANSL2, KBTBD2, KCTD20, KCTD21-AS1, KCTD5, KDM1A, KDM2B, KDM4B, KDM6B, KHDRBS1, KIF11, KIF14, KIF15, KIF16B, KIF18A, KIF18B, KIF20A, KIF20B, KIF22, KIF23, KIF24, KIF2C, KIF4A, KIF7, KIFC1, KLF9, KLHDC1, KLHL25, KMT5A, KNL1, KNOP1, KNSTRN, KNTC1, KPNA2, KPNB1, KRT8P39, KTI12, LAGE3P1, LAMA1, LANCL3, LAPTM4B, LBH, LBR, LCORL, LDAH, LDHD, LEMD1, LETMD1, LGI4, LGR6, LIG1, LIMA1, LIMD1, LIMD1-AS1, LIMD2, LIN9, LINC00511, LINC00526, LINC00664, LINC00900, LINC01224, LINC01560, LINC01578, LINC01637, LINC01670, LINC01679, LINC01778, LINC01816, LINC02134, LINC02256, LINC02293, LINC02588, LIX1L, LLGL2, LMBRD1, LMNB1, LMNB2, LMO1, LNPEP, LONRF1, LPIN3, LRFN4, LRR1, LRRC37BP1, LRRC41, LRRC42, LRRC45, LRRC59, LSM12, LSM12P1, LSM14B, LSM4, LSM8, LTV1, LYNX1-SLURP2, LYSMD2, LZIC, MACROH2A1, MAD1L1, MAD2L1, MAD2L1BP, MAD2L2, MAGEF1, MAGOHB, MAK16, MAML1, MAP1LC3B, MAP3K5, MAP4, MAPK1IP1L, MAPKAP1, MAPKAPK5, MARCKS, MAST4, MASTL, MAT2B, MATN4, MAZ, MBOAT7, MCM10, MCM2, MCM3, MCM4, MCM5, MCM6, MCM7, MCM8, MCMBP, MCPH1, MCRS1, MDFI, MED17, MED19, MED20, MED26, MED27, MED30, MELK, MEMO1, MEN1, MEST, METAP2, METTL22, METTL2B, MEX3A, MFAP3L, MFSD11, MFSD9, MGST3, MID1IP1-AS1, MINPP1, MIR181A2HG, MIR29B2CHG, MIR4453HG, MIR503HG, MIR924HG, MIS12, MIS18A, MIS18BP1, MKI67, MKRN1, MKRN3, MKS1, MLH1, MLXIP, MLXIPL, MMP2, MMP24OS, MMS22L, MND1, MOSPD1, MPHOSPH10, MPHOSPH6, MPZ, MPZL1, MRE11, MROH7, MROH7-TTC4, MRPL20-DT, MRPL3, MRPL42, MRPL52, MRPL9, MRPS10, MRPS18A, MRPS22, MRPS30-DT, MRPS35, MRPS7, MRTO4, MSH2, MSH6, MSI1, MT1E, MT1G, MT3, MTA2, MTBP, MTDHP1, MTF2, MTFR2, MTHFD1, MUS81, MXD3, MYB, MYBBP1A, MYBL1, MYBL2, NAA60, NABP2, NACC2, NAPA, NARF, NASP, NBPF9, NCAPD2, NCAPD3, NCAPG, NCAPG2, NCAPH, NCAPH2, NCL, NCOA5, NDC1, NDC80, NDE1, NDOR1, NDUFAF1, NDUFB5, NEDD1, NEIL3, NEK2, NELFB, NELFCD, NEMP1, NEPRO, NETO2, NEURL1B, NFKBIL1, NFS1, NFYC, NGDN, NHEJ1, NKIRAS2, NKX3-2, NLE1, NME1, NMRK1, NMU, NOC2L, NOC3L, NOC4L, NODAL, NOL10, NOL11, NOL9, NONO, NOP14, NOP56, NOP58, NOP9, NOXA1, NPLOC4, NR1D1, NRARP, NRAS, NRM, NSD2, NSL1, NSMCE4A, NSUN4, NT5C3A, NT5DC2, NTN1, NUBP2, NUDCD1, NUDCD2, NUDT1, NUDT16, NUDT18, NUDT5, NUF2, NUFIP1, NUP107, NUP155, NUP160, NUP188, NUP205, NUP210, NUP35, NUP42, NUP43, NUP50, NUP85, NUP88, NUSAP1, NVL, ODC1, ODF2, OGFRL1, OIP5, OR8T1P, ORAI1, ORC1, ORC6, OSBP, OTP, OXSR1, P2RY2, P4HTM, PA2G4, PAFAH1B3, PAK1IP1, PAK2, PALM3, PAMR1, PANTR1, PANX1, PAQR5, PARD6G, PARD6G-AS1, PARL, PARP1, PARP11, PARP16, PARPBP, PASK, PAXIP1, PAXIP1-AS1, PBK, PBX3, PBXIP1, PCDHB10, PCDHB14, PCDHB16, PCDHB18P, PCDHB19P, PCDHB2, PCDHB8, PCDHB9, PCDHGB8P, PCGF2, PCGF5, PCGF6, PCIF1, PCLAF, PCMTD1, PCNA, PCNPP1, PCNX3, PDCD11, PDCD2, PDE7A, PDE9A-AS1, PDIK1L, PDSS1, PELI1, PES1, PEX13, PFAS, PGAM1P7, PGAM5, PGBD1, PGBD2, PGD, PGM3, PHACTR2, PHACTR4, PHB, PHETA1, PHF13, PHF19, PHF21B, PHLDA1, PHOSPHO2, PHRF1, PHTF1, PHTF2, PHYHD1, PIAS4, PIF1, PIGF, PIGP, PIGU, PIGW, PIK3CD-AS2, PIMREG, PINK1-AS, PINX1, PIP4P2, PITPNA-AS1, PKMYT1, PKN3, PLA2G4C, PLAAT3, PLAG1, PLAT, PLCXD1, PLEKHA8, PLK1, PLK4, PLPP5, PM20D2, PMF1, POC1A, POGLUT1, POGLUT2, POLA1, POLA2, POLD3, POLDIP2, POLE, POLE2, POLE3, POLH, POLH-AS1, POLQ, POLR1C, POLR1E, POLR2D, POLR3C, POLR3D, POLR3F, POLR3GL, POP1, PPAN, PPHLN1, PPIH, PPIL1, PPIL4, PPM1G, PPP1CC, PPP1R10, PPP1R15B, PPP1R3C, PPP1R3D, PPP1R3G, PPP1R8, PPP2R2A, PPP2R3B, PPP4R1, PPP4R2, PPRC1, PRC1, PRCC, PRDM15, PREX2, PRIM1, PRIM2, PRIMPOL, PRKAB1, PRKAG1, PRKRA, PRMT5, PRMT6, PROSER1, PROSER3, PROX1, PRPF4, PRPF6, PRR11, PRR19, PRR3, PRX, PSMB2, PSMB7, PSMC3IP, PSMD11, PSMD14, PSMD3, PSMD5, PSMD6, PSME3, PSRC1, PTMA, PTMS, PTTG1, PUF60, PUS3, PUSL1, PWP1, PXMP2, PYCR1, PYGO2, QRFPR, QRICH1, QSOX2, RAB37, RAB4B, RABEPK, RACGAP1, RAD18, RAD23B, RAD51, RAD51AP1, RAD51D, RAD54B, RAD54L, RAE1, RAF1, RAN, RANBP1, RANBP3, RARA, RARA-AS1, RARS2, RBBP4, RBBP7, RBBP8, RBBP9, RBFA, RBKS, RBL1, RBM10, RBM12, RBM14, RBM15B, RBM19, RBM23, RBM28, RBM4, RBM8A, RBMX, RBMX2, RCC1, RCC2, RCN2, RCOR1, RDM1, RECQL4, REEP6, REPIN1, RETREG3, REXO4, REXO5, RFC2, RFC3, RFC4, RFC5, RFT1, RFWD3, RFX5-AS1, RGS14, RHNO1, RILP, RING1, RIOK1, RMI1, RMI2, RN7SL832P, RNASEH1-AS1, RNASEH2A, RNASEH2B, RNF122, RNF138, RNF168, RNF2, RNF214, RNF216P1, RNF4, RNF6, RNFT1, RNLS, RNPS1, ROBO1, RORA, RORB, RP9P, RPA1, RPA2, RPAIN, RPP38, RPP40, RPS20P22, RPS2P46, RPS6KA4, RPUSD1, RPUSD4, RRM1, RRM2, RRP15, RRP1B, RRS1, RTEL1, RTKN2, RTL8B, RTTN, RUVBL1, RWDD4, RYR3, S100PBP, SAAL1, SAC3D1, SAE1, SAFB, SAMD1, SAP18, SAPCD2, SART1, SASS6, SCARF2, SCFD2, SCLT1, SCLY, SCML2, SEC14L2, SEC14L6, SEC22A, SELENOM, SELENOW, SEMA5B, SENP1, SEPTIN10, SEPTIN9, SERBP1, SERGEF, SERHL2, SERTAD4-AS1, SET, SETD3, SETD7, SETDB1, SETDB2, SETMAR, SF3A2, SF3A3, SF3B2, SF3B3, SF3B4, SFMBT1, SFXN5, SGO1, SGO2, SH3BP4, SHCBP1, SHLD2, SHLD3, SHPK, SHQ1, SHROOM1, SIAH1, SIGMAR1, SIM2, SIRPA, SIX1, SIX2, SIX4, SKA1, SKA2, SKA3, SKP1, SKP2, SLBP, SLC16A1-AS1, SLC25A10, SLC25A15, SLC25A17, SLC27A1, SLC27A3, SLC29A2, SLC2A4, SLC35B1, SLC35B2, SLC37A3, SLC37A4, SLC38A7, SLC39A6, SLC44A3, SLC46A2, SLC5A6, SLC7A11, SLC7A2, SLC7A6, SLC9A3R2, SLCO4A1-AS1, SMAD2, SMAD4, SMARCA4, SMARCB1, SMARCC1, SMARCD1, SMARCE1, SMC1A, SMC2, SMC3, SMC4, SMG1P5, SMIM14, SMUG1, SNAPC5, SNAPIN, SNCAIP, SNF8, SNHG1, SNHG4, SNRNP40, SNRPA, SNRPA1, SNRPD1, SNRPGP10, SNX4, SOD1, SORL1, SOWAHC, SOX11, SOX12, SOX4, SPACA6, SPAG5, SPATA20, SPC24, SPC25, SPCS3, SPDL1, SPICE1, SPIN4, SPINDOC, SPINT2, SPOCK2, SPOUT1, SPRTN, SPSB4, SRBD1, SRM, SRP19, SRPK1, SRSF1, SRSF10, SRSF2, SRSF3, SRSF7, SS18, SSB, SSH3, SSRP1, ST3GAL2, ST7-AS1, ST7L, ST8SIA2, STAMBP, STEAP1B, STIL, STIP1, STON1, STPG1, STRN4, STX2, STX6, SULF2, SULT1A1, SUMO2, SUPT16H, SUPT20H, SURF6, SUSD1, SUV39H1, SUV39H2, SUZ12, SYCE2, SYNCRIP, SYNM, SYNPO2, TACC1, TACC3, TAF11, TAF15, TAF4, TAF5L, TAF6, TARDBP, TARS2, TATDN2, TBC1D10B, TBC1D16, TBC1D17, TBC1D31, TBC1D7, TBC1D9B, TBL3, TBP, TCF19, TCF7L1, TCOF1, TDG, TDP1, TDRD10, TEDC1, TEDC2, TENT4A, TEX30, TFAP2A, TFAP2A-AS1, TFAP2A-AS2, TFB2M, TFDP1, THAP11, THOC5, TIA1, TICRR, TIGD2, TIGD5, TIMELESS, TIMM22, TIMM29, TIMP2, TIPIN, TIPRL, TK1, TLCD3A, TMA16, TMC1, TMEM104, TMEM106C, TMEM131L, TMEM164, TMEM182, TMEM186, TMEM199, TMEM203, TMEM229A, TMEM39B, TMEM97, TMPO, TMPO-AS1, TMPOP2, TMSB15A, TMSB15B, TNFAIP1, TNFRSF21, TNNC2, TOE1, TOMM40P4, TOMM5, TONSL, TOP1MT, TOP2A, TOP3A, TOPBP1, TOR1B, TOR2A, TP73, TP73-AS1, TPCN1, TPI1P2, TPX2, TRA2B, TRAF2, TRAF4, TRAF7, TRAFD1, TRAIP, TRIB2, TRIM11, TRIM16, TRIM24, TRIM27, TRIM28, TRIP13, TRMT10C, TRMT5, TRMT61B, TRNT1, TROAP, TRPM3, TSEN15, TSN, TSPAN18, TTC5, TTC9C, TTF1, TTF2, TTI1, TTI2, TTK, TUBA1A, TUBA1B, TUBB, TUBG1, TUBGCP3, TWNK, TYMS, TYW3, U2AF2, UBA1, UBA6, UBAP2, UBE2C, UBE2E3, UBE2I, UBE2J1, UBE2Q1, UBE2R2, UBE2S, UBE2SP1, UBE2T, UBL7-AS1, UBTD2, UBTF, UBXN2A, UCK2, UFD1, UGDH, UGGT1, UHRF1, UHRF2, UIMC1, ULBP1, UMPS, UNC5B-AS1, UNG, UPK2, USB1, USF1, USP1, USP13, USP21, USP25, USP36, USP39, USP53, UTP11, UTP18, VAC14, VANGL1, VARS1, VASH1, VASH2, VAX2, VBP1, VCP, VEPH1, VHL, VPS16, VPS25, VPS37B, VPS72, VRK1, WAKMAR2, WDCP, WDHD1, WDR33, WDR34, WDR4, WDR5, WDR54, WDR5B, WDR62, WDR70, WDR74, WDR76, WDR77, WDR89, WRAP53, WRN, WWC2-AS2, WWC3, XPNPEP1, XPO5, XRCC1, XRCC2, XRCC4, XRCC5, XRCC6, XXYLT1, YBX1, YBX1P1, YBX1P10, YES1, YIPF3, YME1L1, YRDC, YTHDF2, YWHAQ, YY1AP1, ZBED1, ZBED4, ZBTB17, ZBTB2, ZBTB8A, ZBTB9, ZC3H15, ZC3H3, ZC3HAV1L, ZCCHC7, ZDHHC16, ZEB1-AS1, ZFYVE21, ZFYVE28, ZGRF1, ZHX1-C8orf76, ZIC1, ZIK1, ZKSCAN5, ZKSCAN7, ZMYM1, ZMYND19, ZNF101, ZNF114, ZNF124, ZNF134, ZNF143, ZNF16, ZNF2, ZNF202, ZNF212, ZNF22, ZNF232, ZNF274, ZNF28, ZNF282, ZNF286B, ZNF3, ZNF300, ZNF311, ZNF316, ZNF324B, ZNF326, ZNF35, ZNF367, ZNF385A, ZNF398, ZNF416, ZNF467, ZNF473, ZNF486, ZNF491, ZNF496, ZNF512B, ZNF513, ZNF528-AS1, ZNF530, ZNF544, ZNF551, ZNF559, ZNF574, ZNF578, ZNF584, ZNF586, ZNF589, ZNF598, ZNF625, ZNF628, ZNF639, ZNF668, ZNF669, ZNF674-AS1, ZNF687-AS1, ZNF695, ZNF7, ZNF71, ZNF724, ZNF726, ZNF736, ZNF738, ZNF75A, ZNF764, ZNF77, ZNF777, ZNF788P, ZNF8, ZNF813, ZNF830, ZNF845, ZNF85, ZNF878, ZNF887P, ZNF891, ZNF90, ZNF92, ZNF93, ZNRD2-AS1, ZW10, ZWILCH, ZWINT | |
| Red module  (ME6) | | AATK, ABCA2, ABCA8, ABCF3, ABHD12B, ABHD17B, ABHD6, ABTB2, AC004241.1, AC004466.1, AC004925.1, AC004980.1, AC005329.3, AC006059.1, AC007036.3, AC007114.1, AC007546.2, AC008438.2, AC009063.2, AC009779.2, AC010175.1, AC010378.1, AC010531.1, AC011504.1, AC011825.3, AC012150.2, AC012158.1, AC012459.1, AC012511.1, AC015712.1, AC015712.6, AC016397.2, AC016597.1, AC018521.5, AC018647.1, AC021660.4, AC023024.1, AC023024.2, AC026316.4, AC026691.1, AC068385.1, AC068888.1, AC068896.1, AC069209.2, AC069547.1, AC083809.1, AC084880.1, AC087273.2, AC090515.2, AC090772.3, AC090994.1, AC091982.3, AC092117.1, AC092119.3, AC092809.2, AC093330.1, AC093330.2, AC093503.2, AC093627.7, AC095055.1, AC099518.2, AC104083.1, AC105383.1, AC106791.1, AC106881.1, AC107375.1, AC109635.7, AC110285.1, AC113133.1, AC117415.1, AC127459.3, AC131160.1, AC133785.1, AC135050.3, AC135507.1, AC135893.1, AC138409.2, AC142086.6, AC142381.3, AC188616.1, AC241377.2, AC245297.2, ACOT12, ACOX3, AD001527.2, ADAMTS4, ADAMTS9-AS2, ADAMTSL2, ADAP1, ADIPOR2, ADIRF, ADM2, ADPRM, ADRA2B, AFMID, AGMAT, AIDAP2, AIF1L, AK5, AKAP3, AKT1, AKT2, AL023284.4, AL024508.1, AL031056.1, AL031121.2, AL031651.2, AL035106.1, AL035587.2, AL078590.2, AL078596.1, AL078622.1, AL109628.2, AL118558.3, AL118558.4, AL121612.2, AL133304.3, AL135960.1, AL139353.1, AL139407.1, AL162511.1, AL353751.1, AL354811.1, AL358216.1, AL359091.1, AL359091.2, AL390726.4, AL391244.1, AL513190.1, AL589787.2, AL596244.1, AL928654.4, ALAD, ALKAL2, AMER2, ANGEL1, ANKRD13A, ANKRD40, ANKRD65, ANLN, ANO4, AOPEP, AP000439.2, AP000547.3, AP000941.1, AP000944.7, AP001350.2, AP003390.1, AP004609.3, APLNR, APLP1, APLP2, APOD, ARHGAP22, ARHGAP23, ARHGEF16, ARHGEF37, ARRDC2, ASAP3, ASB4, ASB6, ASPA, ASPHD1, ATG4C, ATP8A1, AZIN2, B4GALT5, BACE1, BBIP1, BCAS1, BCAS2, BDNF-AS, BEST1, BIN1, BMP8A, BOC, BOK, BTBD16, BX323043.1, C10orf90, C11orf52, C12orf75, C12orf76, C1orf198, C21orf91, C22orf46, C2CD4D, C5orf64, CA13, CA2, CACTIN, CAMK2D, CAPN3, CARNS1, CBR1, CCNI2, CCP110, CCT8, CD22, CDC42EP2, CDH19, CDK18, CDK7, CDKN1C, CDR2, CDR2L, CEP20, CERCAM, CETN4P, CFL2, CHADL, CHN2, CHPF, CLCA4, CLDN11, CLDN12, CLDN5, CLDND1, CLEC19A, CLMN, CLN8, CMC2, CMTM5, CNDP1, CNIH3-AS1, CNP, CNR1, CNTF, CNTN2, CNTNAP4, COBL, COL27A1, COL4A5, COPB2, CORO7, CORO7-PAM16, COX6A2, CPB2-AS1, CPM, CPOX, CPPED1, CRNKL1, CRYAB, CSNK1A1, CSNK1D, CTBP2P10, CTBP2P9, CTNNA3, CUL1, CUTC, CXorf56, CYB5R2, CYP2J2, CYP2T1P, CYP2U1-AS1, CYP7B1, CYTH1, DAAM2, DAAM2-AS1, DAPK2, DBNDD2, DCP1B, DDX41, DEPTOR, DHCR24, DHDDS, DHRS9, DIPK1A, DLG1, DNAH17, DNAJB2, DNAJC15, DNAJC2, DNPEP, DNTTIP2, DOCK1, DOCK5, DOCK7, DOHH, DOK5, DPEP3, DPYSL2, DUSP7, EDIL3, EEF2K, EFHD1, EFL1, EFNB1, EHBP1, EHF, ELAPOR2, ELOVL1, ELOVL7, EML2, ENPP2, ENPP4, ENPP6, ENTPD3-AS1, EPB41L2, EPM2A, ERGIC1, ERMN, ERMP1, ETV4, EVI2A, EYA2, EYA4, FA2H, FAAH, FABP7, FAHD2B, FAHD2CP, FAM102A, FAM107B, FAM124A, FAM13C, FAM149B1, FAM171A1, FAM177A1, FAM178B, FAM200A, FAM201A, FAM210B, FAM53A, FAM53B, FAM86HP, FAM95C, FAM98A, FAR1, FARS2, FASTKD5, FAXDC2, FBLN7, FBN2, FBXO7, FBXW4, FCHO1, FEZ1, FGFR1, FGFR2, FKBP9, FMNL2, FN3K, FNBP1, FNTA, FNTB, FOLH1, FOXO4, FRA10AC1, FRK, FRMD4B, FTCD, FUT8, FUT8-AS1, FZD1, GAB2, GAL3ST1, GALNT7, GAREM1, GARS1, GCLC, GDE1, GGCT, GGCX, GJB1, GJC2, GLDN, GLTP, GNG7, GPD1, GPIHBP1, GPR3, GPR37, GPR39, GPR62, GREM1, GRID1, GRM3, GSC, GSN-AS1, GTF2F1, HAGLR, HAPLN2, HAS3, HBS1L, HCCS, HCN2, HDAC11, HDAC3, HDLBP, HERPUD2, HES1, HHATL, HHIP, HHIP-AS1, HIPK2, HKDC1, HMGXB3, HMSD, HOXB3, HOXB4, HOXD1, HPN, HPSE2, HS2ST1, HS3ST3A1, HS3ST5, HSD17B4, HSPA2, HSPB8, ICAM2, ID2-AS1, IGF2BP2, IGSF10, IGSF8, IKZF5, INF2, INPP1, IP6K3, IPO13, IQCB1, IQCJ-SCHIP1, IRS1, IRX3, ISYNA1, ITM2A, ITPK1, JAKMIP3, KARS1, KBTBD12, KCNH8, KCNJ2, KCNJ2-AS1, KCNMB4, KCTD14, KEL, KIAA0930, KIAA1755, KIF13B, KIF1C, KIF6, KLHL2, KLHL32, KLK6, KRT17P1, KRT17P2, KYAT3, LACC1, LANCL1, LARP6, LDB3, LDLRAD4, LDLRAP1, LGI3, LGR5, LHPP, LILRA4, LIMCH1, LINC00320, LINC00323, LINC00578, LINC00639, LINC00706, LINC00844, LINC00863, LINC00891, LINC00987, LINC01135, LINC01141, LINC01315, LINC01338, LINC01356, LINC01549, LINC01554, LINC01711, LINC01978, LINC02473, LINC02525, LINC02610, LINC02712, LIPE, LLGL1, LMAN2L, LMF1-AS1, LMOD3, LONP1, LPAR1, LPCAT1, LRIF1, LRP1, LRP2, LRRC39, LRRC63, LRRC8C-DT, LRRC8D, LSMEM2, LSS, LYPD1, LZTS2, MAFG-DT, MAG, MAIP1, MAL, MALT1, MAN2A1-DT, MAP4K4, MAP6D1, MAP7, MAP7D1, MAPRE2, MBNL2, MBP, MCC, MCFD2, METTL21A, MFAP1, MGC16275, MINDY1, MOBP, MOCS3, MOG, MOSPD2, MRPL48, MRPL49, MRPS2, MRPS6, MSLN, MTCO1P12, MTERF1, MTMR10, MTND2P28, MTURN, MTUS1, MUL1, MVB12B, MYH14, MYL3, MYLK, MYO18A, MYO1D, MYOT, MYOZ1, MYRF, NAA50, NAB2, NACAD, NAIF1, NANP, NEK7, NIFK-AS1, NINJ2, NIPA1, NIPAL3, NIPAL4, NKAIN2, NKAPL, NKX6-2, NLRP4, NOB1, NPC1, NPHP3-ACAD11, NR2F6, NRBP2, NT5DC1, NT5E, NTM-AS1, NUB1, NUCB2, NUMBL, NUTM2A, NUTM2A-AS1, NUTM2D, NXPE3, OAZ2, OLMALINC, OMG, OPALIN, ORAI2, ORC5, OSBPL1A, OTOS, OTUD7A, OTUD7B, P2RY12, PACRG-AS3, PACS2, PADI2, PAIP2B, PAOX, PAQR4, PAQR6, PAQR8, PCGF1, PCSK6, PDCD4, PDE1C, PDE6B, PDE8A, PDIA2, PDK4, PEX5L, PHACTR3-AS1, PI16, PIEZO2, PIK3IP1, PIP4K2A, PKIB, PKP4, PLA2G7, PLCH2, PLCL1, PLD1, PLEKHB1, PLEKHG3, PLEKHH1, PLLP, PLP1, PLPP1, PLPP2, PLXNB3, PMPCA, PMS2P3, POFUT1, PPA1, PPFIBP2, PPP1R14A, PPP2R5A, PRCD, PRIMA1, PRKCQ, PRKCQ-AS1, PROX1-AS1, PRR18, PRRG1, PSEN1, PTGDS, PTGFRN, PTK7, PTPN9, PTPRH, PTPRK, PUS7, PXK, QDPR, RAB40B, RAB6C-AS1, RAPGEF3, RAPGEF5, RARS1, RASEF, RASGEF1C, RASGRF1, RASGRP3, RASL11A, RASL12, RASSF2, RBP7, RCN1, RELA-DT, RETREG1, RFFL, RGCC, RHBDL2, RHOB, RHOU, RHPN2, RIC8A, RILPL1, RINT1, RNF125, RNF13, RNF130, RNF141, RNF220, ROGDI, RPL17-C18orf32, RPRD1B, RPS10P7, RPS6KA2, RSL1D1, S100A1, S100B, S1PR5, SALL4, SCD, SCN1B, SDK1, SEC14L5, SEC23B, SECISBP2L, SEL1L3, SELENON, SELENOP, SEMA3B, SEMA3B-AS1, SEMA4D, SEMA7A, SEPTIN4, SEPTIN8, SFR1, SFRP1, SFTPC, SFXN2, SGK2, SGK3, SGMS1, SGPP1, SH3GL3, SH3GLB2, SH3PXD2A, SH3TC2, SHISA2, SHISA4, SHROOM4, SHTN1, SIPA1L1, SIRT2, SLAIN1, SLC10A4, SLC12A2, SLC1A7, SLC20A2, SLC22A15, SLC24A2, SLC25A38, SLC31A2, SLC35D1, SLC35E1, SLC38A4, SLC3A2, SLC44A1, SLC45A3, SLC48A1, SLC5A11, SLC5A3, SLCO1A2, SLCO3A1, SMIM10L1, SMIM5, SMIM6, SNIP1, SNORC, SNX29, SNX30, SORT1, SOX2-OT, SPART, SPATA2L, SPIDR, SPNS2, SPOCK3, SPTLC2, SRARP, SRCIN1, SSBP2, ST18, ST3GAL5-AS1, ST6GALNAC3, ST7, STK39, STOML2, STRN, SUN2, SVIP, SYNDIG1, SYNE3, SYNJ2, SYT9, TAL1, TANGO2, TARS3, TASP1, TBC1D12, TCEAL3, TCFL5, TCTN3, TDRD6, TESK2, TF, TFEB, TGFA, TIAF1, TJAP1, TJP2, TLCD5, TLE4, TLL2, TM6SF2, TM9SF4, TMC7, TMCC2, TMCC3, TMEM125, TMEM139, TMEM144, TMEM151A, TMEM158, TMEM159, TMEM178A, TMEM184B, TMEM191A, TMEM235, TMEM248, TMEM250, TMEM263, TMEM273, TMEM31, TMEM63A, TMEM88B, TMEM98, TMIGD2, TMTC2, TMTC4, TNFSF18, TNRC6C-AS1, TNS2, TOM1L1, TOR1A, TP53INP2, TP53RK, TP53TG5, TPPP, TPRN, TPTEP1, TRAF3IP2, TRAK2, TRGC1, TRIM25, TRIM41, TRIM59, TRPM6, TRPV3, TSHZ3, TSPAN15, TSPAN8, TTC4, TTLL11, TTYH2, TTYH3, TUBB4A, TWF1P1, TXNDC15, TXNRD3, TYMSOS, TYW1, U2AF1L5, UBE2V1, UGT8, ULK2, UNC5C, USH1C, USP31, USP32P2, USP54, UTP14A, UVRAG, VAV2, VAV3, VGLL4, VRK2, VSTM2B, VWA1, WHRN, WIPI2, WNK1, XRN2, YARS1, YEATS2-AS1, YPEL2, ZBTB47, ZCCHC24, ZDHHC11, ZDHHC11B, ZDHHC2, ZDHHC20, ZDHHC9, ZEB2, ZEB2-AS1, ZFP57, ZFYVE16, ZNF488, ZNF536, ZNF582, ZNF602P | |

**Table S7.** A summary of the pathways enriched by Radscore-related modules. Enrichment analysis was performed using the R package clusterProfiler, querying the following annotated gene set databases: Kyoto Encyclopedia of Genes and Genomes (KEGG), Hallmark, Reactome, BioCarta, Pathway Interaction Database (PID), WikiPathways. False discovery rate (FDR)-adjusted hypergeometric *P* < 0.01 indicated significant enrichment.

| **Pathway** | ***P*** value | **FDR** | **Genes** | **Count** | **Database** |
| --- | --- | --- | --- | --- | --- |
| REACTOME_CELL_CYCLE | 4.87E-100 | 1.05E-96 | ABL1/ANKLE2/ATRIP/AURKA/AURKB/BARD1/BIRC5/BORA/BRCA1/BRCA2/BRIP1/BUB1/BUB1B/BUB3/CCNA2/CCNB1/CCNB2/CCNE1/CCNE2/CDC20/CDC23/CDC25A/CDC25B/CDC25C/CDC45/CDC6/CDC7/CDCA5/CDCA8/CDK1/CDK2/CDK4/CDK6/CDKN2C/CDT1/CENPA/CENPE/CENPF/CENPH/CENPI/CENPJ/CENPK/CENPL/CENPM/CENPN/CENPO/CENPP/CENPQ/CENPU/CENPW/CENPX/CEP131/CEP41/CEP72/CEP76/CHEK1/CHEK2/CHMP6/CHMP7/CHTF18/CKS1B/CLSPN/COP1/DBF4/DCTN3/DHFR/DKC1/DNA2/DSCC1/DSN1/E2F1/E2F2/E2F3/E2F5/E2F6/ERCC6L/ESCO1/ESCO2/ESPL1/EXO1/FBXO5/FEN1/FOXM1/FZR1/GINS1/GINS2/GINS3/GINS4/GMNN/GORASP2/GTSE1/H2AX/H2AZ1/H2BC17/H2BC9/H3-3A/H3C2/H3C3/HAUS1/HAUS2/HAUS3/HAUS6/HAUS8/HDAC8/HJURP/HMMR/INCENP/IST1/KIF18A/KIF20A/KIF23/KIF2C/KMT5A/KNL1/KNTC1/KPNB1/LBR/LIG1/LIN9/LMNB1/LPIN3/MAD1L1/MAD2L1/MASTL/MCM10/MCM2/MCM3/MCM4/MCM5/MCM6/MCM7/MCM8/MCPH1/MIS12/MIS18A/MIS18BP1/MLH1/MND1/MRE11/MYBL2/NCAPD2/NCAPD3/NCAPG/NCAPG2/NCAPH/NCAPH2/NDC1/NDC80/NDE1/NEDD1/NEK2/NSD2/NSL1/NUF2/NUP107/NUP155/NUP160/NUP188/NUP205/NUP210/NUP35/NUP42/NUP43/NUP50/NUP85/NUP88/ODF2/OIP5/ORC1/ORC6/PCNA/PHLDA1/PIAS4/PIF1/PKMYT1/PLK1/PLK4/PMF1/POLA1/POLA2/POLD3/POLE/POLE2/POLE3/POLR2D/PPP1CC/PPP2R2A/PPP2R3B/PRIM1/PRIM2/PSMB2/PSMB7/PSMC3IP/PSMD11/PSMD14/PSMD3/PSMD5/PSMD6/PSME3/PTTG1/RAD51/RAE1/RAN/RBBP4/RBBP7/RBBP8/RBL1/RCC1/RCC2/RFC2/RFC3/RFC4/RFC5/RHNO1/RMI1/RMI2/RNF168/RPA1/RPA2/RRM2/RTEL1/RUVBL1/SET/SGO1/SGO2/SHQ1/SKA1/SKA2/SKP1/SKP2/SMC1A/SMC2/SMC3/SMC4/SPC24/SPC25/SPDL1/SYCE2/TFDP1/TK1/TMPO/TOP2A/TOP3A/TOPBP1/TPX2/TUBA1A/TUBA1B/TUBB/TUBG1/TUBGCP3/TYMS/UBE2C/UBE2I/UBE2S/UIMC1/VRK1/WRAP53/WRN/YWHAQ/ZNF385A/ZW10/ZWILCH/ZWINT | 263 | REACTOME |
| HALLMARK_E2F_TARGETS | 2.70E-94 | 2.90E-91 | ANP32E/ASF1B/ATAD2/AURKA/AURKB/BARD1/BIRC5/BRCA1/BRCA2/BUB1B/CCNB2/CCNE1/CDC20/CDC25A/CDC25B/CDCA3/CDCA8/CDK1/CDK4/CDKN2C/CDKN3/CENPE/CENPM/CHEK1/CHEK2/CKS1B/CKS2/CNOT9/CSE1L/CTPS1/DCLRE1B/DCTPP1/DEK/DEPDC1/DIAPH3/DLGAP5/DNMT1/DONSON/DSCC1/E2F8/EIF2S1/ESPL1/EZH2/GINS1/GINS3/GINS4/H2AX/H2AZ1/HELLS/HMGA1/HMGB2/HMGB3/HMMR/HNRNPD/ILF3/JPT1/KIF18B/KIF22/KIF2C/KIF4A/KPNA2/LBR/LIG1/LMNB1/MAD2L1/MCM2/MCM3/MCM4/MCM5/MCM6/MCM7/MELK/MKI67/MLH1/MMS22L/MRE11/MSH2/MXD3/MYBL2/NASP/NCAPD2/NME1/NOP56/NUP107/NUP205/ORC6/PA2G4/PCNA/PLK1/PLK4/POLA2/POLD3/POLE/PPP1R8/PRIM2/PSMC3IP/PTTG1/RACGAP1/RAD51AP1/RAN/RANBP1/RBBP7/RFC2/RFC3/RNASEH2A/RPA1/RPA2/RRM2/SLBP/SMC1A/SMC3/SMC4/SPAG5/SPC24/SPC25/SRSF1/SRSF2/SSRP1/SUV39H1/SYNCRIP/TACC3/TCF19/TIMELESS/TIPIN/TK1/TMPO/TOP2A/TRA2B/TRIP13/TUBB/TUBG1/UBE2S/UBE2T/UNG/USP1/XRCC6/ZW10 | 137 | HALLMARK |
| REACTOME_CELL_CYCLE_MITOTIC | 2.24E-84 | 1.60E-81 | ABL1/ANKLE2/AURKA/AURKB/BIRC5/BORA/BUB1/BUB1B/BUB3/CCNA2/CCNB1/CCNB2/CCNE1/CCNE2/CDC20/CDC23/CDC25A/CDC25B/CDC25C/CDC45/CDC6/CDC7/CDCA5/CDCA8/CDK1/CDK2/CDK4/CDK6/CDKN2C/CDT1/CENPA/CENPE/CENPF/CENPH/CENPI/CENPJ/CENPK/CENPL/CENPM/CENPN/CENPO/CENPP/CENPQ/CENPU/CEP131/CEP41/CEP72/CEP76/CHMP6/CHMP7/CKS1B/DBF4/DCTN3/DHFR/DNA2/DSN1/E2F1/E2F2/E2F3/E2F5/E2F6/ERCC6L/ESCO1/ESCO2/ESPL1/FBXO5/FEN1/FOXM1/FZR1/GINS1/GINS2/GINS3/GINS4/GMNN/GORASP2/GTSE1/H2AX/H2AZ1/H2BC17/H2BC9/H3-3A/H3C2/H3C3/HAUS1/HAUS2/HAUS3/HAUS6/HAUS8/HDAC8/HMMR/INCENP/IST1/KIF18A/KIF20A/KIF23/KIF2C/KMT5A/KNL1/KNTC1/KPNB1/LBR/LIG1/LIN9/LMNB1/LPIN3/MAD1L1/MAD2L1/MASTL/MCM10/MCM2/MCM3/MCM4/MCM5/MCM6/MCM7/MCM8/MCPH1/MIS12/MYBL2/NCAPD2/NCAPD3/NCAPG/NCAPG2/NCAPH/NCAPH2/NDC1/NDC80/NDE1/NEDD1/NEK2/NSL1/NUF2/NUP107/NUP155/NUP160/NUP188/NUP205/NUP210/NUP35/NUP42/NUP43/NUP50/NUP85/NUP88/ODF2/ORC1/ORC6/PCNA/PHLDA1/PKMYT1/PLK1/PLK4/PMF1/POLA1/POLA2/POLD3/POLE/POLE2/POLE3/PPP1CC/PPP2R2A/PPP2R3B/PRIM1/PRIM2/PSMB2/PSMB7/PSMD11/PSMD14/PSMD3/PSMD5/PSMD6/PSME3/PTTG1/RAE1/RAN/RBBP4/RBL1/RCC1/RCC2/RFC2/RFC3/RFC4/RFC5/RPA1/RPA2/RRM2/SET/SGO1/SGO2/SKA1/SKA2/SKP1/SKP2/SMC1A/SMC2/SMC3/SMC4/SPC24/SPC25/SPDL1/TFDP1/TK1/TMPO/TOP2A/TPX2/TUBA1A/TUBA1B/TUBB/TUBG1/TUBGCP3/TYMS/UBE2C/UBE2I/UBE2S/VRK1/ZW10/ZWILCH/ZWINT | 218 | REACTOME |
| HALLMARK_G2M_CHECKPOINT | 7.79E-80 | 4.18E-77 | ABL1/AURKA/AURKB/BARD1/BIRC5/BRCA2/BUB1/BUB3/CCNA2/CCNB2/CCNF/CDC20/CDC25A/CDC25B/CDC45/CDC6/CDC7/CDK1/CDK4/CDKN2C/CDKN3/CENPA/CENPE/CENPF/CHAF1A/CHEK1/CHMP1A/CKS1B/CKS2/DBF4/DKC1/DTYMK/E2F1/E2F2/E2F3/ESPL1/EXO1/EZH2/FANCC/FBXO5/GINS2/H2AX/H2AZ1/HMGA1/HMGB3/HMGN2/HMMR/HNRNPD/HNRNPU/ILF3/INCENP/JPT1/KIF11/KIF15/KIF20B/KIF22/KIF23/KIF2C/KIF4A/KMT5A/KNL1/KPNA2/KPNB1/LBR/LMNB1/MAD2L1/MARCKS/MCM2/MCM3/MCM5/MCM6/MKI67/MTF2/MYBL2/NASP/NCL/NDC80/NEK2/NSD2/NUP50/NUSAP1/ODC1/ODF2/ORC6/PBK/PLK1/PLK4/POLA2/POLE/POLQ/PRC1/PRIM2/PRMT5/PTTG1/RACGAP1/RAD23B/RAD54L/RBL1/RBM14/RPA2/SMARCC1/SMC1A/SMC2/SMC4/SNRPD1/SRSF1/SRSF10/SRSF2/SS18/STIL/SUV39H1/SYNCRIP/TACC3/TENT4A/TFDP1/TMPO/TOP2A/TPX2/TRA2B/TRAIP/TROAP/TTK/UBE2C/UBE2S/UCK2/WRN | 126 | HALLMARK |
| HALLMARK_INTERFERON_GAMMA_RESPONSE | 5.47E-57 | 1.49E-53 | APOL6/B2M/BATF2/BST2/C1R/C1S/CASP1/CASP4/CASP7/CASP8/CCL2/CCL5/CCL7/CD274/CD40/CD69/CD74/CD86/CDKN1A/CFB/CFH/CIITA/CMKLR1/CMPK2/CSF2RB/CXCL10/CXCL11/CXCL9/DDX60/DHX58/EPSTI1/FAS/FCGR1A/FGL2/FPR1/GBP4/GCH1/GPR18/GZMA/HLA-A/HLA-B/HLA-DMA/HLA-DQA1/HLA-DRB1/ICAM1/IDO1/IFI27/IFI30/IFI35/IFI44/IFIH1/IFIT2/IFIT3/IFITM2/IFITM3/IL10RA/IL15/IL15RA/IL18BP/IL2RB/IL4R/IL6/IL7/IRF1/IRF2/IRF5/IRF7/IRF8/IRF9/ISG20/ITGB7/JAK2/LAP3/LATS2/LCP2/MT2A/MVP/MX1/MX2/NAMPT/NFKB1/NFKBIA/NLRC5/NMI/NOD1/OAS2/OASL/PARP12/PARP14/PDE4B/PIM1/PLSCR1/PML/PSMB10/PSMB8/PSMB9/PTGS2/PTPN2/PTPN6/RNF213/RSAD2/RTP4/SAMHD1/SECTM1/SERPING1/SLAMF7/SOCS1/SOCS3/SOD2/SP110/ST8SIA4/STAT2/TAP1/TAPBP/TNFAIP2/TNFAIP3/TNFSF10/TRIM21/TXNIP/UBE2L6/UPP1/VAMP5/VAMP8/VCAM1/WARS1/XAF1/XCL1/ZBP1/ZNFX1 | 129 | HALLMARK |
| HALLMARK_TNFA_SIGNALING_VIA_NFKB | 5.72E-52 | 7.80E-49 | AREG/ATF3/B4GALT1/BCL2A1/BCL3/BIRC3/BTG2/CCL2/CCL20/CCL4/CCL5/CCN1/CCRL2/CD44/CD69/CD80/CD83/CDKN1A/CEBPB/CEBPD/CFLAR/CLCF1/CSF1/CXCL1/CXCL10/CXCL11/CXCL2/CXCL3/CXCL6/DRAM1/DUSP1/DUSP2/EGR1/EGR2/EIF1/ETS2/FOS/FOSB/FOSL1/FOSL2/FUT4/G0S2/GADD45A/GADD45B/GCH1/GEM/GFPT2/GPR183/HBEGF/ICAM1/ICOSLG/IER2/IER3/IFIH1/IFIT2/IFNGR2/IL15RA/IL18/IL1A/IL1B/IL23A/IL6/IL7R/INHBA/IRF1/JUN/JUNB/KLF10/KLF2/KLF4/KYNU/LIF/LITAF/MAP2K3/MAP3K8/MSC/MXD1/NAMPT/NFKB1/NFKB2/NFKBIA/NFKBIE/NINJ1/NR4A2/NR4A3/OLR1/PDE4B/PFKFB3/PLAU/PLAUR/PLEK/PLPP3/PNRC1/PPP1R15A/PTGER4/PTGS2/PTX3/REL/RELB/RNF19B/SAT1/SERPINE1/SGK1/SLC16A6/SLC2A3/SNN/SOCS3/SOD2/SPHK1/SQSTM1/STAT5A/TAP1/TIPARP/TLR2/TNF/TNFAIP2/TNFAIP3/TNFAIP8/TNIP1/TRAF1/TRIB1/TSC22D1/ZC3H12A/ZFP36 | 124 | HALLMARK |
| REACTOME_CELL_CYCLE_CHECKPOINTS | 1.53E-49 | 6.55E-47 | ATRIP/AURKB/BARD1/BIRC5/BRCA1/BRIP1/BUB1/BUB1B/BUB3/CCNA2/CCNB1/CCNB2/CCNE1/CCNE2/CDC20/CDC23/CDC25A/CDC25C/CDC45/CDC6/CDC7/CDCA8/CDK1/CDK2/CENPA/CENPE/CENPF/CENPH/CENPI/CENPK/CENPL/CENPM/CENPN/CENPO/CENPP/CENPQ/CENPU/CHEK1/CHEK2/CLSPN/COP1/DBF4/DNA2/DSN1/ERCC6L/EXO1/GTSE1/H2AX/H2BC17/H2BC9/INCENP/KIF18A/KIF2C/KNL1/KNTC1/MAD1L1/MAD2L1/MCM10/MCM2/MCM3/MCM4/MCM5/MCM6/MCM7/MCM8/MIS12/MRE11/NDC80/NDE1/NSD2/NSL1/NUF2/NUP107/NUP160/NUP43/NUP85/ORC1/ORC6/PIAS4/PKMYT1/PLK1/PMF1/PPP1CC/PSMB2/PSMB7/PSMD11/PSMD14/PSMD3/PSMD5/PSMD6/PSME3/RBBP8/RCC2/RFC2/RFC3/RFC4/RFC5/RHNO1/RMI1/RMI2/RNF168/RPA1/RPA2/SGO1/SGO2/SKA1/SKA2/SPC24/SPC25/SPDL1/TOP3A/TOPBP1/UBE2C/UBE2S/UIMC1/WRN/YWHAQ/ZNF385A/ZW10/ZWILCH/ZWINT | 121 | REACTOME |
| HALLMARK_ALLOGRAFT_REJECTION | 3.48E-47 | 3.17E-44 | AARS1/B2M/BCL3/C2/CAPG/CCL2/CCL4/CCL5/CCL7/CCND2/CCR1/CCR2/CCR5/CD1D/CD2/CD247/CD28/CD3D/CD3E/CD3G/CD4/CD40/CD40LG/CD7/CD74/CD79A/CD80/CD86/CD8A/CD8B/CD96/CRTAM/CSF1/CSK/CTSS/CXCL13/CXCL9/CXCR3/ELF4/FAS/FASLG/FCGR2B/FGR/FYB1/GBP2/GCNT1/GPR65/GZMA/GZMB/HCLS1/HLA-A/HLA-DMA/HLA-DMB/HLA-DOA/HLA-DOB/HLA-DQA1/HLA-DRA/HLA-E/ICAM1/ICOSLG/IFNGR1/IFNGR2/IGSF6/IL10/IL11/IL12RB1/IL15/IL16/IL18/IL18RAP/IL1B/IL2RA/IL2RB/IL2RG/IL4R/IL6/IL7/INHBA/IRF7/IRF8/ITGAL/ITGB2/ITK/JAK2/LCK/LCP2/LIF/LTB/LY75/LY86/LYN/MAP4K1/NCF4/NLRP3/PRF1/PSMB10/PTPN6/PTPRC/SIT1/SOCS1/SPI1/SRGN/ST8SIA4/STAB1/TAP1/TAP2/TAPBP/TGFB1/TIMP1/TLR1/TLR2/TLR3/TLR6/TNF/TRAT1/UBE2N/WARS1/WAS/ZAP70 | 119 | HALLMARK |
| HALLMARK_INFLAMMATORY_RESPONSE | 2.96E-46 | 2.02E-43 | ADGRE1/AQP9/AXL/BST2/BTG2/C3AR1/C5AR1/CCL2/CCL20/CCL5/CCL7/CCR7/CCRL2/CD14/CD40/CD48/CD55/CD69/CD70/CDKN1A/CHST2/CLEC5A/CMKLR1/CSF1/CSF3/CSF3R/CXCL10/CXCL11/CXCL6/CXCL8/CXCL9/CXCR6/CYBB/DCBLD2/EBI3/EMP3/FFAR2/FPR1/GCH1/GNA15/GPR132/GPR183/HBEGF/HRH1/ICAM1/ICAM4/ICOSLG/IFITM1/IFNGR2/IL10/IL10RA/IL15/IL15RA/IL18/IL18R1/IL18RAP/IL1A/IL1B/IL1R1/IL2RB/IL4R/IL6/IL7R/INHBA/IRAK2/IRF1/IRF7/KCNMB2/KIF1B/LAMP3/LCK/LCP2/LIF/LTA/LYN/MARCO/MEFV/MET/MSR1/MXD1/NAMPT/NFKB1/NFKBIA/NLRP3/NMI/NOD2/OLR1/OSM/P2RX4/PDE4B/PDPN/PIK3R5/PLAUR/PROK2/PTAFR/PTGER2/PTGER4/RGS1/RGS16/RHOG/RNF144B/RTP4/SCARF1/SELE/SELL/SERPINE1/SGMS2/SLAMF1/SPHK1/STAB1/TAPBP/TIMP1/TLR1/TLR2/TLR3/TNFRSF1B/TNFSF10/TNFSF15 | 118 | HALLMARK |
| REACTOME_M_PHASE | 5.84E-44 | 2.09E-41 | ANKLE2/AURKB/BIRC5/BUB1/BUB1B/BUB3/CCNB1/CCNB2/CDC20/CDC23/CDCA5/CDCA8/CDK1/CENPA/CENPE/CENPF/CENPH/CENPI/CENPJ/CENPK/CENPL/CENPM/CENPN/CENPO/CENPP/CENPQ/CENPU/CEP131/CEP41/CEP72/CEP76/CHMP6/CHMP7/DCTN3/DSN1/ERCC6L/ESPL1/FBXO5/GORASP2/H2AX/H2AZ1/H2BC17/H2BC9/H3-3A/H3C2/H3C3/HAUS1/HAUS2/HAUS3/HAUS6/HAUS8/HDAC8/INCENP/IST1/KIF18A/KIF20A/KIF23/KIF2C/KMT5A/KNL1/KNTC1/KPNB1/LBR/LMNB1/LPIN3/MAD1L1/MAD2L1/MASTL/MCPH1/MIS12/NCAPD2/NCAPD3/NCAPG/NCAPG2/NCAPH/NCAPH2/NDC1/NDC80/NDE1/NEDD1/NEK2/NSL1/NUF2/NUP107/NUP155/NUP160/NUP188/NUP205/NUP210/NUP35/NUP42/NUP43/NUP50/NUP85/NUP88/ODF2/PLK1/PLK4/PMF1/PPP1CC/PPP2R2A/PSMB2/PSMB7/PSMD11/PSMD14/PSMD3/PSMD5/PSMD6/PSME3/PTTG1/RAE1/RAN/RCC1/RCC2/SET/SGO1/SGO2/SKA1/SKA2/SMC1A/SMC2/SMC3/SMC4/SPC24/SPC25/SPDL1/TMPO/TUBA1A/TUBA1B/TUBB/TUBG1/TUBGCP3/UBE2C/UBE2I/UBE2S/VRK1/ZW10/ZWILCH/ZWINT | 139 | REACTOME |
| REACTOME_NEUTROPHIL_DEGRANULATION | 1.59E-43 | 8.65E-41 | ABCA13/ACP3/ADA2/ADAM8/ADGRE3/ADGRG3/AGPAT2/ALDH3B1/ALOX5/AMPD3/ANPEP/ANXA2/ARHGAP45/ARHGAP9/ARMC8/ARPC5/ARSA/ASAH1/ATG7/ATP6AP2/ATP8B4/B2M/B4GALT1/BIN2/BRI3/BST1/BST2/C3/C3AR1/C5AR1/CAMP/CAT/CD14/CD300A/CD33/CD36/CD44/CD53/CD55/CD68/CDA/CEACAM1/CFD/CHI3L1/CLEC12A/CLEC5A/CNN2/CR1/CRACR2A/CREG1/CRISPLD2/CSTB/CTSA/CTSB/CTSC/CTSD/CTSH/CTSS/CTSZ/CXCL1/CXCR1/CXCR2/CYBA/CYBB/CYFIP1/DEFA1/DNASE1L1/DOCK2/DOK3/FCAR/FCER1G/FCGR2A/FCGR3B/FCN1/FGL2/FGR/FPR1/FPR2/FTH1/FTL/FUCA1/FUCA2/GAA/GCA/GLB1/GLIPR1/GM2A/GMFG/GNS/GPR84/GRN/GSDMD/GSN/HEXB/HK3/HLA-A/HLA-B/HLA-C/HP/HPSE/HSPA1A/HSPA1B/HSPA6/IMPDH1/IQGAP1/IRAG2/ITGAL/ITGAM/ITGAX/ITGB2/LAIR1/LAMP1/LAMTOR1/LGALS3/LILRB2/LILRB3/LRG1/LTA4H/LTF/LYZ/MAN2B1/MANBA/MCEMP1/MGAM/MGST1/MME/MNDA/MVP/NBEAL2/NCKAP1L/NEU1/NFAM1/NFKB1/NPC2/OLR1/OSCAR/OSTF1/P2RX1/PAFAH1B2/PFKL/PKM/PLAC8/PLAU/PLAUR/PLEKHO2/PPBP/PRKCD/PSAP/PSMA5/PTAFR/PTPN6/PTPRC/PTX3/PYCARD/RAB27A/RAB5B/RAP1A/RAP2B/RETN/RHOG/RNASE2/RNASE3/RNASET2/S100A11/S100A12/S100A8/S100A9/S100P/SELL/SERPINA1/SERPINA3/SERPINB1/SIGLEC5/SIGLEC9/SIRPB1/SLC11A1/SLC2A3/SLC2A5/SLPI/SNAP23/SPTAN1/STING1/STOM/TBC1D10C/TCIRG1/TICAM2/TLR2/TMBIM1/TMC6/TNFRSF1B/TOM1/TRPM2/TYROBP/UNC13D/VAMP8/VNN1 | 196 | REACTOME |
| WP_RETINOBLASTOMA_GENE_IN_CANCER | 2.55E-41 | 7.81E-39 | ABL1/BARD1/CCNA2/CCNB1/CCNB2/CCNE1/CCNE2/CDC25A/CDC25B/CDC45/CDC7/CDK1/CDK2/CDK4/CDK6/CDT1/CHEK1/DHFR/DNMT1/E2F1/E2F2/E2F3/FAF1/FANCG/H2AZ1/HMGB1/HMGB2/KIF4A/MCM3/MCM4/MCM6/MCM7/MSH6/ORC1/PCNA/PLK4/POLA1/POLD3/POLE/POLE2/PRIM1/RAF1/RBBP4/RBBP7/RFC3/RFC4/RFC5/RPA1/RPA2/RRM1/RRM2/SKP2/SMC1A/SMC2/SMC3/SUV39H1/TFDP1/TOP2A/TTK/TYMS | 60 | WP |
| REACTOME_CYTOKINE_SIGNALING_IN_IMMUNE_SYSTEM | 1.17E-39 | 5.30E-37 | AKT3/ALOX5/ANXA1/ANXA2/B2M/BATF/BIRC3/BLNK/BST2/CAPZA1/CASP1/CCL2/CCL20/CCL3/CCL3L3/CCL4/CCL5/CCR1/CCR2/CCR5/CD27/CD36/CD4/CD40/CD40LG/CD44/CD70/CD80/CD86/CDKN1A/CDKN1B/CEBPD/CIITA/CISH/CLCF1/CNN2/CNTFR/CRKL/CSF1/CSF1R/CSF2RA/CSF2RB/CSF3/CSF3R/CSK/CXCL1/CXCL10/CXCL2/CXCL8/EBI3/EGR1/F13A1/FASLG/FBXW11/FCGR1A/FCGR1B/FLNB/FOS/FOXO3/FPR1/FYN/GBP1/GBP2/GBP3/GBP4/GBP5/GRAP2/GRB2/GSDMD/HAVCR2/HCK/HGF/HLA-A/HLA-B/HLA-C/HLA-DPA1/HLA-DPB1/HLA-DQA1/HLA-DQA2/HLA-DQB1/HLA-DRA/HLA-DRB1/HLA-E/HLA-F/HMOX1/ICAM1/IFI27/IFI30/IFI35/IFIT2/IFIT3/IFITM1/IFITM2/IFITM3/IFNGR1/IFNGR2/IFNLR1/IGHG1/IGHG4/IL10/IL10RA/IL10RB/IL11/IL12RB1/IL13RA1/IL15/IL15RA/IL16/IL17RA/IL17RB/IL18/IL18BP/IL18R1/IL18RAP/IL1A/IL1B/IL1R1/IL1R2/IL1RN/IL21R/IL23A/IL2RA/IL2RB/IL2RG/IL32/IL3RA/IL4R/IL6/IL6R/IL7/IL7R/INPP5D/IRAK2/IRAK3/IRF1/IRF2/IRF5/IRF7/IRF8/IRF9/ISG20/ITGAM/ITGAX/ITGB2/JAK2/JAK3/JUN/JUNB/KPNA3/KPNA5/LCK/LCP1/LGALS9/LIF/LTA/LTB/LTBR/LYN/MAP2K3/MAP2K6/MAP3K8/MAPKAPK3/MMP1/MT2A/MUC1/MX1/MX2/NFKB1/NFKB2/NFKBIA/NOD1/NOD2/OAS1/OAS2/OASL/OSM/PELI2/PIK3CD/PIM1/PML/PPM1B/PPP2R1A/PRKCD/PSMA1/PSMA5/PSMB10/PSMB8/PSMB9/PTAFR/PTGS2/PTPN11/PTPN18/PTPN2/PTPN6/PTPN7/RELB/RPS6KA1/RSAD2/S100A12/SAA1/SAMHD1/SLA/SLA2/SOCS1/SOCS3/SOD2/SP100/SQSTM1/STAT2/STAT5A/STAT6/STX3/STX4/STXBP2/SYK/TEC/TGFB1/TIMP1/TNF/TNFRSF11A/TNFRSF11B/TNFRSF12A/TNFRSF14/TNFRSF1A/TNFRSF1B/TNFRSF4/TNFRSF6B/TNFRSF8/TNFSF13/TNFSF13B/TNFSF14/TNFSF15/TNFSF4/TNFSF8/TRIM21/TRIM22/TRIM34/TRIM38/TSLP/TWIST1/UBA7/UBE2D3/UBE2L6/UBE2N/VAV1/VCAM1/VIM/XAF1 | 248 | REACTOME |
| REACTOME_MITOTIC_METAPHASE_AND_ANAPHASE | 5.04E-36 | 1.35E-33 | ANKLE2/AURKB/BIRC5/BUB1/BUB1B/BUB3/CCNB1/CCNB2/CDC20/CDC23/CDCA5/CDCA8/CDK1/CENPA/CENPE/CENPF/CENPH/CENPI/CENPK/CENPL/CENPM/CENPN/CENPO/CENPP/CENPQ/CENPU/CHMP6/CHMP7/DSN1/ERCC6L/ESPL1/FBXO5/HDAC8/INCENP/IST1/KIF18A/KIF2C/KNL1/KNTC1/KPNB1/LBR/LMNB1/MAD1L1/MAD2L1/MIS12/NDC1/NDC80/NDE1/NSL1/NUF2/NUP107/NUP155/NUP160/NUP188/NUP205/NUP35/NUP43/NUP85/PLK1/PMF1/PPP1CC/PPP2R2A/PSMB2/PSMB7/PSMD11/PSMD14/PSMD3/PSMD5/PSMD6/PSME3/PTTG1/RAN/RCC1/RCC2/SGO1/SGO2/SKA1/SKA2/SMC1A/SMC3/SPC24/SPC25/SPDL1/TMPO/TUBA1A/TUBA1B/UBE2C/UBE2I/UBE2S/VRK1/ZW10/ZWILCH/ZWINT | 93 | REACTOME |
| REACTOME_NEURONAL_SYSTEM | 2.64E-36 | 7.11E-33 | ABCC8/ADCY1/ADCY2/ADCY5/AKAP5/ALDH2/AP2A1/AP2A2/AP2M1/APBA1/APBA3/ARHGEF9/BEGAIN/CACNA1A/CACNA1E/CACNA2D2/CACNA2D3/CACNB1/CACNB2/CACNB3/CACNB4/CACNG3/CACNG8/CALM1/CAMK2A/CAMK2B/CAMK2G/CAMK4/CAMKK1/CAMKK2/CHRNA7/CHRNB2/CPLX1/DBNL/DLG2/DLG3/DLG4/DLGAP1/DLGAP3/DNAJC5/EPB41L1/EPB41L3/ERBB4/GABBR1/GABBR2/GABRA1/GABRA2/GABRA4/GABRA5/GABRB1/GABRB2/GABRB3/GAD2/GIT1/GLRA2/GLRB/GLS/GLS2/GNAI1/GNAI2/GNAI3/GNAL/GNB1/GNB2/GNB5/GNG12/GNG3/GNG5/GRIN1/GRIN2A/GRIN2B/GRIN2C/GRIN3A/GRIP1/GRIP2/GRM1/GRM5/HCN4/HOMER1/IL1RAP/IL1RAPL1/KCNA1/KCNA2/KCNA3/KCNA5/KCNAB1/KCNAB2/KCNB1/KCNC1/KCNC3/KCNC4/KCNH1/KCNH3/KCNH4/KCNJ11/KCNJ12/KCNJ3/KCNJ4/KCNJ6/KCNJ9/KCNK1/KCNK10/KCNK3/KCNK4/KCNK9/KCNMA1/KCNN1/KCNQ2/KCNQ3/KCNQ5/KCNS1/KCNS3/KIF17/LIN7B/LRFN1/LRFN2/LRRC7/LRRTM4/MAOA/MAPK1/MAPK3/NBEA/NEFL/NPTN/NRGN/NRXN1/NRXN3/NSF/PANX2/PDPK1/PLCB1/PLCB3/PPFIA2/PPFIA3/PPFIA4/PRKAB2/PRKACB/PRKAG2/PRKAR1A/PRKAR1B/PRKAR2B/PRKCB/PRKCG/RAB3A/RAC1/RASGRF2/RIMS1/RTN3/SHANK1/SHANK2/SHARPIN/SLC17A7/SLC1A2/SLC1A3/SLC32A1/SLC6A1/SLC6A12/SLC6A13/SLITRK1/SLITRK4/SLITRK5/SNAP25/STX1A/STXBP1/SYN1/SYN2/SYN3/SYT1/SYT12/SYT7/TSPOAP1/TUBA4A/TUBA8/TUBB2A/VAMP2 | 175 | REACTOME |
| REACTOME_MITOTIC_PROMETAPHASE | 1.74E-34 | 3.74E-32 | AURKB/BIRC5/BUB1/BUB1B/BUB3/CCNB1/CCNB2/CDC20/CDCA5/CDCA8/CDK1/CENPA/CENPE/CENPF/CENPH/CENPI/CENPJ/CENPK/CENPL/CENPM/CENPN/CENPO/CENPP/CENPQ/CENPU/CEP131/CEP41/CEP72/CEP76/DCTN3/DSN1/ERCC6L/HAUS1/HAUS2/HAUS3/HAUS6/HAUS8/HDAC8/INCENP/KIF18A/KIF2C/KNL1/KNTC1/MAD1L1/MAD2L1/MIS12/NCAPD2/NCAPG/NCAPH/NDC80/NDE1/NEDD1/NEK2/NSL1/NUF2/NUP107/NUP160/NUP43/NUP85/ODF2/PLK1/PLK4/PMF1/PPP1CC/RCC2/SGO1/SGO2/SKA1/SKA2/SMC1A/SMC2/SMC3/SMC4/SPC24/SPC25/SPDL1/TUBA1A/TUBA1B/TUBB/TUBG1/TUBGCP3/ZW10/ZWILCH/ZWINT | 84 | REACTOME |
| HALLMARK_MYC_TARGETS_V1 | 1.74E-32 | 3.40E-30 | APEX1/BUB3/CAD/CCNA2/CCT2/CCT3/CCT4/CCT5/CCT7/CDC20/CDC45/CDK2/CDK4/CSTF2/CTPS1/DDX18/DDX21/DEK/EIF2S1/H2AZ1/HDAC2/HDGF/HNRNPA1/HNRNPA2B1/HNRNPA3/HNRNPC/HNRNPD/HNRNPR/HNRNPU/ILF2/KPNA2/KPNB1/MAD2L1/MCM2/MCM4/MCM5/MCM6/MCM7/MRPL9/NME1/NOP56/ODC1/PA2G4/PCNA/PHB/POLE3/PPM1G/PSMB2/PSMD14/PSMD3/PWP1/RAD23B/RAN/RANBP1/RFC4/RNPS1/RRM1/SERBP1/SET/SF3B3/SMARCC1/SNRPA/SNRPA1/SNRPD1/SRM/SRPK1/SRSF1/SRSF2/SRSF3/SRSF7/SSB/SYNCRIP/TARDBP/TFDP1/TRA2B/TRIM28/TYMS/USP1/VBP1/XRCC6/YWHAQ | 81 | HALLMARK |
| REACTOME_SRP_DEPENDENT_COTRANSLATIONAL_PROTEIN_TARGETING_TO_MEMBRANE | 4.11E-33 | 5.54E-30 | DDOST/FAU/RPL10/RPL10A/RPL11/RPL12/RPL13/RPL13A/RPL14/RPL17/RPL18/RPL18A/RPL19/RPL22L1/RPL23/RPL23A/RPL24/RPL26L1/RPL27/RPL27A/RPL28/RPL29/RPL30/RPL32/RPL35/RPL35A/RPL36/RPL36A/RPL39/RPL41/RPL5/RPL6/RPL7/RPL7A/RPL8/RPL9/RPLP0/RPLP1/RPLP2/RPN1/RPN2/RPS10/RPS11/RPS13/RPS14/RPS15/RPS15A/RPS16/RPS17/RPS18/RPS19/RPS2/RPS20/RPS21/RPS25/RPS27L/RPS28/RPS3/RPS3A/RPS4X/RPS5/RPS7/RPS8/RPS9/RPSA/SEC11A/SEC61A1/SEC61A2/SEC61B/SEC61G/SPCS2/SRPRB/SSR1/SSR2/SSR4/UBA52 | 76 | REACTOME |
| KEGG_CYTOKINE_CYTOKINE_RECEPTOR_INTERACTION | 1.69E-32 | 6.59E-30 | BMP7/CCL18/CCL2/CCL20/CCL26/CCL3/CCL3L3/CCL4/CCL4L2/CCL5/CCL7/CCL8/CCR1/CCR2/CCR4/CCR5/CCR6/CCR7/CD27/CD40/CD40LG/CD70/CLCF1/CNTFR/CSF1/CSF1R/CSF2RA/CSF2RB/CSF3/CSF3R/CXCL1/CXCL10/CXCL11/CXCL12/CXCL13/CXCL16/CXCL2/CXCL3/CXCL5/CXCL6/CXCL8/CXCL9/CXCR1/CXCR2/CXCR3/CXCR4/CXCR6/EGF/EPOR/FAS/FASLG/GDF5/HGF/IFNGR1/IFNGR2/IFNLR1/IL10/IL10RA/IL10RB/IL11/IL12RB1/IL13RA1/IL15/IL15RA/IL17RA/IL17RB/IL18/IL18R1/IL18RAP/IL1A/IL1B/IL1R1/IL1R2/IL21R/IL23A/IL2RA/IL2RB/IL2RG/IL3RA/IL4R/IL6/IL6R/IL7/IL7R/INHBA/LIF/LTA/LTB/LTBR/MET/OSM/PLEKHO2/PPBP/RELT/TGFB1/TGFBR1/TGFBR2/TNF/TNFRSF10A/TNFRSF10C/TNFRSF10D/TNFRSF11A/TNFRSF11B/TNFRSF12A/TNFRSF14/TNFRSF1A/TNFRSF1B/TNFRSF4/TNFRSF6B/TNFRSF8/TNFSF10/TNFSF13/TNFSF13B/TNFSF14/TNFSF15/TNFSF4/TNFSF8/TSLP/VEGFC/XCL1/XCL2 | 121 | KEGG |
| REACTOME_EUKARYOTIC_TRANSLATION_ELONGATION | 9.34E-32 | 8.38E-29 | EEF1A1/EEF1A2/EEF1D/EEF1G/FAU/RPL10/RPL10A/RPL11/RPL12/RPL13/RPL13A/RPL14/RPL17/RPL18/RPL18A/RPL19/RPL22L1/RPL23/RPL23A/RPL24/RPL26L1/RPL27/RPL27A/RPL28/RPL29/RPL30/RPL32/RPL35/RPL35A/RPL36/RPL36A/RPL39/RPL41/RPL5/RPL6/RPL7/RPL7A/RPL8/RPL9/RPLP0/RPLP1/RPLP2/RPS10/RPS11/RPS13/RPS14/RPS15/RPS15A/RPS16/RPS17/RPS18/RPS19/RPS2/RPS20/RPS21/RPS25/RPS27L/RPS28/RPS3/RPS3A/RPS4X/RPS5/RPS7/RPS8/RPS9/RPSA/UBA52 | 67 | REACTOME |
| REACTOME_RESOLUTION_OF_SISTER_CHROMATID_COHESION | 5.84E-30 | 1.04E-27 | AURKB/BIRC5/BUB1/BUB1B/BUB3/CCNB1/CCNB2/CDC20/CDCA5/CDCA8/CDK1/CENPA/CENPE/CENPF/CENPH/CENPI/CENPK/CENPL/CENPM/CENPN/CENPO/CENPP/CENPQ/CENPU/DSN1/ERCC6L/HDAC8/INCENP/KIF18A/KIF2C/KNL1/KNTC1/MAD1L1/MAD2L1/MIS12/NDC80/NDE1/NSL1/NUF2/NUP107/NUP160/NUP43/NUP85/PLK1/PMF1/PPP1CC/RCC2/SGO1/SGO2/SKA1/SKA2/SMC1A/SMC3/SPC24/SPC25/SPDL1/TUBA1A/TUBA1B/ZW10/ZWILCH/ZWINT | 61 | REACTOME |
| KEGG_RIBOSOME | 4.11E-30 | 2.77E-27 | FAU/MRPL13/RPL10/RPL10A/RPL11/RPL12/RPL13/RPL13A/RPL14/RPL17/RPL18/RPL18A/RPL19/RPL22L1/RPL23/RPL23A/RPL24/RPL26L1/RPL27/RPL27A/RPL28/RPL29/RPL30/RPL32/RPL35/RPL35A/RPL36/RPL36A/RPL39/RPL41/RPL5/RPL6/RPL7/RPL7A/RPL8/RPL9/RPLP0/RPLP1/RPLP2/RPS10/RPS11/RPS13/RPS15/RPS15A/RPS16/RPS17/RPS18/RPS19/RPS2/RPS20/RPS21/RPS25/RPS27L/RPS28/RPS3/RPS3A/RPS4X/RPS5/RPS7/RPS8/RPS9/RPSA/UBA52 | 63 | KEGG |
| REACTOME_MITOTIC_G1_PHASE_AND_G1_S_TRANSITION | 1.99E-29 | 3.29E-27 | ABL1/CCNA2/CCNB1/CCNE1/CCNE2/CDC25A/CDC45/CDC6/CDC7/CDK1/CDK2/CDK4/CDK6/CDKN2C/CDT1/CKS1B/DBF4/DHFR/E2F1/E2F2/E2F3/E2F5/E2F6/FBXO5/GMNN/LIN9/MCM10/MCM2/MCM3/MCM4/MCM5/MCM6/MCM7/MCM8/MYBL2/ORC1/ORC6/PCNA/POLA1/POLA2/POLE/POLE2/POLE3/PPP2R2A/PPP2R3B/PRIM1/PRIM2/PSMB2/PSMB7/PSMD11/PSMD14/PSMD3/PSMD5/PSMD6/PSME3/RBBP4/RBL1/RPA1/RPA2/RRM2/SKP1/SKP2/TFDP1/TK1/TOP2A/TYMS | 66 | REACTOME |
| REACTOME_EUKARYOTIC_TRANSLATION_INITIATION | 1.63E-29 | 8.80E-27 | EIF2B2/EIF2S3/EIF3B/EIF3D/EIF3E/EIF3G/EIF3I/EIF3M/EIF4A1/EIF4A2/EIF4EBP1/FAU/PABPC1/RPL10/RPL10A/RPL11/RPL12/RPL13/RPL13A/RPL14/RPL17/RPL18/RPL18A/RPL19/RPL22L1/RPL23/RPL23A/RPL24/RPL26L1/RPL27/RPL27A/RPL28/RPL29/RPL30/RPL32/RPL35/RPL35A/RPL36/RPL36A/RPL39/RPL41/RPL5/RPL6/RPL7/RPL7A/RPL8/RPL9/RPLP0/RPLP1/RPLP2/RPS10/RPS11/RPS13/RPS14/RPS15/RPS15A/RPS16/RPS17/RPS18/RPS19/RPS2/RPS20/RPS21/RPS25/RPS27L/RPS28/RPS3/RPS3A/RPS4X/RPS5/RPS7/RPS8/RPS9/RPSA/UBA52 | 75 | REACTOME |
| KEGG_CELL_CYCLE | 2.91E-28 | 4.47E-26 | ABL1/BUB1/BUB1B/BUB3/CCNA2/CCNB1/CCNB2/CCNE1/CCNE2/CDC20/CDC23/CDC25A/CDC25B/CDC25C/CDC45/CDC6/CDC7/CDK1/CDK2/CDK4/CDK6/CDKN2C/CHEK1/CHEK2/DBF4/E2F1/E2F2/E2F3/E2F5/ESPL1/FZR1/GADD45G/HDAC2/MAD1L1/MAD2L1/MAD2L2/MCM2/MCM3/MCM4/MCM5/MCM6/MCM7/ORC1/ORC6/PCNA/PKMYT1/PLK1/PTTG1/RBL1/SKP1/SKP2/SMAD2/SMAD4/SMC1A/SMC3/TFDP1/TTK/YWHAQ/ZBTB17 | 59 | KEGG |
| HALLMARK_INTERFERON_ALPHA_RESPONSE | 1.34E-28 | 4.57E-26 | B2M/BATF2/BST2/C1S/CASP1/CASP8/CCRL2/CD74/CMPK2/CSF1/CXCL10/CXCL11/DDX60/DHX58/ELF1/EPSTI1/GBP2/GBP4/HLA-C/IFI27/IFI30/IFI35/IFI44/IFIH1/IFIT2/IFIT3/IFITM1/IFITM2/IFITM3/IL15/IL4R/IL7/IRF1/IRF2/IRF7/IRF9/ISG20/LAMP3/LAP3/LPAR6/MX1/NMI/OAS1/OASL/PARP12/PARP14/PARP9/PLSCR1/PROCR/PSMB8/PSMB9/RSAD2/RTP4/SELL/SP110/STAT2/TAP1/TMEM140/TRIM21/TXNIP/UBA7/UBE2L6/WARS1 | 63 | HALLMARK |
| HALLMARK_IL6_JAK_STAT3_SIGNALING | 2.13E-28 | 6.46E-26 | A2M/CCL7/CCR1/CD14/CD36/CD44/CNTFR/CSF1/CSF2RA/CSF2RB/CSF3R/CXCL1/CXCL10/CXCL11/CXCL13/CXCL3/CXCL9/EBI3/FAS/GRB2/HMOX1/IFNGR1/IFNGR2/IL10RB/IL12RB1/IL13RA1/IL15RA/IL17RA/IL17RB/IL18R1/IL1B/IL1R1/IL1R2/IL2RA/IL2RG/IL3RA/IL4R/IL6/IL7/IRF1/IRF9/JUN/LTB/LTBR/MAP3K8/PIK3R5/PIM1/PLA2G2A/PTPN11/PTPN2/SOCS1/SOCS3/STAT2/TGFB1/TLR2/TNF/TNFRSF12A/TNFRSF1A/TNFRSF1B | 59 | HALLMARK |
| WP_TYROBP_CAUSAL_NETWORK_IN_MICROGLIA | 2.64E-28 | 7.21E-26 | ADAP2/APBB1IP/BIN2/C1QC/C3/CAPG/CD37/CD4/CD84/CXCL16/DPYD/ELF4/FKBP15/GAPT/GIMAP2/GPX1/HCLS1/HLX/IGSF6/IL10RA/IL13RA1/IL18/ITGAM/ITGAX/ITGB2/LHFPL2/LYL1/MAF/NCF2/NCKAP1L/NPC2/NRROS/PLEK/PYCARD/RBM47/RGS1/RNASE6/RPS6KA1/RUNX3/SAMSN1/SLC1A5/SLC7A7/SPP1/STAT5A/TCIRG1/TGFBR1/TMEM106A/TNFRSF1B/TYROBP | 49 | WP |
| REACTOME_MITOTIC_SPINDLE_CHECKPOINT | 8.82E-28 | 1.26E-25 | AURKB/BIRC5/BUB1/BUB1B/BUB3/CDC20/CDC23/CDCA8/CENPA/CENPE/CENPF/CENPH/CENPI/CENPK/CENPL/CENPM/CENPN/CENPO/CENPP/CENPQ/CENPU/DSN1/ERCC6L/INCENP/KIF18A/KIF2C/KNL1/KNTC1/MAD1L1/MAD2L1/MIS12/NDC80/NDE1/NSL1/NUF2/NUP107/NUP160/NUP43/NUP85/PLK1/PMF1/PPP1CC/RCC2/SGO1/SGO2/SKA1/SKA2/SPC24/SPC25/SPDL1/UBE2C/UBE2S/ZW10/ZWILCH/ZWINT | 55 | REACTOME |
| REACTOME_ADAPTIVE_IMMUNE_SYSTEM | 1.32E-27 | 3.28E-25 | AKT3/AP1B1/AP2B1/ASB3/ASB5/ASB8/ASB9/ATG7/B2M/BCAP31/BLNK/BTK/BTN3A1/BTN3A2/BTN3A3/C3/CAPZA1/CAPZB/CARD11/CD14/CD1C/CD1D/CD200R1/CD209/CD226/CD247/CD274/CD28/CD300A/CD300C/CD300E/CD300LB/CD300LF/CD33/CD36/CD3D/CD3E/CD3G/CD4/CD40/CD40LG/CD74/CD79A/CD79B/CD80/CD81/CD86/CD8A/CD8B/CD96/CD99/CLEC2B/COLEC12/CRTAM/CSK/CTLA4/CTSA/CTSB/CTSC/CTSD/CTSH/CTSK/CTSL/CTSO/CTSS/CUL7/CYBA/CYBB/DAPP1/DNM2/DTX3L/DYNLL2/FBXO21/FBXO32/FBXO6/FBXW11/FCGR1A/FCGR1B/FCGR2B/FCGR3A/FYB1/FYN/GAN/GRAP2/GRB2/HACE1/HCST/HLA-A/HLA-B/HLA-C/HLA-DMA/HLA-DMB/HLA-DOA/HLA-DOB/HLA-DPA1/HLA-DPB1/HLA-DQA1/HLA-DQA2/HLA-DQB1/HLA-DRA/HLA-DRB1/HLA-E/HLA-F/ICAM1/ICAM3/ICAM4/ICOS/ICOSLG/IFI30/IFITM1/IGHM/IGKV1-5/IGKV1D-39/IGKV3-11/IGKV3-15/IGKV3-20/IGKV4-1/IGLC2/IGLC3/IGLV1-40/IGLV1-44/IGLV2-14/IGLV2-8/INPP5D/ITGAL/ITGB2/ITGB7/ITK/JAML/KBTBD6/KBTBD7/KIF2A/KLC4/KLHL21/KLHL22/KLHL42/KLRB1/LAG3/LAIR1/LCK/LCP2/LGMN/LILRA1/LILRA2/LILRA5/LILRA6/LILRB1/LILRB2/LILRB3/LILRB4/LILRB5/LY96/LYN/MAP3K8/MICA/MICB/MRC1/MYLIP/NCF1/NCF2/NCF4/NECTIN2/NFATC2/NFKB1/NFKBIA/NFKBIE/OSCAR/PDCD1/PDCD1LG2/PIK3AP1/PIK3CD/PILRA/PLCG2/PPP2R1A/PPP2R5C/PSMA1/PSMA5/PSMB10/PSMB8/PSMB9/PTPN11/PTPN22/PTPN6/PTPRC/RAP1A/RCHY1/REL/RNF144B/RNF19B/RNF213/RNF217/S100A8/S100A9/SELL/SH2D1A/SIGLEC1/SIGLEC10/SIGLEC11/SIGLEC12/SIGLEC5/SIGLEC7/SIGLEC9/SIPA1/SLAMF6/SLAMF7/SNAP23/SOCS1/SOCS3/STX4/SYK/TAP1/TAP2/TAPBP/THOP1/TLR1/TLR2/TLR4/TLR6/TNFRSF14/TRAT1/TREM1/TREM2/TREML1/TREML2/TRIM21/TRIM9/TUBA1C/TUBB2B/TYROBP/UBA7/UBE2B/UBE2D3/UBE2F/UBE2L6/UBE2N/UBE2Z/UBE3D/VAMP3/VAMP8/VASP/VAV1/VCAM1/WAS/ZAP70/ZNRF2 | 245 | REACTOME |
| REACTOME_TRANSMISSION_ACROSS_CHEMICAL_SYNAPSES | 8.56E-28 | 3.84E-25 | ADCY1/ADCY2/ADCY5/AKAP5/ALDH2/AP2A1/AP2A2/AP2M1/APBA1/ARHGEF9/CACNA1A/CACNA1E/CACNA2D2/CACNA2D3/CACNB1/CACNB2/CACNB3/CACNB4/CACNG3/CACNG8/CALM1/CAMK2A/CAMK2B/CAMK2G/CAMK4/CAMKK1/CAMKK2/CHRNA7/CHRNB2/CPLX1/DLG2/DLG3/DLG4/DNAJC5/EPB41L1/ERBB4/GABBR1/GABBR2/GABRA1/GABRA2/GABRA4/GABRA5/GABRB1/GABRB2/GABRB3/GAD2/GIT1/GLRA2/GLRB/GLS/GLS2/GNAI1/GNAI2/GNAI3/GNAL/GNB1/GNB2/GNB5/GNG12/GNG3/GNG5/GRIN1/GRIN2A/GRIN2B/GRIN2C/GRIN3A/GRIP1/GRIP2/KCNJ12/KCNJ3/KCNJ4/KCNJ6/KCNJ9/KIF17/LIN7B/LRRC7/MAOA/MAPK1/MAPK3/NBEA/NEFL/NPTN/NRGN/NSF/PDPK1/PLCB1/PLCB3/PPFIA2/PPFIA3/PPFIA4/PRKAB2/PRKACB/PRKAG2/PRKAR1A/PRKAR1B/PRKAR2B/PRKCB/PRKCG/RAB3A/RAC1/RASGRF2/RIMS1/SLC17A7/SLC1A2/SLC1A3/SLC32A1/SLC6A1/SLC6A12/SLC6A13/SNAP25/STX1A/STXBP1/SYN1/SYN2/SYN3/SYT1/TSPOAP1/TUBA4A/TUBA8/TUBB2A/VAMP2 | 121 | REACTOME |
| WP_CELL_CYCLE | 4.69E-27 | 5.60E-25 | ABL1/BUB1/BUB3/CCNA2/CCNB1/CCNB2/CCNE1/CCNE2/CDC20/CDC23/CDC25A/CDC25B/CDC25C/CDC45/CDC6/CDC7/CDK1/CDK2/CDK4/CDK6/CDKN2C/CHEK1/CHEK2/DBF4/E2F1/E2F2/E2F3/E2F5/ESPL1/FZR1/GADD45G/HDAC2/MAD1L1/MAD2L2/MCM2/MCM3/MCM4/MCM5/MCM6/MCM7/ORC1/ORC6/PCNA/PKMYT1/PLK1/PTTG1/RBL1/SKP1/SKP2/SMAD2/SMAD4/SMC1A/SMC3/TFDP1/TTK/YWHAQ/ZBTB17 | 57 | WP |
| REACTOME_RESPONSE_OF_EIF2AK4_GCN2_TO_AMINO_ACID_DEFICIENCY | 2.05E-27 | 7.89E-25 | ATF4/CEBPG/EIF2S3/FAU/RPL10/RPL10A/RPL11/RPL12/RPL13/RPL13A/RPL14/RPL17/RPL18/RPL18A/RPL19/RPL22L1/RPL23/RPL23A/RPL24/RPL26L1/RPL27/RPL27A/RPL28/RPL29/RPL30/RPL32/RPL35/RPL35A/RPL36/RPL36A/RPL39/RPL41/RPL5/RPL6/RPL7/RPL7A/RPL8/RPL9/RPLP0/RPLP1/RPLP2/RPS10/RPS11/RPS13/RPS14/RPS15/RPS15A/RPS16/RPS17/RPS18/RPS19/RPS2/RPS20/RPS21/RPS25/RPS27L/RPS28/RPS3/RPS3A/RPS4X/RPS5/RPS7/RPS8/RPS9/RPSA/UBA52 | 66 | REACTOME |
| REACTOME_S_PHASE | 7.73E-27 | 8.73E-25 | CCNA2/CCNE1/CCNE2/CDC23/CDC25A/CDC25B/CDC45/CDC6/CDCA5/CDK2/CDK4/CDT1/CKS1B/DNA2/E2F1/E2F5/ESCO1/ESCO2/FEN1/FZR1/GINS1/GINS2/GINS3/GINS4/LIG1/LIN9/MCM2/MCM3/MCM4/MCM5/MCM6/MCM7/MCM8/ORC1/ORC6/PCNA/POLA1/POLA2/POLD3/POLE/POLE2/POLE3/PRIM1/PRIM2/PSMB2/PSMB7/PSMD11/PSMD14/PSMD3/PSMD5/PSMD6/PSME3/RBBP4/RFC2/RFC3/RFC4/RFC5/RPA1/RPA2/SKP1/SKP2/SMC1A/SMC3/TFDP1/UBE2C/UBE2S | 66 | REACTOME |
| WP_CYTOPLASMIC_RIBOSOMAL_PROTEINS | 4.19E-27 | 1.41E-24 | FAU/RPL10/RPL10A/RPL11/RPL12/RPL13/RPL13A/RPL14/RPL17/RPL18/RPL18A/RPL19/RPL23/RPL23A/RPL24/RPL27/RPL27A/RPL28/RPL29/RPL30/RPL32/RPL35/RPL35A/RPL36/RPL36A/RPL39/RPL41/RPL5/RPL6/RPL7/RPL7A/RPL8/RPL9/RPLP0/RPLP1/RPLP2/RPS10/RPS11/RPS13/RPS14/RPS15/RPS15A/RPS16/RPS17/RPS18/RPS19/RPS2/RPS20/RPS21/RPS25/RPS28/RPS3/RPS3A/RPS4X/RPS5/RPS6KB2/RPS7/RPS8/RPS9/RPSA/UBA52 | 61 | WP |
| WP_NETWORK_MAP_OF_SARSCOV2_SIGNALING_PATHWAY | 1.21E-26 | 2.74E-24 | ACTB/APOC1/APOL1/BST2/BTN3A1/C1R/C1S/CARD11/CASP5/CASP8/CCL2/CCL20/CCL26/CCL3/CCL4/CCL5/CCL8/CCR5/CCR6/CD14/CD163/CD2/CD226/CD247/CD3E/CD3G/CD4/CD8A/CD8B/CEBPB/CFB/CFH/CFI/COL7A1/CTSB/CTSD/CTSL/CTSZ/CXCL1/CXCL10/CXCL12/CXCL13/CXCL16/CXCL2/CXCL3/CXCL5/CXCL6/CXCL8/CXCL9/CXCR1/CXCR2/DDIT4/DUSP1/EGR1/FOS/FYN/GSN/HLA-DRA/HLA-DRB1/HP/IFI27/IFIH1/IFITM1/IFITM3/IL10/IL16/IL18/IL18RAP/IL1A/IL1B/IL1R2/IL6/IL7/IRF9/JAK2/JUN/JUNB/LCK/LRG1/MX1/NFKB2/NLRP1/NLRP3/OAS2/PARP2/PTGS2/PTPN6/RRAS/SAA1/SAA2/SERPINA5/SERPINE1/SKAP1/STEAP3/TGFBR2/TNF/TNFSF10/TRPM2/VPS18/ZAP70 | 100 | WP |
| REACTOME_DNA_DOUBLE_STRAND_BREAK_REPAIR | 6.27E-26 | 6.73E-24 | ABL1/ATRIP/BARD1/BRCA1/BRCA2/BRIP1/CCNA2/CDK2/CHEK1/CHEK2/CLSPN/DNA2/EME1/EXO1/FEN1/GEN1/H2AX/H2BC17/H2BC9/KDM4B/KPNA2/MRE11/MUS81/NHEJ1/NSD2/PARP1/PAXIP1/PCNA/PIAS4/POLD3/POLE/POLE2/POLE3/POLH/POLQ/PPP4R2/RAD51/RAD51AP1/RAD51D/RBBP8/RFC2/RFC3/RFC4/RFC5/RHNO1/RMI1/RMI2/RNF168/RNF4/RPA1/RPA2/RTEL1/SUMO2/TDP1/TIMELESS/TIPIN/TOP3A/TOPBP1/UBE2I/UIMC1/WRN/XRCC1/XRCC2/XRCC4/XRCC5/XRCC6 | 66 | REACTOME |
| REACTOME_SEPARATION_OF_SISTER_CHROMATIDS | 6.87E-26 | 7.02E-24 | AURKB/BIRC5/BUB1/BUB1B/BUB3/CDC20/CDC23/CDCA5/CDCA8/CENPA/CENPE/CENPF/CENPH/CENPI/CENPK/CENPL/CENPM/CENPN/CENPO/CENPP/CENPQ/CENPU/DSN1/ERCC6L/ESPL1/HDAC8/INCENP/KIF18A/KIF2C/KNL1/KNTC1/MAD1L1/MAD2L1/MIS12/NDC80/NDE1/NSL1/NUF2/NUP107/NUP160/NUP43/NUP85/PLK1/PMF1/PPP1CC/PSMB2/PSMB7/PSMD11/PSMD14/PSMD3/PSMD5/PSMD6/PSME3/PTTG1/RCC2/SGO1/SGO2/SKA1/SKA2/SMC1A/SMC3/SPC24/SPC25/SPDL1/TUBA1A/TUBA1B/UBE2C/UBE2S/ZW10/ZWILCH/ZWINT | 71 | REACTOME |
| REACTOME_NERVOUS_SYSTEM_DEVELOPMENT | 8.09E-25 | 2.42E-22 | ABLIM1/ABLIM2/ACTG1/ADGRV1/AGAP2/AKAP5/ANK1/ANK2/ANK3/AP2A1/AP2A2/AP2M1/APH1A/ARHGEF28/ARPC1A/CACNA1C/CACNA1D/CACNA1G/CACNA1I/CACNB1/CACNB2/CACNB3/CACNB4/CAP2/CDK5R1/CLTB/CNTN6/CNTNAP1/CSNK2B/DLG3/DLG4/DNM1/DNM3/DOK6/DRP2/EFNA1/EFNA3/EFNA5/EFNB3/EPHA10/EPHA4/EPHA5/EPHA7/EPHB6/EVL/FAU/GFRA2/GIT1/GRIN1/GRIN2B/HSP90AB1/ITGA9/ITSN1/KALRN/KCNQ2/KCNQ3/L1CAM/LDB1/LYPLA2/MAGOH/MAP2K1/MAPK1/MAPK3/MAPK7/MYH11/MYL6/MYO9B/NCK1/NCSTN/NELL2/NFASC/NGEF/NTN4/PABPC1/PAK1/PAK3/PAK4/PAK5/PAK6/PDLIM7/PFN1/PFN2/PIK3CB/PIK3R1/PIP5K1C/PITPNA/PLXNA3/PPP3CB/PRKACB/PRNP/PSENEN/PSMA2/PSMA3/PSMA4/PSMA7/PSMB1/PSMB3/PSMB4/PSMC2/PSMC4/PSMD13/PSMD9/PSME1/PSME2/PSMF1/RAC1/RAP1GAP/RELN/RHOA/RHOC/RND1/RPL10/RPL10A/RPL11/RPL12/RPL13/RPL13A/RPL14/RPL17/RPL18/RPL18A/RPL19/RPL22L1/RPL23/RPL23A/RPL24/RPL26L1/RPL27/RPL27A/RPL28/RPL29/RPL30/RPL32/RPL35/RPL35A/RPL36/RPL36A/RPL39/RPL41/RPL5/RPL6/RPL7/RPL7A/RPL8/RPL9/RPLP0/RPLP1/RPLP2/RPS10/RPS11/RPS13/RPS14/RPS15/RPS15A/RPS16/RPS17/RPS18/RPS19/RPS2/RPS20/RPS21/RPS25/RPS27L/RPS28/RPS3/RPS3A/RPS4X/RPS5/RPS6KA5/RPS7/RPS8/RPS9/RPSA/SCN2A/SCN2B/SCN3B/SCN4B/SCN8A/SEM1/SEMA4A/SH3GL2/SLIT2/SLIT3/SPTB/SPTBN1/SPTBN2/SPTBN4/SREBF2/TIAM1/TRPC1/TUBA4A/TUBA8/TUBB2A/UBA52/UNC5A/UNC5D | 196 | REACTOME |
| REACTOME_CELLULAR_RESPONSE_TO_STARVATION | 1.14E-24 | 3.07E-22 | ATF4/ATP6V0C/ATP6V0D1/ATP6V0E1/ATP6V1A/ATP6V1B2/ATP6V1C1/ATP6V1D/ATP6V1E1/ATP6V1G2/ATP6V1H/CEBPG/DEPDC5/EIF2S3/FAU/FNIP2/LAMTOR2/LAMTOR5/RPL10/RPL10A/RPL11/RPL12/RPL13/RPL13A/RPL14/RPL17/RPL18/RPL18A/RPL19/RPL22L1/RPL23/RPL23A/RPL24/RPL26L1/RPL27/RPL27A/RPL28/RPL29/RPL30/RPL32/RPL35/RPL35A/RPL36/RPL36A/RPL39/RPL41/RPL5/RPL6/RPL7/RPL7A/RPL8/RPL9/RPLP0/RPLP1/RPLP2/RPS10/RPS11/RPS13/RPS14/RPS15/RPS15A/RPS16/RPS17/RPS18/RPS19/RPS2/RPS20/RPS21/RPS25/RPS27L/RPS28/RPS3/RPS3A/RPS4X/RPS5/RPS7/RPS8/RPS9/RPSA/SEC13/SESN1/UBA52 | 82 | REACTOME |
| REACTOME_RHO_GTPASES_ACTIVATE_FORMINS | 3.57E-24 | 3.49E-22 | AURKB/BIRC5/BUB1/BUB1B/BUB3/CDC20/CDCA8/CENPA/CENPE/CENPF/CENPH/CENPI/CENPK/CENPL/CENPM/CENPN/CENPO/CENPP/CENPQ/CENPU/DIAPH1/DIAPH3/DSN1/DVL2/DVL3/ERCC6L/INCENP/KIF18A/KIF2C/KNL1/KNTC1/MAD1L1/MAD2L1/MIS12/NDC80/NDE1/NSL1/NUF2/NUP107/NUP160/NUP43/NUP85/PLK1/PMF1/PPP1CC/RCC2/SGO1/SGO2/SKA1/SKA2/SPC24/SPC25/SPDL1/TUBA1A/TUBA1B/ZW10/ZWILCH/ZWINT | 58 | REACTOME |
| REACTOME_IMMUNOREGULATORY_INTERACTIONS_BETWEEN_A_LYMPHOID_AND_A_NON_LYMPHOID_CELL | 2.89E-24 | 6.06E-22 | B2M/C3/CD1C/CD1D/CD200R1/CD226/CD247/CD300A/CD300C/CD300E/CD300LB/CD300LF/CD33/CD3D/CD3E/CD3G/CD40/CD40LG/CD81/CD8A/CD8B/CD96/CD99/CLEC2B/COLEC12/CRTAM/FCGR1A/FCGR2B/FCGR3A/HCST/HLA-A/HLA-B/HLA-C/HLA-E/HLA-F/ICAM1/ICAM3/ICAM4/IFITM1/IGKV1-5/IGKV1D-39/IGKV3-11/IGKV3-15/IGKV3-20/IGKV4-1/IGLC2/IGLC3/IGLV1-40/IGLV1-44/IGLV2-14/IGLV2-8/ITGAL/ITGB2/ITGB7/JAML/KLRB1/LAIR1/LILRA1/LILRA2/LILRA5/LILRA6/LILRB1/LILRB2/LILRB3/LILRB4/LILRB5/MICA/MICB/NECTIN2/OSCAR/PILRA/SELL/SH2D1A/SIGLEC1/SIGLEC10/SIGLEC11/SIGLEC12/SIGLEC5/SIGLEC7/SIGLEC9/SLAMF6/SLAMF7/TREM1/TREM2/TREML1/TREML2/TYROBP/VCAM1 | 88 | REACTOME |
| REACTOME_HOMOLOGY_DIRECTED_REPAIR | 1.07E-23 | 9.95E-22 | ABL1/ATRIP/BARD1/BRCA1/BRCA2/BRIP1/CCNA2/CDK2/CHEK1/CLSPN/DNA2/EME1/EXO1/FEN1/GEN1/H2AX/H2BC17/H2BC9/MRE11/MUS81/NSD2/PARP1/PCNA/PIAS4/POLD3/POLE/POLE2/POLE3/POLH/POLQ/PPP4R2/RAD51/RAD51AP1/RAD51D/RBBP8/RFC2/RFC3/RFC4/RFC5/RHNO1/RMI1/RMI2/RNF168/RNF4/RPA1/RPA2/RTEL1/SUMO2/TIMELESS/TIPIN/TOP3A/TOPBP1/UBE2I/UIMC1/WRN/XRCC1/XRCC2 | 57 | REACTOME |
| REACTOME_SIGNALING_BY_ROBO_RECEPTORS | 4.08E-24 | 9.99E-22 | AKAP5/CAP2/EVL/FAU/LDB1/MAGOH/MYO9B/NCK1/NELL2/PABPC1/PAK1/PAK3/PAK4/PAK5/PAK6/PFN1/PFN2/PPP3CB/PRKACB/PSMA2/PSMA3/PSMA4/PSMA7/PSMB1/PSMB3/PSMB4/PSMC2/PSMC4/PSMD13/PSMD9/PSME1/PSME2/PSMF1/RAC1/RHOA/RPL10/RPL10A/RPL11/RPL12/RPL13/RPL13A/RPL14/RPL17/RPL18/RPL18A/RPL19/RPL22L1/RPL23/RPL23A/RPL24/RPL26L1/RPL27/RPL27A/RPL28/RPL29/RPL30/RPL32/RPL35/RPL35A/RPL36/RPL36A/RPL39/RPL41/RPL5/RPL6/RPL7/RPL7A/RPL8/RPL9/RPLP0/RPLP1/RPLP2/RPS10/RPS11/RPS13/RPS14/RPS15/RPS15A/RPS16/RPS17/RPS18/RPS19/RPS2/RPS20/RPS21/RPS25/RPS27L/RPS28/RPS3/RPS3A/RPS4X/RPS5/RPS7/RPS8/RPS9/RPSA/SEM1/SLIT2/SLIT3/UBA52 | 100 | REACTOME |
| WP_DNA_IRDAMAGE_AND_CELLULAR_RESPONSE_VIA_ATR | 1.50E-23 | 1.29E-21 | ATRIP/BARD1/BRCA1/BRCA2/BRIP1/CDC25C/CDC45/CDK1/CDK2/CHEK1/CHEK2/CLSPN/E2F1/EXO1/FANCA/FANCD2/FANCI/FEN1/FOXM1/H2AX/MCM2/MCPH1/MLH1/MRE11/MSH2/PARP1/PCNA/PLK1/RAD51/RBBP8/RFWD3/RMI1/RPA1/RPA2/SMC1A/TDP1/TOP3A/TOPBP1/TRIM28/UIMC1/USP1/WRN/XRCC5 | 43 | WP |
| REACTOME_DNA_STRAND_ELONGATION | 1.56E-23 | 1.29E-21 | CDC45/DNA2/FEN1/GINS1/GINS2/GINS3/GINS4/LIG1/MCM2/MCM3/MCM4/MCM5/MCM6/MCM7/MCM8/PCNA/POLA1/POLA2/POLD3/PRIM1/PRIM2/RFC2/RFC3/RFC4/RFC5/RPA1/RPA2 | 27 | REACTOME |
| REACTOME_SELENOAMINO_ACID_METABOLISM | 6.14E-24 | 1.38E-21 | AHCY/AIMP1/AIMP2/FAU/PSTK/QARS1/RPL10/RPL10A/RPL11/RPL12/RPL13/RPL13A/RPL14/RPL17/RPL18/RPL18A/RPL19/RPL22L1/RPL23/RPL23A/RPL24/RPL26L1/RPL27/RPL27A/RPL28/RPL29/RPL30/RPL32/RPL35/RPL35A/RPL36/RPL36A/RPL39/RPL41/RPL5/RPL6/RPL7/RPL7A/RPL8/RPL9/RPLP0/RPLP1/RPLP2/RPS10/RPS11/RPS13/RPS14/RPS15/RPS15A/RPS16/RPS17/RPS18/RPS19/RPS2/RPS20/RPS21/RPS25/RPS27L/RPS28/RPS3/RPS3A/RPS4X/RPS5/RPS7/RPS8/RPS9/RPSA/UBA52 | 68 | REACTOME |
| REACTOME_SIGNALING_BY_INTERLEUKINS | 8.89E-24 | 1.73E-21 | ALOX5/ANXA1/ANXA2/BATF/BLNK/CAPZA1/CASP1/CCL2/CCL20/CCL3/CCL3L3/CCL4/CCL5/CCR1/CCR2/CCR5/CD36/CD4/CD80/CD86/CDKN1A/CEBPD/CISH/CLCF1/CNN2/CNTFR/CRKL/CSF1/CSF1R/CSF2RA/CSF2RB/CSF3/CSF3R/CXCL1/CXCL10/CXCL2/CXCL8/EBI3/F13A1/FASLG/FBXW11/FOS/FOXO3/FPR1/FYN/GRB2/GSDMD/HAVCR2/HCK/HGF/HMOX1/ICAM1/IFNLR1/IGHG1/IGHG4/IL10/IL10RA/IL10RB/IL11/IL12RB1/IL13RA1/IL15/IL15RA/IL16/IL17RA/IL17RB/IL18/IL18BP/IL18R1/IL18RAP/IL1A/IL1B/IL1R1/IL1R2/IL1RN/IL21R/IL23A/IL2RA/IL2RB/IL2RG/IL32/IL3RA/IL4R/IL6/IL6R/IL7/IL7R/INPP5D/IRAK2/IRAK3/ITGAM/ITGAX/ITGB2/JAK2/JAK3/JUN/JUNB/LCK/LCP1/LGALS9/LIF/LYN/MAP2K3/MAP2K6/MAP3K8/MAPKAPK3/MMP1/MUC1/NFKB1/NFKB2/NFKBIA/NOD1/NOD2/OSM/PELI2/PIK3CD/PIM1/PPP2R1A/PSMA1/PSMA5/PSMB10/PSMB8/PSMB9/PTAFR/PTGS2/PTPN11/PTPN18/PTPN2/PTPN6/PTPN7/RPS6KA1/S100A12/SAA1/SOCS1/SOCS3/SOD2/SQSTM1/STAT2/STAT5A/STAT6/STX3/STX4/STXBP2/SYK/TEC/TGFB1/TIMP1/TNF/TNFRSF1A/TNFRSF1B/TSLP/TWIST1/UBE2N/VAV1/VCAM1/VIM | 156 | REACTOME |
| REACTOME_DNA_REPLICATION | 3.90E-23 | 3.11E-21 | CCNA2/CCNE1/CCNE2/CDC23/CDC45/CDC6/CDC7/CDK2/CDT1/DBF4/DNA2/E2F1/E2F2/E2F3/FEN1/FZR1/GINS1/GINS2/GINS3/GINS4/GMNN/H2AX/H2AZ1/H2BC17/H2BC9/H3-3A/H3C2/H3C3/KPNB1/LIG1/MCM10/MCM2/MCM3/MCM4/MCM5/MCM6/MCM7/MCM8/ORC1/ORC6/PCNA/POLA1/POLA2/POLD3/POLE/POLE2/POLE3/PRIM1/PRIM2/PSMB2/PSMB7/PSMD11/PSMD14/PSMD3/PSMD5/PSMD6/PSME3/RFC2/RFC3/RFC4/RFC5/RPA1/RPA2/SKP1/SKP2/UBE2C/UBE2S | 67 | REACTOME |
| REACTOME_SYNTHESIS_OF_DNA | 6.82E-23 | 5.23E-21 | CCNA2/CCNE1/CCNE2/CDC23/CDC45/CDC6/CDK2/CDT1/DNA2/FEN1/FZR1/GINS1/GINS2/GINS3/GINS4/LIG1/MCM2/MCM3/MCM4/MCM5/MCM6/MCM7/MCM8/ORC1/ORC6/PCNA/POLA1/POLA2/POLD3/POLE/POLE2/POLE3/PRIM1/PRIM2/PSMB2/PSMB7/PSMD11/PSMD14/PSMD3/PSMD5/PSMD6/PSME3/RFC2/RFC3/RFC4/RFC5/RPA1/RPA2/SKP1/SKP2/UBE2C/UBE2S | 52 | REACTOME |
| REACTOME_NONSENSE_MEDIATED_DECAY_NMD | 7.60E-23 | 1.58E-20 | FAU/MAGOH/PABPC1/PPP2CA/RPL10/RPL10A/RPL11/RPL12/RPL13/RPL13A/RPL14/RPL17/RPL18/RPL18A/RPL19/RPL22L1/RPL23/RPL23A/RPL24/RPL26L1/RPL27/RPL27A/RPL28/RPL29/RPL30/RPL32/RPL35/RPL35A/RPL36/RPL36A/RPL39/RPL41/RPL5/RPL6/RPL7/RPL7A/RPL8/RPL9/RPLP0/RPLP1/RPLP2/RPS10/RPS11/RPS13/RPS14/RPS15/RPS15A/RPS16/RPS17/RPS18/RPS19/RPS2/RPS20/RPS21/RPS25/RPS27L/RPS28/RPS3/RPS3A/RPS4X/RPS5/RPS7/RPS8/RPS9/RPSA/UBA52 | 66 | REACTOME |
| WP_DNA_REPLICATION | 3.43E-22 | 2.54E-20 | CDC45/CDC6/CDC7/CDK2/CDT1/DBF4/GMNN/MCM10/MCM2/MCM3/MCM4/MCM5/MCM6/MCM7/ORC1/ORC6/PCNA/POLA1/POLA2/POLD3/POLE/POLE2/PRIM1/PRIM2/RFC2/RFC3/RFC4/RFC5/RPA1/RPA2 | 30 | WP |
| REACTOME_G2_M_CHECKPOINTS | 8.65E-22 | 6.19E-20 | ATRIP/BARD1/BRCA1/BRIP1/CCNB1/CCNB2/CDC25A/CDC25C/CDC45/CDC6/CDC7/CDK1/CDK2/CHEK1/CHEK2/CLSPN/DBF4/DNA2/EXO1/GTSE1/H2AX/H2BC17/H2BC9/MCM10/MCM2/MCM3/MCM4/MCM5/MCM6/MCM7/MCM8/MRE11/NSD2/ORC1/ORC6/PIAS4/PKMYT1/PSMB2/PSMB7/PSMD11/PSMD14/PSMD3/PSMD5/PSMD6/PSME3/RBBP8/RFC2/RFC3/RFC4/RFC5/RHNO1/RMI1/RMI2/RNF168/RPA1/RPA2/TOP3A/TOPBP1/UIMC1/WRN/YWHAQ | 61 | REACTOME |
| REACTOME_ACTIVATION_OF_THE_PRE_REPLICATIVE_COMPLEX | 3.00E-21 | 2.08E-19 | CDC45/CDC6/CDC7/CDK2/CDT1/DBF4/GMNN/MCM10/MCM2/MCM3/MCM4/MCM5/MCM6/MCM7/MCM8/ORC1/ORC6/POLA1/POLA2/POLE/POLE2/POLE3/PRIM1/PRIM2/RPA1/RPA2 | 26 | REACTOME |
| REACTOME_MRNA_SPLICING | 4.51E-21 | 3.03E-19 | ALYREF/CD2BP2/CHERP/CPSF3/CPSF4/CSTF1/CSTF2/CSTF3/DDX23/DHX16/EFTUD2/EIF4A3/ELAVL1/FIP1L1/FUS/GCFC2/GTF2F2/HNRNPA0/HNRNPA1/HNRNPA2B1/HNRNPA3/HNRNPC/HNRNPD/HNRNPH1/HNRNPK/HNRNPL/HNRNPM/HNRNPR/HNRNPU/HNRNPUL1/LSM4/LSM8/MAGOHB/POLR2D/PPIH/PPIL1/PPIL4/PRCC/PRPF4/PRPF6/PUF60/RBM8A/RBMX/RNPS1/SART1/SF3A2/SF3A3/SF3B2/SF3B3/SF3B4/SNRNP40/SNRPA/SNRPA1/SNRPD1/SRSF1/SRSF10/SRSF2/SRSF3/SRSF7/TRA2B/U2AF2/USP39/WDR33/YBX1 | 64 | REACTOME |
| REACTOME_REGULATION_OF_EXPRESSION_OF_SLITS_AND_ROBOS | 2.50E-21 | 4.81E-19 | FAU/LDB1/MAGOH/PABPC1/PSMA2/PSMA3/PSMA4/PSMA7/PSMB1/PSMB3/PSMB4/PSMC2/PSMC4/PSMD13/PSMD9/PSME1/PSME2/PSMF1/RPL10/RPL10A/RPL11/RPL12/RPL13/RPL13A/RPL14/RPL17/RPL18/RPL18A/RPL19/RPL22L1/RPL23/RPL23A/RPL24/RPL26L1/RPL27/RPL27A/RPL28/RPL29/RPL30/RPL32/RPL35/RPL35A/RPL36/RPL36A/RPL39/RPL41/RPL5/RPL6/RPL7/RPL7A/RPL8/RPL9/RPLP0/RPLP1/RPLP2/RPS10/RPS11/RPS13/RPS14/RPS15/RPS15A/RPS16/RPS17/RPS18/RPS19/RPS2/RPS20/RPS21/RPS25/RPS27L/RPS28/RPS3/RPS3A/RPS4X/RPS5/RPS7/RPS8/RPS9/RPSA/SEM1/SLIT2/UBA52 | 82 | REACTOME |
| REACTOME_INTERLEUKIN_10_SIGNALING | 2.76E-21 | 5.00E-19 | CCL2/CCL20/CCL3/CCL3L3/CCL4/CCL5/CCR1/CCR2/CCR5/CD80/CD86/CSF1/CSF3/CXCL1/CXCL10/CXCL2/CXCL8/FPR1/ICAM1/IL10/IL10RA/IL10RB/IL18/IL1A/IL1B/IL1R1/IL1R2/IL1RN/IL6/LIF/PTAFR/PTGS2/TIMP1/TNF/TNFRSF1A/TNFRSF1B | 36 | REACTOME |
| HALLMARK_COMPLEMENT | 2.93E-21 | 5.00E-19 | ANG/APOBEC3F/APOBEC3G/APOC1/C1QA/C1QC/C1R/C1S/C2/C3/CASP1/CASP10/CASP4/CASP5/CASP7/CCL5/CD36/CD40LG/CD55/CDA/CEBPB/CFB/CFH/CP/CPQ/CR1/CTSB/CTSC/CTSD/CTSH/CTSL/CTSO/CTSS/CXCL1/DPP4/F5/FCER1G/FCN1/FYN/GCA/GNGT2/GRB2/GZMA/GZMB/GZMK/HSPA1A/IL6/IRF1/IRF2/IRF7/ITGAM/JAK2/KIF2A/KYNU/LAP3/LCK/LCP2/LGALS3/LGMN/LIPA/LTA4H/LTF/LYN/OLR1/PIK3CG/PIK3R5/PIM1/PLAUR/PLEK/PLSCR1/PRKCD/PRSS36/PSMB9/RBSN/RHOG/S100A12/S100A9/SCG3/SERPINA1/SERPINE1/SERPING1/STX4/TFPI2/TIMP1/TNFAIP3/WAS | 86 | HALLMARK |
| REACTOME_TRANSLATION | 6.70E-21 | 1.20E-18 | AIMP1/AIMP2/APEH/DAP3/DDOST/EEF1A1/EEF1A2/EEF1D/EEF1G/EIF2B2/EIF2S3/EIF3B/EIF3D/EIF3E/EIF3G/EIF3I/EIF3M/EIF4A1/EIF4A2/EIF4EBP1/FAU/GFM2/MRPL11/MRPL12/MRPL13/MRPL14/MRPL17/MRPL32/MRPL36/MRPL4/MRPL47/MRPL51/MRPL58/MRPS12/MRPS18B/MRPS24/OXA1L/PABPC1/QARS1/RPL10/RPL10A/RPL11/RPL12/RPL13/RPL13A/RPL14/RPL17/RPL18/RPL18A/RPL19/RPL22L1/RPL23/RPL23A/RPL24/RPL26L1/RPL27/RPL27A/RPL28/RPL29/RPL30/RPL32/RPL35/RPL35A/RPL36/RPL36A/RPL39/RPL41/RPL5/RPL6/RPL7/RPL7A/RPL8/RPL9/RPLP0/RPLP1/RPLP2/RPN1/RPN2/RPS10/RPS11/RPS13/RPS14/RPS15/RPS15A/RPS16/RPS17/RPS18/RPS19/RPS2/RPS20/RPS21/RPS25/RPS27L/RPS28/RPS3/RPS3A/RPS4X/RPS5/RPS7/RPS8/RPS9/RPSA/SEC11A/SEC61A1/SEC61A2/SEC61B/SEC61G/SPCS2/SRPRB/SSR1/SSR2/SSR4/TARS1/TRMT112/UBA52/WARS2 | 116 | REACTOME |
| REACTOME_HDR_THROUGH_HOMOLOGOUS_RECOMBINATION_HRR | 3.66E-20 | 2.38E-18 | ATRIP/BARD1/BRCA1/BRCA2/BRIP1/CHEK1/DNA2/EME1/EXO1/GEN1/MRE11/MUS81/PCNA/POLD3/POLE/POLE2/POLE3/POLH/RAD51/RAD51AP1/RAD51D/RBBP8/RFC2/RFC3/RFC4/RFC5/RHNO1/RMI1/RMI2/RPA1/RPA2/RTEL1/TOP3A/TOPBP1/WRN/XRCC2 | 36 | REACTOME |
| KEGG_DNA_REPLICATION | 1.35E-19 | 8.37E-18 | DNA2/FEN1/LIG1/MCM2/MCM3/MCM4/MCM5/MCM6/MCM7/PCNA/POLA1/POLA2/POLD3/POLE/POLE2/POLE3/PRIM1/PRIM2/RFC2/RFC3/RFC4/RFC5/RNASEH2A/RNASEH2B/RPA1/RPA2 | 26 | KEGG |
| REACTOME_CHROMOSOME_MAINTENANCE | 1.36E-19 | 8.37E-18 | CCNA2/CDK2/CENPA/CENPH/CENPI/CENPK/CENPL/CENPM/CENPN/CENPO/CENPP/CENPQ/CENPU/CENPW/CENPX/CHTF18/DKC1/DNA2/DSCC1/FEN1/H2AX/H2AZ1/H2BC17/H2BC9/H3-3A/HJURP/KNL1/LIG1/MIS18A/MIS18BP1/OIP5/PCNA/PIF1/POLA1/POLA2/POLD3/POLR2D/PRIM1/PRIM2/RBBP4/RBBP7/RFC2/RFC3/RFC4/RFC5/RPA1/RPA2/RTEL1/RUVBL1/SHQ1/WRAP53/WRN | 52 | REACTOME |
| WP_ALLOGRAFT_REJECTION | 7.15E-20 | 1.15E-17 | C1QA/C1QB/C1QC/C2/C3/C7/CASP7/CASP8/CD28/CD40/CD40LG/CD55/CD80/CD86/CTLA4/CXCL11/CXCL12/CXCL13/CXCL8/CXCL9/FAS/FASLG/FOXP3/GNLY/GZMB/HARS1/HLA-A/HLA-B/HLA-C/HLA-DMA/HLA-DMB/HLA-DOA/HLA-DOB/HLA-DPA1/HLA-DPB1/HLA-DQA1/HLA-DQA2/HLA-DQB1/HLA-DRA/HLA-DRB1/HLA-E/HLA-F/IL10/IL1A/IL1B/IL2RA/MICA/PRF1/TGFB1/TNF/VIM | 51 | WP |
| REACTOME_ACTIVATION_OF_ATR_IN_RESPONSE_TO_REPLICATION_STRESS | 4.15E-19 | 2.48E-17 | ATRIP/CDC25A/CDC25C/CDC45/CDC6/CDC7/CDK2/CHEK1/CLSPN/DBF4/MCM10/MCM2/MCM3/MCM4/MCM5/MCM6/MCM7/MCM8/ORC1/ORC6/RFC2/RFC3/RFC4/RFC5/RPA1/RPA2 | 26 | REACTOME |
| REACTOME_INTERFERON_GAMMA_SIGNALING | 9.88E-19 | 1.50E-16 | B2M/CD44/CIITA/FCGR1A/FCGR1B/GBP1/GBP2/GBP3/GBP4/GBP5/HLA-A/HLA-B/HLA-C/HLA-DPA1/HLA-DPB1/HLA-DQA1/HLA-DQA2/HLA-DQB1/HLA-DRA/HLA-DRB1/HLA-E/HLA-F/ICAM1/IFI30/IFNGR1/IFNGR2/IRF1/IRF2/IRF5/IRF7/IRF8/IRF9/JAK2/MT2A/OAS1/OAS2/OASL/PML/PRKCD/PTAFR/PTPN11/PTPN2/PTPN6/SOCS1/SOCS3/SP100/TRIM21/TRIM22/TRIM34/TRIM38/VCAM1 | 51 | REACTOME |
| PID_IL12_2PATHWAY | 1.77E-18 | 2.55E-16 | B2M/CCL3/CCL4/CCR5/CD247/CD3D/CD3E/CD3G/CD4/CD8A/CD8B/EOMES/FASLG/FOS/GADD45B/GZMA/GZMB/HLA-A/HLA-DRA/HLX/IL12RB1/IL18/IL18R1/IL18RAP/IL1B/IL1R1/IL2RA/IL2RB/IL2RG/JAK2/LCK/MAP2K3/MAP2K6/NFKB1/NFKB2/RELB/SOCS1/SPHK2/STAT5A/STAT6 | 40 | PID |
| REACTOME_REGULATION_OF_TP53_ACTIVITY | 8.75E-18 | 5.08E-16 | ATRIP/AURKA/AURKB/BANP/BARD1/BRCA1/BRD1/BRIP1/BRPF1/CCNA2/CDK1/CDK2/CHEK1/CHEK2/DNA2/DYRK2/EHMT1/EXO1/HDAC2/KMT5A/MAPKAP1/MAPKAPK5/MRE11/MTA2/NOC2L/PRKAB1/PRKAG1/PRMT5/RBBP4/RBBP7/RBBP8/RFC2/RFC3/RFC4/RFC5/RHNO1/RMI1/RMI2/RPA1/RPA2/SSRP1/SUPT16H/TAF11/TAF15/TAF4/TAF6/TBP/TOP3A/TOPBP1/TP73/TPX2/TTC5/WRN/ZNF385A | 54 | REACTOME |
| PID_FANCONI_PATHWAY | 1.35E-17 | 7.66E-16 | ATRIP/BRCA1/BRCA2/BRIP1/CHEK1/FAAP100/FAAP24/FANCA/FANCB/FANCC/FANCD2/FANCE/FANCG/FANCI/FANCM/H2AX/MRE11/RFC2/RFC3/RFC4/RFC5/RMI1/RPA1/RPA2/TOP3A/TOPBP1/UBE2T/USP1 | 28 | PID |
| WP_CALCIUM_REGULATION_IN_CARDIAC_CELLS | 4.98E-18 | 8.39E-16 | ADCY1/ADCY2/ADCY5/ADRA1A/ADRA1B/ADRB1/ANXA6/ARRB1/ATP1B1/ATP2B1/ATP2B2/ATP2B3/CACNA1A/CACNA1C/CACNA1D/CACNA1E/CACNB1/CACNB3/CALM1/CALM2/CALM3/CALR/CAMK2A/CAMK2B/CAMK2G/CAMK4/CASQ1/CASQ2/CHRM1/CHRM3/CHRM4/GJA3/GJB5/GJB6/GNAI1/GNAI2/GNAI3/GNAO1/GNAQ/GNAZ/GNB1/GNB2/GNB5/GNG12/GNG3/GNG5/ITPR1/KCNB1/KCNJ3/PKIA/PLCB3/PRKACB/PRKAR1A/PRKAR1B/PRKAR2B/PRKCB/PRKCE/PRKCG/PRKCZ/RGS11/RGS20/RGS4/RGS5/RGS7/RYR1/RYR2/SLC8A1/YWHAB/YWHAG/YWHAH/YWHAZ | 71 | WP |
| REACTOME_RRNA_PROCESSING | 7.25E-18 | 1.15E-15 | BUD23/DDX47/DHX37/EMG1/ERI1/EXOSC4/EXOSC7/FAU/FBL/GAR1/GNL3/HSD17B10/ISG20L2/MRM2/NIP7/NOP2/PNO1/RPL10/RPL10A/RPL11/RPL12/RPL13/RPL13A/RPL14/RPL17/RPL18/RPL18A/RPL19/RPL22L1/RPL23/RPL23A/RPL24/RPL26L1/RPL27/RPL27A/RPL28/RPL29/RPL30/RPL32/RPL35/RPL35A/RPL36/RPL36A/RPL39/RPL41/RPL5/RPL6/RPL7/RPL7A/RPL8/RPL9/RPLP0/RPLP1/RPLP2/RPS10/RPS11/RPS13/RPS14/RPS15/RPS15A/RPS16/RPS17/RPS18/RPS19/RPS2/RPS20/RPS21/RPS25/RPS27L/RPS28/RPS3/RPS3A/RPS4X/RPS5/RPS7/RPS8/RPS9/RPSA/RRP36/RRP9/SNU13/TRMT112/UBA52/UTP15/UTP6/WDR46 | 86 | REACTOME |
| REACTOME_REGULATION_OF_TP53_ACTIVITY_THROUGH_PHOSPHORYLATION | 4.28E-17 | 2.36E-15 | ATRIP/AURKA/AURKB/BARD1/BRCA1/BRIP1/CCNA2/CDK2/CHEK1/CHEK2/DNA2/DYRK2/EXO1/MAPKAPK5/MRE11/NOC2L/PRKAB1/PRKAG1/RBBP8/RFC2/RFC3/RFC4/RFC5/RHNO1/RMI1/RMI2/RPA1/RPA2/SSRP1/SUPT16H/TAF11/TAF15/TAF4/TAF6/TBP/TOP3A/TOPBP1/TPX2/WRN | 39 | REACTOME |
| REACTOME_HOMOLOGOUS_DNA_PAIRING_AND_STRAND_EXCHANGE | 5.10E-17 | 2.74E-15 | ATRIP/BARD1/BRCA1/BRCA2/BRIP1/CHEK1/DNA2/EXO1/MRE11/RAD51/RAD51AP1/RAD51D/RBBP8/RFC2/RFC3/RFC4/RFC5/RHNO1/RMI1/RMI2/RPA1/RPA2/TOP3A/TOPBP1/WRN/XRCC2 | 26 | REACTOME |
| REACTOME_NEUROTRANSMITTER_RECEPTORS_AND_POSTSYNAPTIC_SIGNAL_TRANSMISSION | 2.68E-17 | 4.01E-15 | ADCY1/ADCY2/ADCY5/AKAP5/AP2A1/AP2A2/AP2M1/APBA1/ARHGEF9/CACNG3/CACNG8/CALM1/CAMK2A/CAMK2B/CAMK2G/CAMK4/CAMKK1/CAMKK2/CHRNA7/CHRNB2/DLG2/DLG3/DLG4/EPB41L1/ERBB4/GABBR1/GABBR2/GABRA1/GABRA2/GABRA4/GABRA5/GABRB1/GABRB2/GABRB3/GIT1/GLRA2/GLRB/GNAI1/GNAI2/GNAI3/GNAL/GNB1/GNB2/GNB5/GNG12/GNG3/GNG5/GRIN1/GRIN2A/GRIN2B/GRIN2C/GRIN3A/GRIP1/GRIP2/KCNJ12/KCNJ3/KCNJ4/KCNJ6/KCNJ9/KIF17/LIN7B/LRRC7/MAPK1/MAPK3/NBEA/NEFL/NPTN/NRGN/NSF/PDPK1/PLCB1/PLCB3/PRKAB2/PRKACB/PRKAG2/PRKAR1A/PRKAR1B/PRKAR2B/PRKCB/PRKCG/RAC1/RASGRF2/TUBA4A/TUBA8/TUBB2A | 85 | REACTOME |
| HALLMARK_IL2_STAT5_SIGNALING | 3.69E-17 | 5.03E-15 | AHNAK/ANXA4/BATF/CAPG/CCND2/CCR4/CD44/CD48/CD79B/CD81/CD83/CD86/CDCP1/CISH/CSF1/CST7/CTLA4/CTSZ/CXCL10/CYFIP1/DHRS3/ENPP1/EOMES/ETFBKMT/FAH/FGL2/GADD45B/GALM/GBP4/GPR65/ICOS/IFITM3/IFNGR1/IL10/IL10RA/IL18R1/IL1R2/IL2RA/IL2RB/IL3RA/IL4R/IRF8/LIF/LTB/MAP3K8/MUC1/MXD1/MYO1E/NDRG1/NFKBIZ/P2RX4/PIM1/PLAGL1/PLIN2/PLSCR1/PRKCH/PTCH1/PTGER2/RGS16/RHOH/RNH1/SELL/SLC1A5/SLC2A3/SLC39A8/SMPDL3A/SNX9/SOCS1/SPP1/SWAP70/SYNGR2/TGM2/TLR7/TNFRSF1B/TNFRSF4/TNFRSF8/TNFSF10/TRAF1/XBP1 | 79 | HALLMARK |
| REACTOME_INFLUENZA_INFECTION | 4.44E-17 | 6.29E-15 | CALR/FAU/NUP37/NUP54/NUP62/POLR2G/POLR2H/POLR2J/RPL10/RPL10A/RPL11/RPL12/RPL13/RPL13A/RPL14/RPL17/RPL18/RPL18A/RPL19/RPL22L1/RPL23/RPL23A/RPL24/RPL26L1/RPL27/RPL27A/RPL28/RPL29/RPL30/RPL32/RPL35/RPL35A/RPL36/RPL36A/RPL39/RPL41/RPL5/RPL6/RPL7/RPL7A/RPL8/RPL9/RPLP0/RPLP1/RPLP2/RPS10/RPS11/RPS13/RPS14/RPS15/RPS15A/RPS16/RPS17/RPS18/RPS19/RPS2/RPS20/RPS21/RPS25/RPS27L/RPS28/RPS3/RPS3A/RPS4X/RPS5/RPS7/RPS8/RPS9/RPSA/SEC13/UBA52 | 71 | REACTOME |
| PID_E2F_PATHWAY | 1.31E-16 | 6.86E-15 | BRCA1/CCNA2/CCNE1/CCNE2/CDC25A/CDC6/CDK1/CDK2/CDKN2C/DHFR/E2F1/E2F2/E2F3/E2F5/E2F6/E2F7/MCM3/MYBL2/ORC1/POLA1/PRMT5/RANBP1/RBBP4/RBBP8/RBL1/RRM1/RRM2/TFDP1/TK1/TOPBP1/TP73/TRIM28/TYMS/XRCC1 | 34 | PID |
| REACTOME_G1_S_SPECIFIC_TRANSCRIPTION | 4.22E-16 | 2.11E-14 | CCNE1/CDC25A/CDC45/CDC6/CDK1/CDT1/DHFR/E2F1/E2F5/E2F6/FBXO5/LIN9/ORC1/PCNA/POLA1/RBBP4/RBL1/RRM2/TFDP1/TK1/TYMS | 21 | REACTOME |
| HALLMARK_KRAS_SIGNALING_UP | 1.93E-16 | 2.50E-14 | ADAM8/ADAMDEC1/ALDH1A2/BIRC3/C3AR1/CBR4/CCL20/CCND2/CD37/CDADC1/CFB/CFH/CLEC4A/CMKLR1/CSF2RA/CTSS/CXCL10/CXCR4/DCBLD2/DOCK2/F13A1/FCER1G/FUCA1/G0S2/GFPT2/GLRX/GNG11/GPNMB/GPRC5B/GYPC/HBEGF/IKZF1/IL10RA/IL1B/IL2RG/IL7R/INHBA/IRF8/ITGA2/ITGB2/ITGBL1/KCNN4/KLF4/LAPTM5/LAT2/LCP1/LIF/LY96/MAFB/MALL/MAP4K1/MMP10/NGF/PDCD1LG2/PLAU/PLAUR/PPBP/PPP1R15A/PRDM1/PSMB8/PTGS2/RETN/RGS16/SCG3/SERPINA3/SLPI/SPON1/SPP1/TLR8/TMEM176A/TMEM176B/TNFAIP3/TNFRSF1B/TRAF1/TRIB1/TSPAN1/TSPAN7/VWA5A | 78 | HALLMARK |
| KEGG_CELL_ADHESION_MOLECULES_CAMS | 2.02E-16 | 2.50E-14 | CD2/CD226/CD274/CD28/CD4/CD40/CD40LG/CD6/CD80/CD86/CD8A/CD8B/CD99/CLDN1/CLDN23/CLDN4/CLDN7/CNTN1/CTLA4/F11R/HLA-A/HLA-B/HLA-C/HLA-DMA/HLA-DMB/HLA-DOA/HLA-DOB/HLA-DPA1/HLA-DPB1/HLA-DQA1/HLA-DQA2/HLA-DQB1/HLA-DRA/HLA-DRB1/HLA-E/HLA-F/ICAM1/ICAM3/ICOS/ICOSLG/ITGAL/ITGAM/ITGB2/ITGB7/NCAM2/NECTIN2/NEO1/NLGN3/NRXN2/PDCD1/PDCD1LG2/PTPRC/SDC2/SDC3/SELE/SELL/SELPLG/SIGLEC1/SPN/VCAM1 | 60 | KEGG |
| WP_G1_TO_S_CELL_CYCLE_CONTROL | 7.21E-16 | 3.52E-14 | CCNB1/CCNE1/CCNE2/CDC25A/CDC45/CDK1/CDK2/CDK4/CDK6/CDKN2C/CREB3L4/E2F1/E2F2/E2F3/MCM2/MCM3/MCM4/MCM5/MCM6/MCM7/ORC1/ORC6/PCNA/POLA2/POLE/POLE2/PRIM1/PRIM2/RPA1/RPA2/TFDP1 | 31 | WP |
| KEGG_LEISHMANIA_INFECTION | 4.15E-16 | 4.92E-14 | C3/CR1/CYBA/FCGR1A/FCGR2A/FCGR2C/FCGR3A/FCGR3B/FOS/HLA-DMA/HLA-DMB/HLA-DOA/HLA-DOB/HLA-DPA1/HLA-DPB1/HLA-DQA1/HLA-DQA2/HLA-DQB1/HLA-DRA/HLA-DRB1/IFNGR1/IFNGR2/IL10/IL1A/IL1B/ITGAM/ITGB2/JAK2/JUN/MAPK13/NCF1/NCF2/NCF4/NFKB1/NFKBIA/PTGS2/PTPN6/TGFB1/TLR2/TLR4/TNF | 41 | KEGG |
| PID_PLK1_PATHWAY | 1.19E-15 | 5.66E-14 | AURKA/BORA/BUB1/BUB1B/CCNB1/CDC20/CDC25B/CDC25C/CDK1/CENPE/CENPU/CLSPN/ECT2/ERCC6L/FBXO5/FZR1/INCENP/KIF20A/NDC80/ODF2/PLK1/PRC1/SGO1/SPC24/TPX2/TUBG1 | 26 | PID |
| WP_MICROGLIA_PATHOGEN_PHAGOCYTOSIS_PATHWAY | 7.58E-16 | 8.61E-14 | ARPC1B/C1QA/C1QB/C1QC/CYBA/CYBB/FCER1G/FCGR1A/HCK/ITGAM/ITGB2/LYN/NCF1/NCF2/NCF4/NCKAP1L/PIK3C3/PIK3CD/PIK3CG/PIK3R6/PLCG2/PTPN6/RAC2/SIGLEC7/SYK/TREM1/TREM2/TYROBP/VAV1 | 29 | WP |
| WP_MRNA_PROCESSING | 4.36E-15 | 1.99E-13 | CD2BP2/CPSF3/CPSF4/CSTF1/CSTF2/CSTF3/DHX16/EFTUD2/FUS/HNRNPA1/HNRNPA2B1/HNRNPAB/HNRNPC/HNRNPD/HNRNPH1/HNRNPK/HNRNPL/HNRNPM/HNRNPR/HNRNPU/NONO/PPM1G/PRPF4/PRPF6/RBMX/RNPS1/SF3A2/SF3A3/SF3B2/SF3B3/SF3B4/SMC1A/SNRNP40/SNRPA/SNRPA1/SNRPD1/SRPK1/SRSF1/SRSF10/SRSF2/SRSF3/SRSF7/TRA2B/U2AF2/YBX1 | 45 | WP |
| KEGG_ALLOGRAFT_REJECTION | 5.87E-15 | 6.41E-13 | CD28/CD40/CD40LG/CD80/CD86/FAS/FASLG/GZMB/HLA-A/HLA-B/HLA-C/HLA-DMA/HLA-DMB/HLA-DOA/HLA-DOB/HLA-DPA1/HLA-DPB1/HLA-DQA1/HLA-DQA2/HLA-DQB1/HLA-DRA/HLA-DRB1/HLA-E/HLA-F/IL10/PRF1/TNF | 27 | KEGG |
| REACTOME_RHO_GTPASE_EFFECTORS | 1.48E-14 | 6.57E-13 | ABL1/AURKB/BIRC5/BUB1/BUB1B/BUB3/CDC20/CDC25C/CDCA8/CENPA/CENPE/CENPF/CENPH/CENPI/CENPK/CENPL/CENPM/CENPN/CENPO/CENPP/CENPQ/CENPU/DIAPH1/DIAPH3/DSN1/DVL2/DVL3/ERCC6L/H2AX/H2AZ1/H2BC17/H2BC9/H3-3A/H3C2/H3C3/INCENP/IQGAP3/KDM1A/KIF14/KIF18A/KIF2C/KNL1/KNTC1/MAD1L1/MAD2L1/MEN1/MIS12/NDC80/NDE1/NOXA1/NSL1/NUF2/NUP107/NUP160/NUP43/NUP85/PAK2/PKN3/PLK1/PMF1/PPP1CC/PRC1/RCC2/SGO1/SGO2/SKA1/SKA2/SPC24/SPC25/SPDL1/TUBA1A/TUBA1B/YWHAQ/ZW10/ZWILCH/ZWINT | 76 | REACTOME |
| PID_ATR_PATHWAY | 1.50E-14 | 6.57E-13 | ATRIP/BRCA2/CCNA2/CDC25A/CDC25C/CDC6/CDK2/CHEK1/CLSPN/FANCD2/MCM2/MCM7/PLK1/RAD51/RFC2/RFC3/RFC4/RFC5/RPA1/RPA2/TIMELESS/TIPIN/TOPBP1 | 23 | PID |
| HALLMARK_APOPTOSIS | 8.98E-15 | 9.42E-13 | ADD1/ANXA1/ATF3/BCAP31/BIK/BIRC3/BMF/BTG2/CASP1/CASP4/CASP7/CASP8/CCND2/CD14/CD2/CD44/CD69/CDKN1A/CDKN1B/CFLAR/DCN/DPYD/FAS/FASLG/GADD45A/GADD45B/GCH1/GNA15/GPX1/GPX3/GSN/HGF/HMOX1/IER3/IFITM3/IFNGR1/IGFBP6/IL18/IL1A/IL1B/IL6/IRF1/ISG20/JUN/KRT18/LEF1/LGALS3/PLCB2/PMAIP1/PPT1/PRF1/PTK2/SAT1/SLC20A1/SMAD7/SOD2/SPTAN1/SQSTM1/TAP1/TIMP1/TNF/TNFRSF12A/TNFSF10/TSPO/TXNIP | 65 | HALLMARK |
| REACTOME_PROCESSING_OF_DNA_DOUBLE_STRAND_BREAK_ENDS | 2.34E-14 | 9.84E-13 | ATRIP/BARD1/BRCA1/BRIP1/CCNA2/CDK2/CHEK1/CLSPN/DNA2/EXO1/H2AX/H2BC17/H2BC9/MRE11/NSD2/PIAS4/PPP4R2/RBBP8/RFC2/RFC3/RFC4/RFC5/RHNO1/RMI1/RMI2/RNF168/RNF4/RPA1/RPA2/SUMO2/TIMELESS/TIPIN/TOP3A/TOPBP1/UBE2I/UIMC1/WRN | 37 | REACTOME |
| KEGG_HEMATOPOIETIC_CELL_LINEAGE | 1.23E-14 | 1.25E-12 | ANPEP/CD14/CD1C/CD1D/CD2/CD33/CD36/CD37/CD3D/CD3E/CD3G/CD4/CD44/CD5/CD55/CD7/CD8A/CD8B/CR1/CSF1/CSF1R/CSF2RA/CSF3/CSF3R/EPOR/FCGR1A/HLA-DRA/HLA-DRB1/IL11/IL1A/IL1B/IL1R1/IL1R2/IL2RA/IL3RA/IL4R/IL6/IL6R/IL7/IL7R/ITGA2/ITGAM/MME/TNF | 44 | KEGG |
| REACTOME_EXTENSION_OF_TELOMERES | 3.31E-14 | 1.37E-12 | CCNA2/CDK2/CHTF18/DKC1/DNA2/DSCC1/FEN1/LIG1/PCNA/PIF1/POLA1/POLA2/POLD3/PRIM1/PRIM2/RFC2/RFC3/RFC4/RFC5/RPA1/RPA2/RTEL1/RUVBL1/SHQ1/WRAP53/WRN | 26 | REACTOME |
| REACTOME_HDR_THROUGH_SINGLE_STRAND_ANNEALING_SSA | 4.45E-14 | 1.80E-12 | ABL1/ATRIP/BARD1/BRCA1/BRIP1/DNA2/EXO1/MRE11/RAD51/RBBP8/RFC2/RFC3/RFC4/RFC5/RHNO1/RMI1/RMI2/RPA1/RPA2/TOP3A/TOPBP1/WRN | 22 | REACTOME |
| REACTOME_INTERFERON_SIGNALING | 2.19E-14 | 2.13E-12 | B2M/BST2/CD44/CIITA/EGR1/FCGR1A/FCGR1B/FLNB/GBP1/GBP2/GBP3/GBP4/GBP5/HLA-A/HLA-B/HLA-C/HLA-DPA1/HLA-DPB1/HLA-DQA1/HLA-DQA2/HLA-DQB1/HLA-DRA/HLA-DRB1/HLA-E/HLA-F/ICAM1/IFI27/IFI30/IFI35/IFIT2/IFIT3/IFITM1/IFITM2/IFITM3/IFNGR1/IFNGR2/IRF1/IRF2/IRF5/IRF7/IRF8/IRF9/ISG20/JAK2/KPNA3/KPNA5/MT2A/MX1/MX2/OAS1/OAS2/OASL/PML/PPM1B/PRKCD/PSMB8/PTAFR/PTPN11/PTPN2/PTPN6/RSAD2/SAMHD1/SOCS1/SOCS3/SP100/STAT2/TRIM21/TRIM22/TRIM34/TRIM38/UBA7/UBE2L6/UBE2N/VCAM1/XAF1 | 75 | REACTOME |
| REACTOME_MITOTIC_G2_G2_M_PHASES | 9.21E-14 | 3.60E-12 | AURKA/BORA/CCNA2/CCNB1/CCNB2/CDC25A/CDC25B/CDC25C/CDK1/CDK2/CENPF/CENPJ/CEP131/CEP41/CEP72/CEP76/DCTN3/E2F1/E2F3/FOXM1/GTSE1/HAUS1/HAUS2/HAUS3/HAUS6/HAUS8/HMMR/LIN9/MYBL2/NDE1/NEDD1/NEK2/ODF2/PHLDA1/PKMYT1/PLK1/PLK4/PPP2R2A/PPP2R3B/PSMB2/PSMB7/PSMD11/PSMD14/PSMD3/PSMD5/PSMD6/PSME3/RBBP4/SKP1/TPX2/TUBA1A/TUBA1B/TUBB/TUBG1/TUBGCP3 | 55 | REACTOME |
| REACTOME_CHEMOKINE_RECEPTORS_BIND_CHEMOKINES | 4.19E-14 | 3.94E-12 | CCL2/CCL20/CCL3/CCL3L3/CCL4/CCL5/CCL7/CCR1/CCR2/CCR4/CCR5/CCR6/CCR7/CCRL2/CXCL1/CXCL10/CXCL11/CXCL12/CXCL13/CXCL16/CXCL2/CXCL3/CXCL5/CXCL6/CXCL8/CXCL9/CXCR1/CXCR2/CXCR3/CXCR4/CXCR6/PPBP/XCL1/XCL2 | 34 | REACTOME |
| REACTOME_TRANSPORT_OF_MATURE_TRANSCRIPT_TO_CYTOPLASM | 1.57E-13 | 6.02E-12 | ALYREF/CHTOP/CPSF3/CPSF4/DDX39B/EIF4A3/FIP1L1/GLE1/MAGOHB/NDC1/NUP107/NUP155/NUP160/NUP188/NUP205/NUP210/NUP35/NUP42/NUP43/NUP50/NUP85/NUP88/RAE1/RBM8A/RNPS1/SLBP/SRSF1/SRSF2/SRSF3/SRSF7/THOC5/U2AF2/WDR33 | 33 | REACTOME |
| REACTOME_ACTIVATION_OF_THE_MRNA_UPON_BINDING_OF_THE_CAP_BINDING_COMPLEX_AND_EIFS_AND_SUBSEQUENT_BINDING_TO_43S | 4.61E-14 | 6.21E-12 | EIF2S3/EIF3B/EIF3D/EIF3E/EIF3G/EIF3I/EIF3M/EIF4A1/EIF4A2/EIF4EBP1/FAU/PABPC1/RPS10/RPS11/RPS13/RPS14/RPS15/RPS15A/RPS16/RPS17/RPS18/RPS19/RPS2/RPS20/RPS21/RPS25/RPS27L/RPS28/RPS3/RPS3A/RPS4X/RPS5/RPS7/RPS8/RPS9/RPSA | 36 | REACTOME |
| REACTOME_SUMOYLATION_OF_DNA_REPLICATION_PROTEINS | 1.65E-13 | 6.23E-12 | AURKA/AURKB/BIRC5/CDCA8/INCENP/NDC1/NUP107/NUP155/NUP160/NUP188/NUP205/NUP210/NUP35/NUP42/NUP43/NUP50/NUP85/NUP88/PCNA/PIAS4/RAE1/SUMO2/TOP2A/UBE2I | 24 | REACTOME |
| REACTOME_INTERLEUKIN_4_AND_INTERLEUKIN_13_SIGNALING | 6.97E-14 | 6.34E-12 | ALOX5/ANXA1/BATF/CCL2/CD36/CDKN1A/CEBPD/CXCL8/F13A1/FASLG/FOS/FOXO3/HGF/HMOX1/ICAM1/IGHG1/IGHG4/IL10/IL13RA1/IL18/IL1A/IL1B/IL23A/IL2RG/IL4R/IL6/IL6R/ITGAM/ITGAX/ITGB2/JAK2/JAK3/JUNB/LIF/MMP1/MUC1/OSM/PIM1/PTGS2/SAA1/SOCS1/SOCS3/STAT6/TGFB1/TIMP1/TNF/TNFRSF1B/TWIST1/VCAM1/VIM | 50 | REACTOME |
| REACTOME_DNA_REPLICATION_PRE_INITIATION | 2.03E-13 | 7.52E-12 | CDC45/CDC6/CDC7/CDK2/CDT1/DBF4/E2F1/E2F2/E2F3/GMNN/H2AX/H2AZ1/H2BC17/H2BC9/H3-3A/H3C2/H3C3/KPNB1/MCM10/MCM2/MCM3/MCM4/MCM5/MCM6/MCM7/MCM8/ORC1/ORC6/POLA1/POLA2/POLE/POLE2/POLE3/PRIM1/PRIM2/PSMB2/PSMB7/PSMD11/PSMD14/PSMD3/PSMD5/PSMD6/PSME3/RPA1/RPA2 | 45 | REACTOME |
| HALLMARK_P53_PATHWAY | 9.99E-14 | 8.80E-12 | ABAT/ALOX15B/ATF3/BTG2/CASP1/CCND2/CCNG1/CD81/CDKN1A/CEBPA/CSRNP2/CTSD/DDIT4/DEF6/DRAM1/FAS/FOS/FOXO3/FUCA1/GADD45A/GM2A/H1-2/H2AJ/HBEGF/HMOX1/IER3/IFI30/IL1A/IRAG2/ITGB4/JUN/KLF4/LDHB/LIF/MAPKAPK3/MKNK2/MXD1/NDRG1/NINJ1/NUPR1/OSGIN1/PERP/PHLDA3/PITPNC1/PLK3/PPP1R15A/PROCR/RAP2B/RCHY1/RGS16/RHBDF2/RNF19B/RRAD/S100A10/S100A4/SAT1/SERTAD3/SOCS1/SPHK1/ST14/STEAP3/STOM/TAP1/TAX1BP3/TCN2/TGFB1/TSC22D1/TXNIP/UPP1/VAMP8/VDR/VWA5A/ZFP36L1 | 73 | HALLMARK |
| REACTOME_SUMOYLATION_OF_RNA_BINDING_PROTEINS | 3.08E-13 | 1.12E-11 | CBX2/CBX8/HNRNPC/HNRNPK/NDC1/NOP58/NUP107/NUP155/NUP160/NUP188/NUP205/NUP210/NUP35/NUP42/NUP43/NUP50/NUP85/NUP88/PCGF2/RAE1/RING1/RNF2/SUMO2/UBE2I | 24 | REACTOME |
| PID_FOXM1_PATHWAY | 4.09E-13 | 1.46E-11 | AURKB/BIRC5/BRCA2/CCNA2/CCNB1/CCNB2/CCNE1/CDC25B/CDK1/CDK2/CDK4/CENPA/CENPF/CHEK2/CKS1B/FOXM1/GAS1/MMP2/NEK2/PLK1/SKP2/XRCC1 | 22 | PID |
| REACTOME_UNWINDING_OF_DNA | 4.20E-13 | 1.48E-11 | CDC45/GINS1/GINS2/GINS3/GINS4/MCM2/MCM3/MCM4/MCM5/MCM6/MCM7/MCM8 | 12 | REACTOME |
| KEGG_SPLICEOSOME | 4.31E-13 | 1.49E-11 | ALYREF/CHERP/DDX23/DDX39B/DHX16/EFTUD2/EIF4A3/HNRNPA1/HNRNPA3/HNRNPC/HNRNPK/HNRNPM/HNRNPU/LSM4/LSM8/MAGOHB/PPIH/PPIL1/PRPF4/PRPF6/PUF60/RBM8A/RBMX/SART1/SF3A2/SF3A3/SF3B2/SF3B3/SF3B4/SNRNP40/SNRPA/SNRPA1/SNRPD1/SRSF1/SRSF10/SRSF2/SRSF3/SRSF7/TRA2B/U2AF2/USP39 | 41 | KEGG |
| WP_PROSTAGLANDIN_SIGNALING | 2.21E-13 | 1.88E-11 | AREG/CASP1/CCL2/CCL3/CCR2/CD28/CSF1/CXCL1/CXCL10/CXCL8/CXCL9/IL1A/IL1B/IL6/IRF7/NFKB1/NLRP3/PIK3CG/PTGER2/PTGER4/PTGES/PYCARD/TGFB1/TNF | 24 | WP |
| KEGG_GRAFT_VERSUS_HOST_DISEASE | 3.17E-13 | 2.62E-11 | CD28/CD80/CD86/FAS/FASLG/GZMB/HLA-A/HLA-B/HLA-C/HLA-DMA/HLA-DMB/HLA-DOA/HLA-DOB/HLA-DPA1/HLA-DPB1/HLA-DQA1/HLA-DQA2/HLA-DQB1/HLA-DRA/HLA-DRB1/HLA-E/HLA-F/IL1A/IL1B/IL6/PRF1/TNF | 27 | KEGG |
| REACTOME_DISEASES_OF_DNA_REPAIR | 8.59E-13 | 2.88E-11 | BARD1/BRCA1/BRCA2/BRIP1/DNA2/EXO1/MLH1/MRE11/MSH2/MSH6/NEIL3/RAD51/RAD51AP1/RAD51D/RBBP8/RMI1/RMI2/TOP3A/WRN/XRCC2 | 20 | REACTOME |
| REACTOME_RESOLUTION_OF_D_LOOP_STRUCTURES | 8.59E-13 | 2.88E-11 | BARD1/BRCA1/BRCA2/BRIP1/DNA2/EME1/EXO1/GEN1/MRE11/MUS81/RAD51/RAD51AP1/RAD51D/RBBP8/RMI1/RMI2/RTEL1/TOP3A/WRN/XRCC2 | 20 | REACTOME |
| KEGG_CHEMOKINE_SIGNALING_PATHWAY | 4.70E-13 | 3.77E-11 | ADCY7/AKT3/ARRB2/CCL18/CCL2/CCL20/CCL26/CCL3/CCL3L3/CCL4/CCL4L2/CCL5/CCL7/CCL8/CCR1/CCR2/CCR4/CCR5/CCR6/CCR7/CRKL/CSK/CXCL1/CXCL10/CXCL11/CXCL12/CXCL13/CXCL16/CXCL2/CXCL3/CXCL5/CXCL6/CXCL8/CXCL9/CXCR1/CXCR2/CXCR3/CXCR4/CXCR6/DOCK2/FGR/FOXO3/GNG10/GNG11/GNGT2/GRB2/GRK4/HCK/ITK/JAK2/JAK3/LYN/NCF1/NFKB1/NFKBIA/PIK3CD/PIK3CG/PIK3R5/PLCB2/PPBP/PRKCD/PTK2/RAC2/RAP1A/STAT2/VAV1/WAS/XCL1/XCL2 | 69 | KEGG |
| HALLMARK_MYC_TARGETS_V2 | 1.55E-12 | 5.12E-11 | BYSL/CDK4/DCTPP1/DDX18/MCM4/MCM5/MPHOSPH10/MRTO4/MYBBP1A/NOC4L/NOP56/PA2G4/PES1/PHB/PLK1/PLK4/PPAN/PPRC1/RABEPK/SLC29A2/SRM/TCOF1/TFB2M/TMEM97/UNG/WDR74 | 26 | HALLMARK |
| REACTOME_TOLL_LIKE_RECEPTOR_CASCADES | 8.56E-13 | 6.67E-11 | BIRC3/BTK/CASP8/CD14/CD180/CD36/CNPY3/CTSB/CTSK/CTSL/CTSS/DNM2/FBXW11/FOS/GSDMD/IKBKE/IRAK2/IRAK3/IRF7/ITGAM/ITGB2/JUN/LGMN/LY86/LY96/MAP2K3/MAP2K6/MAP3K8/MAPKAPK3/NFKB1/NFKB2/NFKBIA/NOD1/NOD2/PELI2/PIK3C3/PLCG2/PPP2R1A/PTPN11/RBSN/RIPK3/RPS6KA1/S100A12/S100A8/S100A9/SAA1/SOCS1/TICAM1/TICAM2/TLR1/TLR2/TLR3/TLR4/TLR5/TLR6/TLR7/TLR8/UBE2D3/UBE2N/UNC93B1 | 60 | REACTOME |
| KEGG_LYSOSOME | 9.55E-13 | 7.23E-11 | ACP2/ACP5/AP1B1/AP3S1/AP3S2/ARSA/ASAH1/ATP6V0B/ATP6V0D2/CD68/CTSA/CTSB/CTSC/CTSD/CTSH/CTSK/CTSL/CTSO/CTSS/CTSW/CTSZ/DNASE2/FUCA1/GAA/GALC/GBA/GGA2/GLB1/GM2A/GNS/HEXA/HEXB/LAMP1/LAMP3/LAPTM5/LGMN/LIPA/M6PR/MAN2B1/MANBA/MCOLN1/NAGA/NEU1/NPC2/PLA2G15/PPT1/PSAP/SLC11A1/SUMF1/TCIRG1/TPP1 | 51 | KEGG |
| WP_TYPE_II_INTERFERON_SIGNALING_IFNG | 1.00E-12 | 7.38E-11 | CIITA/CXCL10/CXCL9/CYBB/GBP1/HLA-B/ICAM1/IFIT2/IFNGR1/IFNGR2/IL1B/IRF1/IRF2/IRF8/IRF9/JAK2/OAS1/PRKCD/PSMB9/PTPN11/SOCS1/SOCS3/SPI1/STAT2/TAP1 | 25 | WP |
| KEGG_INTESTINAL_IMMUNE_NETWORK_FOR_IGA_PRODUCTION | 1.07E-12 | 7.70E-11 | CD28/CD40/CD40LG/CD80/CD86/CXCL12/CXCR4/HLA-DMA/HLA-DMB/HLA-DOA/HLA-DOB/HLA-DPA1/HLA-DPB1/HLA-DQA1/HLA-DQA2/HLA-DQB1/HLA-DRA/HLA-DRB1/ICOS/ICOSLG/IL10/IL15/IL15RA/IL6/ITGB7/LTBR/TGFB1/TNFSF13/TNFSF13B | 29 | KEGG |
| PID_AURORA_B_PATHWAY | 2.45E-12 | 7.97E-11 | AURKA/AURKB/BIRC5/BUB1/CDCA8/CENPA/INCENP/KIF20A/KIF23/KIF2C/NCAPD2/NCAPG/NCAPH/NCL/NDC80/PPP1CC/RACGAP1/SGO1/SMC2/SMC4/TACC1 | 21 | PID |
| REACTOME_LAGGING_STRAND_SYNTHESIS | 3.28E-12 | 1.05E-10 | DNA2/FEN1/LIG1/PCNA/POLA1/POLA2/POLD3/PRIM1/PRIM2/RFC2/RFC3/RFC4/RFC5/RPA1/RPA2 | 15 | REACTOME |
| KEGG_TYPE_I_DIABETES_MELLITUS | 1.74E-12 | 1.22E-10 | CD28/CD80/CD86/FAS/FASLG/GZMB/HLA-A/HLA-B/HLA-C/HLA-DMA/HLA-DMB/HLA-DOA/HLA-DOB/HLA-DPA1/HLA-DPB1/HLA-DQA1/HLA-DQA2/HLA-DQB1/HLA-DRA/HLA-DRB1/HLA-E/HLA-F/IL1A/IL1B/LTA/PRF1/TNF | 27 | KEGG |
| REACTOME_RESOLUTION_OF_D_LOOP_STRUCTURES_THROUGH_SYNTHESIS_DEPENDENT_STRAND_ANNEALING_SDSA | 3.91E-12 | 1.24E-10 | BARD1/BRCA1/BRCA2/BRIP1/DNA2/EXO1/MRE11/RAD51/RAD51AP1/RAD51D/RBBP8/RMI1/RMI2/RTEL1/TOP3A/WRN/XRCC2 | 17 | REACTOME |
| WP_DNA_MISMATCH_REPAIR | 4.00E-12 | 1.24E-10 | EXO1/LIG1/MLH1/MSH2/MSH6/PCNA/POLD3/POLE/POLE2/POLE3/RFC2/RFC3/RFC4/RFC5/RPA1/RPA2 | 16 | WP |
| REACTOME_INTERFERON_ALPHA_BETA_SIGNALING | 2.87E-12 | 1.96E-10 | BST2/EGR1/GBP2/HLA-A/HLA-B/HLA-C/HLA-E/HLA-F/IFI27/IFI35/IFIT2/IFIT3/IFITM1/IFITM2/IFITM3/IRF1/IRF2/IRF5/IRF7/IRF8/IRF9/ISG20/MX1/MX2/OAS1/OAS2/OASL/PSMB8/PTPN11/PTPN6/RSAD2/SAMHD1/SOCS1/SOCS3/STAT2/XAF1 | 36 | REACTOME |
| REACTOME_G2_M_DNA_DAMAGE_CHECKPOINT | 8.71E-12 | 2.67E-10 | ATRIP/BARD1/BRCA1/BRIP1/CCNB1/CDC25C/CDK1/CHEK1/CHEK2/DNA2/EXO1/H2AX/H2BC17/H2BC9/MRE11/NSD2/PIAS4/RBBP8/RFC2/RFC3/RFC4/RFC5/RHNO1/RMI1/RMI2/RNF168/RPA1/RPA2/TOP3A/TOPBP1/UIMC1/WRN/YWHAQ | 33 | REACTOME |
| REACTOME_METABOLISM_OF_AMINO_ACIDS_AND_DERIVATIVES | 3.31E-12 | 4.25E-10 | ACADSB/AHCY/AIMP1/AIMP2/ALDH4A1/ALDH6A1/AUH/BCKDK/CKMT1A/CKMT1B/CRYM/DBH/DIO3/FAU/GLS/GLS2/GLUD1/GLUD2/GOT1/GOT2/HIBADH/HSD17B10/NAALAD2/NAT8L/OAT/PDHA1/PDHX/PHYKPL/PNMT/PPM1K/PRODH/PSMA2/PSMA3/PSMA4/PSMA7/PSMB1/PSMB3/PSMB4/PSMC2/PSMC4/PSMD13/PSMD9/PSME1/PSME2/PSMF1/PSTK/QARS1/RIMKLA/RPL10/RPL10A/RPL11/RPL12/RPL13/RPL13A/RPL14/RPL17/RPL18/RPL18A/RPL19/RPL22L1/RPL23/RPL23A/RPL24/RPL26L1/RPL27/RPL27A/RPL28/RPL29/RPL30/RPL32/RPL35/RPL35A/RPL36/RPL36A/RPL39/RPL41/RPL5/RPL6/RPL7/RPL7A/RPL8/RPL9/RPLP0/RPLP1/RPLP2/RPS10/RPS11/RPS13/RPS14/RPS15/RPS15A/RPS16/RPS17/RPS18/RPS19/RPS2/RPS20/RPS21/RPS25/RPS27L/RPS28/RPS3/RPS3A/RPS4X/RPS5/RPS7/RPS8/RPS9/RPSA/SEM1/SERINC1/SERINC3/SLC25A12/SLC25A13/SLC6A12/UBA52 | 116 | REACTOME |
| WP_TCELL_RECEPTOR_TCR_SIGNALING_PATHWAY | 1.22E-11 | 8.03E-10 | CARD11/CCR5/CD247/CD28/CD3D/CD3E/CD3G/CD4/CD83/CD8A/CRKL/FAS/FOS/FYB1/FYN/GRAP2/GRB2/ICOS/IL15RA/IL1A/IL1B/IL6/ITK/JUN/LCK/LCP2/MAP3K8/MAP4K1/NFATC2/NFKB1/NFKBIA/PRKCD/PSTPIP1/PTPN11/REL/SKAP1/TGFB1/VAV1/VIM/WAS/ZAP70 | 41 | WP |
| WP_OVERVIEW_OF_PROINFLAMMATORY_AND_PROFIBROTIC_MEDIATORS | 1.24E-11 | 8.03E-10 | AREG/CCL18/CCL2/CCL20/CCL26/CCL3/CCL3L3/CCL4/CCL4L2/CCL5/CCL7/CCL8/CSF1/CSF3/CXCL1/CXCL10/CXCL11/CXCL12/CXCL13/CXCL16/CXCL2/CXCL3/CXCL5/CXCL6/CXCL8/CXCL9/EBI3/IL10/IL11/IL15/IL18/IL1A/IL1B/IL1RN/IL23A/IL6/IL7/LIF/LTA/MMP1/NFKB1/OSM/PPBP/SPP1/TGFB1/TNF/TNFSF13/TNFSF13B/TSLP/XCL1/XCL2 | 51 | WP |
| REACTOME_OPIOID_SIGNALLING | 7.08E-12 | 8.68E-10 | ADCY1/ADCY2/ADCY5/AHCYL1/CALM1/CAMK2A/CAMK2B/CAMK2G/CAMK4/CAMKK1/CAMKK2/GNAI1/GNAI2/GNAI3/GNAL/GNAQ/GNB1/GNB2/GNB5/GNG12/GNG3/GNG5/ITPR1/MAPK1/NBEA/PDE1A/PDE1B/PDE4A/PDYN/PLCB1/PLCB3/PLCB4/PPP1CA/PPP1R1B/PPP2CA/PPP3CA/PPP3CB/PPP3R1/PRKACB/PRKAR1A/PRKAR1B/PRKAR2B/PRKCG | 43 | REACTOME |
| WP_SARSCOV2_INNATE_IMMUNITY_EVASION_AND_CELLSPECIFIC_IMMUNE_RESPONSE | 1.92E-11 | 1.22E-09 | CASP8/CCL2/CCL3/CCL4/CCL5/CXCL1/CXCL10/CXCL11/CXCL12/CXCL13/CXCL2/CXCL3/CXCL5/CXCL6/CXCL8/CXCL9/CXCR2/DHX58/HAVCR2/IFIT2/IFITM1/IL10/IL6/IL6R/IRF7/LAG3/MX1/NFKB1/PPBP/STAT2/TGFB1/TLR7/TNF/TRADD | 34 | WP |
| REACTOME_REGULATION_OF_INSULIN_SECRETION | 1.13E-11 | 1.32E-09 | ABCC8/ADCY5/ADRA2A/ADRA2C/AHCYL1/AKAP5/CACNA1A/CACNA1C/CACNA1D/CACNA1E/CACNA2D2/CACNB2/CACNB3/CHRM3/GNAI1/GNAI2/GNAQ/GNB1/GNB2/GNB5/GNG12/GNG3/GNG5/ITPR1/KCNB1/KCNJ11/KCNS3/PLCB1/PLCB3/PRKACB/PRKAR1A/PRKAR1B/PRKAR2B/RAPGEF4/SNAP25/STX1A/STXBP1/SYT5/VAMP2 | 39 | REACTOME |
| BIOCARTA_CTL_PATHWAY | 2.40E-11 | 1.49E-09 | B2M/CD247/CD3D/CD3E/CD3G/FAS/FASLG/GZMB/HLA-A/ICAM1/ITGAL/ITGB2/PRF1 | 13 | BIOCARTA |
| PID_BARD1_PATHWAY | 4.95E-11 | 1.50E-09 | BARD1/BRCA1/CCNE1/CDK2/CSTF1/FANCA/FANCC/FANCD2/FANCE/FANCG/MRE11/PCNA/RAD51/RBBP8/TOPBP1/XRCC5/XRCC6 | 17 | PID |
| WP_SYNAPTIC_VESICLE_PATHWAY | 2.46E-11 | 2.76E-09 | AP2A1/AP2A2/AP2M1/ATP1A2/CACNA1A/CPLX1/CPLX2/CPLX3/DNM1/DNM1L/DNM3/NSF/RAB3A/RIMS1/SLC17A7/SLC1A3/SLC22A3/SLC25A4/SLC32A1/SNAP25/STX1A/STX1B/STXBP1/SYN1/SYN2/SYN3/SYP/SYT1/UNC13A/VAMP2 | 30 | WP |
| KEGG_TOLL_LIKE_RECEPTOR_SIGNALING_PATHWAY | 5.71E-11 | 3.46E-09 | AKT3/CASP8/CCL3/CCL4/CCL5/CD14/CD40/CD80/CD86/CTSK/CXCL10/CXCL11/CXCL8/CXCL9/FOS/IKBKE/IL1B/IL6/IRF5/IRF7/JUN/LY96/MAP2K3/MAP2K6/MAP3K8/MAPK13/NFKB1/NFKBIA/PIK3CD/PIK3CG/PIK3R5/SPP1/TICAM1/TICAM2/TLR1/TLR2/TLR3/TLR4/TLR5/TLR6/TLR7/TLR8/TNF | 43 | KEGG |
| REACTOME_TELOMERE_C_STRAND_LAGGING_STRAND_SYNTHESIS | 1.34E-10 | 3.95E-09 | CHTF18/DNA2/DSCC1/FEN1/LIG1/PCNA/POLA1/POLA2/POLD3/PRIM1/PRIM2/RFC2/RFC3/RFC4/RFC5/RPA1/RPA2/WRN | 18 | REACTOME |
| REACTOME_TRANSCRIPTIONAL_REGULATION_BY_E2F6 | 1.34E-10 | 3.95E-09 | BRCA1/CDC7/CHEK1/E2F1/E2F6/EHMT1/EZH2/PCGF2/PCGF6/RAD51/RBBP4/RBBP7/RBBP8/RING1/RNF2/RRM2/SUZ12/TFDP1 | 18 | REACTOME |
| REACTOME_RESPIRATORY_ELECTRON_TRANSPORT_ATP_SYNTHESIS_BY_CHEMIOSMOTIC_COUPLING_AND_HEAT_PRODUCTION_BY_UNCOUPLING_PROTEINS | 1.58E-12 | 3.95E-09 | ATP5F1D/ATP5F1E/ATP5MC1/ATP5MC2/ATP5MC3/ATP5ME/ATP5MF/ATP5MG/ATP5PD/COX11/COX14/COX18/COX4I1/COX5B/COX6B1/COX6C/COX7B/COX7C/COX8A/ETFB/LRPPRC/NDUFA1/NDUFA11/NDUFA13/NDUFA2/NDUFA3/NDUFA7/NDUFA8/NDUFAF3/NDUFB1/NDUFB10/NDUFB11/NDUFB2/NDUFB3/NDUFB4/NDUFB7/NDUFS5/NDUFS7/NDUFS8/NDUFV1/SCO1/SURF1/UQCR10/UQCR11/UQCRH/UQCRQ | 46 | REACTOME |
| REACTOME_PROTEIN_PROTEIN_INTERACTIONS_AT_SYNAPSES | 3.77E-11 | 4.07E-09 | APBA1/APBA3/BEGAIN/DBNL/DLG2/DLG3/DLG4/DLGAP1/DLGAP3/EPB41L1/EPB41L3/GRIN1/GRIN2A/GRIN2B/GRIN2C/GRM1/GRM5/HOMER1/IL1RAP/IL1RAPL1/LIN7B/LRFN1/LRFN2/LRRTM4/NRXN1/NRXN3/PPFIA2/PPFIA3/PPFIA4/RTN3/SHANK1/SHANK2/SHARPIN/SLITRK1/SLITRK4/SLITRK5/STX1A/STXBP1/SYT1/SYT12/SYT7 | 41 | REACTOME |
| REACTOME_COSTIMULATION_BY_THE_CD28_FAMILY | 7.21E-11 | 4.28E-09 | AKT3/CD247/CD274/CD28/CD3D/CD3E/CD3G/CD4/CD80/CD86/CSK/CTLA4/FYN/GRAP2/GRB2/HLA-DPA1/HLA-DPB1/HLA-DQA1/HLA-DQA2/HLA-DQB1/HLA-DRA/HLA-DRB1/ICOS/ICOSLG/LCK/LYN/MAP3K8/PDCD1/PDCD1LG2/PPP2R1A/PPP2R5C/PTPN11/PTPN6/TNFRSF14/VAV1 | 35 | REACTOME |
| REACTOME_G0_AND_EARLY_G1 | 1.48E-10 | 4.29E-09 | CCNA2/CCNE1/CCNE2/CDC25A/CDC6/CDK1/CDK2/E2F1/E2F5/LIN9/MYBL2/PCNA/RBBP4/RBL1/TFDP1/TOP2A | 16 | REACTOME |
| REACTOME_RESOLUTION_OF_ABASIC_SITES_AP_SITES | 1.50E-10 | 4.30E-09 | APEX1/FEN1/LIG1/PARP1/PCNA/POLD3/POLE/POLE2/POLE3/RFC2/RFC3/RFC4/RFC5/RPA1/RPA2/SMUG1/TDG/UNG/XRCC1 | 19 | REACTOME |
| REACTOME_PCNA_DEPENDENT_LONG_PATCH_BASE_EXCISION_REPAIR | 2.23E-10 | 6.30E-09 | APEX1/FEN1/LIG1/PCNA/POLD3/POLE/POLE2/POLE3/RFC2/RFC3/RFC4/RFC5/RPA1/RPA2 | 14 | REACTOME |
| REACTOME_TRANSPORT_OF_MATURE_MRNAS_DERIVED_FROM_INTRONLESS_TRANSCRIPTS | 2.60E-10 | 7.26E-09 | ALYREF/CPSF3/CPSF4/FIP1L1/NDC1/NUP107/NUP155/NUP160/NUP188/NUP205/NUP210/NUP35/NUP42/NUP43/NUP50/NUP85/NUP88/RAE1/SLBP/WDR33 | 20 | REACTOME |
| REACTOME_FANCONI_ANEMIA_PATHWAY | 2.68E-10 | 7.37E-09 | ATRIP/CENPX/DCLRE1B/EME1/FAAP100/FAAP24/FANCA/FANCB/FANCC/FANCD2/FANCE/FANCG/FANCI/FANCM/MUS81/RPA1/RPA2/UBE2T/USP1 | 19 | REACTOME |
| REACTOME_MITOTIC_PROPHASE | 3.10E-10 | 8.42E-09 | CCNB1/CCNB2/CDK1/GORASP2/H2AX/H2AZ1/H2BC17/H2BC9/H3-3A/H3C2/H3C3/KMT5A/LMNB1/LPIN3/MASTL/MCPH1/NCAPD3/NCAPG2/NCAPH2/NDC1/NUP107/NUP155/NUP160/NUP188/NUP205/NUP210/NUP35/NUP42/NUP43/NUP50/NUP85/NUP88/PLK1/RAE1/SET/SMC2/SMC4/TMPO/VRK1 | 39 | REACTOME |
| KEGG_NOD_LIKE_RECEPTOR_SIGNALING_PATHWAY | 1.48E-10 | 8.60E-09 | BIRC3/CARD6/CARD9/CASP1/CASP5/CASP8/CCL2/CCL5/CCL7/CCL8/CXCL1/CXCL2/CXCL8/IL18/IL1B/IL6/MAPK13/MEFV/NAIP/NFKB1/NFKBIA/NLRC4/NLRP1/NLRP3/NOD1/NOD2/PSTPIP1/PYCARD/SUGT1/TNF/TNFAIP3 | 31 | KEGG |
| REACTOME_NEUROTRANSMITTER_RELEASE_CYCLE | 8.62E-11 | 8.93E-09 | APBA1/CPLX1/DNAJC5/GAD2/GLS/GLS2/LIN7B/MAOA/PPFIA2/PPFIA3/PPFIA4/RAB3A/RIMS1/SLC17A7/SLC1A2/SLC1A3/SLC32A1/SLC6A1/SLC6A12/SLC6A13/SNAP25/STX1A/STXBP1/SYN1/SYN2/SYN3/SYT1/TSPOAP1/VAMP2 | 29 | REACTOME |
| REACTOME_NUCLEAR_ENVELOPE_NE_REASSEMBLY | 3.80E-10 | 1.02E-08 | ANKLE2/CCNB1/CCNB2/CDK1/CHMP6/CHMP7/IST1/KPNB1/LBR/LMNB1/NDC1/NUP107/NUP155/NUP160/NUP188/NUP205/NUP35/NUP43/NUP85/PPP2R2A/RAN/RCC1/TMPO/TUBA1A/TUBA1B/UBE2I/VRK1 | 27 | REACTOME |
| REACTOME_DNA_DAMAGE_BYPASS | 3.84E-10 | 1.02E-08 | DTL/MAD2L2/NPLOC4/PCLAF/PCNA/POLD3/POLE/POLE2/POLE3/POLH/RAD18/RFC2/RFC3/RFC4/RFC5/RPA1/RPA2/SPRTN/UFD1/USP1/VCP | 21 | REACTOME |
| REACTOME_RESOLUTION_OF_AP_SITES_VIA_THE_MULTIPLE_NUCLEOTIDE_PATCH_REPLACEMENT_PATHWAY | 4.41E-10 | 1.15E-08 | APEX1/FEN1/LIG1/PARP1/PCNA/POLD3/POLE/POLE2/POLE3/RFC2/RFC3/RFC4/RFC5/RPA1/RPA2 | 15 | REACTOME |
| REACTOME_METABOLISM_OF_RNA | 1.22E-10 | 1.21E-08 | ADARB1/APOBEC3B/BUD23/BUD31/CLP1/CNOT11/CTNNBL1/CWC25/DDX39A/DDX47/DHX37/DNAJC8/EIF4A1/EIF4A2/ELAVL2/EMG1/ERI1/EXOSC4/EXOSC7/FAU/FBL/GAR1/GEMIN7/GNL3/GTF2H5/HNRNPF/HSD17B10/ISG20L2/ISY1/LSM1/LSM10/LSM11/LSM2/LSM5/LSM6/LSM7/MAGOH/MAPKAPK2/MRM2/NIP7/NOP2/NSUN2/NUP37/NUP54/NUP62/NXT1/PABPC1/PCBP1/PCBP2/PHF5A/PNO1/POLR2G/POLR2H/POLR2J/POP5/PPIE/PPIL3/PPP2CA/PRPF19/PSMA2/PSMA3/PSMA4/PSMA7/PSMB1/PSMB3/PSMB4/PSMC2/PSMC4/PSMD13/PSMD9/PSME1/PSME2/PSMF1/PTBP1/QTRT2/RBM22/RPL10/RPL10A/RPL11/RPL12/RPL13/RPL13A/RPL14/RPL17/RPL18/RPL18A/RPL19/RPL22L1/RPL23/RPL23A/RPL24/RPL26L1/RPL27/RPL27A/RPL28/RPL29/RPL30/RPL32/RPL35/RPL35A/RPL36/RPL36A/RPL39/RPL41/RPL5/RPL6/RPL7/RPL7A/RPL8/RPL9/RPLP0/RPLP1/RPLP2/RPS10/RPS11/RPS13/RPS14/RPS15/RPS15A/RPS16/RPS17/RPS18/RPS19/RPS2/RPS20/RPS21/RPS25/RPS27L/RPS28/RPS3/RPS3A/RPS4X/RPS5/RPS7/RPS8/RPS9/RPSA/RRP36/RRP9/SARNP/SEC13/SEM1/SF3B5/SF3B6/SKIV2L/SLU7/SMN1/SNRPB/SNRPB2/SNRPC/SNRPD2/SNRPE/SNRPF/SNRPG/SNRPN/SNU13/SNW1/SRSF9/SYF2/THADA/THG1L/THOC6/TPRKB/TRMT112/TRMT6/TRMT61A/TSEN34/UBA52/URM1/UTP15/UTP6/WBP4/WDR46/YWHAB/YWHAZ/ZBTB8OS | 176 | REACTOME |
| KEGG_LONG_TERM_POTENTIATION | 1.28E-10 | 1.23E-08 | ADCY1/ARAF/ATF4/CACNA1C/CALM1/CALM2/CALM3/CAMK2A/CAMK2B/CAMK2G/CAMK4/GNAQ/GRIN1/GRIN2A/GRIN2B/GRIN2C/GRM1/GRM5/ITPR1/MAP2K1/MAPK1/MAPK3/PLCB1/PLCB3/PLCB4/PPP1CA/PPP1CB/PPP1R1A/PPP3CA/PPP3CB/PPP3R1/PRKACB/PRKCB/PRKCG/RAP1B | 35 | KEGG |
| REACTOME_NUCLEAR_ENVELOPE_BREAKDOWN | 5.06E-10 | 1.31E-08 | CCNB1/CCNB2/CDK1/LMNB1/LPIN3/NDC1/NUP107/NUP155/NUP160/NUP188/NUP205/NUP210/NUP35/NUP42/NUP43/NUP50/NUP85/NUP88/PLK1/RAE1/TMPO/VRK1 | 22 | REACTOME |
| REACTOME_INTERACTIONS_OF_REV_WITH_HOST_CELLULAR_PROTEINS | 8.19E-10 | 2.09E-08 | KPNB1/NDC1/NUP107/NUP155/NUP160/NUP188/NUP205/NUP210/NUP35/NUP42/NUP43/NUP50/NUP85/NUP88/RAE1/RAN/RANBP1/RCC1 | 18 | REACTOME |
| REACTOME_TRNA_PROCESSING_IN_THE_NUCLEUS | 9.13E-10 | 2.31E-08 | CPSF4/CSTF2/ELAC2/NDC1/NUP107/NUP155/NUP160/NUP188/NUP205/NUP210/NUP35/NUP42/NUP43/NUP50/NUP85/NUP88/POP1/RAE1/RAN/RPP38/RPP40/TRNT1/TSEN15 | 23 | REACTOME |
| REACTOME_HIV_LIFE_CYCLE | 9.36E-10 | 2.32E-08 | CHMP6/CHMP7/ELOA/FEN1/GTF2E1/GTF2F2/GTF2H4/HMGA1/LIG1/NDC1/NELFB/NELFCD/NUP107/NUP155/NUP160/NUP188/NUP205/NUP210/NUP35/NUP42/NUP43/NUP50/NUP85/NUP88/POLR2D/RAE1/RAN/RANBP1/RCC1/SSRP1/SUPT16H/TAF11/TAF15/TAF4/TAF6/TBP/VPS37B/XRCC4/XRCC5/XRCC6 | 40 | REACTOME |
| REACTOME_TRNA_PROCESSING | 9.41E-10 | 2.32E-08 | CDKAL1/CPSF4/CSTF2/CTU2/DUS2/ELAC2/FTSJ1/NDC1/NUP107/NUP155/NUP160/NUP188/NUP205/NUP210/NUP35/NUP42/NUP43/NUP50/NUP85/NUP88/POP1/PUS3/RAE1/RAN/RPP38/RPP40/TRMT10C/TRMT5/TRMT61B/TRNT1/TSEN15/TYW3/WDR4 | 33 | REACTOME |
| WP_EBOLA_VIRUS_INFECTION_IN_HOST | 4.29E-10 | 2.44E-08 | ACTB/ACTN1/AXL/BST2/CD209/CD300A/CLEC10A/CTSB/CTSL/FLNB/FLNC/GAS6/GSN/HAVCR2/HLA-A/HLA-B/HLA-C/HLA-DMA/HLA-DMB/HLA-DOA/HLA-DOB/HLA-DPA1/HLA-DPB1/HLA-DQA1/HLA-DQA2/HLA-DQB1/HLA-DRA/HLA-DRB1/HLA-E/HLA-F/ICAM3/IKBKE/IQGAP1/IRF7/ITGA2/MERTK/NFKB1/NFKB2/NPC2/PIK3CD/REL/RELB/SCIN/SOCS3/TIMD4/TLR4/VPS18/VPS39/VPS4A | 49 | WP |
| WP_IL18_SIGNALING_PATHWAY | 4.71E-10 | 2.62E-08 | AARS1/APBA2/ATF3/B2M/BIRC3/BTG2/CASP8/CCL18/CCL2/CCL20/CCL3/CCL4/CCL5/CD36/CD81/CD83/CEBPB/CFLAR/CLDN1/CLDN4/CXCL16/CXCL2/CXCL3/CXCL8/FAS/FASLG/FOS/GRN/HCAR2/HMOX1/ICAM1/IER3/IL10/IL18/IL18BP/IL18R1/IL18RAP/IL1B/IL2RA/IL6/IRF1/ITM2C/JUN/KLF2/LCK/LRRFIP1/LTB/MMP1/MYH7/NCF1/NCF2/NFKB1/NFKB2/NFKBIA/NFKBIE/NFKBIZ/NR1H3/PIGT/PRKCA/PRKCD/PTGS2/PTPN7/PTX3/PWWP3A/REL/RGS16/RUNX2/S1PR4/SEMA6D/SOCS3/SPON1/SPP1/TGM2/TICAM2/TIMP1/TMSB4X/TNF/TNFAIP2/TNFAIP3/TNFRSF11B/TNFRSF1A/TRAF1/ZC3H12A | 83 | WP |
| WP_TOLLLIKE_RECEPTOR_SIGNALING_PATHWAY | 4.82E-10 | 2.63E-08 | AKT3/CASP8/CCL3/CCL4/CCL5/CD14/CD40/CD80/CD86/CXCL10/CXCL11/CXCL8/CXCL9/FOS/IKBKE/IL1B/IL6/IRF5/IRF7/JUN/LY96/MAP2K3/MAP2K6/MAP3K8/MAPK13/NFKB1/NFKBIA/PIK3CD/PIK3CG/PIK3R5/SPP1/TICAM1/TICAM2/TLR1/TLR2/TLR3/TLR4/TLR5/TLR6/TLR7/TLR8/TNF | 42 | WP |
| REACTOME_PD_1_SIGNALING | 5.03E-10 | 2.68E-08 | CD247/CD274/CD3D/CD3E/CD3G/CD4/CSK/HLA-DPA1/HLA-DPB1/HLA-DQA1/HLA-DQA2/HLA-DQB1/HLA-DRA/HLA-DRB1/LCK/PDCD1/PDCD1LG2/PTPN11/PTPN6 | 19 | REACTOME |
| WP_CANCER_IMMUNOTHERAPY_BY_PD1_BLOCKADE | 5.11E-10 | 2.68E-08 | BATF/CD274/CD3D/CD3E/CD3G/CD8A/CD8B/HLA-A/HLA-DRB1/JUN/LCK/NFATC2/NFKB1/PDCD1/PDCD1LG2/PTPN11/ZAP70 | 17 | WP |
| REACTOME_NEUREXINS_AND_NEUROLIGINS | 3.02E-10 | 2.80E-08 | APBA1/APBA3/BEGAIN/DBNL/DLG2/DLG3/DLG4/DLGAP1/DLGAP3/EPB41L1/EPB41L3/GRIN1/GRIN2A/GRIN2B/GRIN2C/GRM1/GRM5/HOMER1/LIN7B/LRRTM4/NRXN1/NRXN3/SHANK1/SHANK2/SHARPIN/STX1A/STXBP1/SYT1/SYT12/SYT7 | 30 | REACTOME |
| WP_CHEMOKINE_SIGNALING_PATHWAY | 5.94E-10 | 3.06E-08 | ADCY7/AKT3/ARRB2/CCL20/CCL26/CCL3/CCL4/CCL5/CCL7/CCR1/CCR2/CCR4/CCR6/CCR7/CRKL/CSK/CXCL10/CXCL11/CXCL12/CXCL13/CXCL16/CXCL3/CXCL5/CXCL9/CXCR2/CXCR3/CXCR4/CXCR6/DOCK2/FGR/FOXO3/GNG10/GNG11/GNGT2/GRB2/GRK4/HCK/ITK/JAK2/JAK3/LYN/NCF1/NFKB1/NFKBIA/PIK3CD/PIK3CG/PIK3R5/PLCB2/PPBP/PRKCD/PTK2/RAC2/RAP1A/STAT2/VAV1/WAS/XCL1 | 57 | WP |
| REACTOME_NUCLEAR_IMPORT_OF_REV_PROTEIN | 1.41E-09 | 3.45E-08 | KPNB1/NDC1/NUP107/NUP155/NUP160/NUP188/NUP205/NUP210/NUP35/NUP42/NUP43/NUP50/NUP85/NUP88/RAE1/RAN/RCC1 | 17 | REACTOME |
| KEGG_AUTOIMMUNE_THYROID_DISEASE | 7.64E-10 | 3.86E-08 | CD28/CD40/CD40LG/CD80/CD86/CTLA4/FAS/FASLG/GZMB/HLA-A/HLA-B/HLA-C/HLA-DMA/HLA-DMB/HLA-DOA/HLA-DOB/HLA-DPA1/HLA-DPB1/HLA-DQA1/HLA-DQA2/HLA-DQB1/HLA-DRA/HLA-DRB1/HLA-E/HLA-F/IL10/PRF1 | 27 | KEGG |
| REACTOME_SWITCHING_OF_ORIGINS_TO_A_POST_REPLICATIVE_STATE | 1.63E-09 | 3.92E-08 | CCNA2/CCNE1/CCNE2/CDC23/CDC6/CDK2/CDT1/FZR1/MCM2/MCM3/MCM4/MCM5/MCM6/MCM7/MCM8/ORC1/ORC6/PSMB2/PSMB7/PSMD11/PSMD14/PSMD3/PSMD5/PSMD6/PSME3/SKP1/SKP2/UBE2C/UBE2S | 29 | REACTOME |
| REACTOME_PURINERGIC_SIGNALING_IN_LEISHMANIASIS_INFECTION | 8.78E-10 | 4.36E-08 | C3/C3AR1/CASP1/GSDMD/HMOX1/IL18/IL1A/IL1B/MEFV/NFKB1/NFKB2/NLRP3/P2RX4/PSTPIP1/PYCARD/SUGT1/TXN/TXNIP | 18 | REACTOME |
| WP_BURN_WOUND_HEALING | 1.01E-09 | 4.93E-08 | CCL2/CD3E/CNN2/CXCL1/CXCL12/CXCL8/CXCR2/CXCR4/DCN/EGF/F13A1/HGF/ICAM1/IL15/IL1A/IL1B/IL6/INHBA/KLF4/LY96/MMP1/NFKB1/NFKBIA/NFKBIZ/NOD1/NOD2/S100A11/S100A6/S100A9/SFRP2/TGFB1/TIMP1/TLR1/TLR2/TLR3/TLR4/TLR5/TLR6/TLR7/TLR8/TNF/TNFAIP3/VIM | 43 | WP |
| REACTOME_G_PROTEIN_MEDIATED_EVENTS | 5.52E-10 | 4.96E-08 | ADCY1/ADCY2/ADCY5/AHCYL1/CALM1/CAMK2A/CAMK2B/CAMK2G/CAMK4/CAMKK1/CAMKK2/GNAI1/GNAI2/GNAI3/GNAL/GNAQ/ITPR1/MAPK1/NBEA/PDE1A/PDE1B/PLCB1/PLCB3/PLCB4/PRKACB/PRKAR1A/PRKAR1B/PRKAR2B/PRKCG | 29 | REACTOME |
| REACTOME_DEPOSITION_OF_NEW_CENPA_CONTAINING_NUCLEOSOMES_AT_THE_CENTROMERE | 2.10E-09 | 5.02E-08 | CENPA/CENPH/CENPI/CENPK/CENPL/CENPM/CENPN/CENPO/CENPP/CENPQ/CENPU/CENPW/CENPX/H2AX/H2AZ1/H2BC17/H2BC9/HJURP/KNL1/MIS18A/MIS18BP1/OIP5/RBBP4/RBBP7/RUVBL1 | 25 | REACTOME |
| REACTOME_HEMOSTASIS | 1.11E-09 | 5.30E-08 | A2M/ACTB/ACTN1/ANXA2/APBB1IP/ARRB2/ATP1B3/ATP2A3/CAPZA1/CAPZB/CD109/CD2/CD244/CD36/CD44/CD48/CD74/CD84/CD99/CEACAM1/CFD/CSK/CTSW/CYB5R1/CYRIB/DAGLA/DOCK11/DOCK2/DOCK8/DOK2/EGF/EHD2/F11R/F13A1/F5/FCER1G/FERMT3/FGR/FYN/GAS6/GATA6/GNA15/GNAS/GNG10/GNG11/GNGT2/GRB2/GUCY1A2/GYPC/HGF/IGF1/IGHA1/IGHA2/IGHM/IGKV1-5/IGKV1D-39/IGKV3-11/IGKV3-15/IGKV3-20/IGKV4-1/IGLC2/IGLC3/IGLV1-40/IGLV1-44/IGLV2-14/IGLV2-8/INPP5D/IRF1/IRF2/ISLR/ITGA2/ITGAL/ITGAM/ITGAX/ITGB2/JAK2/JAML/JCHAIN/KCNMB1/KCNMB2/KIF19/KIF1B/KIF21A/KIF2A/KLC4/LCK/LCP2/LHFPL2/LRP8/LYN/MAFK/MAGED2/MERTK/MMP1/NFE2/OLR1/P2RX1/P2RX4/PDE9A/PDPN/PIK3CG/PIK3R5/PIK3R6/PLAU/PLAUR/PLCG2/PLEK/PPBP/PPP2R1A/PPP2R5C/PRKCA/PRKCD/PRKCH/PROCR/PROS1/PSAP/PTK2/PTPN11/PTPN6/RAC2/RAP1A/RARRES2/RBSN/RHOG/S100A10/SCG3/SDC2/SDC3/SELE/SELL/SELPLG/SERPINA1/SERPINA3/SERPINA5/SERPINE1/SERPINF2/SERPING1/SIRPG/SLC16A3/SLC7A7/SLC7A8/SPN/SRGN/STX4/STXBP2/SYK/TAGLN2/TGFB1/THBD/THBS1/TIMP1/TLN1/TMSB4X/TNFRSF10A/TNFRSF10D/TOR4A/TREM1/TSPAN7/TUBA1C/TUBB2B/VAV1/VEGFC | 162 | REACTOME |
| REACTOME_SUMOYLATION_OF_UBIQUITINYLATION_PROTEINS | 2.41E-09 | 5.62E-08 | NDC1/NUP107/NUP155/NUP160/NUP188/NUP205/NUP210/NUP35/NUP42/NUP43/NUP50/NUP85/NUP88/PIAS4/RAE1/TRIM27/UBE2I/VHL | 18 | REACTOME |
| REACTOME_TRANSLESION_SYNTHESIS_BY_Y_FAMILY_DNA_POLYMERASES_BYPASSES_LESIONS_ON_DNA_TEMPLATE | 2.41E-09 | 5.62E-08 | MAD2L2/NPLOC4/PCLAF/PCNA/POLD3/POLE/POLE2/POLE3/POLH/RFC2/RFC3/RFC4/RFC5/RPA1/RPA2/SPRTN/UFD1/VCP | 18 | REACTOME |
| WP_ELECTRON_TRANSPORT_CHAIN_OXPHOS_SYSTEM_IN_MITOCHONDRIA | 4.52E-11 | 5.64E-08 | ATP5F1D/ATP5F1E/ATP5MC1/ATP5MC2/ATP5MC3/ATP5ME/ATP5MF/ATP5MG/ATP5PD/COX11/COX17/COX4I1/COX5B/COX6B1/COX6C/COX7B/COX7C/COX8A/NDUFA1/NDUFA2/NDUFA3/NDUFA7/NDUFA8/NDUFB1/NDUFB10/NDUFB2/NDUFB3/NDUFB4/NDUFB7/NDUFS5/NDUFS7/NDUFS8/NDUFV1/SCO1/SURF1/UQCR10/UQCR11/UQCRH/UQCRQ | 39 | WP |
| WP_COMPLEMENT_SYSTEM | 1.22E-09 | 5.75E-08 | ARRB2/C1R/C1S/C2/C3/C3AR1/C5AR1/C5AR2/C7/CD40/CD55/CFB/CFD/CFH/CFI/CR1/DCN/F13A1/FCGR3A/FCN1/FPR1/GNA15/ICAM1/ITGA2/ITGAX/ITGB2/PLAUR/PRKCA/PROS1/PTX3/SELE/SELL/SELPLG/SERPING1/SPP1/THBS1/TLR2/TXN/VSIG4/WAS | 40 | WP |
| REACTOME_SUMOYLATION_OF_SUMOYLATION_PROTEINS | 2.51E-09 | 5.79E-08 | NDC1/NUP107/NUP155/NUP160/NUP188/NUP205/NUP210/NUP35/NUP42/NUP43/NUP50/NUP85/NUP88/PIAS4/RAE1/SUMO2/UBE2I | 17 | REACTOME |
| REACTOME_INTEGRATION_OF_ENERGY_METABOLISM | 6.83E-10 | 5.94E-08 | ABCC8/ADCY1/ADCY2/ADCY5/ADRA2A/ADRA2C/AHCYL1/AKAP5/CACNA1A/CACNA1C/CACNA1D/CACNA1E/CACNA2D2/CACNB2/CACNB3/CHRM3/GNAI1/GNAI2/GNAQ/GNB1/GNB2/GNB5/GNG12/GNG3/GNG5/ITPR1/KCNB1/KCNJ11/KCNS3/MLX/PLCB1/PLCB3/PPP2CA/PRKAB2/PRKACB/PRKAG2/PRKAR1A/PRKAR1B/PRKAR2B/RAPGEF4/SNAP25/STX1A/STXBP1/SYT5/VAMP2 | 45 | REACTOME |
| PID_CD8_TCR_PATHWAY | 1.33E-09 | 6.03E-08 | B2M/CARD11/CD247/CD28/CD3D/CD3E/CD3G/CD80/CD86/CD8A/CD8B/CSK/FYN/GRAP2/GRB2/HLA-A/LCK/LCP2/MAP3K8/PRF1/PRKCA/PTPN6/PTPRC/RAP1A/RASSF5/VAV1/ZAP70 | 27 | PID |
| REACTOME_CELL_SURFACE_INTERACTIONS_AT_THE_VASCULAR_WALL | 1.33E-09 | 6.03E-08 | ATP1B3/CD2/CD244/CD44/CD48/CD74/CD84/CD99/CEACAM1/DOK2/F11R/FCER1G/FYN/GAS6/GRB2/GYPC/IGHA1/IGHA2/IGHM/IGKV1-5/IGKV1D-39/IGKV3-11/IGKV3-15/IGKV3-20/IGKV4-1/IGLC2/IGLC3/IGLV1-40/IGLV1-44/IGLV2-14/IGLV2-8/INPP5D/ITGAL/ITGAM/ITGAX/ITGB2/JAML/JCHAIN/LCK/LYN/MERTK/MMP1/OLR1/PROCR/PROS1/PTPN11/PTPN6/SDC2/SDC3/SELE/SELL/SELPLG/SIRPG/SLC16A3/SLC7A7/SLC7A8/SPN/TGFB1/THBD/TNFRSF10A/TNFRSF10D/TREM1/TSPAN7 | 63 | REACTOME |
| REACTOME_LEISHMANIA_INFECTION | 1.35E-09 | 6.03E-08 | ABI2/ACTB/ADCY7/ADCYAP1R1/ADRB2/ARPC1B/ARPC2/ARPC3/ARPC4/ARPC5/BRK1/BTK/C3/C3AR1/CASP1/CD163/CD247/CD3G/CYBA/CYFIP1/CYSLTR1/DPEP2/ELMO2/FCGR1A/FCGR2A/FCGR3A/FGR/FYN/FZD7/GGT5/GNAS/GNG10/GNG11/GNGT2/GPBAR1/GPR84/GRB2/GSDMD/HCK/HMOX1/HTR7/IGHG1/IGHG2/IGHG4/IGKV1-5/IGKV1D-39/IGKV3-11/IGKV3-15/IGKV3-20/IGKV4-1/IGLC2/IGLC3/IGLV1-40/IGLV1-44/IGLV2-14/IGLV2-8/IL10/IL18/IL1A/IL1B/IL6/JUN/LHB/LYN/MEFV/MYO10/NCKAP1L/NFKB1/NFKB2/NLRP3/P2RX4/PLCG2/PSTPIP1/PTGER2/PTGER4/PTK2/PYCARD/RAMP3/RHBDF2/SUGT1/SYK/TXN/TXNIP/VAV1/WAS/WASF2/WIPF1/WNT5A | 88 | REACTOME |
| KEGG_VIRAL_MYOCARDITIS | 1.38E-09 | 6.09E-08 | ACTB/CASP8/CD28/CD40/CD40LG/CD55/CD80/CD86/DMD/FYN/HLA-A/HLA-B/HLA-C/HLA-DMA/HLA-DMB/HLA-DOA/HLA-DOB/HLA-DPA1/HLA-DPB1/HLA-DQA1/HLA-DQA2/HLA-DQB1/HLA-DRA/HLA-DRB1/HLA-E/HLA-F/ICAM1/ITGAL/ITGB2/MYH7/PRF1/RAC2 | 32 | KEGG |
| REACTOME_INFECTIOUS_DISEASE | 8.29E-10 | 6.98E-08 | ABI1/ACTG1/ADCY1/ADCY2/ADCY5/ADCYAP1/ADRB1/AHCYL1/AP1S2/AP2A1/AP2A2/AP2M1/ARPC1A/ATP1A2/ATP1A3/ATP1B1/ATP6V1H/BANF1/BRMS1/BTRC/CALM1/CALR/CCNK/CD9/CHD3/CRBN/CRHR1/CTDP1/CTNND1/CYFIP2/DAD1/DAXX/DDOST/DRD1/DVL1/DYNC1H1/DYNC1I1/EED/ELK1/ELMO1/EPS15/FAU/FXYD7/GANAB/GATAD2A/GNAI1/GNAI2/GNAI3/GNAZ/GNB1/GNB2/GNB5/GNG12/GNG3/GNG5/GPR150/GPR176/GPR27/GSK3A/GTF2A2/GTF2B/GTF2E2/GTF2H5/H2AC20/H2AC8/H3C10/H4C11/HDAC1/HMG20B/HRH2/HSP90AB1/IMPDH2/ITPR1/KEAP1/LIG4/MAN1B1/MAP2K1/MAP2K4/MAPK1/MAPK3/MC1R/MGAT4B/MOGS/MTA3/MVB12A/MYO5A/MYO9B/NCK1/NCKIPSD/NEDD4L/NELFE/NMT1/NMT2/NPM1/NUP37/NUP54/NUP62/PARP4/PLK2/POLR2G/POLR2H/POLR2J/PPIA/PRKACB/PRKAR1A/PRKAR1B/PRKAR2B/PRKCSH/PRMT1/PSMA2/PSMA3/PSMA4/PSMA7/PSMB1/PSMB3/PSMB4/PSMC2/PSMC4/PSMD13/PSMD9/PSME1/PSME2/PSMF1/PTH1R/RAC1/RANGAP1/RELA/REST/RIPK1/RPL10/RPL10A/RPL11/RPL12/RPL13/RPL13A/RPL14/RPL17/RPL18/RPL18A/RPL19/RPL22L1/RPL23/RPL23A/RPL24/RPL26L1/RPL27/RPL27A/RPL28/RPL29/RPL30/RPL32/RPL35/RPL35A/RPL36/RPL36A/RPL39/RPL41/RPL5/RPL6/RPL7/RPL7A/RPL8/RPL9/RPLP0/RPLP1/RPLP2/RPN1/RPN2/RPS10/RPS11/RPS13/RPS14/RPS15/RPS15A/RPS16/RPS17/RPS18/RPS19/RPS2/RPS20/RPS21/RPS25/RPS27L/RPS28/RPS3/RPS3A/RPS4X/RPS5/RPS7/RPS8/RPS9/RPSA/S1PR1/SAP30/SEC13/SEM1/SH3GL1/SH3GL2/SLC25A4/SNAP25/STAM/STT3A/STX1A/STX1B/SV2A/SV2B/SV2C/SYT1/TAF12/TAF4B/TCEA1/TUBA4A/TUBA8/TUBB2A/TUSC3/TYK2/UBA52/VAMP1/VAMP2/VIP/VIPR1/VIPR2/VPS36/WASF3/WIPF2/WIPF3 | 226 | REACTOME |
| KEGG_FC_GAMMA_R_MEDIATED_PHAGOCYTOSIS | 1.63E-09 | 7.04E-08 | AKT3/ARPC1B/ARPC2/ARPC3/ARPC4/ARPC5/CRKL/DNM2/DOCK2/FCGR1A/FCGR2A/FCGR2B/FCGR2C/FCGR3A/GSN/HCK/INPP5D/LIMK2/LYN/MYO10/NCF1/PIK3CD/PIK3CG/PIK3R5/PLCG2/PLD2/PLPP3/PRKCA/PRKCD/PTPRC/RAC2/SCIN/SPHK1/SPHK2/SYK/VASP/VAV1/WAS/WASF2 | 39 | KEGG |
| REACTOME_APC_C_MEDIATED_DEGRADATION_OF_CELL_CYCLE_PROTEINS | 3.23E-09 | 7.38E-08 | AURKA/AURKB/BUB1B/BUB3/CCNA2/CCNB1/CDC20/CDC23/CDK1/CDK2/FBXO5/FZR1/MAD2L1/NEK2/PLK1/PSMB2/PSMB7/PSMD11/PSMD14/PSMD3/PSMD5/PSMD6/PSME3/PTTG1/SKP1/SKP2/UBE2C/UBE2S | 28 | REACTOME |
| KEGG_PRIMARY_IMMUNODEFICIENCY | 1.77E-09 | 7.55E-08 | BLNK/BTK/CD3D/CD3E/CD4/CD40/CD40LG/CD79A/CD8A/CD8B/CIITA/ICOS/IL2RG/IL7R/JAK3/LCK/PTPRC/RFXAP/TAP1/TAP2/ZAP70 | 21 | KEGG |
| REACTOME_SEROTONIN_NEUROTRANSMITTER_RELEASE_CYCLE | 1.05E-09 | 8.55E-08 | CPLX1/PPFIA2/PPFIA3/PPFIA4/RAB3A/RIMS1/SNAP25/STX1A/STXBP1/SYN1/SYN2/SYN3/SYT1/TSPOAP1/VAMP2 | 15 | REACTOME |
| REACTOME_TELOMERE_MAINTENANCE | 3.94E-09 | 8.91E-08 | CCNA2/CDK2/CHTF18/DKC1/DNA2/DSCC1/FEN1/H2AX/H2AZ1/H2BC17/H2BC9/H3-3A/LIG1/PCNA/PIF1/POLA1/POLA2/POLD3/POLR2D/PRIM1/PRIM2/RFC2/RFC3/RFC4/RFC5/RPA1/RPA2/RTEL1/RUVBL1/SHQ1/WRAP53/WRN | 32 | REACTOME |
| PID_TCR_PATHWAY | 2.14E-09 | 8.99E-08 | CARD11/CD247/CD28/CD3D/CD3E/CD3G/CD4/CD80/CD86/CSK/FYB1/FYN/GRAP2/GRB2/HLA-DRA/ITK/LCK/LCP2/MAP3K8/MAP4K1/PRKCA/PTPN11/PTPN6/PTPRC/RAP1A/RASSF5/SLA2/VAV1/WAS/ZAP70 | 30 | PID |
| REACTOME_CELLULAR_RESPONSES_TO_STIMULI | 1.15E-09 | 9.08E-08 | ACTR10/ACTR1A/AJUBA/AKT1S1/ANAPC11/ATF4/ATP6V0C/ATP6V0D1/ATP6V0E1/ATP6V1A/ATP6V1B2/ATP6V1C1/ATP6V1D/ATP6V1E1/ATP6V1G2/ATP6V1H/BAG4/BLVRA/CALR/CAMK2A/CAMK2B/CAMK2G/CBX6/CCNA1/CDC26/CDKN2D/CEBPG/COX16/COX5A/COX6A1/CRTC1/CSNK2B/DCTN1/DEPDC5/DNAJA4/DNAJB11/DYNC1H1/DYNC1I1/EED/EEF1A1/EIF2S3/ERF/ERN1/EXOSC4/EXOSC7/EXTL1/FAU/FBXL17/FNIP2/GSK3A/GSTP1/H2AC20/H2AC8/H3-3B/H3C10/H4C11/HELZ2/HIF1A/HIF1AN/HIF3A/HM13/HMOX2/HSF1/HSP90AB1/HSP90B1/HSPA12A/HSPA12B/HSPA4L/HSPA5/HSPH1/KEAP1/KLHDC3/LAMTOR2/LAMTOR5/LMNA/MAP2K4/MAPK1/MAPK10/MAPK3/MAPK7/MAPK9/MAPKAPK2/MEF2C/MEF2D/MINK1/MOV10/MYDGF/NCOA2/NDUFA4/NR3C2/NUP37/NUP54/NUP62/P4HB/PDIA6/PHC2/POT1/PPP2R5B/PSMA2/PSMA3/PSMA4/PSMA7/PSMB1/PSMB3/PSMB4/PSMC2/PSMC4/PSMD13/PSMD9/PSME1/PSME2/PSMF1/RELA/RPA3/RPL10/RPL10A/RPL11/RPL12/RPL13/RPL13A/RPL14/RPL17/RPL18/RPL18A/RPL19/RPL22L1/RPL23/RPL23A/RPL24/RPL26L1/RPL27/RPL27A/RPL28/RPL29/RPL30/RPL32/RPL35/RPL35A/RPL36/RPL36A/RPL39/RPL41/RPL5/RPL6/RPL7/RPL7A/RPL8/RPL9/RPLP0/RPLP1/RPLP2/RPS10/RPS11/RPS13/RPS14/RPS15/RPS15A/RPS16/RPS17/RPS18/RPS19/RPS2/RPS20/RPS21/RPS25/RPS27L/RPS28/RPS3/RPS3A/RPS4X/RPS5/RPS7/RPS8/RPS9/RPSA/SEC13/SEM1/SESN1/SNCB/SRPRB/SSR1/STAP2/SYVN1/TERF2IP/TINF2/TP53/TSPYL2/TUBA4A/TUBA8/TUBB2A/UBA52/UBE2E1/USP46/WFS1/YIF1A | 195 | REACTOME |
| REACTOME_NUCLEAR_PORE_COMPLEX_NPC_DISASSEMBLY | 4.34E-09 | 9.61E-08 | CCNB1/CCNB2/CDK1/NDC1/NUP107/NUP155/NUP160/NUP188/NUP205/NUP210/NUP35/NUP42/NUP43/NUP50/NUP85/NUP88/RAE1 | 17 | REACTOME |
| REACTOME_PROCESSIVE_SYNTHESIS_ON_THE_LAGGING_STRAND | 4.34E-09 | 9.61E-08 | DNA2/FEN1/LIG1/PCNA/POLA1/POLA2/POLD3/PRIM1/PRIM2/RPA1/RPA2 | 11 | REACTOME |
| BIOCARTA_MCM_PATHWAY | 4.57E-09 | 1.00E-07 | CCNE1/CDC6/CDK2/CDT1/MCM2/MCM3/MCM4/MCM5/MCM6/MCM7/ORC1/ORC6 | 12 | BIOCARTA |
| BIOCARTA_CTLA4_PATHWAY | 2.50E-09 | 1.03E-07 | CD247/CD28/CD3D/CD3E/CD3G/CD80/CD86/CTLA4/GRB2/HLA-DRA/HLA-DRB1/ICOS/ICOSLG/ITK/LCK/PTPN11 | 16 | BIOCARTA |
| REACTOME_HIV_INFECTION | 6.41E-09 | 1.39E-07 | CHMP6/CHMP7/ELOA/FEN1/GTF2E1/GTF2F2/GTF2H4/HMGA1/KPNB1/LIG1/NDC1/NELFB/NELFCD/NUP107/NUP155/NUP160/NUP188/NUP205/NUP210/NUP35/NUP42/NUP43/NUP50/NUP85/NUP88/PAK2/POLR2D/PSMB2/PSMB7/PSMD11/PSMD14/PSMD3/PSMD5/PSMD6/PSME3/RAE1/RAN/RANBP1/RCC1/SKP1/SSRP1/SUPT16H/TAF11/TAF15/TAF4/TAF6/TBP/VPS37B/XRCC4/XRCC5/XRCC6 | 51 | REACTOME |
| WP_FRAGILE_X_SYNDROME | 1.89E-09 | 1.45E-07 | AGAP2/AKAP5/AKT1S1/AP2A1/AP2M1/ARAF/ARHGAP32/CAMK2A/CAMK2B/CAMK4/CLTB/CYFIP2/DLG4/DLGAP3/DNM1/EEF1A1/EIF4A1/EPHA4/GABRA1/GABRB2/GABRD/GRIN1/GRIN2A/GRIN2B/GRIP1/GRIP2/GRM1/GRM5/HOMER1/ITPR1/KCNC1/MAP2K1/MAPK1/NTRK2/PIK3CB/PLCB1/PPP1CA/PPP2R5B/PPP3CA/PRKAR1A/PTPN5/RAP1GAP/SH3GL1/SHANK1/SLC16A1/SLC6A1/SYNGAP1/TARBP2 | 48 | WP |
| WP_GASTRIC_CANCER_NETWORK_1 | 7.29E-09 | 1.56E-07 | AURKA/CENPF/E2F7/ECT2/H3-3A/KIF15/KIF20B/LIN9/MCM4/MYBL2/NUP107/RUVBL1/TOP2A/TPX2/UBE2C | 15 | WP |
| REACTOME_DOPAMINE_NEUROTRANSMITTER_RELEASE_CYCLE | 2.14E-09 | 1.60E-07 | APBA1/CPLX1/LIN7B/PPFIA2/PPFIA3/PPFIA4/RAB3A/RIMS1/SNAP25/STX1A/STXBP1/SYN1/SYN2/SYN3/SYT1/TSPOAP1/VAMP2 | 17 | REACTOME |
| HALLMARK_DNA_REPAIR | 7.74E-09 | 1.64E-07 | ALYREF/CSTF3/DGCR8/DGUOK/ELOA/FEN1/GTF3C5/LIG1/NELFB/NELFCD/NME1/NT5C3A/PCNA/POLA1/POLA2/POLD3/POLH/POLR1C/POLR2D/POLR3C/POLR3GL/PRIM1/RAD51/RAE1/RFC2/RFC3/RFC4/RFC5/RPA2/SAC3D1/SF3A3/SNAPC5/SSRP1/TAF6/TYMS/UMPS/VPS37B/ZWINT | 38 | HALLMARK |
| WP_PYRIMIDINE_METABOLISM | 8.52E-09 | 1.79E-07 | CAD/CTPS1/DCTPP1/DHODH/DTYMK/NME1/POLA1/POLA2/POLD3/POLE/POLE2/POLE3/POLR1C/POLR1E/POLR2D/POLR3C/POLR3D/POLR3F/POLR3GL/PRIM1/PRIM2/RRM1/RRM2/TK1/TYMS/UCK2/UMPS | 27 | WP |
| REACTOME_CARDIAC_CONDUCTION | 2.80E-09 | 2.04E-07 | AHCYL1/ATP1A2/ATP1A3/ATP1B1/ATP2B1/ATP2B2/ATP2B3/CACNA1C/CACNA2D2/CACNB1/CACNB2/CACNG8/CALM1/CAMK2A/CAMK2B/CAMK2G/CASQ1/CASQ2/FGF12/FGF13/FKBP1B/FXYD7/ITPR1/KCNIP2/KCNIP3/KCNIP4/KCNJ11/KCNJ12/KCNJ4/KCNK1/KCNK10/KCNK12/KCNK3/KCNK4/KCNK9/NKX2-5/NOS1/NPPC/RYR1/RYR2/SCN2A/SCN2B/SCN3B/SCN4B/SCN8A/SLC8A1/SLC8A2/STIM1/TRPC1 | 49 | REACTOME |
| KEGG_CALCIUM_SIGNALING_PATHWAY | 2.87E-09 | 2.04E-07 | ADCY1/ADCY2/ADRA1A/ADRA1B/ADRB1/ATP2B1/ATP2B2/ATP2B3/CACNA1A/CACNA1C/CACNA1D/CACNA1E/CACNA1G/CACNA1I/CALM1/CALM2/CALM3/CAMK2A/CAMK2B/CAMK2G/CAMK4/CCKBR/CHRM1/CHRM3/CHRNA7/DRD1/ERBB4/F2R/GNAL/GNAQ/GRIN1/GRIN2A/GRIN2C/GRM1/GRM5/HRH2/HTR2A/ITPKA/ITPR1/NOS1/P2RX5/P2RX6/PDE1A/PDE1B/PLCB1/PLCB3/PLCB4/PPP3CA/PPP3CB/PPP3R1/PRKACB/PRKCB/PRKCG/PTK2B/RYR1/RYR2/SLC25A4/SLC8A1/SLC8A2/TACR2/TRPC1/VDAC3 | 62 | KEGG |
| BIOCARTA_NOS1_PATHWAY | 3.08E-09 | 2.13E-07 | CALM1/CALM2/CALM3/DLG4/GRIN1/GRIN2A/GRIN2B/GRIN2C/NOS1/PPP3CA/PPP3CB/PRKACB/PRKAR1A/PRKAR1B/PRKAR2B/PRKCB | 16 | BIOCARTA |
| KEGG_ANTIGEN_PROCESSING_AND_PRESENTATION | 5.49E-09 | 2.23E-07 | B2M/CD4/CD74/CD8A/CD8B/CIITA/CTSB/CTSL/CTSS/HLA-A/HLA-B/HLA-C/HLA-DMA/HLA-DMB/HLA-DOA/HLA-DOB/HLA-DPA1/HLA-DPB1/HLA-DQA1/HLA-DQA2/HLA-DQB1/HLA-DRA/HLA-DRB1/HLA-E/HLA-F/HSPA1A/HSPA1B/HSPA6/IFI30/LGMN/LTA/NFYB/RFXAP/TAP1/TAP2/TAPBP | 36 | KEGG |
| REACTOME_RESPIRATORY_ELECTRON_TRANSPORT | 3.14E-10 | 2.28E-07 | COX11/COX14/COX18/COX4I1/COX5B/COX6B1/COX6C/COX7B/COX7C/COX8A/ETFB/LRPPRC/NDUFA1/NDUFA11/NDUFA13/NDUFA2/NDUFA3/NDUFA7/NDUFA8/NDUFAF3/NDUFB1/NDUFB10/NDUFB11/NDUFB2/NDUFB3/NDUFB4/NDUFB7/NDUFS5/NDUFS7/NDUFS8/NDUFV1/SCO1/SURF1/UQCR10/UQCR11/UQCRH/UQCRQ | 37 | REACTOME |
| REACTOME_CHROMATIN_MODIFYING_ENZYMES | 3.65E-10 | 2.28E-07 | ARID1A/ARID1B/ARID2/ARID4A/ARID4B/ASH1L/ATF2/ATF7IP/ATXN7/BRPF3/CHD4/CLOCK/COPRS/CREBBP/DNMT3A/DPY30/DR1/ELP1/ELP3/EP300/EP400/EPC1/GATAD2B/H2AC15/H2BC7/H3C13/H4-16/HCFC1/ING3/KANSL1/KAT6A/KAT7/KDM1B/KDM2A/KDM3A/KDM3B/KDM4A/KDM5A/KDM5B/KDM5C/KDM6A/KDM7A/KMT2A/KMT2C/KMT2D/KMT2E/MSL2/NCOR1/NCOR2/NSD1/NSD3/PBRM1/PHF2/PHF20/PHF21A/PHF8/PRMT3/RBBP5/SAP130/SAP30L/SETD1A/SETD1B/SETD2/TADA2B/TAF10/TBL1X/TBL1XR1/TRRAP/YEATS2/ZZZ3 | 70 | REACTOME |
| REACTOME_SUMOYLATION_OF_CHROMATIN_ORGANIZATION_PROTEINS | 1.12E-08 | 2.33E-07 | CBX2/CBX8/HDAC2/NDC1/NUP107/NUP155/NUP160/NUP188/NUP205/NUP210/NUP35/NUP42/NUP43/NUP50/NUP85/NUP88/PCGF2/RAE1/RING1/RNF2/SUMO2/SUZ12/UBE2I/ZBED1 | 24 | REACTOME |
| REACTOME_TRANSLESION_SYNTHESIS_BY_POLH | 1.13E-08 | 2.34E-07 | NPLOC4/PCNA/POLH/RFC2/RFC3/RFC4/RFC5/RPA1/RPA2/SPRTN/UFD1/VCP | 12 | REACTOME |
| REACTOME_POLO_LIKE_KINASE_MEDIATED_EVENTS | 1.27E-08 | 2.58E-07 | CCNB1/CCNB2/CDC25A/CDC25C/CENPF/FOXM1/LIN9/MYBL2/PKMYT1/PLK1/RBBP4 | 11 | REACTOME |
| WP_REGULATION_OF_SISTER_CHROMATID_SEPARATION_AT_THE_METAPHASEANAPHASE_TRANSITION | 1.27E-08 | 2.58E-07 | BUB1/BUB1B/BUB3/CDC20/CENPE/ESPL1/MAD1L1/MAD2L1/PTTG1/SMC1A/SMC3 | 11 | WP |
| PID_ATM_PATHWAY | 1.33E-08 | 2.66E-07 | ABL1/BRCA1/CDC25A/CDC25C/CHEK2/COP1/FANCD2/H2AX/MRE11/RBBP8/SMC1A/SMC3/TOP3A/TRIM28/UIMC1/XRCC4 | 16 | PID |
| WP_DISRUPTION_OF_POSTSYNAPTIC_SIGNALING_BY_CNV | 4.43E-09 | 2.99E-07 | CAMK2A/CAMK2B/CAMK2G/DLG2/DLGAP1/GRIN1/GRIN2A/GRIN2B/GRIN2C/GRM1/HOMER1/MAPK1/MAPK3/NRXN1/NRXN3/RPH3A/RYR2/SHANK1/STX1A/SYNGAP1/YWHAG | 21 | WP |
| REACTOME_SIGNALING_BY_RHO_GTPASES_MIRO_GTPASES_AND_RHOBTB3 | 1.51E-08 | 3.00E-07 | ABL1/ANKLE2/ARAP3/ARHGAP11A/ARHGAP11B/ARHGAP19/ARHGAP26/ARHGDIA/ARHGEF2/ARHGEF39/AURKB/BIRC5/BUB1/BUB1B/BUB3/CCNE1/CCT2/CCT6A/CCT7/CDC20/CDC25C/CDC42SE2/CDCA8/CENPA/CENPE/CENPF/CENPH/CENPI/CENPK/CENPL/CENPM/CENPN/CENPO/CENPP/CENPQ/CENPU/DDX39B/DEPDC1B/DIAPH1/DIAPH3/DSN1/DVL2/DVL3/ECT2/ERCC6L/FGD4/FLOT2/GJA1/H2AX/H2AZ1/H2BC17/H2BC9/H3-3A/H3C2/H3C3/HNRNPC/INCENP/IQGAP3/KDM1A/KIF14/KIF18A/KIF2C/KNL1/KNTC1/LBR/LMNB1/LRRC41/MAD1L1/MAD2L1/MEN1/MIS12/NDC80/NDE1/NOXA1/NSL1/NUF2/NUP107/NUP160/NUP43/NUP85/PAK2/PKN3/PLK1/PMF1/PPP1CC/PRC1/PREX2/RACGAP1/RBMX/RCC2/SENP1/SGO1/SGO2/SKA1/SKA2/SOWAHC/SPC24/SPC25/SPDL1/STIP1/TMPO/TNFAIP1/TRA2B/TUBA1A/TUBA1B/VANGL1/VCP/VHL/YWHAQ/ZNF512B/ZW10/ZWILCH/ZWINT | 113 | REACTOME |
| REACTOME_AURKA_ACTIVATION_BY_TPX2 | 1.53E-08 | 3.01E-07 | AURKA/CDK1/CENPJ/CEP131/CEP41/CEP72/CEP76/DCTN3/HAUS1/HAUS2/HAUS3/HAUS6/HAUS8/HMMR/NDE1/NEDD1/NEK2/ODF2/PLK1/PLK4/TPX2/TUBA1A/TUBB/TUBG1 | 24 | REACTOME |
| PID_P53_DOWNSTREAM_PATHWAY | 7.90E-09 | 3.17E-07 | AIFM2/APC/ARID3A/ATF3/BCL2A1/BDKRB2/BTG2/CASP1/CASP10/CCNG1/CDKN1A/CTSD/DDIT4/DKK1/DROSHA/DUSP1/FAS/GADD45A/GDF15/GPX1/HGF/IRF5/JMY/JUN/LIF/MET/NDRG1/NFYB/NLRC4/PCBP4/PERP/PLK3/PMAIP1/PML/PRDM1/PYCARD/RCHY1/RNF144B/SERPINE1/SH2D1A/SPP1/STEAP3/TAP1/TNFRSF10A/TNFRSF10C/TNFRSF10D/TP53BP2/VDR | 48 | PID |
| KEGG_MISMATCH_REPAIR | 1.82E-08 | 3.55E-07 | EXO1/LIG1/MLH1/MSH2/MSH6/PCNA/POLD3/RFC2/RFC3/RFC4/RFC5/RPA1/RPA2 | 13 | KEGG |
| BIOCARTA_NKT_PATHWAY | 9.96E-09 | 3.94E-07 | CCL3/CCL4/CCR1/CCR4/CCR5/CCR7/CD28/CD4/CD40LG/CXCR3/CXCR4/IFNGR1/IFNGR2/IL12RB1/IL18R1/IL4R/TGFB1 | 17 | BIOCARTA |
| REACTOME_GLUTAMATE_NEUROTRANSMITTER_RELEASE_CYCLE | 6.18E-09 | 4.06E-07 | CPLX1/GLS/GLS2/PPFIA2/PPFIA3/PPFIA4/RAB3A/RIMS1/SLC17A7/SLC1A2/SLC1A3/SNAP25/STX1A/STXBP1/SYT1/TSPOAP1/VAMP2 | 17 | REACTOME |
| BIOCARTA_TCYTOTOXIC_PATHWAY | 1.07E-08 | 4.12E-07 | CD2/CD247/CD28/CD3D/CD3E/CD3G/CD8A/ICAM1/ITGAL/ITGB2/PTPRC | 11 | BIOCARTA |
| BIOCARTA_THELPER_PATHWAY | 1.07E-08 | 4.12E-07 | CD2/CD247/CD28/CD3D/CD3E/CD3G/CD4/ICAM1/ITGAL/ITGB2/PTPRC | 11 | BIOCARTA |
| WP_VITAMIN_D_RECEPTOR_PATHWAY | 1.18E-08 | 4.47E-07 | ABCA11P/ADRB2/ALOX5/ATP2C2/CAMP/CASP5/CD14/CD40/CDKN1A/CDKN1B/CEACAM1/CEBPA/CRACR2A/CRACR2B/CST6/CTLA4/CYP2S1/EPHB4/G0S2/G6PD/GADD45A/GXYLT2/HLA-DQA1/HLA-DQA2/HLA-DRB1/ID1/IGFBP1/IRF5/IRF8/ITGAM/JUNB/KLF4/LGALS9/LRRC25/MED9/MX2/MXD1/NFATC2/NINJ1/PPARD/PRDM1/PTGER4/S100A4/S100A6/S100A8/S100A9/SERPINB1/SLC37A2/SPP1/STEAP4/SULT1C2/TGFB1/THBD/TNFAIP3/TNFRSF11B/TNFSF4/TRAK1/TREM1/VDR | 59 | WP |
| WP_BASE_EXCISION_REPAIR | 2.36E-08 | 4.56E-07 | APEX1/FEN1/HMGB1/LIG1/NEIL3/PARP1/PCNA/POLD3/POLE/POLE2/POLE3/SMUG1/TDG/UNG/XRCC1 | 15 | WP |
| WP_SELECTIVE_EXPRESSION_OF_CHEMOKINE_RECEPTORS_DURING_TCELL_POLARIZATION | 1.22E-08 | 4.57E-07 | CCL3/CCL4/CCR1/CCR2/CCR4/CCR5/CCR7/CD28/CD4/CD40LG/CXCR3/CXCR4/IFNGR1/IFNGR2/IL12RB1/IL18R1/IL4R/TGFB1 | 18 | WP |
| WP_NUCLEOTIDEBINDING_OLIGOMERIZATION_DOMAIN_NOD_PATHWAY | 1.29E-08 | 4.77E-07 | ACAP1/AIM2/CARD6/CARD9/CASP1/CASP5/CASP7/CASP8/IL18/IL1B/MEFV/NAIP/NFKBIA/NLRC4/NLRP1/NLRP12/NLRP3/NOD1/NOD2/PRDM1/PYCARD/SUGT1 | 22 | WP |
| WP_PHOTODYNAMIC_THERAPYINDUCED_NFKB_SURVIVAL_SIGNALING | 1.41E-08 | 5.12E-07 | BCL2A1/BIRC3/CD40LG/CFLAR/CXCL2/CXCL8/ICAM1/IL1A/IL1B/IL6/MMP1/NFKB1/NFKB2/PTGS2/REL/RELB/SELE/TNF/TNFRSF1A/VCAM1 | 20 | WP |
| REACTOME_TP53_REGULATES_TRANSCRIPTION_OF_CELL_CYCLE_GENES | 2.94E-08 | 5.59E-07 | AURKA/CCNA2/CCNB1/CCNE1/CCNE2/CDC25C/CDK1/CDK2/CENPJ/CNOT10/CNOT8/CNOT9/E2F1/E2F7/E2F8/PCNA/RBL1/TFDP1/ZNF385A | 19 | REACTOME |
| WP_DNA_DAMAGE_RESPONSE | 3.04E-08 | 5.73E-07 | ABL1/ATRIP/BRCA1/CCNB1/CCNB2/CCNE1/CCNE2/CDC25A/CDC25C/CDK1/CDK2/CDK4/CDK6/CHEK1/CHEK2/E2F1/FANCD2/GADD45G/H2AX/MRE11/RAD51/RPA2/SMC1A | 23 | WP |
| REACTOME_MUSCLE_CONTRACTION | 8.95E-09 | 5.74E-07 | ACTA1/ACTC1/AHCYL1/ANXA6/ATP1A2/ATP1A3/ATP1B1/ATP2B1/ATP2B2/ATP2B3/CACNA1C/CACNA2D2/CACNB1/CACNB2/CACNG8/CALM1/CAMK2A/CAMK2B/CAMK2G/CASQ1/CASQ2/FGF12/FGF13/FKBP1B/FXYD7/GUCY1A1/GUCY1B1/ITGB5/ITPR1/KCNIP2/KCNIP3/KCNIP4/KCNJ11/KCNJ12/KCNJ4/KCNK1/KCNK10/KCNK12/KCNK3/KCNK4/KCNK9/LMOD1/MYBPC1/MYH11/MYL6/NKX2-5/NOS1/NPPC/PAK1/PXN/RYR1/RYR2/SCN2A/SCN2B/SCN3B/SCN4B/SCN8A/SLC8A1/SLC8A2/SORBS1/STIM1/TMOD2/TNNT1/TPM2/TRPC1 | 65 | REACTOME |
| WP_ATM_SIGNALING_PATHWAY | 3.12E-08 | 5.82E-07 | ABL1/BRCA1/CASP2/CCNB1/CCNE1/CDC25A/CDC25C/CDK1/CDK2/CHEK1/CHEK2/FANCD2/H2AX/MRE11/RAD51/SMC1A/TP73 | 17 | WP |
| REACTOME_SNRNP_ASSEMBLY | 3.19E-08 | 5.90E-07 | GEMIN2/GEMIN4/GEMIN6/NDC1/NUP107/NUP155/NUP160/NUP188/NUP205/NUP210/NUP35/NUP42/NUP43/NUP50/NUP85/NUP88/PRMT5/RAE1/SNRPD1/WDR77 | 20 | REACTOME |
| REACTOME_POLYMERASE_SWITCHING | 3.44E-08 | 6.32E-07 | PCNA/POLA1/POLA2/POLD3/PRIM1/PRIM2/RFC2/RFC3/RFC4/RFC5 | 10 | REACTOME |
| REACTOME_TRANSPORT_OF_THE_SLBP_DEPENDANT_MATURE_MRNA | 3.67E-08 | 6.68E-07 | ALYREF/NDC1/NUP107/NUP155/NUP160/NUP188/NUP205/NUP210/NUP35/NUP42/NUP43/NUP50/NUP85/NUP88/RAE1/SLBP | 16 | REACTOME |
| REACTOME_ACTIVATION_OF_NMDA_RECEPTORS_AND_POSTSYNAPTIC_EVENTS | 1.07E-08 | 6.70E-07 | ADCY1/APBA1/CALM1/CAMK2A/CAMK2B/CAMK2G/CAMK4/CAMKK1/CAMKK2/DLG2/DLG3/DLG4/ERBB4/GIT1/GRIN1/GRIN2A/GRIN2B/GRIN2C/GRIN3A/KIF17/LIN7B/LRRC7/MAPK1/MAPK3/NBEA/NEFL/NRGN/PDPK1/PRKAB2/PRKACB/PRKAG2/PRKAR1A/PRKAR1B/PRKAR2B/RAC1/RASGRF2/TUBA4A/TUBA8/TUBB2A | 39 | REACTOME |
| REACTOME_THE_CITRIC_ACID_TCA_CYCLE_AND_RESPIRATORY_ELECTRON_TRANSPORT | 1.80E-09 | 7.50E-07 | ATP5F1D/ATP5F1E/ATP5MC1/ATP5MC2/ATP5MC3/ATP5ME/ATP5MF/ATP5MG/ATP5PD/COX11/COX14/COX18/COX4I1/COX5B/COX6B1/COX6C/COX7B/COX7C/COX8A/DLAT/DLD/ETFB/GSTZ1/LRPPRC/MPC2/NDUFA1/NDUFA11/NDUFA13/NDUFA2/NDUFA3/NDUFA7/NDUFA8/NDUFAF3/NDUFB1/NDUFB10/NDUFB11/NDUFB2/NDUFB3/NDUFB4/NDUFB7/NDUFS5/NDUFS7/NDUFS8/NDUFV1/PDPR/SCO1/SURF1/UQCR10/UQCR11/UQCRH/UQCRQ | 51 | REACTOME |
| REACTOME_PARASITE_INFECTION | 2.27E-08 | 8.13E-07 | ABI2/ACTB/ARPC1B/ARPC2/ARPC3/ARPC4/ARPC5/BRK1/BTK/CD247/CD3G/CYFIP1/ELMO2/FCGR3A/FGR/FYN/GRB2/HCK/IGHG1/IGHG2/IGHG4/IGKV1-5/IGKV1D-39/IGKV3-11/IGKV3-15/IGKV3-20/IGKV4-1/IGLC2/IGLC3/IGLV1-40/IGLV1-44/IGLV2-14/IGLV2-8/LYN/MYO10/NCKAP1L/PTK2/SYK/VAV1/WAS/WASF2/WIPF1 | 42 | REACTOME |
| REACTOME_GABA_RECEPTOR_ACTIVATION | 1.33E-08 | 8.13E-07 | ADCY1/ADCY2/ADCY5/ARHGEF9/GABBR1/GABBR2/GABRA1/GABRA2/GABRA4/GABRA5/GABRB1/GABRB2/GABRB3/GNAI1/GNAI2/GNAI3/GNAL/GNB1/GNB2/GNB5/GNG12/GNG3/GNG5/KCNJ12/KCNJ3/KCNJ4/KCNJ6/KCNJ9/NPTN | 29 | REACTOME |
| WP_DNA_IRDOUBLE_STRAND_BREAKS_AND_CELLULAR_RESPONSE_VIA_ATM | 4.58E-08 | 8.26E-07 | ABL1/BRCA1/BRCA2/CDC25C/CHEK1/CHEK2/E2F1/EXO1/FANCD2/H2AX/MCPH1/MRE11/NABP2/PARP1/PCNA/RAD51/SMC1A/SMC3/TP73/TRIM28 | 20 | WP |
| REACTOME_NS1_MEDIATED_EFFECTS_ON_HOST_PATHWAYS | 4.86E-08 | 8.70E-07 | CPSF4/KPNA2/KPNB1/NDC1/NUP107/NUP155/NUP160/NUP188/NUP205/NUP210/NUP35/NUP42/NUP43/NUP50/NUP85/NUP88/RAE1 | 17 | REACTOME |
| REACTOME_REGULATION_OF_PLK1_ACTIVITY_AT_G2_M_TRANSITION | 4.99E-08 | 8.86E-07 | AURKA/BORA/CCNB1/CCNB2/CDK1/CENPJ/CEP131/CEP41/CEP72/CEP76/DCTN3/HAUS1/HAUS2/HAUS3/HAUS6/HAUS8/NDE1/NEDD1/NEK2/ODF2/PLK1/PLK4/SKP1/TUBA1A/TUBB/TUBG1 | 26 | REACTOME |
| REACTOME_GENERATION_OF_SECOND_MESSENGER_MOLECULES | 2.56E-08 | 9.06E-07 | CD247/CD3D/CD3E/CD3G/CD4/FYB1/GRAP2/HLA-DPA1/HLA-DPB1/HLA-DQA1/HLA-DQA2/HLA-DQB1/HLA-DRA/HLA-DRB1/ITK/LCK/LCP2/PLCG2/VASP/WAS/ZAP70 | 21 | REACTOME |
| KEGG_ASTHMA | 2.62E-08 | 9.17E-07 | CD40/CD40LG/FCER1A/FCER1G/HLA-DMA/HLA-DMB/HLA-DOA/HLA-DOB/HLA-DPA1/HLA-DPB1/HLA-DQA1/HLA-DQA2/HLA-DQB1/HLA-DRA/HLA-DRB1/IL10/RNASE3/TNF | 18 | KEGG |
| REACTOME_ORC1_REMOVAL_FROM_CHROMATIN | 5.58E-08 | 9.83E-07 | CCNA2/CDC6/CDK2/CDT1/MCM2/MCM3/MCM4/MCM5/MCM6/MCM7/MCM8/ORC1/ORC6/PSMB2/PSMB7/PSMD11/PSMD14/PSMD3/PSMD5/PSMD6/PSME3/SKP1/SKP2 | 23 | REACTOME |
| REACTOME_RNA_POLYMERASE_II_TRANSCRIPTION_TERMINATION | 6.06E-08 | 1.06E-06 | ALYREF/CHTOP/CPSF3/CPSF4/CSTF1/CSTF2/CSTF3/DDX39B/EIF4A3/FIP1L1/MAGOHB/RBM8A/RNPS1/SLBP/SRSF1/SRSF2/SRSF3/SRSF7/THOC5/U2AF2/WDR33/ZNF473 | 22 | REACTOME |
| WP_PLATELETMEDIATED_INTERACTIONS_WITH_VASCULAR_AND_CIRCULATING_CELLS | 3.11E-08 | 1.07E-06 | CCL2/CCL5/CD40/CD40LG/ICAM1/IL1B/SELE/SELPLG/TGFB1/TLR2/TLR4/TLR7/VCAM1 | 13 | WP |
| REACTOME_POTASSIUM_CHANNELS | 1.90E-08 | 1.14E-06 | ABCC8/GABBR1/GABBR2/GNB1/GNB2/GNB5/GNG12/GNG3/GNG5/HCN4/KCNA1/KCNA2/KCNA3/KCNA5/KCNAB1/KCNAB2/KCNB1/KCNC1/KCNC3/KCNC4/KCNH1/KCNH3/KCNH4/KCNJ11/KCNJ12/KCNJ3/KCNJ4/KCNJ6/KCNJ9/KCNK1/KCNK10/KCNK3/KCNK4/KCNK9/KCNMA1/KCNN1/KCNQ2/KCNQ3/KCNQ5/KCNS1/KCNS3 | 41 | REACTOME |
| REACTOME_EXPORT_OF_VIRAL_RIBONUCLEOPROTEINS_FROM_NUCLEUS | 6.79E-08 | 1.18E-06 | NDC1/NUP107/NUP155/NUP160/NUP188/NUP205/NUP210/NUP35/NUP42/NUP43/NUP50/NUP85/NUP88/RAE1/RAN | 15 | REACTOME |
| REACTOME_CYCLIN_A_B1_B2_ASSOCIATED_EVENTS_DURING_G2_M_TRANSITION | 6.91E-08 | 1.18E-06 | CCNA2/CCNB1/CCNB2/CDC25A/CDC25B/CDC25C/CDK1/CDK2/FOXM1/PKMYT1/PLK1/PPP2R2A/PPP2R3B | 13 | REACTOME |
| REACTOME_GAP_FILLING_DNA_REPAIR_SYNTHESIS_AND_LIGATION_IN_GG_NER | 6.91E-08 | 1.18E-06 | LIG1/PCNA/POLD3/POLE/POLE2/POLE3/RFC2/RFC3/RFC4/RFC5/RPA1/RPA2/XRCC1 | 13 | REACTOME |
| WP_COMMON_PATHWAYS_UNDERLYING_DRUG_ADDICTION | 2.07E-08 | 1.21E-06 | ACTG1/ADCY1/ARAF/CALM1/CALM2/CAMK2A/CAMK4/DRD1/GNAI1/GRIN1/GRIN2A/GRM1/GRM5/MAP2K1/MAPK1/MAPK3/PPP1CA/PPP1CB/PPP1R1A/PRKACB/PRKCB/PRKCG/RAP1B | 23 | WP |
| WP_G_PROTEIN_SIGNALING_PATHWAYS | 2.16E-08 | 1.24E-06 | ADCY1/ADCY2/ADCY5/AKAP11/AKAP5/AKAP6/CALM1/CALM2/GNA13/GNAI1/GNAI2/GNAI3/GNAL/GNAO1/GNAQ/GNAZ/GNB1/GNB2/GNB5/GNG12/GNG3/GNG5/ITPR1/KCNJ3/PDE1A/PDE1B/PDE4A/PDE8B/PLCB3/PPP3CA/PRKACB/PRKAR1A/PRKAR1B/PRKAR2B/PRKCB/PRKCE/PRKCG/PRKCZ/RHOA | 39 | WP |
| REACTOME_CYCLIN_D_ASSOCIATED_EVENTS_IN_G1 | 8.53E-08 | 1.44E-06 | ABL1/CCNE1/CCNE2/CDK2/CDK4/CDK6/CDKN2C/CKS1B/E2F1/E2F2/E2F3/E2F5/PPP2R2A/PPP2R3B/RBL1/SKP1/SKP2/TFDP1 | 18 | REACTOME |
| WP_B_CELL_RECEPTOR_SIGNALING_PATHWAY | 4.34E-08 | 1.48E-06 | BLNK/BTK/CARD11/CD79A/CD79B/CD81/CRKL/DAPP1/FYN/GRB2/HCLS1/INPP5D/JUN/KLF11/LAT2/LCK/LYN/MAP2K6/MAP4K1/MAX/NFATC2/NFKB1/NFKBIA/PIK3AP1/PIK3CG/PLCG2/PRKCD/PTPN11/PTPN18/PTPN6/PTPRC/RAC2/REL/RPS6KA1/SYK/TEC/VAV1 | 37 | WP |
| BIOCARTA_TCAPOPTOSIS_PATHWAY | 4.48E-08 | 1.51E-06 | CCR5/CD247/CD28/CD3D/CD3E/CD3G/CD4/FAS/FASLG | 9 | BIOCARTA |
| WP_INTERACTIONS_BETWEEN_IMMUNE_CELLS_AND_MICRORNAS_IN_TUMOR_MICROENVIRONMENT | 4.61E-08 | 1.53E-06 | CCL2/CCL5/CD274/CD80/CD86/CTLA4/CXCL10/IL2RA/IL2RB/IL2RG/IL4R/NFKB1/NFKB2/PDCD1/SOCS1/STAT6/TGFB1/TGFBR2/TLR4/TLR7/TLR8 | 21 | WP |
| REACTOME_E2F_MEDIATED_REGULATION_OF_DNA_REPLICATION | 1.11E-07 | 1.87E-06 | CCNB1/CDK1/E2F1/MCM8/ORC1/ORC6/POLA1/POLA2/PPP2R3B/PRIM1/PRIM2/TFDP1 | 12 | REACTOME |
| WP_THYMIC_STROMAL_LYMPHOPOIETIN_TSLP_SIGNALING_PATHWAY | 5.89E-08 | 1.94E-06 | BTK/CISH/CXCL8/FES/FYN/HCK/IL2RA/IL6/IL7R/JAK2/LCK/LYN/NFKB1/NFKB2/NFKBIA/PI4K2A/PTPN11/RELB/STAT5A/STAT6/TEC/TNFSF4/TSLP | 23 | WP |
| WP_MODULATORS_OF_TCR_SIGNALING_AND_T_CELL_ACTIVATION | 6.17E-08 | 2.00E-06 | CARD11/CD247/CD28/CD3D/CD3E/CD3G/CD5/CD8A/CDKN1B/GRAP2/GRB2/ITK/LCK/LCP2/MAP3K8/MAP4K1/NFKB1/NFKBIA/PTPN6/REL/RHOH/SH2D1A/SOCS1/TNFAIP3/VAV1/ZAP70/ZFP36L1 | 27 | WP |
| REACTOME_RECOGNITION_OF_DNA_DAMAGE_BY_PCNA_CONTAINING_REPLICATION_COMPLEX | 1.24E-07 | 2.07E-06 | DTL/PCNA/POLD3/POLE/POLE2/POLE3/RAD18/RFC2/RFC3/RFC4/RFC5/RPA1/RPA2/USP1 | 14 | REACTOME |
| WP_IMMUNE_RESPONSE_TO_TUBERCULOSIS | 7.98E-08 | 2.56E-06 | IFI35/IFIT3/IFITM1/IFNGR1/IFNGR2/IRF1/IRF9/JAK2/MX1/OAS1/PSMB8/PTPN2/SOCS1/STAT2/TAP1 | 15 | WP |
| KEGG_COMPLEMENT_AND_COAGULATION_CASCADES | 8.15E-08 | 2.59E-06 | A2M/BDKRB2/C1QA/C1QB/C1QC/C1R/C1S/C2/C3/C3AR1/C5AR1/C7/CD55/CFB/CFD/CFH/CFI/CR1/F13A1/F5/PLAU/PLAUR/PROS1/SERPINA1/SERPINA5/SERPINE1/SERPINF2/SERPING1/THBD | 29 | KEGG |
| KEGG_JAK_STAT_SIGNALING_PATHWAY | 8.34E-08 | 2.62E-06 | AKT3/CCND2/CISH/CLCF1/CNTFR/CSF2RA/CSF2RB/CSF3/CSF3R/EPOR/GRB2/IFNGR1/IFNGR2/IFNLR1/IL10/IL10RA/IL10RB/IL11/IL12RB1/IL13RA1/IL15/IL15RA/IL21R/IL23A/IL2RA/IL2RB/IL2RG/IL3RA/IL4R/IL6/IL6R/IL7/IL7R/IRF9/JAK2/JAK3/LIF/OSM/PIK3CD/PIK3CG/PIK3R5/PIM1/PTPN11/PTPN6/SOCS1/SOCS3/STAT2/STAT5A/STAT6/TSLP | 50 | KEGG |
| HALLMARK_EPITHELIAL_MESENCHYMAL_TRANSITION | 8.59E-08 | 2.63E-06 | ABI3BP/ANPEP/AREG/CAPG/CCN1/CD44/COL7A1/COL8A2/COLGALT1/CXCL1/CXCL12/CXCL6/CXCL8/DAB2/DCN/DKK1/ECM2/EMP3/FAP/FAS/FBLN1/FBLN5/FSTL3/FUCA1/GADD45A/GADD45B/GEM/GLIPR1/IL15/IL32/IL6/INHBA/ITGA2/JUN/LAMC2/LGALS1/MFAP5/MGP/MMP1/NNMT/PLAUR/PTX3/SAT1/SERPINE1/SFRP4/SPP1/TFPI2/TGFB1/TGFBI/TGM2/THBS1/TIMP1/TNFAIP3/TNFRSF11B/TNFRSF12A/VCAM1/VEGFC/VIM/WIPF1/WNT5A | 60 | HALLMARK |
| HALLMARK_HYPOXIA | 8.59E-08 | 2.63E-06 | AMPD3/ANXA2/ATF3/BCAN/CA12/CAVIN1/CAVIN3/CCN1/CDKN1A/CDKN1B/CHST2/CITED2/CP/CXCR4/DCN/DDIT4/DUSP1/ERRFI1/FBP1/FOS/FOSL2/FOXO3/GAA/GLRX/HAS1/HEXA/HMOX1/IER3/IGFBP1/IL6/ISG20/JUN/MT2A/NAGK/NCAN/NDRG1/PFKFB3/PFKL/PIM1/PLAC8/PLAUR/PLIN2/PNRC1/PPARGC1A/PPP1R15A/PRKCA/S100A4/SDC2/SDC3/SERPINE1/SLC2A3/SLC2A5/SLC6A6/STC1/TGFBI/TGM2/TIPARP/TNFAIP3/TPST2/ZFP36 | 60 | HALLMARK |
| WP_HEMATOPOIETIC_STEM_CELL_DIFFERENTIATION | 9.34E-08 | 2.80E-06 | CIITA/CSF1/CSF3/CXCR4/FLI1/FOS/FOSB/HES6/HMGN5/IKZF1/IL1A/IL1B/IL6/IRF5/LEF1/LYL1/MUC1/NCKAP1L/NFATC2/NFE2/PIM1/RHOH/SPI1/STAT5A/TRAF3IP3/VAV1/ZNF835 | 27 | WP |
| WP_TCELL_ANTIGEN_RECEPTOR_TCR_PATHWAY_DURING_STAPHYLOCOCCUS_AUREUS_INFECTION | 9.34E-08 | 2.80E-06 | CARD11/CD28/CD3D/CD4/CD40LG/CD8A/CTLA4/FOS/FYN/GRAP2/GRB2/ICOS/IL10/ITK/JUN/LCK/LCP2/MAP3K8/NFATC2/NFKB1/NFKBIA/PDCD1/PTPN6/PTPRC/RRAS/TNF/ZAP70 | 27 | WP |
| WP_CYTOKINES_AND_INFLAMMATORY_RESPONSE | 9.61E-08 | 2.85E-06 | CD4/CSF1/CSF3/CXCL1/CXCL2/HLA-DRA/HLA-DRB1/IL10/IL11/IL15/IL1A/IL1B/IL6/IL7/TGFB1/TNF | 16 | WP |
| KEGG_BASE_EXCISION_REPAIR | 1.78E-07 | 2.94E-06 | APEX1/FEN1/HMGB1/LIG1/NEIL3/PARP1/PCNA/POLD3/POLE/POLE2/POLE3/SMUG1/TDG/UNG/XRCC1 | 15 | KEGG |
| KEGG_PYRIMIDINE_METABOLISM | 1.79E-07 | 2.94E-06 | CAD/CTPS1/DHODH/DTYMK/NME1/NT5C3A/POLA1/POLA2/POLD3/POLE/POLE2/POLE3/POLR1C/POLR1E/POLR2D/POLR3C/POLR3D/POLR3F/POLR3GL/PRIM1/PRIM2/RRM1/RRM2/TK1/TYMS/UCK2/UMPS | 27 | KEGG |
| REACTOME_TNFS_BIND_THEIR_PHYSIOLOGICAL_RECEPTORS | 1.04E-07 | 3.05E-06 | CD27/CD70/FASLG/LTA/TNFRSF11B/TNFRSF14/TNFRSF1A/TNFRSF1B/TNFRSF4/TNFRSF6B/TNFRSF8/TNFSF13/TNFSF13B/TNFSF14/TNFSF15/TNFSF4/TNFSF8 | 17 | REACTOME |
| PID_IL12_STAT4_PATHWAY | 1.05E-07 | 3.05E-06 | CD247/CD28/CD3D/CD3E/CD3G/CD4/CD80/CD86/FOS/HLA-DRA/IL18/IL18R1/IL18RAP/IL2RA/IRF1/JUN/PRF1/TGFB1 | 18 | PID |
| REACTOME_FCGAMMA_RECEPTOR_FCGR_DEPENDENT_PHAGOCYTOSIS | 1.07E-07 | 3.09E-06 | ABI2/ACTB/ARPC1B/ARPC2/ARPC3/ARPC4/ARPC5/BRK1/BTK/CD247/CD3G/CYFIP1/ELMO2/FCGR1A/FCGR2A/FCGR3A/FGR/FYN/GRB2/HCK/IGHG1/IGHG2/IGHG4/IGKV1-5/IGKV1D-39/IGKV3-11/IGKV3-15/IGKV3-20/IGKV4-1/IGLC2/IGLC3/IGLV1-40/IGLV1-44/IGLV2-14/IGLV2-8/LYN/MYO10/NCKAP1L/PLCG2/PLD2/PRKCD/PTK2/SYK/VAV1/WAS/WASF2/WIPF1 | 47 | REACTOME |
| WP_COMPLEMENT_AND_COAGULATION_CASCADES | 1.21E-07 | 3.42E-06 | C1QA/C1QB/C1QC/C1R/C1S/C2/C3/C3AR1/C5AR1/C7/CD55/CFB/CFD/CFH/CFI/CR1/F5/PLAU/PLAUR/PROS1/SERPINA1/SERPINA5/SERPINE1/SERPINF2/SERPING1/THBD | 26 | WP |
| BIOCARTA_RANMS_PATHWAY | 2.15E-07 | 3.50E-06 | AURKA/KIF15/KPNA2/KPNB1/RAN/RANBP1/RCC1/TPX2 | 8 | BIOCARTA |
| REACTOME_POSTMITOTIC_NUCLEAR_PORE_COMPLEX_NPC_REFORMATION | 2.23E-07 | 3.60E-06 | KPNB1/NDC1/NUP107/NUP155/NUP160/NUP188/NUP205/NUP35/NUP43/NUP85/RAN/RCC1/UBE2I | 13 | REACTOME |
| REACTOME_GABA_SYNTHESIS_RELEASE_REUPTAKE_AND_DEGRADATION | 6.42E-08 | 3.61E-06 | CPLX1/DNAJC5/GAD2/RAB3A/RIMS1/SLC32A1/SLC6A1/SLC6A12/SLC6A13/SNAP25/STX1A/STXBP1/SYT1/VAMP2 | 14 | REACTOME |
| WP_INTEGRATED_CANCER_PATHWAY | 2.46E-07 | 3.95E-06 | BARD1/BRCA1/CDC25A/CDC25B/CDK1/CDK2/CDK4/CHEK1/CHEK2/E2F1/MAP3K5/MRE11/MSH2/MSH6/NOXA1/PLK1/SMAD2 | 17 | WP |
| REACTOME_ANTIGEN_PROCESSING_CROSS_PRESENTATION | 1.41E-07 | 3.97E-06 | B2M/BTK/CD14/CD36/CTSL/CTSS/CYBA/CYBB/FCGR1A/FCGR1B/HLA-A/HLA-B/HLA-C/HLA-E/HLA-F/LY96/MRC1/NCF1/NCF2/NCF4/PSMA1/PSMA5/PSMB10/PSMB8/PSMB9/S100A8/S100A9/SNAP23/STX4/TAP1/TAP2/TAPBP/TLR1/TLR2/TLR4/TLR6/VAMP3/VAMP8 | 38 | REACTOME |
| REACTOME_INFLAMMASOMES | 1.46E-07 | 4.02E-06 | AIM2/CASP1/HMOX1/MEFV/NFKB1/NFKB2/NLRC4/NLRP1/NLRP3/PSTPIP1/PYCARD/SUGT1/TXN/TXNIP | 14 | REACTOME |
| WP_PATHOGENESIS_OF_SARSCOV2_MEDIATED_BY_NSP9NSP10_COMPLEX | 1.46E-07 | 4.02E-06 | CD2/CD247/CD3E/CD3G/CD4/CD8A/CD8B/CXCL8/FYN/HLA-DRA/HLA-DRB1/IL6/LCK/ZAP70 | 14 | WP |
| REACTOME_NUCLEOTIDE_BINDING_DOMAIN_LEUCINE_RICH_REPEAT_CONTAINING_RECEPTOR_NLR_SIGNALING_PATHWAYS | 1.54E-07 | 4.21E-06 | AIM2/BIRC3/CARD9/CASP1/CASP4/CASP8/HMOX1/IRAK2/MAP2K6/MAPK13/MEFV/NFKB1/NFKB2/NLRC4/NLRP1/NLRP3/NOD1/NOD2/PSTPIP1/PYCARD/SUGT1/TNFAIP3/TXN/TXNIP/UBE2N | 25 | REACTOME |
| REACTOME_CASPASE_ACTIVATION_VIA_DEATH_RECEPTORS_IN_THE_PRESENCE_OF_LIGAND | 1.57E-07 | 4.24E-06 | CASP8/CD14/CFLAR/FAS/FASLG/LY96/TICAM1/TICAM2/TLR4/TNFRSF10A/TNFSF10/TRADD | 12 | REACTOME |
| WP_ALZHEIMERS_DISEASE | 7.89E-08 | 4.34E-06 | ADRM1/APBB1/APH1A/ARAF/ATF4/ATG13/ATG2B/CACNA1C/CACNA1D/CALM1/CALM2/CALM3/CAPN2/CASP3/CDK5R1/CHRNA7/CSNK2B/DKK2/DVL1/ERN1/FADD/FRAT1/FZD2/FZD9/GNAQ/GRIN1/GRIN2A/GRIN2B/GRIN2C/HSD17B10/IKBKB/ITPR1/KIF5A/KIF5C/KLC1/KLC2/LPL/MAP2K1/MAPK1/MAPK10/MAPK3/MAPK9/NCSTN/NOS1/PIK3CB/PIK3R1/PLCB1/PLCB3/PLCB4/PPP3CA/PPP3CB/PPP3R1/PSENEN/PSMA2/PSMA3/PSMA4/PSMA7/PSMB1/PSMB3/PSMB4/PSMC2/PSMC4/PSMD13/PSMD9/RB1CC1/RELA/RTN3/RTN4/SEM1/SLC25A4/SNCA/TUBA4A/TUBA8/TUBB2A/ULK1/VDAC3/WNT10B/WNT2B/WNT7A | 79 | WP |
| KEGG_B_CELL_RECEPTOR_SIGNALING_PATHWAY | 1.84E-07 | 4.92E-06 | AKT3/BLNK/BTK/CARD11/CD72/CD79A/CD79B/CD81/DAPP1/FCGR2B/FOS/GRB2/IFITM1/INPP5D/JUN/LILRB3/LYN/NFATC2/NFKB1/NFKBIA/NFKBIE/PIK3AP1/PIK3CD/PIK3CG/PIK3R5/PLCG2/PTPN6/RAC2/SYK/VAV1 | 30 | KEGG |
| REACTOME_REGULATION_OF_GLUCOKINASE_BY_GLUCOKINASE_REGULATORY_PROTEIN | 3.36E-07 | 5.35E-06 | NDC1/NUP107/NUP155/NUP160/NUP188/NUP205/NUP210/NUP35/NUP42/NUP43/NUP50/NUP85/NUP88/RAE1 | 14 | REACTOME |
| KEGG_NATURAL_KILLER_CELL_MEDIATED_CYTOTOXICITY | 2.03E-07 | 5.38E-06 | CD244/CD247/CD48/FAS/FASLG/FCER1G/FCGR3A/FCGR3B/FYN/GRB2/GZMB/HCST/HLA-A/HLA-B/HLA-C/HLA-E/ICAM1/IFNGR1/IFNGR2/ITGAL/ITGB2/LCK/LCP2/MICA/MICB/NFATC2/PIK3CD/PIK3CG/PIK3R5/PLCG2/PRF1/PRKCA/PTPN11/PTPN6/RAC2/SH2D1A/SYK/TNF/TNFRSF10A/TNFRSF10C/TNFRSF10D/TNFSF10/TYROBP/VAV1/ZAP70 | 45 | KEGG |
| KEGG_HOMOLOGOUS_RECOMBINATION | 3.80E-07 | 6.01E-06 | BRCA2/EME1/MRE11/MUS81/POLD3/RAD51/RAD51D/RAD54B/RAD54L/RPA1/RPA2/TOP3A/XRCC2 | 13 | KEGG |
| BIOCARTA_TCRA_PATHWAY | 2.41E-07 | 6.32E-06 | CD247/CD3D/CD3E/CD3G/CD4/FYN/HLA-DRA/HLA-DRB1/LCK/PTPRC/ZAP70 | 11 | BIOCARTA |
| REACTOME_GLOBAL_GENOME_NUCLEOTIDE_EXCISION_REPAIR_GG_NER | 4.11E-07 | 6.40E-06 | ACTR5/COPS3/COPS7B/GTF2H4/INO80C/LIG1/MCRS1/PARP1/PCNA/POLD3/POLE/POLE2/POLE3/RAD23B/RFC2/RFC3/RFC4/RFC5/RPA1/RPA2/RUVBL1/SUMO2/UBE2I/XRCC1 | 24 | REACTOME |
| REACTOME_HCMV_LATE_EVENTS | 4.11E-07 | 6.40E-06 | CHMP1A/CHMP6/CHMP7/H2AC11/H2AC13/H2AC16/H2AC17/H2BC17/H2BC9/H3C2/H3C3/HNRNPK/NDC1/NUP107/NUP155/NUP160/NUP188/NUP205/NUP210/NUP35/NUP42/NUP43/NUP50/NUP85/NUP88/RAE1/SNF8/VPS25/VPS37B | 29 | REACTOME |
| KEGG_T_CELL_RECEPTOR_SIGNALING_PATHWAY | 2.47E-07 | 6.41E-06 | AKT3/CARD11/CD247/CD28/CD3D/CD3E/CD3G/CD4/CD40LG/CD8A/CD8B/CTLA4/FOS/FYN/GRAP2/GRB2/ICOS/IL10/ITK/JUN/LCK/LCP2/MAP3K8/MAPK13/NFATC2/NFKB1/NFKBIA/NFKBIE/PDCD1/PIK3CD/PIK3CG/PIK3R5/PTPN6/PTPRC/TEC/TNF/VAV1/ZAP70 | 38 | KEGG |
| REACTOME_INTERACTIONS_OF_VPR_WITH_HOST_CELLULAR_PROTEINS | 4.28E-07 | 6.61E-06 | HMGA1/NDC1/NUP107/NUP155/NUP160/NUP188/NUP205/NUP210/NUP35/NUP42/NUP43/NUP50/NUP85/NUP88/RAE1 | 15 | REACTOME |
| BIOCARTA_BARD1_PATHWAY | 4.49E-07 | 6.84E-06 | BARD1/BRCA1/FANCA/FANCC/FANCD2/FANCE/FANCG | 7 | BIOCARTA |
| REACTOME_DNA_REPLICATION_INITIATION | 4.49E-07 | 6.84E-06 | POLA1/POLA2/POLE/POLE2/POLE3/PRIM1/PRIM2 | 7 | REACTOME |
| REACTOME_HCMV_INFECTION | 4.72E-07 | 7.14E-06 | CHMP1A/CHMP6/CHMP7/EZH2/H2AC11/H2AC13/H2AC16/H2AC17/H2BC17/H2BC9/H3C2/H3C3/HNRNPK/NDC1/NUP107/NUP155/NUP160/NUP188/NUP205/NUP210/NUP35/NUP42/NUP43/NUP50/NUP85/NUP88/RAE1/RBBP4/RBBP7/SNF8/SUZ12/TRIM28/TUBA1A/TUBA1B/VPS25/VPS37B | 36 | REACTOME |
| REACTOME_CYCLIN_A_CDK2_ASSOCIATED_EVENTS_AT_S_PHASE_ENTRY | 5.23E-07 | 7.85E-06 | CCNA2/CCNE1/CCNE2/CDC25A/CDC25B/CDK2/CDK4/CKS1B/E2F1/E2F5/FZR1/LIN9/PSMB2/PSMB7/PSMD11/PSMD14/PSMD3/PSMD5/PSMD6/PSME3/RBBP4/SKP1/SKP2/TFDP1 | 24 | REACTOME |
| WP_COMPLEMENT_SYSTEM_IN_NEURONAL_DEVELOPMENT_AND_PLASTICITY | 3.23E-07 | 8.32E-06 | ATP8B3/AXL/C1QA/C1QB/C1QC/C1R/C1S/C2/C3/C3AR1/C5AR1/C5AR2/C7/CASP10/CASP7/CASP8/CD55/CFB/CFD/CFH/CFI/COLEC12/CR1/CRB1/FAS/FASLG/FCN1/GAS6/ITGAM/ITGAX/ITGB2/MARK1/MERTK/PLSCR1/PROS1/SCRIB/SERPING1/TGFB1 | 38 | WP |
| REACTOME_PHOSPHORYLATION_OF_EMI1 | 6.57E-07 | 9.79E-06 | CCNB1/CDC20/CDK1/FBXO5/FZR1/PLK1 | 6 | REACTOME |
| REACTOME_DISEASES_OF_IMMUNE_SYSTEM | 3.92E-07 | 9.99E-06 | BTK/CD14/CD36/LY96/NFKB1/NFKB2/NFKBIA/S100A8/S100A9/TICAM1/TLR1/TLR2/TLR3/TLR4/TLR5/TLR6/UNC93B1 | 17 | REACTOME |
| REACTOME_CONDENSATION_OF_PROMETAPHASE_CHROMOSOMES | 7.24E-07 | 1.07E-05 | CCNB1/CCNB2/CDK1/NCAPD2/NCAPG/NCAPH/SMC2/SMC4 | 8 | REACTOME |
| KEGG_HUNTINGTONS_DISEASE | 3.43E-08 | 1.12E-05 | AP2S1/APAF1/ATP5F1D/ATP5F1E/ATP5MC1/ATP5MC2/ATP5MC3/ATP5PD/CLTC/COX4I1/COX5B/COX6B1/COX6C/COX7B/COX7C/COX8A/CREB1/CREB5/CREBBP/DCTN4/EP300/HIP1/NDUFA1/NDUFA2/NDUFA3/NDUFA7/NDUFA8/NDUFB1/NDUFB10/NDUFB2/NDUFB3/NDUFB4/NDUFB7/NDUFS5/NDUFS7/NDUFS8/NDUFV1/POLR2A/POLR2B/POLR2C/POLR2E/POLR2I/SIN3A/SP1/TFAM/UQCR10/UQCR11/UQCRH/UQCRQ | 49 | KEGG |
| KEGG_UBIQUITIN_MEDIATED_PROTEOLYSIS | 3.61E-08 | 1.12E-05 | ANAPC1/BIRC2/BIRC6/CBL/CDC27/CUL3/CUL4B/CUL5/DDB1/DET1/ELOB/ERCC8/FBXW8/HERC2/HUWE1/ITCH/KLHL13/MAP3K1/MGRN1/MID1/NEDD4/PIAS1/PIAS2/PIAS3/RBX1/SMURF1/SMURF2/STUB1/TRAF6/TRIM32/TRIP12/UBA2/UBE2G2/UBE2L3/UBE3A/UBE3B/UBE3C/UBE4A/UBR5/XIAP | 40 | KEGG |
| WP_SPINAL_CORD_INJURY | 4.82E-07 | 1.22E-05 | AIF1/ANXA1/BCAN/BTG2/C1QB/CCL2/CCNG1/CCR2/CDKN1B/CXCL1/CXCL10/CXCL2/CXCL8/EGR1/FCGR2A/FCGR2C/FOS/FOXO3/GADD45A/ICAM1/IL1A/IL1B/IL1R1/IL6/LGALS3/LILRB2/LILRB3/LTB/NCAN/PLA2G2A/PRKCA/PTGS2/PTPRA/TGFB1/TLR4/TNF/TNFSF13/TNFSF13B/VIM/ZFP36 | 40 | WP |
| REACTOME_INTERLEUKIN_3_INTERLEUKIN_5_AND_GM_CSF_SIGNALING | 4.88E-07 | 1.22E-05 | BLNK/CRKL/CSF2RA/CSF2RB/FYN/GRB2/HCK/IL2RA/IL2RB/IL2RG/IL3RA/INPP5D/JAK2/JAK3/LYN/PIK3CD/PTPN11/PTPN6/STAT5A/SYK/TEC/VAV1 | 22 | REACTOME |
| WP_LUNG_FIBROSIS | 5.82E-07 | 1.44E-05 | BMP7/CCL2/CCL3/CCL4/CCL5/CCR2/CEBPB/CSF3/CXCL2/CXCL8/EGF/HGF/HMOX1/IGF1/IL1B/IL6/MT2A/PLAU/PTX3/SERPINA1/SMAD7/SPP1/STN1/TGFB1/TIMP1/TNF | 26 | WP |
| REACTOME_LONG_TERM_POTENTIATION | 2.71E-07 | 1.46E-05 | CALM1/CAMK2A/CAMK2B/CAMK2G/DLG2/DLG3/DLG4/ERBB4/GRIN1/GRIN2A/GRIN2B/GRIN2C/LRRC7/NEFL/NRGN | 15 | REACTOME |
| REACTOME_VIRAL_MESSENGER_RNA_SYNTHESIS | 1.02E-06 | 1.49E-05 | GTF2F2/NDC1/NUP107/NUP155/NUP160/NUP188/NUP205/NUP210/NUP35/NUP42/NUP43/NUP50/NUP85/NUP88/POLR2D/RAE1 | 16 | REACTOME |
| PID_RB_1PATHWAY | 1.03E-06 | 1.51E-05 | ABL1/CCNA2/CCNE1/CDK2/CDK4/CDK6/DNMT1/E2F1/E2F2/E2F3/PPP2R3B/RAF1/RBBP4/SKP2/SMARCA4/SMARCB1/SUV39H1/TBP/TFDP1/UBTF | 20 | PID |
| REACTOME_NOREPINEPHRINE_NEUROTRANSMITTER_RELEASE_CYCLE | 2.86E-07 | 1.51E-05 | CPLX1/MAOA/PPFIA2/PPFIA3/PPFIA4/RAB3A/RIMS1/SNAP25/STX1A/STXBP1/SYT1/TSPOAP1/VAMP2 | 13 | REACTOME |
| KEGG_OXIDATIVE_PHOSPHORYLATION | 5.80E-08 | 1.61E-05 | ATP5F1D/ATP5F1E/ATP5MC1/ATP5MC2/ATP5MC3/ATP5ME/ATP5MF/ATP5MG/ATP5PD/ATP6V1F/COX11/COX17/COX4I1/COX5B/COX6B1/COX6C/COX7B/COX7C/COX8A/NDUFA1/NDUFA11/NDUFA2/NDUFA3/NDUFA7/NDUFA8/NDUFB1/NDUFB10/NDUFB2/NDUFB3/NDUFB4/NDUFB7/NDUFS5/NDUFS7/NDUFS8/NDUFV1/UQCR10/UQCR11/UQCRH/UQCRQ | 39 | KEGG |
| KEGG_LEUKOCYTE_TRANSENDOTHELIAL_MIGRATION | 6.63E-07 | 1.63E-05 | ACTB/ACTN1/ARHGAP35/CD99/CLDN1/CLDN23/CLDN4/CLDN7/CXCL12/CXCR4/CYBA/CYBB/F11R/ICAM1/ITGAL/ITGAM/ITGB2/ITK/MAPK13/MYL12A/MYL12B/NCF1/NCF2/NCF4/PIK3CD/PIK3CG/PIK3R5/PLCG2/PRKCA/PTK2/PTPN11/RAC2/RAP1A/RASSF5/RHOH/SIPA1/VASP/VAV1/VCAM1 | 39 | KEGG |
| WP_APOPTOSIS_MODULATION_AND_SIGNALING | 6.81E-07 | 1.66E-05 | AIFM2/BAG3/BCL2A1/BIK/BIRC3/BIRC7/BMF/CASP1/CASP10/CASP4/CASP7/CASP8/CFLAR/ENDOG/FAS/FASLG/FOS/HSPA1A/IL1R1/IL1R2/JUN/NAIP/NFKB1/NFKBIA/PMAIP1/TNFRSF10A/TNFRSF10C/TNFRSF10D/TNFRSF1A/TNFRSF1B/TNFRSF6B/TNFSF10/TRADD | 33 | WP |
| WP_ACUTE_VIRAL_MYOCARDITIS | 6.94E-07 | 1.66E-05 | ACTB/AIF1/CASP1/CASP7/CASP8/CCR5/CD4/CD40LG/CD55/CD80/CXCR4/DMD/ENDOG/FYN/HLA-DMA/IL10/IL6/ITGAL/ITGB2/MICA/NFKB2/NOD2/PTCRA/PYCARD/RAC2/SOCS1/TGFB1/TICAM1/TLR3/TLR4/TLR5/TNF | 32 | WP |
| WP_TCELL_ACTIVATION_SARSCOV2 | 6.94E-07 | 1.66E-05 | CARD11/CD247/CD28/CD3D/CD3E/CD3G/CD4/CD80/CD86/CDKN1A/CTLA4/FOS/FOXO3/FYN/GRAP2/GRB2/HLA-DRA/HLA-DRB1/ICOS/IL12RB1/IL18R1/IL23A/JAK2/JUN/LCK/LCP2/LTA/NFKB1/NFKBIA/PIK3CD/TNF/ZAP70 | 32 | WP |
| REACTOME_TRANSCRIPTIONAL_REGULATION_BY_TP53 | 6.76E-08 | 1.69E-05 | AGO1/AGO2/AGO3/AGO4/APAF1/ATF2/ATM/ATR/BLM/BRPF3/CCNT1/CDK12/CDK13/CHD4/CNOT1/CNOT4/CNOT6/CNOT6L/CNOT7/COX11/COX14/COX18/COX4I1/COX5B/COX6B1/COX6C/COX7B/COX7C/COX8A/CREBBP/CSNK2A1/CSNK2A2/ELOB/EP300/GATAD2B/GTF2H2/GTF2H3/KAT6A/LAMTOR3/LAMTOR4/LRPPRC/MAPK14/MDC1/MTOR/NBN/PHF20/PMS2/POLR2A/POLR2B/POLR2C/POLR2E/POLR2I/PRDX1/PRDX5/PRKAA1/PRR5/RABGGTA/RAD17/RAD50/RBL2/RNF34/RPS27A/RPTOR/RRAGD/SCO1/SLC38A9/SURF1/TAF1/TAF10/TAF2/TAF7/TAF9B/TFDP2/TIGAR/TMEM219/TNRC6A/TNRC6B/TP53INP1/USP7/ZNF420 | 80 | REACTOME |
| REACTOME_POLYMERASE_SWITCHING_ON_THE_C_STRAND_OF_THE_TELOMERE | 1.17E-06 | 1.69E-05 | CHTF18/DSCC1/PCNA/POLA1/POLA2/POLD3/PRIM1/PRIM2/RFC2/RFC3/RFC4/RFC5 | 12 | REACTOME |
| BIOCARTA_ATRBRCA_PATHWAY | 1.21E-06 | 1.74E-05 | BRCA1/BRCA2/CHEK1/CHEK2/FANCA/FANCC/FANCD2/FANCE/FANCG/MRE11/RAD51 | 11 | BIOCARTA |
| PID_INTEGRIN2_PATHWAY | 7.76E-07 | 1.81E-05 | C3/CCN1/CD40LG/F11R/FCGR2A/ICAM1/ICAM3/ICAM4/ITGAL/ITGAM/ITGAX/ITGB2/PLAU/PLAUR/TGFBI/VCAM1 | 16 | PID |
| WP_COVID19_ADVERSE_OUTCOME_PATHWAY | 7.78E-07 | 1.81E-05 | CCL2/CCL3/CSF3/CXCL10/CXCL8/IL10/IL1B/IL2RA/IL6/IL7/TNF | 11 | WP |
| WP_FOXP3_IN_COVID19 | 7.78E-07 | 1.81E-05 | CD28/CD80/CD86/FOXP3/IL2RA/IL2RB/IL2RG/IL6/IL6R/IL7R/STAT5A | 11 | WP |
| REACTOME_BASE_EXCISION_REPAIR | 1.31E-06 | 1.87E-05 | APEX1/FEN1/H2AX/H2AZ1/H2BC17/H2BC9/LIG1/NEIL3/PARP1/PCNA/POLD3/POLE/POLE2/POLE3/RFC2/RFC3/RFC4/RFC5/RPA1/RPA2/SMUG1/TDG/UNG/XRCC1 | 24 | REACTOME |
| REACTOME_DAG_AND_IP3_SIGNALING | 3.88E-07 | 2.01E-05 | ADCY1/ADCY2/ADCY5/AHCYL1/CALM1/CAMK2A/CAMK2B/CAMK2G/CAMK4/CAMKK1/CAMKK2/ITPR1/NBEA/PDE1A/PDE1B/PRKACB/PRKAR1A/PRKAR1B/PRKAR2B/PRKCE/PRKCG | 21 | REACTOME |
| REACTOME_PLATELET_ACTIVATION_SIGNALING_AND_AGGREGATION | 9.07E-07 | 2.10E-05 | A2M/ACTN1/APBB1IP/ARRB2/CD109/CD36/CFD/CSK/CTSW/CYB5R1/CYRIB/DAGLA/EGF/F13A1/F5/FCER1G/FERMT3/FYN/GAS6/GNA15/GNG10/GNG11/GNGT2/GRB2/HGF/IGF1/ISLR/LCK/LCP2/LHFPL2/LYN/MAGED2/PDPN/PIK3CG/PIK3R5/PIK3R6/PLCG2/PLEK/PPBP/PRKCA/PRKCD/PRKCH/PROS1/PSAP/PTK2/PTPN11/PTPN6/RAC2/RAP1A/RARRES2/RHOG/SCG3/SERPINA1/SERPINA3/SERPINE1/SERPINF2/SERPING1/SRGN/STX4/STXBP2/SYK/TAGLN2/TGFB1/THBS1/TIMP1/TLN1/TMSB4X/TOR4A/VAV1/VEGFC | 70 | REACTOME |
| REACTOME_MISMATCH_REPAIR | 1.57E-06 | 2.23E-05 | EXO1/LIG1/MLH1/MSH2/MSH6/PCNA/POLD3/RPA1/RPA2 | 9 | REACTOME |
| REACTOME_NEGATIVE_REGULATION_OF_NMDA_RECEPTOR_MEDIATED_NEURONAL_TRANSMISSION | 4.59E-07 | 2.33E-05 | CALM1/CAMK2A/CAMK2B/CAMK2G/CAMK4/DLG2/DLG3/DLG4/GRIN1/GRIN2A/GRIN2B/GRIN2C/LRRC7/NEFL | 14 | REACTOME |
| KEGG_PHOSPHATIDYLINOSITOL_SIGNALING_SYSTEM | 5.32E-07 | 2.65E-05 | CALM1/CALM2/CALM3/CDS1/CDS2/DGKB/DGKE/DGKG/DGKQ/DGKZ/IMPA1/INPP4A/INPP4B/INPP5A/INPP5J/INPPL1/ITPKA/ITPR1/PI4KA/PIK3CB/PIK3R1/PIP4K2B/PIP4K2C/PIP5K1B/PIP5K1C/PLCB1/PLCB3/PLCB4/PRKCB/PRKCG/SYNJ1 | 31 | KEGG |
| REACTOME_ANCHORING_OF_THE_BASAL_BODY_TO_THE_PLASMA_MEMBRANE | 1.97E-06 | 2.78E-05 | CDK1/CENPJ/CEP131/CEP41/CEP72/CEP76/CEP89/DCTN3/HAUS1/HAUS2/HAUS3/HAUS6/HAUS8/KIF24/MKS1/NDE1/NEDD1/NEK2/ODF2/PLK1/PLK4/SCLT1/TUBA1A/TUBB/TUBG1 | 25 | REACTOME |
| REACTOME_INITIATION_OF_NUCLEAR_ENVELOPE_NE_REFORMATION | 2.05E-06 | 2.86E-05 | ANKLE2/CCNB1/CCNB2/CDK1/KPNB1/LBR/LMNB1/PPP2R2A/TMPO/VRK1 | 10 | REACTOME |
| REACTOME_TRANSCRIPTION_OF_E2F_TARGETS_UNDER_NEGATIVE_CONTROL_BY_DREAM_COMPLEX | 2.05E-06 | 2.86E-05 | CDC25A/CDC6/E2F1/E2F5/LIN9/PCNA/RBBP4/RBL1/TFDP1/TOP2A | 10 | REACTOME |
| REACTOME_TRAFFICKING_AND_PROCESSING_OF_ENDOSOMAL_TLR | 1.25E-06 | 2.86E-05 | CNPY3/CTSB/CTSK/CTSL/CTSS/LGMN/TLR3/TLR7/TLR8/UNC93B1 | 10 | REACTOME |
| BIOCARTA_CELLCYCLE_PATHWAY | 2.12E-06 | 2.94E-05 | CCNB1/CCNE1/CDC25A/CDK1/CDK2/CDK4/CDK6/CDKN2C/E2F1/RBL1/TFDP1 | 11 | BIOCARTA |
| WP_SPLICING_FACTOR_NOVA_REGULATED_SYNAPTIC_PROTEINS | 6.54E-07 | 3.20E-05 | ANK3/ATP2B1/CADM3/CAMK2G/CLSTN1/EFNA5/EPB41L1/EPB41L3/GABBR2/GRIN1/GRIN2B/KCNJ6/KCNMA1/KCNQ2/MAPK9/NCDN/PLCB4/PRKCZ/RAP1GAP/SNW1/TERF2IP | 21 | WP |
| REACTOME_TERMINATION_OF_TRANSLESION_DNA_SYNTHESIS | 2.47E-06 | 3.38E-05 | PCLAF/PCNA/POLD3/POLE/POLE2/POLE3/POLH/RFC2/RFC3/RFC4/RFC5/RPA1/RPA2 | 13 | REACTOME |
| REACTOME_TP53_REGULATES_METABOLIC_GENES | 1.68E-07 | 3.82E-05 | AGO1/AGO2/AGO3/AGO4/COX11/COX14/COX18/COX4I1/COX5B/COX6B1/COX6C/COX7B/COX7C/COX8A/LAMTOR3/LAMTOR4/LRPPRC/MTOR/PRDX1/PRDX5/PRKAA1/RPTOR/RRAGD/SCO1/SLC38A9/SURF1/TIGAR/TNRC6A/TNRC6B | 29 | REACTOME |
| REACTOME_NEUROTOXICITY_OF_CLOSTRIDIUM_TOXINS | 8.53E-07 | 4.10E-05 | SNAP25/STX1A/STX1B/SV2A/SV2B/SV2C/SYT1/VAMP1/VAMP2 | 9 | REACTOME |
| BIOCARTA_IL2RB_PATHWAY | 1.82E-06 | 4.11E-05 | CFLAR/CRKL/FAS/FASLG/FOS/GRB2/IKZF3/IL2RA/IL2RB/IL2RG/JAK3/NMI/PIK3CG/PTPN6/SOCS1/SOCS3/STAT5A/SYK | 18 | BIOCARTA |
| PID_IL23_PATHWAY | 1.82E-06 | 4.11E-05 | CCL2/CD3E/CD4/CXCL1/CXCL9/IL12RB1/IL18/IL18R1/IL18RAP/IL1B/IL23A/IL6/JAK2/NFKB1/NFKBIA/SOCS3/STAT5A/TNF | 18 | PID |
| WP_MIRNA_REGULATION_OF_DNA_DAMAGE_RESPONSE | 3.06E-06 | 4.16E-05 | ABL1/ATRIP/BRCA1/CCNB1/CCNB2/CCNE1/CCNE2/CDC25A/CDC25C/CDK1/CDK2/CDK4/CDK6/CHEK1/CHEK2/E2F1/FANCD2/GADD45G/H2AX/MCM7/MRE11/RAD51/RPA2/SMC1A | 24 | WP |
| BIOCARTA_G1_PATHWAY | 3.08E-06 | 4.16E-05 | ABL1/CCNE1/CDC25A/CDK1/CDK2/CDK4/CDK6/DHFR/E2F1/SKP2/SMAD4/TFDP1 | 12 | BIOCARTA |
| WP_MEASLES_VIRUS_INFECTION | 1.87E-06 | 4.18E-05 | CASP8/CCND2/CD209/CD28/CD3D/CD3E/CD3G/CDKN1B/FAS/FASLG/FCGR2B/FOS/HSPA1A/HSPA1B/HSPA6/IFIH1/IKBKE/IL1A/IL1B/IL2RA/IL2RB/IL2RG/IL6/IRF7/IRF9/JAK3/JUN/MX1/NFKB1/NFKB2/NFKBIA/OAS1/OAS2/PIK3CD/RCHY1/SLAMF1/STAT2/STAT5A/TLR2/TLR4/TLR7/TNFAIP3/TRADD | 43 | WP |
| REACTOME_INTERLEUKIN_2_FAMILY_SIGNALING | 1.90E-06 | 4.21E-05 | CSF2RA/CSF2RB/GRB2/HAVCR2/IL15/IL15RA/IL21R/IL2RA/IL2RB/IL2RG/IL3RA/INPP5D/JAK2/JAK3/LCK/LGALS9/PIK3CD/PTPN6/STAT5A/SYK | 20 | REACTOME |
| REACTOME_RECRUITMENT_OF_MITOTIC_CENTROSOME_PROTEINS_AND_COMPLEXES | 3.15E-06 | 4.22E-05 | CDK1/CENPJ/CEP131/CEP41/CEP72/CEP76/DCTN3/HAUS1/HAUS2/HAUS3/HAUS6/HAUS8/NDE1/NEDD1/NEK2/ODF2/PLK1/PLK4/TUBA1A/TUBB/TUBG1/TUBGCP3 | 22 | REACTOME |
| REACTOME_TRANSCRIPTION_OF_E2F_TARGETS_UNDER_NEGATIVE_CONTROL_BY_P107_RBL1_AND_P130_RBL2_IN_COMPLEX_WITH_HDAC1 | 3.28E-06 | 4.38E-05 | CCNA2/CDK1/E2F1/E2F5/LIN9/MYBL2/RBBP4/RBL1/TFDP1 | 9 | REACTOME |
| REACTOME_DEATH_RECEPTOR_SIGNALLING | 9.88E-07 | 4.67E-05 | AATF/ABR/APH1A/ARHGEF17/ARHGEF33/ARHGEF4/ARHGEF40/ARHGEF9/BAG4/BEX3/CASP3/CLIP3/FADD/GNA13/HDAC1/IKBKB/IRAK1/ITGB3BP/ITSN1/KALRN/MADD/MCF2/MCF2L/NCSTN/NET1/NGEF/NSMAF/OTULIN/PLEKHG2/PLEKHG5/PSENEN/RAC1/RACK1/RASGRF2/RBCK1/RELA/RHOA/RIPK1/RIPK2/RTN4/RTN4R/SHARPIN/SMPD3/SPPL2A/TIAM1/TNFRSF10B/UBA52 | 47 | REACTOME |
| KEGG_OTHER_GLYCAN_DEGRADATION | 2.14E-06 | 4.68E-05 | FUCA1/FUCA2/GBA/GLB1/HEXA/HEXB/MAN2B1/MAN2B2/MANBA/NEU1/NEU4 | 11 | KEGG |
| REACTOME_THE_NLRP3_INFLAMMASOME | 2.14E-06 | 4.68E-05 | CASP1/HMOX1/MEFV/NFKB1/NFKB2/NLRP3/PSTPIP1/PYCARD/SUGT1/TXN/TXNIP | 11 | REACTOME |
| REACTOME_HEME_SIGNALING | 2.28E-07 | 4.74E-05 | ATF2/CHD9/CLOCK/CREB1/CREBBP/CRTC3/EP300/HBA1/HBA2/HBB/MED1/NCOA6/NCOR1/NRIP1/PPARA/RAI1/TBL1X/TBL1XR1/TGS1/XPO1 | 20 | REACTOME |
| WP_MYOMETRIAL_RELAXATION_AND_CONTRACTION_PATHWAYS | 1.05E-06 | 4.88E-05 | ACKR3/ACTA1/ACTC1/ACTG1/ADCY1/ADCY2/ADCY5/ARRB1/ATF4/CACNB3/CALM1/CALM2/CALM3/CAMK2A/CAMK2B/CAMK2G/CRHR1/DGKZ/GNAQ/GNB1/GNB2/GNB5/GNG12/GNG3/GNG5/GUCY1A1/IGFBP2/ITPR1/NOS1/PKIA/PLCB3/PRKACB/PRKAR1A/PRKAR1B/PRKAR2B/PRKCB/PRKCE/PRKCG/PRKCZ/RGS11/RGS20/RGS4/RGS5/RGS7/RYR1/RYR2/SLC8A1/YWHAB/YWHAG/YWHAH/YWHAZ | 51 | WP |
| WP_MACROPHAGE_MARKERS | 2.29E-06 | 4.95E-05 | CD14/CD163/CD68/CD74/CD83/CD86/LYZ/RAC2 | 8 | WP |
| WP_OXIDATIVE_DAMAGE_RESPONSE | 2.39E-06 | 5.13E-05 | C1QA/C1QB/C1QC/C1R/C1S/C2/C3AR1/C5AR1/CDKN1A/CDKN1B/GADD45A/MAPK13/NFKB1/NFKBIE/TDP2/TNF/TNFRSF1B/TNK2/TRAF1 | 19 | WP |
| WP_NUCLEOTIDE_EXCISION_REPAIR_IN_XERODERMA_PIGMENTOSUM | 3.89E-06 | 5.15E-05 | BRCA1/GTF2H4/H3-3A/HMGN1/LIG1/PARP1/PCNA/POLD3/POLE/POLE2/POLE3/POLH/RAD18/RAD23B/RFC2/RFC3/RFC4/RFC5/RPA1/RPA2/XRCC1 | 21 | WP |
| REACTOME_DISEASES_OF_MITOTIC_CELL_CYCLE | 4.03E-06 | 5.31E-05 | CCNE1/CCNE2/CDC23/CDK2/CDK4/CDK6/E2F1/E2F2/E2F3/FZR1/SKP2/TFDP1/UBE2C/UBE2S | 14 | REACTOME |
| REACTOME_CYTOPROTECTION_BY_HMOX1 | 3.00E-07 | 5.54E-05 | CHD9/COX11/COX14/COX18/COX4I1/COX5B/COX6B1/COX6C/COX7B/COX7C/COX8A/CREBBP/CSNK2A1/CSNK2A2/CUL3/HBA1/HBA2/HBB/LRPPRC/MED1/NCOA6/NCOR1/NCOR2/PPARA/PSMD1/PSMD12/PSMD2/PSME4/RBX1/RPS27A/SCO1/SIN3A/SURF1/TBL1X/TBL1XR1/TGS1 | 36 | REACTOME |
| WP_PATHWAYS_AFFECTED_IN_ADENOID_CYSTIC_CARCINOMA | 3.11E-07 | 5.54E-05 | ARID1A/ARID4B/ATM/ATRX/BCORL1/CMTR2/CREBBP/EP300/HRAS/IL17RD/JMJD1C/KANSL1/KAT6A/KDM6A/KMT2C/MAP2K2/MGA/NCOR1/NOTCH1/NSD1/PIK3CA/PRKDC/SETD2/SRCAP | 24 | WP |
| BIOCARTA_NDKDYNAMIN_PATHWAY | 1.25E-06 | 5.60E-05 | AP2A1/AP2M1/CALM1/CALM2/CALM3/DNM1/EPN1/EPS15/NME2/PPP3CA/PPP3CB/SYNJ1 | 12 | BIOCARTA |
| REACTOME_ACETYLCHOLINE_NEUROTRANSMITTER_RELEASE_CYCLE | 1.25E-06 | 5.60E-05 | CPLX1/PPFIA2/PPFIA3/PPFIA4/RAB3A/RIMS1/SNAP25/STX1A/STXBP1/SYT1/TSPOAP1/VAMP2 | 12 | REACTOME |
| BIOCARTA_IL12_PATHWAY | 2.77E-06 | 5.81E-05 | CCR5/CD247/CD3D/CD3E/CD3G/CXCR3/IL12RB1/IL18/IL18R1/JAK2/JUN/MAP2K6 | 12 | BIOCARTA |
| WP_MYD88_DISTINCT_INPUTOUTPUT_PATHWAY | 2.77E-06 | 5.81E-05 | IL1A/JUN/NFKB1/TIFA/TLR1/TLR2/TLR4/TLR5/TLR6/TLR7/TLR8/UBE2N | 12 | WP |
| REACTOME_CA_DEPENDENT_EVENTS | 1.32E-06 | 5.85E-05 | ADCY1/ADCY2/ADCY5/CALM1/CAMK2A/CAMK2B/CAMK2G/CAMK4/CAMKK1/CAMKK2/MAPK1/NBEA/PDE1A/PDE1B/PRKACB/PRKAR1A/PRKAR1B/PRKAR2B/PRKCG | 19 | REACTOME |
| WP_OXIDATIVE_PHOSPHORYLATION | 3.61E-07 | 6.00E-05 | ATP5F1D/ATP5F1E/ATP5MC1/ATP5MC2/ATP5MC3/ATP5ME/ATP5MF/ATP5MG/ATP5PD/NDUFA11/NDUFA2/NDUFA3/NDUFA7/NDUFA8/NDUFB1/NDUFB10/NDUFB2/NDUFB4/NDUFB7/NDUFS5/NDUFS7/NDUFS8/NDUFV1 | 23 | WP |
| REACTOME_GLYCOSPHINGOLIPID_METABOLISM | 2.92E-06 | 6.09E-05 | ARSA/ARSI/ARSJ/ASAH1/B3GALNT1/CTSA/ESYT1/ESYT2/GALC/GBA/GBA2/GLB1/GM2A/HEXA/HEXB/NEU1/NEU4/PSAP/SUMF1/UGCG | 20 | REACTOME |
| REACTOME_ADRENALINE_NORADRENALINE_INHIBITS_INSULIN_SECRETION | 1.41E-06 | 6.13E-05 | ADCY5/ADRA2A/ADRA2C/CACNA1C/CACNA1D/CACNA2D2/CACNB2/CACNB3/GNAI1/GNAI2/GNB1/GNB2/GNB5/GNG12/GNG3/GNG5 | 16 | REACTOME |
| REACTOME_INTERACTION_BETWEEN_L1_AND_ANKYRINS | 1.45E-06 | 6.18E-05 | ACTG1/ANK1/ANK2/ANK3/KCNQ2/KCNQ3/L1CAM/NFASC/SCN2A/SCN2B/SCN3B/SCN4B/SCN8A/SPTB/SPTBN1/SPTBN2/SPTBN4 | 17 | REACTOME |
| PID_FCER1_PATHWAY | 3.06E-06 | 6.32E-05 | BTK/DOK1/DUSP1/FCER1A/FCER1G/FCGR2B/FOS/FYN/GRB2/HCLS1/ITK/JUN/LAT2/LCP2/LYN/NFATC2/NFKB1/PLD2/PTK2/PTPN11/SPHK1/SYK/VAV1/WIPF1 | 24 | PID |
| REACTOME_ION_HOMEOSTASIS | 1.62E-06 | 6.81E-05 | AHCYL1/ATP1A2/ATP1A3/ATP1B1/ATP2B1/ATP2B2/ATP2B3/CALM1/CAMK2A/CAMK2B/CAMK2G/CASQ1/CASQ2/FKBP1B/FXYD7/ITPR1/KCNJ11/NOS1/RYR1/RYR2/SLC8A1/SLC8A2/STIM1/TRPC1 | 24 | REACTOME |
| WP_IL3_SIGNALING_PATHWAY | 3.40E-06 | 6.97E-05 | CD69/CD86/CRKL/CSF2RB/CXCL8/FOS/FYN/GRB2/HCK/IL3RA/INPP5D/JAK2/JUN/LYN/PIK3CD/PTPN11/PTPN6/STAT5A/SYK/TGFB1/VAV1 | 21 | WP |
| REACTOME_RESPONSE_TO_ELEVATED_PLATELET_CYTOSOLIC_CA2 | 3.52E-06 | 7.16E-05 | A2M/ACTN1/CD109/CD36/CFD/CTSW/CYB5R1/CYRIB/EGF/F13A1/F5/FERMT3/GAS6/HGF/IGF1/ISLR/LHFPL2/MAGED2/PLEK/PPBP/PRKCA/PROS1/PSAP/RARRES2/SCG3/SERPINA1/SERPINA3/SERPINE1/SERPINF2/SERPING1/SRGN/STX4/STXBP2/TAGLN2/TGFB1/THBS1/TIMP1/TLN1/TMSB4X/TOR4A/VEGFC | 41 | REACTOME |
| WP_IL2_SIGNALING_PATHWAY | 3.74E-06 | 7.56E-05 | CCND2/CISH/CRKL/FOS/FOXO3/FYN/GRB2/IL2RA/IL2RB/IL2RG/JAK3/JUN/LCK/MAPT/NMI/PTPN11/SOCS3/STAT5A/SYK | 19 | WP |
| BIOCARTA_BLYMPHOCYTE_PATHWAY | 3.77E-06 | 7.56E-05 | CD40/CD80/CR1/FCGR2B/HLA-DRA/HLA-DRB1/ICAM1/ITGAL/ITGB2/PTPRC | 10 | BIOCARTA |
| REACTOME_COMPLEMENT_CASCADE | 4.16E-06 | 8.29E-05 | C1QA/C1QB/C1QC/C1R/C1S/C2/C3/C3AR1/C5AR1/C5AR2/C7/CD55/CD81/CFB/CFD/CFH/CFI/CR1/FCN1/GZMM/IGHG1/IGHG2/IGHG4/IGKV1-5/IGKV1D-39/IGKV3-11/IGKV3-15/IGKV3-20/IGKV4-1/IGLC2/IGLC3/IGLV1-40/IGLV1-44/IGLV2-14/IGLV2-8/PROS1/SERPING1 | 37 | REACTOME |
| REACTOME_ABERRANT_REGULATION_OF_MITOTIC_G1_S_TRANSITION_IN_CANCER_DUE_TO_RB1_DEFECTS | 6.39E-06 | 8.37E-05 | CCNE1/CCNE2/CDK2/CDK4/CDK6/E2F1/E2F2/E2F3/TFDP1 | 9 | REACTOME |
| WP_TOLLLIKE_RECEPTOR_SIGNALING_RELATED_TO_MYD88 | 4.34E-06 | 8.59E-05 | IKBKE/IRF7/NFKB1/NFKB2/REL/RELB/TICAM1/TICAM2/TLR1/TLR2/TLR3/TLR4/TLR5/TLR6/TLR7/TLR8 | 16 | WP |
| PID_CD8_TCR_DOWNSTREAM_PATHWAY | 4.44E-06 | 8.72E-05 | B2M/CD247/CD3D/CD3E/CD3G/CD8A/CD8B/EGR1/EOMES/FASLG/FOS/FOSL1/GZMB/HLA-A/IL2RA/IL2RB/IL2RG/JUN/JUNB/NFATC2/PRF1/PRKCA/PTPN7/TNF/TNFRSF4 | 25 | PID |
| REACTOME_ASPARAGINE_N_LINKED_GLYCOSYLATION | 2.12E-06 | 8.79E-05 | ACTR10/ACTR1A/ALG2/ALG3/ALG5/ALG8/ANK1/ANK2/ANK3/ARF3/ARF4/ARFGAP2/ARFGAP3/B4GALT4/B4GALT6/BET1/CALR/CMAS/CNIH3/COG1/COPG1/COPZ1/DAD1/DCTN1/DDOST/DERL2/DPAGT1/DPM3/DYNC1H1/DYNC1I1/GANAB/GMPPA/GMPPB/KDELR1/KDELR2/LMAN2/MAN1B1/MAN2A2/MARCHF6/MGAT3/MGAT4B/MLEC/MOGS/MPDU1/NAPB/NAPG/NEU3/NSF/PDIA3/PRKCSH/RNF103/RPN1/RPN2/SEC13/SEC16B/SPTB/SPTBN1/SPTBN2/SPTBN4/ST6GAL2/ST6GALNAC1/ST6GALNAC5/ST6GALNAC6/ST8SIA3/ST8SIA6/STT3A/STX5/SYVN1/TFG/TMED10/TMED2/TMED3/TMED9/TMEM115/TRAPPC3/TRAPPC4/TRAPPC6B/TUBA4A/TUBA8/TUBB2A/TUSC3/UBA52/YKT6 | 83 | REACTOME |
| PID_MYC_ACTIV_PATHWAY | 7.59E-06 | 9.87E-05 | BCAT1/BIRC5/CAD/CCNB1/CDC25A/CDCA7/CDK4/DDX18/E2F3/EIF2S1/HMGA1/NCL/NME1/ODC1/POLR3D/PTMA/RCC1/RUVBL1/SMAD4/TK1/UBTF | 21 | PID |
| BIOCARTA_LAIR_PATHWAY | 5.23E-06 | 0.000101205 | C3/C7/CXCL8/ICAM1/IL1A/IL6/ITGAL/ITGB2/SELPLG/TNF/VCAM1 | 11 | BIOCARTA |
| WP_CELLS_AND_MOLECULES_INVOLVED_IN_LOCAL_ACUTE_INFLAMMATORY_RESPONSE | 5.23E-06 | 0.000101205 | C3/C7/CXCL8/ICAM1/IL1A/IL6/ITGAL/ITGB2/SELPLG/TNF/VCAM1 | 11 | WP |
| WP_COHESIN_COMPLEX_CORNELIA_DE_LANGE_SYNDROME | 8.04E-06 | 0.000104017 | AURKB/CDCA5/CDK1/ESCO1/ESCO2/ESPL1/HDAC8/PLK1/PTTG1/SGO1/SGO2/SMC1A/SMC3 | 13 | WP |
| REACTOME_CELLULAR_RESPONSE_TO_CHEMICAL_STRESS | 6.76E-07 | 0.000105518 | ATOX1/ATP7A/CCS/CHD9/COX11/COX14/COX18/COX4I1/COX5B/COX6B1/COX6C/COX7B/COX7C/COX8A/CREBBP/CSNK2A1/CSNK2A2/CUL3/HBA1/HBA2/HBB/LRPPRC/MED1/NCOA6/NCOR1/NCOR2/PPARA/PRDX1/PRDX5/PSMD1/PSMD12/PSMD2/PSME4/RBX1/RPS27A/SCO1/SIN3A/SURF1/TBL1X/TBL1XR1/TGS1/TXNRD2 | 42 | REACTOME |
| REACTOME_HOST_INTERACTIONS_OF_HIV_FACTORS | 8.42E-06 | 0.000108213 | HMGA1/KPNB1/NDC1/NUP107/NUP155/NUP160/NUP188/NUP205/NUP210/NUP35/NUP42/NUP43/NUP50/NUP85/NUP88/PAK2/PSMB2/PSMB7/PSMD11/PSMD14/PSMD3/PSMD5/PSMD6/PSME3/RAE1/RAN/RANBP1/RCC1/SKP1 | 29 | REACTOME |
| BIOCARTA_TH1TH2_PATHWAY | 6.04E-06 | 0.00011595 | CD28/CD40/CD40LG/CD86/HLA-DRA/HLA-DRB1/IFNGR1/IFNGR2/IL12RB1/IL18/IL18R1/IL2RA/IL4R | 13 | BIOCARTA |
| PID_AP1_PATHWAY | 6.12E-06 | 0.000116749 | ATF3/CCL2/CCN1/CDKN1B/CXCL8/DUSP1/EGR1/ELF1/FABP4/FOS/FOSB/FOSL1/FOSL2/HLA-A/IL10/IL6/JUN/JUNB/MAF/MMP1/MT2A/NFATC2/PLAU/TCF7L2/TGFB1/TIMP1 | 26 | PID |
| REACTOME_BETA_CATENIN_INDEPENDENT_WNT_SIGNALING | 2.98E-06 | 0.00012171 | AP2A1/AP2A2/AP2M1/CALM1/CAMK2A/CLTB/DVL1/FZD2/GNAO1/GNB1/GNB2/GNB5/GNG12/GNG3/GNG5/ITPR1/MOV10/NLK/PARD6A/PFN1/PLCB1/PLCB3/PPP3CA/PPP3CB/PPP3R1/PRICKLE1/PRKCB/PRKCG/PSMA2/PSMA3/PSMA4/PSMA7/PSMB1/PSMB3/PSMB4/PSMC2/PSMC4/PSMD13/PSMD9/PSME1/PSME2/PSMF1/RAC1/RHOA/RYK/SEM1/UBA52 | 47 | REACTOME |
| REACTOME_CLASS_A_1_RHODOPSIN_LIKE_RECEPTORS | 6.75E-06 | 0.000127848 | ADORA3/ADRB2/ANXA1/BDKRB2/C3/C3AR1/C5AR1/C5AR2/CCL2/CCL20/CCL3/CCL3L3/CCL4/CCL5/CCL7/CCR1/CCR2/CCR4/CCR5/CCR6/CCR7/CCRL2/CMKLR1/CXCL1/CXCL10/CXCL11/CXCL12/CXCL13/CXCL16/CXCL2/CXCL3/CXCL5/CXCL6/CXCL8/CXCL9/CXCR1/CXCR2/CXCR3/CXCR4/CXCR6/CYSLTR1/EDNRB/FFAR2/FFAR3/FFAR4/FPR1/FPR2/FPR3/GHRL/GPBAR1/GPR132/GPR18/GPR183/GPR37L1/GPR65/HCAR2/HCAR3/HRH1/HTR2B/HTR7/LHB/LPAR5/LPAR6/OPN3/P2RY10/P2RY13/P2RY6/PPBP/PROK2/PSAP/PTAFR/PTGER1/PTGER2/PTGER4/PTGFR/S1PR4/SAA1/SUCNR1/UTS2/XCL1/XCL2 | 81 | REACTOME |
| REACTOME_TP53_REGULATES_TRANSCRIPTION_OF_GENES_INVOLVED_IN_G1_CELL_CYCLE_ARREST | 1.02E-05 | 0.00013012 | CCNA2/CCNE1/CCNE2/CDK2/E2F1/E2F7/E2F8/ZNF385A | 8 | REACTOME |
| REACTOME_CELLULAR_SENESCENCE | 1.05E-05 | 0.000132771 | CBX2/CBX8/CCNA2/CCNE1/CCNE2/CDC23/CDK2/CDK4/CDK6/CDKN2C/E2F1/E2F2/E2F3/EHMT1/EZH2/FZR1/H1-3/H2AX/H2AZ1/H2BC17/H2BC9/H3-3A/H3C2/H3C3/HMGA1/KDM6B/LMNB1/MAP3K5/MAPKAPK5/MRE11/RBBP4/RBBP7/RING1/RNF2/SUZ12/TFDP1/UBE2C/UBE2S | 38 | REACTOME |
| REACTOME_TRANSPORT_OF_SMALL_MOLECULES | 3.39E-06 | 0.000136257 | ABCA1/ABCA5/ABCA9/ABCB1/ABCB9/ABCD1/ABCD2/ABCG2/ABCG4/ADCY1/ADCY2/ADCY5/ADD3/ANO3/ANO5/ANO7/AP2A1/AP2A2/AP2M1/AQP11/AQP3/ASIC2/ATP10A/ATP13A2/ATP1A2/ATP1A3/ATP1B1/ATP2B1/ATP2B2/ATP2B3/ATP6V0A1/ATP6V0C/ATP6V0D1/ATP6V0E1/ATP6V1A/ATP6V1B2/ATP6V1C1/ATP6V1D/ATP6V1E1/ATP6V1G2/ATP6V1H/ATP7B/ATP8A2/ATP9A/CA4/CALM1/CAMK2A/CAMK2B/CAMK2G/CASQ1/CASQ2/CLCN4/CLCN6/CTNS/CYGB/DERL2/DMTN/EIF2S3/FKBP1B/FXYD7/GNB1/GNB2/GNB5/GNG12/GNG3/GNG5/HMOX2/KCNJ11/LCN12/LPL/LRRC8B/LSR/MICU3/NALCN/NCEH1/NEDD4L/NGB/NIPAL2/P4HB/PHB2/PRKACB/PRKAR1A/PRKAR1B/PRKAR2B/PSMA2/PSMA3/PSMA4/PSMA7/PSMB1/PSMB3/PSMB4/PSMC2/PSMC4/PSMD13/PSMD9/PSME1/PSME2/PSMF1/RAB11FIP2/RHCG/RIPK1/RYR1/RYR2/SEM1/SLC12A4/SLC12A5/SLC13A3/SLC13A5/SLC14A1/SLC16A1/SLC16A7/SLC16A8/SLC17A7/SLC1A2/SLC1A3/SLC1A4/SLC22A17/SLC22A3/SLC24A4/SLC25A18/SLC25A22/SLC25A4/SLC26A2/SLC26A4/SLC27A4/SLC2A10/SLC2A11/SLC2A12/SLC2A13/SLC2A6/SLC30A10/SLC30A3/SLC30A5/SLC30A7/SLC32A1/SLC33A1/SLC35A2/SLC35B3/SLC35D2/SLC39A1/SLC39A10/SLC39A7/SLC4A10/SLC4A2/SLC5A4/SLC66A1/SLC6A1/SLC6A12/SLC6A13/SLC6A15/SLC7A10/SLC8A1/SLC8A2/SLC9A5/SLC9A6/SLC9A7/SLC9B2/SLCO1C1/STEAP2/TRPC1/TRPM7/TUSC3/UBA52/UNC79/UNC80/VDAC3/WNK2/WWP1 | 168 | REACTOME |
| WP_ADIPOGENESIS | 7.33E-06 | 0.00013789 | AGPAT2/ASIP/CDKN1A/CEBPA/CEBPB/CEBPD/CFD/CNTFR/EGR2/FAS/GADD45A/GADD45B/ID3/IGF1/IL6/KLF15/KLF5/LIF/MBNL1/NAMPT/NCOA1/NR1H3/OSM/PCK2/PLIN2/PPARD/PPARG/PPARGC1A/RETN/SERPINE1/SFRP4/SOCS1/SOCS3/STAT2/STAT5A/STAT6/TGFB1/TNF/TWIST1/WWTR1 | 40 | WP |
| PID_CXCR4_PATHWAY | 7.39E-06 | 0.000138151 | ARRB2/CD247/CD3D/CD3E/CD3G/CD4/CSK/CXCL12/CXCR4/FGR/FYN/HCK/HLA-DRA/ITGA11/ITGA2/JAK2/LCK/LYN/PIK3CD/PIK3CG/PIK3R5/PIK3R6/PLCB2/PTK2/PTPN11/PTPN6/PTPRC/RGS1/SSH1/STAT2/STAT5A/VAV1/VPS4A | 33 | PID |
| PID_AURORA_A_PATHWAY | 1.10E-05 | 0.00013837 | AURKA/AURKB/BIRC5/BRCA1/CDC25B/CENPA/DLGAP5/FZR1/RAN/TACC1/TACC3/TPX2 | 12 | PID |
| WP_RETT_SYNDROME_CAUSING_GENES | 3.56E-06 | 0.000140997 | ACTL6B/CDKL5/EIF2B2/GABBR2/GABRD/GNAO1/GRIN2A/GRIN2B/HDAC1/HDAC5/HIVEP2/HTT/IMPDH2/MEF2C/RHOBTB2/SCN2A/SCN8A/SHANK3/SMARCA2/SRRM3/STXBP1/SYNGAP1 | 22 | WP |
| REACTOME_ORGANELLE_BIOGENESIS_AND_MAINTENANCE | 9.64E-07 | 0.000141561 | AKAP9/ALMS1/APOOL/ASAP1/ATF2/ATP5F1D/ATP5F1E/ATP5MC1/ATP5MC2/ATP5MC3/ATP5ME/ATP5MF/ATP5MG/ATP5PD/BBS10/BBS9/C2CD3/CDK5RAP2/CEP135/CEP152/CEP162/CEP192/CEP250/CEP57/CEP70/CHD9/CKAP5/CLASP1/CNTRL/CREB1/CREBBP/CRTC3/DNAJC11/DYNC1I2/DYNLL1/DYNLRB1/EXOC1/EXOC2/EXOC4/EXOC5/EXOC7/EXOC8/GABPA/HCFC1/IFT81/MAPK14/MED1/MICOS10/MICOS13/NCOA6/NCOR1/PCM1/PKD1/PPARA/RPGRIP1L/SEPTIN2/TBL1X/TBL1XR1/TFAM/TGS1/TNPO1/TRIP11/TTC21B/UNC119B/WDR35 | 65 | REACTOME |
| REACTOME_MITOCHONDRIAL_BIOGENESIS | 1.06E-06 | 0.000146909 | APOOL/ATF2/ATP5F1D/ATP5F1E/ATP5MC1/ATP5MC2/ATP5MC3/ATP5ME/ATP5MF/ATP5MG/ATP5PD/CHD9/CREB1/CREBBP/CRTC3/DNAJC11/GABPA/HCFC1/MAPK14/MED1/MICOS10/MICOS13/NCOA6/NCOR1/PPARA/TBL1X/TBL1XR1/TFAM/TGS1 | 29 | REACTOME |
| KEGG_OOCYTE_MEIOSIS | 1.17E-05 | 0.000147462 | AURKA/BUB1/CCNB1/CCNB2/CCNE1/CCNE2/CDC20/CDC23/CDC25C/CDK1/CDK2/CHP1/ESPL1/FBXO43/FBXO5/MAD2L1/MAD2L2/PKMYT1/PLK1/PPP1CC/PTTG1/SGO1/SKP1/SMC1A/SMC3/YWHAQ | 26 | KEGG |
| WP_NONGENOMIC_ACTIONS_OF_125_DIHYDROXYVITAMIN_D3 | 8.26E-06 | 0.000153376 | CAMP/CCL2/CD40/CD40LG/CXCL8/IL6/JUN/MAPK13/NFKB1/NFKB2/NOD2/OAS2/PLCB2/PLCG2/PRKCA/PRKCD/PRKCH/RELB/RSAD2/STAT2/TLR2/TLR4/TLR8/TNF/TNFRSF1A/VDR | 26 | WP |
| REACTOME_DAP12_INTERACTIONS | 8.67E-06 | 0.000158697 | B2M/BTK/CD300E/CD300LB/CLEC5A/FYN/GRAP2/GRB2/HLA-B/HLA-C/HLA-E/LCK/LCP2/PLCG2/SIRPB1/SYK/TREM1/TREM2/TYROBP | 19 | REACTOME |
| WP_FIBRIN_COMPLEMENT_RECEPTOR_3_SIGNALING_PATHWAY | 8.67E-06 | 0.000158697 | CCL2/CD14/CXCL10/CXCL3/IL6/IRAK2/ITGAM/ITGB2/LY96/NFKB1/RASSF5/REL/SYK/TICAM1/TICAM2/TLR3/TLR4/TNF/TYROBP | 19 | WP |
| REACTOME_UNBLOCKING_OF_NMDA_RECEPTORS_GLUTAMATE_BINDING_AND_ACTIVATION | 4.12E-06 | 0.000160767 | CALM1/CAMK2A/CAMK2B/CAMK2G/DLG2/DLG3/DLG4/GRIN1/GRIN2A/GRIN2B/GRIN2C/LRRC7/NEFL | 13 | REACTOME |
| REACTOME_NUCLEOTIDE_EXCISION_REPAIR | 1.28E-06 | 0.000168582 | ACTR8/AQR/CUL4B/DDB1/EP300/ERCC4/ERCC6/ERCC8/GTF2H2/GTF2H3/INO80/INO80D/LIG3/NFRKB/PIAS1/PIAS3/POLK/POLR2A/POLR2B/POLR2C/POLR2E/POLR2I/RBX1/RFC1/RNF111/RPS27A/TFPT/USP45/USP7/UVSSA/XPC/YY1 | 32 | REACTOME |
| PID_FRA_PATHWAY | 9.37E-06 | 0.000169647 | CCL2/CXCL8/DCN/FOSL1/FOSL2/HMOX1/IL6/ITGB4/JUN/JUNB/LIF/MGP/MMP1/NFATC2/PLAU/PLAUR/THBD | 17 | PID |
| REACTOME_TNFR2_NON_CANONICAL_NF_KB_PATHWAY | 9.39E-06 | 0.000169647 | BIRC3/CD27/CD40/CD40LG/CD70/FASLG/FBXW11/LTA/LTB/LTBR/NFKB2/PSMA1/PSMA5/PSMB10/PSMB8/PSMB9/RELB/TNF/TNFRSF11A/TNFRSF11B/TNFRSF12A/TNFRSF14/TNFRSF1A/TNFRSF1B/TNFRSF4/TNFRSF6B/TNFRSF8/TNFSF13/TNFSF13B/TNFSF14/TNFSF15/TNFSF4/TNFSF8 | 33 | REACTOME |
| BIOCARTA_ASBCELL_PATHWAY | 9.75E-06 | 0.000171554 | CD28/CD4/CD40/CD40LG/CD80/FAS/FASLG/HLA-DRA/HLA-DRB1/IL10 | 10 | BIOCARTA |
| BIOCARTA_GRANULOCYTES_PATHWAY | 9.75E-06 | 0.000171554 | CSF3/CXCL8/ICAM1/IL1A/ITGAL/ITGAM/ITGB2/SELL/SELPLG/TNF | 10 | BIOCARTA |
| BIOCARTA_IL17_PATHWAY | 9.75E-06 | 0.000171554 | CD2/CD247/CD3D/CD3E/CD3G/CD4/CD8A/CSF3/CXCL8/IL6 | 10 | BIOCARTA |
| BIOCARTA_NO2IL12_PATHWAY | 9.75E-06 | 0.000171554 | CCR5/CD2/CD247/CD3D/CD3E/CD3G/CD4/CXCR3/IL12RB1/JAK2 | 10 | BIOCARTA |
| WP_GABA_RECEPTOR_SIGNALING | 4.54E-06 | 0.000174567 | AP2A1/AP2A2/AP2M1/GABBR1/GABBR2/GABRA1/GABRA2/GABRA4/GABRA5/GABRB1/GABRB2/GABRB3/GABRD/GABRG1/GAD2/SLC32A1/SLC6A1 | 17 | WP |
| PID_S1P_S1P2_PATHWAY | 4.69E-06 | 0.000177863 | ELK1/GNA13/GNAI1/GNAI2/GNAI3/GNAO1/GNAQ/GNAZ/MAPK1/MAPK3/PAK1/RAC1/RHOA/S1PR2 | 14 | PID |
| REACTOME_DNA_REPAIR | 1.43E-06 | 0.000178547 | ABRAXAS1/ACTR8/AQR/ASCC3/ATM/ATR/BABAM1/BAZ1B/BLM/BRCC3/CUL4B/DCLRE1A/DCLRE1C/DDB1/EP300/ERCC4/ERCC6/ERCC8/EYA3/FAN1/GTF2H2/GTF2H3/H2BC7/H4-16/HERC2/INO80/INO80D/KDM4A/LIG3/MDC1/MGMT/MPG/MSH3/NBN/NFRKB/OGG1/PALB2/PARG/PIAS1/PIAS3/PMS2/POLK/POLL/POLR2A/POLR2B/POLR2C/POLR2E/POLR2I/PRKDC/RAD17/RAD50/RAD51B/RBX1/REV1/REV3L/RFC1/RIF1/RNF111/RPS27A/SLX4/SMARCA5/TFPT/TP53BP1/UBXN1/USP10/USP45/USP7/UVSSA/XPC/YY1 | 70 | REACTOME |
| REACTOME_CDC6_ASSOCIATION_WITH_THE_ORC_ORIGIN_COMPLEX | 1.44E-05 | 0.000179371 | CDC6/E2F1/E2F2/E2F3/MCM8/ORC1/ORC6 | 7 | REACTOME |
| PID_THROMBIN_PAR1_PATHWAY | 5.05E-06 | 0.000186433 | ARRB1/DNM1/F2R/GNA13/GNAI1/GNAI2/GNAI3/GNAO1/GNAQ/GNAZ/GNB1/GRK3/PIK3R1/PKN1/PLCB1/PLCB3/PRKCB/PRKCG/RHOA/ZYX | 20 | PID |
| REACTOME_GABA_B_RECEPTOR_ACTIVATION | 5.05E-06 | 0.000186433 | ADCY1/ADCY2/ADCY5/GABBR1/GABBR2/GNAI1/GNAI2/GNAI3/GNAL/GNB1/GNB2/GNB5/GNG12/GNG3/GNG5/KCNJ12/KCNJ3/KCNJ4/KCNJ6/KCNJ9 | 20 | REACTOME |
| REACTOME_RECRUITMENT_OF_NUMA_TO_MITOTIC_CENTROSOMES | 1.54E-05 | 0.000190585 | CDK1/CENPJ/CEP131/CEP41/CEP72/CEP76/DCTN3/HAUS1/HAUS2/HAUS3/HAUS6/HAUS8/NDE1/NEDD1/NEK2/ODF2/PLK1/PLK4/TUBA1A/TUBA1B/TUBB/TUBG1/TUBGCP3 | 23 | REACTOME |
| REACTOME_REGULATED_NECROSIS | 1.11E-05 | 0.000193296 | BIRC3/CASP1/CASP4/CASP5/CASP8/CFLAR/CHMP4C/FAS/FASLG/FLOT1/GSDMD/GZMB/IL18/IL1A/IL1B/IRF1/IRF2/MLKL/RIPK3/TNFRSF10A/TNFSF10/TRADD | 22 | REACTOME |
| BIOCARTA_CK1_PATHWAY | 5.32E-06 | 0.000193851 | CDK5R1/DRD1/GRM1/PLCB1/PPP1R1B/PPP2CA/PPP3CA/PRKACB/PRKAR1A/PRKAR1B/PRKAR2B | 11 | BIOCARTA |
| WP_GENES_ASSOCIATED_WITH_THE_DEVELOPMENT_OF_RHEUMATOID_ARTHRITIS | 1.16E-05 | 0.000201347 | CCR6/CD244/CD40/CIITA/CTLA4/HLA-DRB1/IL2RA/IRF5/PTPN22/SLC22A4/TRAF1 | 11 | WP |
| PID_IL8_CXCR2_PATHWAY | 1.17E-05 | 0.000202692 | ARRB2/CXCL8/CXCR2/DOCK2/FGR/GNA15/HCK/LYN/PIK3CG/PIK3R6/PLCB2/PLD2/PPP2R1A/PRKCA/RAC2/VASP | 16 | PID |
| REACTOME_PHOSPHOLIPID_METABOLISM | 6.05E-06 | 0.000217339 | ABHD4/ACP6/AGPAT3/ARF3/CDS1/CDS2/CPNE1/CPNE6/CPNE7/CRLS1/CSNK2B/DDHD2/ETNPPL/FIG4/GDPD5/GPAM/GPAT4/GPD1L/INPP4A/INPP4B/INPP5F/INPP5J/INPPL1/LPCAT3/LPCAT4/LPGAT1/LPIN1/LPIN2/MGLL/MIGA1/MTMR12/MTMR6/MTMR7/MTMR9/PCYT1A/PI4KA/PIK3CB/PIK3R1/PIP4K2B/PIP4K2C/PIP5K1B/PIP5K1C/PISD/PITPNM2/PITPNM3/PLA1A/PLA2G12A/PLAAT5/PLD6/PLEKHA1/PLEKHA4/PLEKHA5/PLEKHA6/PTDSS1/RAB4A/RUFY1/SACM1L/SBF1/SELENOI/STARD10/SYNJ1 | 61 | REACTOME |
| HALLMARK_OXIDATIVE_PHOSPHORYLATION | 1.89E-06 | 0.000219219 | ABCB7/ATP5F1D/ATP5F1E/ATP5MC1/ATP5MC2/ATP5MC3/ATP5ME/ATP5MF/ATP5MG/ATP5PD/ATP6V1F/COX11/COX17/COX4I1/COX5B/COX6B1/COX6C/COX7B/COX7C/COX8A/DLAT/DLD/ECH1/ECHS1/ETFB/GPX4/LRPPRC/MRPL34/MRPS30/NDUFA1/NDUFA2/NDUFA3/NDUFA7/NDUFA8/NDUFB1/NDUFB2/NDUFB3/NDUFB4/NDUFB7/NDUFS7/NDUFS8/NDUFV1/SURF1/TIMM10/UQCR10/UQCR11/UQCRH/UQCRQ | 48 | HALLMARK |
| KEGG_ALZHEIMERS_DISEASE | 1.93E-06 | 0.000219219 | ADAM17/APAF1/ATF6/ATP2A2/ATP5F1D/ATP5F1E/ATP5MC1/ATP5MC2/ATP5MC3/ATP5PD/BAD/COX4I1/COX5B/COX6B1/COX6C/COX7B/COX7C/COX8A/EIF2AK3/GSK3B/IDE/ITPR2/NAE1/NDUFA1/NDUFA2/NDUFA3/NDUFA7/NDUFA8/NDUFB1/NDUFB10/NDUFB2/NDUFB3/NDUFB4/NDUFB7/NDUFS5/NDUFS7/NDUFS8/NDUFV1/UQCR10/UQCR11/UQCRH/UQCRQ | 42 | KEGG |
| PID_NFAT_TFPATHWAY | 1.29E-05 | 0.000220724 | CD40LG/CTLA4/CXCL8/EGR1/EGR2/FASLG/FOS/FOSL1/FOXP3/GBP3/IKZF1/IL2RA/JUN/JUNB/MAF/NFATC2/PPARG/PTGS2/TNF | 19 | PID |
| REACTOME_HCMV_EARLY_EVENTS | 1.80E-05 | 0.000222299 | EZH2/H2AC11/H2AC13/H2AC16/H2AC17/H2BC17/H2BC9/H3C2/H3C3/NDC1/NUP107/NUP155/NUP160/NUP188/NUP205/NUP210/NUP35/NUP42/NUP43/NUP50/NUP85/NUP88/RAE1/RBBP4/RBBP7/SUZ12/TRIM28/TUBA1A/TUBA1B | 29 | REACTOME |
| BIOCARTA_NEUTROPHIL_PATHWAY | 1.34E-05 | 0.000228134 | CD44/ICAM1/ITGAL/ITGAM/ITGB2/SELE/SELL | 7 | BIOCARTA |
| REACTOME_EPH_EPHRIN_SIGNALING | 6.82E-06 | 0.000241833 | ACTG1/AP2A1/AP2A2/AP2M1/APH1A/ARHGEF28/ARPC1A/CLTB/DNM1/EFNA1/EFNA3/EFNA5/EFNB3/EPHA10/EPHA4/EPHA5/EPHA7/EPHB6/GIT1/GRIN1/GRIN2B/ITSN1/KALRN/MYH11/MYL6/NCSTN/NGEF/PAK1/PAK3/PSENEN/RAC1/RHOA/TIAM1 | 33 | REACTOME |
| WP_PROSTAGLANDIN_AND_LEUKOTRIENE_METABOLISM_IN_SENESCENCE | 1.45E-05 | 0.000244962 | ALOX15B/ALOX5/ALOX5AP/CDKN1A/CYSLTR1/GNAS/LTA4H/LTC4S/PTGER1/PTGER2/PTGER4/PTGES/PTGS1/PTGS2/TBXAS1 | 15 | WP |
| REACTOME_FCGR_ACTIVATION | 1.52E-05 | 0.000256053 | CD247/CD3G/FCGR1A/FCGR2A/FCGR3A/FGR/FYN/HCK/IGHG1/IGHG2/IGHG4/IGKV1-5/IGKV1D-39/IGKV3-11/IGKV3-15/IGKV3-20/IGKV4-1/IGLC2/IGLC3/IGLV1-40/IGLV1-44/IGLV2-14/IGLV2-8/LYN/SYK | 25 | REACTOME |
| WP_MAPK_SIGNALING_PATHWAY | 7.71E-06 | 0.000269859 | ARAF/ARRB1/ATF4/CACNA1A/CACNA1C/CACNA1D/CACNA1E/CACNA1G/CACNA1I/CACNA2D1/CACNA2D2/CACNA2D3/CACNB1/CACNB2/CACNB3/CACNB4/CACNG3/CACNG8/CASP3/DAXX/DUSP10/DUSP6/DUSP8/DUSP9/ELK1/FGF12/FGF13/FGF17/FGF18/FGF22/FGF9/FGFR3/GNG12/IKBKB/MAP2K1/MAP2K4/MAP3K14/MAP4K2/MAPK1/MAPK10/MAPK3/MAPK7/MAPK8IP1/MAPK8IP2/MAPK8IP3/MAPK9/MAPKAPK2/MEF2C/MRAS/NLK/NTRK2/PAK1/PPM1A/PPP3CA/PPP3CB/PPP3R1/PRKACB/PRKCG/PTPN5/PTPRR/RAC1/RAP1B/RAPGEF2/RASGRF2/RASGRP1/RELA/RPS6KA5/STK3/TP53 | 69 | WP |
| REACTOME_ASSEMBLY_AND_CELL_SURFACE_PRESENTATION_OF_NMDA_RECEPTORS | 7.81E-06 | 0.000269859 | APBA1/CAMK2A/CAMK2B/CAMK2G/DLG2/DLG3/DLG4/GRIN1/GRIN2A/GRIN2B/GRIN2C/GRIN3A/KIF17/LIN7B/LRRC7/NBEA/NEFL/TUBA4A/TUBA8/TUBB2A | 20 | REACTOME |
| WP_GASTRIC_CANCER_NETWORK_2 | 2.31E-05 | 0.000281616 | ATAD2/CEBPZ/CHTF18/DSCC1/FANCI/LBR/LMNB2/RFC3/RFC4/TOP2A/UBE2C/UBE2T | 12 | WP |
| WP_TUMOR_SUPPRESSOR_ACTIVITY_OF_SMARCB1 | 2.31E-05 | 0.000281616 | CDK4/CDK6/DPF2/EZH2/H3-3A/RBBP4/SMARCA4/SMARCB1/SMARCC1/SMARCD1/SMARCE1/SUZ12 | 12 | WP |
| REACTOME_TRAFFICKING_OF_AMPA_RECEPTORS | 8.41E-06 | 0.00028683 | AKAP5/AP2A1/AP2A2/AP2M1/CACNG3/CACNG8/CAMK2A/CAMK2B/CAMK2G/DLG4/EPB41L1/GRIP1/GRIP2/NSF/PRKCB/PRKCG | 16 | REACTOME |
[truncated: 107,028 more chars]
